# Supplementary material for: LipidCreator workbench to probe the lipidomic landscape
Source: Nat Commun. 2020 Apr 28;11:2057. doi: 10.1038/s41467-020-15960-z (PMC7188904; doi:10.1038/s41467-020-15960-z)
Supplement: Supplementary file 1 — Supplementary Information [file 41467_2020_15960_MOESM1_ESM.pdf]

Supplementary Information for

LipidCreator workbench to probe the lipidomic landscape

Bing Peng[1,2,#], Dominik Kopczynski[1,#], Brian S Pratt[3], Christer S Ejsing[4,5], Bo Burla[6], Martin Hermansson[4,7], Peter Imre Benke[8], Sock Hwee Tan[9,10], Mark Y Chan[9,10,11], Federico Torta[8], Dominik Schwudke[12,13,14], Sven Meckelmann[15], Cristina Coman[1,17], Oliver J Schmitz[15], Brendan MacLean[3], Mailin-Christin Manke[16], Oliver Borst[16], Markus R Wenk[6,8], Nils Hoffmann[1], Robert Ahrends[1,17,*]

[1] Leibniz-Institut für Analytische Wissenschaften – ISAS - e.V., 44139 Dortmund, Germany
[2] Division of Rheumatology, Department of Medicine Solna, Karolinska Institutet, Karolinska University Hospital, SE-171 76 Stockholm, Sweden
[3] University of Washington, Department of Genome Sciences, WA 98195 Seattle, USA
[4] Department of Biochemistry and Molecular Biology, University of Southern Denmark, DK-5230 Odense, Denmark
[5] Cell Biology and Biophysics Unit, European Molecular Biology Laboratory, 69117 Heidelberg, Germany
[6] Singapore Lipidomics Incubator (SLING), Life Science Institute, National University of Singapore, 117456 Singapore, Singapore
[7] Wihuri Research Institute, 00290 Helsinki, Finland
[8] Singapore Lipidomics Incubator (SLING), Department of Biochemistry, Yong Loo Lin School of Medicine, National University of Singapore, 117596 Singapore, Singapore
[9] Department of Medicine, Yong Loo Lin School of Medicine, National University Hospital, 119228 Singapore, Singapore
[10] Cardiovascular Research Institute, National University of Singapore, 117599 Singapore, Singapore
[11] National University Heart Centre, National University Health System, 117599 Singapore, Singapore
[12] Research Center Borstel, Leibniz Lung Center, Borstel, Germany
[13] German Center for Infection Research (DZIF), 38124 Braunschweig, Germany
[14] Airway Research Center North Member of the German Center for Lung Research (DZL), 22927 Großhansdorf, Germany
[15] Institute of Applied Analytical Chemistry, University of Duisburg-Essen, 45141 Essen, Germany
[16] Department of Cardiology and Cardiovascular Medicine, University of Tübingen, 72076 Tübingen, Germany
[17] Department of Analytical Chemistry, University of Vienna, Währinger Strasse 38, 1090 Vienna, Austria
[#] Two authors contributed equally to this work
[*] Corresponding author

Table of Contents

Supplementary Methods 4
- Chemicals 4
- Preparation of samples 5
- Platelet isolation and stimulation 5
- Plasma collection 5
- Yeast culture 5
- Ethics 5

Lipid extraction 6
Platelets 6
Plasma 6
Yeast 7
LC-MS/MS 7
Targeted LC-MS/MS analysis of phospholipids and glycerolipids 7
Targeted analysis of mediators 8
Targeted analysis of plasma samples 8
Targeted analysis of yeast samples 9
Direct infusion of lipid standards on QEx HF and Q-TOF 9
Supplementary Note 1 10
LipidCreator design and architecture - Collision Energy Optimization Dialog 10
Validation of LipidCreator 11
Validation of lipid identification and quantification with calibration curves 11
Applicability on a variety of diverse organisms 12
Verification of lipid mediators using the *in silico* spectral library from LipidCreator 13
Human plasma comparison to NIST SRM 1950 and Bowden ring trial 16
Data analysis and repository information 17
Supplementary Note 2 18
Manual for LipidCreator 18
An Overview of the LipidCreator Workflow 18
Using the LipidCreator Wizard 20
Overview of the LipidCreator User Interface 24
Generating lipid molecule transitions 25
Import/export lipid list/setting/project 35
Lipid name translator 36
Selection of MS2 fragments 37
Adding user-defined fragments 40
Managing heavy isotopes 44
Filters for the transition list 47
Collision energy optimization function 48

Reviewing the lipid transition list 50
Storing a transition list 53
Storing a spectral library 53
Integration with Skyline 53
Statistics for LipidCreator launching 55
Command line usage 56
Support for additional platforms for CE Optimization 59
Supplementary Note 3 65
Modeling results for lipid mediators 65
Exemplary Results for 10-HDoHE Mediator 65
Global Comparison of non-linear fit results 72
Supplementary Tables 74
Supplementary References 79

Supplementary Methods

Chemicals

Formic acid, tert-butyl methyl ether (MTBE), ammonium formate, ammonium acetate, acetate acid (HAc), sodium chloride, sodium bicarbonate, potassium chloride, glucose, disodium phosphate, HEPES and calcium chloride were purchased from Sigma Aldrich (Steinheim, Germany). The ULC/MS-grade solvents, acetonitrile (ACN), methanol (MeOH) were obtained from Biosolve (Valkenswaard, Netherlands) and isopropanol (IPA) was purchased from Merck (Darmstadt, Germany). Ultrapure water (18 MΩ cm at 25 °C) was used to prepare solutions. Sodium dodecyl sulfate (SDS) was obtained from Roth (Karlsruhe, Germany), tris(hydroxymethyl)-aminomethane (Tris) from Applichem (Darmstadt, Germany), and sodium chloride (NaCl) from Merck (Darmstadt, Germany). Platelets were activated using collagen-related peptide (Richard Farndale, University of Cambridge, United Kingdom) or thrombin from human plasma (Roche, Germany). SPLASH Lipidomics Mass Spec Standard (330707) was purchased from Avanti (Alabaster, AL, USA). Thromboxane B2-d4, 12-HETE-d8, Arachidonic Acid-d8, Prostaglandin D2-d4, Prostaglandin E2-d4, 15-HETE-d8, 13-HODE-d4, DHA-d5, and EPA-d5 were purchased from Cayman Chemical (Ann Arbor, Michigan, USA) and used as internal standards.

136 lipid standards were used to study the lipid fragmentation, build optimal collision energy model and create *in silico* spectral library. Lipid fragment masses of mediators were validated with METLIN[46] database (https://metlin.scripps.edu/). 10-HDoHE, 11(12)-EET-d11, 11,12-DHET-d11, 11-HDoHE, 11-HETE, 12(13)-EpOME-d4, 12-HEPE, 12-HETE-d8, 12-HHTrE, 12-OxoETE, 13-HODE-d4, 13-HOTrE, 14(15)-EET-d11, 14(15)-EpETE, 14,15-DHET-d11, 15d-PGJ2-d4, 15-HEPE, 15-HETE-d8, 15R-LXA4, 16-HDoHE, 16-HETE, 18-HEPE, 5(6)-EET-d11, 5,12-DiHETE, 5,6-DiHETE, 5-HEPE, 5-HETE-d8, 5-HpETE, 5-OxoETE-d7, 8(9)-EET-d11, 8,9-DHET-d11, 8-HDoHE, 8-HETE, 8-iso-PGF2a, 9(10)-EpOME-d4, 9-HEPE, 9-HETE, 9-HODE, 9-HOTrE, AA-d8, alpha-LA-d14, DHA-d5, EPA-d5, LA-d11, LA-d4, LTB4-d4, LTC4-d5, LTD4-d5, Maresin 1, PA-d2, PGB2-d4, PGD2-d4, PGE2-d4, PGE2-d9, PGF2alpha-d4, PGI2, Resolvin D1-d5, Resolvin D2-d5, Resolvin D3, Resolvin D5, tetranor-12-HETE, TXB1, TXB2-d4, and TXB3 were purchased from Cayman Chemical (Ann Arbor, Michigan, USA). BMP 18:1-18:1, CDPDAG 18:1-18:1, Cer 18:0;2/12:0, Cer 18:1;2/12:0, Cer 18:1;2/17:0;1, CerP 18:0;2/16:0, CerP 18:1;2/12:0, Ch-d7, ChE 17:0, ChE 18:1-d7, ChE 22:0, CL 14:1-14:1-14:1-15:1, CL 14:1-22:1-22:1-22:1, CL 14:1-24:1-24:1-24:1, CL 15:0-15:0-15:0-16:1, CL 18:1-18:1-18:1-18:1, DAG 10:0-10:0, DAG 15:0-18:1-d7, DAG 16:0-16:0, DMPE 18:1-18:1, Hex2Cer 18:1;2/12:0, HexCer 18:1;2/12:0, LCB 17:0;2, LCB 17:1;2, LCBP 17:0;2, LCBP 17:1;2, LPA 17:1, LPC 13:0, LPC 18:1-d7, LPC 19:0, LPE 17:1, LPE 18:1-d7, LPG 17:1, LPI 17:1, LPS 17:1, LSM 17:1;2, MAG 16:0, MAG 18:1-d7, MMPE 18:1-18:1, PA 14:1-17:0, PA 15:0-18:1-d7, PA 17:0-20:4, PC 14:1-17:0, PC 15:0-18:1-d7, PC 17:0-20:4, PC 21:0-22:6, PE 14:1-17:0, PE 15:0-18:1-d7, PE 17:0-17:0, PE 21:0-22:6, PEt 16:0-18:1, PG 14:1-17:0, PG 15:0-18:1-d7, PG 17:0-20:4, PI 14:1-17:0, PI 15:0-18:1-d7, PI 21:0-22:6, PIP 17:0-20:4, PIP2 17:0-20:4, PIP3 17:0-20:4, PC O 18:1p-20:4, PS 12:0-13:0, PS 14:1-17:0, PS 15:0-18:1-d7, PS 17:0-20:4, SHexCer 18:1;2/12:0, SM 18:0;2/12:0, SM 18:1;2/12:0, SM 18:1;2/18:1-d7, TAG 15:0-15:0-18:1-d7, TAG 16:0-16:0-16:0, and TAG 17:0-17:0-17:1-d5 were purchased from Avanti (Alabaster, AL, USA).

Preparation of samples

Platelet isolation and stimulation

Blood from five individual healthy volunteers (about 32-48 mL per person) was collected in ACD-buffer and centrifuged at 200 g for 20 min. The obtained platelet-rich plasma was added to modified Tyrode-HEPES (N-2-hydroxyethyl-piperazone-N´2-ethanesulfonic acid) buffer (137 mM NaCl, 2 mM KCl, 12 mM $NaHCO_3$, 5 mM glucose, 0.3 mM Na_2HPO_4, 10 mM HEPES, pH 6.5). After centrifugation at 900g for 10 minutes and removal of the supernatant, the resulting platelet pellet was resuspended in Tyrode-HEPES buffer (pH 7.4, supplemented with 1 mM $CaCl_2$).

Freshly isolated and resuspended human platelets were stimulated with 0.01 U per mL thrombin, 1 U per mL thrombin, 1 µg per mL CRP or 5 µg per mL CRP for 5 min. After centrifugation for 5 min at 640 g at 25°C, the pellet and supernatant were separated and separately shock frozen in liquid nitrogen.

Plasma collection

Blood from 21 healthy individuals (12 males, 9 females; 22 - 44 years old) was obtained by venipuncture into K3EDTA BD Vacutainer tubes. Platelet poor plasma was collected after centrifugation for 10 min at 3,850 g (4 °C) and stored at −80 °C until analysis. The NIST Standard Reference Material for Human Plasma (SRM1950) was purchased from the National Institute for Standards and Technology (Gaithersburg, MD, USA).

Yeast culture

S. cerevisiae cells isogenic to BY4742 (MATα his3Δ1 leu2Δ0 lys2Δ0 ura3Δ0) were cultured in YPD medium (20 g per l yeast extract, 20 g per l peptone, 10 g per l D-glucose) at 30 °C to exponential phase. Next, 5 OD units of cells were collected, washed with 155 mM NH_4HCO_3 (pH 8) in water and the cell pellets stored at -80 °C.

Ethics

All volunteers gave informed consent for blood samples. The platelet study was approved by the institutional ethics committee (270/2011BO1) at University Hospital Tübingen (Germany) and complies with the declaration of Helsinki and good clinical practice guidelines.

The collection and use of human plasma samples has been approved by the Institutional Review Board of the National University of Singapore (NUS-IRB N-17-082E and NUS-IRB B-15-094, Singapore).

Lipid extraction

Platelets

Lipid (except fatty acids and their derivatives) extraction was carried out according to Matyash *et al.*[1] with small modifications. In brief, 225 µL of MeOH (4 °C) were added to platelet cell pellet or platelet supernatant in an Eppendorf polypropylene tube that was placed on ice. After a few seconds of treatment with ultrasonication and vortexing, 8 µL of the SPLASH standard and 750 µL MTBE (4 °C) were added. The mixture was incubated for 1 h at 4 °C in a thermomixer at 650 rpm. Subsequently 188 µL of ultrapure water was added to induce phase separation. The samples were centrifuged at 10,000 g for 10 min at 4 °C, afterwards the upper organic layer was transferred to another Eppendorf tube and dried under continuous N_2 flow. The dried lipid extract was re-suspended in 100 µL of IPA/MeOH/$CHCl_3$ (4:2:1, v/v/v) for further MS analysis. The lower layer was used to precipitate proteins by adding 903 µL MeOH after removing the interphase and samples were stored at -80 °C for 3 h in order to perform protein precipitation. Protein pellets were collected after centrifugation at 19,000 g for 30 min at 4 °C and stored in 1 % SDS, 150 mM NaCl, 50 mM Tris (pH 7.8) solution at -80 °C. Protein amount was quantified by BCA (bicinchoninic acid assay, Thermo Fisher Scientific, Rockford, USA).

To extract lipid mediators, additional 20 µL of acetic acid (99.99%, 17.5M) was added together with 10 µL of mediator internal standard mixture (consisting of 0.5 mg per L of TXB2-d4 and PGE2-d4; 5 mg per L of PGD2-d4, 12-HETE-d8 and AA-d8; 0.2 mg per L of 15-HETE-d8 and 13-HODE-d4; 0.01 mg per L of DHA-d5; and 0.02 mg per L of EPA-d5) and 750 µL MTBE into the sample. After that, same incubation and centrifugation procedures as described above were applied. The dried lipid extract was re-suspended in 50 µL of MeOH for further MS analysis. Protein pellets were collected and protein amount was quantified as described above.

Plasma

Process Quality Control (PQC) samples were generated by pooling equal volumes of each plasma sample. Aliquots (10 µL) of the corresponding pooled PQC samples and blank samples, which did not contain any plasma, were also prepared and analyzed together with the experimental samples.

Plasma lipids were extracted using a single-phase, butanol/methanol-based method[2]. 15 µL from each plasma sample (including PQC) was transferred to a 2 mL Eppendorf tube. Subsequently, 150 µL of 1-butanol:methanol (1:1, v/v) containing 7.5 µL deuterated internal standard mix SPLASH® LIPIDOMIX® (Avanti Polar Lipids, Alabaster, USA), 44 nmol per L ceramide d18:1/16:0-D_{31} (Avanti) and 100 nmol per L lactosylceramide d18:1/16:0-D_3 (Matreya LCC, State College, PA, USA) was added. Tubes were vortexed for 30 sec and sonicated for 30 min in an ultrasound water bath (20 °C). After centrifugation (10 min, 14,000 *g*, 4 °C), the supernatant was transferred to polypropylene autosampler vials for subsequent LC-MS analysis.

Yeast

21 lipid standards (Avanti, Alabaster, AL, USA), PS 18:0-22:6, PG 18:0-22:6, PE 18:0-22:6, PA 18:0-22:6, PC 18:0-22:6, PA 16:0-22:6, PC 16:0-20:4, PE O 18:1p/20:4, PC O 18:1p/20:4, PC O 18:1p/22:6, PA 18:0-20:4, PC 18:0-20:4, PE 18:0-20:4, PG 18:0-20:4, PI 18:0-20:4, PS 18:0-20:4, ChE 22:0, ChE 17:0, SHexCer 18:1;2/12:0, SM 18:0;2/12:0, CerP 18:0;2/16:0 which are not naturally occurring in *S. cerevisiae*, were used to prepare a lipid standard mixture. Briefly, from the original stocks, standards were pipetted out into one vial, dried under continuous N_2 flow and dissolved in IPA/MeOH/$CHCl_3$ (4:2:1, v/v/v) in order to reach a final concentration of ~15 µM. Until further processing, the standards and the standard mixture were stored at -20 °C under nitrogen.

For lipid extraction, yeast cells were resuspended in 1ml 150 mM NH_4HCO_3 (pH 8) and lysed at 4°C with 500 µm acid-washed glass beads (Sigma) using a Mini-Beadbeater (Biospec) with 4 cycles of 1 min, as described by Ejsing *et al.*[3] The cell lysate was further diluted to 0.2 OD units per 200 µL. The cell lysate generated in this way were then extracted in a thermomixer with 990 µL $CHCl_3$/MeOH (17:1, v/v) for 2 h, at 650 rpm. Subsequently, the lipid-containing phase was collected and the remaining aqueous phase was reextracted with 990 µL $CHCl_3$/MeOH (2:1, v/v) for 2 h. The lower lipid-containing phase was collected, mixed with the first lipid extract and dried under continuous N_2 flow. Finally, the dried lipid extracts were dissolved in 40 µL of IPA/MeOH/$CHCl_3$ (4:2:1, v/v/v) and 5 lipid extracts were pooled together. The previously prepared lipid standard mixture was spiked into the total yeast lipid extract to a final concentration of ~3 µM.

LC-MS/MS

Targeted LC-MS/MS analysis of phospholipids and glycerolipids

For the reverse-phase liquid chromatography (LC), an UltiMate 3000-system from Thermo Fischer Scientific (Darmstadt, Germany) was employed. The chromatographic separation was performed according to Bird *et al.*[4] on an Ascentis Express C18 main column (150 mm × 2.1 mm, 2.7 µm, Supelco) fitted with a guard cartridge (5.0 mm × 2.1 mm, 2.7 µm, Supelco). The temperatures of the autosampler and the column oven were set at 10 °C and 45 °C, respectively. Solvent A was ACN/H_2O (3:2, v/v) and solvent B was IPA/ACN (9:1, v/v). Both solvents contained 0.1 % formic acid, and 10 mM ammonium formate. The separation was carried out at a flow rate of 0.26 mL per min with the following 30 min long gradient: 0.0-1.5 min (hold 32 % B), 1.5-4.0 min (32-45 % B), 4.0-5.0 min (45-52 % B), 5.0-8.0 min (52-58 % B), 8.0-11.0 min (58-66 % B), 11.0-14.0 min (66-70 % B), 14.0-18.0 min (70-75 % B), 18.0-21.0 min (75-97 % B), 21.0-25.0 min (97 % B), 25.0-30.0 min (32 % B). Samples were injected with a volume of 5 µL and analyzed in triplicates.

The LC was coupled to a Q-Exactive HF (QEx HF) mass spectrometer (Thermo Scientific, Bremen, Germany). The following HESI source settings were used: sheath gas 50 arbitrary units, aux gas 15 arbitrary units, spare gas 1 arbitrary units, S-lens RF level 60 arbitrary units, capillary temperature 285 °C, aux gas heater temperature 370 °C, spray voltage 4000 V (positive mode). In PRM acquisition, the method was set as following: 0-9 min, negative mode, resolution of 120,000, AGC target value of 1×10^5 , maximum injection time of 250 ms, isolation window of 0.4 m/z, and normalized collision energy (NCE)

of 28; 9-16.4 min, negative mode, resolution of 60,000, AGC value of 1 × 10^5, maximum injection time of 115 ms, and NCE of 35; 16.5-30.0 min, positive mode, mass resolution of 120,000, AGC value of 1 × 10^5 , maximum injection time of 250 ms, and NCE of 35.

Targeted analysis of mediators

The chromatographic setup described above was applied with the following changes: the temperatures of the autosampler and the column oven were set to 10 °C and 30 °C, respectively. Solvent A was ACN/H_2O (3:7, v/v) and 0.1 % formic acid while solvent B was IPA/ACN (1:1, v/v). The separation was carried out at a flow rate of 0.4 mL per min with the following 20 min long gradient: initial (0 % B), 0.0-1.0 min (hold 0 % B), 1.0-13.0 min (0-100 % B), 13.0-16.0 min (hold 100 % B), 16.0 min (0 % B), 16.0-20.0 min (hold 0 % B). Solvent A was also used as transfer solution for injection. Samples and standards were injected with a volume of 5 µL and analyzed in triplicates, respectively. The LC was coupled to a QTRAP 6500 (Applied Biosystems, Darmstadt, Germany) which was equipped with an electrospray ion source (Turbo V Ion Source). The following ESI source settings were used for negative mode: curtain gas 10 arbitrary units, temperature 525 °C, ion source gas I 30 arbitrary units, ion source gas II 55 arbitrary units, collision gas medium; ion spray voltage -4500 V, entrance potential -10 V, and exit potential 10 V.

To validate lipid mediator species identified from QTRAP, the QEx HF mass spectrometer (Thermo Scientific, Bremen, Germany) was used to perform high resolution MS full scan (HR-FS) and data independent acquisition (DIA) analyses. The following HESI source settings were used: sheath gas 50 arbitrary units, aux gas 14 arbitrary units, sweep gas 3 arbitrary units, S-lens RF level 60 arbitrary units, capillary temperature 270 °C, aux gas heater temperature 380 °C, spray voltage 3400 V (negative mode). HR-FS was acquired at mass resolution of 240,000 at m/z 200 with an AGC value of 1 × 10^6 and maximum injection time of 100 ms. The negative mode of HR-FS was acquired from 200 to 400 m/z. In DIA, an inclusion list (with m/z 210, 230, 250, 270, 290, 310, 330, 350, 370, 390) was used. The DIA parameters were set as following: resolution of 60,000, AGC value at 1 × 10^5, maximum injection time at 50 ms, loop count at 100, isolation width at 20 m/z and a NCE at 21. DIA method preparation and data analysis were performed in Skyline according to:

[https://skyline.ms/wiki/home/software/Skyline/page.view?name=tutorial_dia].

Targeted analysis of plasma samples

LipidCreator was used to create the transition list. 433 lipid species which contain the species previously reported by Bowden *et al.*[5] were selected for quantification. A liquid chromatography mass spectrometry method, previously published by Huynh *et al.*[6] was used with modifications. The LC gradient (flow rate 0.4 mL per min) was as following: 15% to 50% B in 2.5 min; 57% B at 2.6 min; 70% B at 9 min; 93% B at 9.1 min; 96% B at 11.0 min; 100%B at 11.9 min; 15% at 12 min until 15 min. An Agilent 6495 triple quadrupole MS (Agilent Technologies, Santa Clara, CA) was used with the following source parameters: gas temperature 150°C, gas flow 17 L per min, nebulizer 20 psi, sheat gas heater 250°C, sheat gas flow 10 L per min, capillary voltage 3500 V. Ion Funnel parameters: pos high pressure RF 180, pos low pressure RF 160. Selected-reaction monitoring (SRM) measurements in positive mode were conducted as reported by Saw *et al.*[7]. Each sample was measured in quadruplicate.

Peak integration of SRM traces was done using Skyline. Each lipid species was manually validated for the correct annotation[6, 8] and quantified when (i) the signal-to-noise ratio was greater than three, compared to lipid species signals in blank samples and (ii) the coefficient of variation of the species in the PQC samples was less than 20% [8]. The concentrations of the lipid species were calculated by relating the peak area of each lipid species to the peak area of the corresponding internal standard.

Targeted analysis of yeast samples

Lipid separation was carried out as described above on a LC system connected to a QEx HF mass spectrometer. The same HESI source settings were used and the samples were analyzed with a PRM method, in both positive and negative ion mode respectively. The MS settings were: mass resolution 60 000, AGC target 1×10^6 and maximum injection time of 50 ms for MS1 and, mass resolution 30 000, AGC target 1×10^5, maximum injection time of 100 ms and isolation window of 0.5 m/z for MS2. Target inclusion lists corresponding to the 21 (positive mode) and 19 (negative mode) lipid standards were obtained from LipidCreator. Samples were measured in triplicate for both polarities. Additionally, a decoy inclusion list was generated with LipidCreator. 21 random lipids naturally occurring in human platelets, different from the selected standards and not occurring in *S. cerevisiae* were queried in LipidCreator and analyzed using the described settings.

PRM data was analyzed in Skyline and all matching scans were included. The transition list files were imported as downloaded from LipidCreator. Each feature was manually validated in all replicates. A lipid species was defined as being identified if peaks were observed both in the MS1 and MS2 spectra at the same retention time and with a mass error lower than 5 ppm. As the total number of transitions per lipid species is variable, a uniquely identified lipid needed to have at least 75% matching transitions.

Direct infusion of lipid standards on QEx HF and Q-TOF

All 136 lipid standards were separately diluted to ~1 µM in IPA/MeOH/CHCl3 (4:2:1, v/v/v) with 7.5 mM ammonium acetate and then infused via robotic nanoflow ion source TriVersa NanoMate (Advion BioSciences, Ithaca NY, USA) into the QEx HF mass spectrometer using chips with spraying nozzles of 4.1 µm. The ion source was operated with the Chipsoft 8.3.1 software (Advion Biosciences). The following settings were used for positive or negative mode: ionization voltage +1.25 kV/ -1.25 kV, backpressure 0.95 psi, temperature of the ion transfer capillary 250 °C and S-lens level 60 arbitrary units. The PRM inclusion list was adapted for each lipid standard in terms of exact mass, ranging from 50 m/z to 650 m/z for MS1 and from 50 m/z to 350 m/z for MS2. The NCE was increased from 10-60 with a step increase of 1 and an average of 16 repetitions per NCE (minimum 1, median 18, maximum 21) and precursor using the following parameters: resolution of 30,000, AGC target of 2e5, maximum IT of 100 ms and isolation window of 0.5 m/z. The acquisition time was 2 min for each measurement.

Lipid standards were separately diluted to ~10 µM in MeOH with 0.1 % formic acid and then infused via syringe into Agilent Q-TOF 6545 (Agilent Technologies, Waldbronn, Germany). The following source parameters were used for Dual Agilent Jet Stream Electrospray Ionization in positive or negative mode:

gas temperature 200 °C, drying gas 5 L/min, nebulizer 25 psig, sheath gas temperature 200 °C, sheath gas flow 5 L per min, VCap 3500V, Nozzle voltage 2000 V, Fragmentor 200V, Skimmer 80V, Oct IRF Vpp 500V. For MS1, the method was set as following: m/z 100-1700 of mass range, 10 spectra per s of rate and 100 ms per spectrum of time. For MS/MS, the method was set as following: m/z 50-1700 of mass range, 10 spectra per s of rate and 100 ms per spectrum of time. Inclusion list was adapted for each lipid standard with collision energy from 10-100 V with a step increase of 1 and an average of 3 repetitions per NCE (minimum 1, median 3, maximum 7) and precursor. The acquisition time was 1 min for each measurement. An overview of the total number of samples per fragment and PPM mass error is available in Supplementary Notes 3 for 10-HDoHE. Supplementary Data 5 and 6 (A: QExactive-HF and B: Q-TOF) report summary information and diagnostic plots for all mediators.

Supplementary Note 1

LipidCreator design and architecture - Collision Energy Optimization Dialog

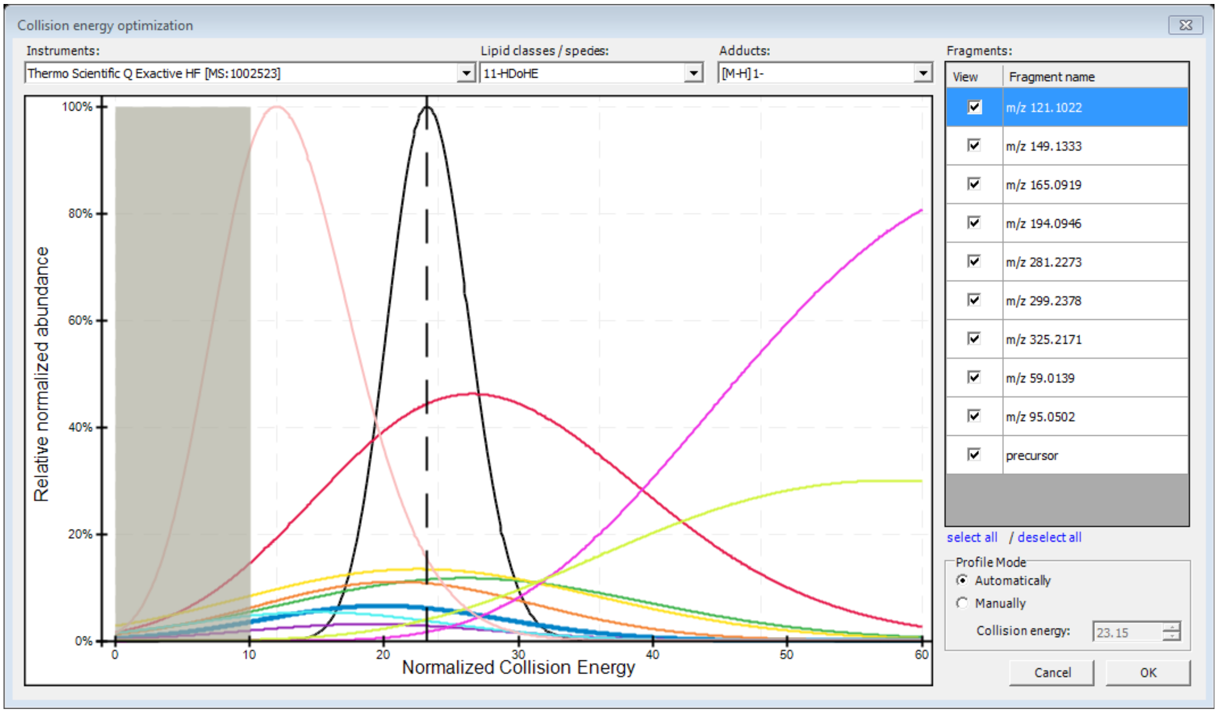

Supplementary Figure 1: Collision Energy Optimization dialog of LipidCreator enables fragment specific assay adaptation. The main window shows the collision energy (CE) plotted against the predicted relative normalized abundance for each fragment of 11-HDoHE in negative mode for the $[M\text{-}H]^{1-}$ precursor adduct on the Thermo Scientific Q Exactive HF platform. The right-hand side displays a sub-selection of the available fragments, controlling the location of the mode (dashed black line) of the product density distribution (solid black curve) that defines the automatically calculated CE. This ensures broad and simultaneous coverage of fragments. By manually including or excluding specific fragments, the user can focus on specific fragments or on all of them.

Validation of LipidCreator

Validation of lipid identification and quantification with calibration curves

In order to prove that true lipids are detected and analyzed we investigated the concentration response of PUFA-PL used in platelets. The rationale behind this was that only a true lipid will display a linear response. We used PUFA-PL; including PA, PC, PE, PG, PI and PS with the fatty acyl combination 18:0-20:4 which are endogenous lipid species. To create the calibration curve, the following concentrations were used and analyzed by LC/ESI SRM (see Section Targeted analysis of mediators). For all analyzed lipids a linear response was observed (Supplementary Figure 2), displaying an average correlation coefficient of 0.99 proving the correct output of LipidCreator.

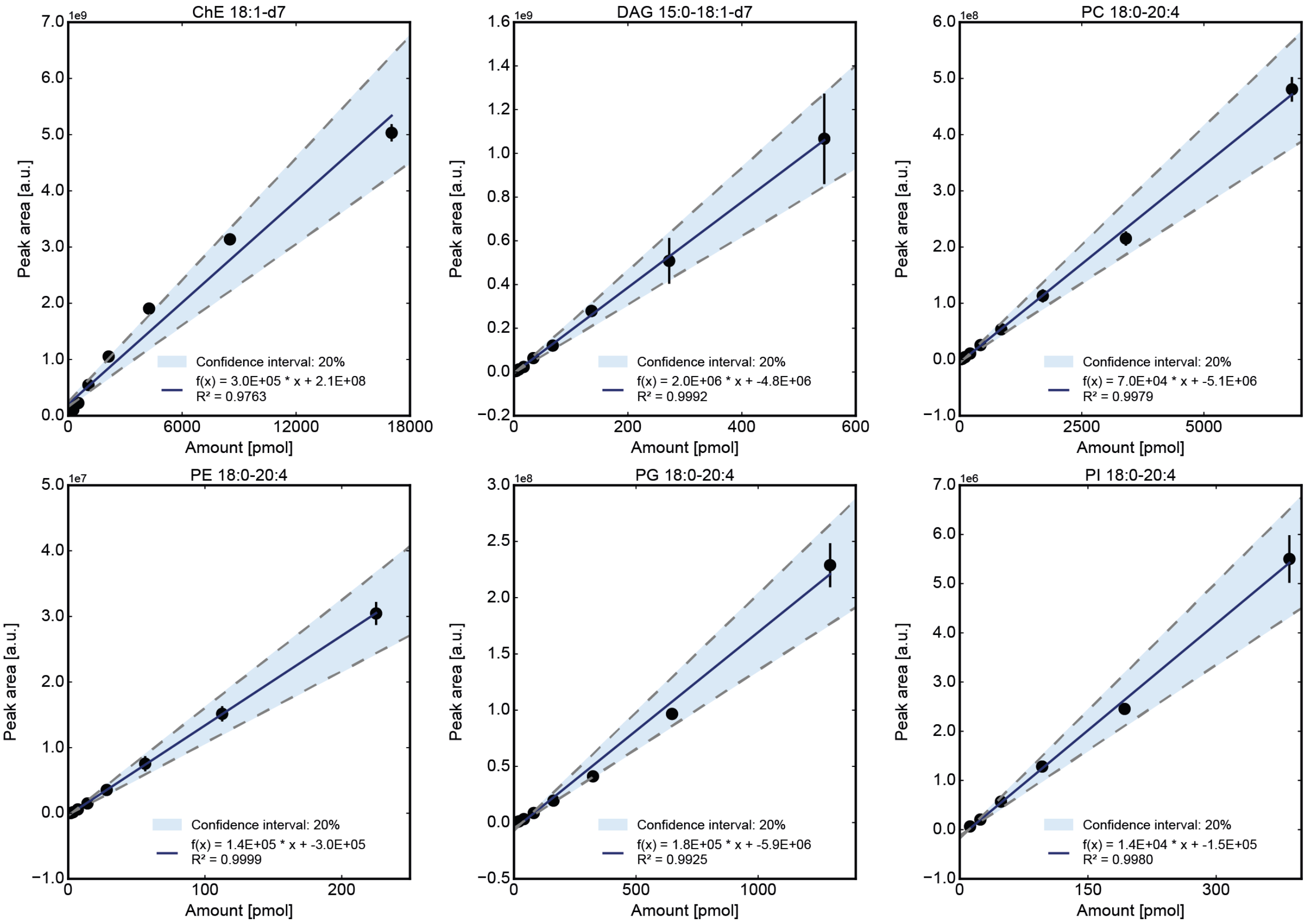

Supplementary Figure 2. Calibration curves of individual lipid species show a linear concentration response in human platelets. The calibration curves of glycerophospholipids (PC, PE, PG, and PI) 18:0-20:4 in samples were obtained by spiking the matrix with standard lipids with amounts ranging from 1.76 pmol to 225.17 pmol for PE 18:0-20:4 to amounts ranging from 66.52 pmol to 17030.49 pmol for ChE 18:1-d7, followed by a subsequent LC/ESI PRM analysis. All spiked concentrations were prepared and measured in triplicates. Source data are provided as a Source Data file. Dots represent the arithmetic mean concentration (n=3 independent experimental replicates). Error bars represent the standard deviation.

Applicability on a variety of diverse organisms

LipidCreator provides lipid classes that occur in almost every biological organism. To demonstrate the broad usage of LipidCreator, we collected several lipid distributions of different model organisms from the literature and calculated the percentage of the reported lipids that are supported by LipidCreator (Figure 4). Therefore, we used the lipid distributions from the following publications:

A) Human plasma by Begum *et al.*[9]

B) Human platelet by Peng *et al.*[10]

C) Mouse heart by Keat *et al.*[11], unsupported: 1 x GM1, 12 x Acylcarnitine, 1 x Ubiquinone

D) Mouse platelet by Peng *et al.*[10]

E) Mouse brain by Almeida *et al.*[12] and Ellis *et al.*[13]

F) Yeast lipidome by Ejsing *et al.*[3],

G) Zebrafish lipidome by Fraher *et al.*[14]

H) Drosophila lipidome by Guan *et al.*[15]

I) Arabidopsis lipidome by Higashi *et al.*[16]

J) Top 100 most abundant lipids from Escherichia coli by Herzog *et al.*[17], Jeucken *et al.*[18], and Matyash *et al.*[1], unsupported: 3 x DLCL, 15 x aPG, 6 x aPE

Please see Supplementary Data 1 for details on unsupported classes.

Verification of lipid mediators using the *in silico* spectral library from LipidCreator

The *in silico* spectral library was generated from LipidCreator, using same CE as in the experimental setting. The fragments for each lipid mediator were extracted and reviewed in Skyline. The predicted relative intensities of fragments in spectral library increase the confidence of lipid identification. The results are shown as dot-product (Dotp) values (Supplementary Figures 3-8).

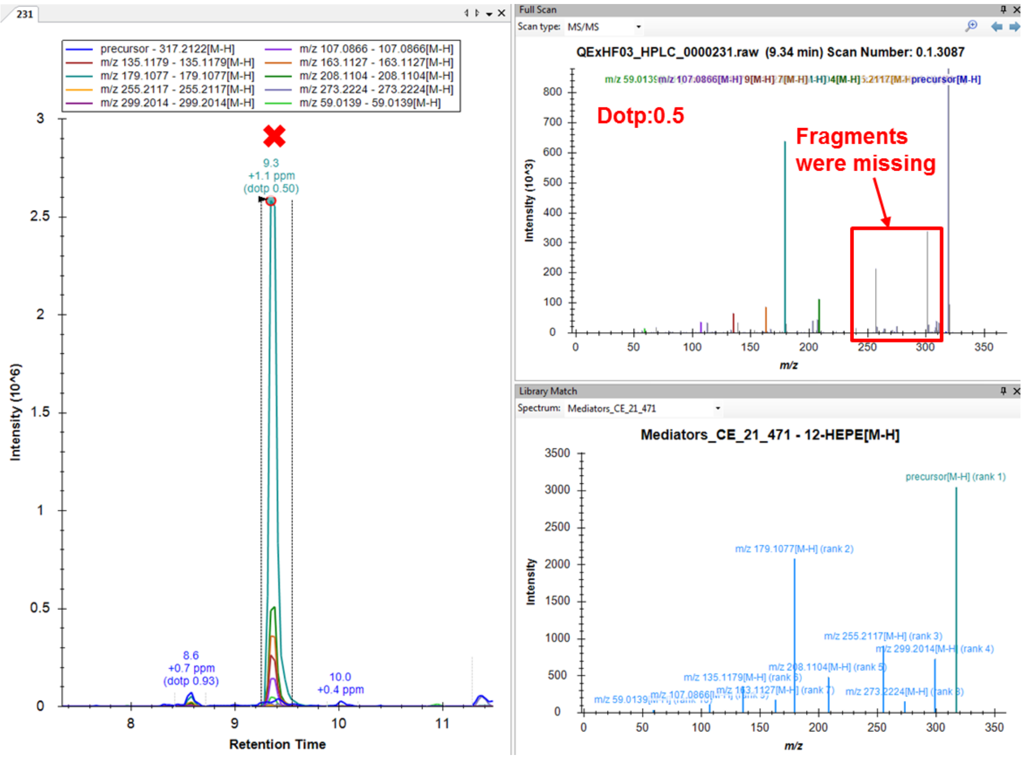

Supplementary Figure 3. Screenshot of Skyline interface of library matching for the wrong identification of 12-HEPE.

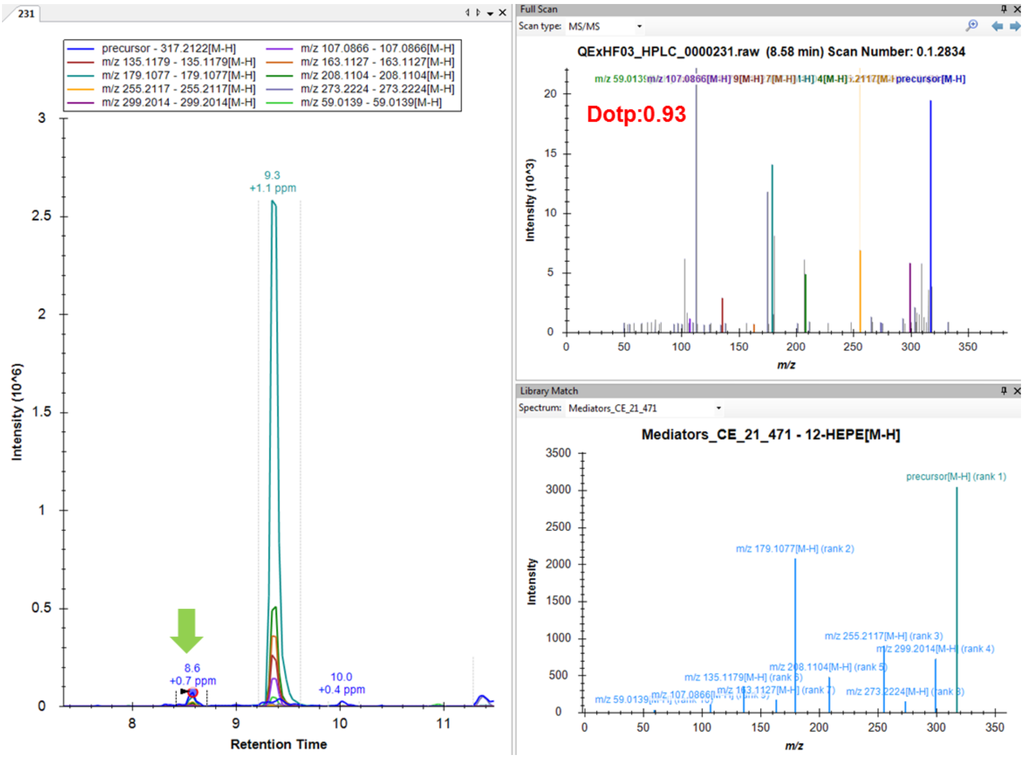

Supplementary Figure 4. Screenshot of Skyline interface of library matching for the correct identification of 12-HEPE.

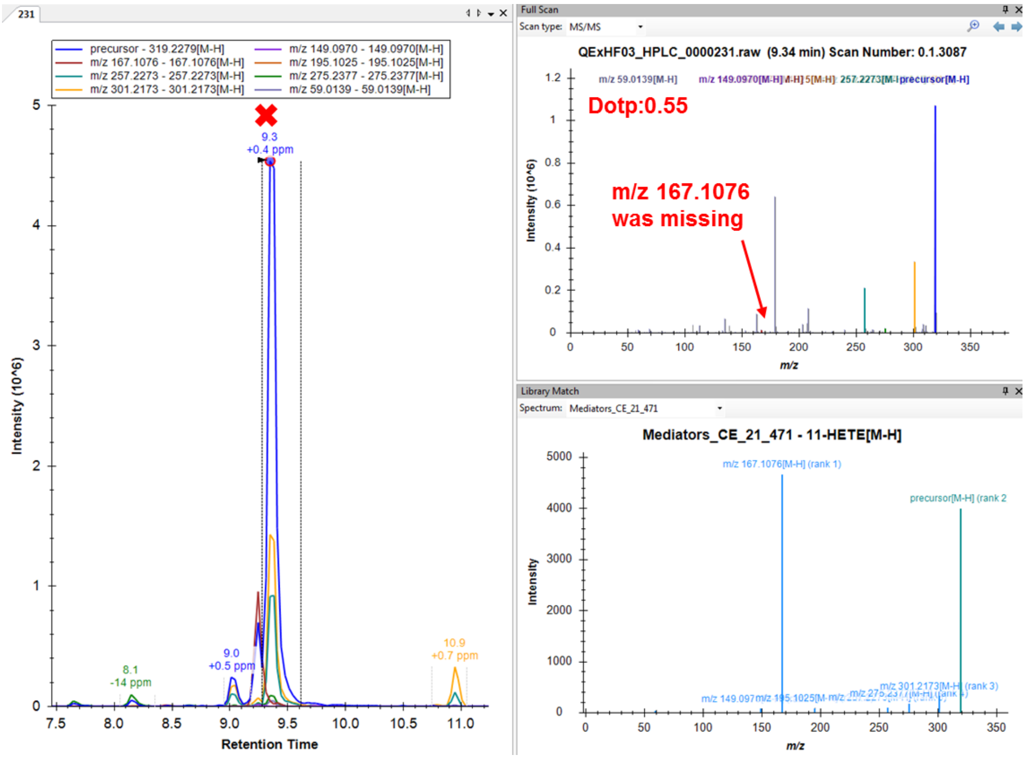

Supplementary Figure 5. Screenshot of Skyline interface of library matching for the wrong identification of 11-HETE.

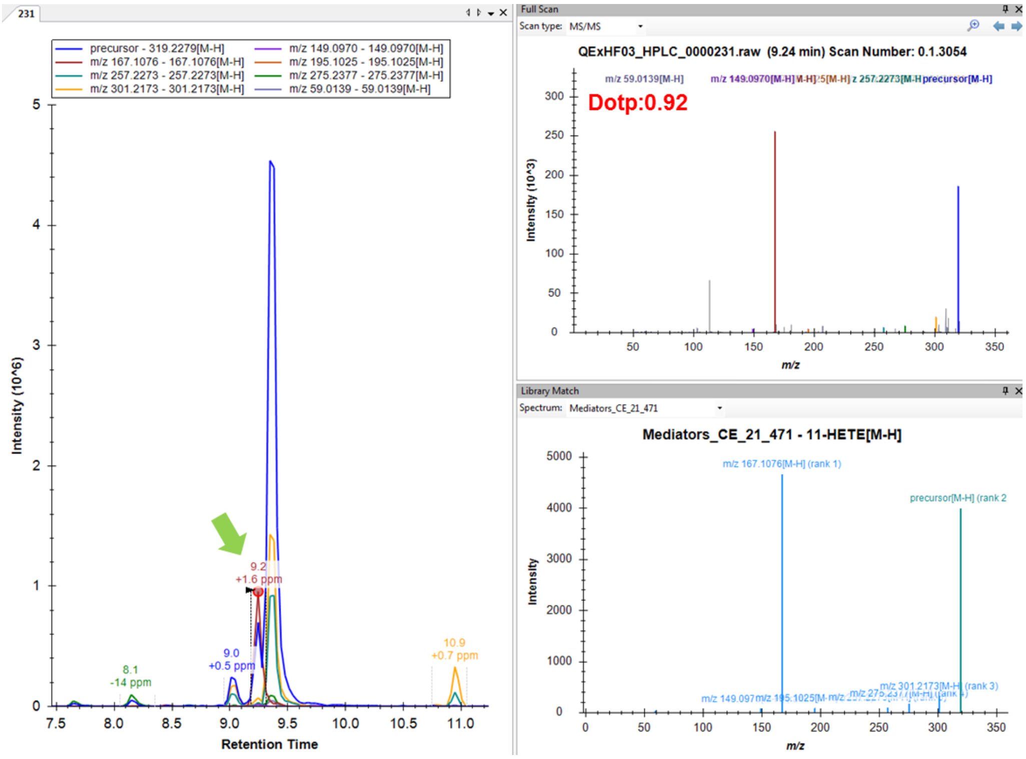

Supplementary Figure 6. Screenshot of Skyline interface of library matching for the correct identification of 11-HETE.

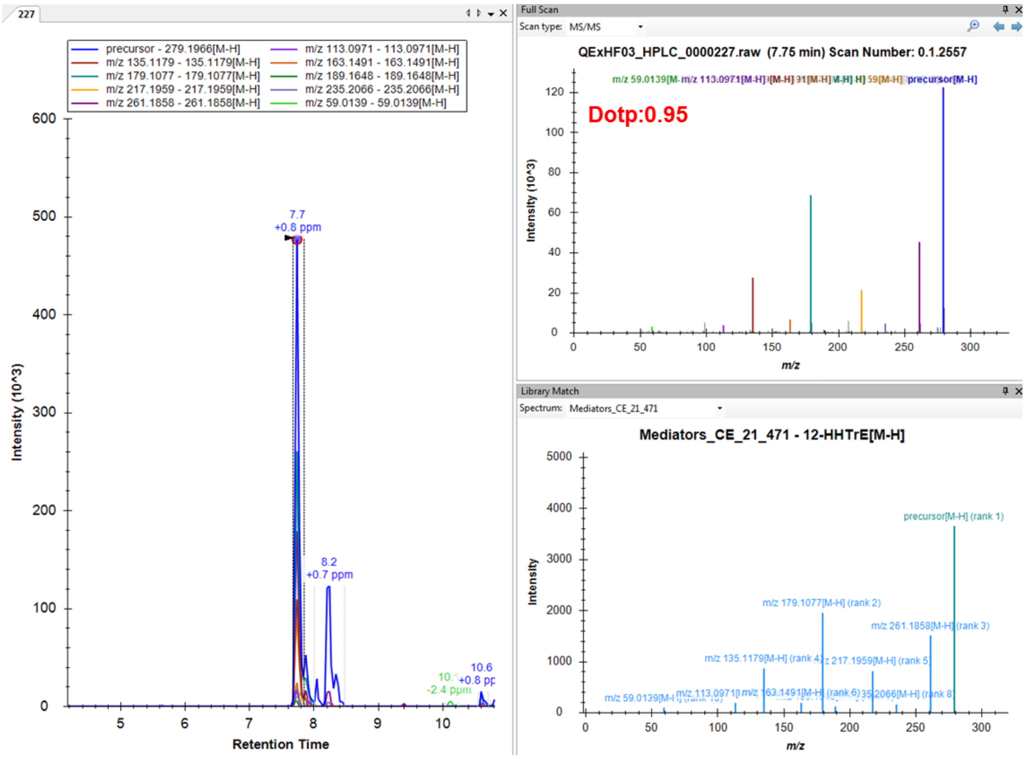

Supplementary Figure 7. Screenshot of Skyline interface of library matching for the identification of 12-HHTrE.

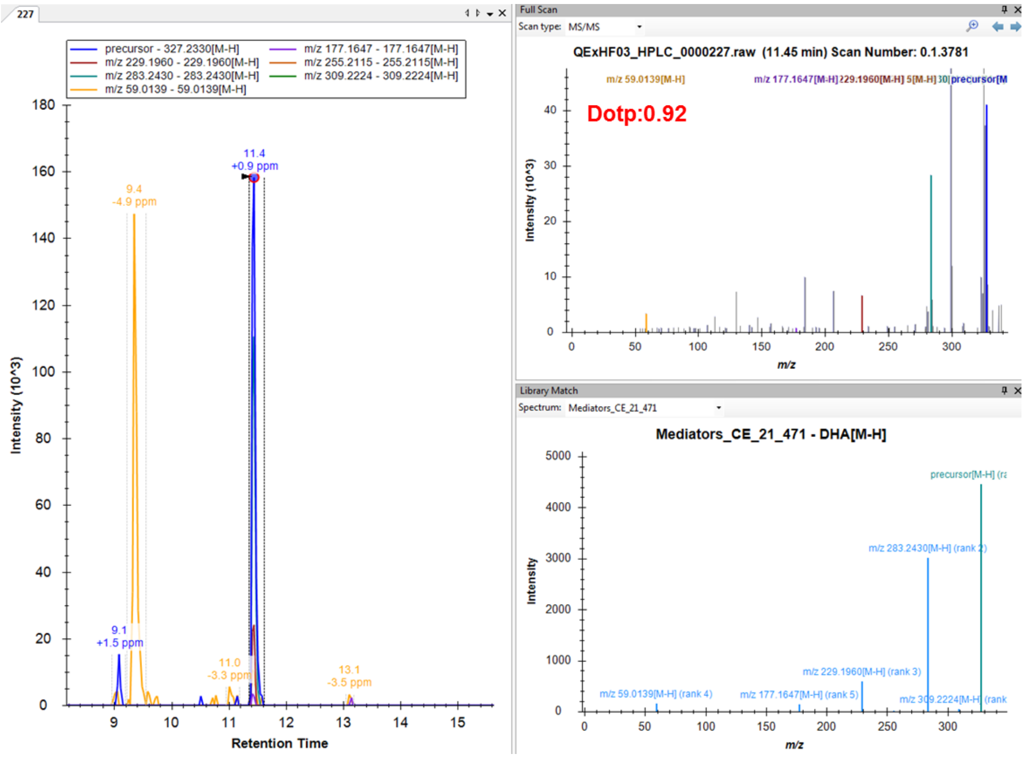

Supplementary Figure 8. Screenshot of Skyline interface of library matching for the identification of DHA.

Human plasma comparison to NIST SRM 1950 and Bowden ring trial

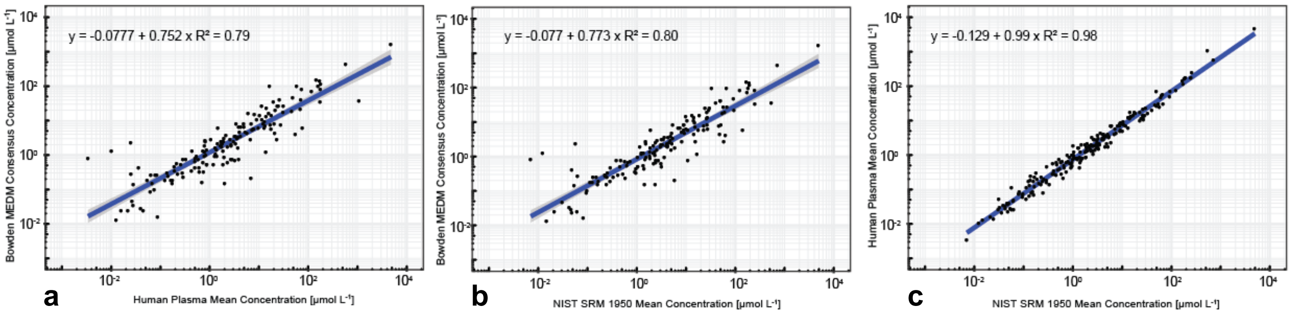

Supplementary Figure 9. a) The Log-Log scatterplot between the Human plasma samples and the Bowden ring trial MEDM consensus values shows a linear correspondence of R^2=0.79. b) The Log-Log scatterplot between NIST SRM 1950 standard and the Bowden ring trial MEDM consensus shows a linear correspondence of R^2=0.8. c) The Log-Log scatterplot between NIST SRM 1950 standard and the Human plasma samples shows high linear correspondence with R^2=0.98. Some differences in quantities are expected due to the different background of the NIST reference material and the plasma donors within this study. Confidence interval bounds were calculated at a level of 0.95.

Data analysis and repository information

Skyline (64-bit, 4.2.0.18305 and 19.1) were used to visualize results, integrate signals, and quantify all lipids that were detected by MS.

LipidCreator (version 1.1.0.x) can be downloaded from: [https://lifs.isas.de/lipidcreator].

The source code for LipidCreator is available at: [https://github.com/lifs-tools/LipidCreator].

The source code for flipR (version 1.0.6) is available at: [https://github.com/lifs-tools/flipr].

A representative subset of averaged CE spectra of lipid mediator standards is available from MassBank: [https://massbank.eu/MassBank/Result.jsp?type=rcdidx&idxtype=site&srchkey=ISAS_Dortmund]. The spectra were generated as averaged mass spectra from CE-series measurements on a Thermo QExactive HF platform and an Agilent Q-TOF platform of lipid mediators. Representative spectra were extracted and averaged at CEs 20,30,40 NCE for the QEx platform and at CEs 10,20,30 eV for the Q-TOF platform, both using a 5ppm selection window for the signals.

The representative measurements of human platelet activation and DIA validation (including a custom spectral library for NCE=21) were stored in a Skyline file and uploaded to Panorama: [https://panoramaweb.org/lipidcreator.url].

All other project files were uploaded to the MetaboLights repository. The corresponding accession numbers are provided in the main manuscript.

Supplementary Note 2

Manual for LipidCreator

This manual provides general information on the usage of LipidCreator. It explains all functionalities covered by LipidCreator's wizard, the four interactive tutorials and provides additional information for more advanced use-cases. To start a tutorial, the user needs to click on one of the four tutorial buttons in the "Home" tab as illustrated in Supplementary Figure 11 and 17. The following tutorials are currently provided: i) setting up a PRM or ii) SRM analysis, iii) creating heavy isotope labeled lipids and iv) adding optimized collision energies for mediator analysis. These tutorials guide the user through the user interface of LipidCreator and explain step by step all important information relevant for the understanding of the four workflows.

We provide the most current LipidCreator software releases, additional workshop training material and support at

[https://lifs.isas.de/lipidcreator]

An Overview of the LipidCreator Workflow

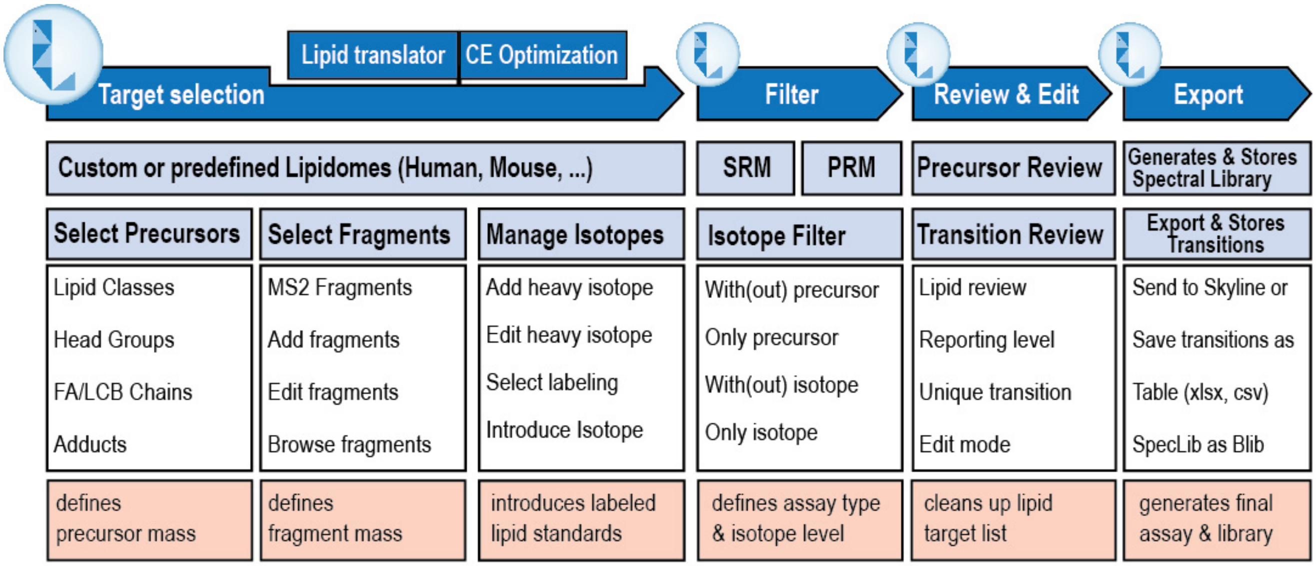

Supplementary Figure 10. LipidCreator Workflow.

The LipidCreator workflow consists of four main steps to generate SRM or PRM transition lists that can either be transferred to Skyline or exported for use in other software for targeted mass spectrometry method development, refinement and analysis.
These steps are:

1. Target selection – define, which lipid classes and species, which MS2 fragments and which labels, if any, should be included in the transition lists.
2. Filter – select, whether precursor and / or fragments should be included in the transition list and whether unlabeled, labeled or both combinations should be included in the transition list, where applicable.

3. Review & Edit – select, edit, or refine the lipid transitions, define the reporting level, e.g. lipid species or subspecies level, check and warn about potentially interfering transitions (with the same m/z).
4. Export – transfer the reviewed transitions directly to Skyline, or store them in tabular format. If CE Optimization has been enabled, optionally transfer an *in-silico* spectral library directly to Skyline, or store it in Bibliospec (BLIB) format.

Using the LipidCreator Wizard

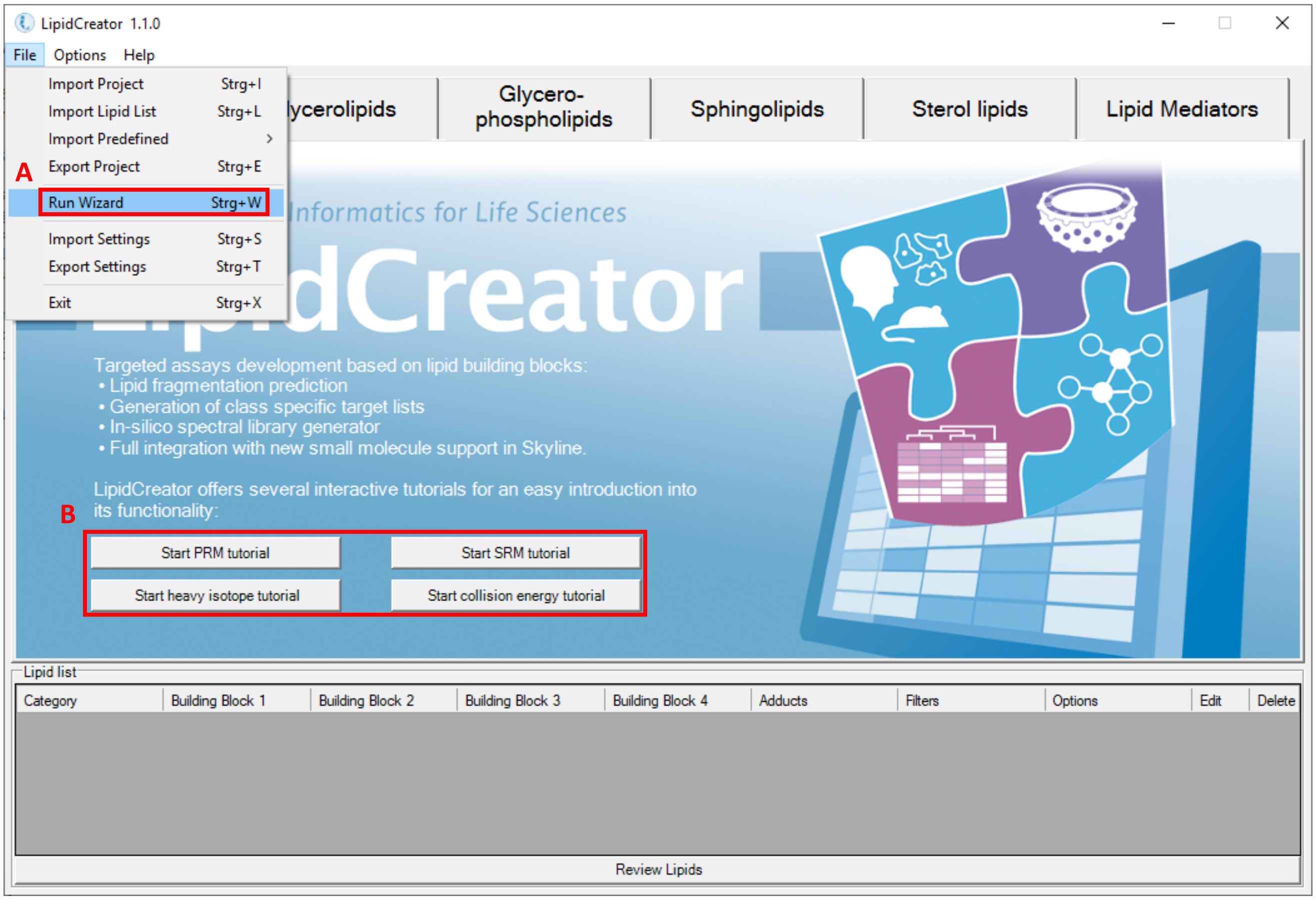

Supplementary Figure 11. Home tab of LipidCreator, File menu.

A: The LipidCreator Wizard can be started via the **File → Run Wizard** menu entry. Please note that the wizard panels are context-dependent. Thus, not all panels will be available for every lipid species. Also, the wizard does not provide direct filters for isotope labeling. However, after the transitions have been added to the 'Lipid list', they are editable individually. See Filters for the transition list for more details.

B: Interactive tutorials that guide the user step-by-step through LipidCreator.

To learn how to exploit LipidCreator's full potential, especially for users with a more advanced background in Lipidomics, Generating lipid molecule transitions provides a step-by-step description of the LipidCreator user interface and all options.

The next pages describe the individual wizard panels in more detail.

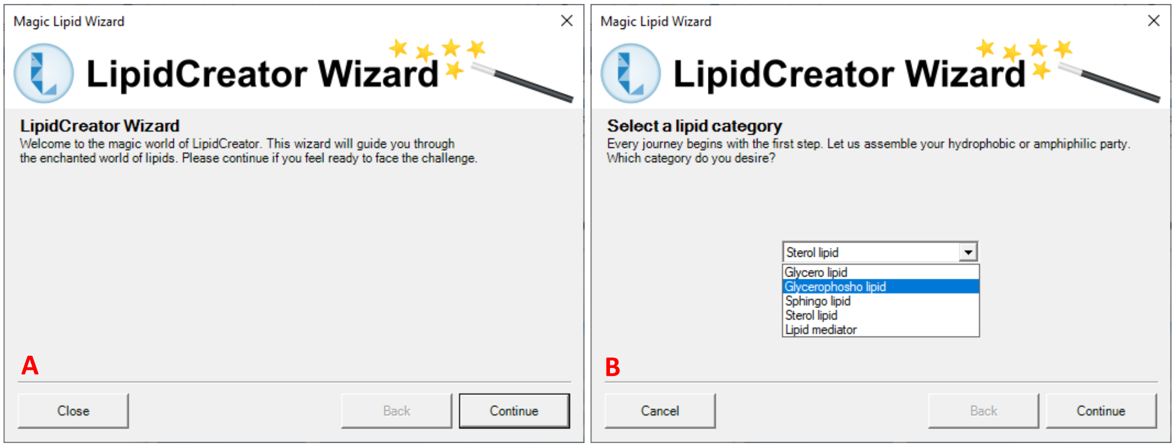

Supplementary Figure 12. LipidCreator Wizard welcome screen and Lipid Category selection panel.

A: By clicking on 'Continue', the Wizard will transition to the 'Lipid category' selection panel. The user can decide to close the Wizard. In that case, no wizard data is saved! Click 'Continue' to go to the next panel.

B: After selection of an initial lipid category, the user can proceed to the next step ''. Please note that the Wizard is intentionally simple. If you want to have full flexibility, please use the regular user interface (See <u>Generating lipid molecule transitions</u>). Click 'Continue' to proceed.

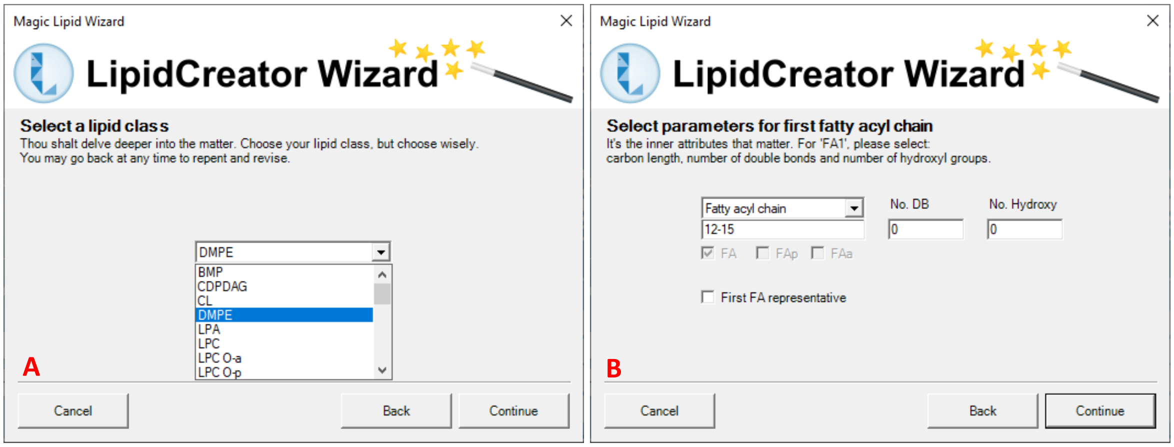

Supplementary Figure 13. LipidCreator Wizard lipid class selection and first fatty acyl chain definition panels.

A: The lipid class selection shows the available lipid classes for the previously selected lipid category. Choose one and click 'Continue' to proceed to the FA chain definition panels.

B: The FA definition panel (depending on the lipid class, there may be multiple FAs to define) allows to set the number of carbon atoms (also as ranges), the number of double bonds and hydroxylations. Also, the bond can be set to be of type plasmenyl (FAp) or plasmanyl (FAa), depending on the lipid class. Click 'Continue' for the next panel.

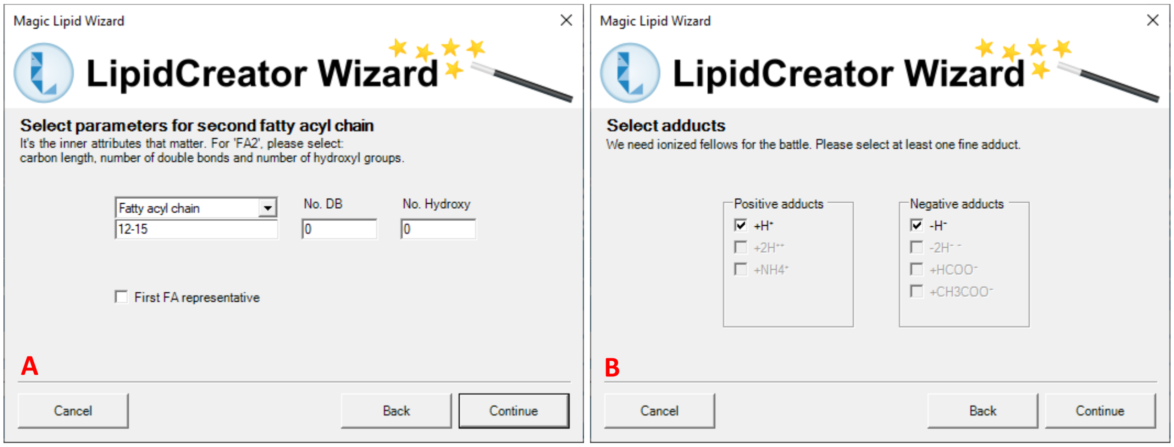

Supplementary Figure 14. Second fatty acyl chain definition and adduct selection panels.

A: Some lipids may have more than one FA to define, or a long-chain-base (LCB). Thus, more than one FA panel may be part of your Wizard sequence. The definitions are basically the same, but may differ slightly based on the lipid species context. Click 'Continue' to go to the adduct selection panel.

B: The adduct selection panel shows potential adducts for the specific lipid species, in positive or negative mode. Any or all of the chemically feasible adducts can be selected. At least one adduct must be selected. Click on 'Continue' to proceed to precursor and transition filtering.

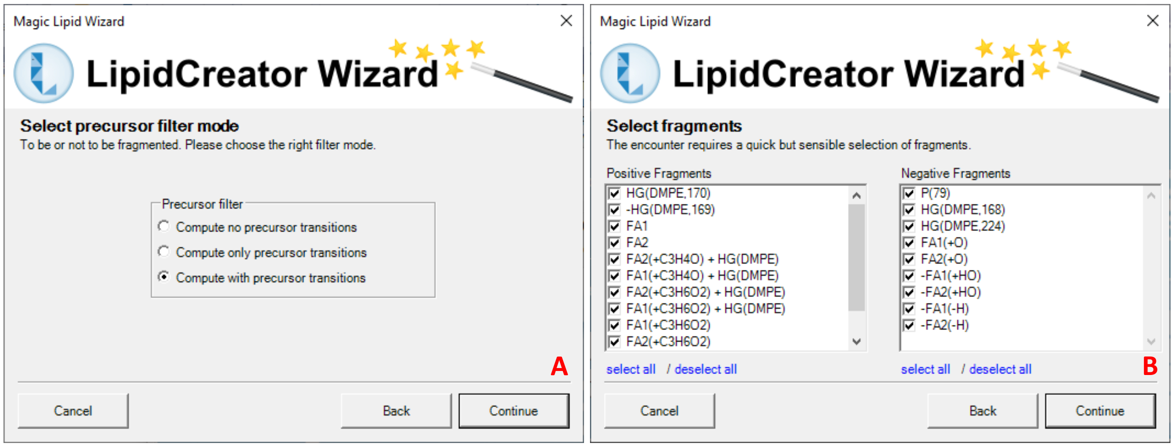

Supplementary Figure 15. Precursor filter selection and MS2 fragment selection panels.

A: This panel allows to select either only MS2 fragment transitions, only the precursor transitions, or both. Click 'Continue' to select the MS2 fragments to include.

B: Here, the MS2 fragments (positive and negative mode, where applicable) defined in the LipidCreator knowledgebase for the currently configured lipid species are shown. Select all, or deselect undesirable ones and click 'Continue' to proceed to the final steps.

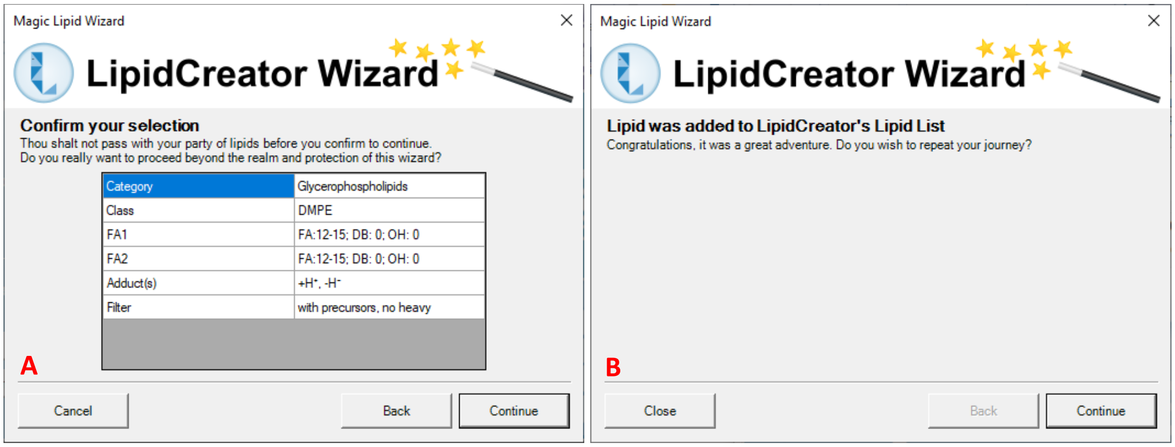

Supplementary Figure 16. Selection confirmation and continuation panels.

A: After clicking on 'Continue', the currently configured lipid transitions are transferred to LipidCreator's 'Lipid list' (See Generating lipid molecule transitions). The Wizard can be canceled at any time. In that case, no wizard data is saved!

B: After successful addition of the transitions to the 'Lipid list', the user can decide to either close the Wizard, or to continue from the beginning. The Wizard will then start over from the beginning. To review the transitions in the 'Lipid list' and to save or transfer them, see Overview of the LipidCreator User Interface and Reviewing the lipid transition list.

Overview of the LipidCreator User Interface

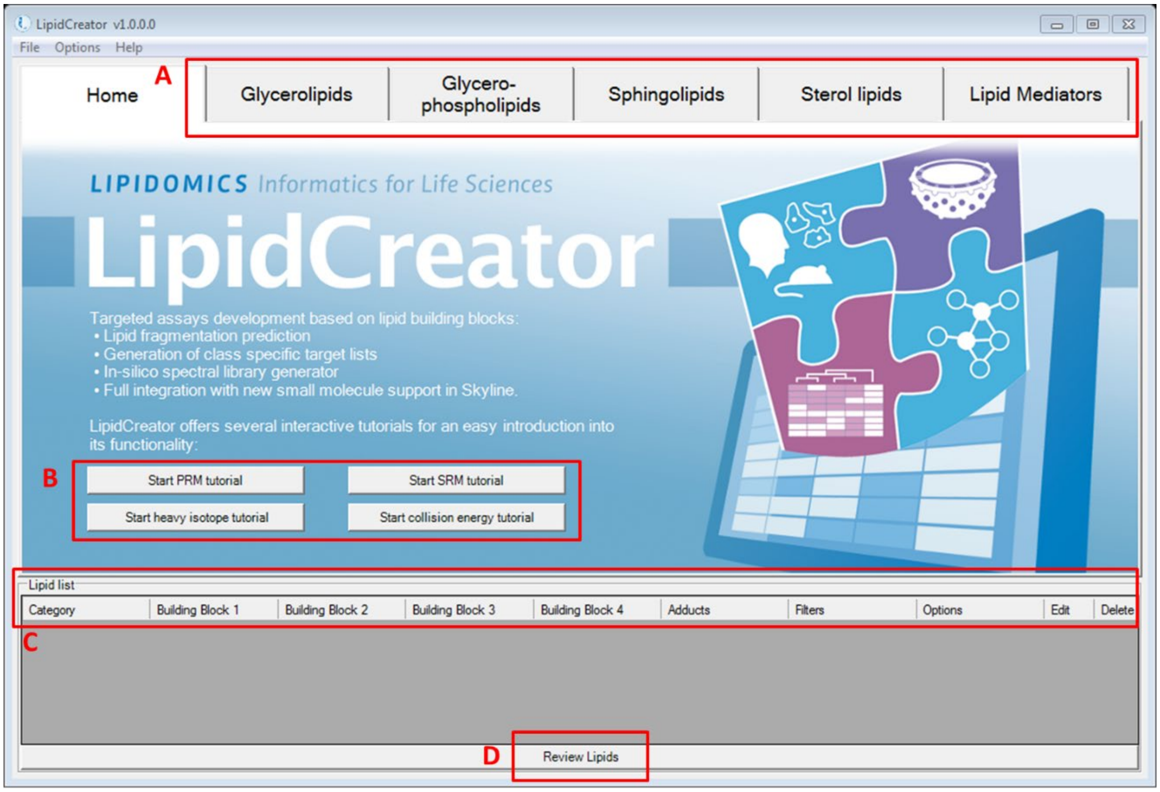

Supplementary Figure 17. Home tab of LipidCreator.

The main user interface after starting LipidCreator shows the home tab, four buttons to start workflow-specific, interactive tutorials, and the 'Lipid list' that collects user-defined lipid specifications for the later transition list generation.

A: Tab pages for five lipid categories. Each tab provides an individual interface for a specific lipid category.

B: Interactive tutorials that guide the user step-by-step through LipidCreator.

C: 'Lipid list' serves as a "shopping basket" to collect lipid assemblies.

D: Launching the computation of a transition list according to the 'Lipid list' selection.

The next pages give an overview of the individual tabs, their functionalities and the 'Lipid list' that collects the used-defined lipid specifications from the different tabs.

Generating lipid molecule transitions

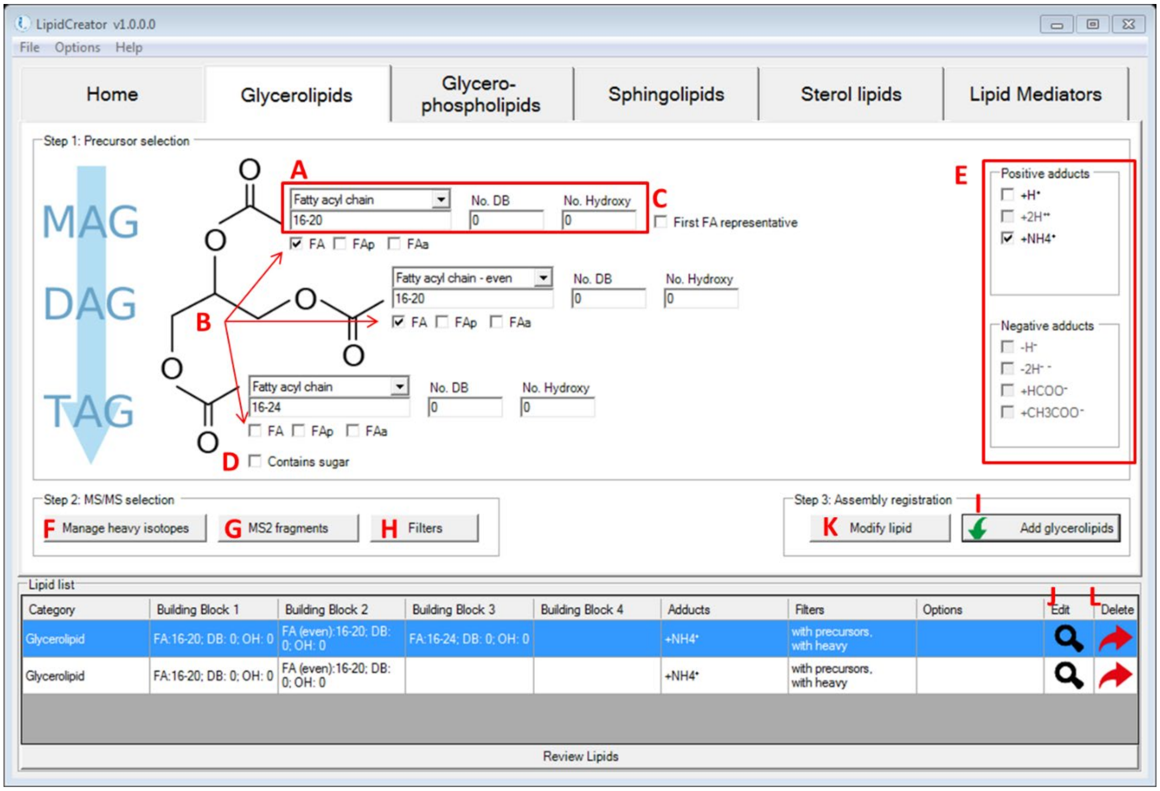

Supplementary Figure 18. Interface for the definition of transitions for glycerolipids.

A: These fields let the user define one fatty acyl chain. From the drop-down list, the calculation for fatty acyl (FA) length can be chosen for even, odd or all potentially possible FA lengths. Input of either FA length, number of double bonds (DB), or number of hydroxy groups on this FA may be a range of numbers or individual values, e.g. '8, 9, 16-20, 23'. In LipidCreator, the allowed range for one FA length is 2-30, No. DB is 0-6, Hydroxy No. is 0-10 (except for sphingolipids). For sphingolipids, Hydroxy No. for long chain base (LCB) is 2 and 3, for FA, it is 0-3.

B: The check boxes for (de)selecting different types of FAs. For glycerolipids, the number of checked FAs defines the lipid class. TAG has three FAs; DAG has two FAs, whereas MAG has only one FA. FA here refers to an ester-linked fatty acid. FAp and FAa are ether-linked fatty acids. FAp has an ether bond to an alkenyl group, and FAa has an ether bond to an alkyl group, respectively. The minimum number of DBs for FAp is 1.

C: The "First FA representative" checkbox is to quickly apply FA information from A) to all other FAs in current interface.

D: This checkbox is to replace one FA with a glucose head group.

E: All supported adducts in LipidCreator. For positive mode, [M+H]1+, [M+2H]2+ and [M+NH4]1+ are valid. For negative mode, [M-H]1-, [M-2H]2-, [M+HCOO]1- and [M+CH3COO]1- are valid. In

LipidCreator, recommendation on adduct selection can be reviewed, when hovering the mouse cursor over different head groups (HG).

F: To manage heavy isotopes for glycerolipids, please go to Managing heavy isotopes for details.

G: To select MS^2 fragments for glycerolipids, please go to Selection of MS2 fragments for details.

H: To apply filters for glycerolipids, please go to Filters for the transition list for details.

I: Adds the complete lipid assembly into the 'Lipid list" basket.

J-K: To modify a lipid assembly, double click on the icon to retrieve information according to Step 1 window. After making changes (including HG selection, FA profile, adducts selection, management of heavy isotopes, MS^2 fragments selection and filters selection), click on 'Modify lipid' from Step 2 window to update the assembly.

L: To delete a lipid assembly from the 'Lipid list', double click on the Icon.

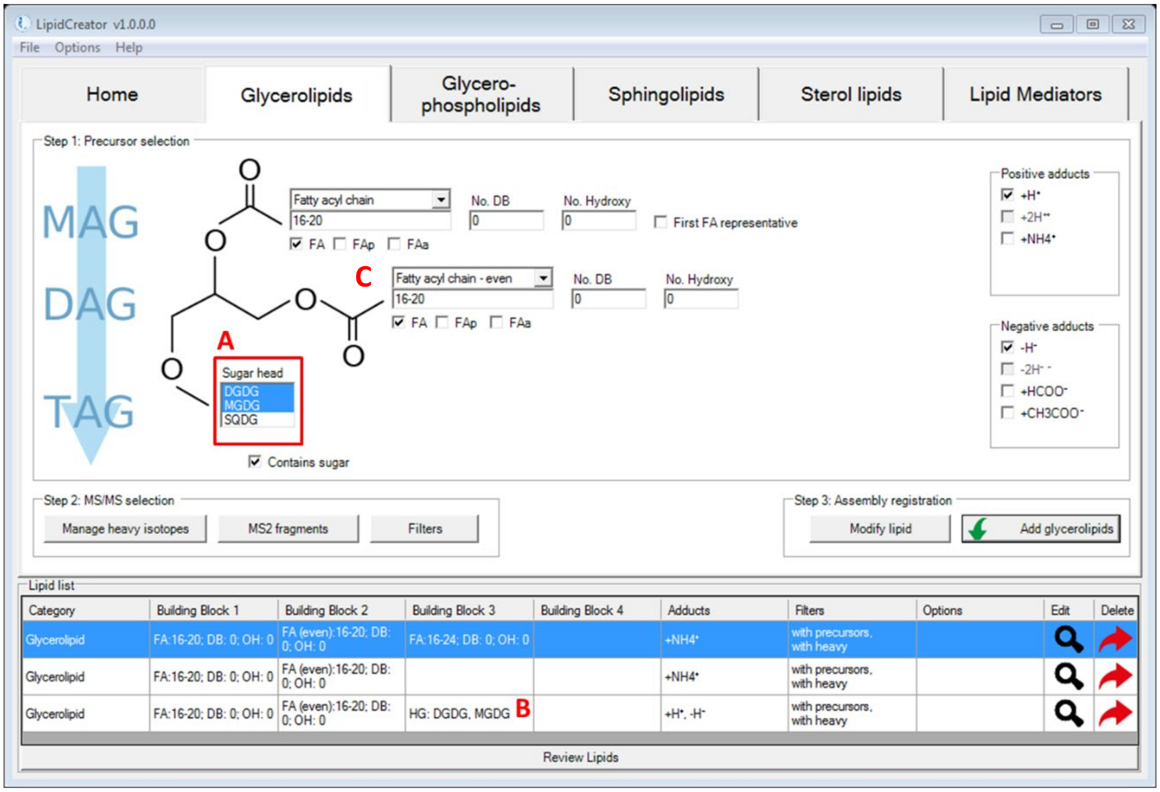

Supplementary Figure 19. Interface for the definition of transitions for glycerolipids with glucose head group.

A: Multiple selection is possible when choosing head groups in LipidCreator.

B: The selected head groups are displayed in 'Lipid list'.

C: Ester- or ether-linked fatty acyls (fatty acid, plasmenyl or plasmanyl) can be defined and parameterized.

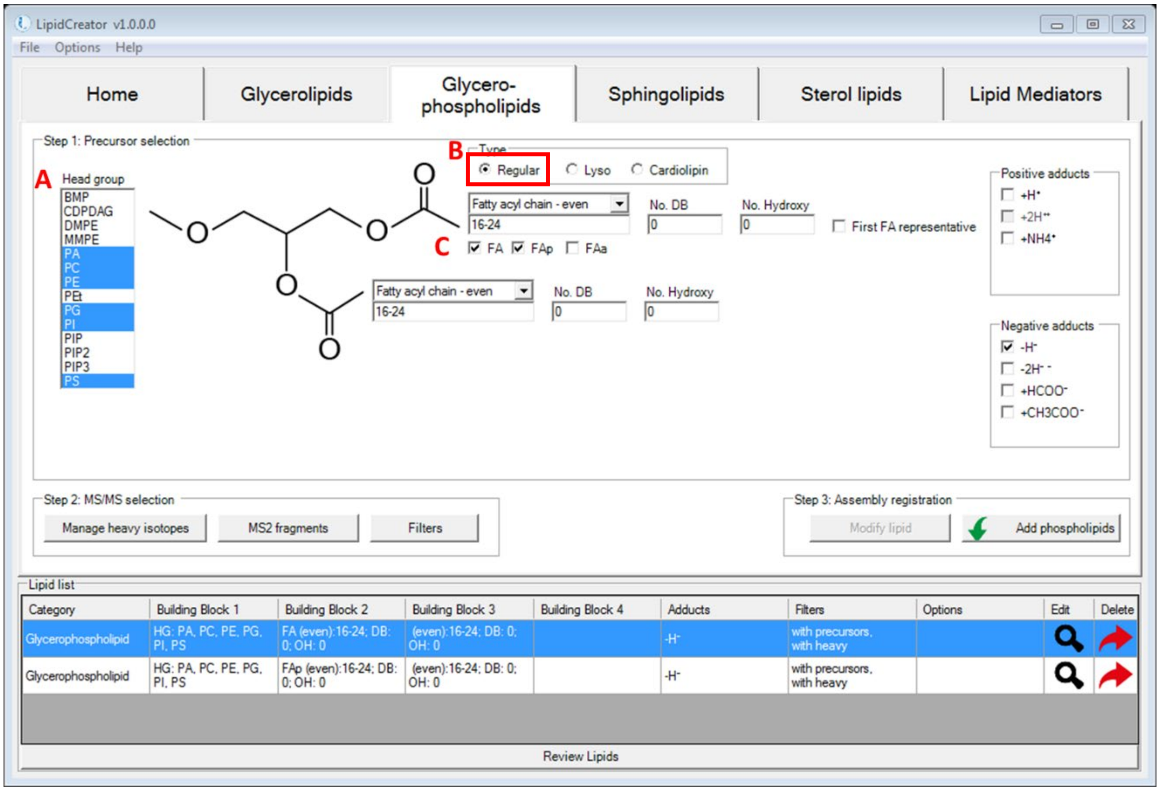

Supplementary Figure 20. Interface for the definition of transitions for glycerophospholipids.

A: Multiple selection of head groups is possible when choosing them from the head group menu.

B: Radio buttons switch between different types of glycerophospholipids.

C: Ester- or ether-linked fatty acyls (fatty acid, plasmenyl or plasmanyl) can be defined and parameterized.

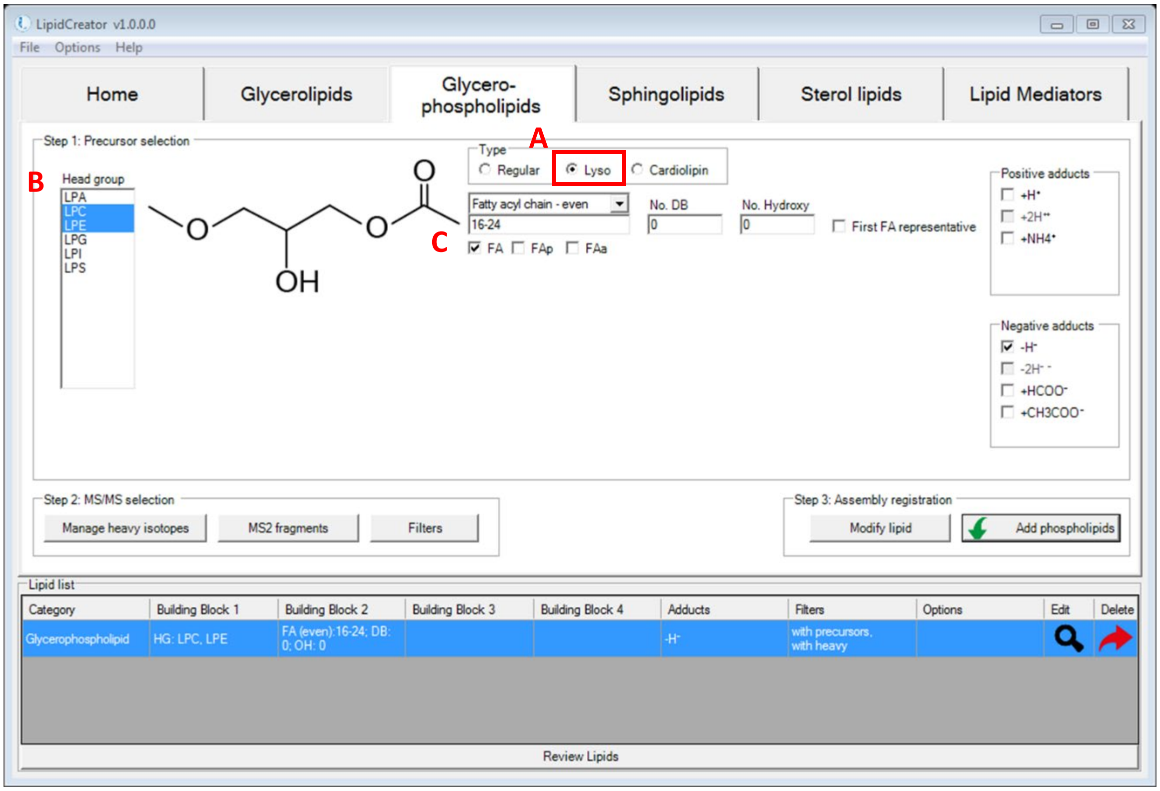

Supplementary Figure 21. Interface for the definition of transitions for lyso-glycerophospholipids.

A: Selecting the ‘Lyso’ radio button switches the Glycerophospholipids tab into ‘lyso’ mode.

B: Here, only ‘Lyso’ headgroups for PA, PC, PE, PG, PI and PS are displayed.

C: Ester- or ether-linked fatty acyls (fatty acid, plasmenyl or plasmanyl) can be defined and parameterized.

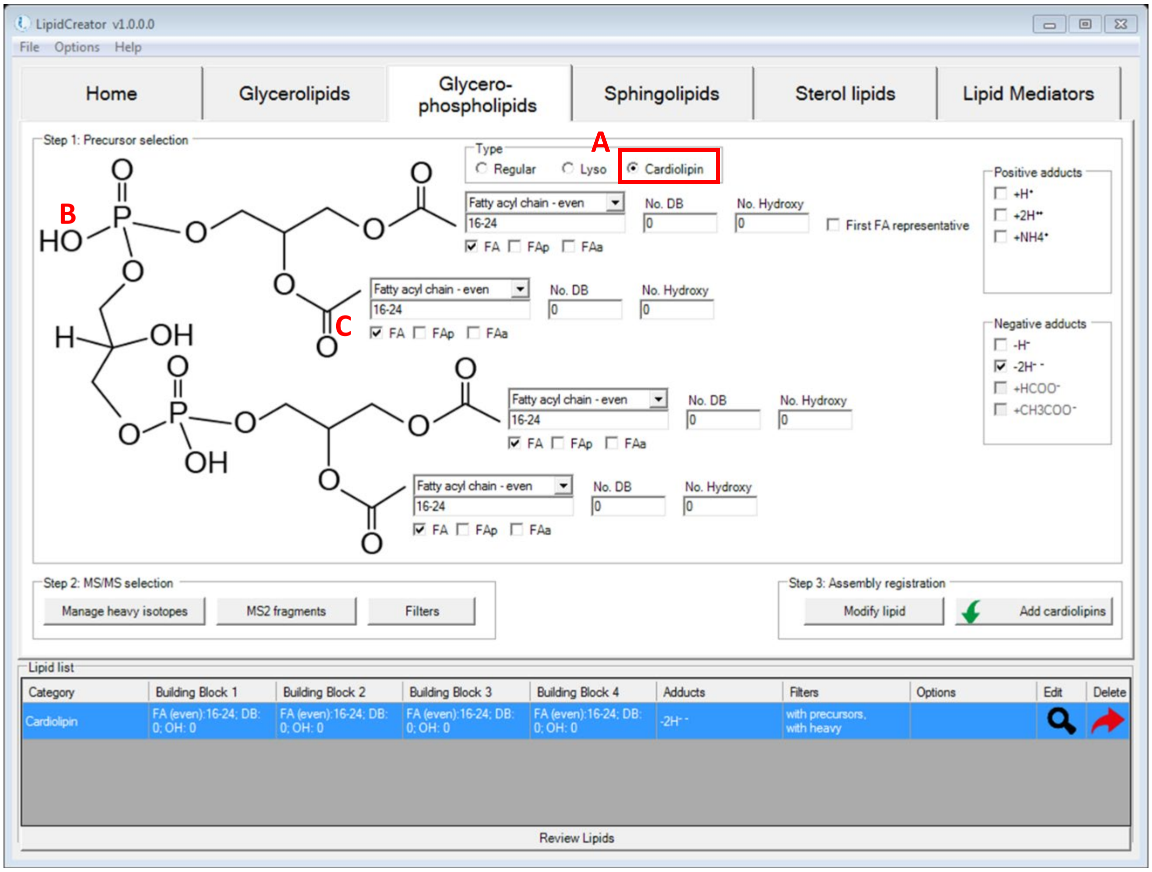

Supplementary Figure 22. Interface for the definition of transitions for cardiolipins.

A: Selecting the 'Cardiolipin' radio button switches the Glycerophospholipids tab into 'Cardiolipin' mode.

B: Here, only the four fatty acyl chains can be defined. The headgroup is fixed and non-modfiable.

C: Ester- or ether-linked fatty acyls (fatty acid, plasmenyl or plasmanyl) can be defined and parameterized.

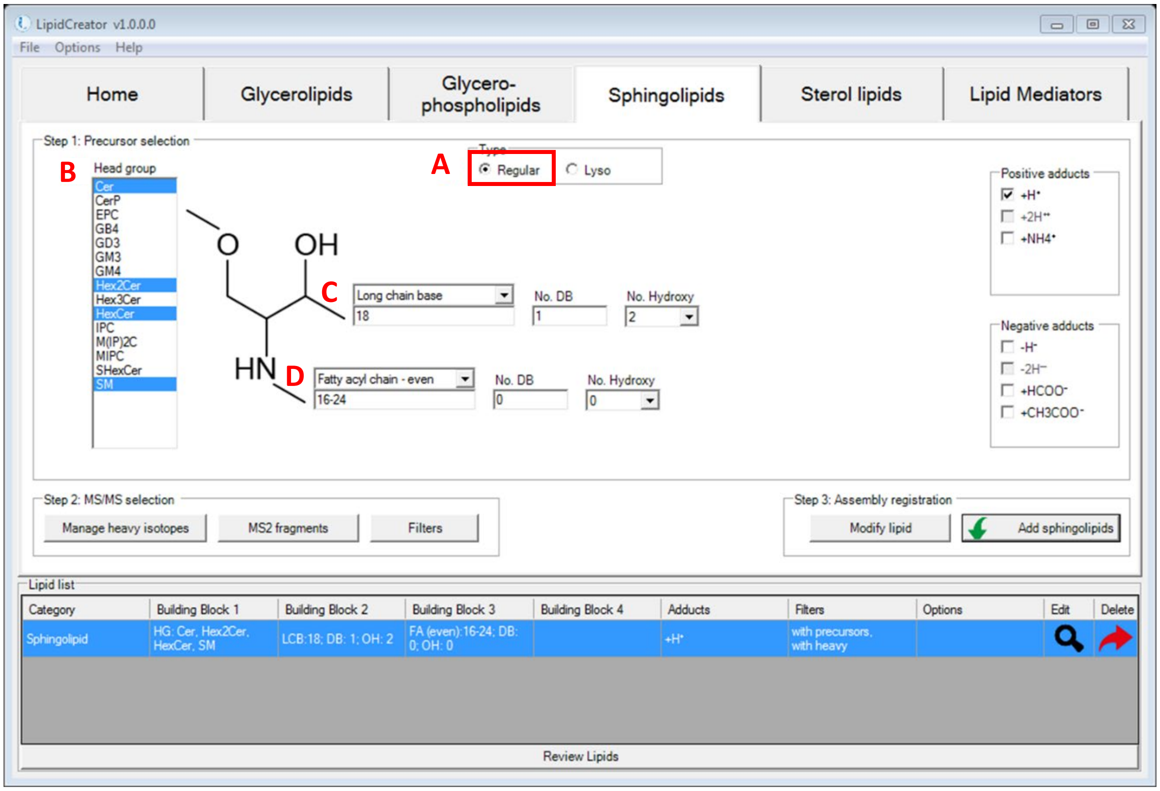

Supplementary Figure 23. Interface for the generation of transitions for sphingolipids.

A: Radio buttons switch between different backbones of sphingolipids. In this case, the ‘Regular’ type is selected.

B: Head group selection for sphingolipids in LipidCreator.

C: Long-chain base definition for sphingolipids.

D: The fatty acyl chain can be defined and parameterized.

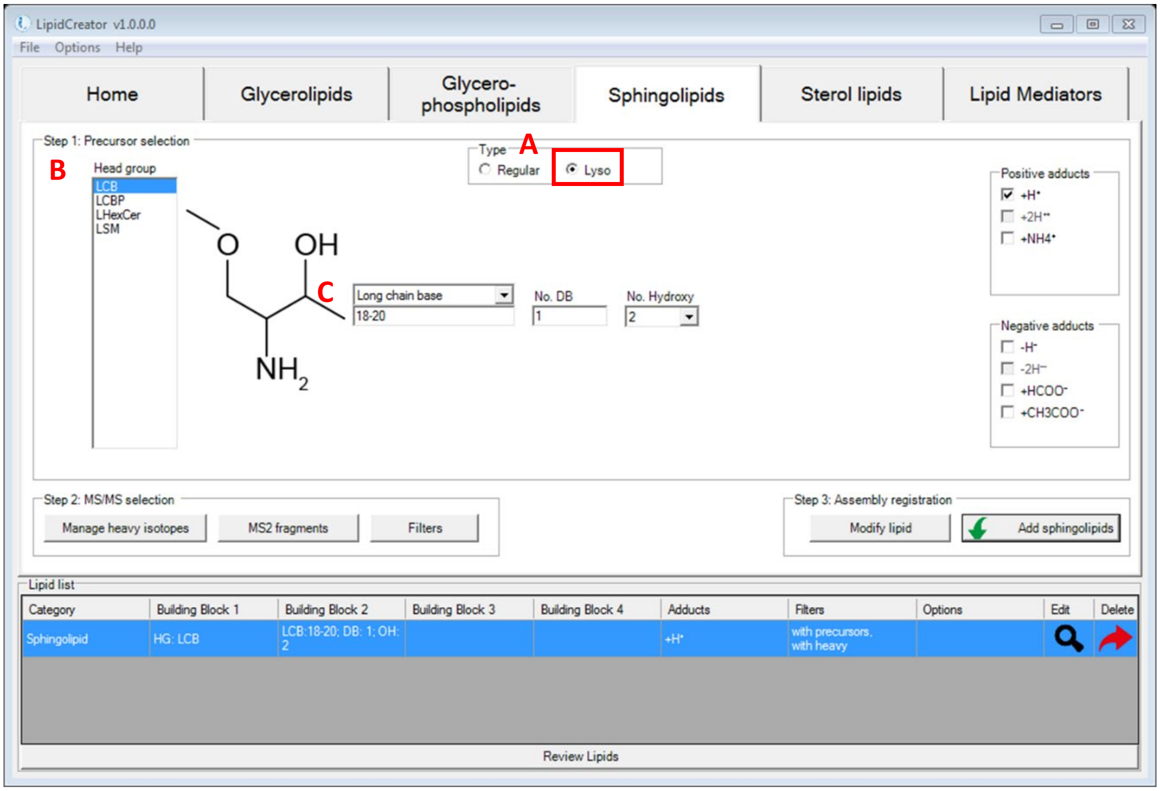

Supplementary Figure 24. Interface for the definition of transitions for lyso-sphingolipids.

A: Radio buttons switch between different backbones of sphingolipids. In this case, the ‘Lyso’ type is selected.

B: Head group selection for Lyso-sphingolipids in LipidCreator.

C: Long-chain base definition for sphingolipids.

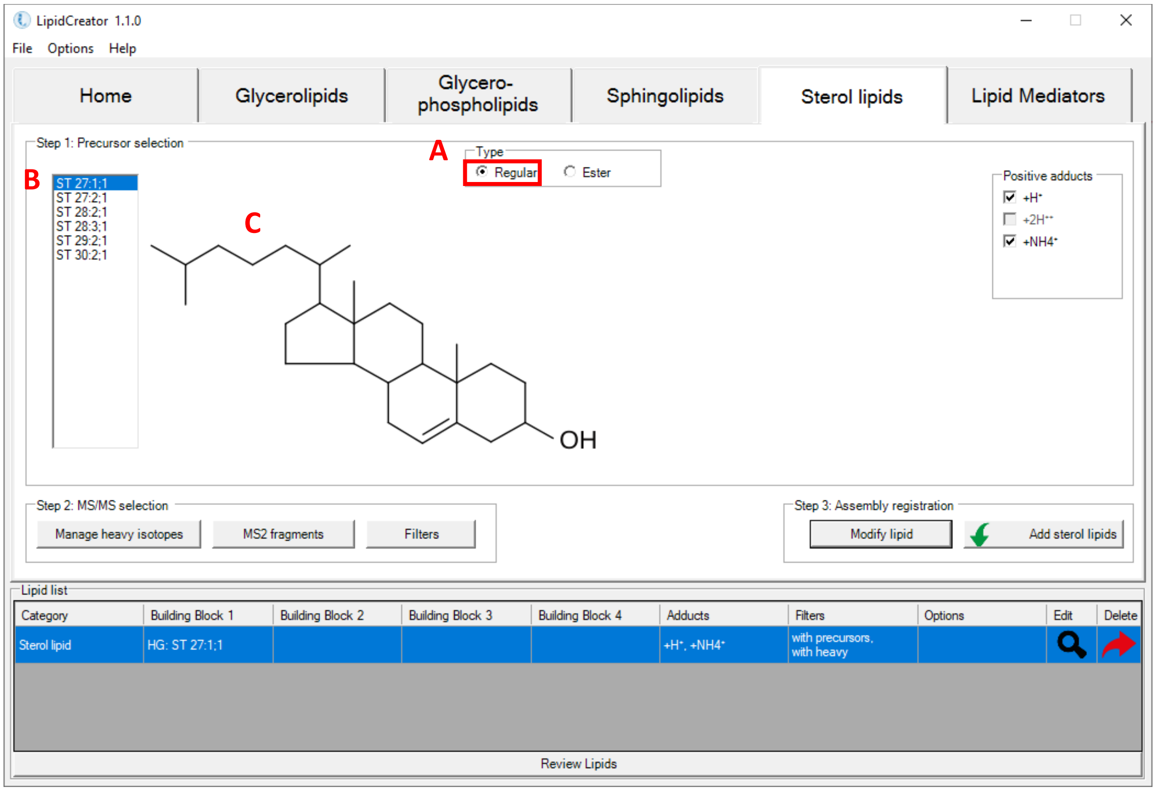

Supplementary Figure 25. Interface for the definition of transitions for sterols.

A: Radio buttons switch between regular sterols and sterol esters. In this case, the ‘Regular’ type is selected.

B: Backbone selection for sterol lipids in LipidCreator.

C: Cholesterol backbone. In this special case, no further parameterization is supported, apart from the adducts selection. Please see the next page for sterol esters.

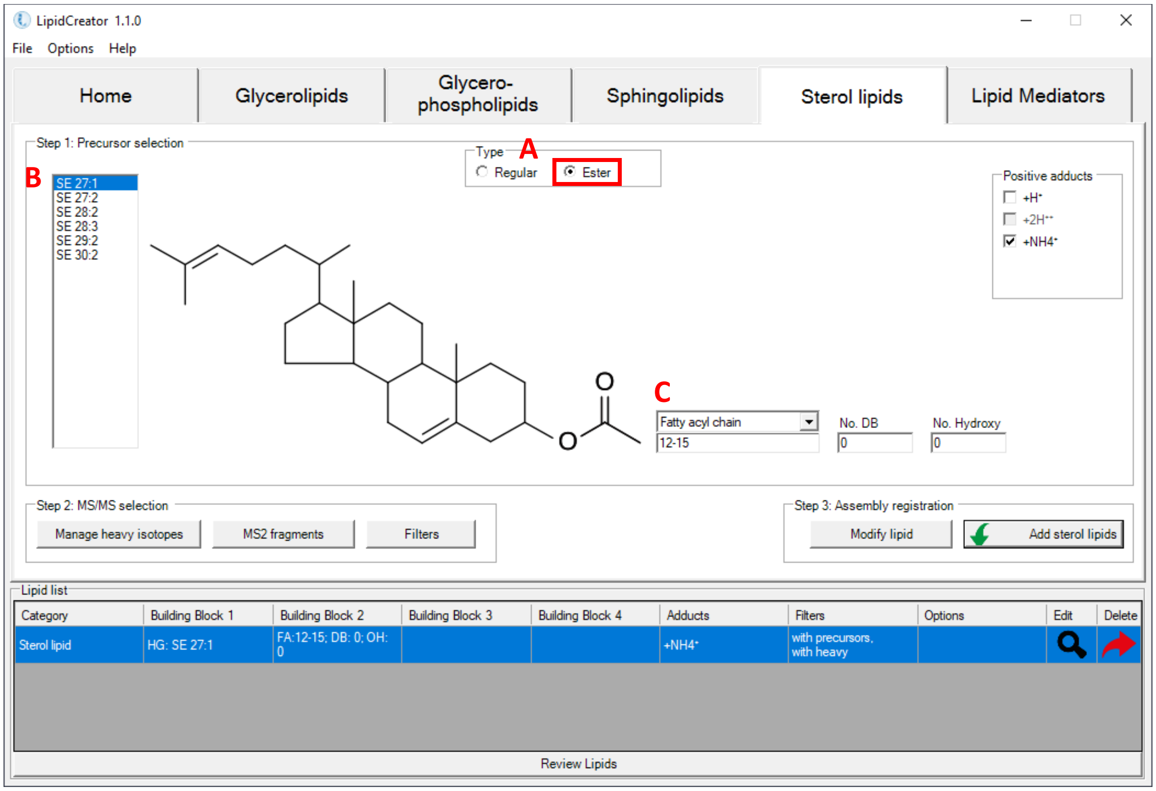

Supplementary Figure 26. Interface for the definition of transitions for sterol esters.

A: Selecting the ‘Ester’ radio button switches the ‘Sterol lipids’ tab to sterol ester mode.

B: Backbone selection for sterol ester lipids.

C: The fatty acyl chain can be defined and parameterized.

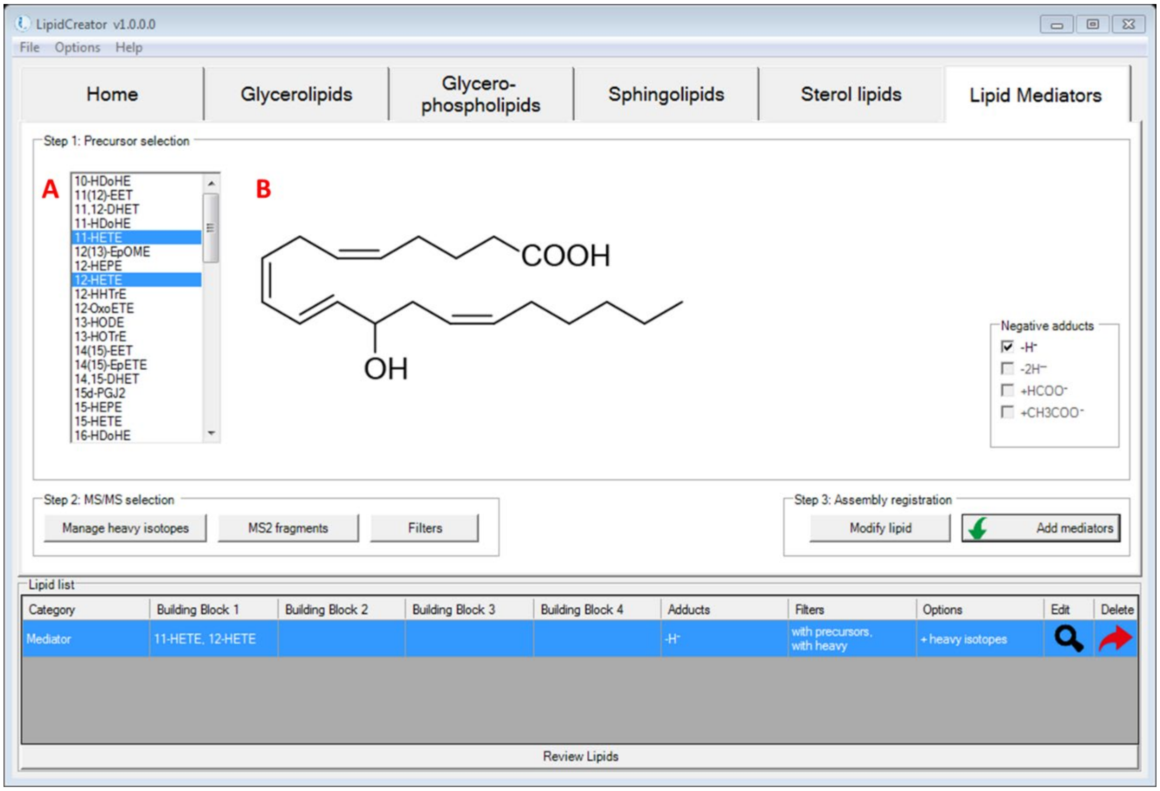

Supplementary Figure 27. Interface for the definition of transitions for lipid mediators.

A: Head group selection for lipid mediators in LipidCreator.

B: This area displays the chemical structure of each lipid mediator when hovering the mouse cursor over different mediator names.

Import/export lipid list/setting/project

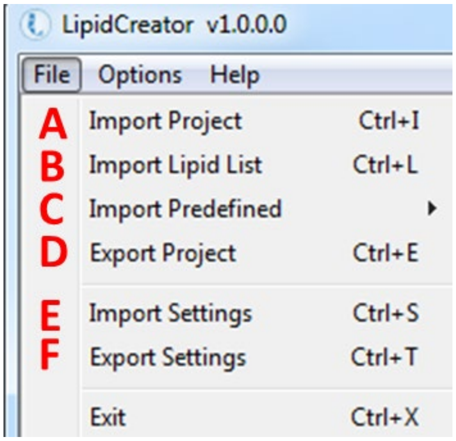

Supplementary Figure 28. File Menu of LipidCreator.

A: Import project into LipidCreator. A project includes the lipid list, user defined MS^2 fragments, user-defined heavy labelled isotopes, and a selection of optimal collision energies.

B: Import lipid list from *.csv file. The lipid list should follow the nomenclature described in Table S2. Otherwise, please use the lipid name translator (Lipid name translator) for import. In the *.csv file, lipid names (including adduct name) should be given one per line.

C: Import predefined lipid lists from previous work:

Yeast[3], Mouse brain[13, 19],

Mouse heart[11],

Mouse platelet[10],

Human platelet[10].

D: Export current project in *.lcXML format from LipidCreator for storage.

It is possible to add user-defined project into 'Predefined' when use LipidCreator standalone. Copy *.lcXML file into the folder (or create new folder) at…/LipidCreator/data/predefined.

E: Import settings in LipidCreator. Settings include user defined MS^2 fragments, user defined heavy labelled isotopes, and a selection of optimal collision energies.

F: Export the current settings in *.lcXML format.

Lipid name translator

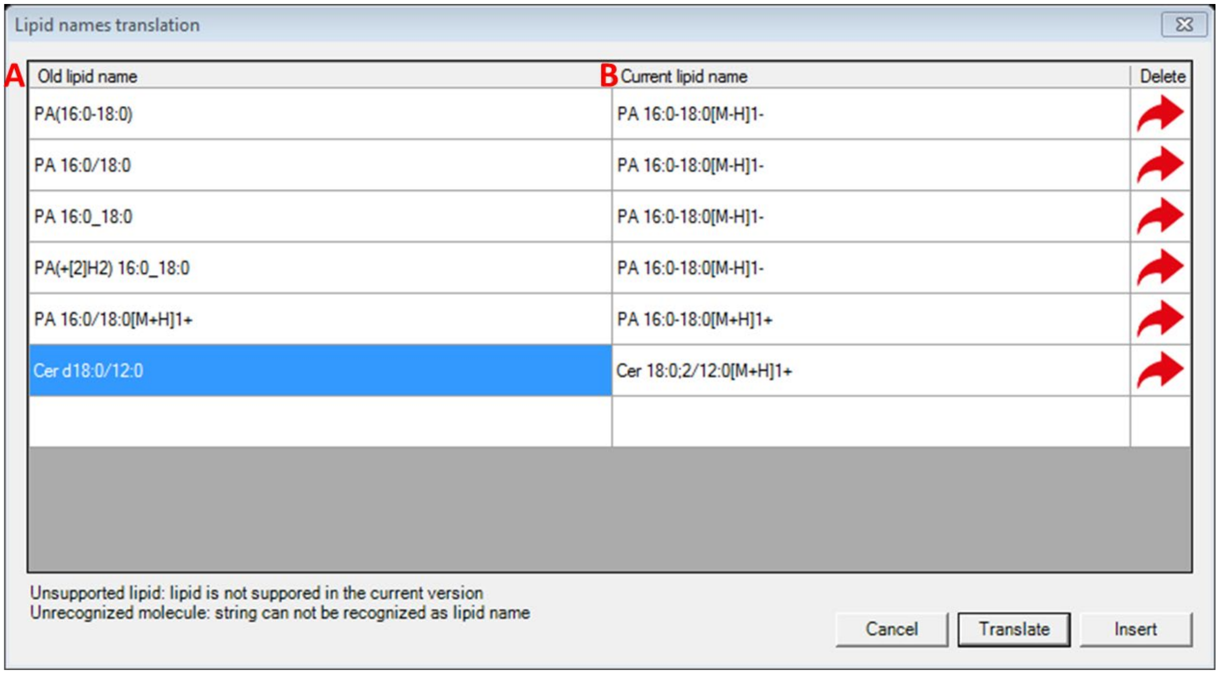

Supplementary Figure 29. Interface for the lipid name translator (Options→Lipid name translator).

A: List of old lipid names. Not all isotope formats can be recognized directly from the name. The isotope labels need to be defined additionally in LipidCreator.

B: List of translated lipid name according to the nomenclature in LipidCreator. When the old lipid name has no adduct defined, a default adduct will be appended to the translated name automatically. After translation, the list will be imported into LipidCreator by clicking on ‘Insert’.

Please see Supplementary Table 4 for more examples of the lipid name translator and Supplementary Data 3 for examples of the general lipid nomenclature which LipidCreator uses for labeling and naming of fragments.

Selection of MS2 fragments

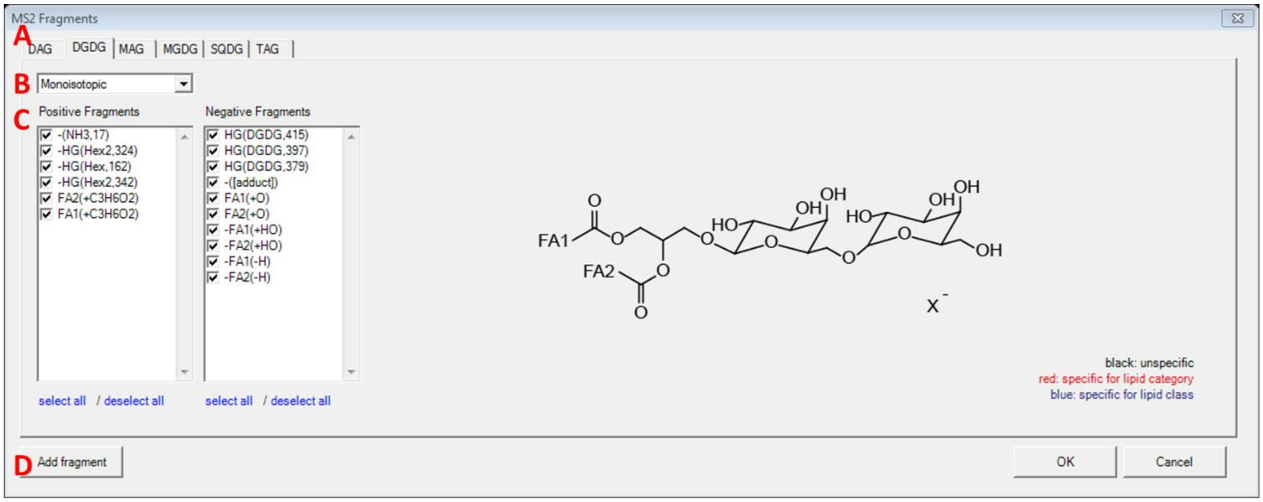

Supplementary Figure 30. Interface for MS^2 fragments for glycerolipids.

A: Tabs for selecting lipid classes.

B: Drop-down list for selecting either monoisotopic or isotopic species. Isotopic coded species will only show up after being defined (Managing heavy isotopes).

C: List of MS^2 fragment types of each lipid class for positive and negative mode. Each predefined fragment type offers a chemical structure preview on the right side.

D: Add user-defined fragments, please see Adding user-defined fragments.

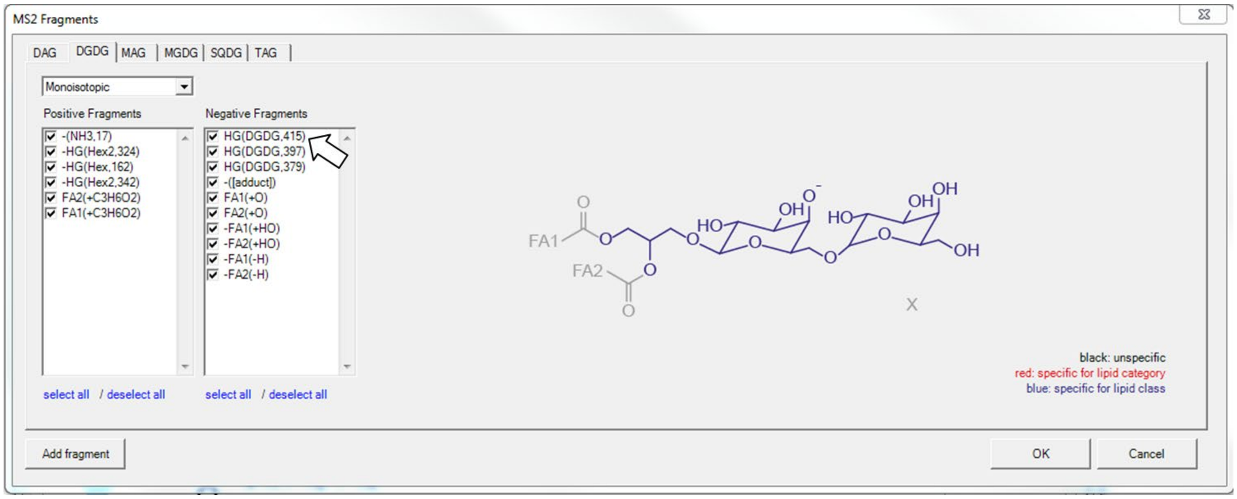

Supplementary Figure 31. Preview of MS^2 fragments for glycerolipids when hovering over fragment names.

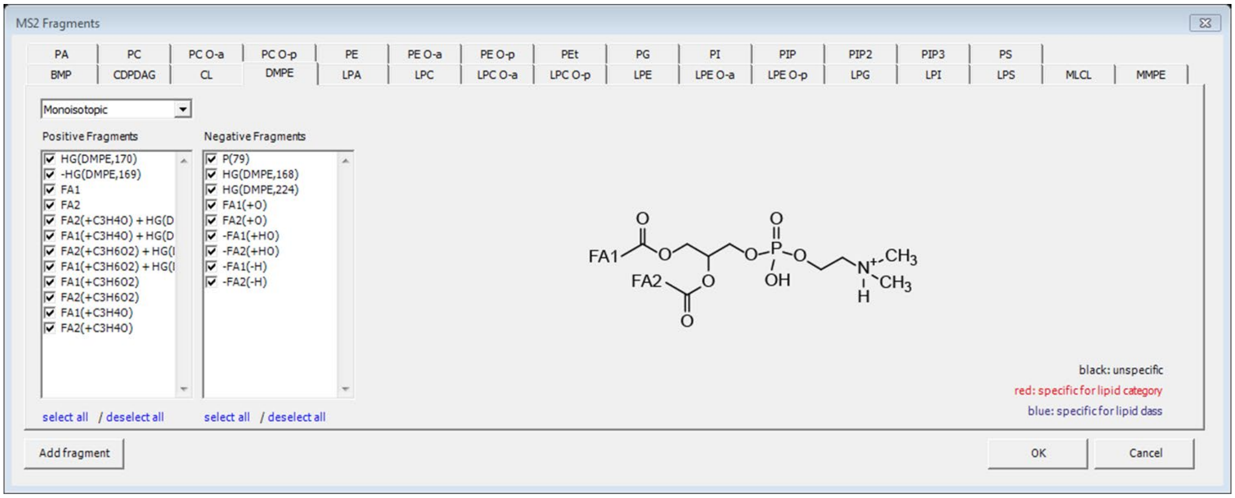

Supplementary Figure 32. Interface for MS^2 fragments for glycerophospholipids.

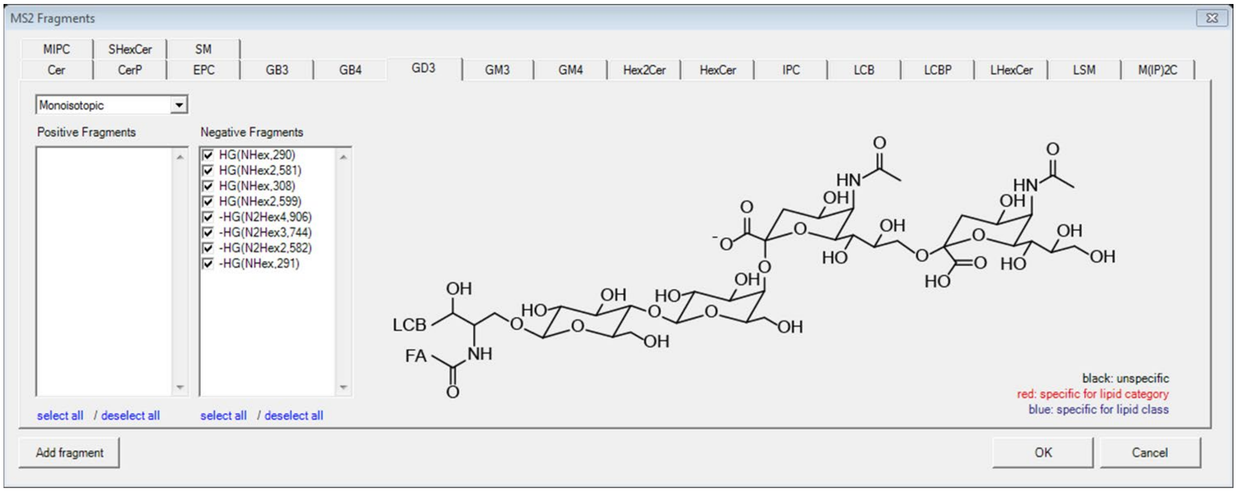

Supplementary Figure 33. Interface for MS^2 fragments for sphingolipids.

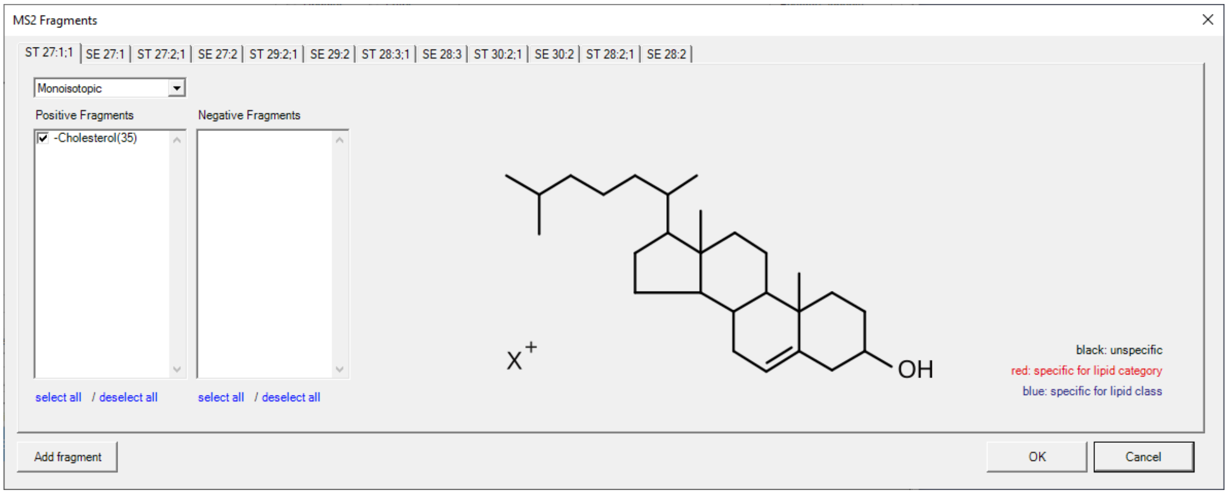

Supplementary Figure 34. Interface for MS^2 fragments for cholesterol and cholesterol esters.

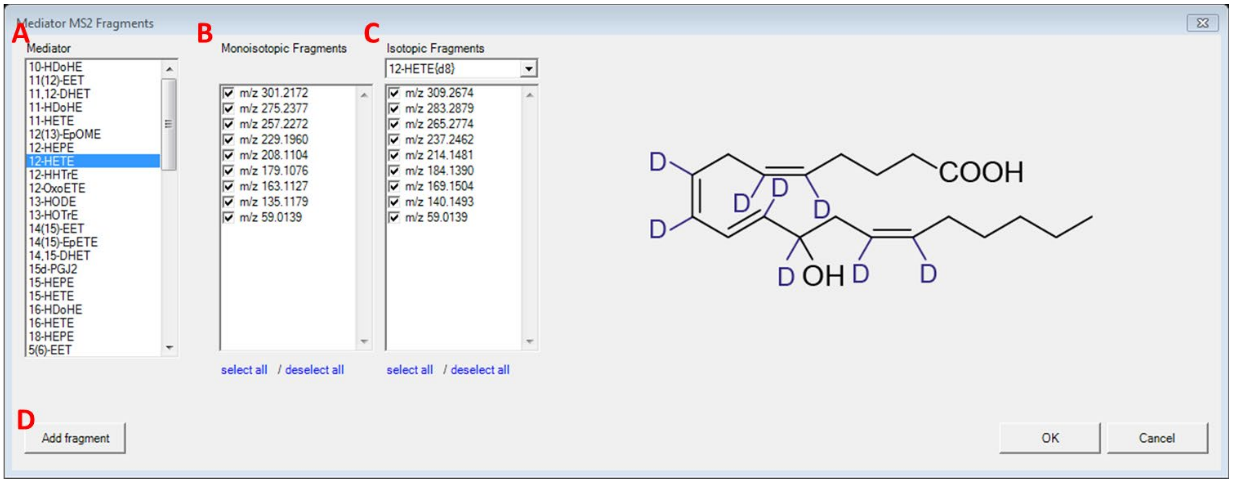

Supplementary Figure 35. Interface for MS^2 fragment masses for lipid mediators.

A: List of individual lipid mediators.

B: List of MS^2 fragment masses for selected lipid mediator.

C: List of MS^2 fragment masses for isotope labelled versions of the selected lipid mediator.

D: Adding user-defined fragments, please see Adding user-defined fragments for details.

Adding user-defined fragments

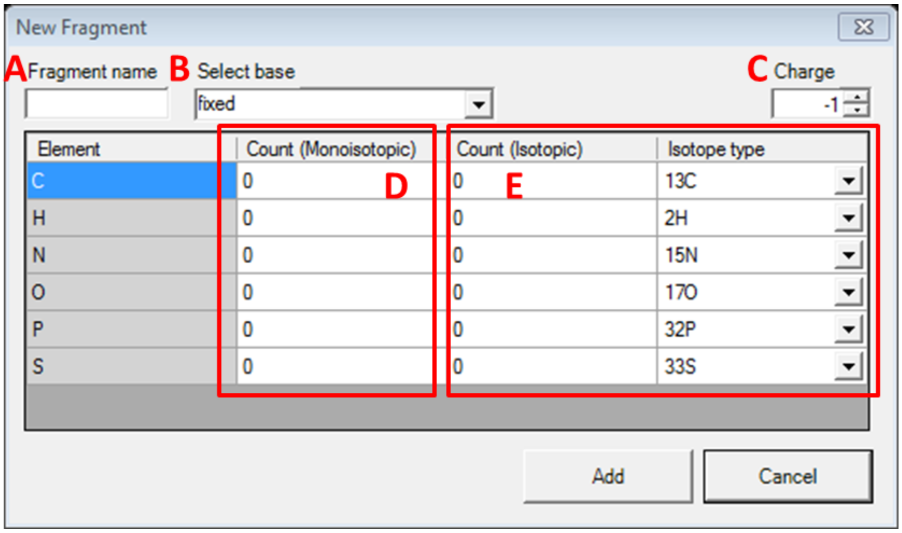

Supplementary Figure 36. Interface for adding new fragments.

A: User-defined fragment name.

B: Select base of the user-defined fragment type. Depending on the chosen lipid class, either the base can be fixed or it can contain building blocks, e.g. HG, HG + FA1, FA1 + FA2, etc.

C: Fragment charge. A positive value indicates that this fragment originated from positive ionization mode, while a negative value indicates that this fragment originated from negative ionization mode.

D: A constant set of elements can be defined which will be added to the fragment. When 'fixed' base is selected, element numbers can only be positive; otherwise negative counts are also allowed.

E: A field to input constant set of isotopic elements and drop-down list to select the type of isotopic elements.

The next page shows how to add new types of fragments for PG. Supplementary Data 4 provides examples for custom MS2 fragments used in LipidCreator.

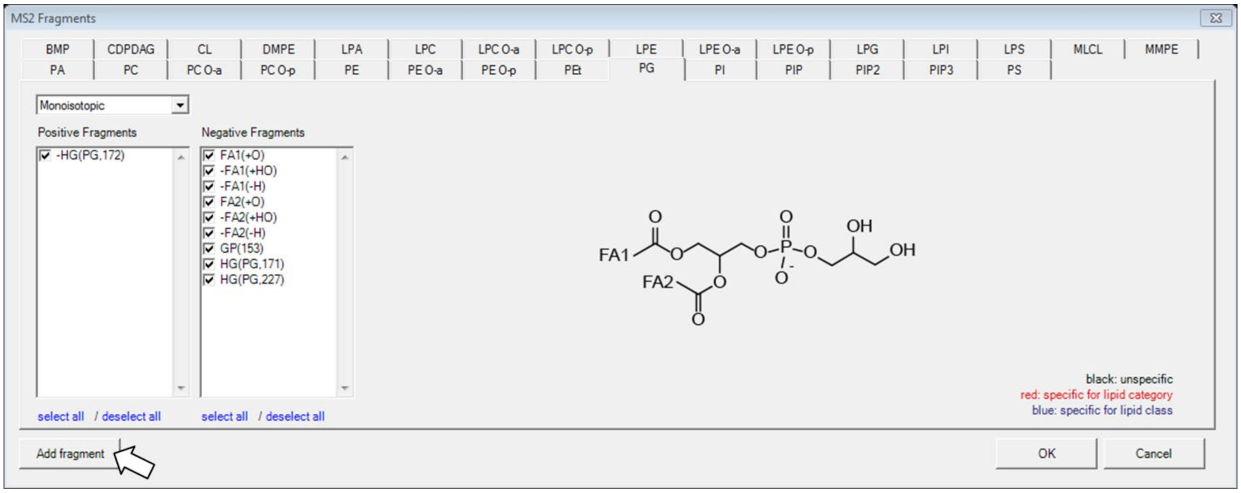

Supplementary Figure 37. Click on ‘Add fragment’ from the PG tab of glycerophospholipids.

This will preselect the ‘PG’ class as the target class for the new fragment.

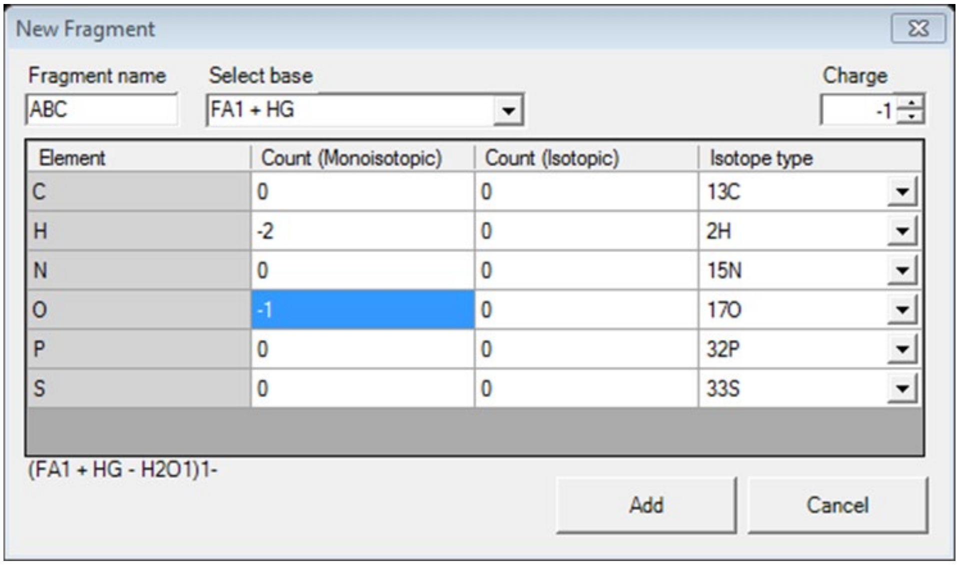

Supplementary Figure 38. Create a new fragment ‘ABC’ for PG class.

In order to add a new fragment, enter its (unique) name, select the proper base and charge. Then, define the elemental composition of the fragment by entering the respective atom counts, either monoisotopic, or isotopic if you want to define a heavy labeled fragment. Click ‘Add’ to add the fragment to LipidCreator’s knowledge base. You can now include this new fragment within the ‘MS2 Fragments’ dialog.

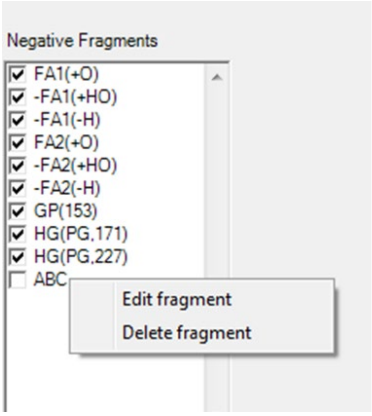

Supplementary Figure 39. The ‘ABC’ fragment has been added to the fragment list.

User-defined fragments can be edited or deleted by right clicking on the fragment name.

Example: adding a new mediator fragment

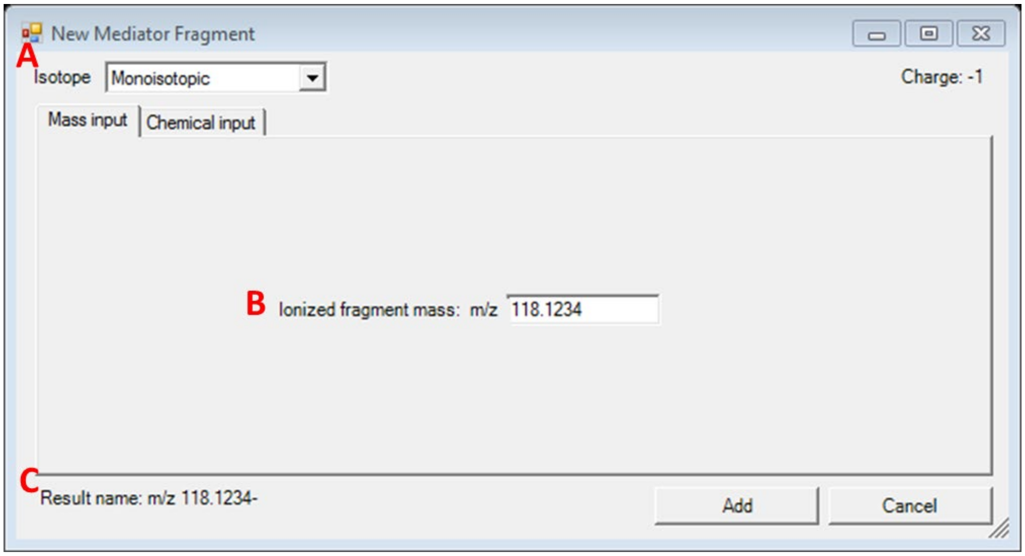

Supplementary Figure 40. Adding a monoisotopic mediator fragment with direct mass input for Mediator.

A: In the ‘Isotope’ drop-down list, either monoisotopic or other isotopes can be selected to add a fragment, depending on the mediator selection in Supplementary Figure 28.

B: In the ‘Mass Input’ tab, please directly type in the ionized fragment mass. The fragment charge is set to -1 by default.

C: The preview for display name of the fragment.

The mass will appear in the transition list with exactly this value and will be identical in the current list. Identified replicates will be denied when adding.

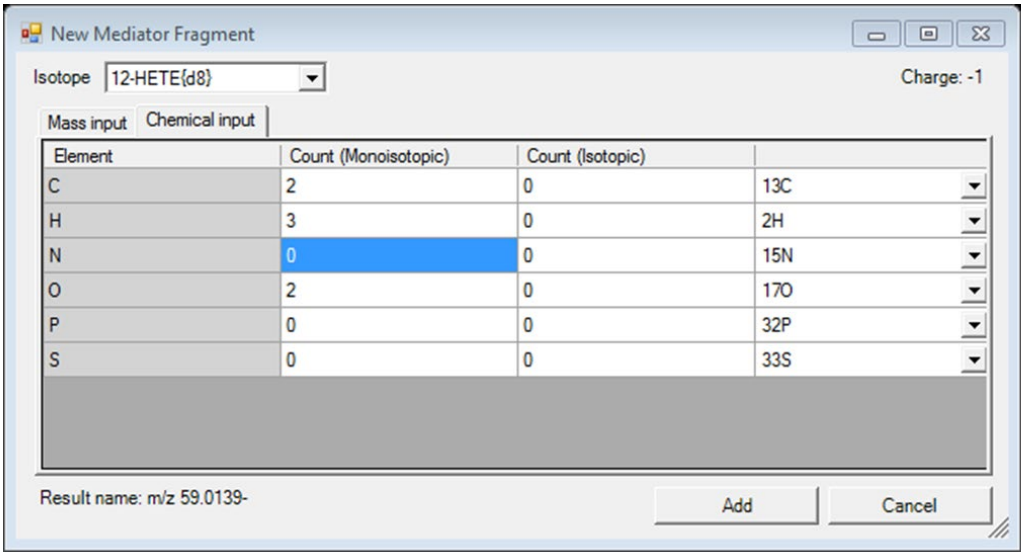

Supplementary Figure 41. Adding a fragment using the chemical formula input for Mediator.

To add a fragment for a monoisotopic species, use only the 'Count (Monoisotopic)' column to enter the counts of elements. For heavy isotope species, both 'Count (Monoisotopic)' and 'Count (Isotopic)' are valid. The result name will be displayed as the (m/z) mass.

Managing heavy isotopes

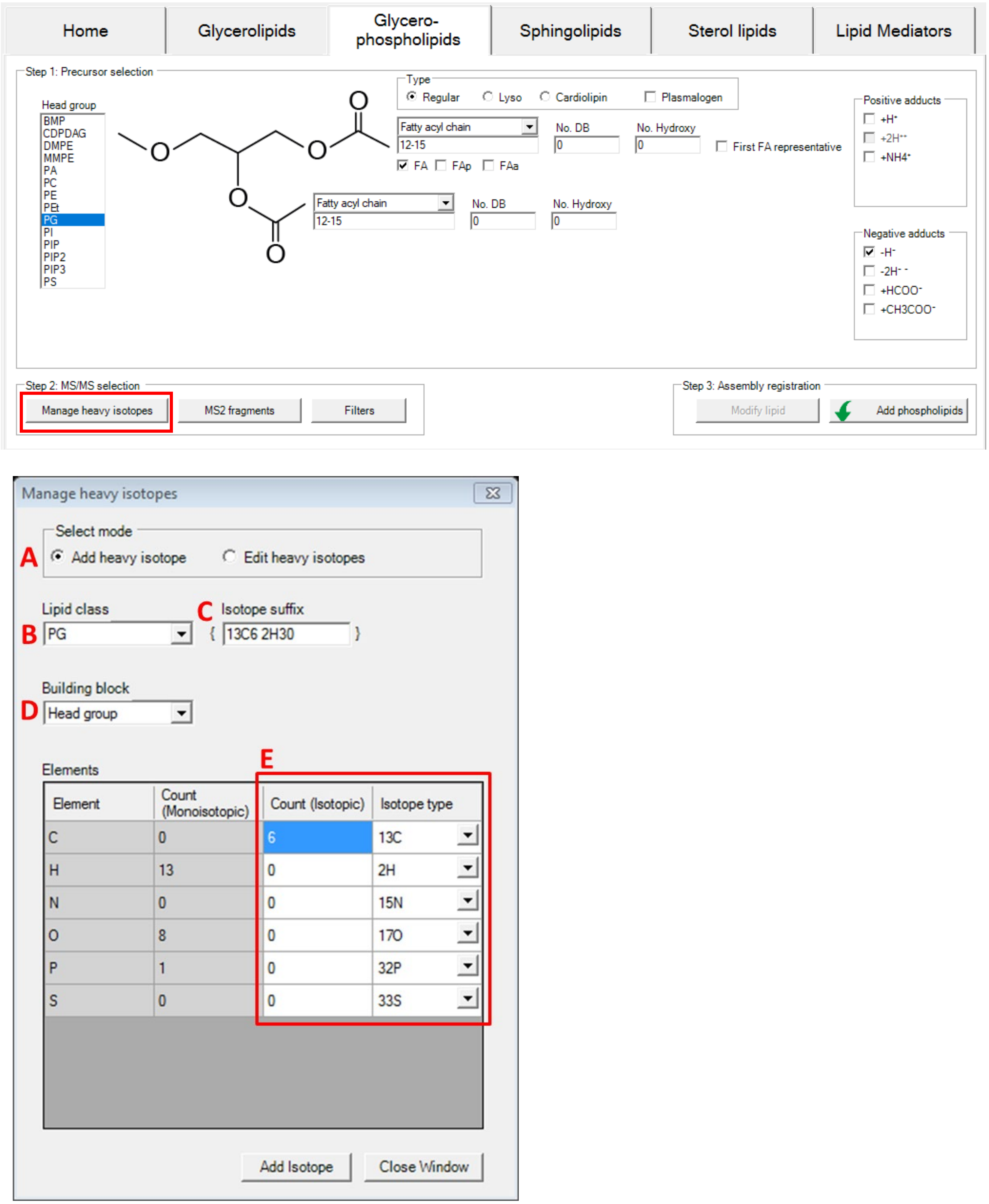

Supplementary Figure 42. Interface for managing heavy isotopes for glycerophospholipids.

This interface is automatically adapted to currently selected lipid category tab. It is accessible from the ‘Manage heavy isotopes’ button in the ‘Step 2: MS/MS selection’ pane of each of the lipid category tabs within the LipidCreator user interface.

A: Radio buttons for either adding new isotopes or editing existing user-defined isotopes.

B: Drop-down list for selecting the lipid class (depending on the lipid category).

C: User-defined name as suffix for the lipid class.

D: Drop-down list for selecting building blocks.

E: A field to input constant set of isotopic elements. The count for monoisotopic elements will be automatically changed according to the typed count of isotopic elements.

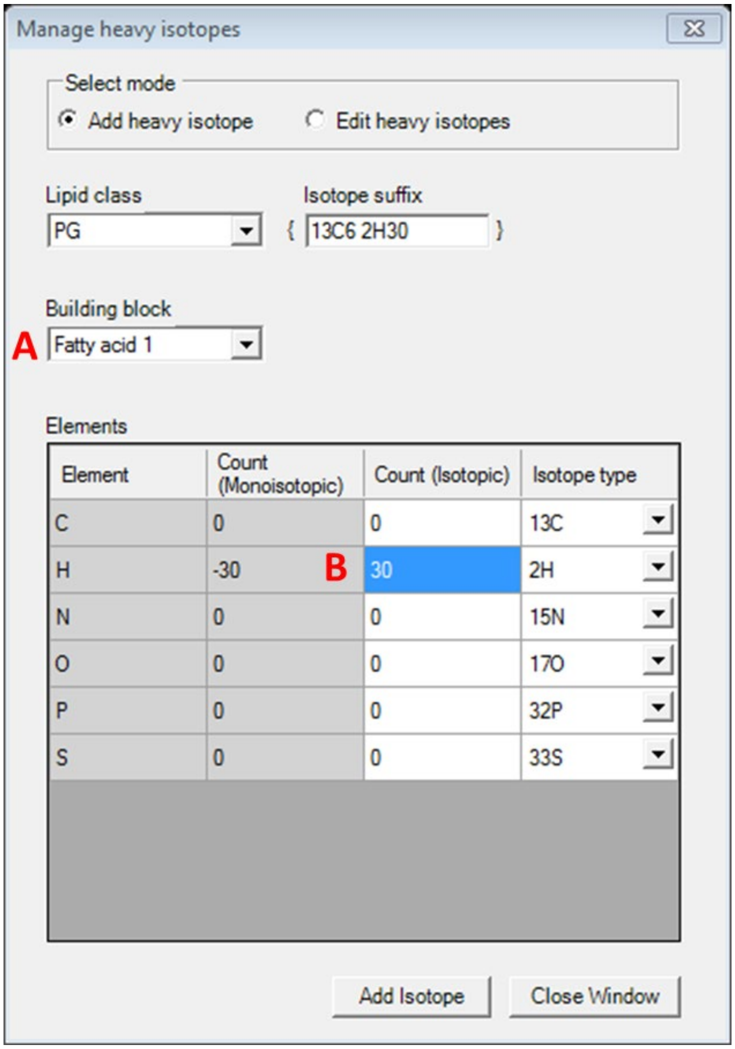

Supplementary Figure 43. Interface for managing heavy isotopes for glycerophospholipids.

A: Select ‘Fatty acyl 1’ to add isotope elements.

B: The heavy-labelled element numbers act as an upper limit for the element, since the fatty acyl building block has a variable number of elements depending e.g. on the carbon chain length.

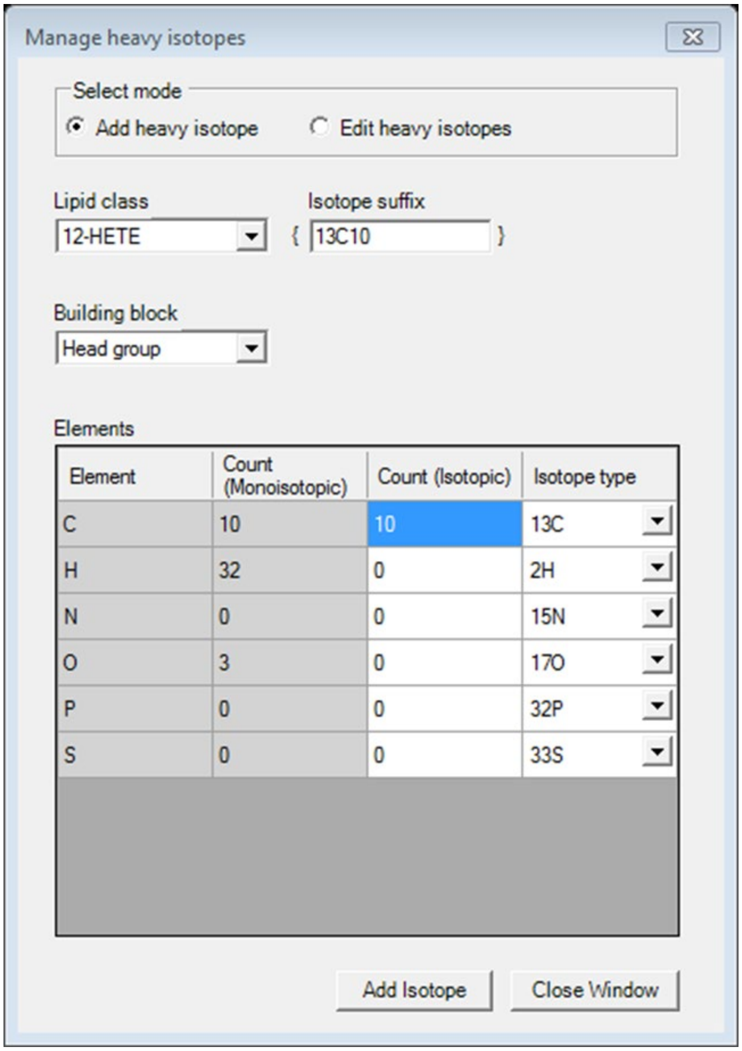

Supplementary Figure 44. Interface for managing heavy isotopes for mediators.

The chemical formula (numbers of elements in ‘Count (Monoisotopic)’ column) for all mediators are provided when adding heavy isotopes. The only selectable building block is the ‘Head group’ in this case, which entails the complete mediator molecule.

Filters for the transition list

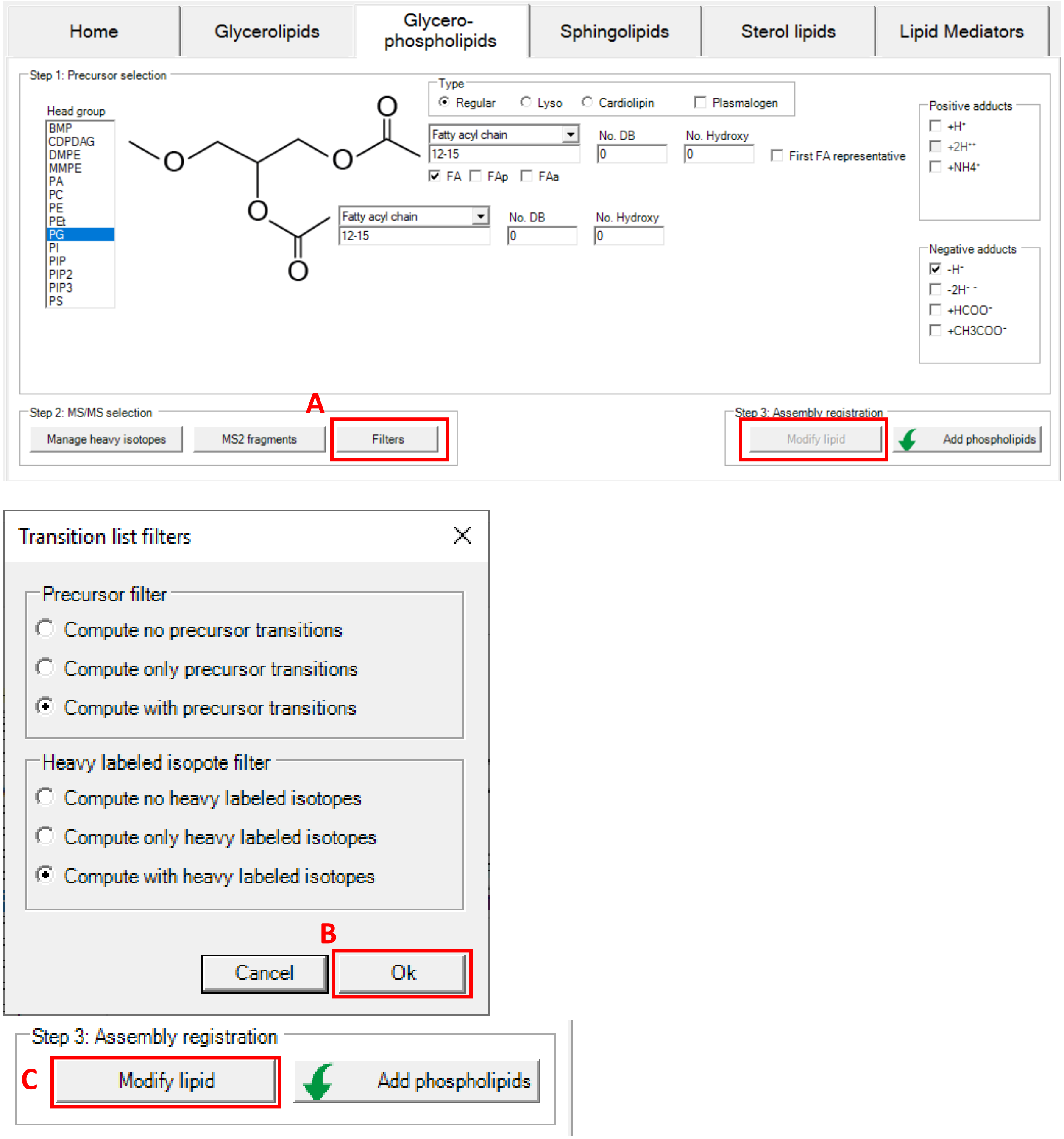

Supplementary Figure 45. Filters are applied before the transition lists are calculated.

After changing the transition list filter settings (A), e.g. to exclude precursor transitions, click ‘Ok’ (B), then click on ‘Modify lipid’ (C) to update the ‘Lipid list’. Note: the button (C) is only enabled when you have at least one lipid of the current tab / lipid category in your ‘Lipid list’.

Collision energy optimization function

The collision energy optimization function is currently only available for lipid mediators. To activate this function, first click on Options→ 'Collision Energy Computation'→ 'Thermo Scientific Q Exactive HF' or 'Agilent 6545 Q-TOF LC/MS'.

Afterwards, open the dialog from Options→ 'Collision Energy optimization'.

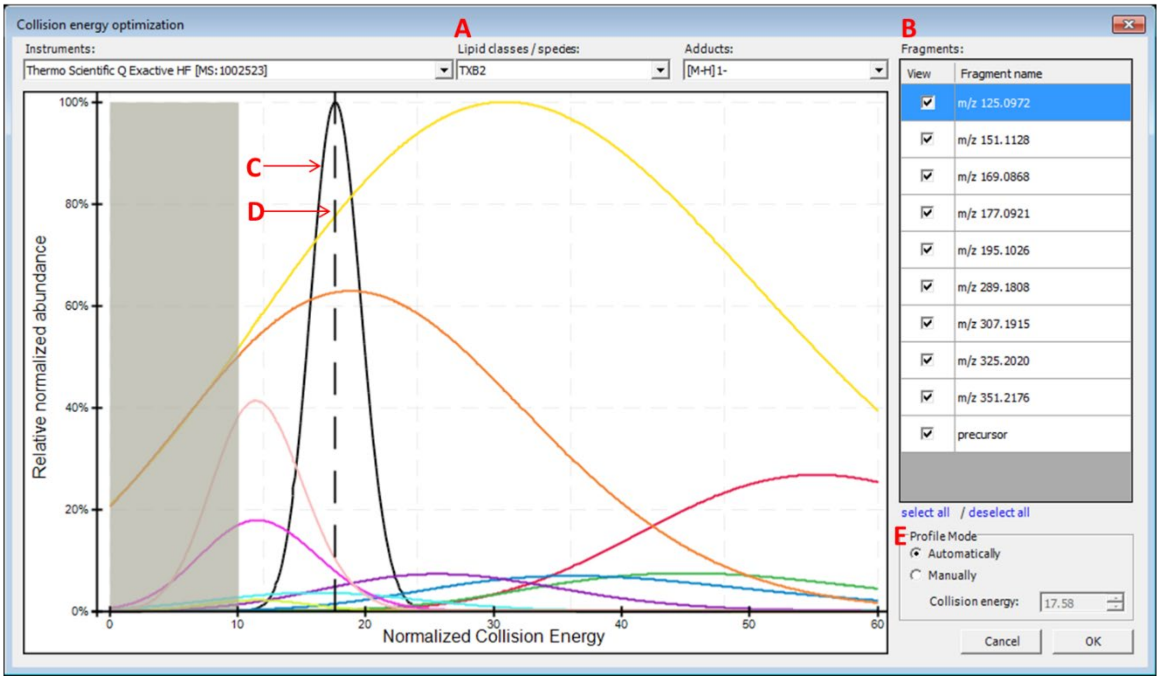

Supplementary Figure 46. Collision energy optimization interface.

A: Choose a lipid mediator species from the drop-down list. Isotopes appear as separate entries to their monoisotopic counterparts.

B: Fragment list for the chosen lipid species. Optimal collision energy depends on fragment selection.

C: The black curve is the automatically calculated product distribution over all selected fragment distributions from the list. Its mode (center) indicates the optimal collision energy over all selected fragments.

D: The dashed line indicates the chosen collision energy. This line is moveable in manual profile mode.

E: Automatic or manual profile mode can be selected. When the manual profile mode is activated, either move the dashed line with the mouse or type in a collision energy value of your choice.

Activating the computation of collision energy is independent from added lipid species in the ‘Lipid list’. The collision energy can be defined before or after lipid assembly, these two pieces of information will be combined after clicking on ‘Review Lipids’.

By applying collision energy computation, the CE value can be used either to generate an MS method from the transition list or to provide corresponding relative intensities for fragments in the generated *in silico* spectral library.

Reviewing the lipid transition list

After clicking on 'Review Lipids', the calculated lipid precursor names (Supplementary Figure 47) will be listed for further selecting/deselecting before calculation of the final transitions (Supplementary Figure 48 & 49).

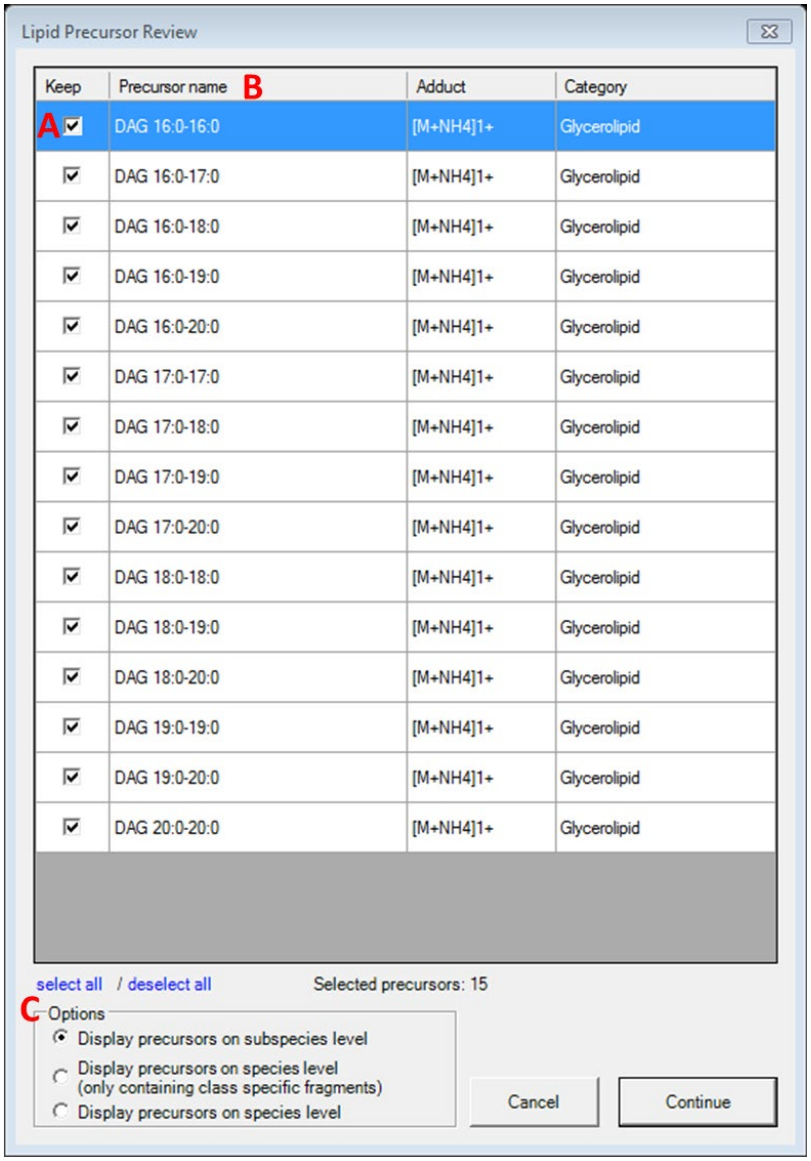

Supplementary Figure 47. Interface for reviewing the name of calculated lipid precursors.

A: The checkbox allows you to select/deselect the generated lipids for further calculation of transitions.

B: Click on the name of each column to activate sort ascending/descending functions.

C: Display options between subspecies and species level of lipid name. e.g. DAG 16:0-16:0 in subspecies level, DAG 32:0 in species level.

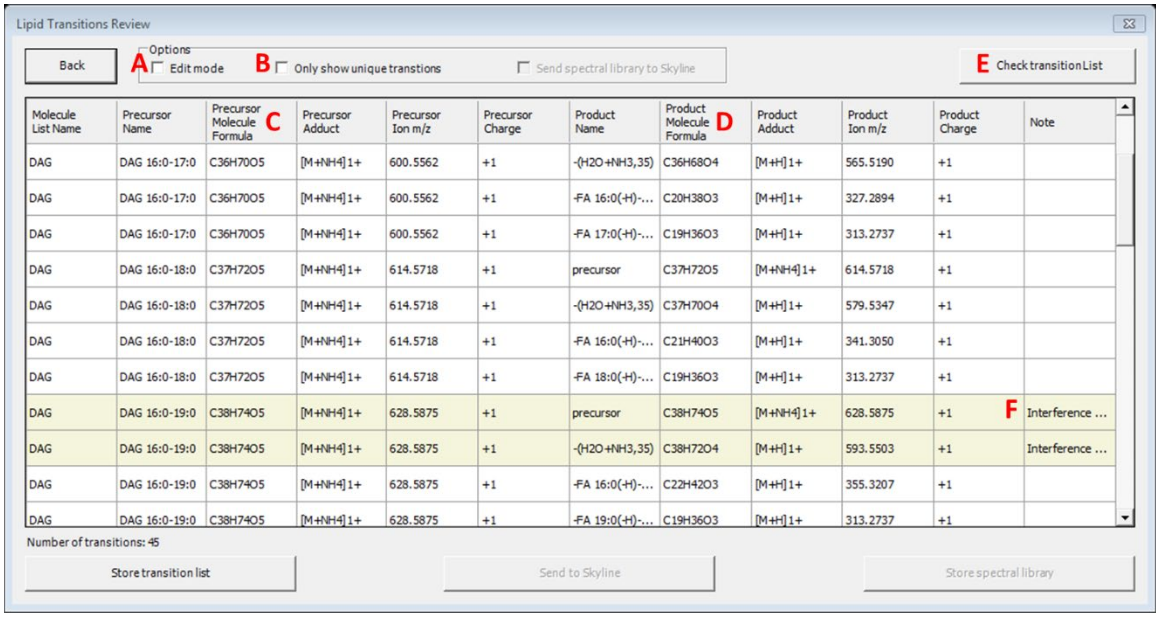

Supplementary Figure 48. Interface for reviewing calculated lipid transitions.

A: The checkbox allows you to activate user's edit mode for manually editing, adding and deleting items within the transition list.

B: The checkbox allows you to discard the non-unique transitions from the review panel and further actions.

C & D: The chemical formulas in the transition list represent neutral lipids/fragments. The masses represent ionized precursors/fragments after adding the corresponding adducts.

E: This button is to check whether the current transition list is compatible with Skyline.

F: In the review list, the non-unique transitions (only the precursor and product masses are considered) will be highlighted and noted when being repeated. The note will be imported to Skyline when the transition list is being sent.

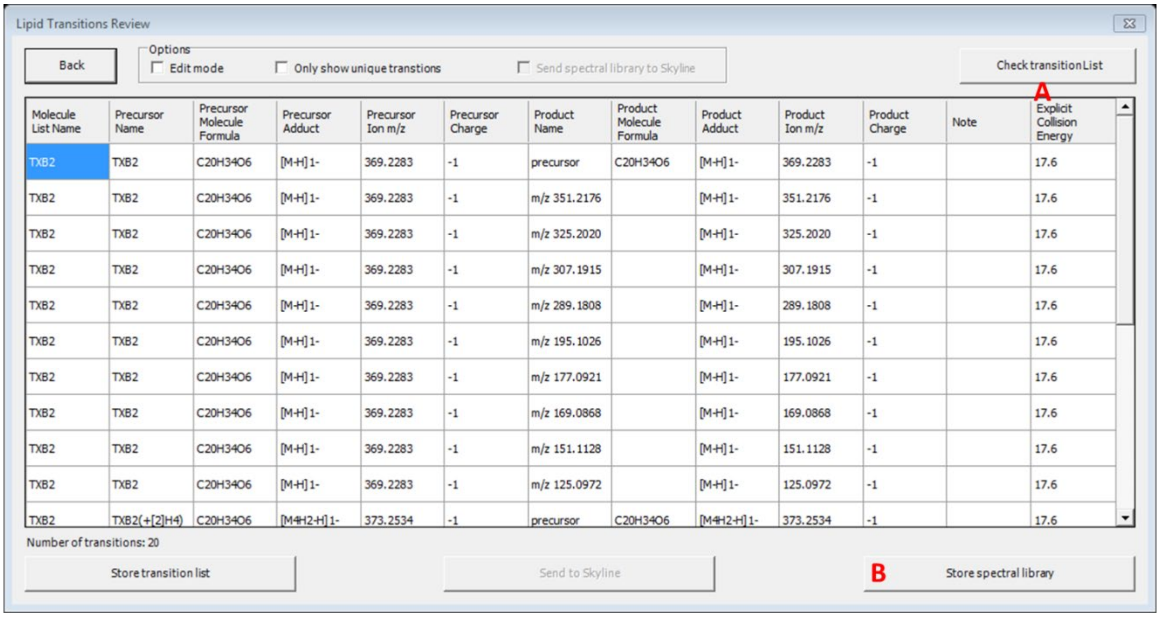

Molecule List Name	Precursor Name	Precursor Molecule Formula	Precursor Adduct	Precursor Ion m/z	Precursor Charge	Product Name	Product Molecule Formula	Product Adduct	Product Ion m/z	Product Charge	Note	Explicit Collision Energy
TXB2	TXB2	C20H34O6	[M-H]1-	369.2283	-1	precursor	C20H34O6	[M-H]1-	369.2283	-1		17.6
TXB2	TXB2	C20H34O6	[M-H]1-	369.2283	-1	m/z 351.2176		[M-H]1-	351.2176	-1		17.6
TXB2	TXB2	C20H34O6	[M-H]1-	369.2283	-1	m/z 325.2020		[M-H]1-	325.2020	-1		17.6
TXB2	TXB2	C20H34O6	[M-H]1-	369.2283	-1	m/z 307.1915		[M-H]1-	307.1915	-1		17.6
TXB2	TXB2	C20H34O6	[M-H]1-	369.2283	-1	m/z 289.1808		[M-H]1-	289.1808	-1		17.6
TXB2	TXB2	C20H34O6	[M-H]1-	369.2283	-1	m/z 195.1026		[M-H]1-	195.1026	-1		17.6
TXB2	TXB2	C20H34O6	[M-H]1-	369.2283	-1	m/z 177.0921		[M-H]1-	177.0921	-1		17.6
TXB2	TXB2	C20H34O6	[M-H]1-	369.2283	-1	m/z 169.0868		[M-H]1-	169.0868	-1		17.6
TXB2	TXB2	C20H34O6	[M-H]1-	369.2283	-1	m/z 151.1128		[M-H]1-	151.1128	-1		17.6
TXB2	TXB2	C20H34O6	[M-H]1-	369.2283	-1	m/z 125.0972		[M-H]1-	125.0972	-1		17.6
TXB2	TXB2(+[2]H4)	C20H34O6	[M4H2-H]1-	373.2534	-1	precursor	C20H34O6	[M4H2-H]1-	373.2534	-1		17.6

Supplementary Figure 49. Interface for reviewing calculated lipid transitions with collision energy.

A: The ‘Explicit Collision Energy’ column will appear after collision energy computation has been activated.

B: The ‘Store spectral library’ button is valid after collision energy computation has been activated.

Storing a transition list

Following the steps described in Reviewing the lipid transition list, the reviewed transition list can be exported as a *.csv or *.xls file by clicking on ‘Store transition list’. The list can be stored in either one full list or it can be split into two lists separated by polarity mode.

Storing a spectral library

The creation of a spectral library is possible after collision energy computation is activated according to Collision energy optimization function. Spectral libraries are written in Skyline *.blib format, which is an SQLIte database file.

Integration with Skyline

1. Installation of Skyline

Please install Skyline through [https://skyline.ms/project/home/software/Skyline/begin.view]

2. Install LipidCreator in Skyline

Please install LipidCreator from Skyline Tools→Tool store.

Or

Please add the downloaded .zip file to Skyline through Tools→ External Tools→ Add→ From file…→ Choose LipidCreator.zip→ Wait until LipidCreator shows in the ‘Menu contents’ (this step may take some seconds)→ OK.

3. Create a transition list for Skyline

Start LipidCreator from Tools and use it as described in Overview of the LipidCreator User Interface and the following sections. After the created transition list has been reviewed, click on ‘Send to Skyline’. The lipid list will appear in the ‘Targets’ window in Skyline. Then either export the project (see Import/export lipid list/setting/project) for further editing or close LipidCreator directly.

4. Create *In silico* spectral library

After activating the collision energy computation (see Collision energy optimization function), the created transition list and spectral library can be sent to Skyline directly (Supplementary Figure 50).

Or

Add the *.blib file manually through Settings→Peptide Settings→Library→Edit list→Add

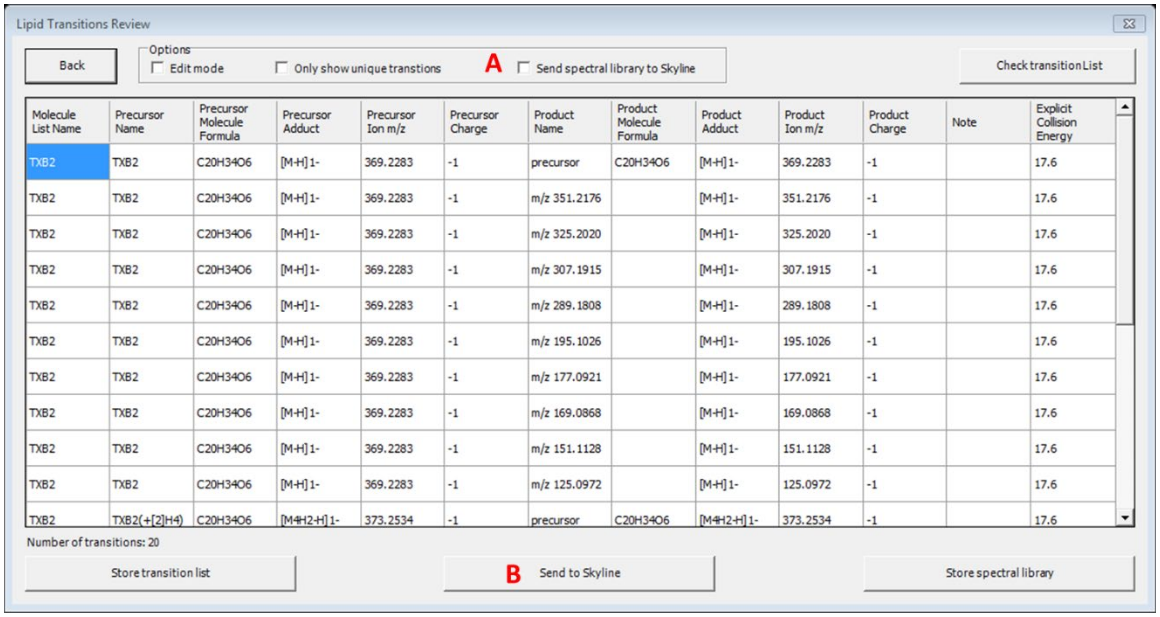

Molecule List Name	Precursor Name	Precursor Molecule Formula	Precursor Adduct	Precursor Ion m/z	Precursor Charge	Product Name	Product Molecule Formula	Product Adduct	Product Ion m/z	Product Charge	Note	Explicit Collision Energy
TXB2	TXB2	C20H34O6	[M-H]1-	369.2283	-1	precursor	C20H34O6	[M-H]1-	369.2283	-1		17.6
TXB2	TXB2	C20H34O6	[M-H]1-	369.2283	-1	m/z 351.2176		[M-H]1-	351.2176	-1		17.6
TXB2	TXB2	C20H34O6	[M-H]1-	369.2283	-1	m/z 325.2020		[M-H]1-	325.2020	-1		17.6
TXB2	TXB2	C20H34O6	[M-H]1-	369.2283	-1	m/z 307.1915		[M-H]1-	307.1915	-1		17.6
TXB2	TXB2	C20H34O6	[M-H]1-	369.2283	-1	m/z 289.1808		[M-H]1-	289.1808	-1		17.6
TXB2	TXB2	C20H34O6	[M-H]1-	369.2283	-1	m/z 195.1026		[M-H]1-	195.1026	-1		17.6
TXB2	TXB2	C20H34O6	[M-H]1-	369.2283	-1	m/z 177.0921		[M-H]1-	177.0921	-1		17.6
TXB2	TXB2	C20H34O6	[M-H]1-	369.2283	-1	m/z 169.0868		[M-H]1-	169.0868	-1		17.6
TXB2	TXB2	C20H34O6	[M-H]1-	369.2283	-1	m/z 151.1128		[M-H]1-	151.1128	-1		17.6
TXB2	TXB2	C20H34O6	[M-H]1-	369.2283	-1	m/z 125.0972		[M-H]1-	125.0972	-1		17.6
TXB2	TXB2(+[2]H4)	C20H34O6	[M4H2-H]1-	373.2534	-1	precursor	C20H34O6	[M4H2-H]1-	373.2534	-1		17.6

Supplementary Figure 50. Interface for reviewing calculated lipid transition with collision energy on Skyline platform.

A. This check box allows you to send the spectral library to Skyline without additionally needing to save *.blib file to your local drive.

B. By clicking on this button, transitions (and spectral library) will be sent to Skyline.

5. Export MS method from Skyline

The final MS method can be generated from Skyline by selecting favored MS vendor/type.

6. Viewing LipidCreator log messages in Skyline

LipidCreator displays warnings and errors directly to the user as dialogs. However, detailed error messages are also logged to the Tools→Immediate Window in Skyline and into a log file “lipidcreator.log” below the Tools\LipidCreator\data directory of the Skyline installation.

7. Review PRM/DIA results with spectral matching

To review transitions including precursor, please set Transition setting→Filter→Ion types: f,p.

Select *in silico* spectral library through Settings→Peptide Settings→Library. Then click on Library Match from View menu. After import data, extracted MS2 spectral through click on chromatographic peak.

Statistics for LipidCreator launching

When initially launching LipidCreator, a dialog will appear with the following message:

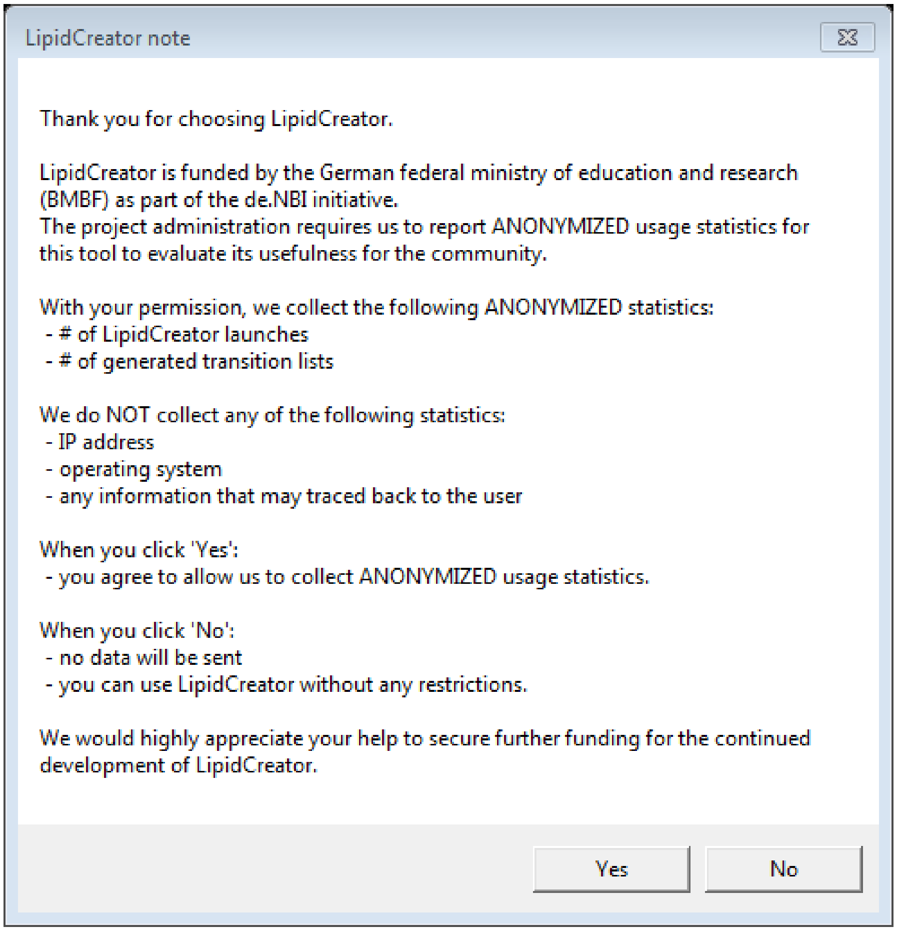

Supplementary Figure 51. Note when launching LipidCreator for the first time.

If the user changes his/her mind after clicking on 'Yes' or 'No', this function can be turned off/on at any time by (de)selecting Options→ 'Send anonymous statistics'.

Command line usage

LipidCreator has a comprehensive command line interface allowing the user to run several tasks without a graphical user interface. Therefore, it is suitable for an integration into automated pipeline frameworks capable of adding customized processing nodes[20, 21] or running command line executions[22] Launching LipidCreator from the command line with the argument “help” prints the following output:

```
> .\LipidCreator.exe help
usage: LipidCreator.exe (option)

options are:
  dev:                  launching LipidCreator as developer
  transitionlist:       creating transition list from lipid list
  translate:            translating a list with old lipid names into
                        current nomenclature
  library:              creating a spectral library in *.blib format
                        from a lipid list
  random:               generating a random lipid name (not
                        necessarily reasonable in terms of chemistry)
  agentmode:            secret agent mode
```

The options ‘transitionlist’, ‘translate’ and ‘library’ are the most common modes.

Creating a transition list

A transition list contains all necessary information about the precursor name / mass / charge / adduct in combination with its fragment name / mass / charge / adduct / (collision energy). To create a transition list, a lipid list is necessary. An 'example-lipid-list.csv' can be found within the LipidCreator root directory in the folder 'data\examples'. The 'transitionlist' mode has the following options:

```
> .\LipidCreator.exe transitionlist help
Creating a transition list from a lipid list

usage: LipidCreator.exe transitionlist input_csv output_csv [opts
[opts ...]]
  opts are:
    -p 0:              Compute no precursor transitions
    -p 1:              Compute only precursor transitions
    -p 2:              Compute with precursor transitions
    -h 0:              Compute no heavy labeled isotopes
    -h 1:              Compute only heavy labeled isotopes
    -h 2:              Compute with heavy labeled isotopes
    -s:                Split in positive and negative list
    -x:                Developer or Xpert mode
    -l:                Create LipidCreator project file instead
                       of transition list
    -d:                Delete replicate transitions (equal
                       precursor and fragment mass)
    -c instrument mode: Compute with optimal collision energy (not
                       available for all lipid classes)
[illegible]
```

Optionally, several options (e.g. precursor or heavy labeled fragments) can be set or collision energy optimization can be enabled. A command for creating a transition list without replicates and heavy labeled isotope fragments (if by default included in LipidCreator) looks like this:

```
> .\LipidCreator.exe transitionlist data\examples\example-lipid-
list.csv output-transition-list.csv -h 0 -d
```

The resulting transition list file can be further imported in follow-up analysis tools such as Skyline.

Translating lipid names

Several different structural nomenclatures for lipid names exist. Especially, many legacy names exist for the same lipid species. This results in problems when parsing lipid lists in tools which only have one nomenclature implemented. To solve this issue, a lipid name translation engine was developed to recognize old and deprecated lipid names (e.g. as still used by LIPID MAPS) to translate them into lipid species names according to the current nomenclature[23, 24]. To translate lipid lists into the current nomenclature, the user has to type in:

```
> .\LipidCreator.exe translate data\examples\old-lipid-name-
list.csv new-nomenclature-lipid-list.csv
```

The old lipid names will be preserved in the new list. The recognition system is far from being complete, so we encourage all users who encounter issues with the translator to send us[1] their lipid lists. Such feedback allows us to extend the translation engine further.

Creating a spectral library

It is possible to create an *in silico* spectral library in *.blib format with the command line interface. To estimate the relative fragment abundances, true MS^2 measurements were used to train parameterized statistical models. Although we measured different lipid species with different adducts on different platforms, we do not guarantee correctness of the estimated spectra or generalizability to arbitrary platforms. To create a spectral library for lipids, a lipid list (*.csv) or LipidCreator project file (*.lcXML) as well as the PSI-MS controlled vocabulary term of the measurement platform is required. For example, the user has to type:

```
> .\LipidCreator.exe library data/examples/example-lipid-list.csv
output-library.blib MS:1002523
```

Here, 'MS:1002523' is the CV term for the instrument 'Thermo Scientific Q Exactive HF'. The created spectral library can be now easily imported in follow-up analysis tools such as Skyline[25].

[1] [https://lifs.isas.de/]

Support for additional platforms for CE Optimization

Due to slight differences in the way that vendor data is transformed, and specifically, how activation/collision dissociation details are reported in ‘mzML’ files, an adaptation of the existing codebase may be necessary.

Please refer to the flipR projects and their documentation at [https://github.com/lifs-tools/flipr-trainer] if you want to add data for a specific new platform or if you wish to use your own reference data for an existing platform. If you need assistance, please contact us at [https://lifs.isas.de/support] or via Github.

The following sections describe the tables that are required for a) LipidCreator to read/use a custom model, and b) the tables used by flipR as input and those produced by it for import in LipidCreator.

MS Instrument Table

The comma-separated MS instrument table for LipidCreator (data\ms-instruments.csv) contains PSI-MS controlled vocabulary terms for instrument model to identify individual MS platforms on each row. The following columns are required in this file:

- CV_term: the PSI-MS CV term id identifying the instrument.
- model: the PSI-MS CV term’s description, e.g. the name of the instrument (term MS:1002523, ‘Q Exactive HF’).
- min_CE: the minimum collision energy covered by the instrument. Collision energies calculated by LipidCreator can not be lower than this threshold. The model calculated by flipR may exceed the range set by min_CE and max_CE.
- max_CE: the maximum collision energy covered by the instrument. Collision energies calculated by LipidCreator can not be higher than this threshold. The model calculated by flipR may exceed the range set by min_CE and max_CE.
- x axis label: the label for the collision energy axis, e.g. ‘Collision Energy [eV]’ or ‘Relative Collision Energy’.
- modes: the modes to enable for the instrument platform, e.g. ‘PRM’ for PRM-only mode.

Example (‘…’ represent skipped lines in the file):

```
CV_term,model,min_CE,max_CE,x axis label,modes
...
MS:1002523,Thermo Scientific Q Exactive HF,10,60,Normalized Collision Energy,PRM
```

To feed data into the fragment intensity prediction (FIP) pipeline, MS/MS data needs to be available in '*.mzML' format. We recommend msConvert [http://proteowizard.sourceforge.net/download.html] for the conversion from native vendor format into '*.mzML'. msConvert is part of the Proteowizard suite of tools. Please make sure to install the 64bit version.

msConvert may not always report the most precise term for the instrument, but rather a more generic term that identifies the instrument family.

A list of the available instruments is available via the OntologyLookupService[2].

Transition Table

The transition table used for the CE model calculation can be generated using LipidCreator, similarly to how a transition list is created for Skyline. However, LipidCreator uses specific internal IDs for heavy labeled species, which requires the "developer" mode to create modified transition lists that allow a reimport of the parameter files after the parameter estimation step.

You can start LipidCreator from the command line in developer mode as follows:

```
LipidCreator.exe dev
```

This will ensure that precursor names for heavy-labeled instances are written with a specific placeholder, e.g. "{d8}" for 8 Deuterium atoms replacing 8 hydrogens. This allows the correct mapping of the corresponding model parameters in LipidCreator. Adduct names for heavy labeled instances are also exported unaltered in developer mode, not with the Skyline specific nomenclature, e.g. [M8H2-H]1-, to ensure correct mapping to the originating monoisotopic precursor. Precursor and product masses are correctly calculated for heavy labeled instances. The following columns are required in this file (following the format for Skyline transition lists[3]):

- MoleculeGroup
- PrecursorName
- PrecursorFormula
- PrecursorAdduct
- PrecursorMz
- PrecursorCharge

[2] [https://www.ebi.ac.uk/ols/ontologies/ms/terms?iri=http%3A%2F%2Fpurl.obolibrary.org%2Fobo%2FMS_1000031]

[3] [https://skyline.ms/_webdav/home/software/Skyline/%40files/tutorials/SmallMolecule-3_6.pdf]

- ProductName
- ProductFormula
- ProductAdduct
- ProductMz
- ProductCharge
- Note

Example ('…' represent skipped lines in the file):

```
MoleculeGroup PrecursorName PrecursorFormula     PrecursorAdduct      PrecursorMz
      PrecursorCharge      ProductName   ProductFormula      ProductAdduct ProductMz
      ProductCharge Note
…
PIP2   PIP2 17:0-20:4      C46H83O19P3   [M-H]1-      1031.46686541991    -1
      precursor    C46H83O19P3   [M-H]1-      1031.46686541991    -1
```

Please follow the transition list tutorial for LipidCreator to create a transition list for your target molecules (see Overview of the LipidCreator User Interface and following sections). When you reach the step "LipidsReview", select "Store transition list", choose "tsv files (*.tsv)" as your output file format and select "No" when asked whether to split the output by polarity.

Mapping Table (Transitions to measurements)

This tab-separated table defines the mapping from transitions to actual measurements and acts as the glue between LipidCreator transitions lists and the transition extraction and model training with flipR.

- Instrument: the PSI-MS CV term for the instrument, e.g. 'MS:1002523'.
- MoleculeGroup: the molecule group / class of the molecule, e.g. 'PIP2'.
- PrecursorName: the name of the precursor molecule, e.g. 'PIP2 17:0-20:4'.
- PrecursorAdduct: the precursor adduct, e.g. '[M-H]1-'.
- PPMS: a '|' (bar) separated list of ppms to use for transition / m/z matching, e.g. '5|10'.
- File: the source file for this instance, containing MS2 scans.
- Group: a group identifier to distinguish multiple measurements of the same molecule.

Example ('…' represent skipped lines in the file):

```
Instrument     MoleculeGroup  PrecursorName  PrecursorAdduct       PPMS    File    Group
…
MS:1002523     PIP2    PIP2 17:0-20:4  [M-H]1- 5|10    measurements/QExHF03_NM_0001427.mzML  0001427
```

Feature Table (Transitions applied to mzMLs)

The feature table (*-fip.tsv) is created by the transition extraction step and serves as the main data input for flipR. It holds one m/z feature per row, with the following columns, specifying its provenance, parameters and information that is used by downstream steps to maintain a mapping between input transition list and output parameter file for LipidCreator. The following columns are reported in the output file (some correspond to *.mzML elements / attributes, some come from LipidCreator / Skyline):

- instrument
- localDateTimeCreated
- origin
- scanNumber
- polarity
- basePeakMz
- basePeakIntensity
- totalIonCurrent
- id
- scanDefinition
- msLevel
- isolationWindowTargetMz[0]
- isolationWindowLowerOffset[0]
- isolationWindowUpperOffset[0]
- precursorActivationType
- precursorCollisionEnergy
- precursorCollisionEnergyUnit
- ionInjectionTime[0]
- isolationMzMin[0]
- isolationMzMax[0]
- precursorCharge[0]
- precursorMz[0]
- msFunction

- retentionTime
- spectrumType
- rawTic
- group
- foundMass
- foundMassRange[ppm]
- foundMassLowerBound
- foundMassUpperBound
- foundMassError[ppm]
- foundIntensity
- scanRelativeIntensity
- calculatedMass
- species
- precursorAdduct
- fragment
- adduct

Example (some columns omitted for brevity):

```
instrument	localDateTimeCreated	origin	scanNumber	polarity	basePeakMz	basePeakIntensity	totalIonCurrent	…	isolationWindowTargetMz[0]	isolationWindowLowerOffset[0]	isolationWindowUpperOffset[0]	precursorActivationType	precursorCollisionEnergy	precursorCollisionEnergyUnit	…	rawTic	group	foundMass	foundMassRange[ppm]	foundMassLowerBound	foundMassUpperBound	foundMassError[ppm]	foundIntensity	scanRelativeIntensity	calculatedMass	species	precursorAdduct	fragment	adduct
MS:1002523	2018-11-21T08:02:11.851	QExHF03_NM_0001279.mzML	1	NEGATIVE	317.2117523	1.8311354e07	2.737138e07	…	317.212188720703	0.25	0.25	HCD	10.0	electronvolt	…	2.6918232E7	0001279	299.2013854980469	5	299.19990399299996	299.20289600700005	-0.04846886780363687	567272.8	0.021073926	299.2014	9-HEPE	[M-H]1-	299.201	[M-H]1-
```

LipidCreator Parameter Table (After model training and selection)

The comma-separated lipid creator parameter file (data\ce-parameters\MS_CVTERMID.csv, e.g. **MS_1002523.csv** for the Thermo Scientific Q Exactive HF) contains collision energy calculation parameters for each lipid class, as reported and concatenated by flipR. There are as many rows for each fragment, as there are parameters. The following columns are required in this file:

- instrument: the PSI-MS CV term id identifying the instrument, e.g. MS:1002523 for Thermo Scientific Q Exactive HF.
- class: the lipid class, e.g. 10-HDoHE, needs to be double quoted, when the name contains a comma.
- adduct: the precursor adduct for this lipid class, e.g. [M-H]1-.
- fragment: the fragment identifier. If no common name is available, use e.g. “m/z 121.0658”. The precursor must be reported as “precursor”.
- ParKey: the model parameter name, currently one of “model”, “meanlog”, “sdlog”, “scale”, and “shift”.
- ParValue: the model parameter values, currently, for “model” only “dlnormPar” is recognized. Other parameters are expected to be reported as double numbers with a “.” as the decimal separator.

Example (‘…’ represent skipped lines in the file):

```
instrument,class,adduct,fragment,ParKey,ParValue
…
MS:1002523,10-HDoHE,[M-H]1-,m/z 121.0658,meanlog,4.10313116901712
MS:1002523,10-HDoHE,[M-H]1-,m/z 121.0658,model,dlnormPar
MS:1002523,10-HDoHE,[M-H]1-,m/z 121.0658,scale,0.140234378276546
MS:1002523,10-HDoHE,[M-H]1-,m/z 121.0658,sdlog,0.512948788298359
MS:1002523,10-HDoHE,[M-H]1-,m/z 121.0658,shift,2.97212321271515
```

Supplementary Note 3

Modeling results for lipid mediators

The following section, describes the principle figures that we provide in the additional Supplementary Data 5 and 6 (Modeling Results for Thermo QExactive-HF and Waters Q-TOF, respectively). The material here furthermore shows overall summaries of the data that help assess the quality of the predicted fragmentation models. We present the details and background of these models in the main manuscript.

Exemplary Results for 10-HDoHE Mediator

The following Figures show side-by-side comparisons of the nonlinear fit results, predictions and quality control results for the Thermo QExactive-HF and Waters Q-TOF platforms for the 10-HDoHE Mediator

(LMFA04000027 [http://www.lipidmaps.org/data/LMSDRecord.php?LMID=LMFA04000027]).

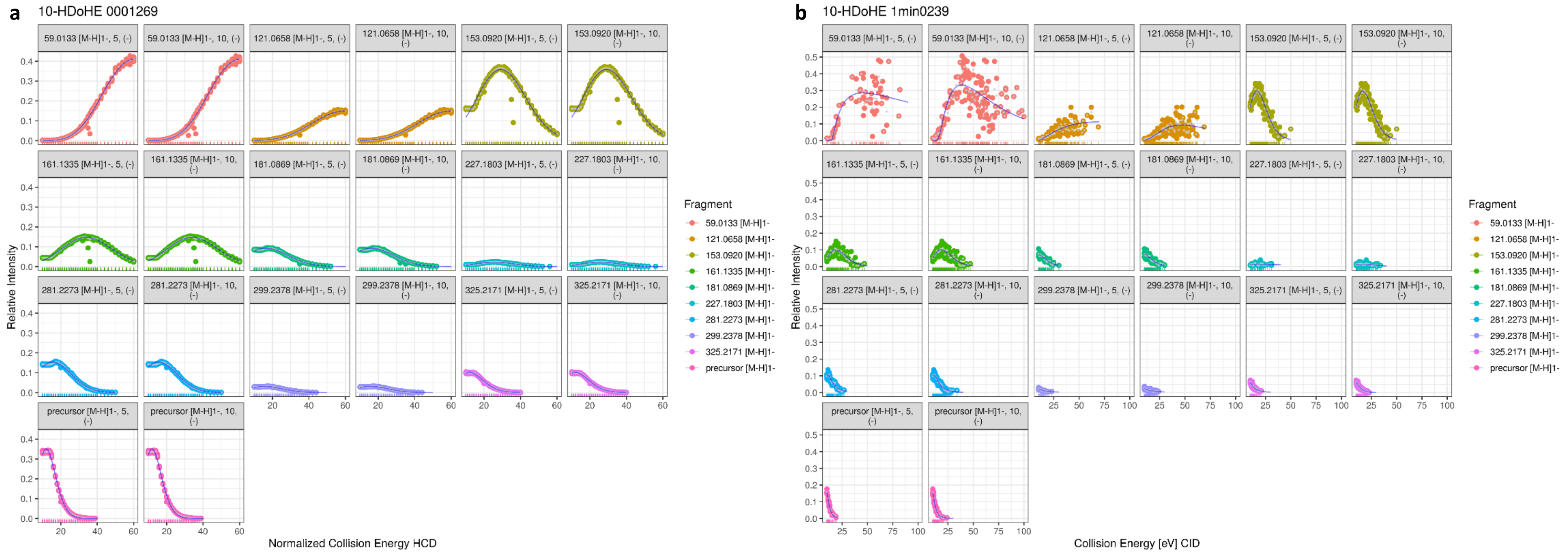

Supplementary Figure 52. Nonlinear fits of 10-HDoHE fragment data. a) Thermo QExactive-HF. b) Agilent Q-TOF. The blue line in each panel represents the non-linear function fitted to the collision energy-dependent relative intensity profile of the particular fragment. Each panel indicates the particular fragment's mass (e.g. 59.0133), adduct (e.g. [M-H]1-), the ppm selection window (e.g. 5 or 10 ppm), and the ionization mode (- or + for negative or positive ionization mode). In each panel, dots colored according to the fragment represent normalized collision energy (arbitrary units or eV) on the horizontal axis versus the measured relative scan intensity on the vertical axis. The bottom of each panel shows the location and spacing of measured data.

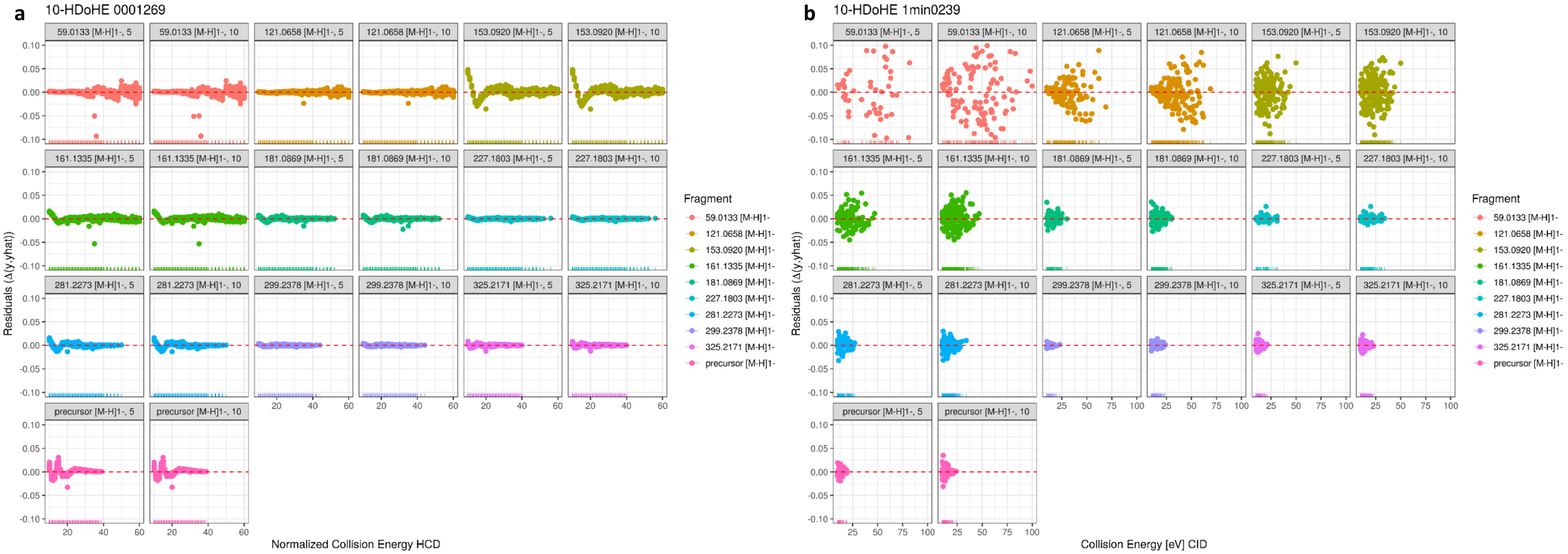

Supplementary Figure 53. Residuals of nonlinear fits of 10-HDoHE fragment data (with 5 and 10 ppm selection window). a) Thermo QExactive-HF. b) Agilent Q-TOF. For the QExactive platform, we used normalized collision energy versus the residuals (the difference between measured 'y' and predicted 'yhat'), while for the Q-TOF platform, we used collision energy in eV, as reported by the vendor software and the subsequent conversion. The bottom of each panel shows the location and spacing of measured data. A limited number of outliers are visible for most of the panels. Ideally, the residuals should be distributed evenly around the zero central axis over the range of the normalized collision energy. Deviations on the left indicate a sub-par fit at low normalized collision energy values, while deviations on the right indicate a sub-par fit at higher normalized collision energies. Supplementary Figure 54 examines the residual behavior in greater detail.

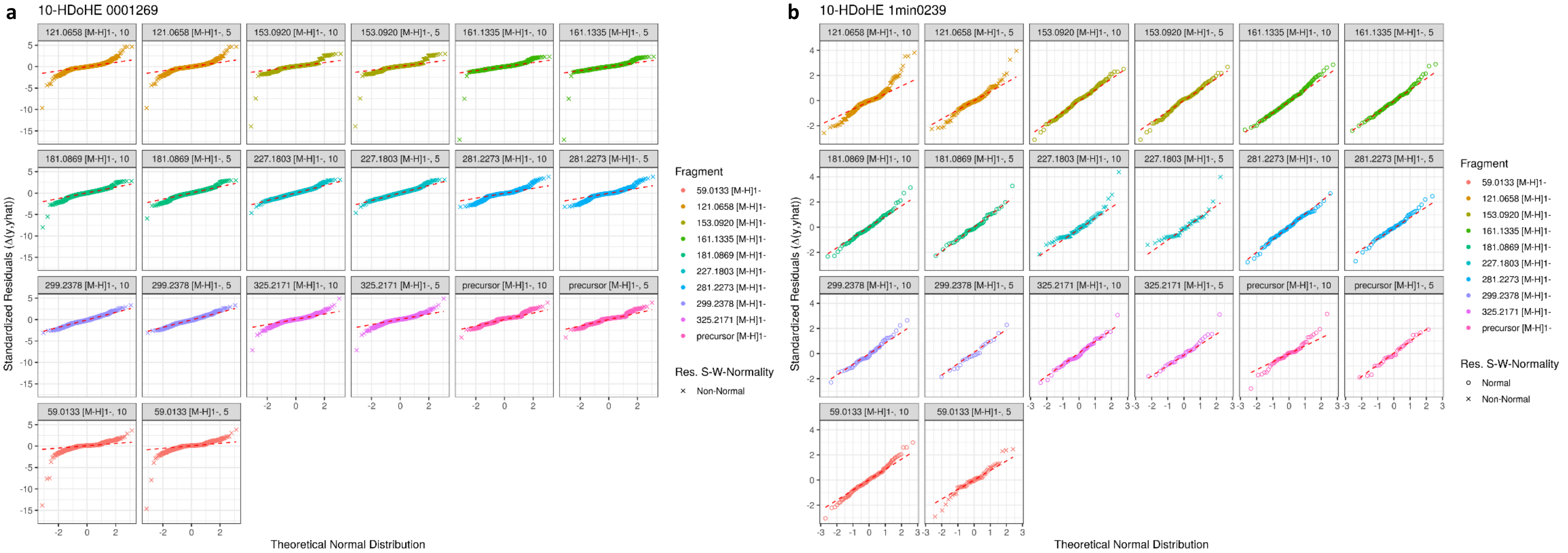

Supplementary Figure 54. QExactive HF Quantile-Quantile plot of nonlinear fit residuals of 10-HDoHE with overlay of Shapiro-Wilk Normality test. a) Thermo QExactive-HF. b) Agilent Q-TOF. ‘x’ symbols indicate a rejection of the normality test hypothesis of the S-W test, given the provided data. ‘o’ symbols indicate acceptance of the S-W test hypothesis. The QExactive HF platform data fit predictions generally show lower residuals than the Q-TOF platform data. However, the residuals of the former, at least for the 10-HDoHE data shown here, do not follow a normal distribution, as indicated by the outcome of the S-W test. This is mainly attributable to the almost constant relative intensities in the low collision energy range. On the Q-TOF platform, most of the residuals pass the S-W test, while the residuals are generally much higher.

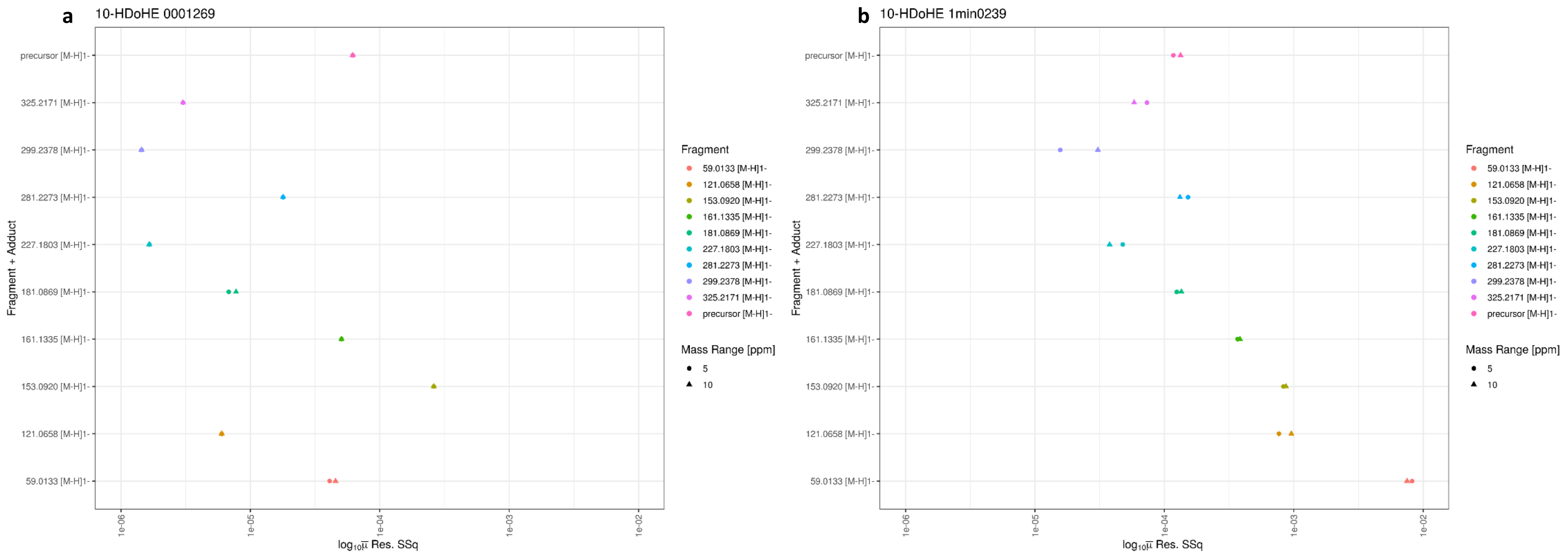

Supplementary Figure 55. QExactive HF Mean of Sum-of-Squared Residuals (MSE) plot of 10-HDoHE (log_{10} scale). a) Thermo QExactive-HF. b) Agilent Q-TOF. The mean of the sum of squared residuals is normalized so that fit statistics for different ppm mass range extraction window parameter values are comparable in terms of the average error they result in. The lower the log10 value of this statistic is, the lower the overall error is for that mass range. This allows a selection of the better mass range extraction window to use for the fit. On the QExactive-HF platform, we found 5 ppm to suffice in almost all cases. For the Q-TOF platform, 5 ppm was often too low to select enough ions to fit a proper regression model, which is why we chose 10 ppm for that platform in most cases.

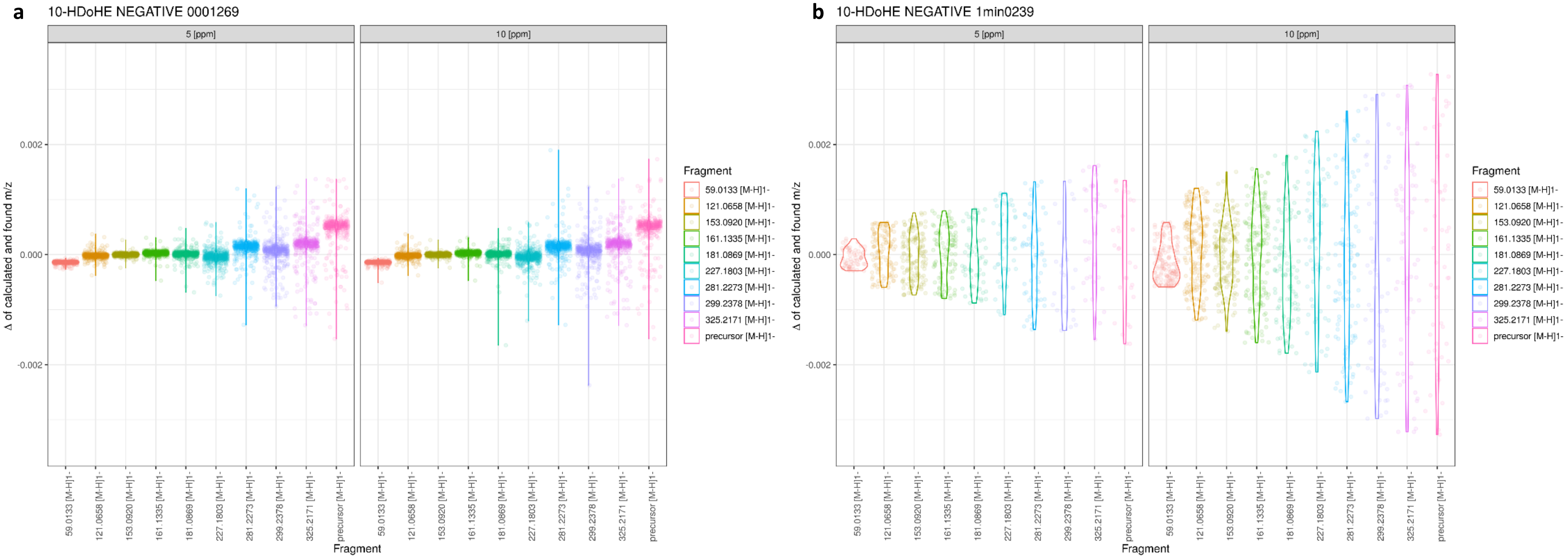

Supplementary Figure 56. QExactive HF delta mass plot of 10-HDoHE for 5 and 10 ppm search window. a) Thermo QExactive-HF. b) Agilent Q-TOF. Due to the high number of samples measured and a low mass deviation, the QExactive-HF data shows a tighter density and overall smaller spread over the mass difference range than the Q-TOF data. The latter shows a more even distribution of the mass error but at the same time also a larger spread over the mass difference range. We attribute these differences to the different mass detector technologies used by the two platforms.

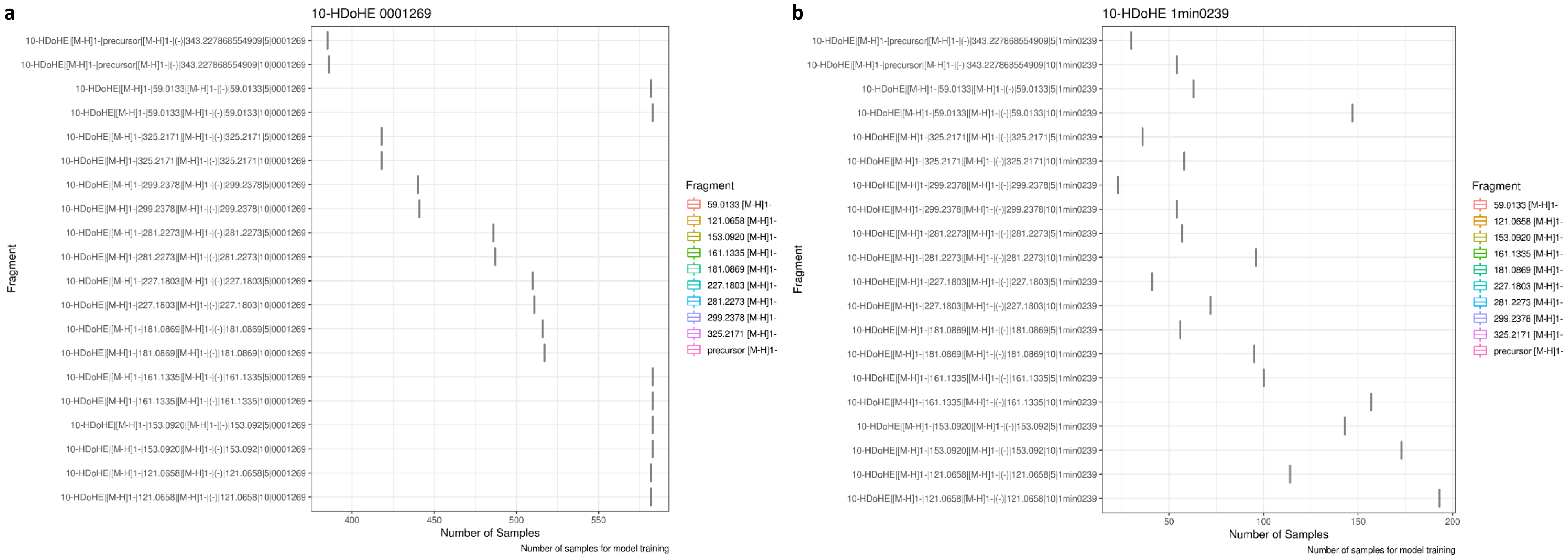

Supplementary Figure 57. Number of (m/z, I) pair samples of 10-HDoHE for model training. a) Thermo QExactive-HF. b) Agilent Q-TOF. For the QExactive-HF platform, an increase of the ppm selection window increases the number of samples only marginally, while marginally worsening the the fit and its residuals (see Supplementary Figures 52-54). For the Q-TOF platform, a selection window of 5 ppm leads to a significantly reduced number of samples and therefor to many sub-optimal fits and consequently high residual values.

Global Comparison of non-linear fit results

The following Figures show overall statistics, such as average residuals and the estimated mean of sum-of-squared residuals for all lipid mediator species that we used for non-linear modeling.

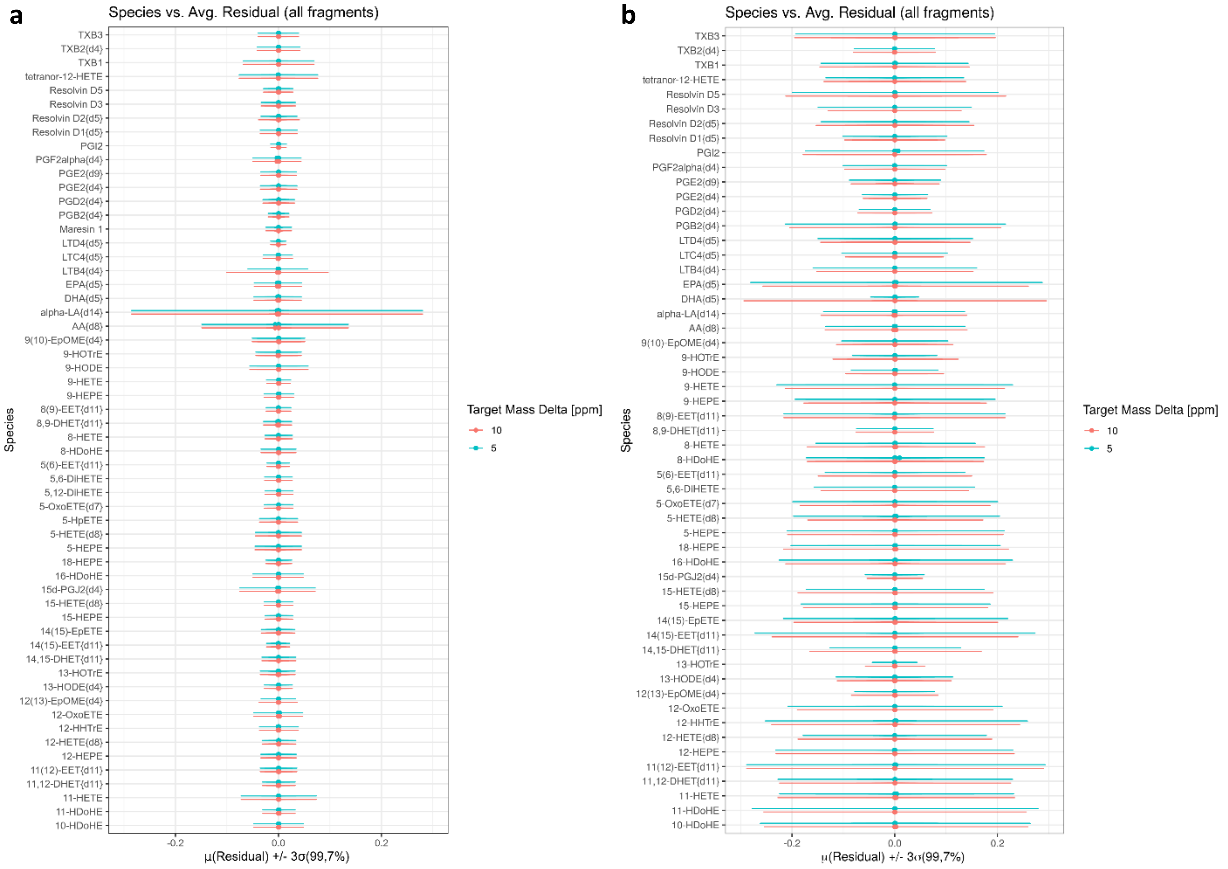

Supplementary Figure 58. Average residual within three sigma for all mediator species. a) Thermo QExactive-HF. b) Agilent Q-TOF. This figure shows the average residual for all mediator species within a range of +/- three standard deviations, covering 99.7% of all residual values. In general, a smaller range around zero indicates a better fit with small deviation. These plots show that for the QExactive-HF platform, the choice between 5 or 10 ppm results in slight advantages towards 5 ppm. It further allows to spot species that have either suboptimal fits, insufficient data, or both, as they are immediately visible through larger residual deviation. For the Q-TOF platform, the plot shows a slight advantage of the 10 ppm window, but not for all species.

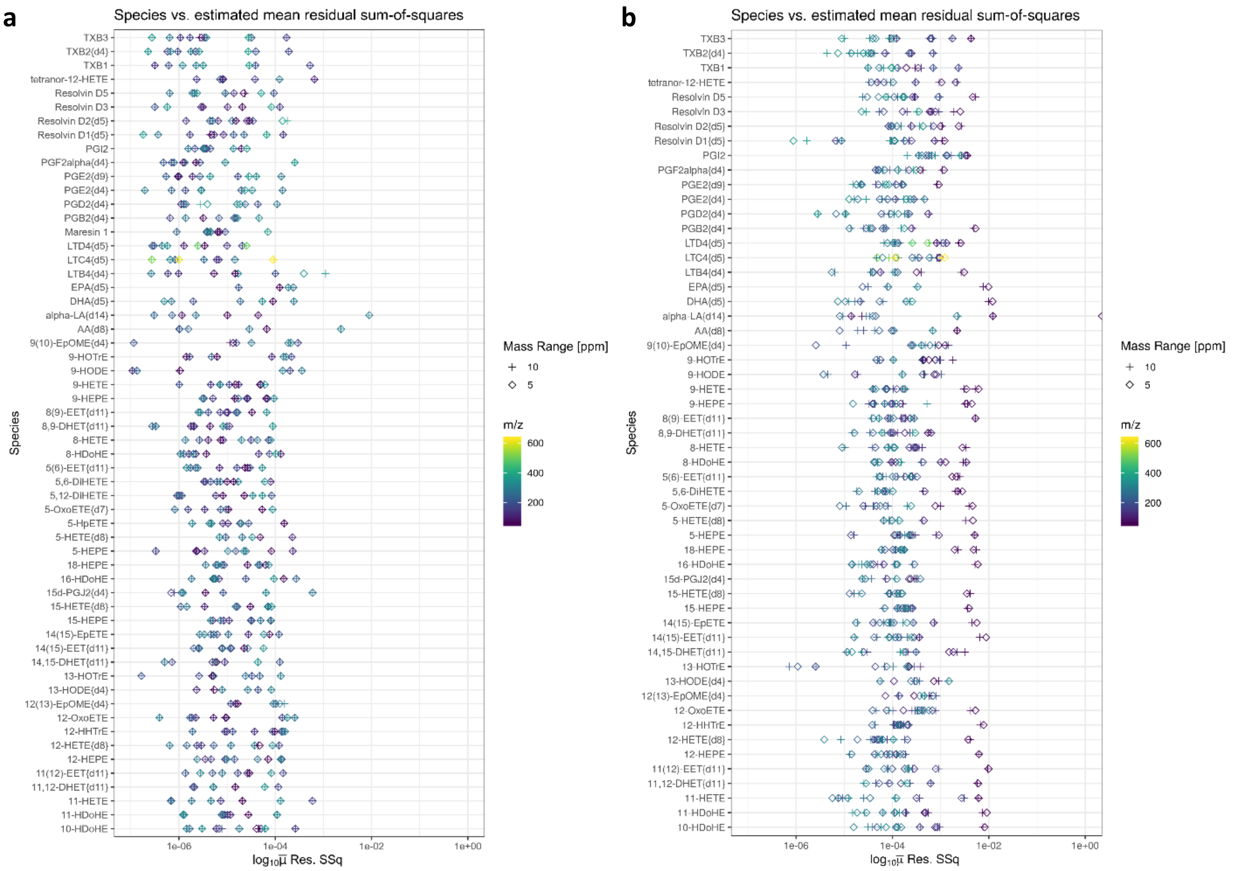

Supplementary Figure 59. Estimated Mean Residual Sum-of-Squares error (MSE) of all mediator species (in log10 scale). a) Thermo QExactive-HF. b) Agilent Q-TOF. This plot shows the log10 of the mean of sum-of-squared residuals for all mediator species fragments. The shape indicates whether the fragment model was calculated based on the 5 ppm or 10 ppm selection window, while the color grading indicates the adduct ion mass of the particular fragment. Thus, if the cross and diamond shapes for a fragment line up, the MSE value is (nearly) identical. These plots show, that there is about a one order of magnitude difference in the MSE values between the QExactive-HF platform on the left (centered around 1e-05) and the Q-TOF platform (centered around 1e-04). In addition, the Q-TOF platform data shows a trend of higher average deviations for lower mass values, which also agrees with the relative intensity profiles and their deviation for lower mass fragments in Supplementary Figure 56.

Supplementary Tables

Supplementary Table 1. Numbers of lipid species from curated or computed sources.

Source		Curated or computed
Literature	LIPID MAPS[1]	43000
	Yang, 2009[25]	36,000
	Brügger, 2014[26]	180,000
	Foster, 2013[27]	36,000,000
	Han, 2016[28]	10,000-1,000,000
LipidCreator	Each FA[2]	1722
	Two FAs containing lipid classes (e.g. PA, PC, PE…)	$\approx \frac{1722^2}{2} = 1.5E+06$
	Three FAs containing lipid classes (TAG)	$\approx \frac{1722^3}{3} = 1.5E+09$
	Four FAs containing lipid classes (CL)	$\approx \frac{1722^4}{4} = 2.2E+12$

[1] Statistics from Dec. 2018, [2] The number of computed fatty acyl (FA) chains in following conditions: chain length :2-30; number of double bonds: 0-6; number of hydroxy group: 0-10. The ether-linked fatty acyl chain and long chain base are not considered. The stereospecific numbering, symmetry, double bond positions of lipids are not considered.

Supplementary Table 2. Literature sources for lipid fragments used in LipidCreator.

Lipid class	***DOI***
Ceramide/sphingolipids[30]	10.1006/abio.2001.5536
Glycerophospholipids/sphingolipids[31]	10.1002/mas.20342
Sphingolipids[32]	10.1021/acs.analchem.7b03576
IPC, MIPC, MIP2C[33]	10.1002/jms.997
Ganglioside[34]	10.1194/jlr.D800038-JLR200
PE (PE P)[35]	10.1016/j.jasms.2004.07.009
PE O/P[36]	10.1016/j.jasms.2007.08.019
PC O/P[37]	10.1007/s13361-014-0908-x
Ganglioside (GM4)[38]	10.1194/jlr.M200010-JLR200
Ganglioside (Hex3Cer)[39]	10.1021/ac702175f
Ganglioside (GM3)[40]	10.1093/glycob/cwp154
Glycerophospholipids (PG)[41]	10.1016/S1044-0305(01)00285-9
Glycerophospholipids[42]	10.1002/rcm.4846
MMPE, DMPE[43]	10.1016/j.bbalip.2011.09.018
TAG[44]	10.1016/j.chroma.2007.10.008
DAG[44]	10.3390/metabo4010098
Mediators[45]	10.1097/01.ftd.0000179845.53213.39

Supplementary Table 3. Calculation of fatty acyls (FA) and long chain base (LCB).

	C	H	O	N
FA	Carbon[1]	2 * Carbon - 1 - 2 * DB[2]	Hydro[3] + 1	0
FAp	Carbon	2 * Carbon + 1 - 2 * DB	Hydro	0
FAa	Carbon	2 * Carbon + 1 - 2 * DB	Hydro	0
LCB	Carbon	2 * Carbon + 2 - 2 * DB	Hydro	1

[1] Carbon: the number of carbon or the fatty acid length

[2] DB: the number of double bonds

[3] Hydro: the number of hydroxyl groups

Supplementary Table 4. Examples of lipid translator usage in LipidCreator.

Before	After[1]	
Name	Name	Adduct
PA 16:0-18:0	PA 16:0-18:0	[M-H]1-[2]
PA(16:0-18:0)		
PA (16:0-18:0)		
PA 16:0/18:0		
PA(16:0/18:0)		
PA (16:0/18:0)		
PA 16:0\18:0		
PA(16:0\18:0)		
PA (16:0\18:0)		
PA 16:0_18:0		
PA(16:0_18:0)		
PA (16:0_18:0)		
PA(+[2]H2) 16:0_18:0[3]		
PA 16:0/18:0[M+NH4]1+		[M+NH4]1+
Cer d18:0/12:0	Cer 18:0;2/12:0	[M+H]1+

[1] This is the form that LipidCreator can recognize.

[2] If the adduct is not specified from the name, the default will be applied. Only the following adduct formats are available: [M+H]1+, [M+NH4]1+, [M+2H]2+, [M-H]1-, [M+HCOO]1-, [M+CH3COO]1-, [M-2H]2-. The adduct format is fixed in order to keep it compatible with Skyline.

[3] All isotope nomenclature cannot be recognized directly from the name. The isotope labels need to be defined in LipidCreator additionally.

Supplementary Table 5. List of targeted lipid species for platelet activation. List of targeted lipid species for platelet activation.

No.	Lipid species	No.	Lipid species
1	ChE 20:4	33	PG 18:0-20:4
2	DAG 18:0-20:4	34	PI 16:0-20:4
3	LPC 16:0	35	PI 17:0-20:4
4	LPC 18:0	36	PI 18:0-20:4
5	LPC 18:1	37	PI 18:1-20:4
6	LPC 18:2	38	PI 20:0-20:4
7	LPC 20:4	39	PI 20:1-20:4
8	LPE 16:0	40	PS 18:0-20:4
9	LPE 18:0	41	PS 18:1-20:4
10	LPE 18:1	42	PS 20:0-20:4
11	LPE 18:2	43	PS 20:1-20:4
12	LPE 20:4	44	11-HETE
13	LPE 20:5	45	12-HEPE
14	LPE 22:4	46	12-HETE
15	LPE 22:5	47	12-HHTrE
16	LPI 18:0	48	12-OxoETE
17	PA 16:0-20:4	49	13-HODE
18	PA 18:0-20:4	50	15-HETE
19	PA 18:1-20:4	51	9-HODE
20	PC 16:0-20:4	52	AA
21	PC 16:1-20:4	53	DHA
22	PC 18:0-20:4	54	EPA
23	PC 18:1-20:4	55	PGD2
24	PC 19:0-20:4	56	PGE2
25	PC 20:0-20:4	57	tetranor-12 HETE
26	PC 20:1-20:4	58	TXB2
27	PC 20:2-20:4		
28	PE 16:0-20:4		
29	PE 16:1-20:4		
30	PE 18:0-20:4		
31	PE 18:1-20:4		
32	PE 20:1-20:4		

Supplementary References

1. Matyash V, Liebisch G, Kurzchalia TV, Shevchenko A, Schwudke D. Lipid extraction by methyl-tert-butyl ether for high-throughput lipidomics. *J Lipid Res* **49**, 1137-1146 (2008).

2. Alshehry ZH, Barlow CK, Weir JM, Zhou Y, McConville MJ, Meikle PJ. An Efficient Single Phase Method for the Extraction of Plasma Lipids. *Metabolites* **5**, 389-403 (2015).

3. Ejsing CS*, et al.* Global analysis of the yeast lipidome by quantitative shotgun mass spectrometry. *PNAS* **106**, 2136-2141 (2009).

4. Bird SS, Marur VR, Sniatynski MJ, Greenberg HK, Kristal BS. Lipidomics profiling by high-resolution LC-MS and high-energy collisional dissociation fragmentation: focus on characterization of mitochondrial cardiolipins and monolysocardiolipins. *Anal Chem* **83**, 940-949 (2011).

5. Bowden JA*, et al.* Harmonizing Lipidomics: NIST Interlaboratory Comparison Exercise for Lipidomics using Standard Reference Material 1950 Metabolites in Frozen Human Plasma. *J Lipid Res* **58**, 2275-2288 (2017).

6. Huynh K*, et al.* High-Throughput Plasma Lipidomics: Detailed Mapping of the Associations with Cardiometabolic Risk Factors. *Cell Chem Biol* **26**, 71-84 e74 (2019).

7. Saw WY*, et al.* Establishing multiple omics baselines for three Southeast Asian populations in the Singapore Integrative Omics Study. *Nat Com* **8**, 653 (2017).

8. Sieber-Ruckstuhl NS*, et al.* Changes in the Canine Plasma Lipidome after Short- and Long-Term Excess Glucocorticoid Exposure. *Sci Rep* **9**, 6015 (2019).

9. Begum H*, et al.* Discovering and validating between-subject variations in plasma lipids in healthy subjects. *Sci Rep* **6**, (2016).

10. Peng B*, et al.* Identification of key lipids critical for platelet activation by comprehensive analysis of the platelet lipidome. *Blood* **132**, e1-e12 (2018).

11. Keat Tham Y*, et al.* Lipidomic Profiles of the Heart and Circulation in Response to Exercise versus Cardiac Pathology: A Resource of Potential Biomarkers and Drug Targets. *Cell Reports* **24**, 2757-2772 (2018).

12. Almeida R, Pauling JK, Sokol E, Hannibal-Bach HK, Ejsing CS. Comprehensive Lipidome Analysis by Shotgun Lipidomics on a Hybrid Quadrupole-Orbitrap-Linear Ion Trap Mass Spectrometer. *J Am Soc Mass Spectrom* **26**, 133-148 (2015).

13. Ellis SR*, et al.* Automated, parallel mass spectrometry imaging and structural identification of lipids. *Nat Methods* **15**, 515-518 (2018).

14. Fraher D, Sanigorski A, Mellett NA, Meikle PJ, Sinclair AJ, Gibert Y. Zebrafish Embryonic Lipidomic Analysis Reveals that the Yolk Cell Is Metabolically Active in Processing Lipid. *Cell Reports* **14**, 1317-1329 (2016).

15. Guan XL*, et al.* Biochemical Membrane Lipidomics during Drosophila Development. *Dev Cell* **24**, 98-111 (2013).

16. Higashi Y, Okazaki Y, Myouga F, K. S, Saito K. Landscape of the lipidome and transcriptome under heat stress in Arabidopsis thaliana. *Sci Rep* **5**, (2015).

17. Herzog R*, et al.* LipidXplorer: a software for consensual cross-platform lipidomics. *PLoS One* **7**, e29851 (2012).

18. Jeucken A, Molenaar MR, van de Lest CHA, Jansen JWA, Helms JB, Brouwers JF. A Comprehensive Functional Characterization of Escherichia coli Lipid Genes. *Cell Rep* **27**, 1597-1606 e1592 (2019).

19. Almeida R*, et al.* Quantitative spatial analysis of the mouse brain lipidome by pressurized liquid extraction surface analysis. *Anal Chem* **87**, 1749-1756 (2015).

20. Berthold MR*, et al.* KNIME-the Konstanz information miner: version 2.0 and beyond. *AcM SIGKDD explorations Newsletter* **11**, 26-31 (2009).

21. Goecks J, Nekrutenko A, Taylor J, Galaxy T. Galaxy: a comprehensive approach for supporting accessible, reproducible, and transparent computational research in the life sciences. *Genome Biol* **11**, R86 (2010).

22. Koster J, Rahmann S. Snakemake--a scalable bioinformatics workflow engine. *Bioinformatics* **28**, 2520-2522 (2012).

23. Liebisch G*, et al.* Shorthand notation for lipid structures derived from mass spectrometry. *J Lipid Res* **54**, 1523-1530 (2013).

24. Pauling JK*, et al.* Proposal for a common nomenclature for fragment ions in mass spectra of lipids. *PLoS One* **12**, e0188394 (2017).

25. MacLean B*, et al.* Skyline: an open source document editor for creating and analyzing targeted proteomics experiments. *Bioinformatics* **26**, 966-968 (2010).

26. Yang K, Cheng H, Gross RW, Han X. Automated lipid identification and quantification by multidimensional mass spectrometry-based shotgun lipidomics. *Anal Chem* **81**, 4356-4368 (2009).

27. Brugger B. Lipidomics: analysis of the lipid composition of cells and subcellular organelles by electrospray ionization mass spectrometry. *Annu Rev Biochem* **83**, 79-98 (2014).

28. Foster JM*, et al.* LipidHome: a database of theoretical lipids optimized for high throughput mass spectrometry lipidomics. *PLoS One* **8**, e61951 (2013).

29. Han X. Lipidomics for studying metabolism. *Nat Rev Endocrinol* **12**, 668-679 (2016).

30. Han X. Characterization and direct quantitation of ceramide molecular species from lipid extracts of biological samples by electrospray ionization tandem mass spectrometry. *Anal Biochem* **302**, 199-212 (2002).

31. Han X, Yang K, Gross RW. Multi-dimensional mass spectrometry-based shotgun lipidomics and novel strategies for lipidomic analyses. *Mass Spec Rev* **31**, 134-178 (2012).

32. Peng B*, et al.* A Comprehensive High-Resolution Targeted Workflow for the Deep Profiling of Sphingolipids. *Anal Chem* **89**, 12480-12487 (2017).

33. Ejsing CS*, et al.* Collision-induced dissociation pathways of yeast sphingolipids and their molecular profiling in total lipid extracts: a study by quadrupole TOF and linear ion trap-orbitrap mass spectrometry. *J Mass Spectrom* **41**, 372-389 (2006).

34. Ikeda K, Shimizu T, Taguchi R. Targeted analysis of ganglioside and sulfatide molecular species by LC/ESI-MS/MS with theoretically expanded multiple reaction monitoring. *J Lipid Res* **49**, 2678-2689 (2008).

35. Zemski Berry KA, Murphy RC. Electrospray ionization tandem mass spectrometry of glycerophosphoethanolamine plasmalogen phospholipids. *J Am Soc Mass Spectrom* **15**, 1499-1508 (2004).

36. Hsu FF, Turk J. Differentiation of 1-O-alk-1'-enyl-2-acyl and 1-O-alkyl-2-acyl glycerophospholipids by multiple-stage linear ion-trap mass spectrometry with electrospray ionization. *J Am Soc Mass Spectrom* **18**, 2065-2073 (2007).

37. Hsu FF, Lodhi IJ, Turk J, Semenkovich CF. Structural distinction of diacyl-, alkylacyl, and alk-1-enylacyl glycerophosphocholines as [M - 15](-) ions by multiple-stage linear ion-trap mass spectrometry with electrospray ionization. *J Am Soc Mass Spectrom* **25**, 1412-1420 (2014).

38. Li YT*, et al.* Association of GM4 ganglioside with the membrane surrounding lipid droplets in shark liver. *J Lipid Res* **43**, 1019-1025 (2002).

39. Kirsch S, Zarei M, Cindric M, Muthing J, Bindila L, Peter-Katalinic J. On-line nano-HPLC/ESI QTOF MS and tandem MS for separation, detection, and structural elucidation of human erythrocytes neutral glycosphingolipid mixture. *Anal Chem* **80**, 4711-4722 (2008).

40. Zarei M, Muthing J, Peter-Katalinic J, Bindila L. Separation and identification of GM1b pathway Neu5Ac- and Neu5Gc gangliosides by on-line nanoHPLC-QToF MS and tandem MS: toward glycolipidomics screening of animal cell lines. *Glycobiology* **20**, 118-126 (2010).

41. Hsu FF, Turk J. Studies on phosphatidylglycerol with triple quadrupole tandem mass spectrometry with electrospray ionization: Fragmentation processes and structural characterization. *J Am Soc Spectrom* **12**, 1036-1043 (2001).

42. Hou W, Zhou H, Bou Khalil M, Seebun D, Bennett SA, Figeys D. Lyso-form fragment ions facilitate the determination of stereospecificity of diacyl glycerophospholipids. *Rapid Commun Mass Spectrom* **25**, 205-217 (2011).

43. Bilgin M*, et al.* Quantitative profiling of PE, MMPE, DMPE, and PC lipid species by multiple precursor ion scanning: a tool for monitoring PE metabolism. *Biochim Biophys Acta* **1811**, 1081-1089 (2011).

44. Cai SS, Short LC, Syage JA, Potvin M, Curtis JM. Liquid chromatography-atmospheric pressure photoionization-mass spectrometry analysis of triacylglycerol lipids--effects of mobile phases on sensitivity. *J Chromatogr A* **1173**, 88-97 (2007).

45. Ogiso H*, et al.* Comparative Analysis of Biological Sphingolipids with Glycerophospholipids and Diacylglycerol by LC-MS/MS. *Metabolites* **4**, 98-114 (2014).

46. Smith CA*, et al.* METLIN: a metabolite mass spectral database. *Ther Drug Monit* **27**, 747-751 (2005).
