# Supplementary material for: LipidCreator workbench to probe the lipidomic landscape
Source: Nat Commun. 2020 Apr 28;11:2057. doi: 10.1038/s41467-020-15960-z (PMC7188904; doi:10.1038/s41467-020-15960-z)
Supplement: Supplementary file 3 — Description of Additional Supplementary Files [file 41467_2020_15960_MOESM3_ESM.docx]

# Description of Additional Supplementary Files

File Name: Supplementary-Data-1.xlsx
Description: Lipid classes/species support status in LipidCreator.

File Name: Supplementary-Data-2.xlsx
Description: The chemical formulas for the head group (HG) building blocks in LipidCreator.

File Name: Supplementary-Data-3.xlsx
Description: Examples of lipid nomenclature in LipidCreator.

File Name: Supplementary-Data-4.xlsx
Description: Lipid fragmentation examples.

File Name: Supplementary-Data-5.pdf
Description: Collision energy-specific relative fragment intensity prediction results for the Thermo Scientific Q Exactive HF platform.

File Name: Supplementary-Data-6.pdf
Description: Collision energy-specific relative fragment intensity prediction results for the Agilent 6545 Q-TOF LC/MS platform.

File Name: Supplementary-Data-7.zip
Description: The scripts underlying Figures 4, 5, 6, 7 and Supplementary Figure 2.

File Name: Supplementary Software 1.zip
Description: Binary release version of LipidCreator 1.1.0.

File Name: Source Data.xlsx.
Description: Source data file containing the data tables behind Figures 4, 5, 6, 7 and Supplementary Figure 2.
