# Supplementary material for: LipidCreator workbench to probe the lipidomic landscape
Source: Nat Commun. 2020 Apr 28;11:2057. doi: 10.1038/s41467-020-15960-z (PMC7188904; doi:10.1038/s41467-020-15960-z)
Supplement: Supplementary file 8 — Supplementary Data 5 [file 41467_2020_15960_MOESM8_ESM.pdf]

Supplementary Data 5 - Modeling Results for the Thermo Q Exactive HF [MS:1002523] platform for:

LipidCreator workbench to probe the lipidomic landscape

Bing Peng[1,2,#], Dominik Kopczynski[1,#], Brian S Pratt[3], Christer S Ejsing[4,5], Bo Burla[6], Martin Hermansson[4,7], Peter Imre Benke[8], Sock Hwee Tan[9,10], Mark Y Chan[9,10,11], Federico Torta[8], Dominik Schwudke[12,13,14], Sven Meckelmann[15], Cristina Coman[1,17], Oliver J Schmitz[15], Brendan MacLean[3], Mailin-Christin Manke[16], Oliver Borst[16], Markus R Wenk[6,8], Nils Hoffmann[1], Robert Ahrends[1,17,*]

[1] Leibniz-Institut für Analytische Wissenschaften – ISAS - e.V., 44139 Dortmund, Germany

[2] Division of Rheumatology, Department of Medicine Solna, Karolinska Institutet, Karolinska University Hospital, SE-171 76 Stockholm, Sweden

[3] University of Washington, Department of Genome Sciences, WA 98195 Seattle, USA

[4] Department of Biochemistry and Molecular Biology, University of Southern Denmark, DK-5230 Odense, Denmark

[5] Cell Biology and Biophysics Unit, European Molecular Biology Laboratory, 69117 Heidelberg, Germany

[6] Singapore Lipidomics Incubator (SLING), Life Science Institute, National University of Singapore, 117456 Singapore, Singapore

[7] Wihuri Research Institute, 00290 Helsinki, Finland

[8] Singapore Lipidomics Incubator (SLING), Department of Biochemistry, Yong Loo Lin School of Medicine, National University of Singapore, 117596 Singapore, Singapore

[9] Department of Medicine, Yong Loo Lin School of Medicine, National University Hospital, 119228 Singapore, Singapore

[10] Cardiovascular Research Institute, National University of Singapore, 117599 Singapore, Singapore

[11] National University Heart Centre, National University Health System, 117599 Singapore, Singapore

[12] Research Center Borstel, Leibniz Lung Center, Borstel, Germany

[13] German Center for Infection Research (DZIF), 38124 Braunschweig, Germany

[14] Airway Research Center North Member of the German Center for Lung Research (DZL), 22927 Großhansdorf, Germany

[15] Institute of Applied Analytical Chemistry, University of Duisburg-Essen, 45141 Essen, Germany

[16] Department of Cardiology and Cardiovascular Medicine, University of Tübingen, 72076 Tübingen, Germany

[17] Department of Analytical Chemistry, University of Vienna, Währinger Strasse 38, 1090 Vienna, Austria

[#] Two authors contributed equally to this work

[*] Corresponding author

Table of Contents

1. Thermo Scientific Q Exactive HF [MS:1002523] 2
1.1. 10-HDoHE [M-H]1- 0001269 2
1.2. 11(12)-EET{d11} [M-H]1- 0001307 5
1.3. 11,12-DHET{d11} [M-H]1- 0000157 8
1.4. 11-HDoHE [M-H]1- 0001275 11
1.5. 11-HETE [M-H]1- 0001289 14
1.6. 12(13)-EpOME{d4} [M-H]1- 0001303 17
1.7. 12-HEPE [M-H]1- 0001281 20
1.8. 12-HETE{d8} [M-H]1- 0001295 23
1.9. 12-HHTrE [M-H]1- 0001347 26
1.10. 12-OxoETE [M-H]1- 0001283 29
1.11. 13-HODE{d4} [M-H]1- 0001277 32
1.12. 13-HOTrE [M-H]1- 0001341 35
1.13. 14(15)-EET{d11} [M-H]1- 0001305 38
1.14. 14(15)-EpETE [M-H]1- 0001285 41
1.15. 14,15-DHET{d11} [M-H]1- 0000155 44
1.16. 15-HEPE [M-H]1- 0000139 47
1.17. 15-HETE{d8} [M-H]1- 0001329 50
1.18. 15d-PGJ2{d4} [M-H]1- 0000149 53
1.19. 16-HDoHE [M-H]1- 0001271 56
1.20. 18-HEPE [M-H]1- 0000131 59
1.21. 5(6)-EET{d11} [M-H]1- 0000161 62
1.22. 5,12-DiHETE [M-H]1- 0001299 65
1.23. 5,6-DiHETE [M-H]1- 0001301 68
1.24. 5-HEPE [M-H]1- 0000133 71
1.25. 5-HETE{d8} [M-H]1- 0000145 74
1.26. 5-HpETE [M-H]1- 0001297 77
1.27. 5-OxoETE{d7} [M-H]1- 0000147 80
1.28. 8(9)-EET{d11} [M-H]1- 0001309 83
1.29. 8,9-DHET{d11} [M-H]1- 0000159 86
1.30. 8-HDoHE [M-H]1- 0001273 89
1.31. 8-HETE [M-H]1- 0001287 92
1.32. 9(10)-EpOME{d4} [M-H]1- 0001355 95
1.33. 9-HEPE [M-H]1- 0001279 98
1.34. 9-HETE [M-H]1- 0001291 101
1.35. 9-HODE [M-H]1- 0001339 104
1.36. 9-HOTrE [M-H]1- 0000137 107
1.37. AA{d8} [M-H]1- 0001337 110
1.38. DHA{d5} [M-H]1- 0001353 113
1.39. EPA{d5} [M-H]1- 0001335 116
1.40. LTB4{d4} [M-H]1- 0001331 119

1.41. LTC4{d5} [M-H]1- 0000163 122
1.42. LTD4{d5} [M-H]1- 0000153 125
1.43. Maresin 1 [M-H]1- 0001319 128
1.44. PGB2{d4} [M-H]1- 0000151 131
1.45. PGD2{d4} [M-H]1- 0001311 134
1.46. PGE2{d4} [M-H]1- 0001313 137
1.47. PGE2{d9} [M-H]1- 0001321 140
1.48. PGF2alpha{d4} [M-H]1- 0000125 143
1.49. PGI2 [M-H]1- 0000127 146
1.50. Resolvin D1{d5} [M-H]1- 0001333 149
1.51. Resolvin D2{d5} [M-H]1- 0000129 152
1.52. Resolvin D3 [M-H]1- 0000141. 155
1.53. Resolvin D5 [M-H]1- 0000143. 158
1.54. TXB1 [M-H]1- 0001325 161
1.55. TXB2{d4} [M-H]1- 0001327. 164
1.56. TXB3 [M-H]1- 0001323 167
1.57. alpha-LA{d14} [M-H]1- 0000135 170
1.58. tetranor-12-HETE [M-H]1- 0001351 173

1. Thermo Scientific Q Exactive HF [MS:1002523]

1.1. 10-HDoHE [M-H]1- 0001269

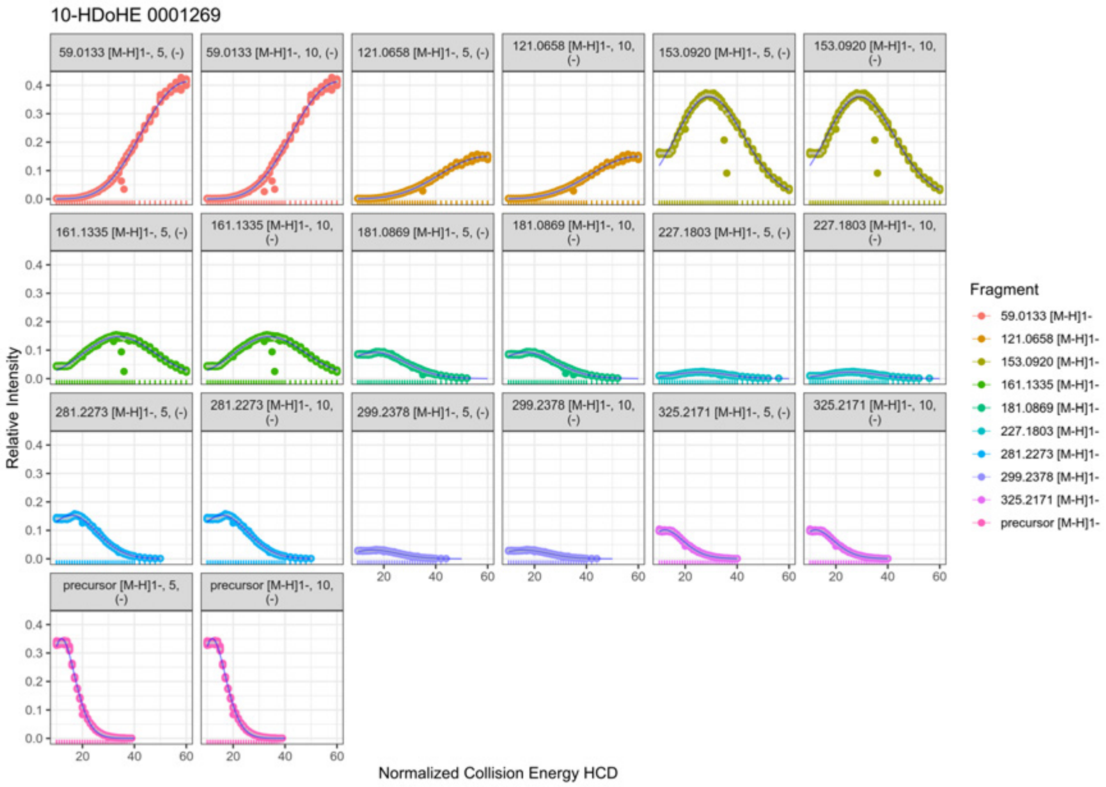

Figure 1. Nonlinear fit

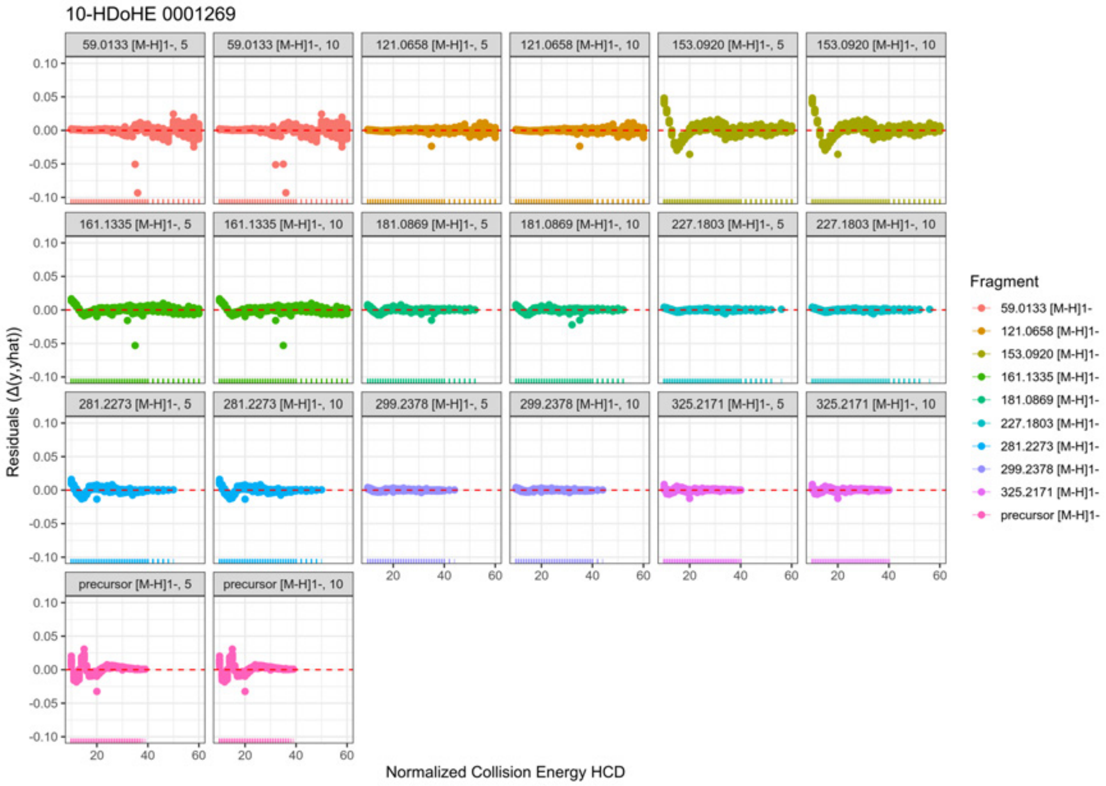

Figure 2. Residuals of nonlinear fit

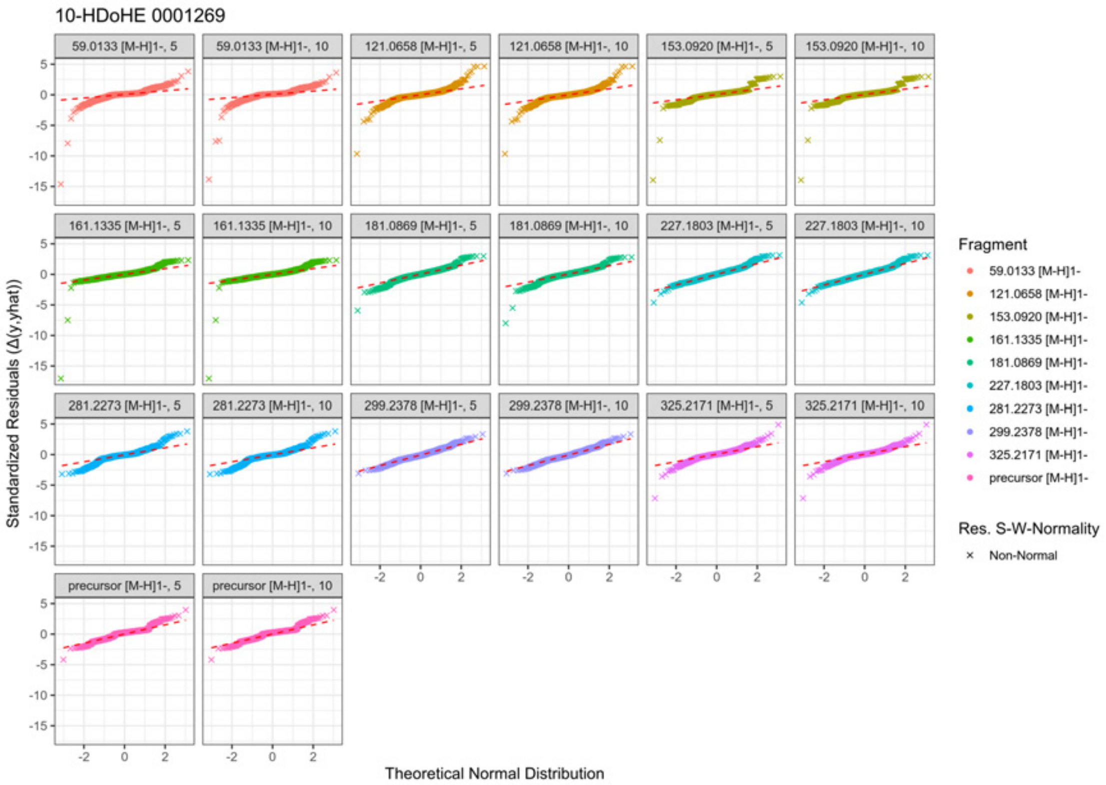

Figure 3. Quantile-quantile plot of residuals

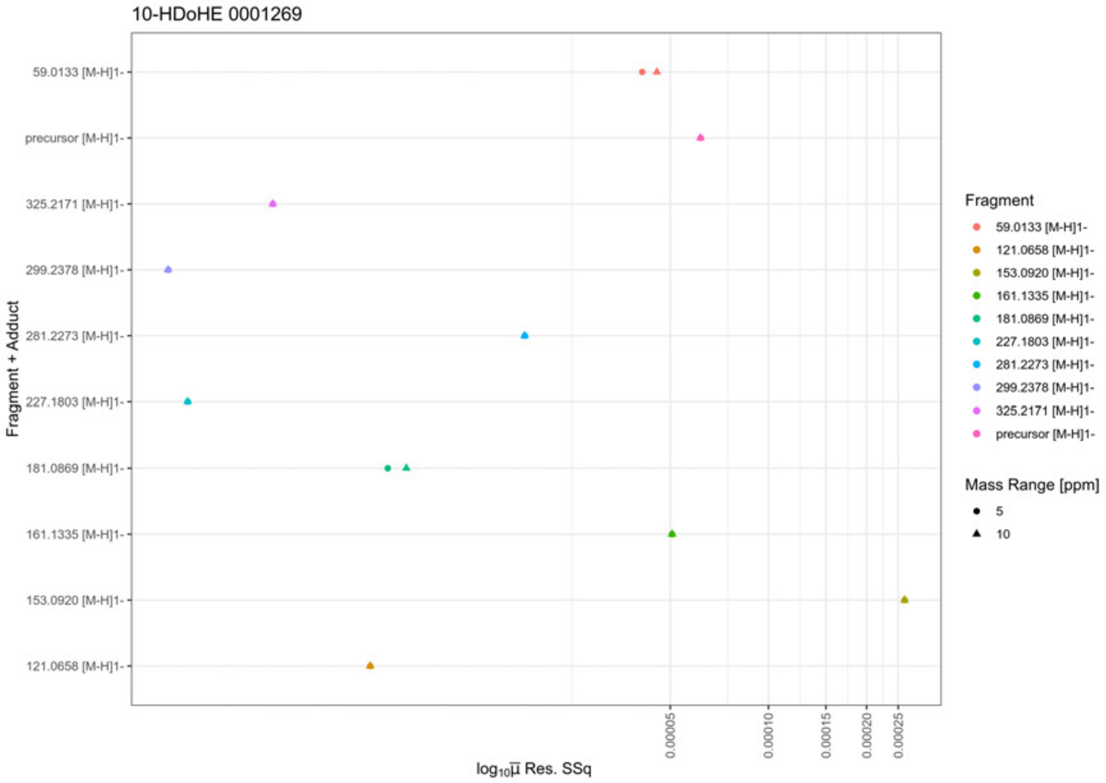

Figure 4. Normalized sum-of-squares of the residuals

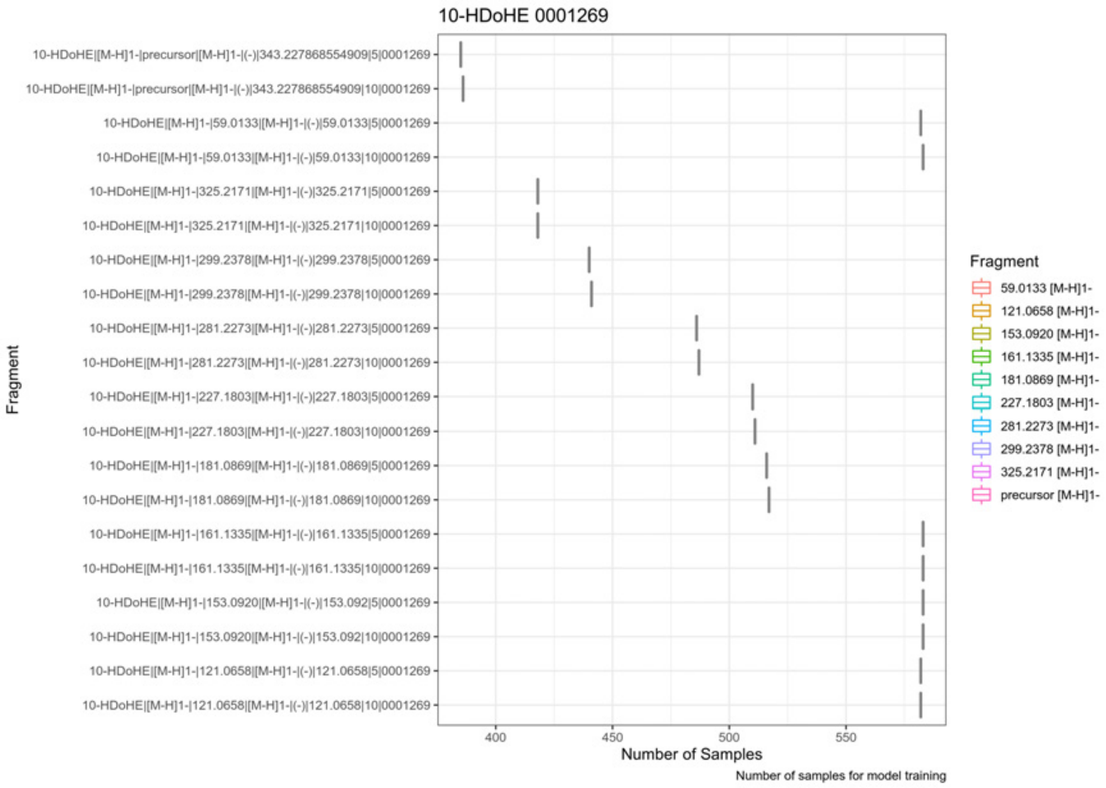

Figure 5. Number of samples used for training per combination Id

1.2. 11(12)-EET{d11} [M-H]1- 0001307

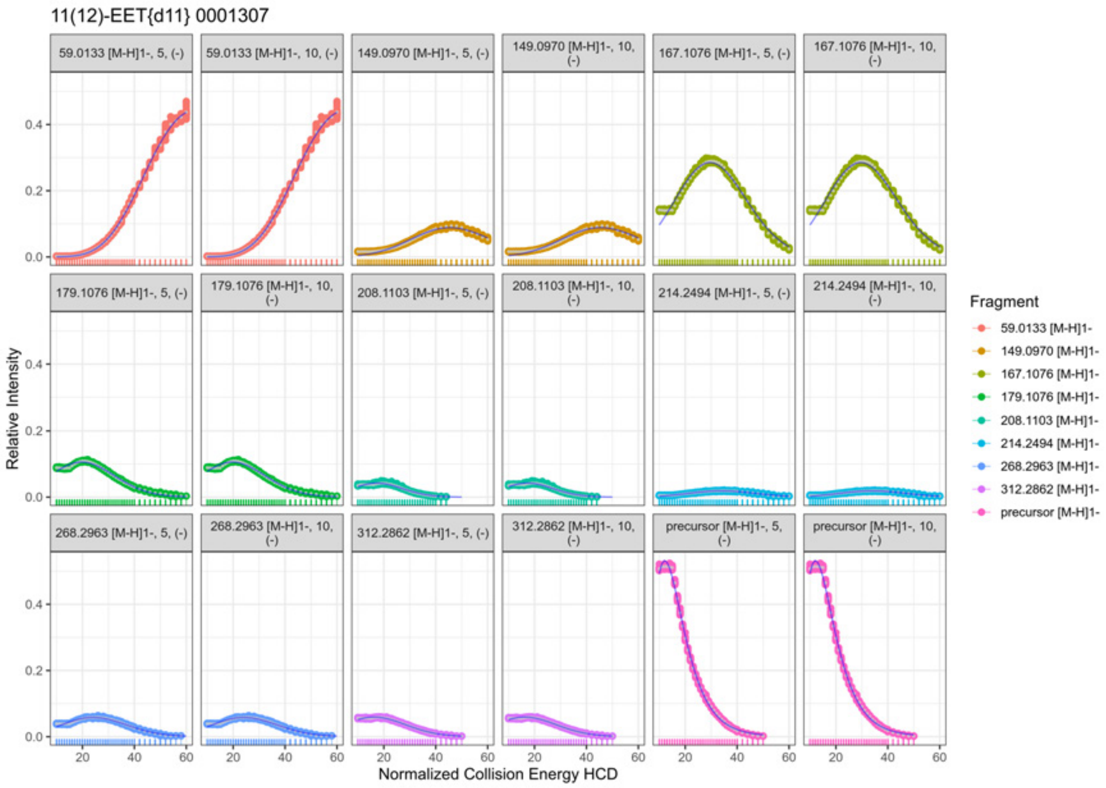

Figure 6. Nonlinear fit

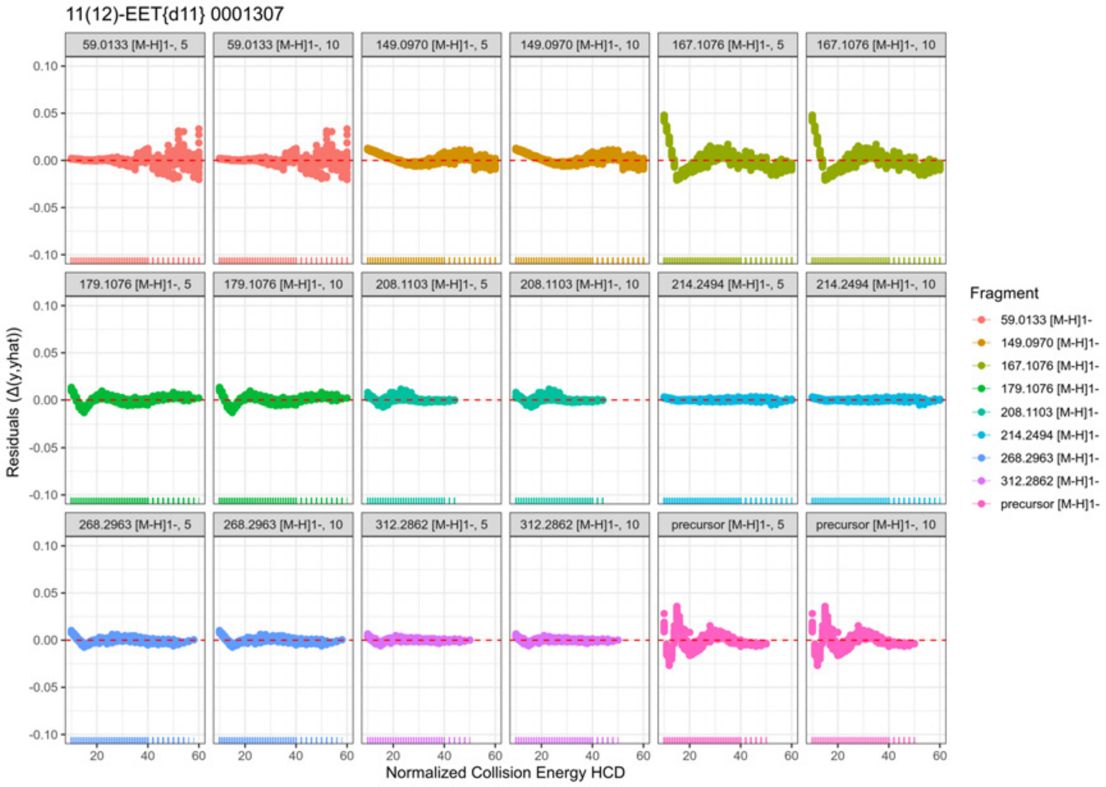

Figure 7. Residuals of nonlinear fit

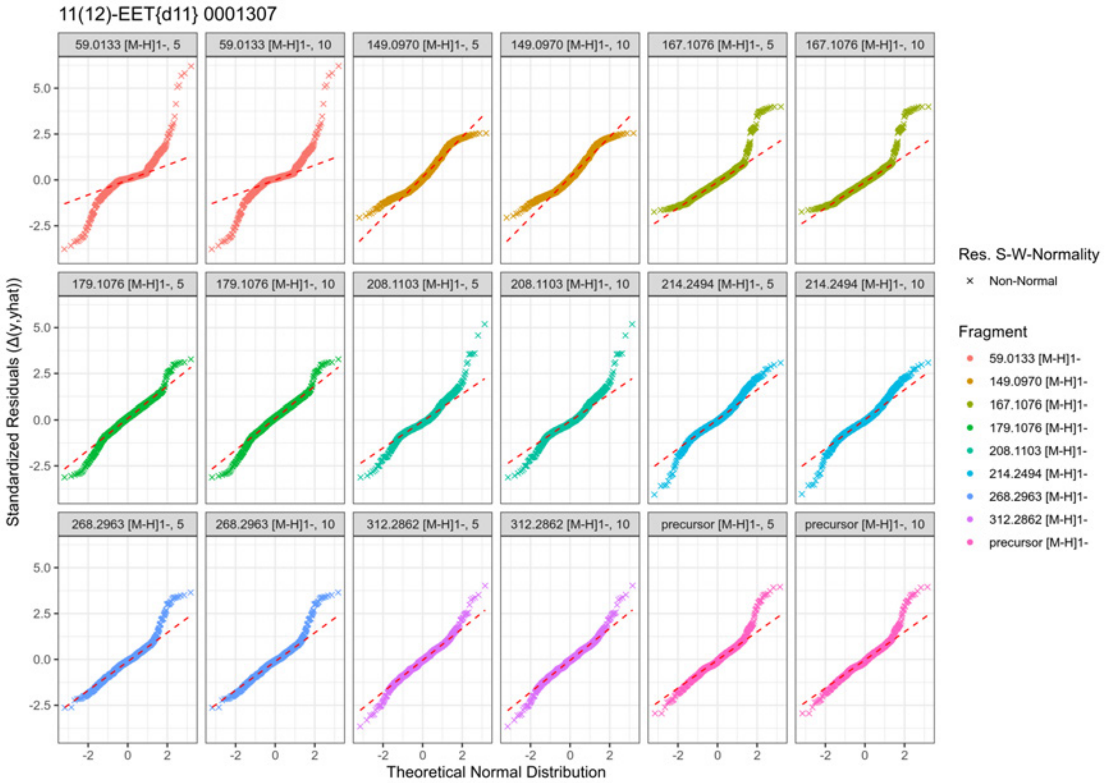

Figure 8. Quantile-quantile plot of residuals

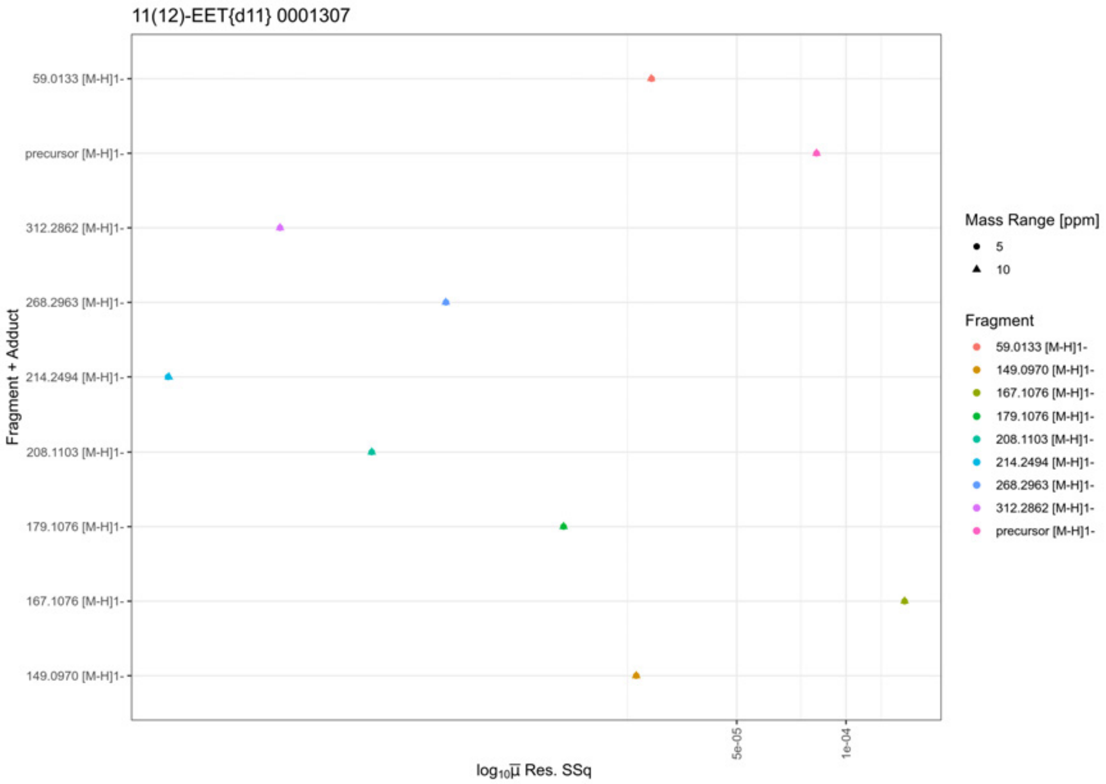

Figure 9. Normalized sum-of-squares of the residuals

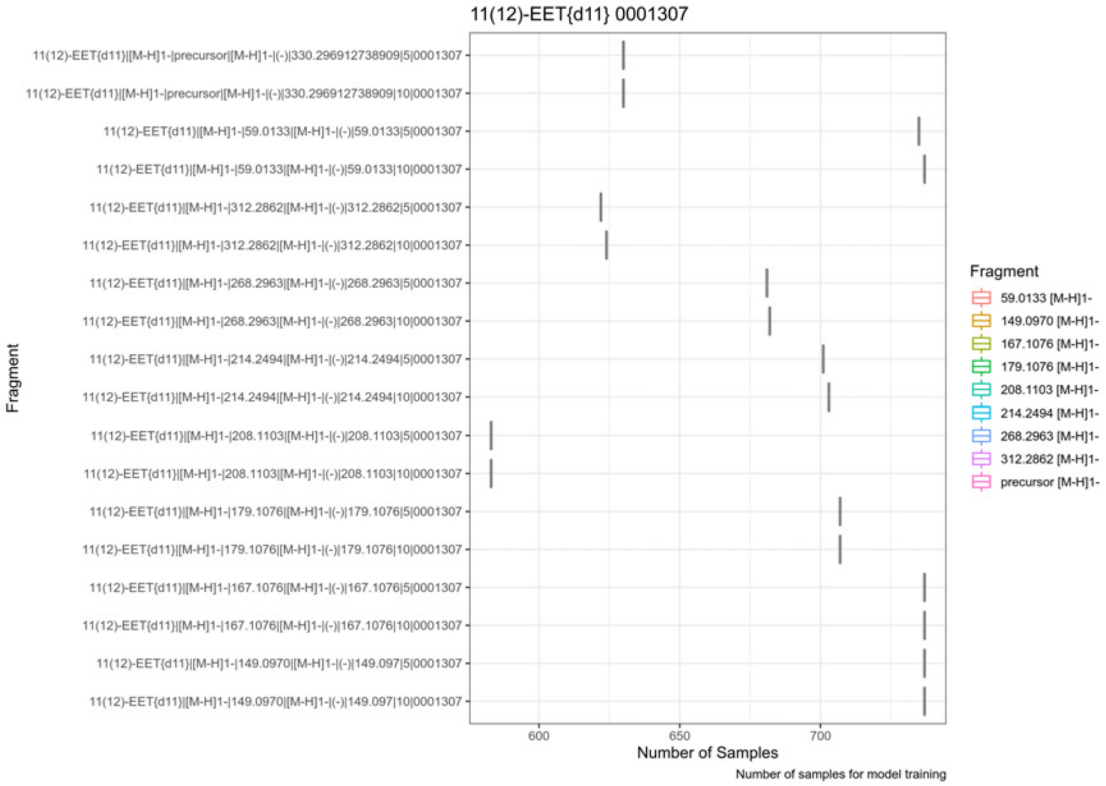

Figure 10. Number of samples used for training per combination Id

1.3. 11,12-DHET{d11} [M-H]1- 0000157

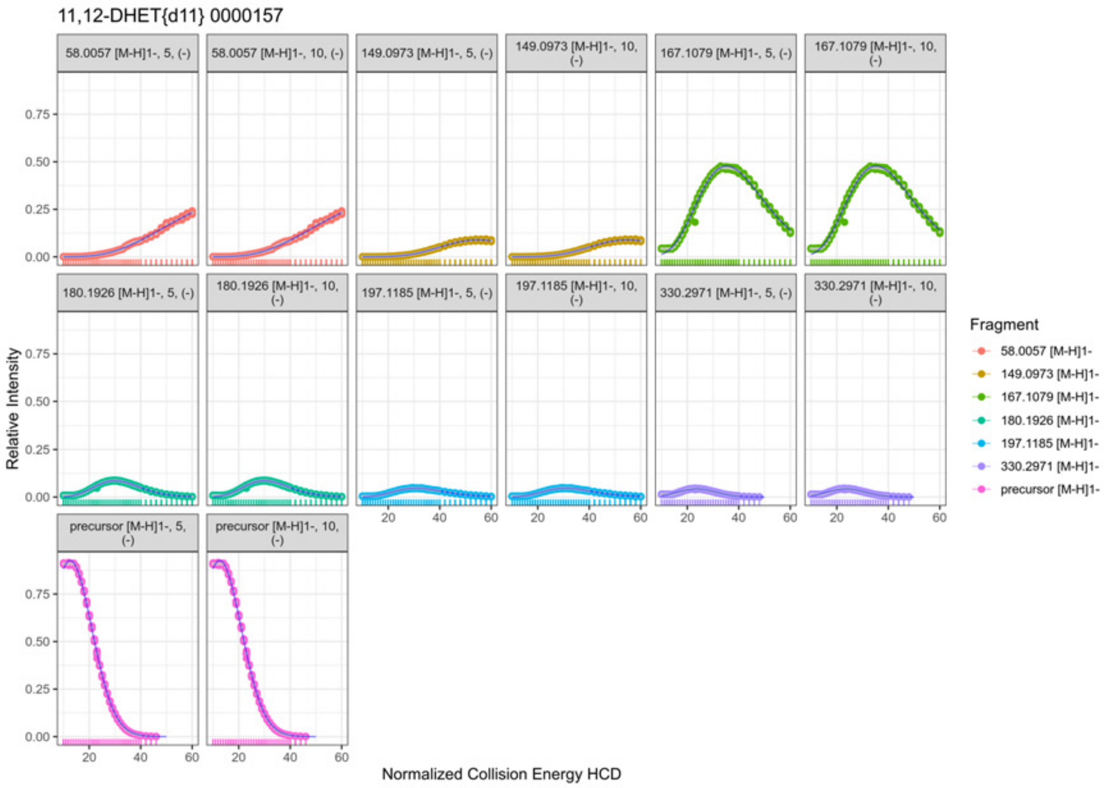

Figure 11. Nonlinear fit

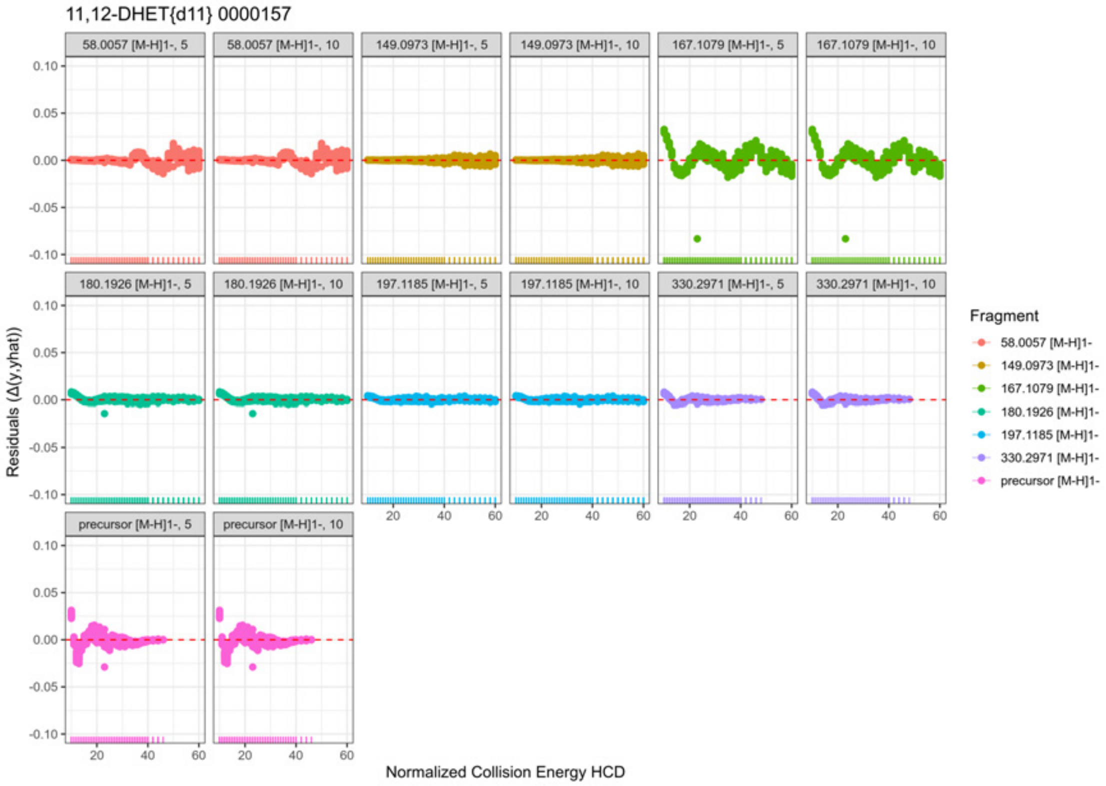

Figure 12. Residuals of nonlinear fit

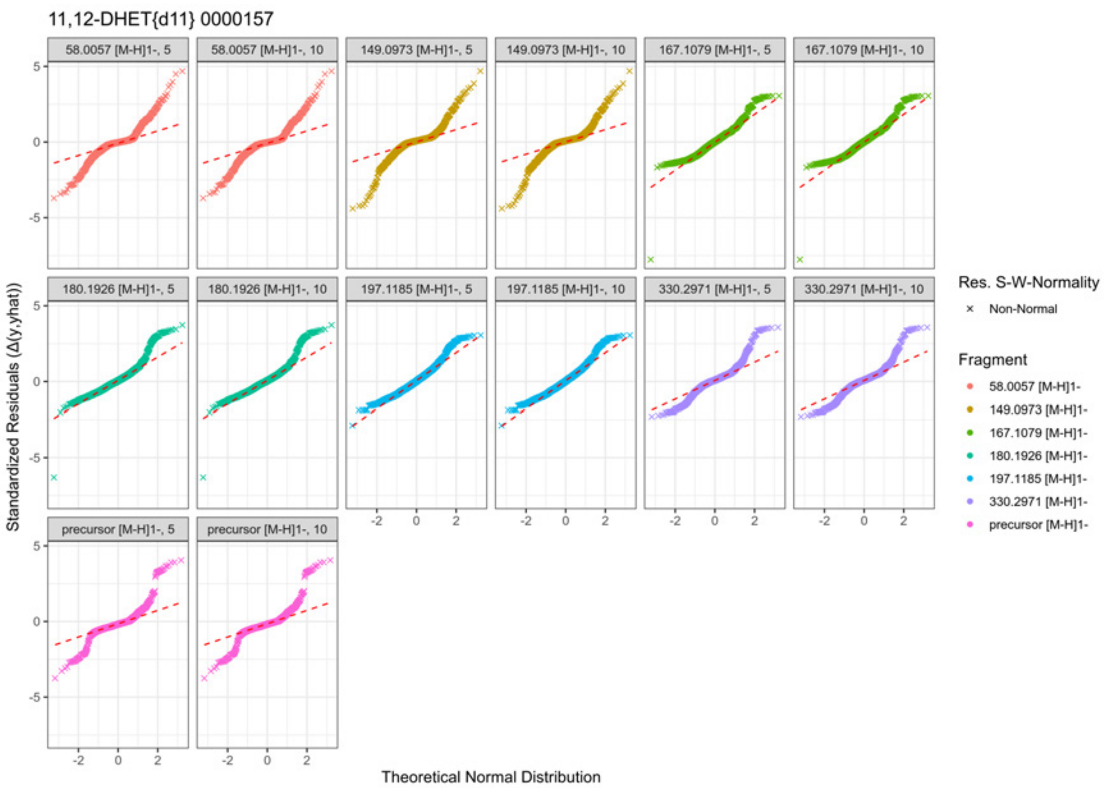

Figure 13. Quantile-quantile plot of residuals

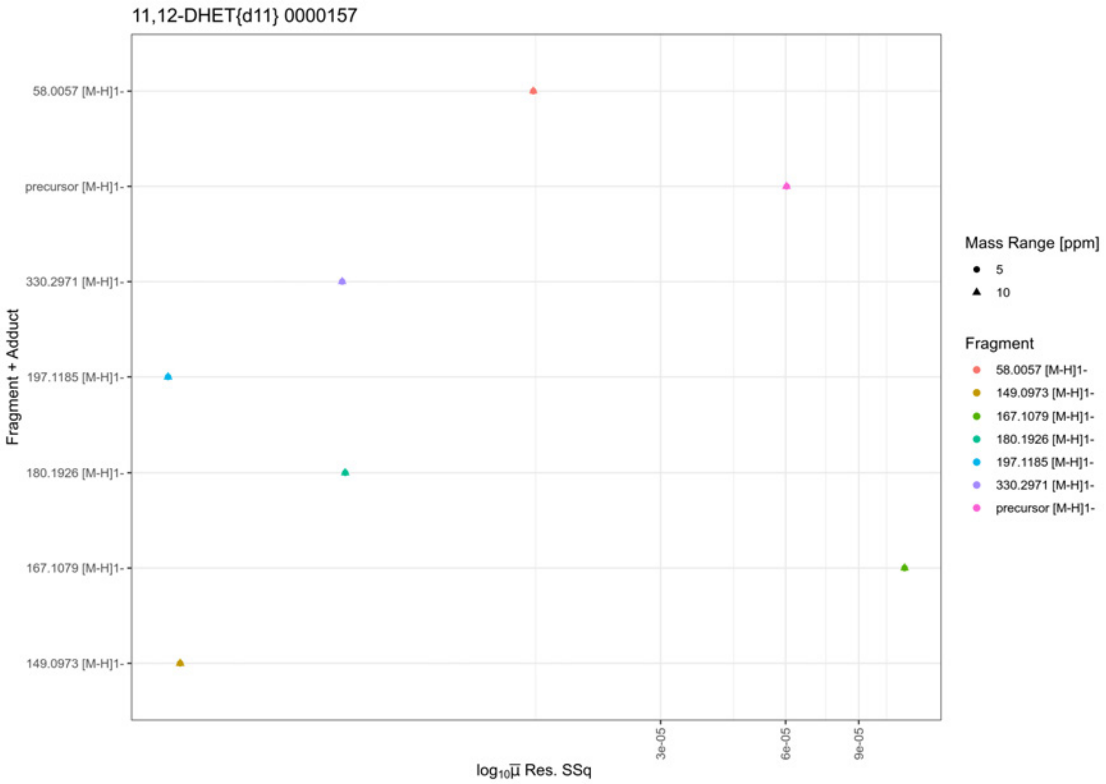

Figure 14. Normalized sum-of-squares of the residuals

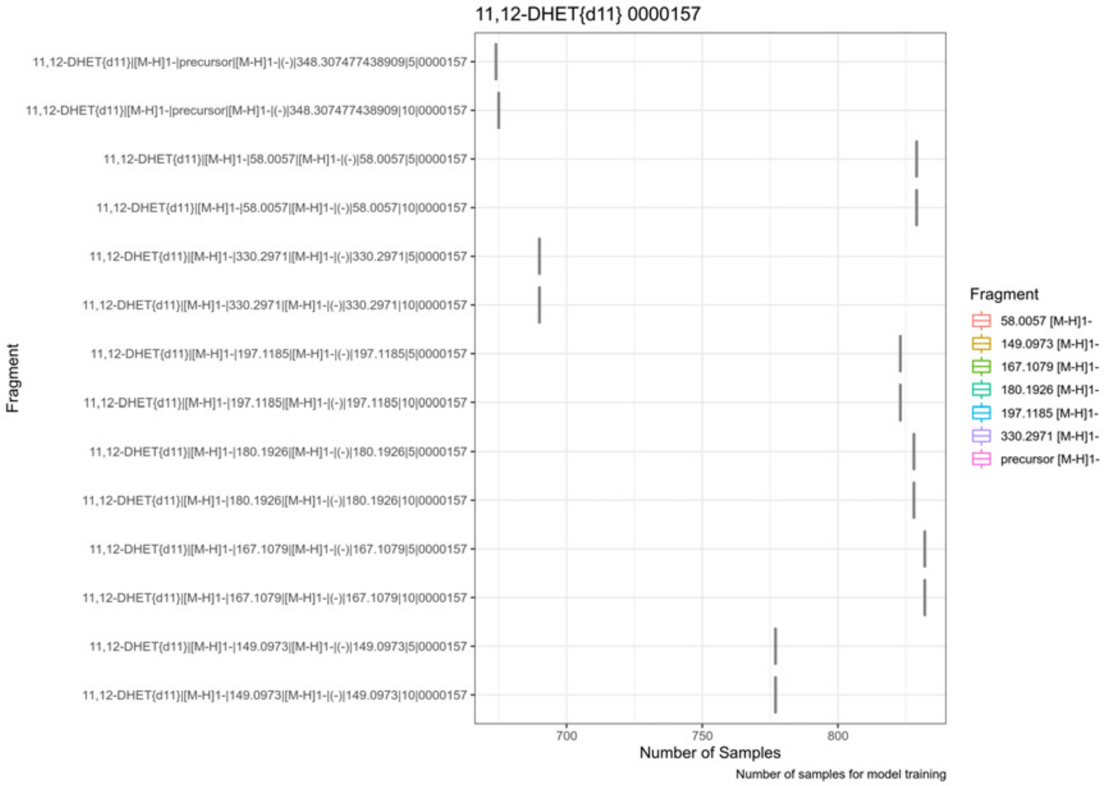

Figure 15. Number of samples used for training per combination Id

1.4. 11-HDoHE [M-H]1- 0001275

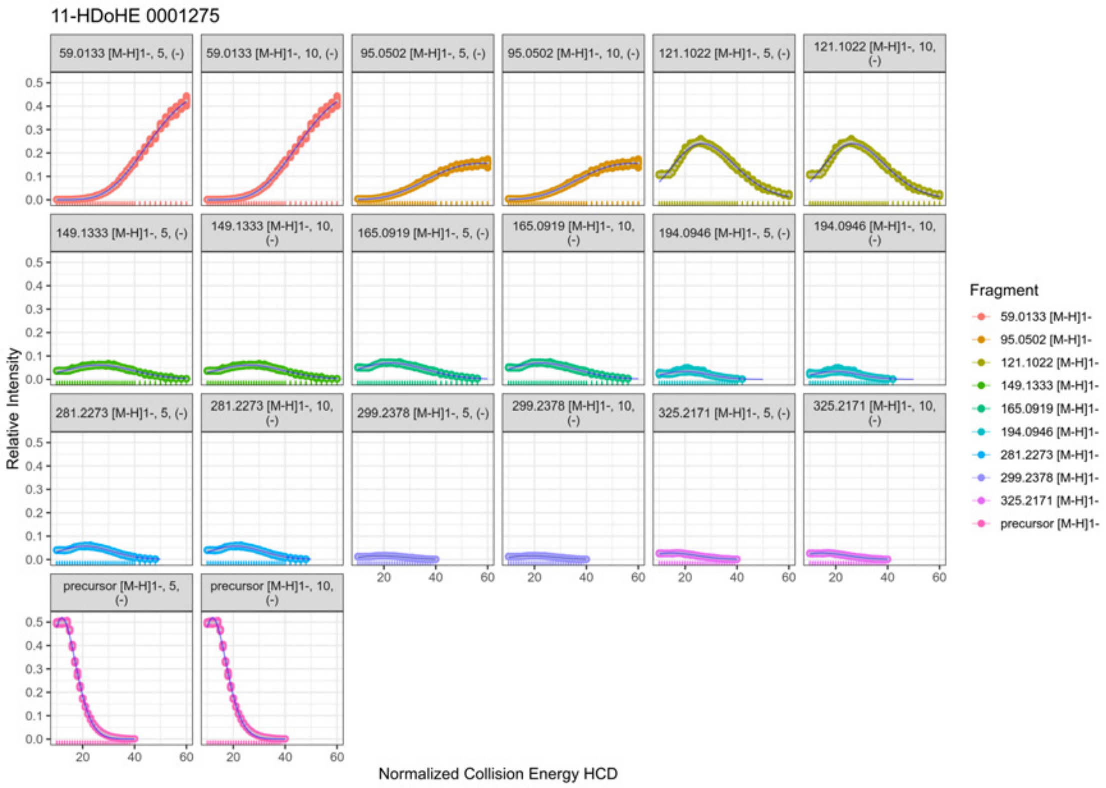

Figure 16. Nonlinear fit

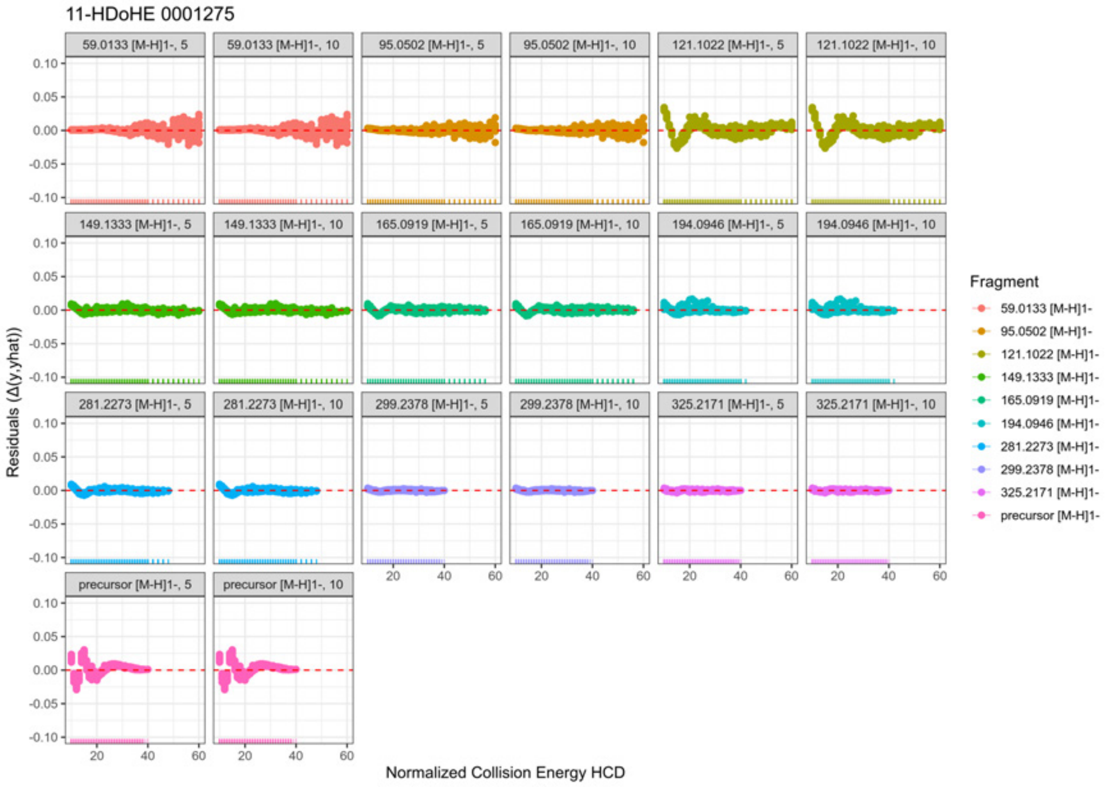

Figure 17. Residuals of nonlinear fit

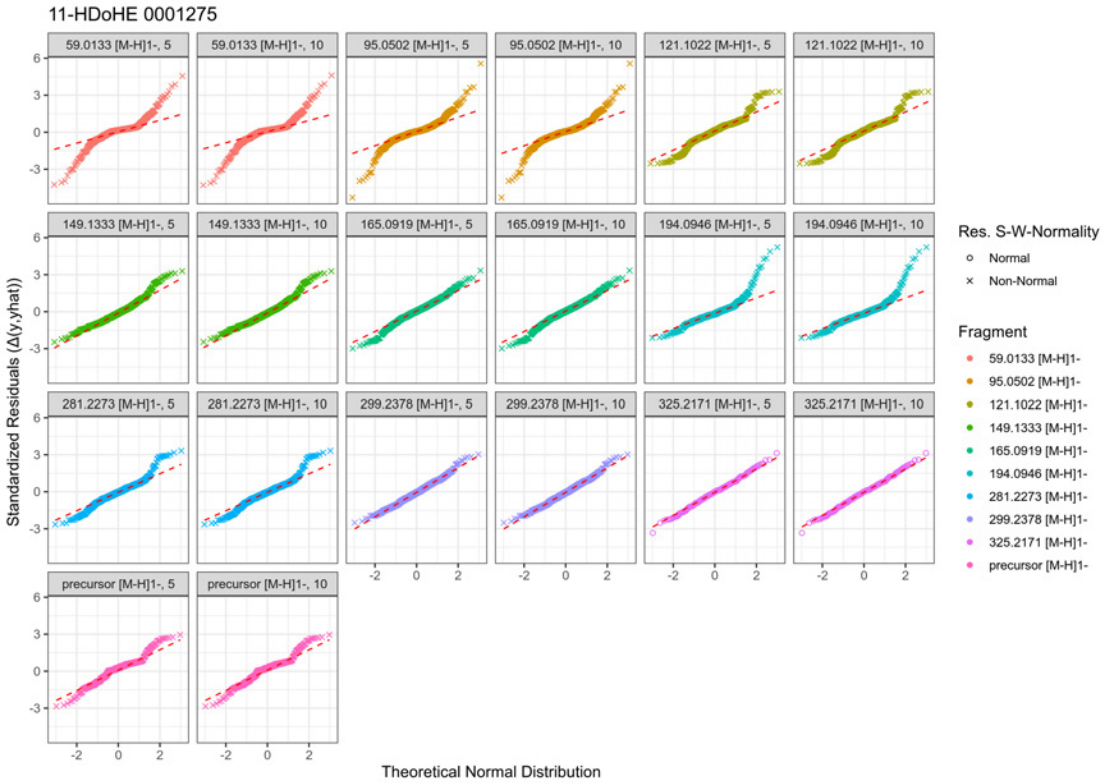

Figure 18. Quantile-quantile plot of residuals

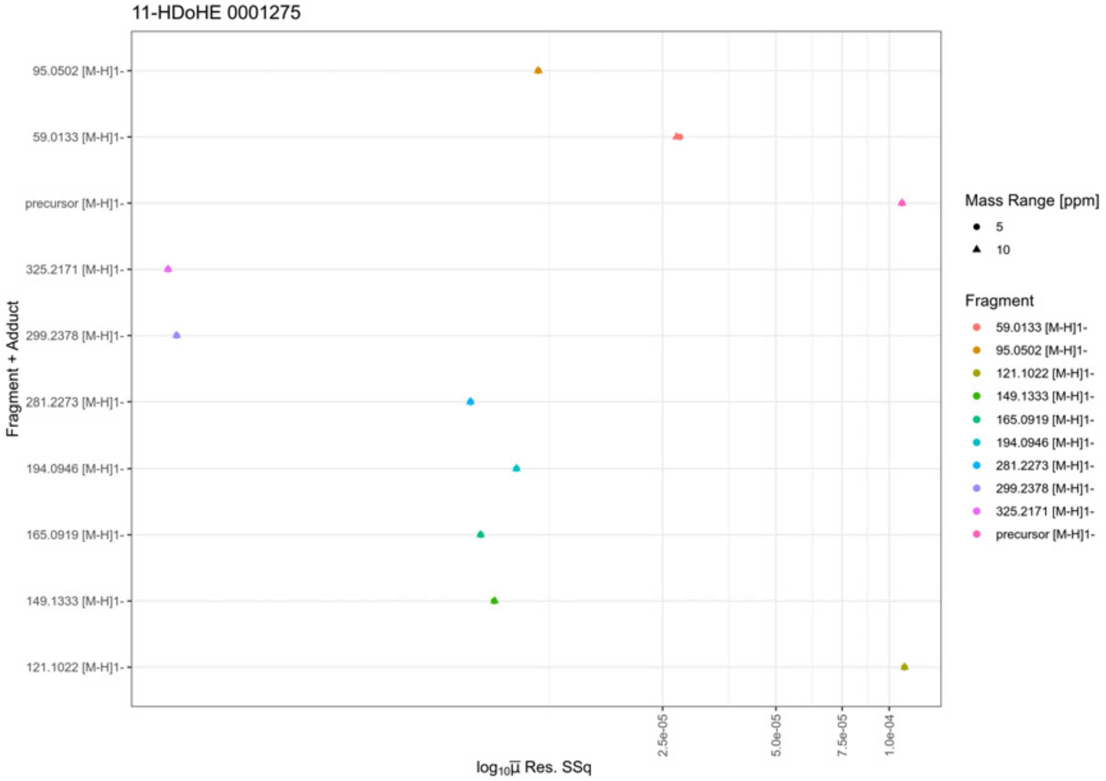

Figure 19. Normalized sum-of-squares of the residuals

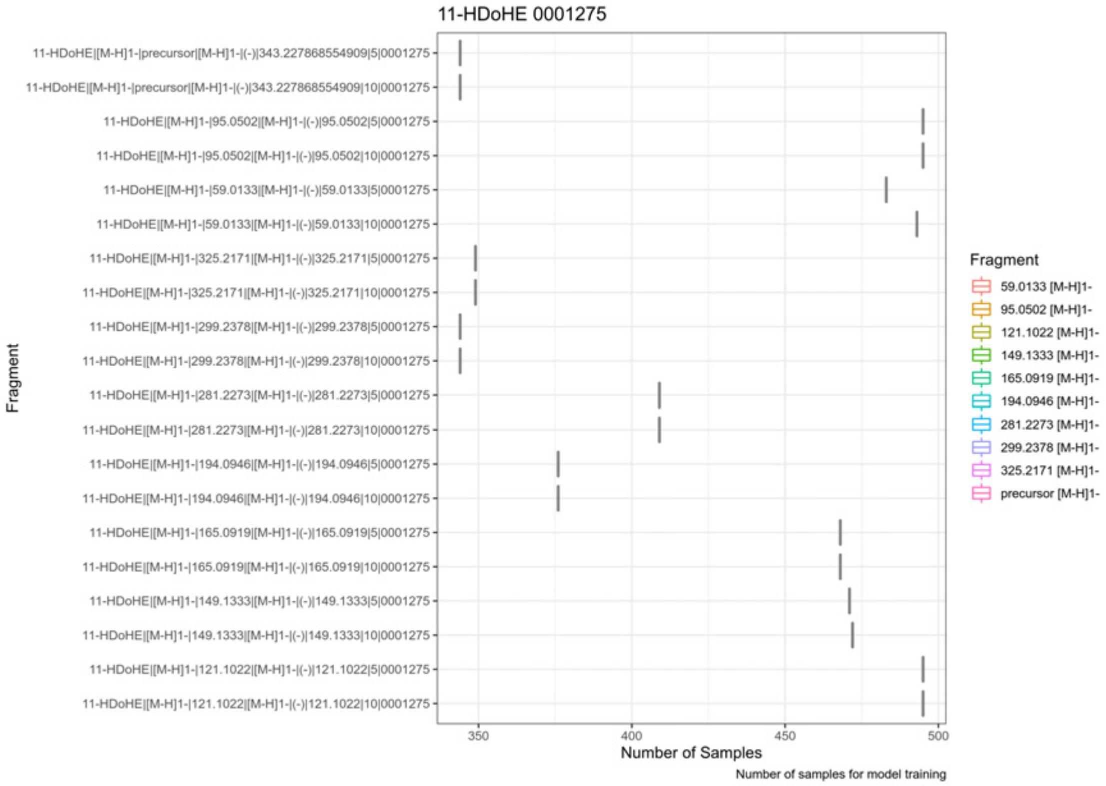

Figure 20. Number of samples used for training per combination Id

1.5. 11-HETE [M-H]1- 0001289

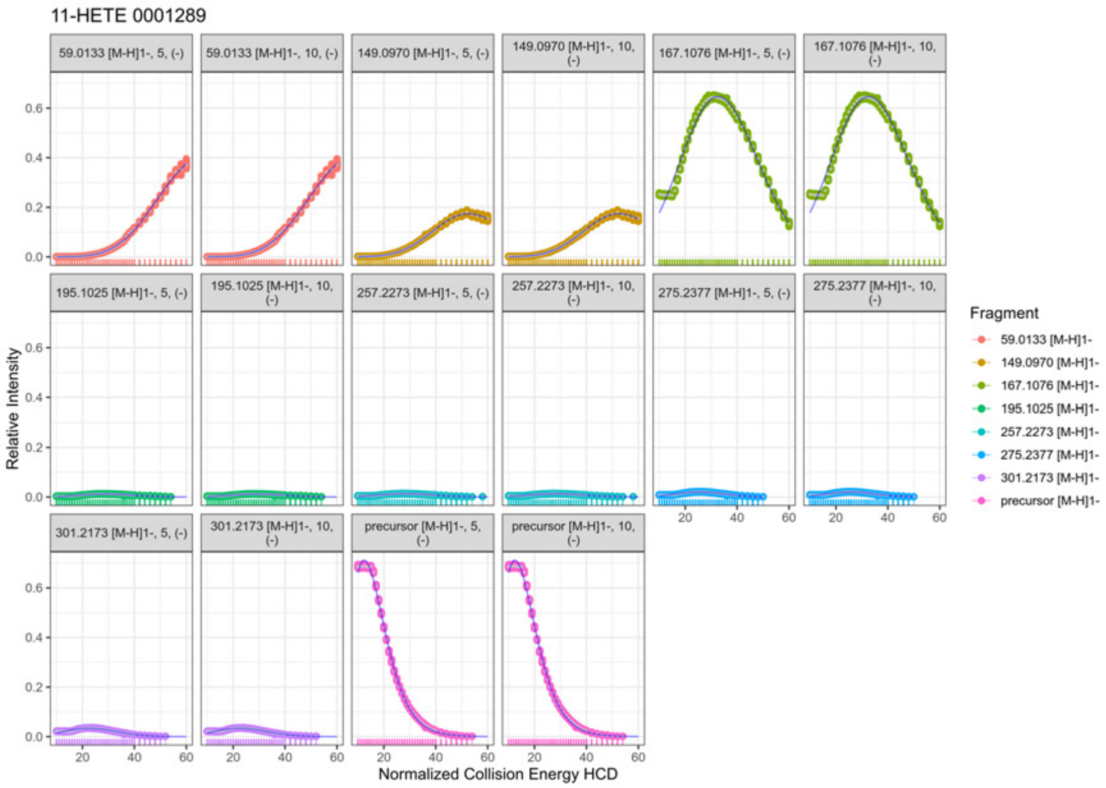

Figure 21. Nonlinear fit

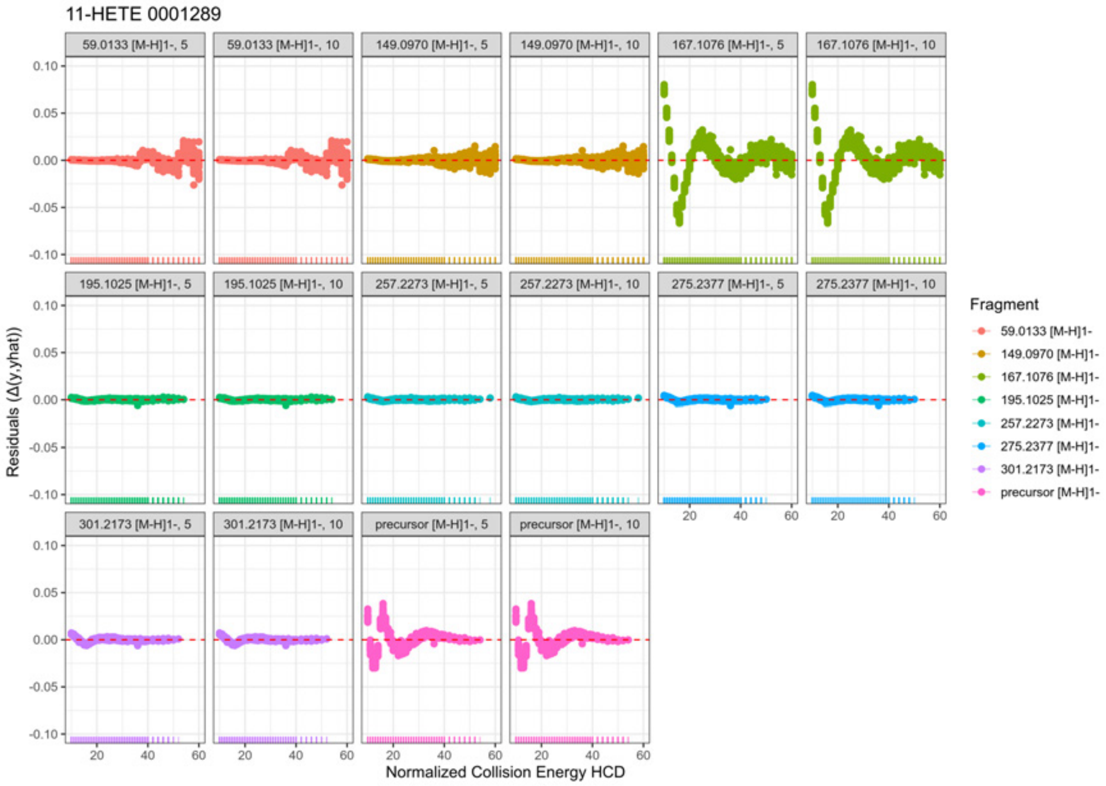

Figure 22. Residuals of nonlinear fit

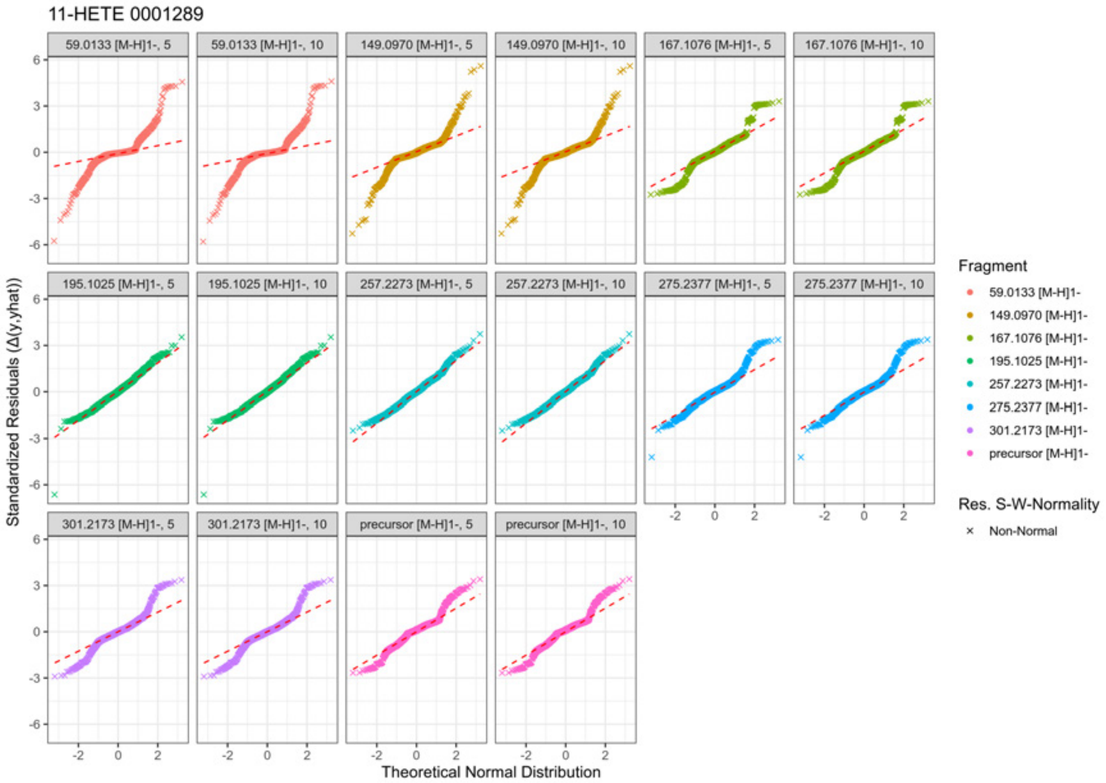

Figure 23. Quantile-quantile plot of residuals

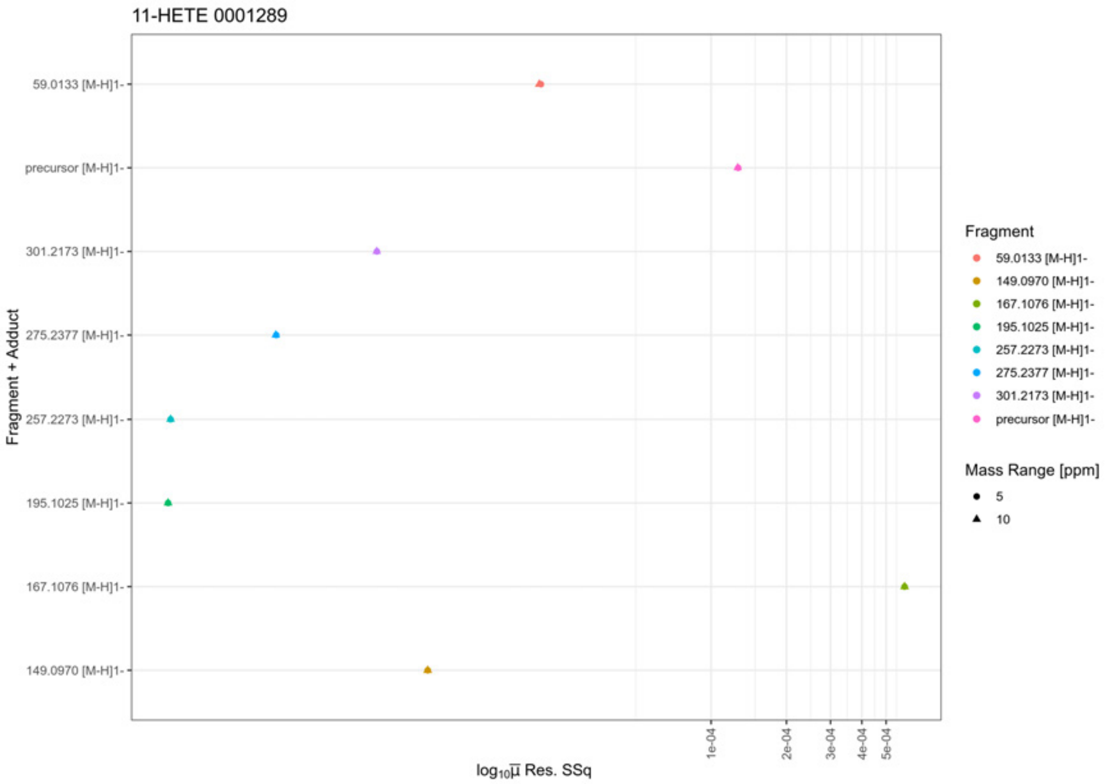

Figure 24. Normalized sum-of-squares of the residuals

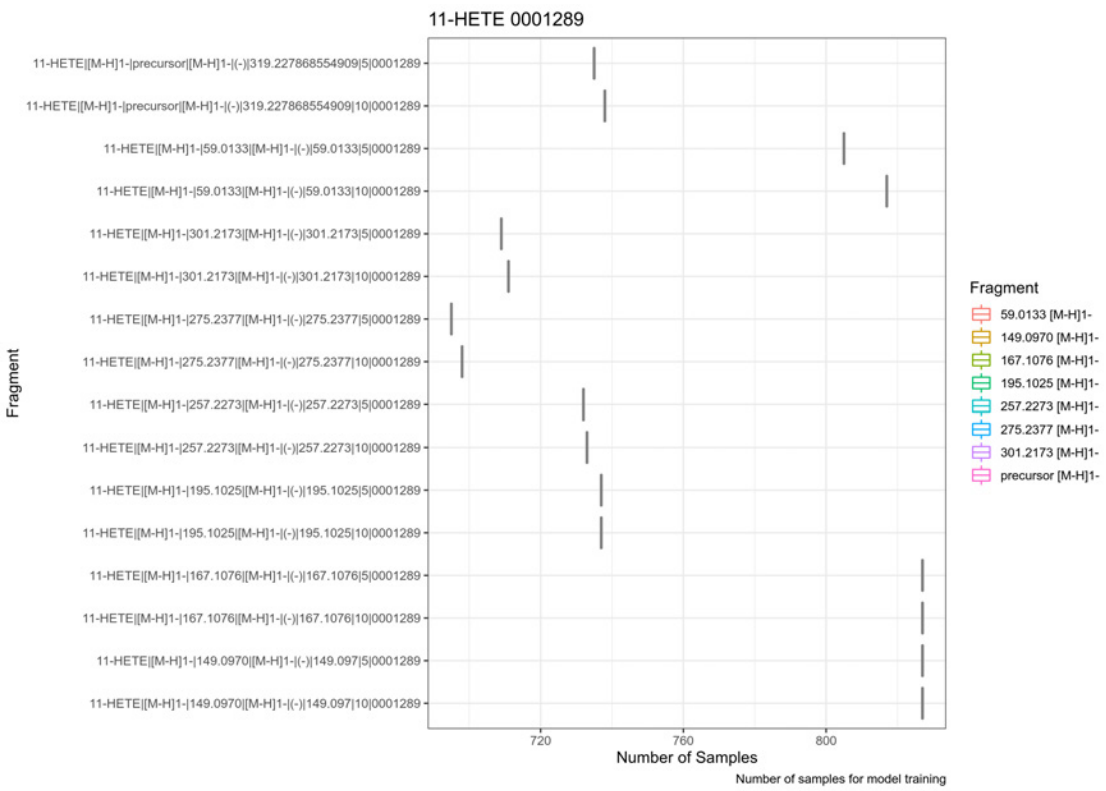

Figure 25. Number of samples used for training per combination Id

1.6. 12(13)-EpOME{d4} [M-H]1- 0001303

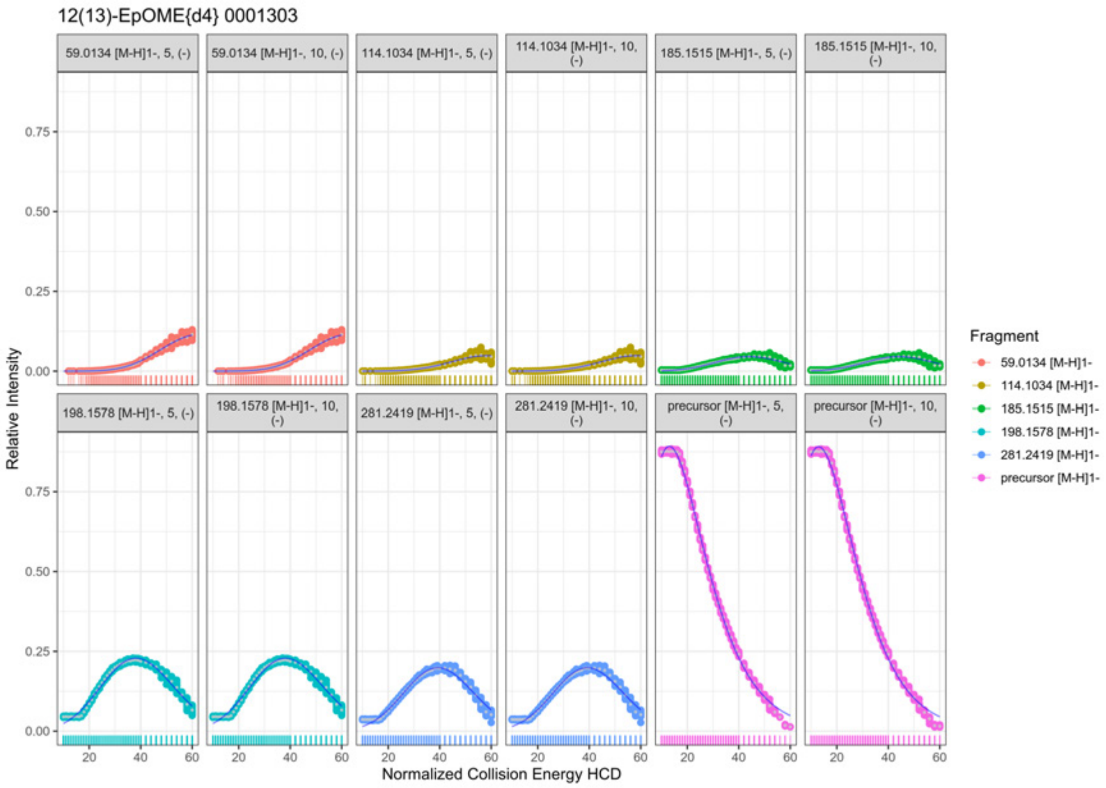

Figure 26. Nonlinear fit

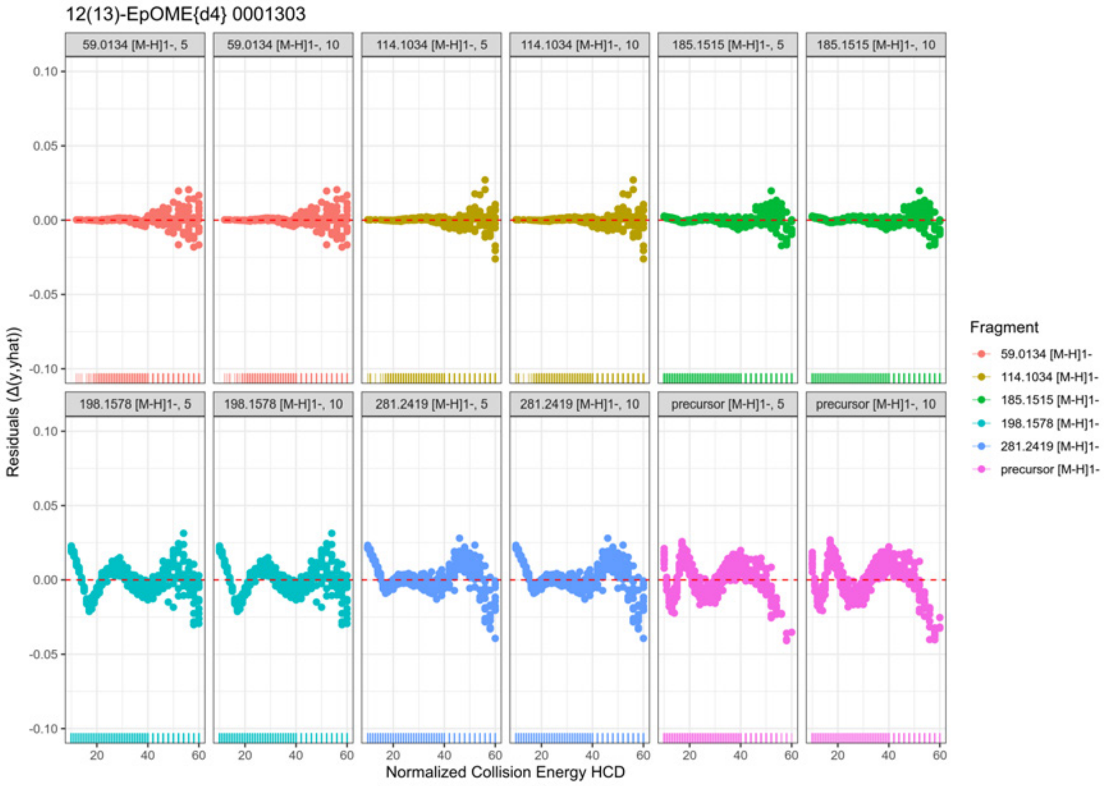

Figure 27. Residuals of nonlinear fit

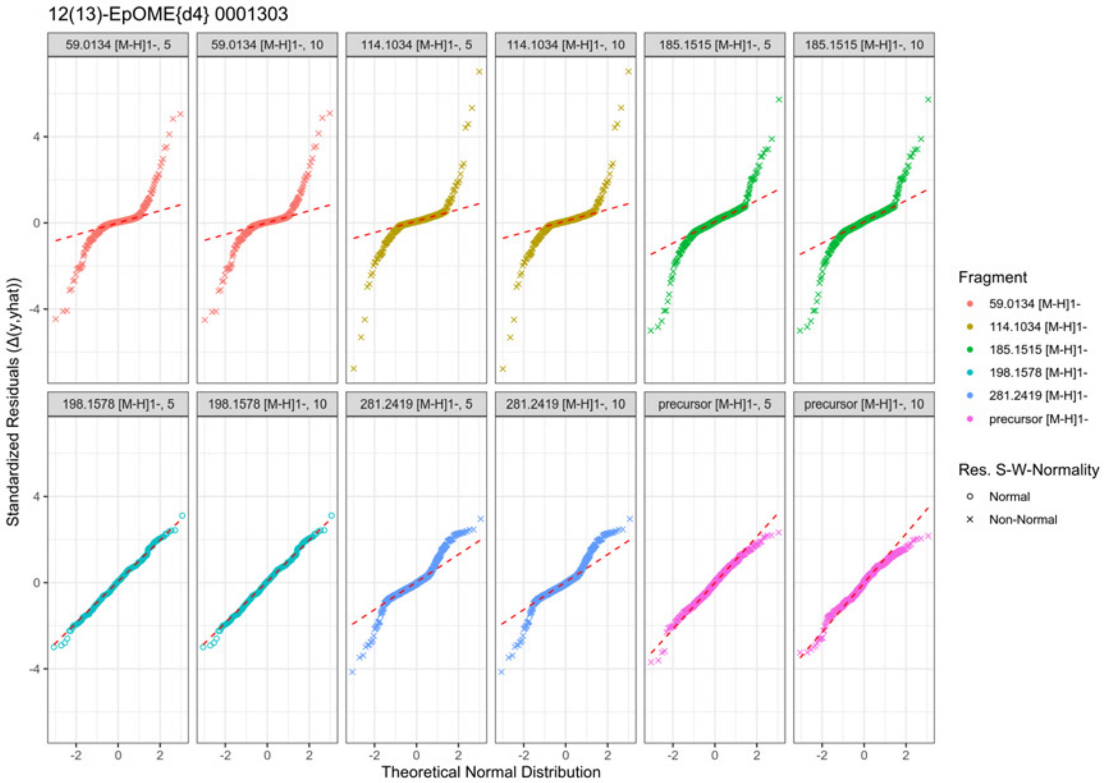

Figure 28. Quantile-quantile plot of residuals

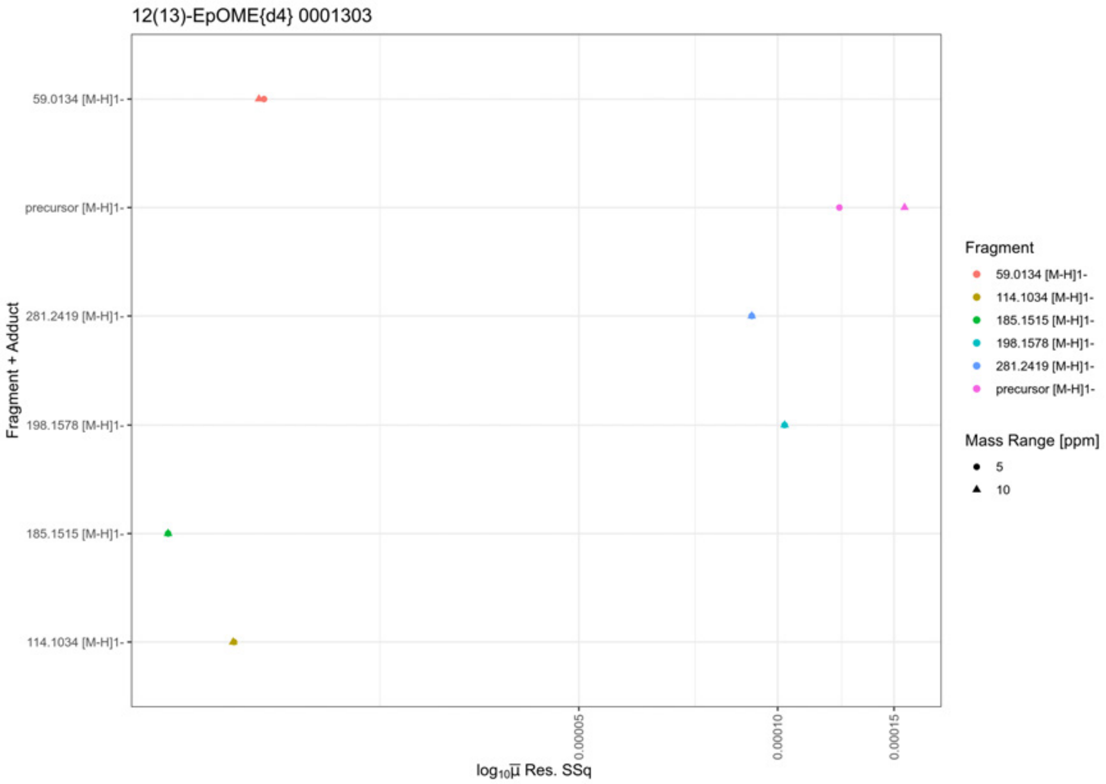

Figure 29. Normalized sum-of-squares of the residuals

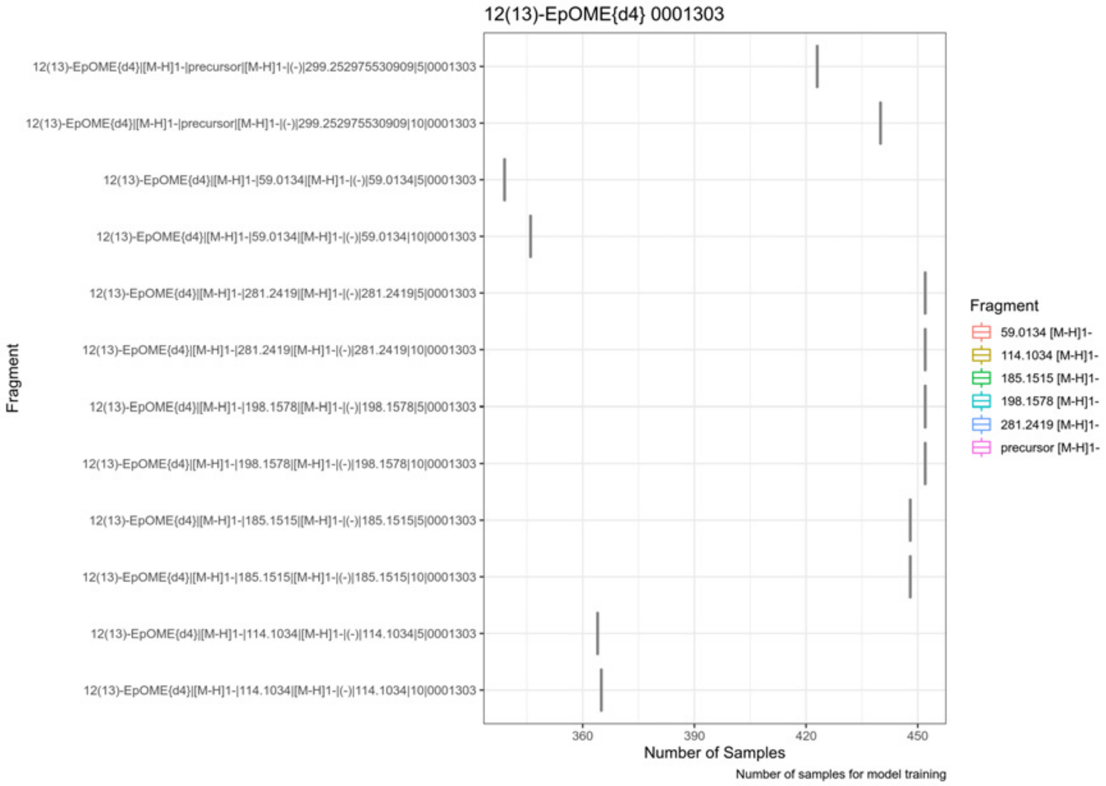

Figure 30. Number of samples used for training per combination Id

1.7. 12-HEPE [M-H]1- 0001281

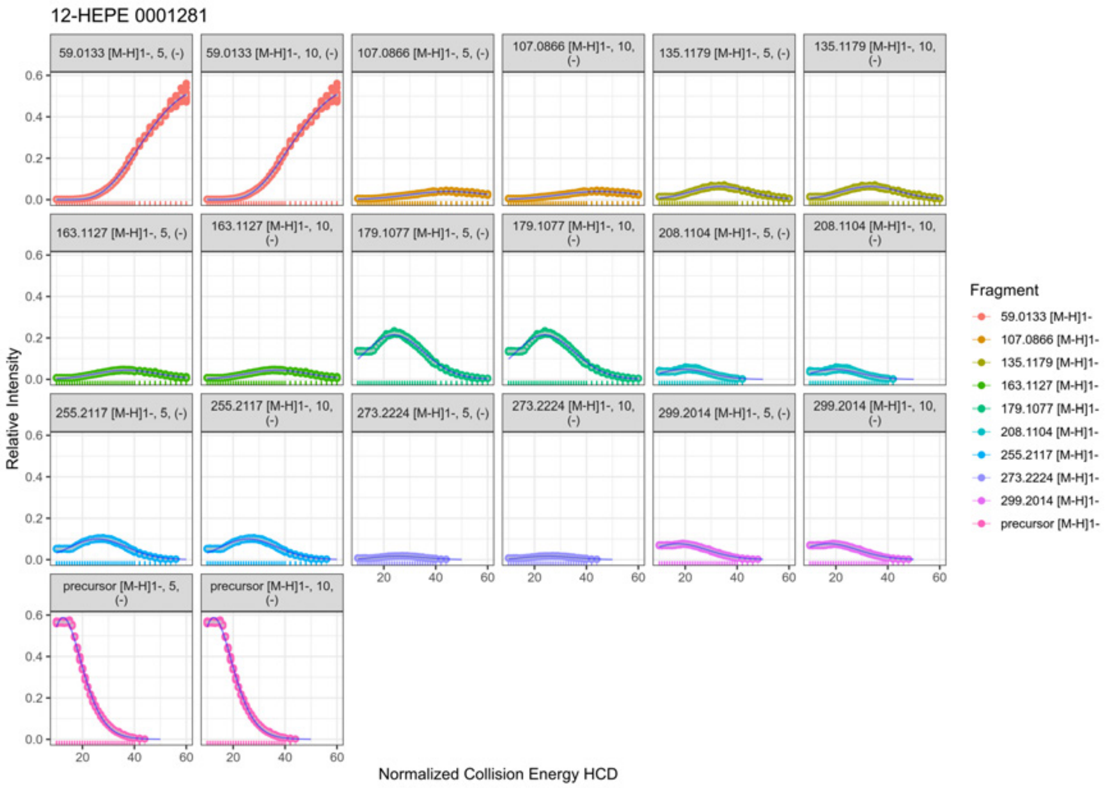

Figure 31. Nonlinear fit

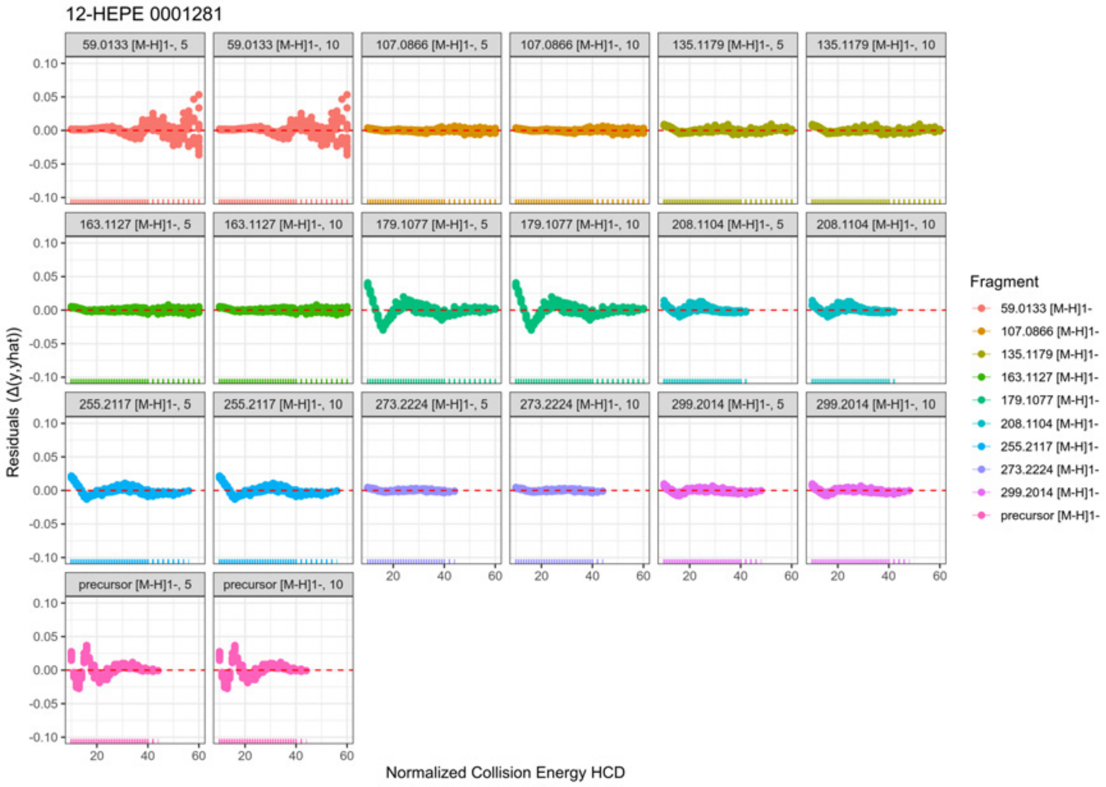

Figure 32. Residuals of nonlinear fit

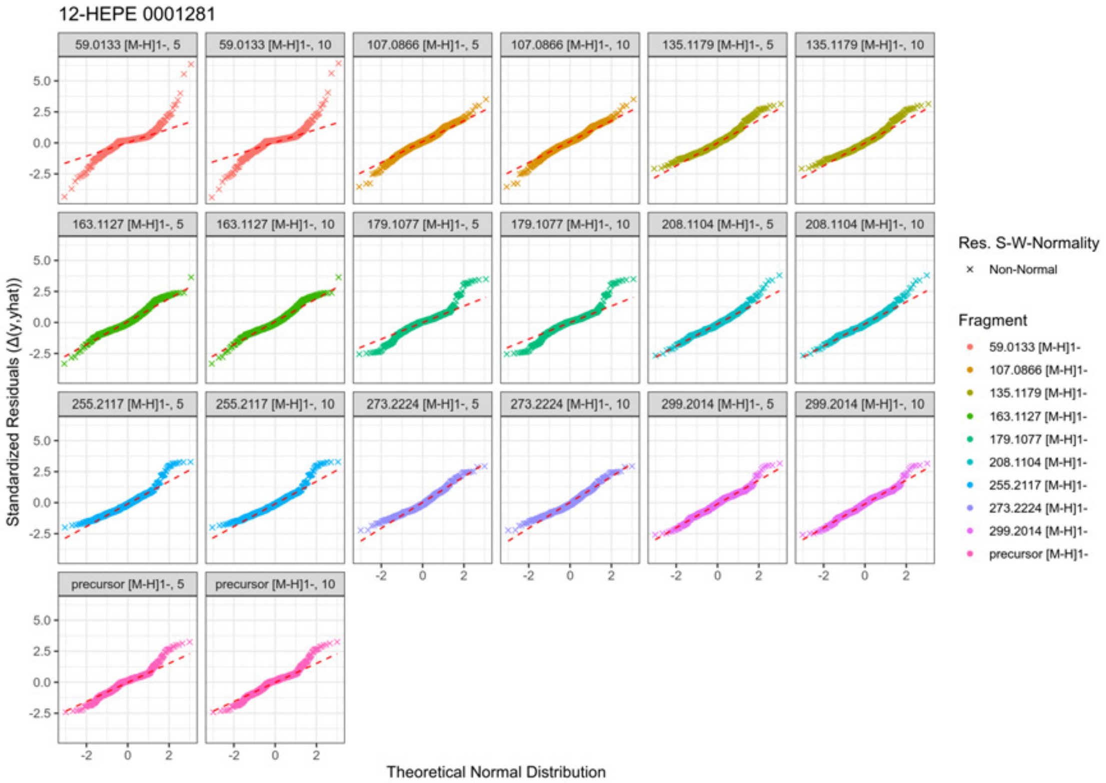

Figure 33. Quantile-quantile plot of residuals

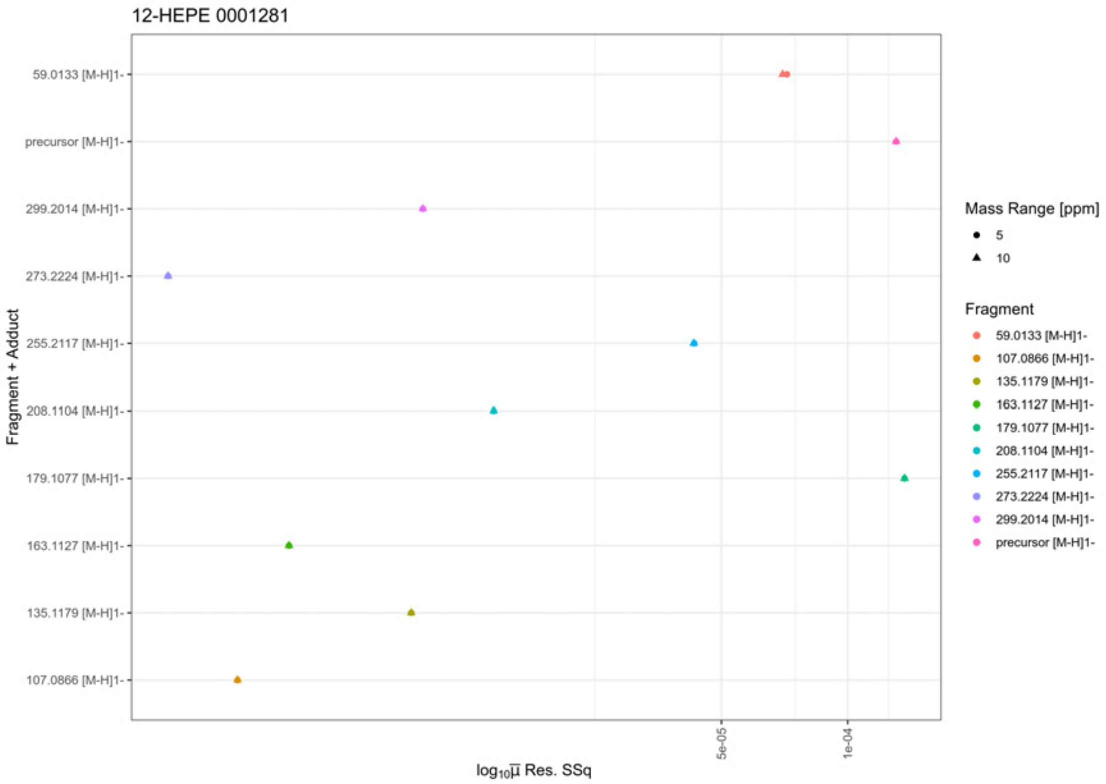

Figure 34. Normalized sum-of-squares of the residuals

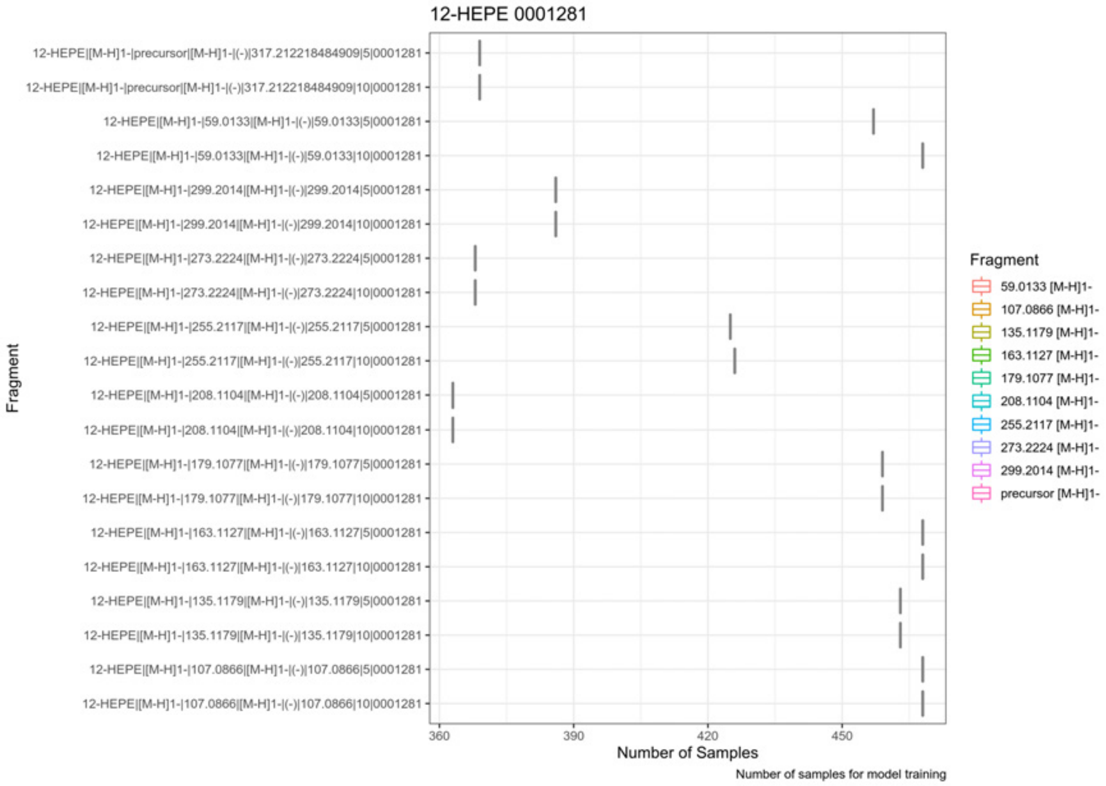

Figure 35. Number of samples used for training per combination Id

1.8. 12-HETE{d8} [M-H]1- 0001295

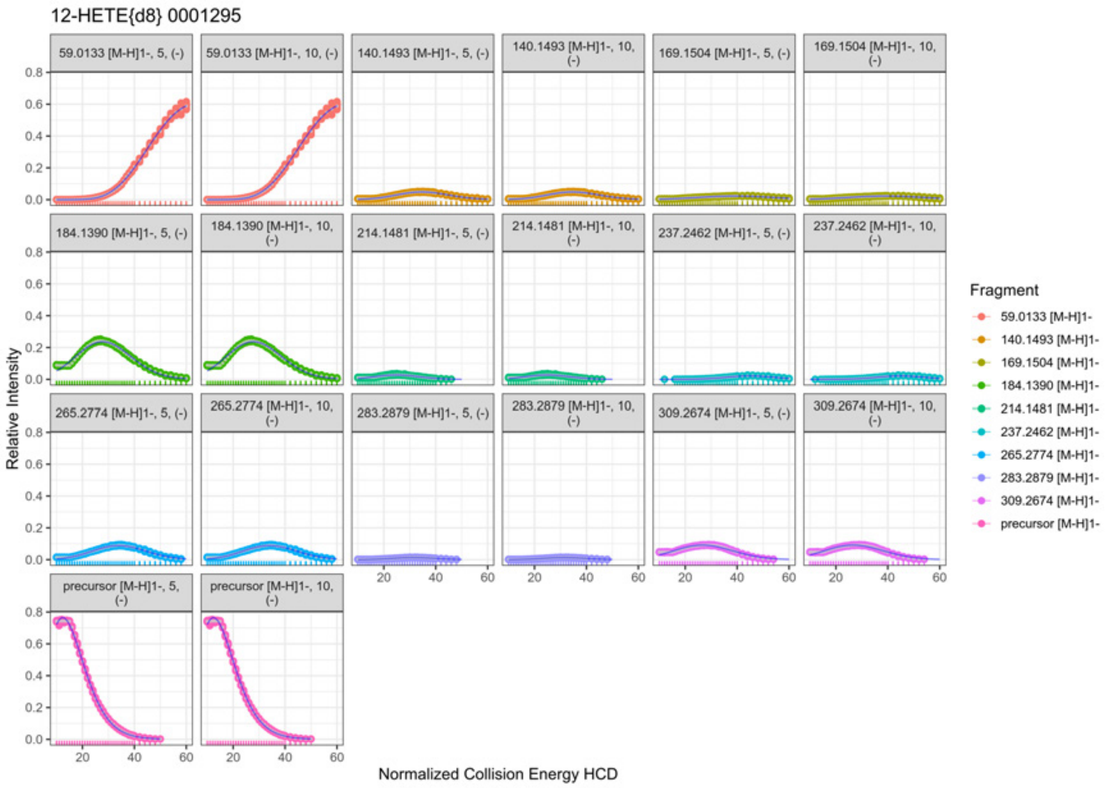

Figure 36. Nonlinear fit

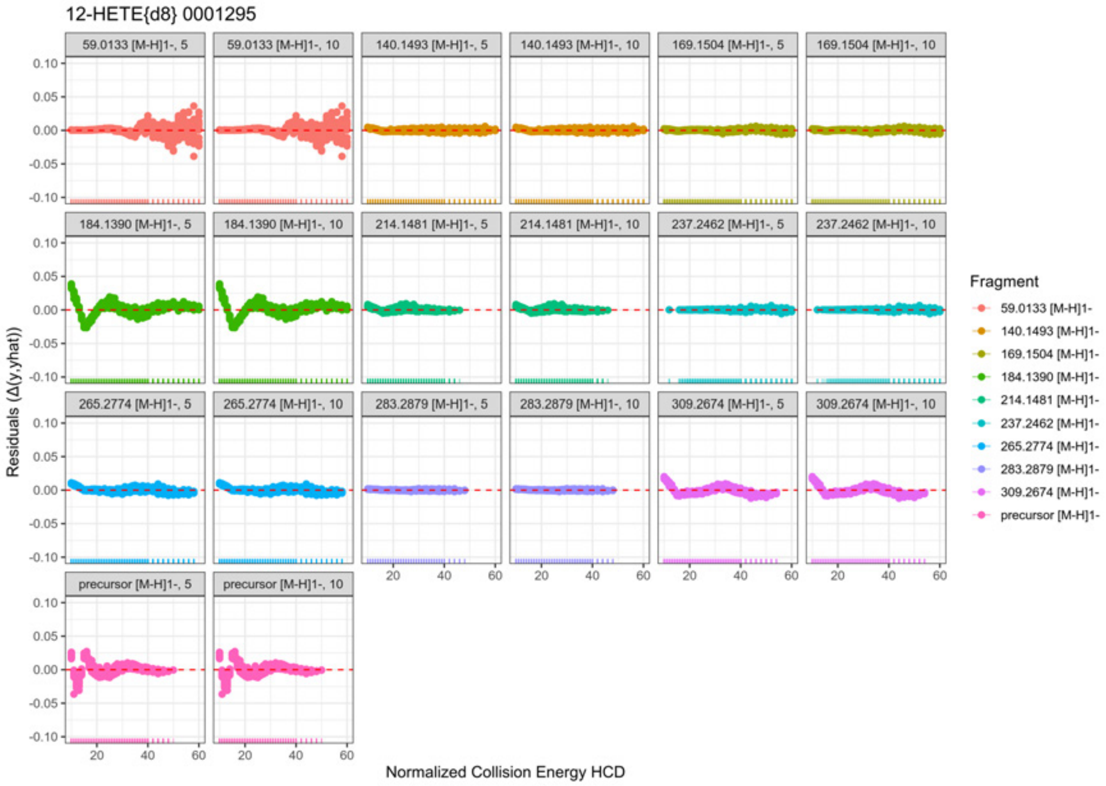

Figure 37. Residuals of nonlinear fit

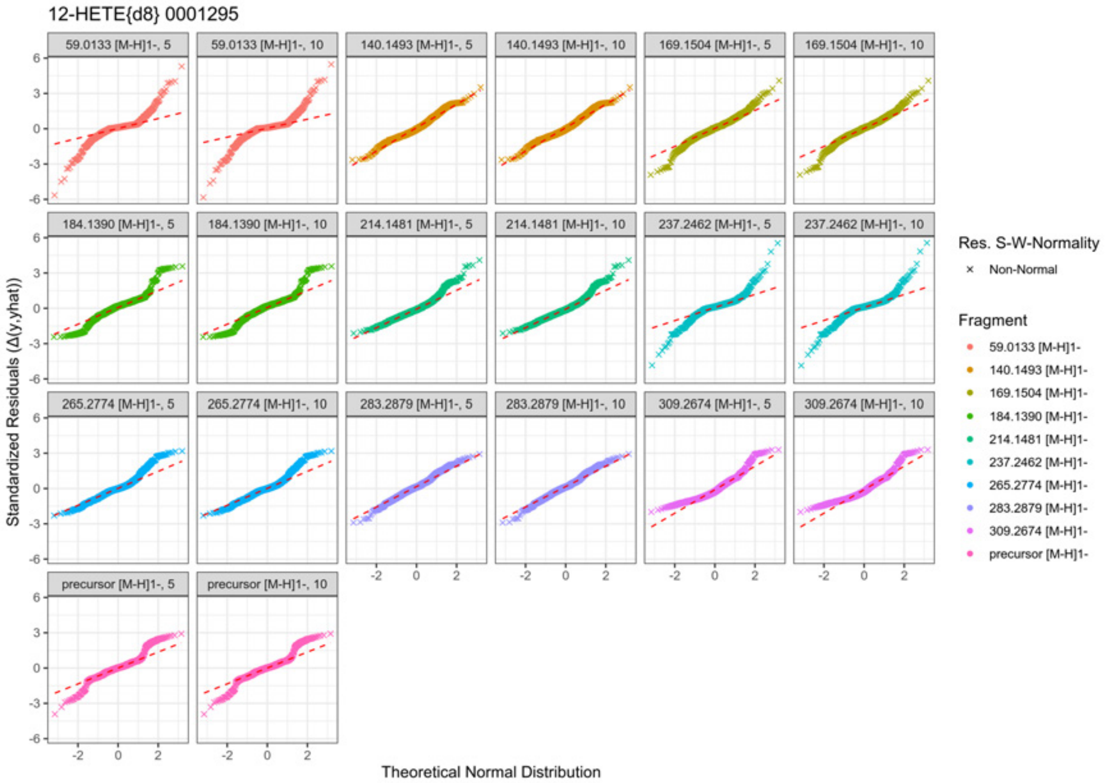

Figure 38. Quantile-quantile plot of residuals

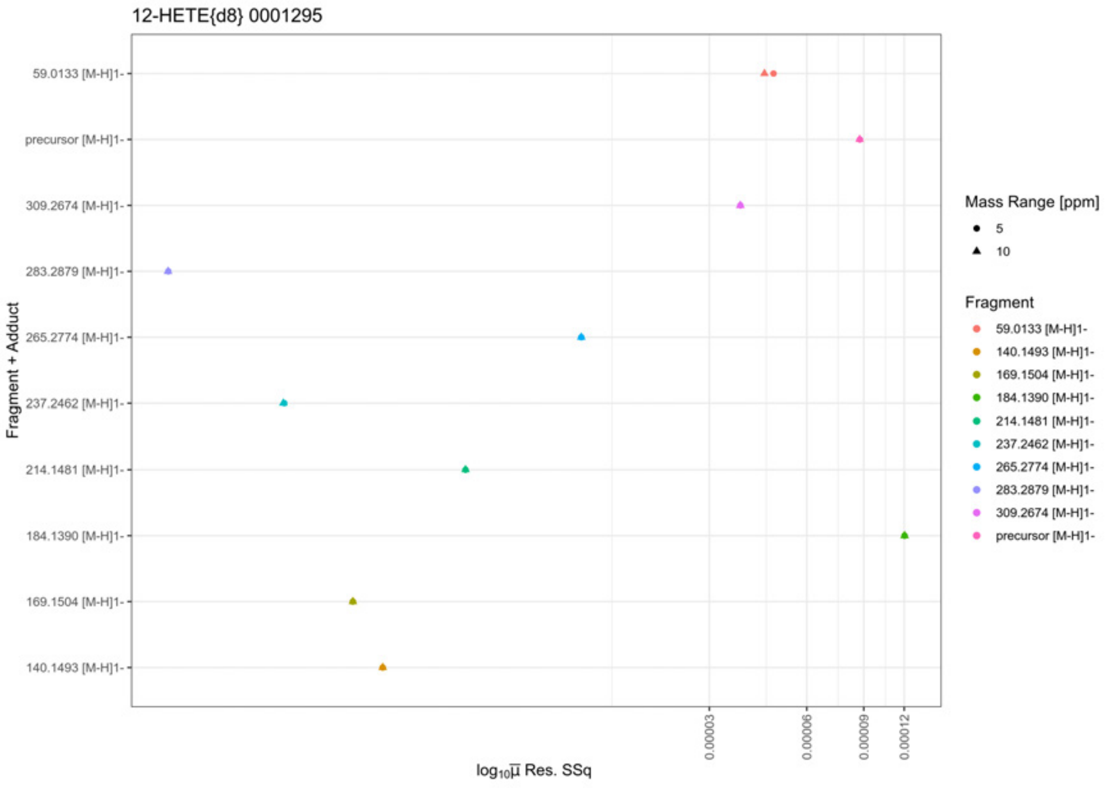

Figure 39. Normalized sum-of-squares of the residuals

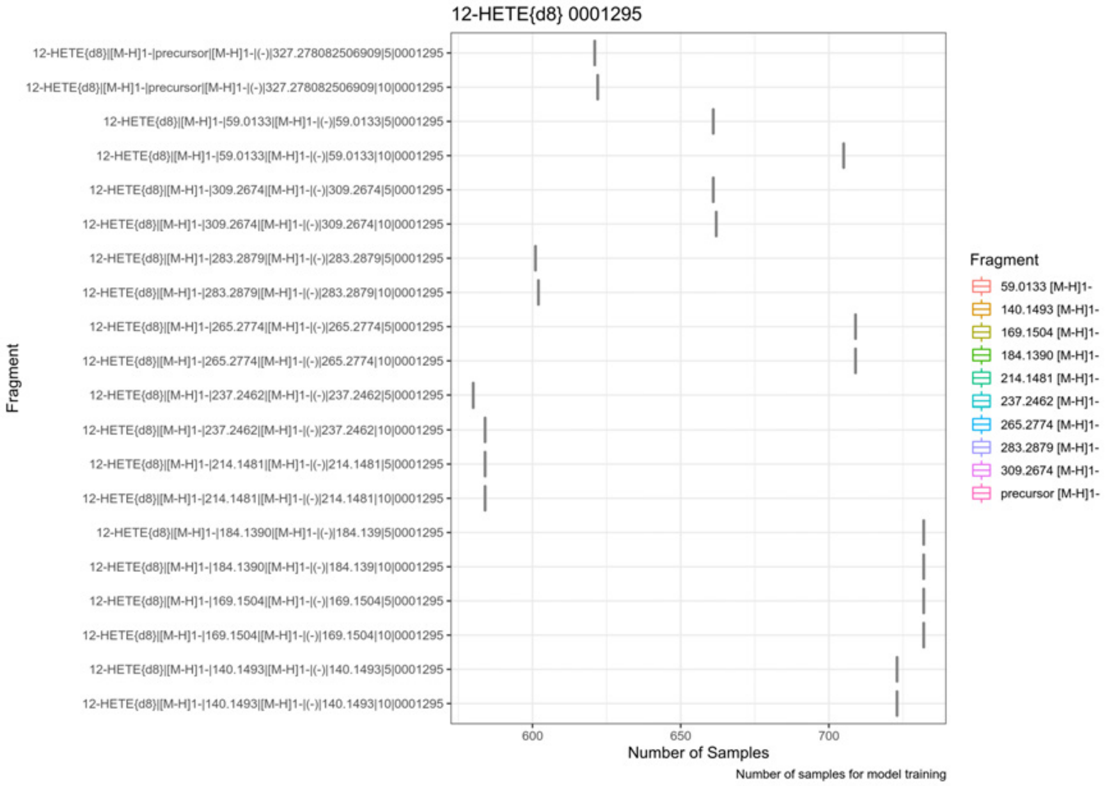

Figure 40. Number of samples used for training per combination Id

1.9. 12-HHTrE [M-H]1- 0001347

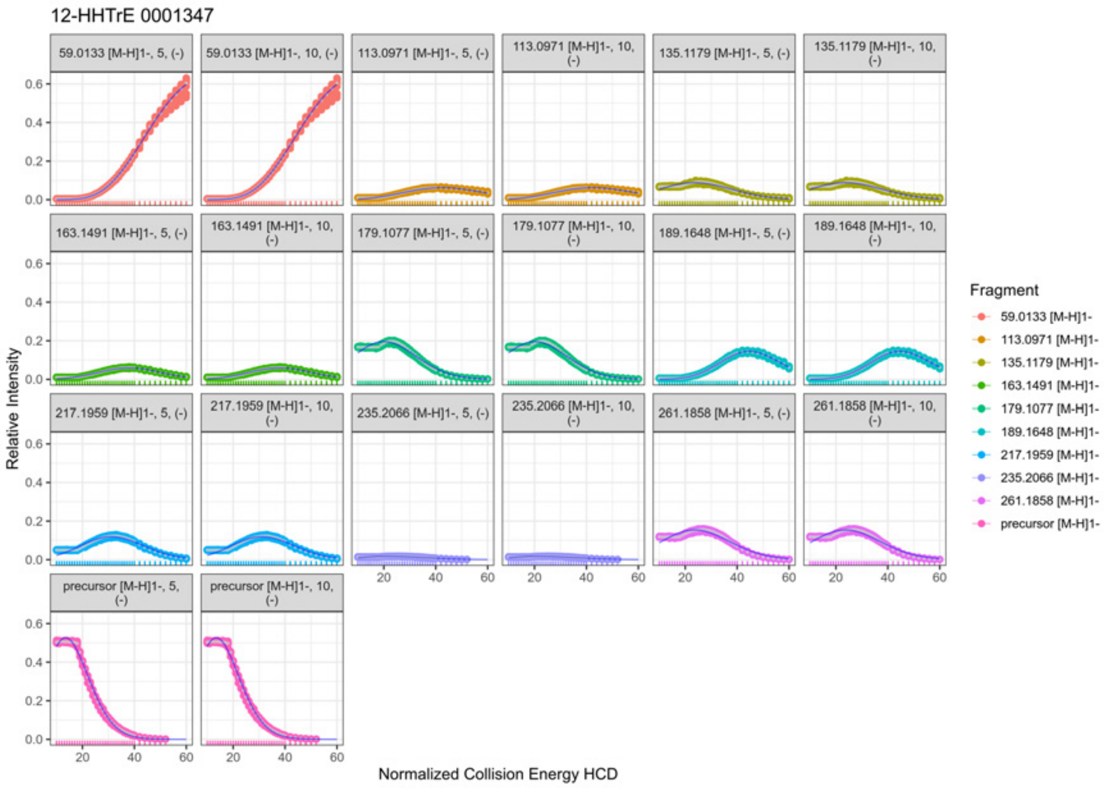

Figure 41. Nonlinear fit

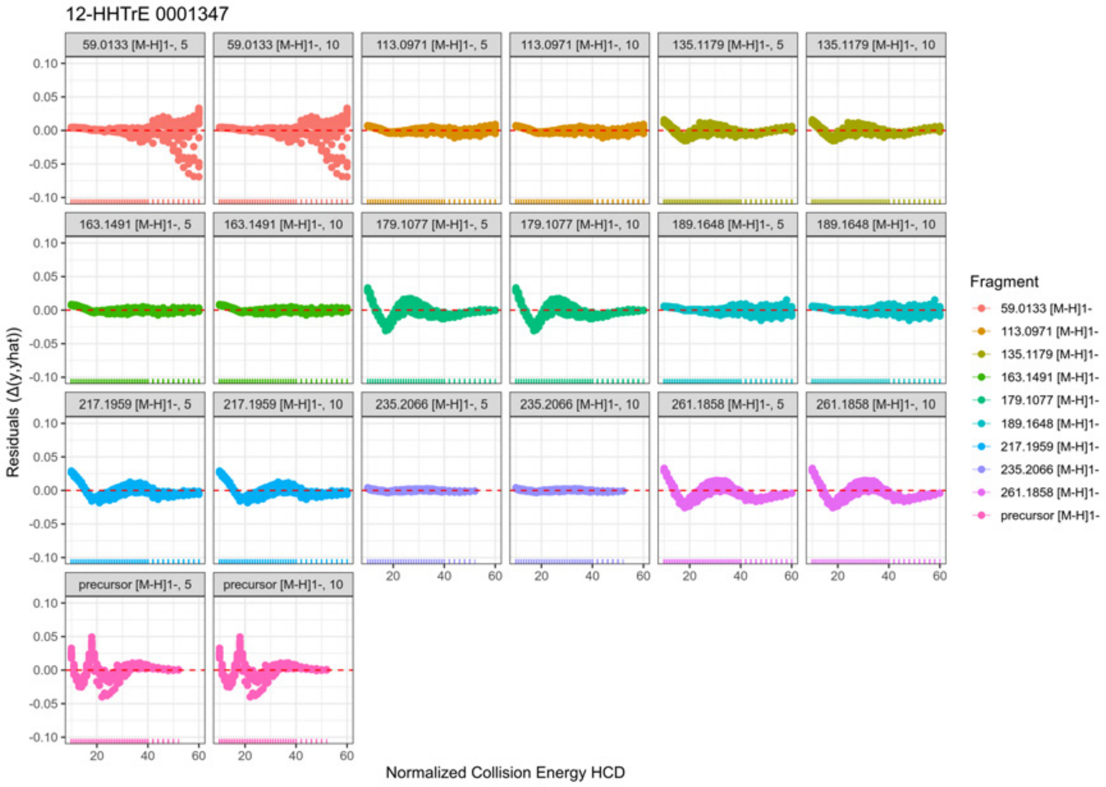

Figure 42. Residuals of nonlinear fit

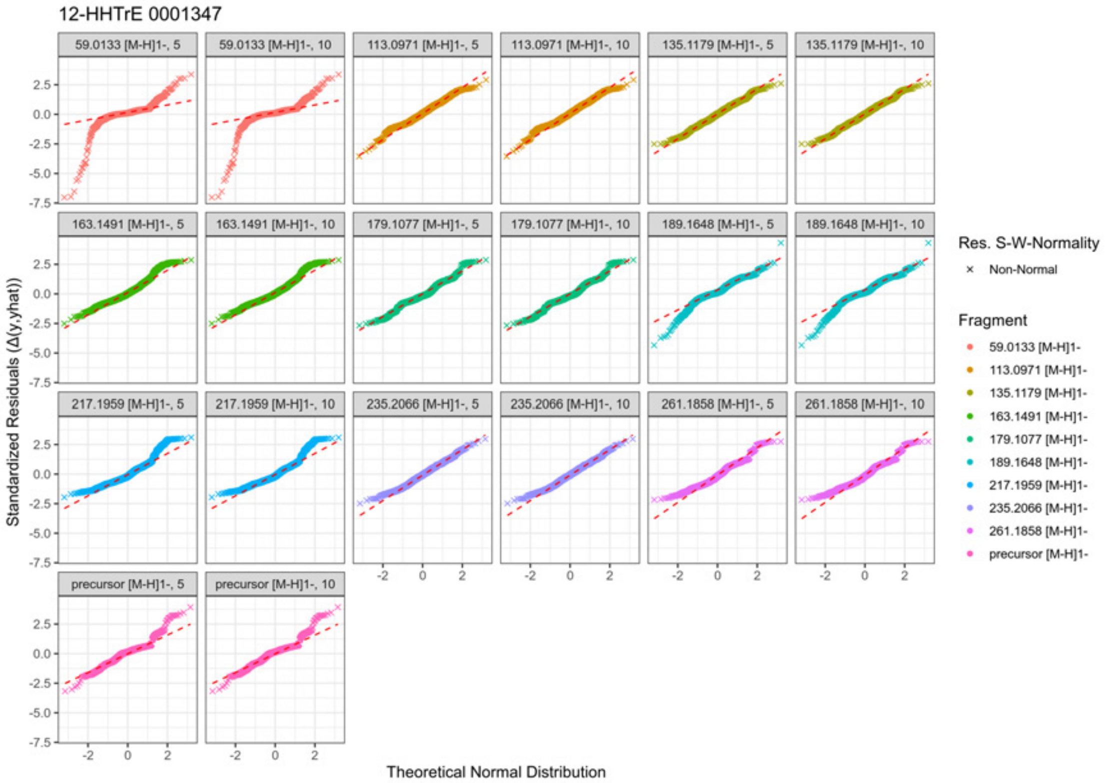

Figure 43. Quantile-quantile plot of residuals

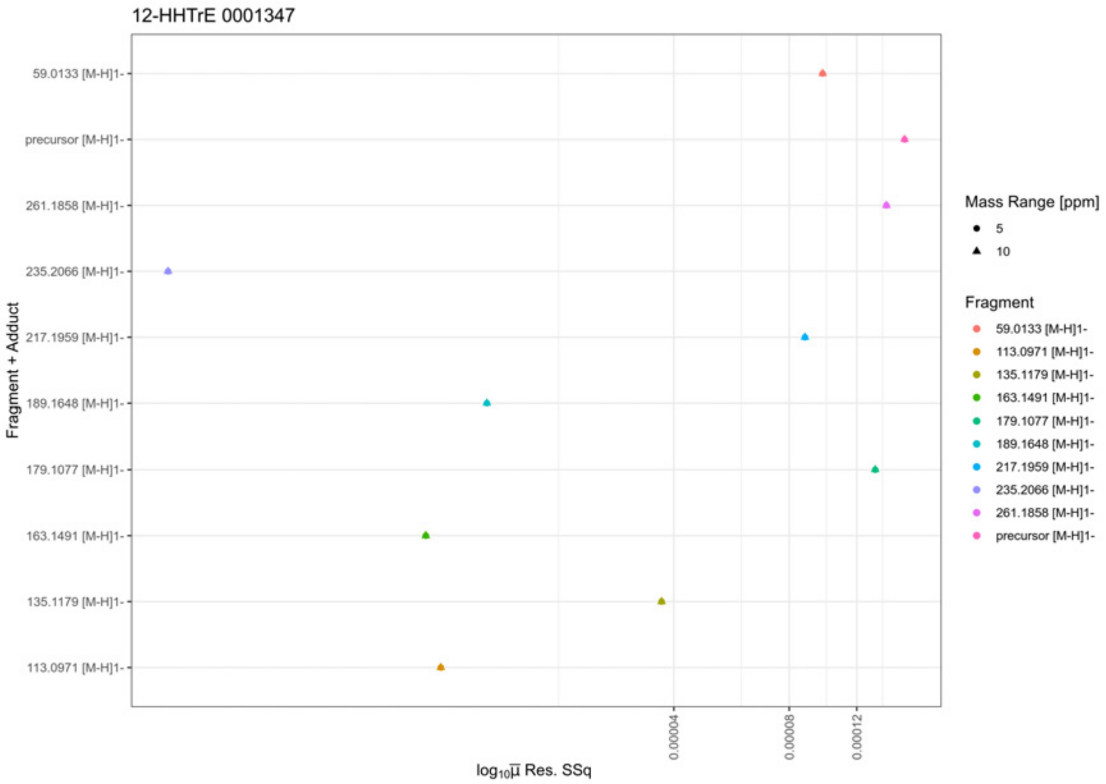

Figure 44. Normalized sum-of-squares of the residuals

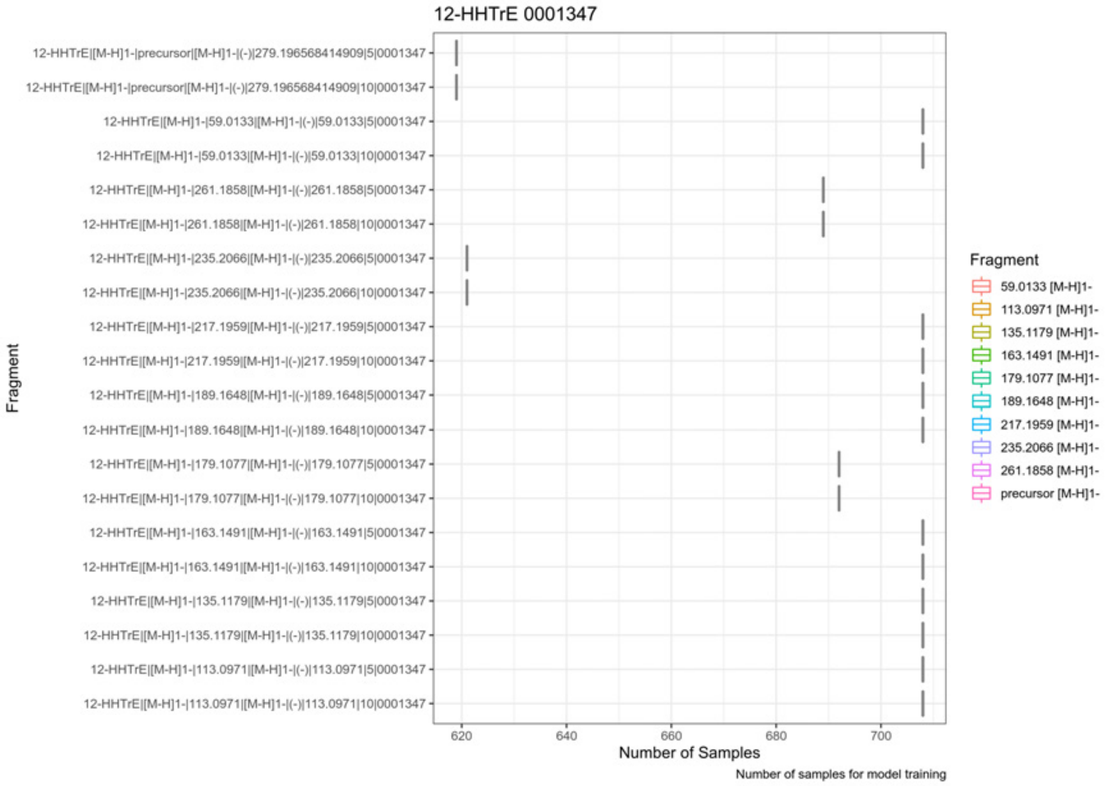

Figure 45. Number of samples used for training per combination Id

1.10. 12-OxoETE [M-H]1- 0001283

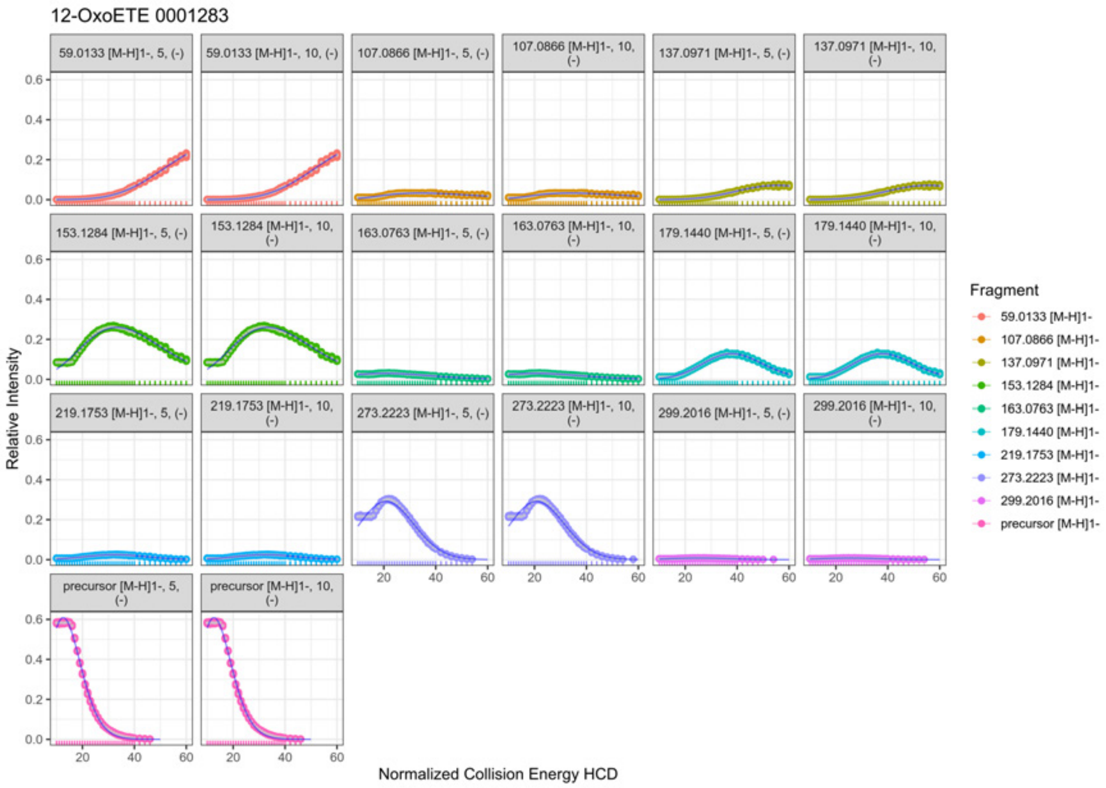

Figure 46. Nonlinear fit

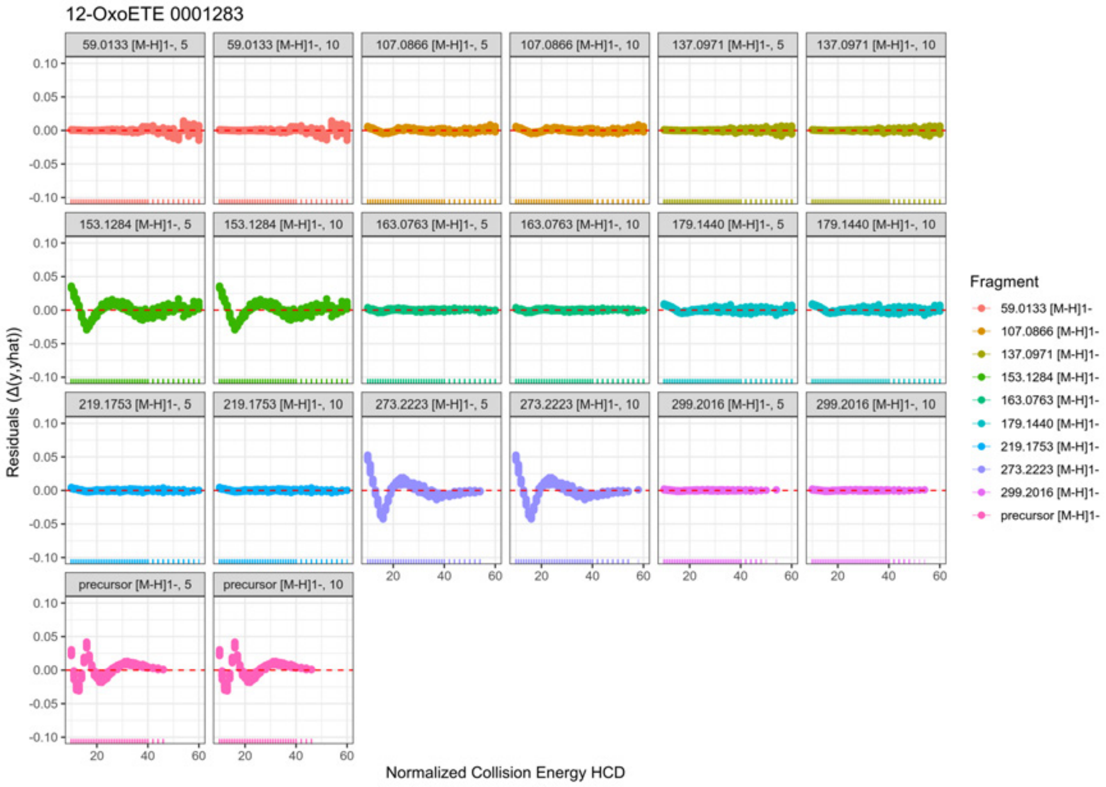

Figure 47. Residuals of nonlinear fit

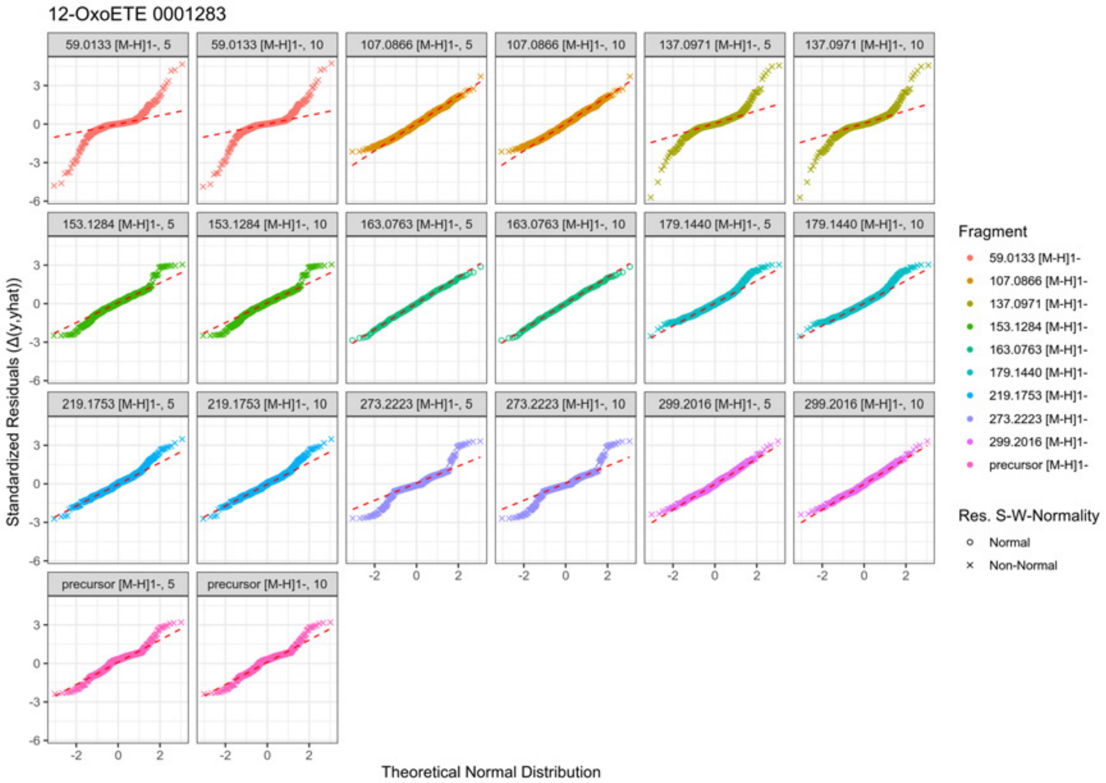

Figure 48. Quantile-quantile plot of residuals

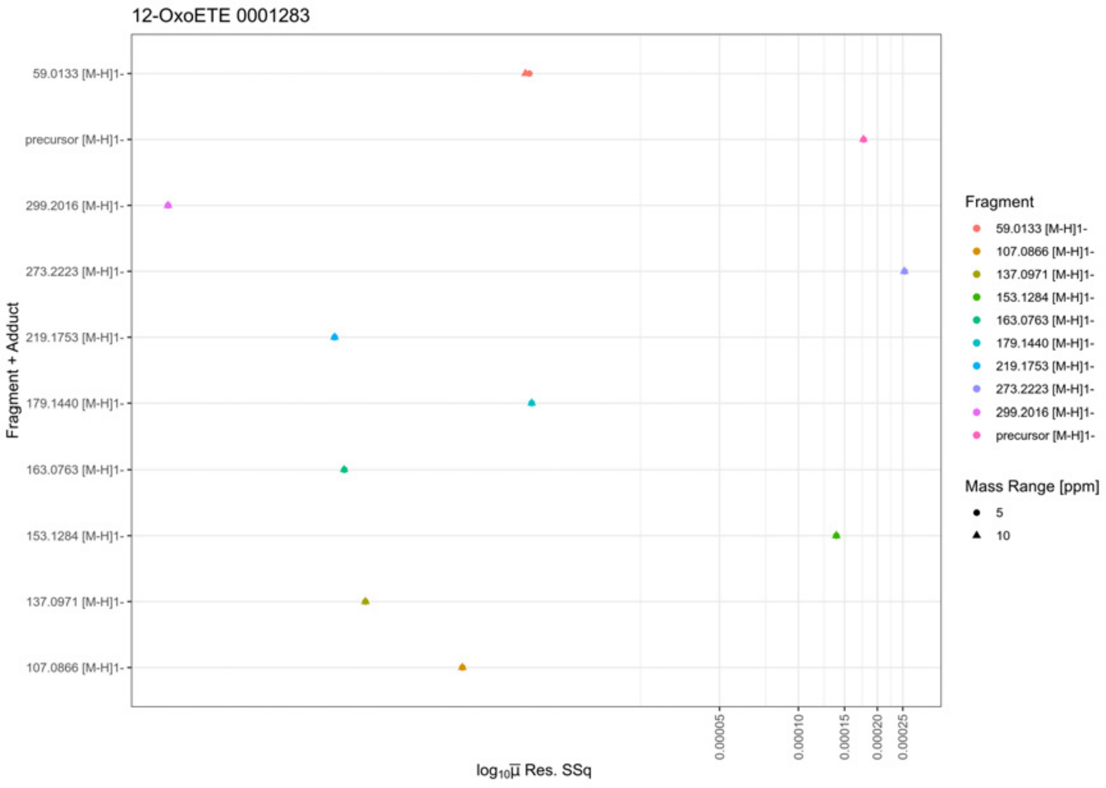

Figure 49. Normalized sum-of-squares of the residuals

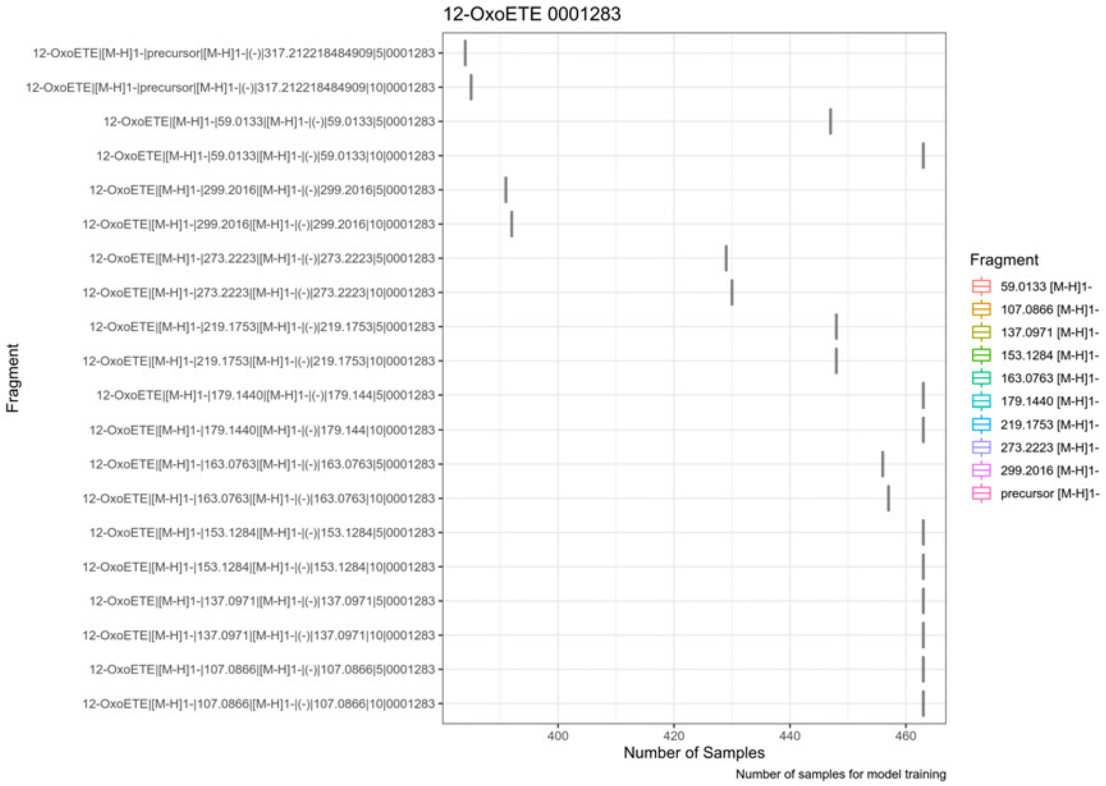

Figure 50. Number of samples used for training per combination Id

1.11. 13-HODE{d4} [M-H]1- 0001277

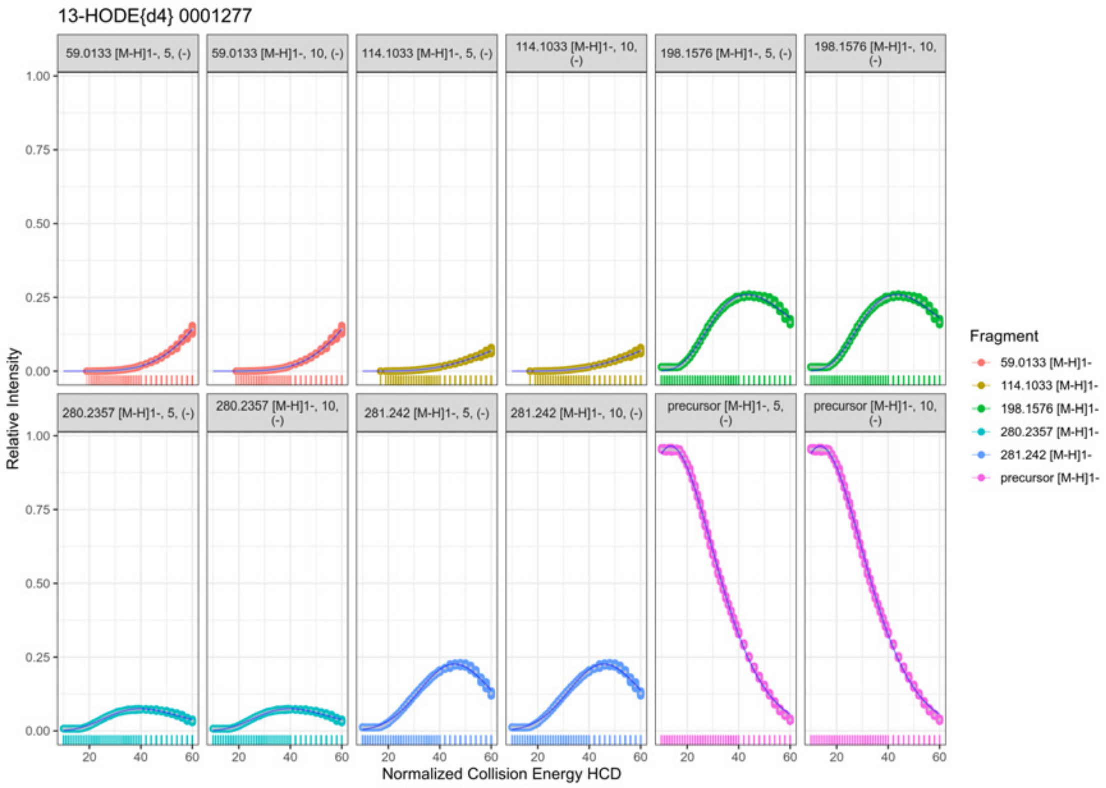

Figure 51. Nonlinear fit

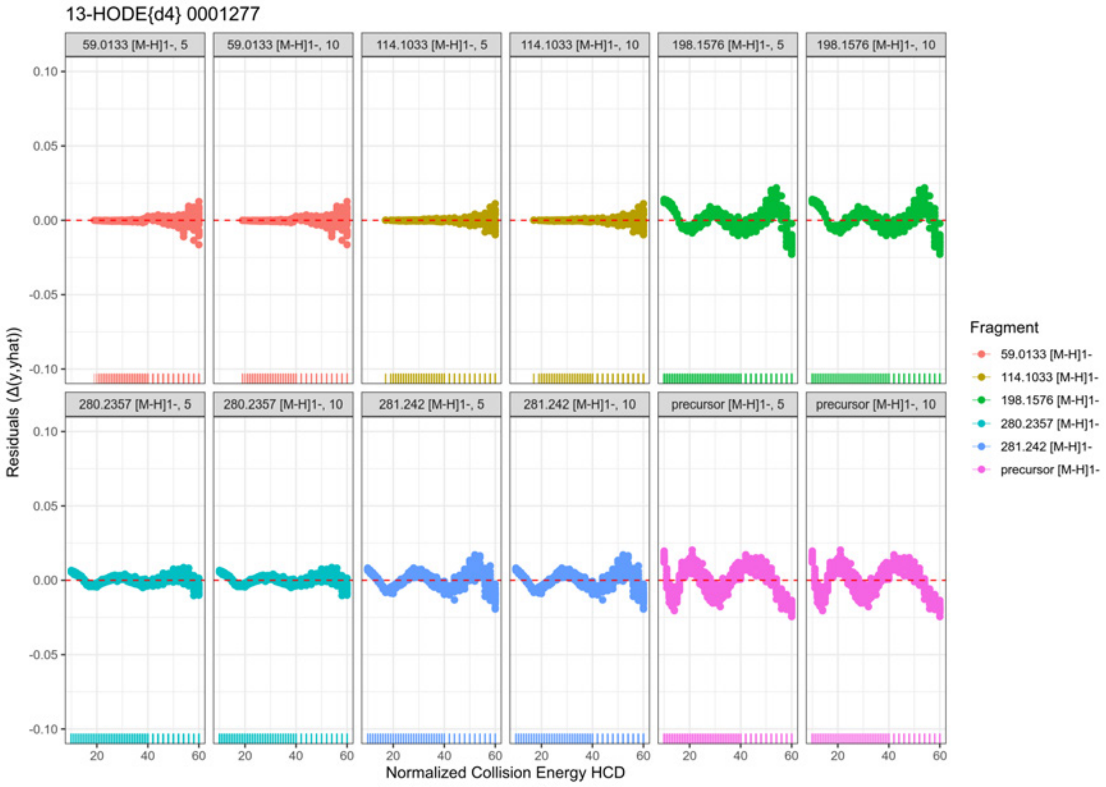

Figure 52. Residuals of nonlinear fit

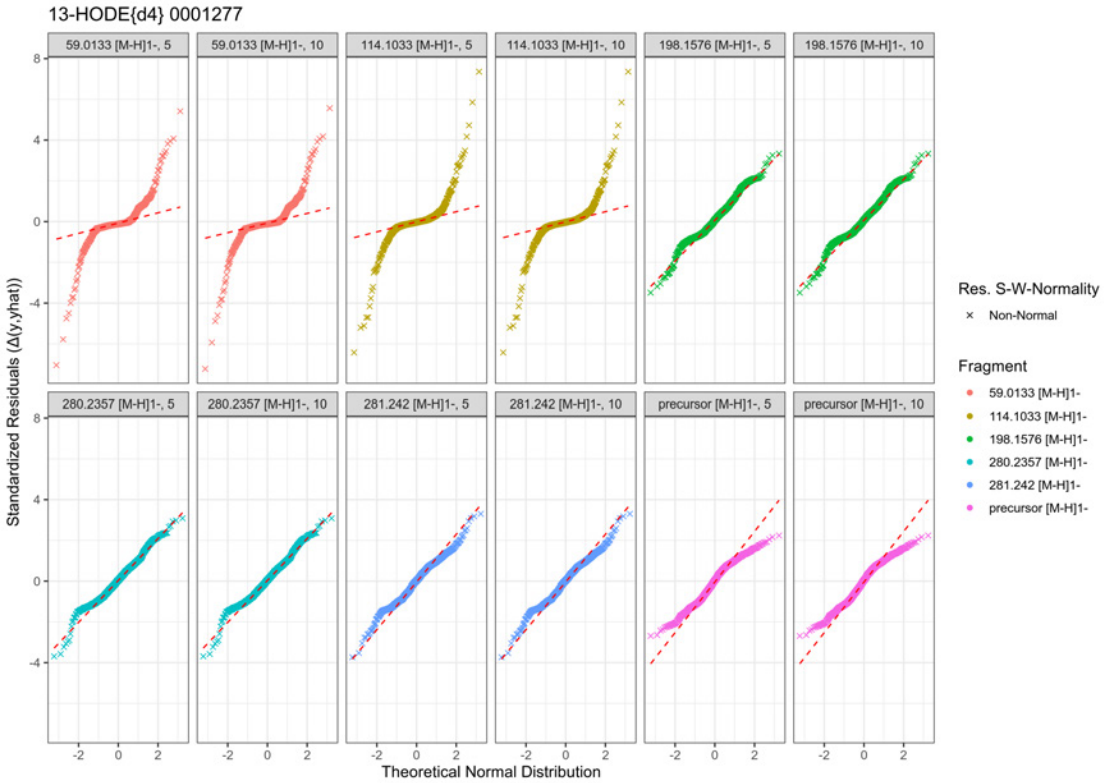

Figure 53. Quantile-quantile plot of residuals

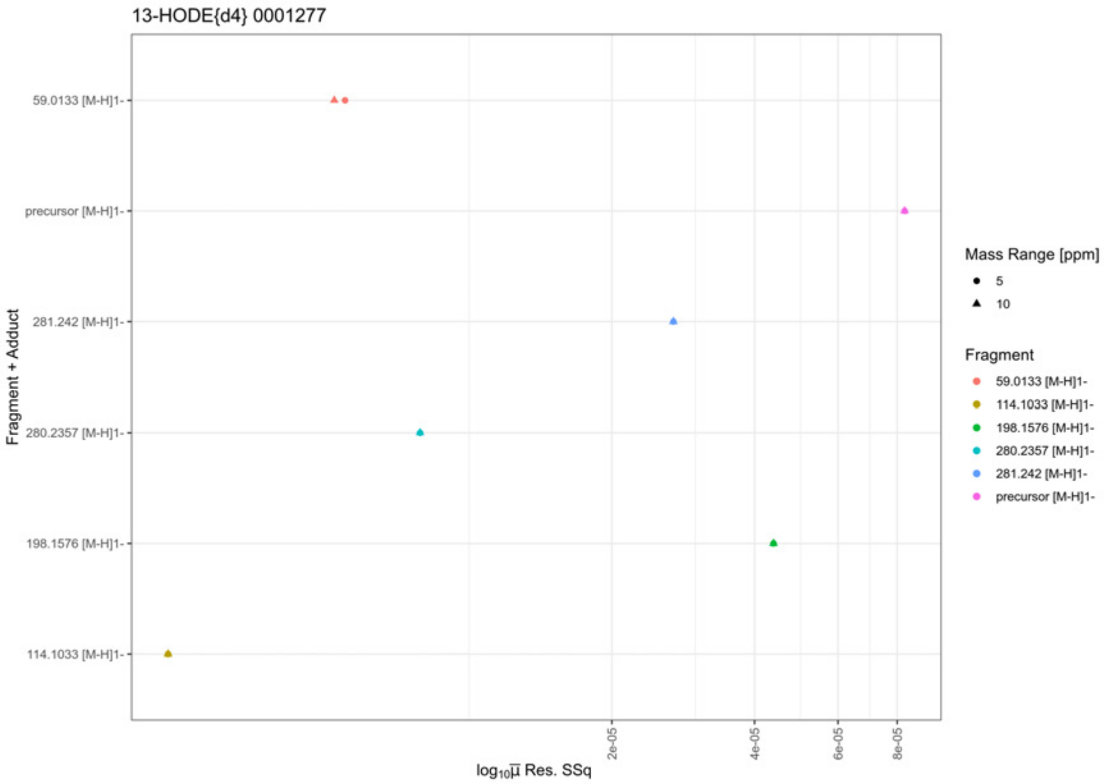

Figure 54. Normalized sum-of-squares of the residuals

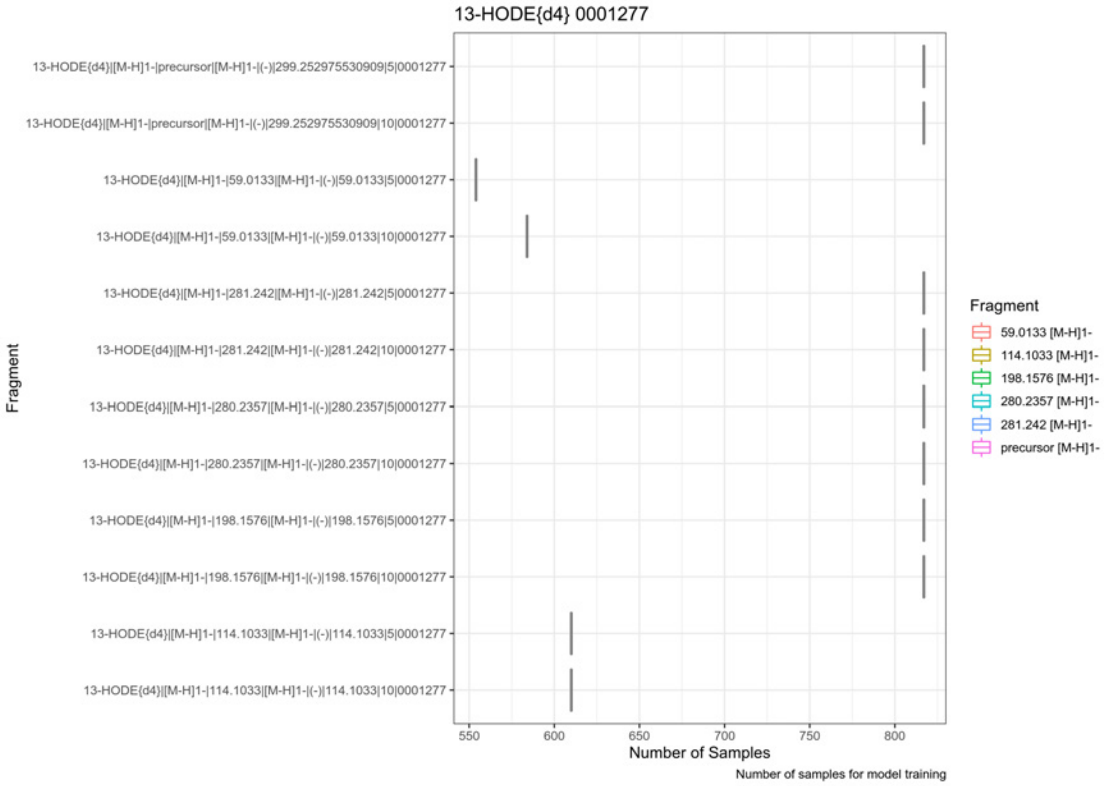

Figure 55. Number of samples used for training per combination Id

1.12. 13-HOTrE [M-H]1- 0001341

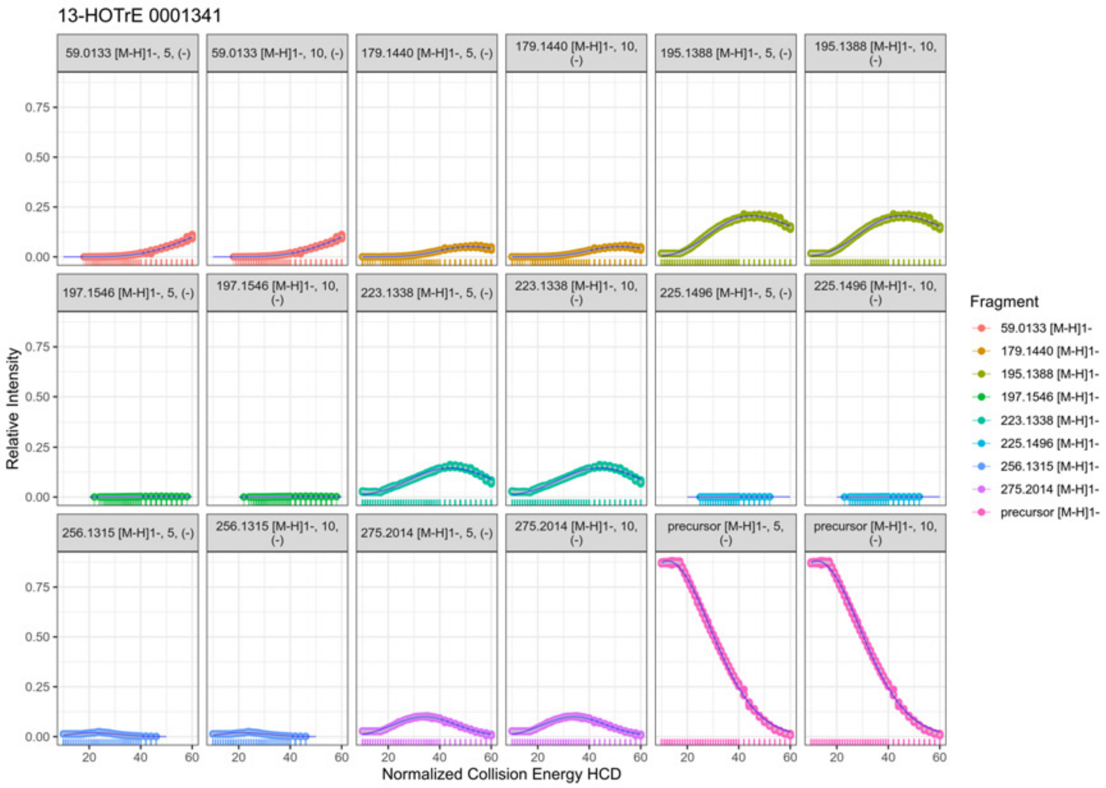

Figure 56. Nonlinear fit

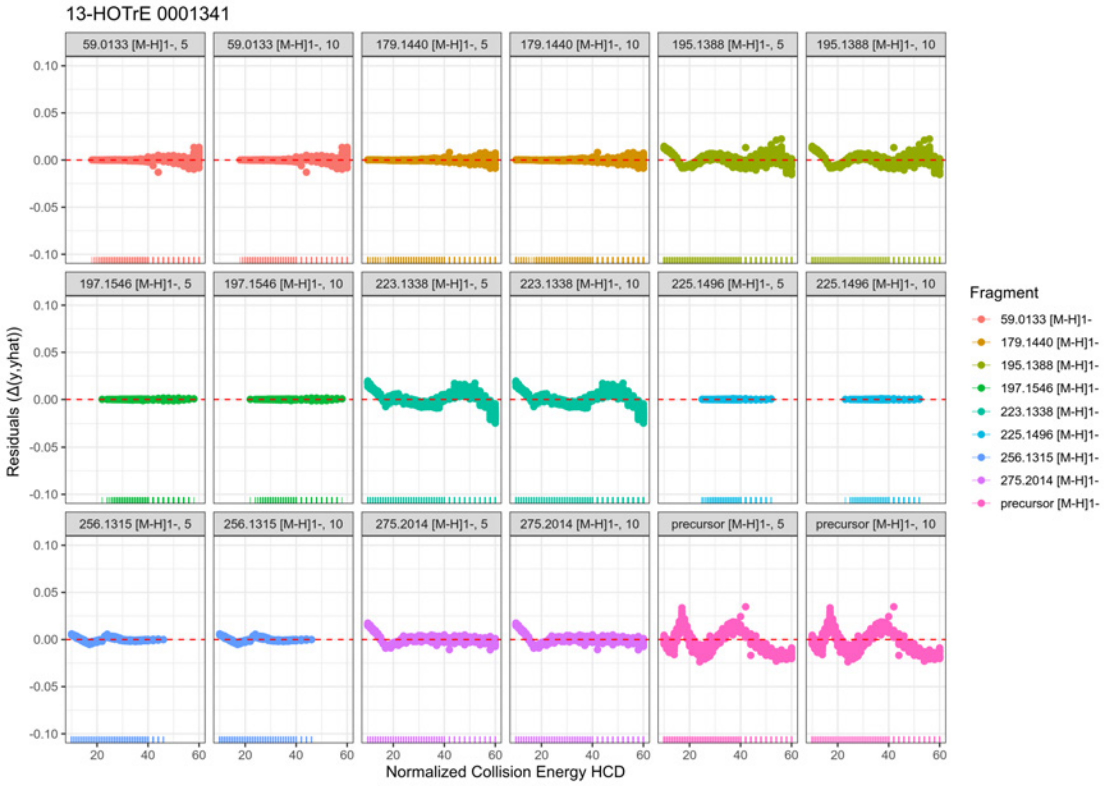

Figure 57. Residuals of nonlinear fit

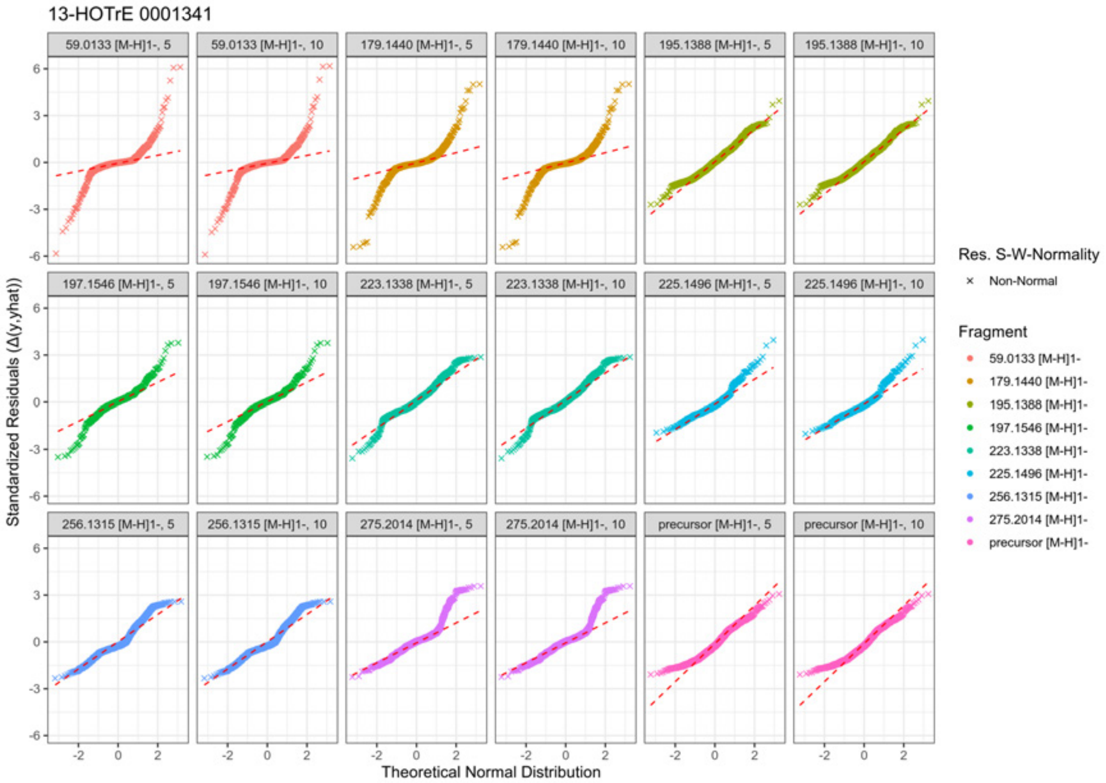

Figure 58. Quantile-quantile plot of residuals

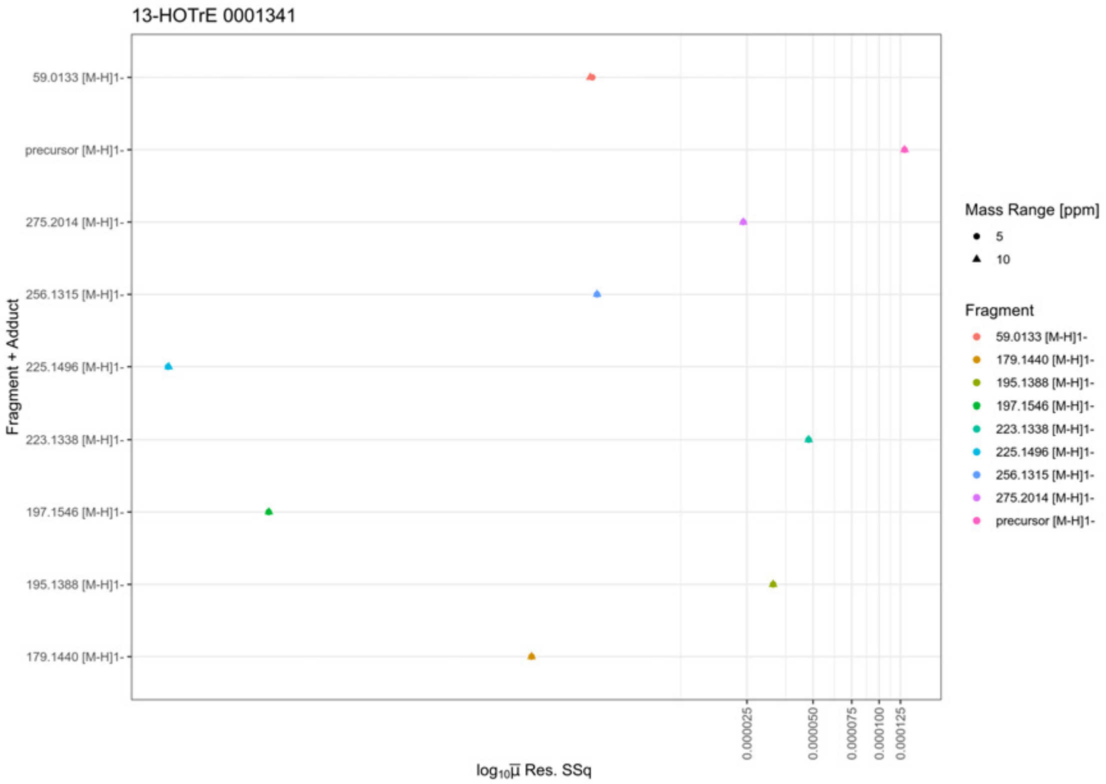

Figure 59. Normalized sum-of-squares of the residuals

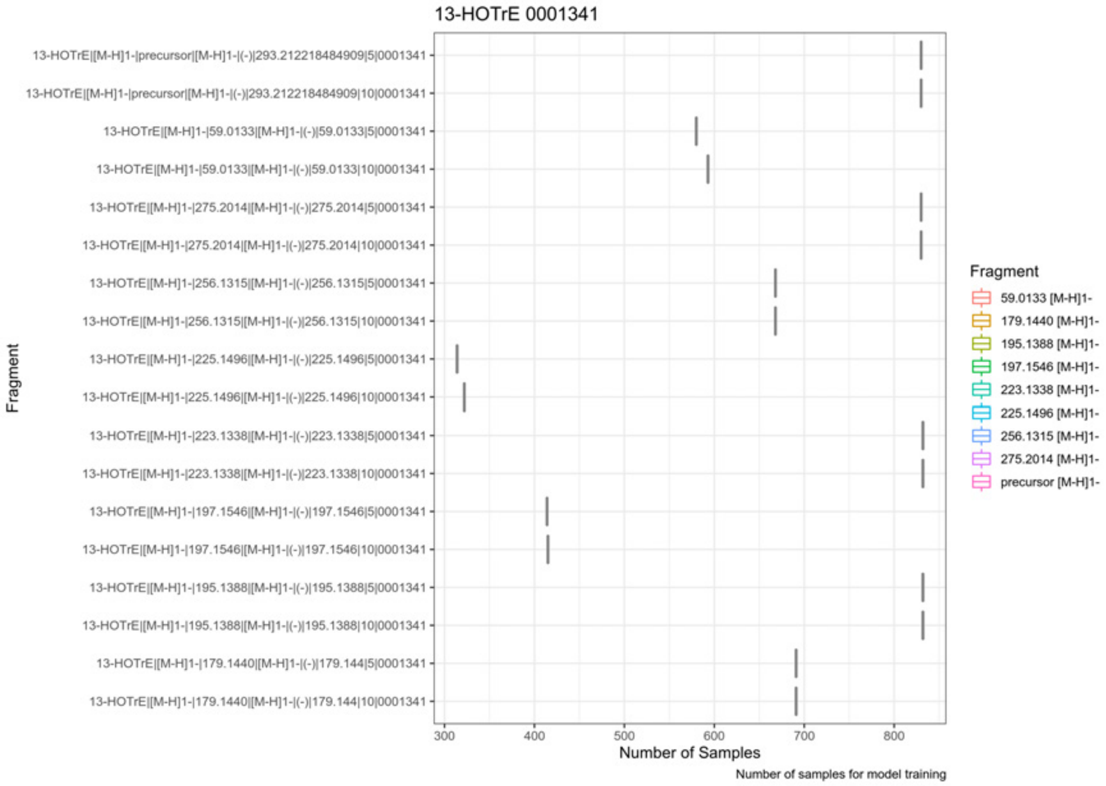

Figure 60. Number of samples used for training per combination Id

1.13. 14(15)-EET{d11} [M-H]1- 0001305

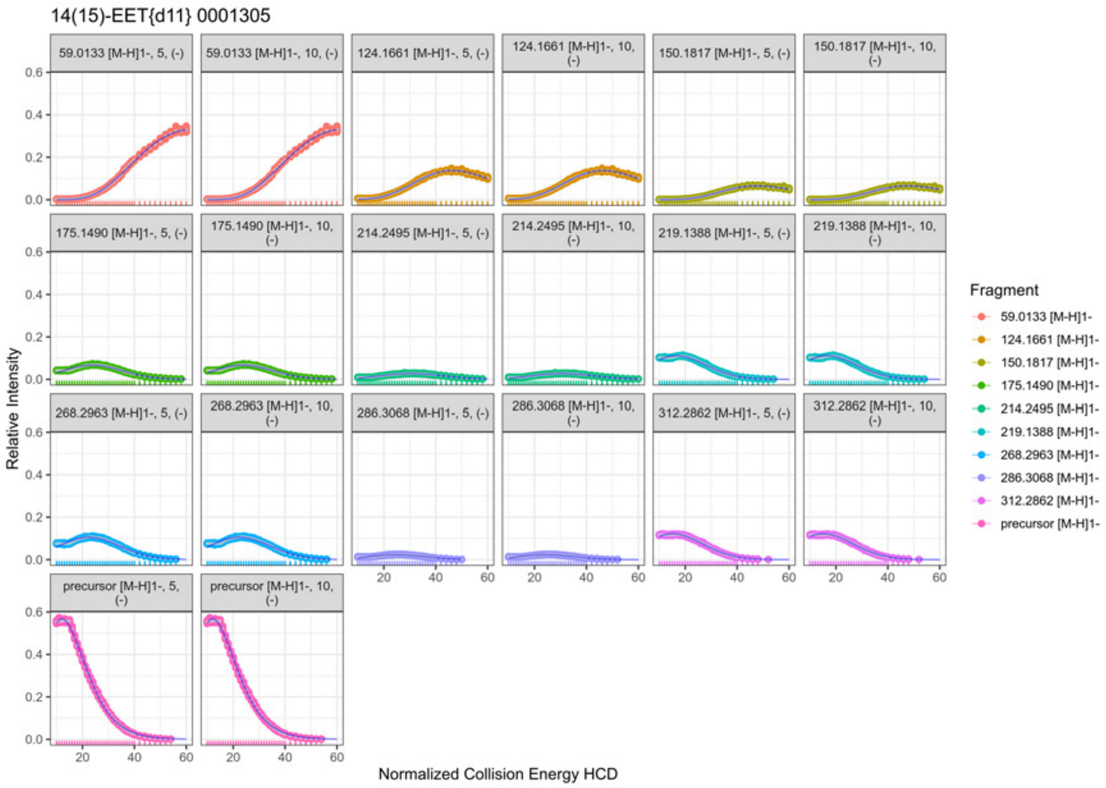

Figure 61. Nonlinear fit

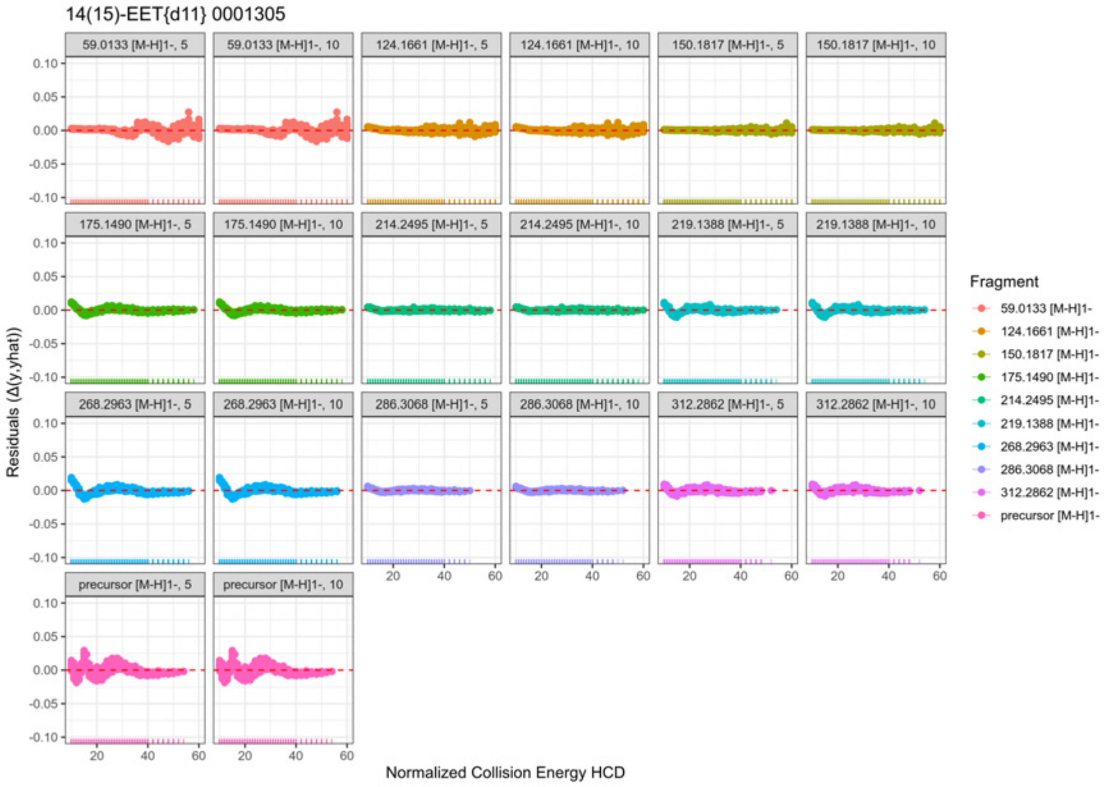

Figure 62. Residuals of nonlinear fit

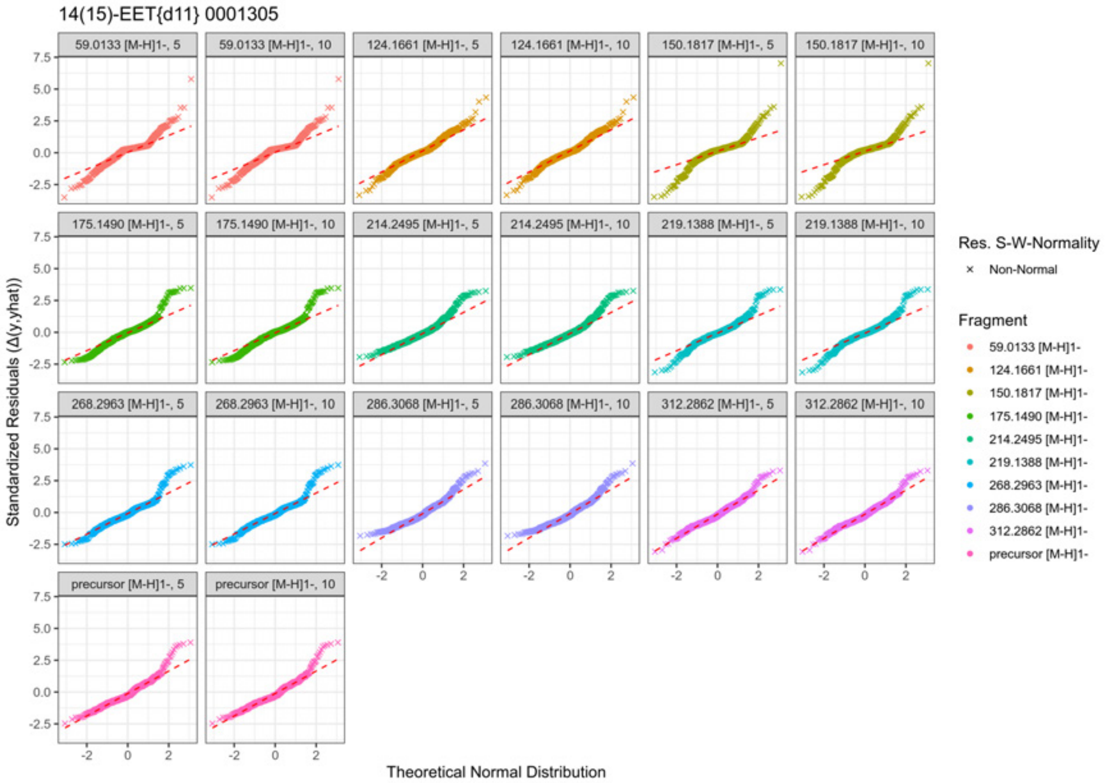

Figure 63. Quantile-quantile plot of residuals

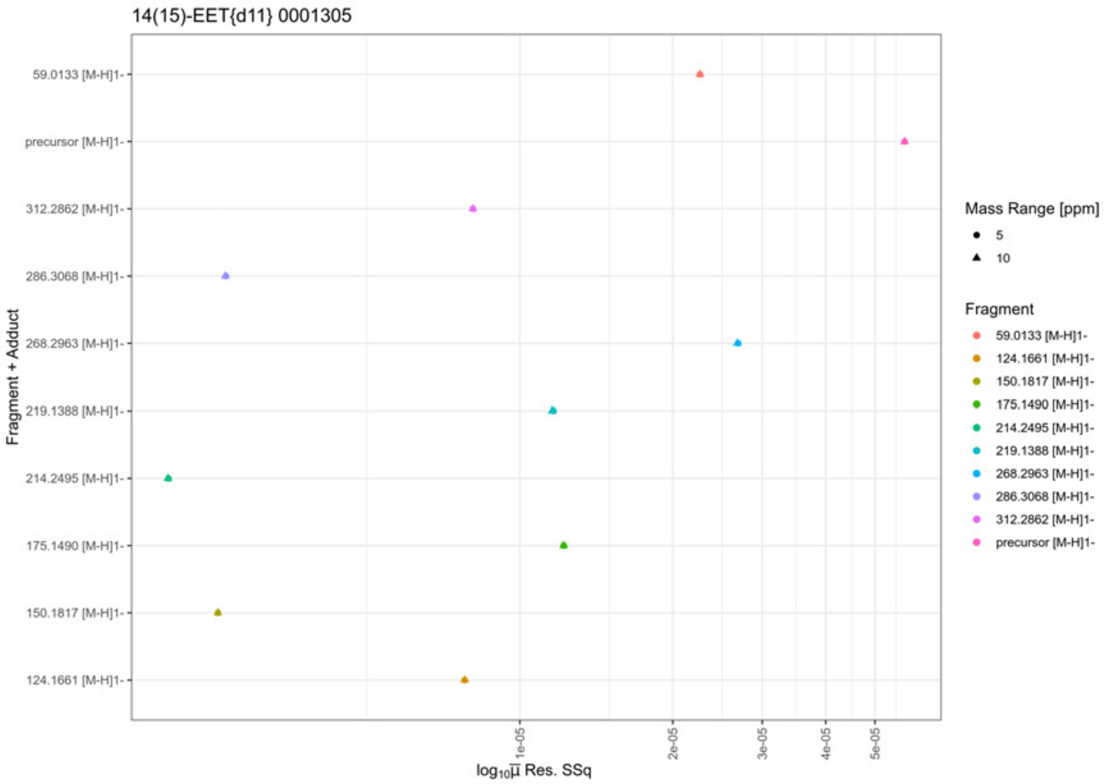

Figure 64. Normalized sum-of-squares of the residuals

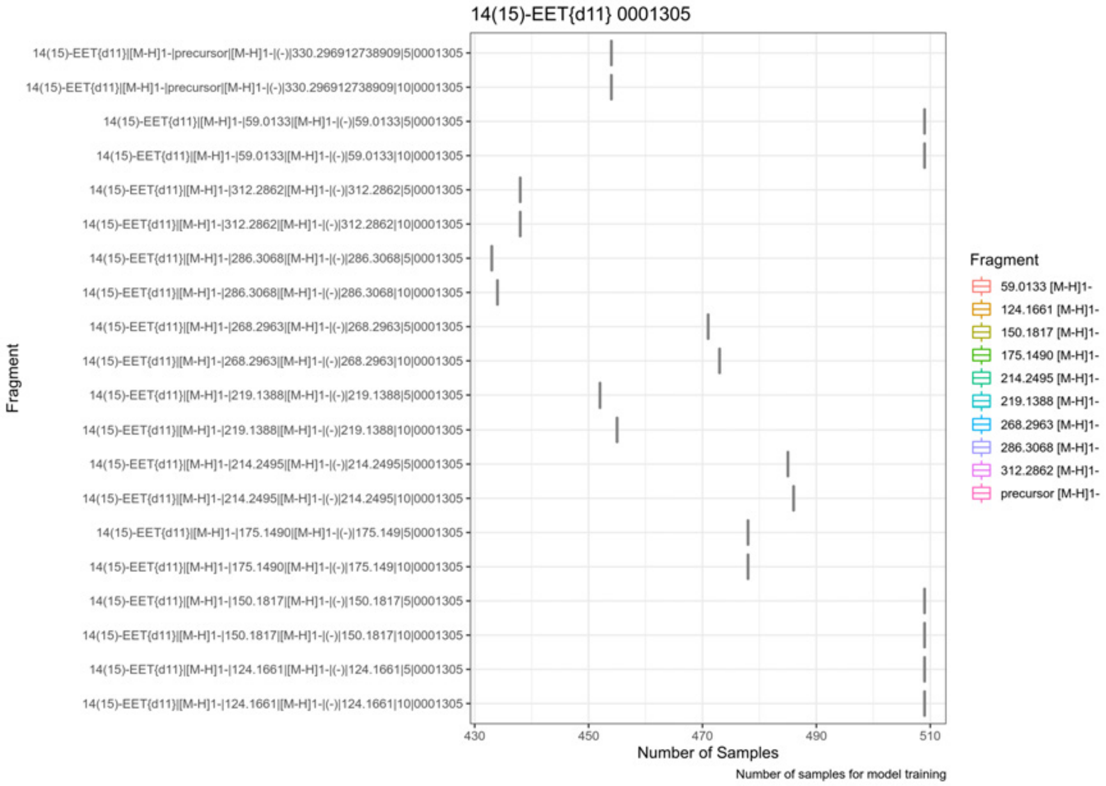

Figure 65. Number of samples used for training per combination Id

1.14. 14(15)-EpETE [M-H]1- 0001285

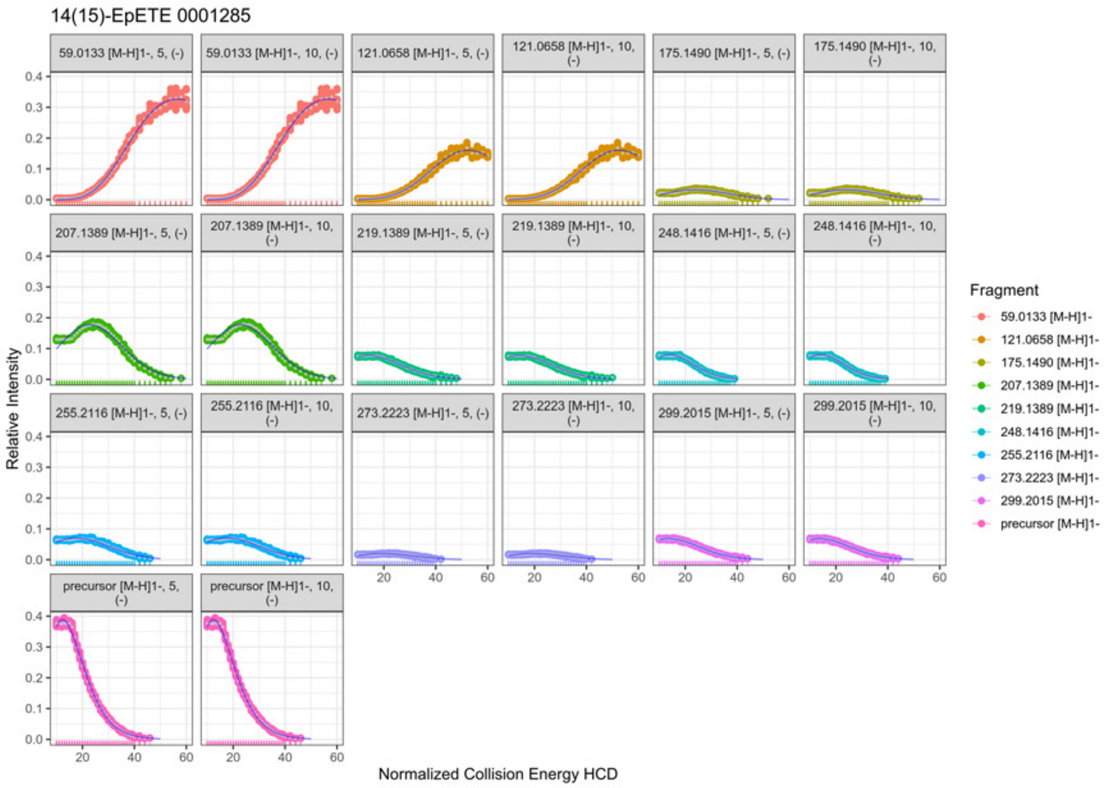

Figure 66. Nonlinear fit

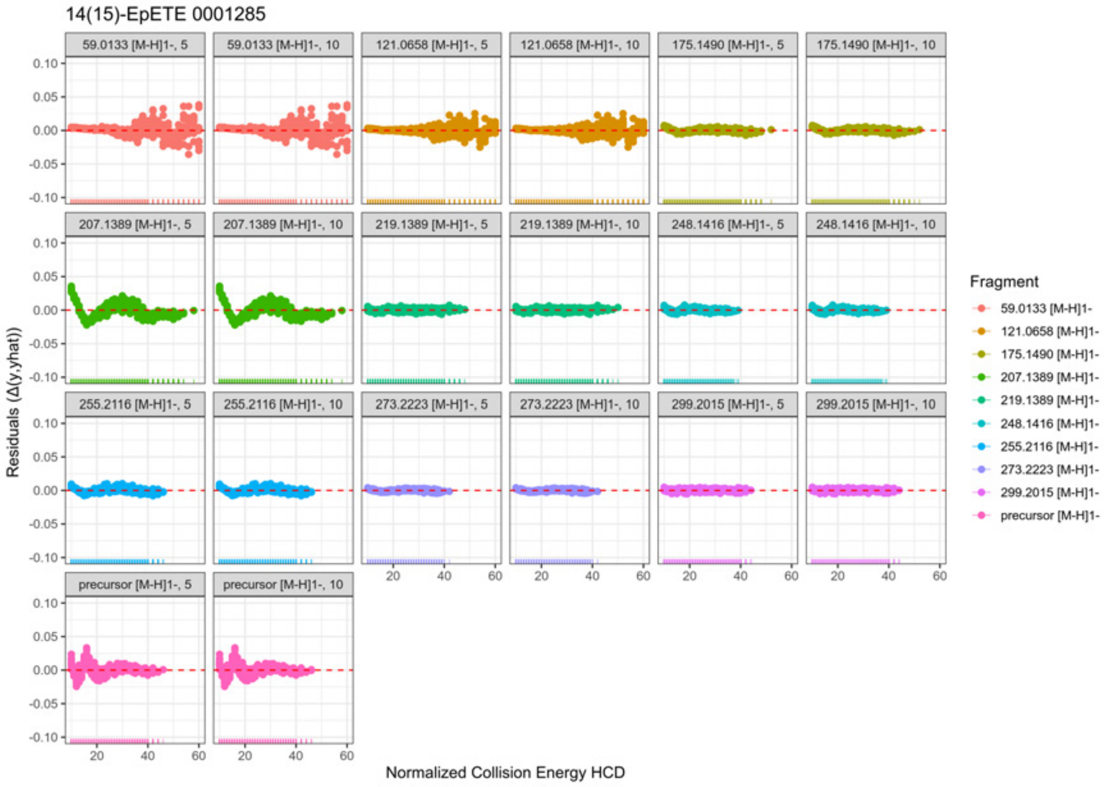

Figure 67. Residuals of nonlinear fit

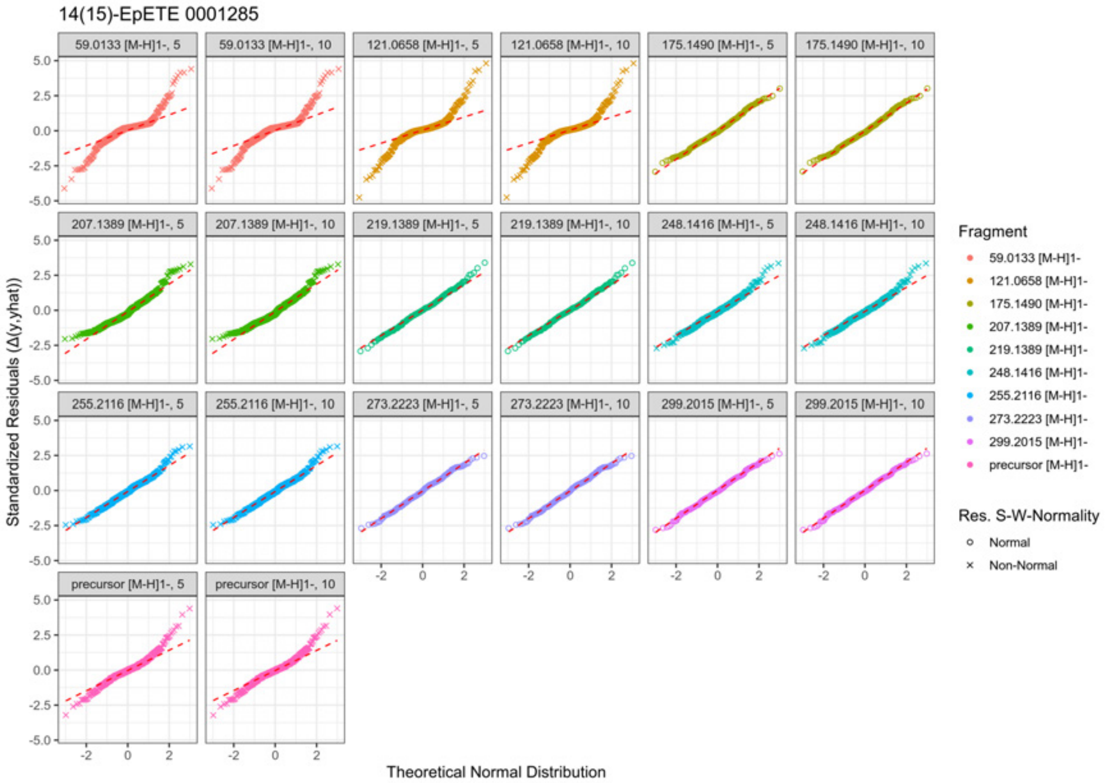

Figure 68. Quantile-quantile plot of residuals

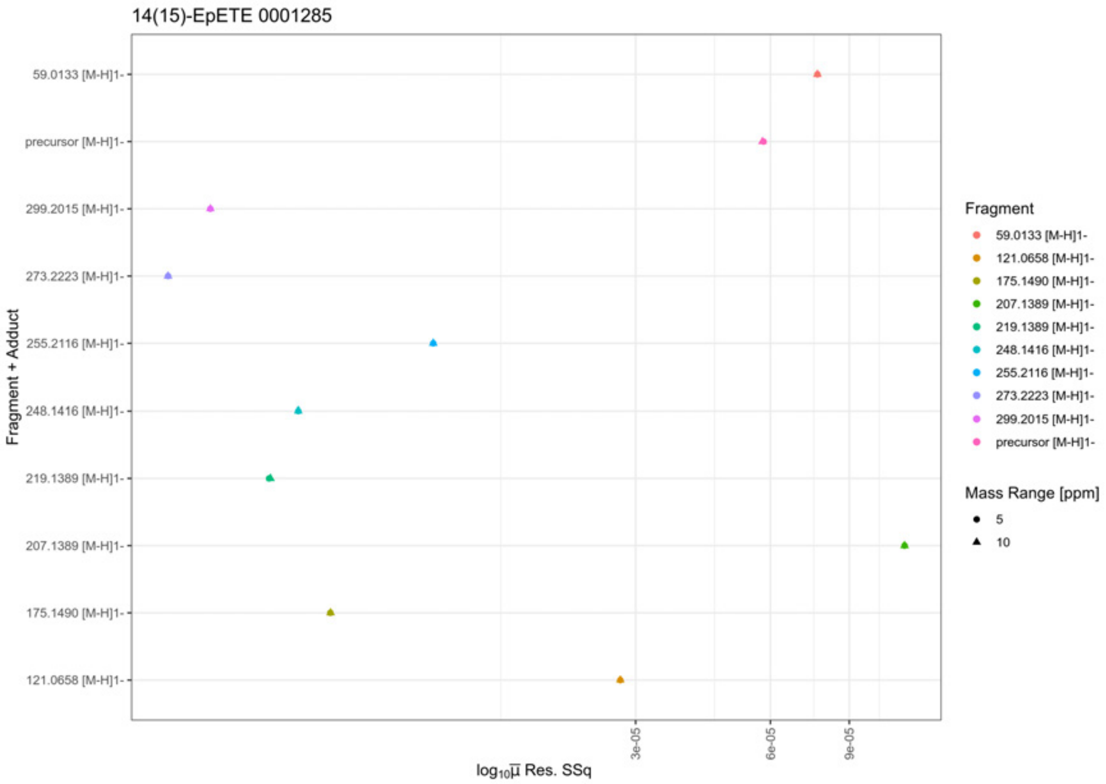

Figure 69. Normalized sum-of-squares of the residuals

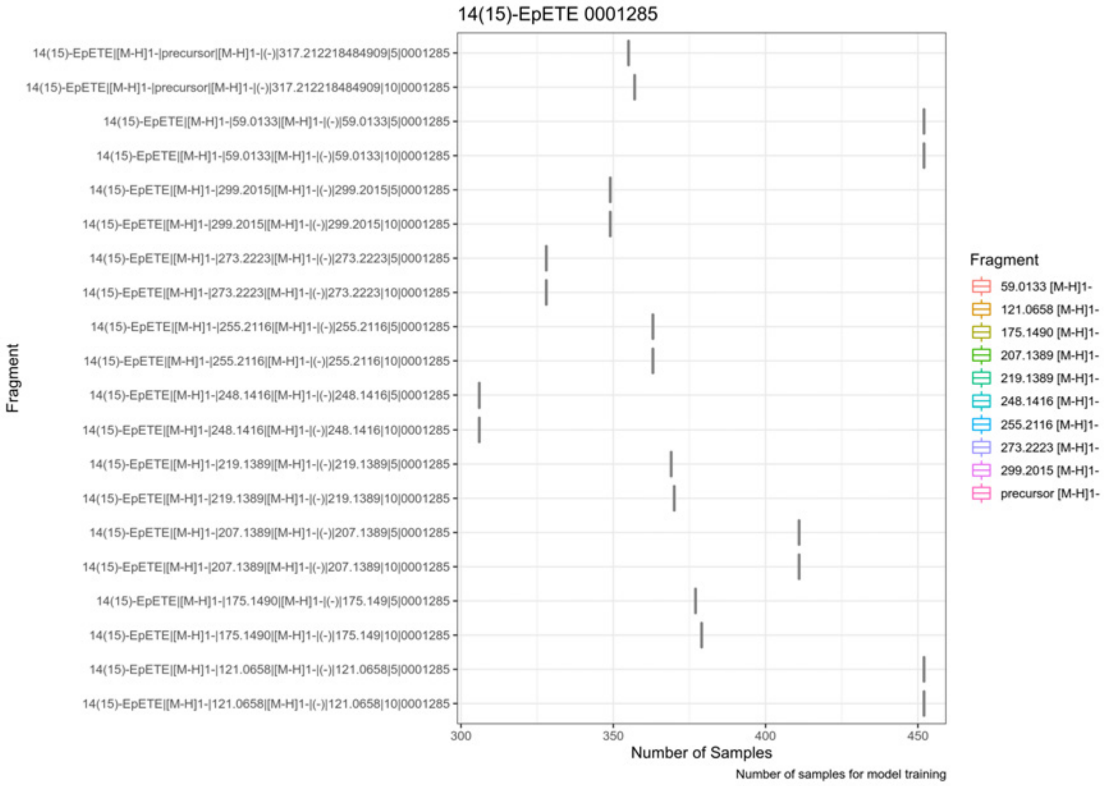

Figure 70. Number of samples used for training per combination Id

1.15. 14,15-DHET{d11} [M-H]1- 0000155

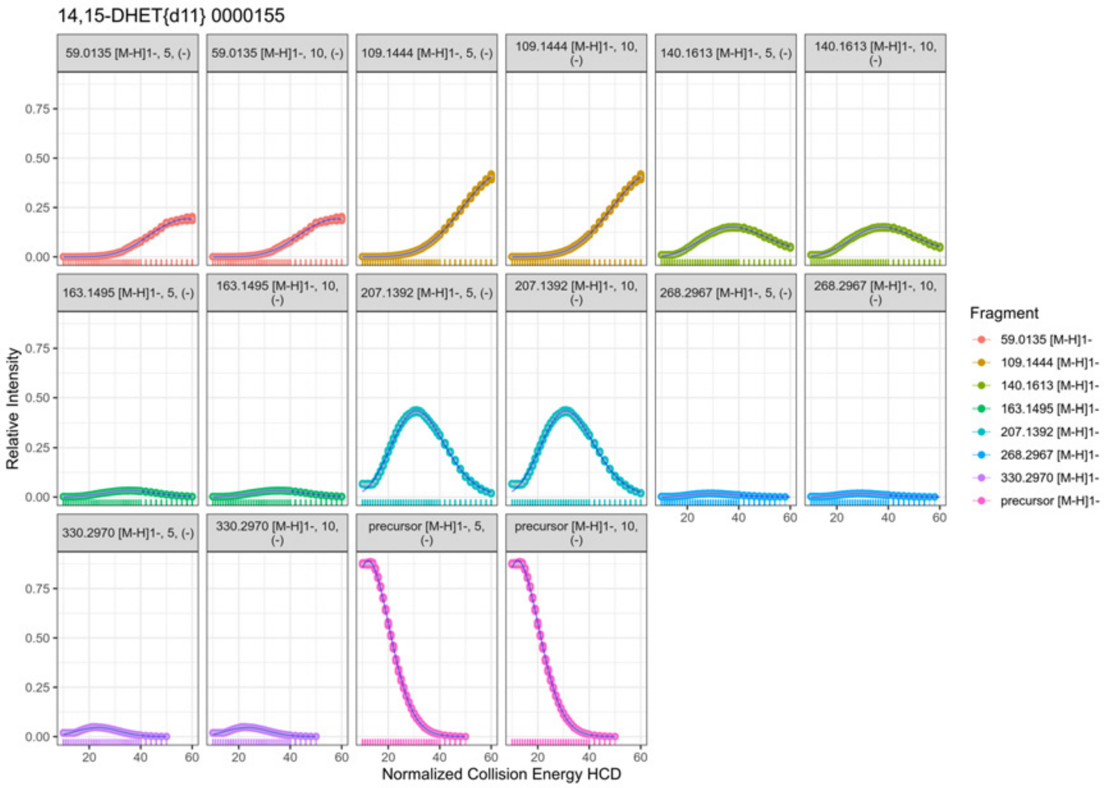

Figure 71. Nonlinear fit

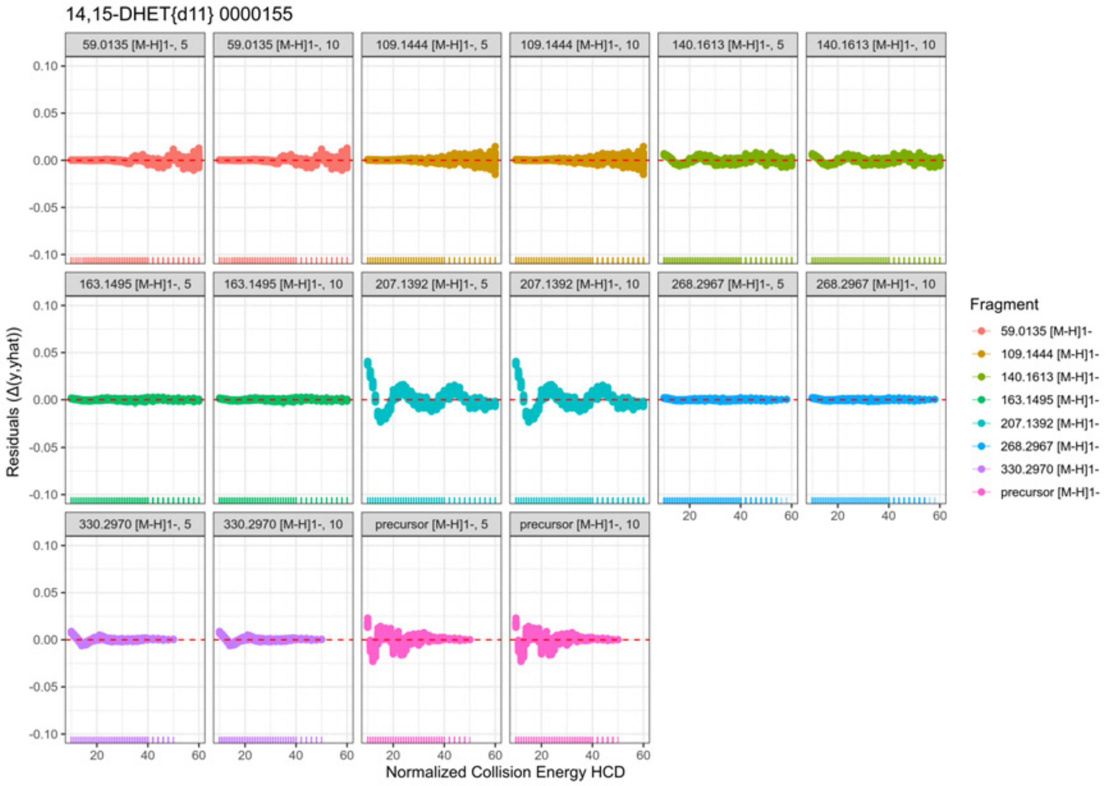

Figure 72. Residuals of nonlinear fit

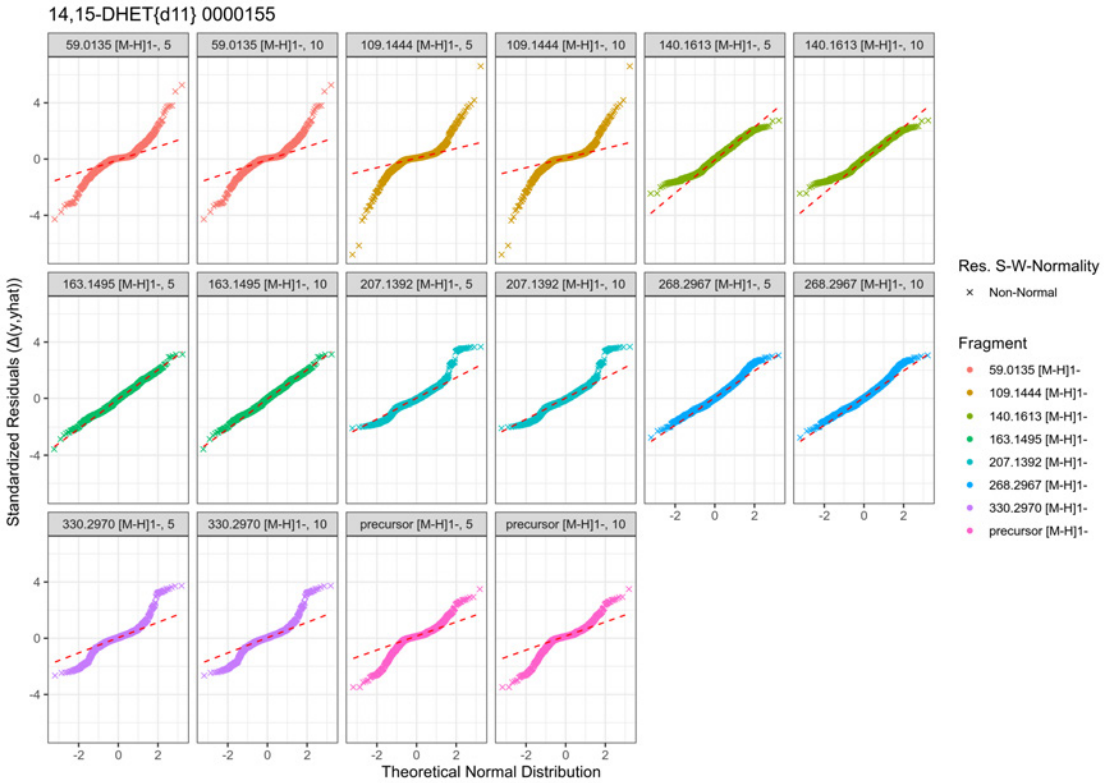

Figure 73. Quantile-quantile plot of residuals

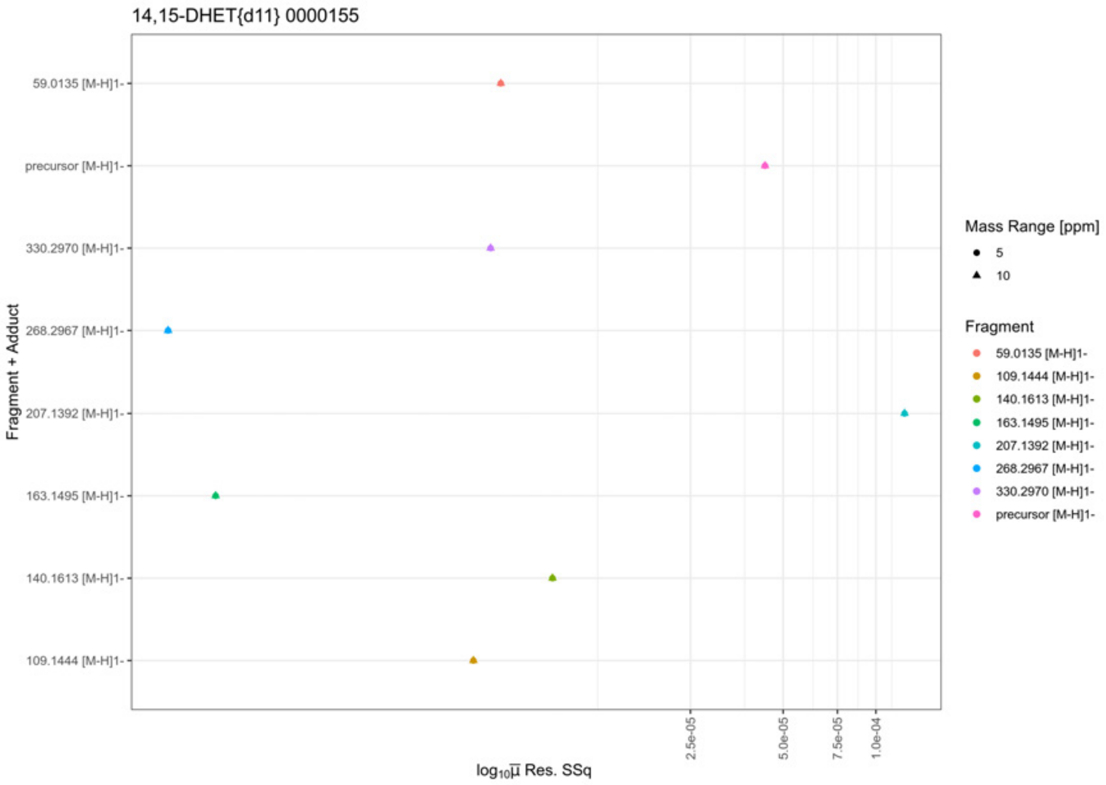

Figure 74. Normalized sum-of-squares of the residuals

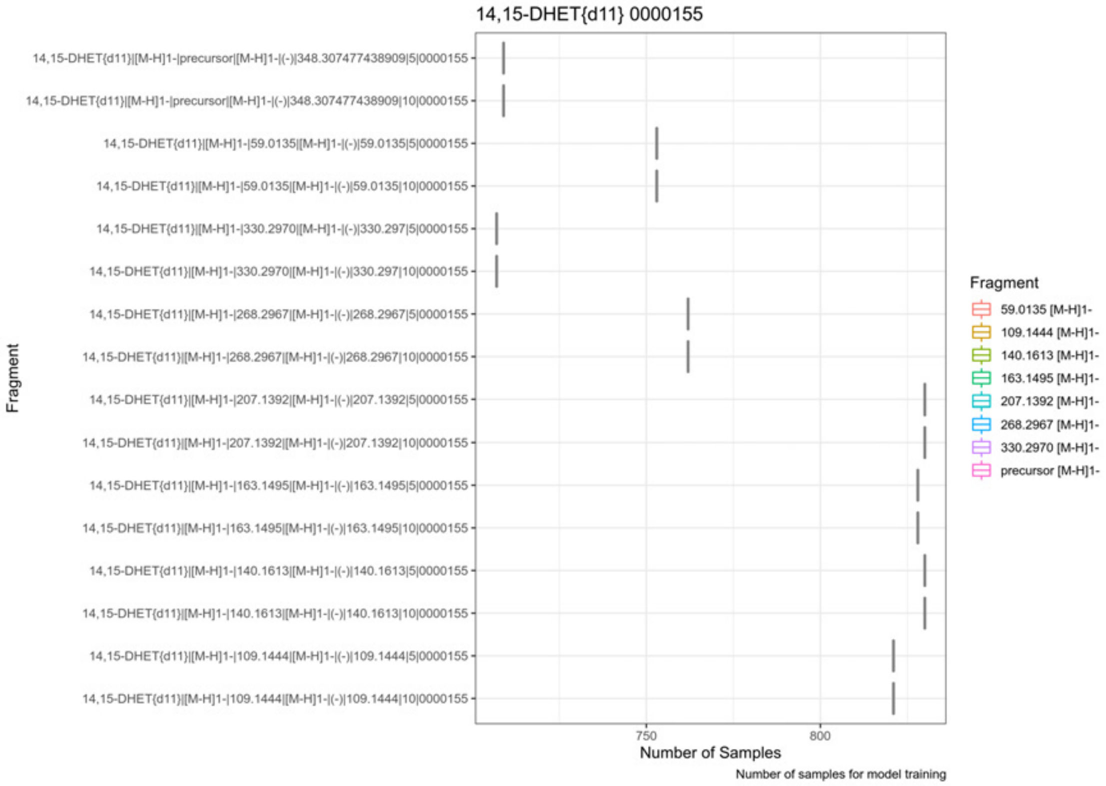

Figure 75. Number of samples used for training per combination Id

1.16. 15-HEPE [M-H]1- 0000139

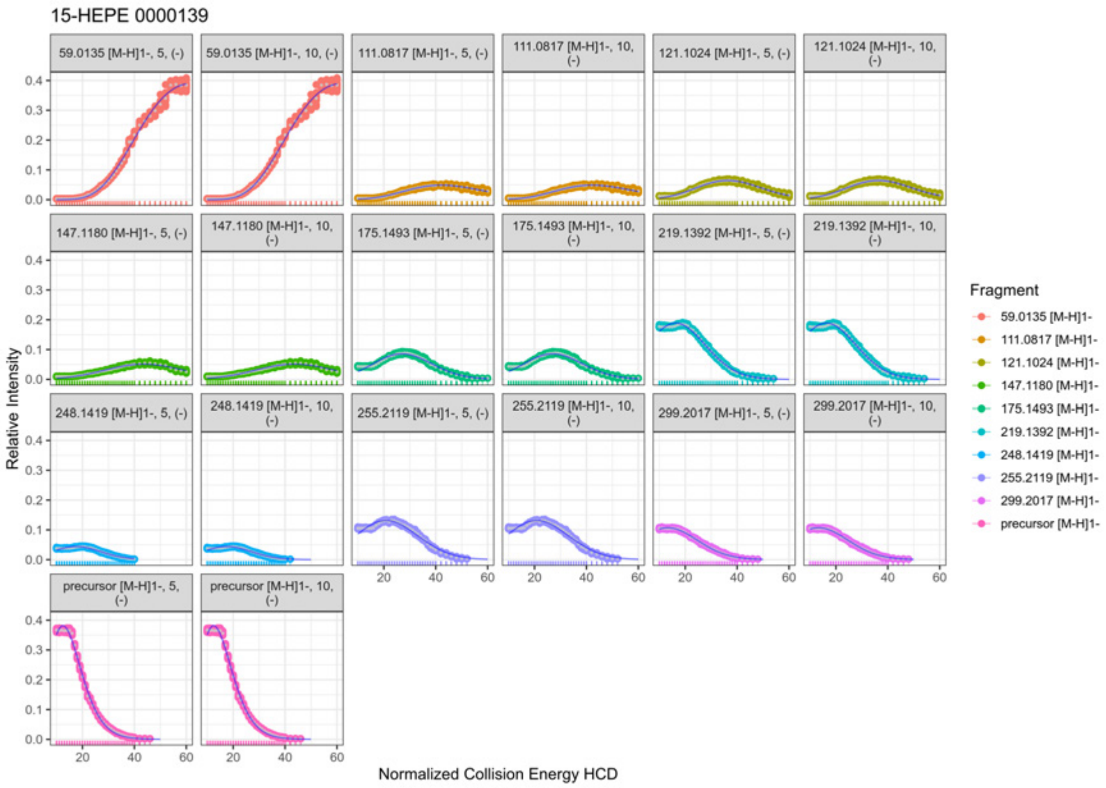

Figure 76. Nonlinear fit

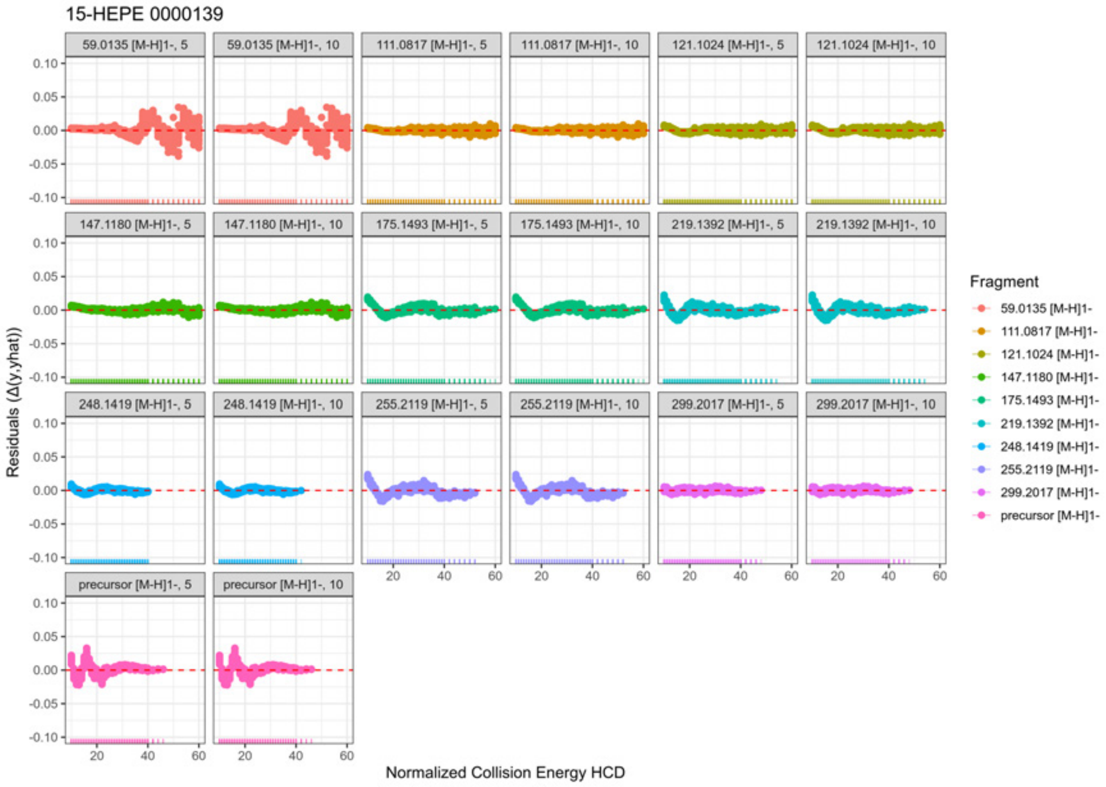

Figure 77. Residuals of nonlinear fit

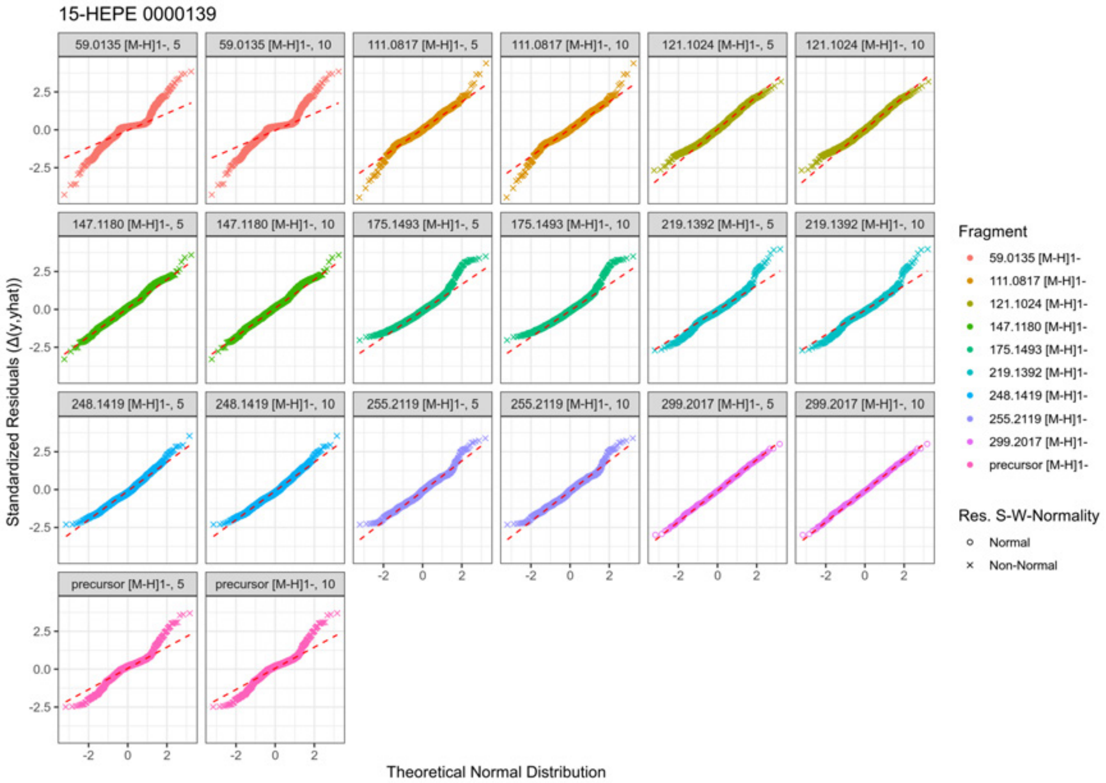

Figure 78. Quantile-quantile plot of residuals

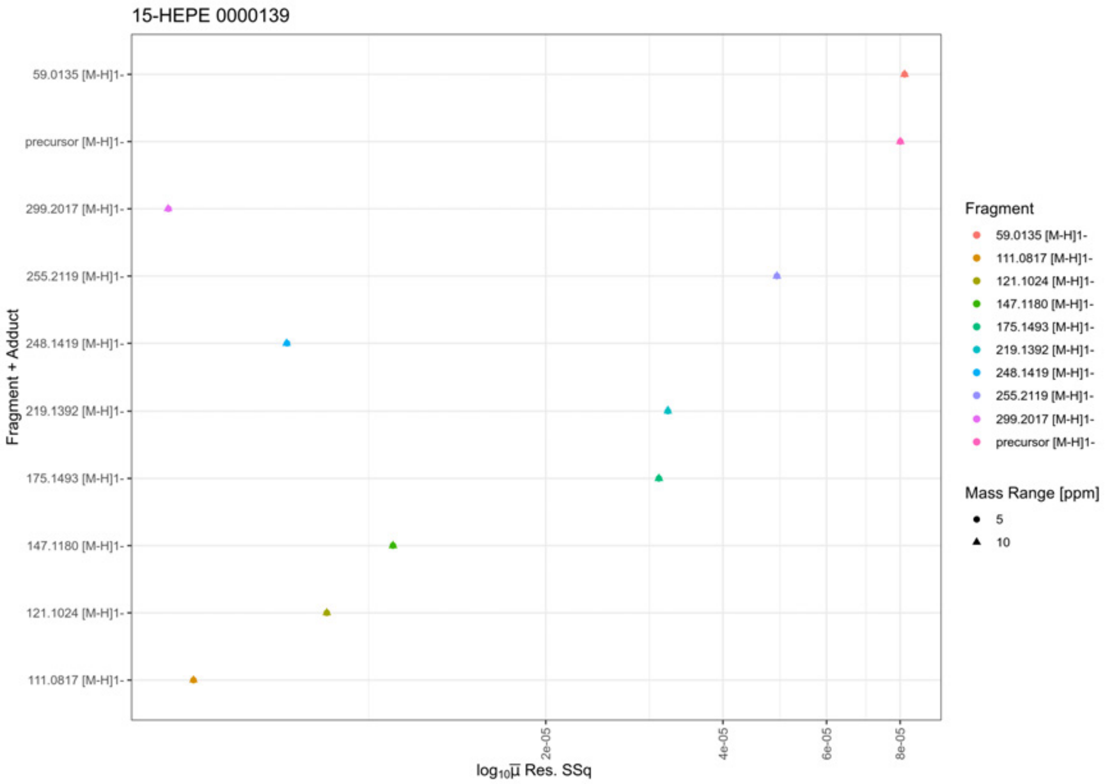

Figure 79. Normalized sum-of-squares of the residuals

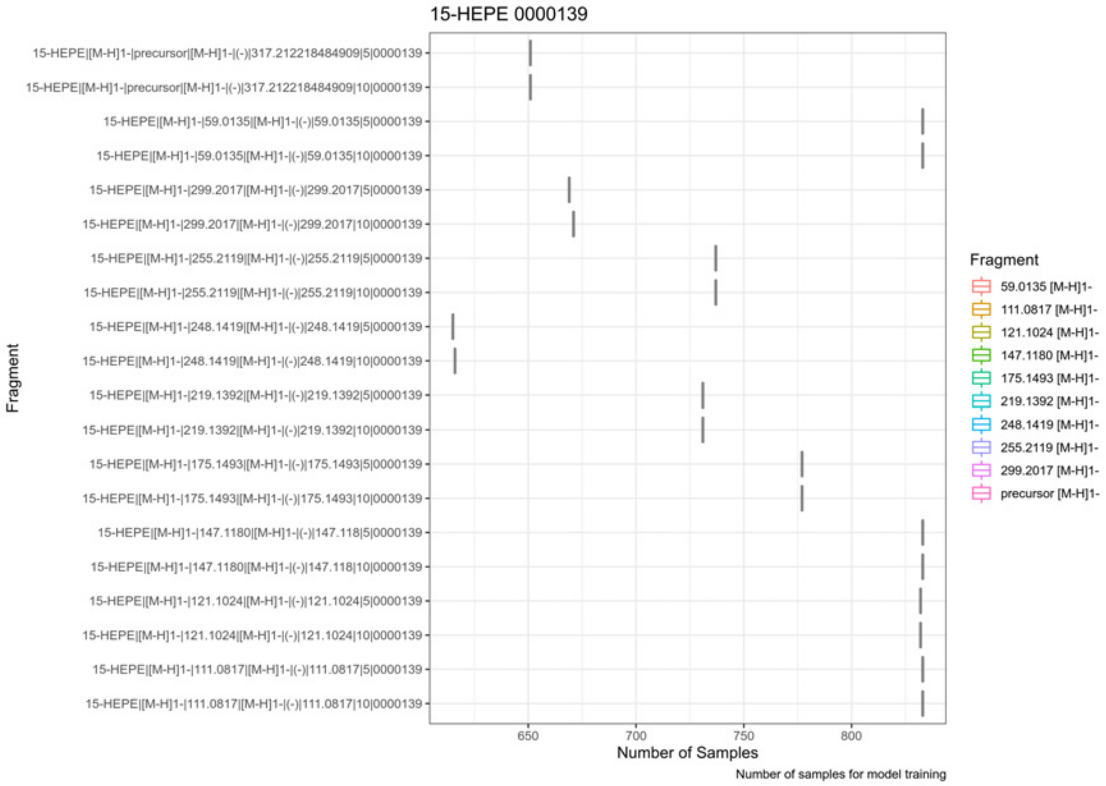

Figure 80. Number of samples used for training per combination Id

1.17. 15-HETE{d8} [M-H]1- 0001329

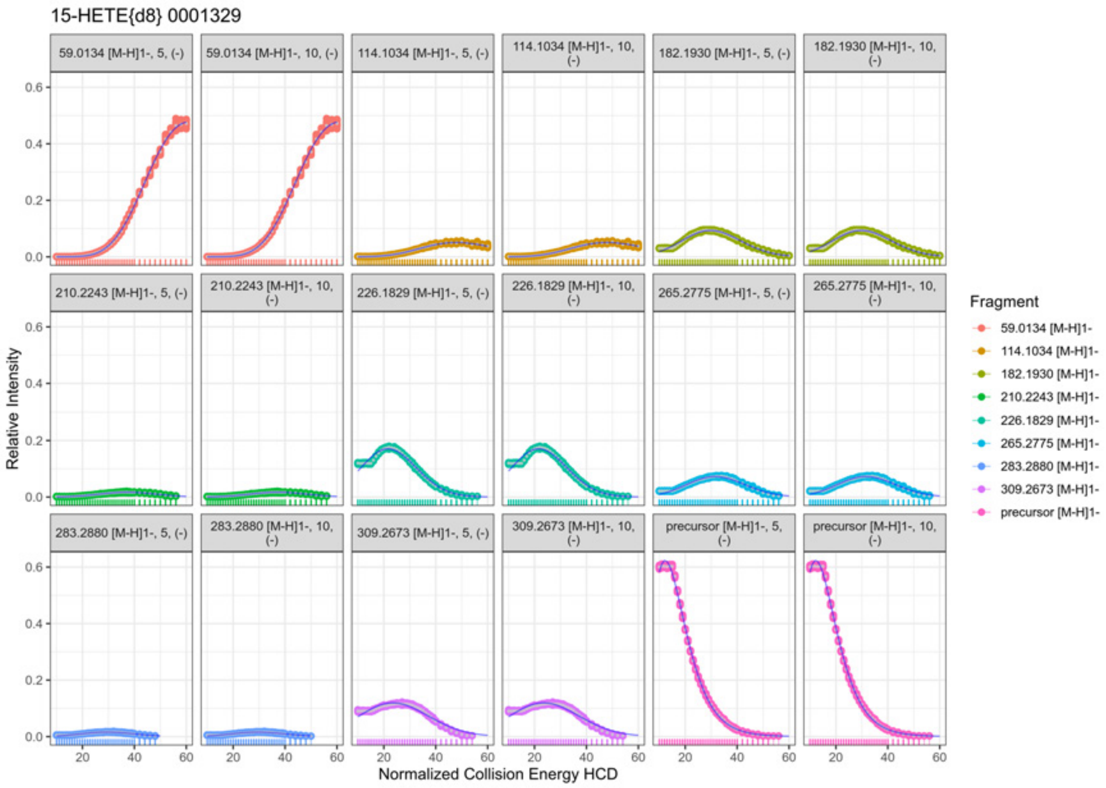

Figure 81. Nonlinear fit

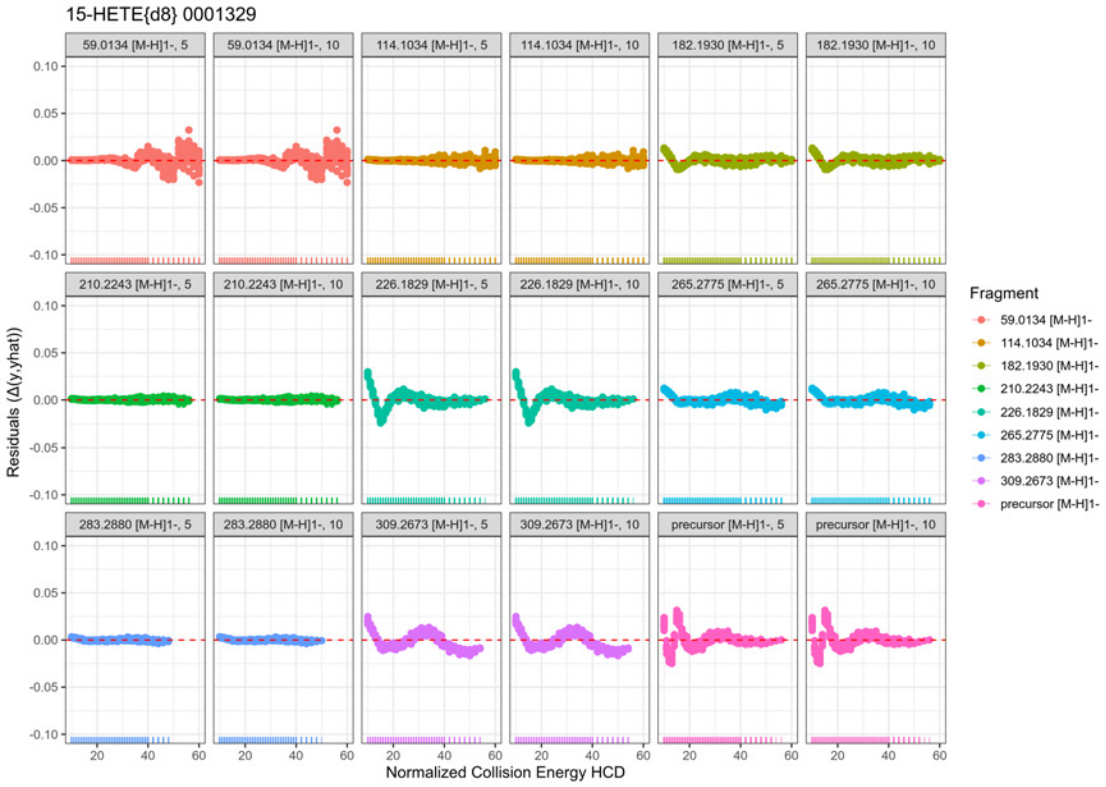

Figure 82. Residuals of nonlinear fit

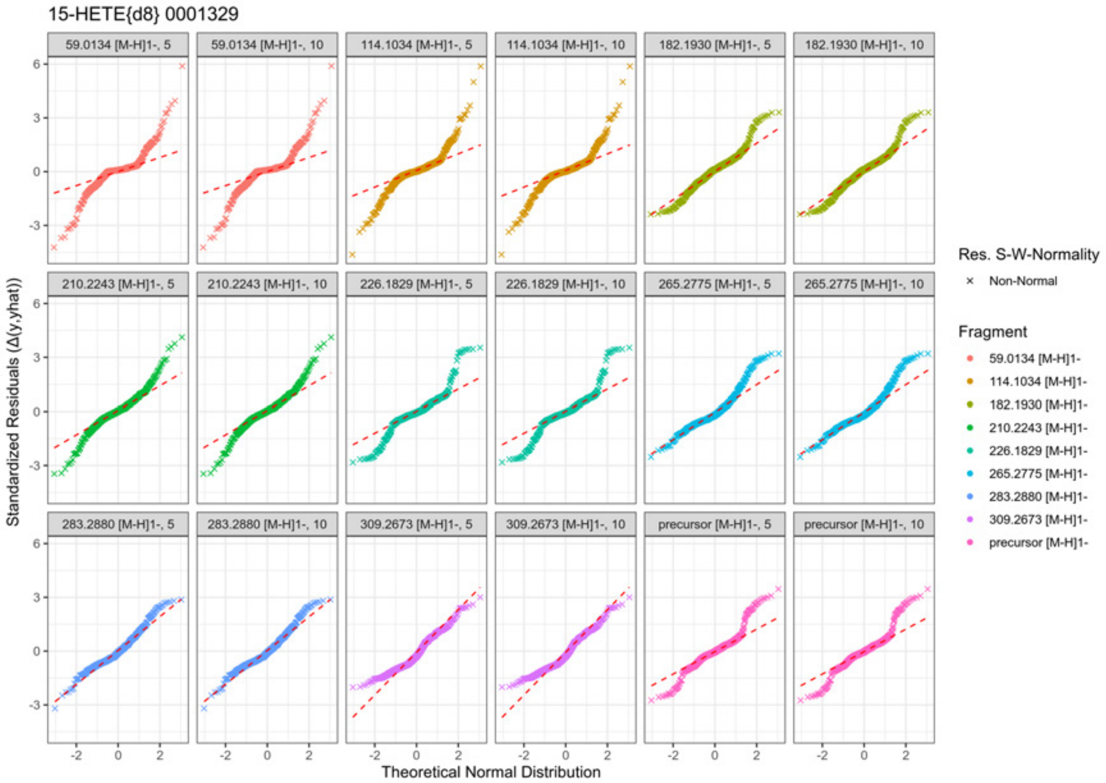

Figure 83. Quantile-quantile plot of residuals

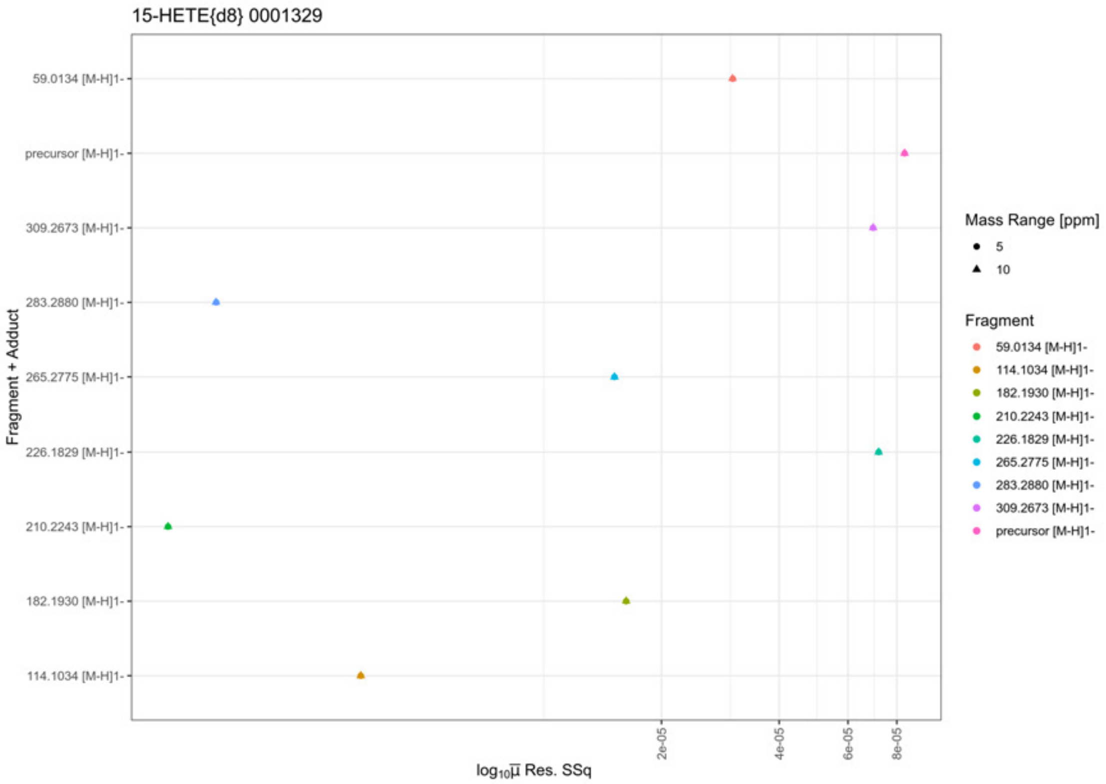

Figure 84. Normalized sum-of-squares of the residuals

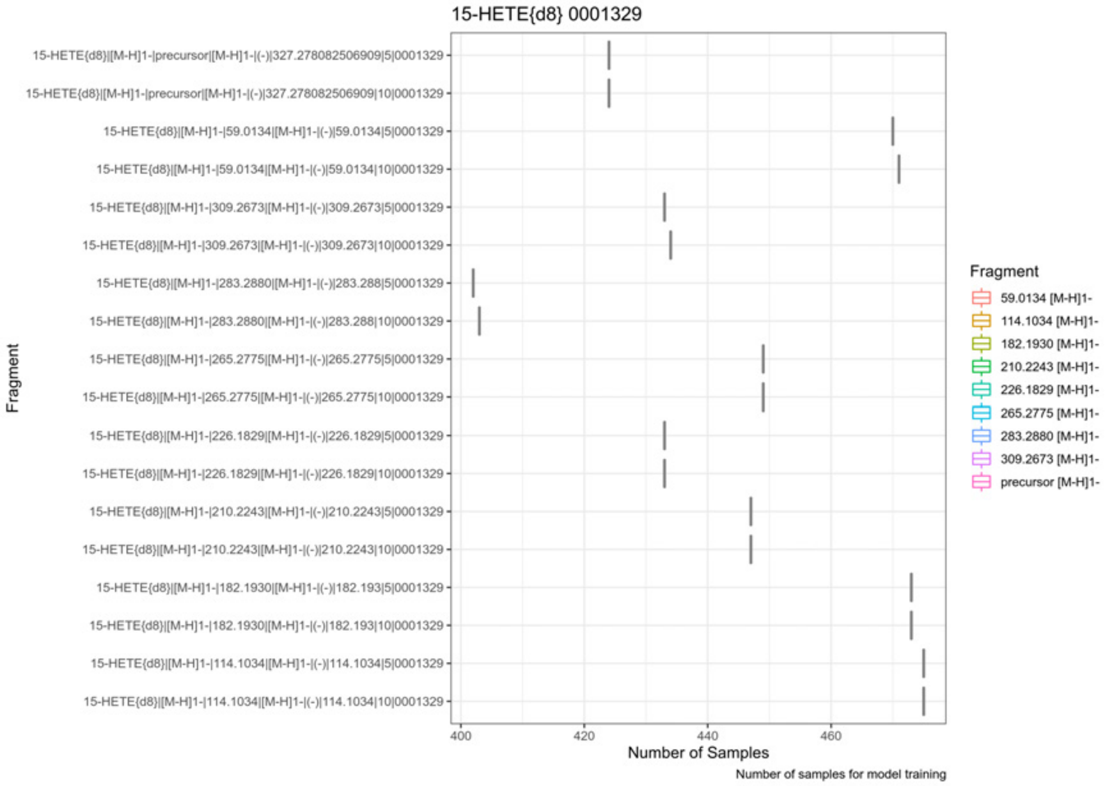

Figure 85. Number of samples used for training per combination Id

1.18. 15d-PGJ2{d4} [M-H]1- 0000149

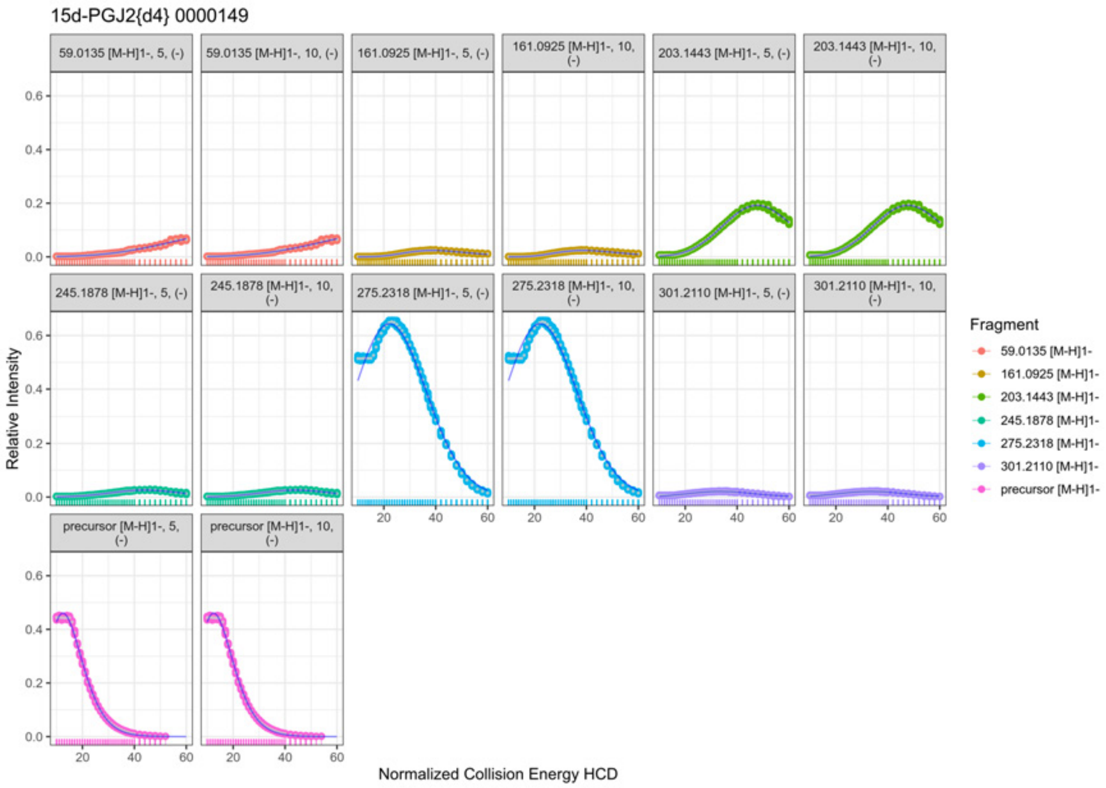

Figure 86. Nonlinear fit

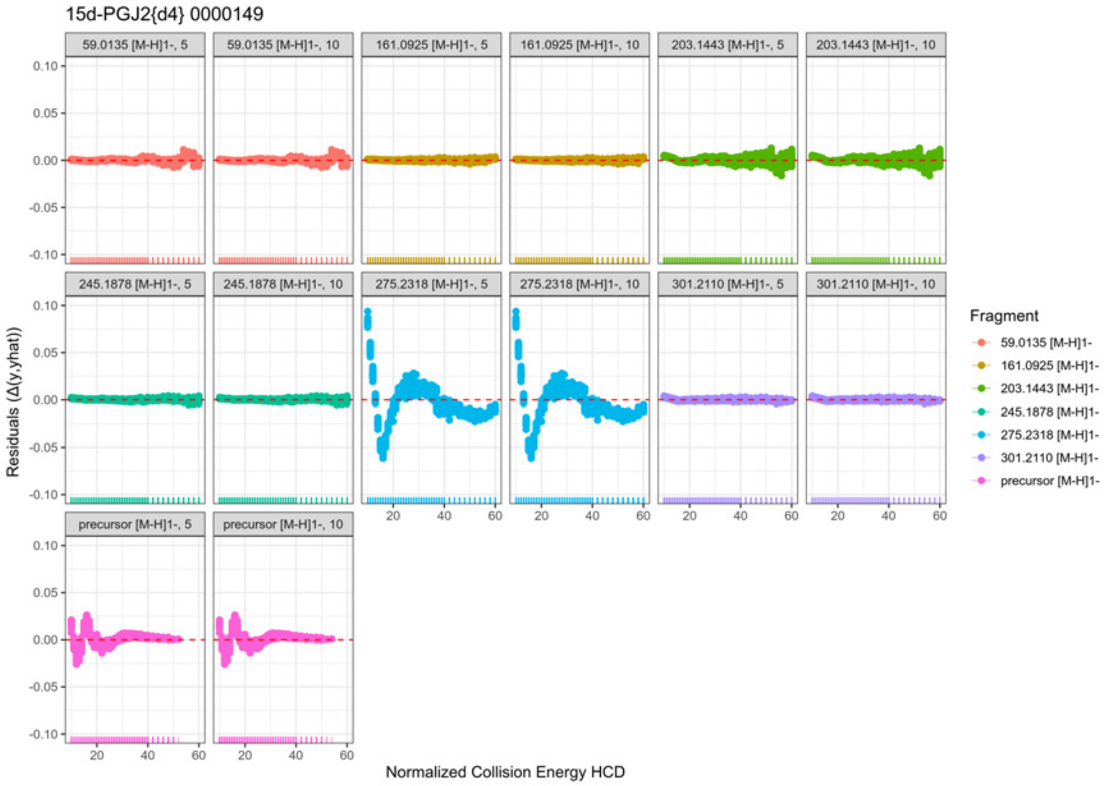

Figure 87. Residuals of nonlinear fit

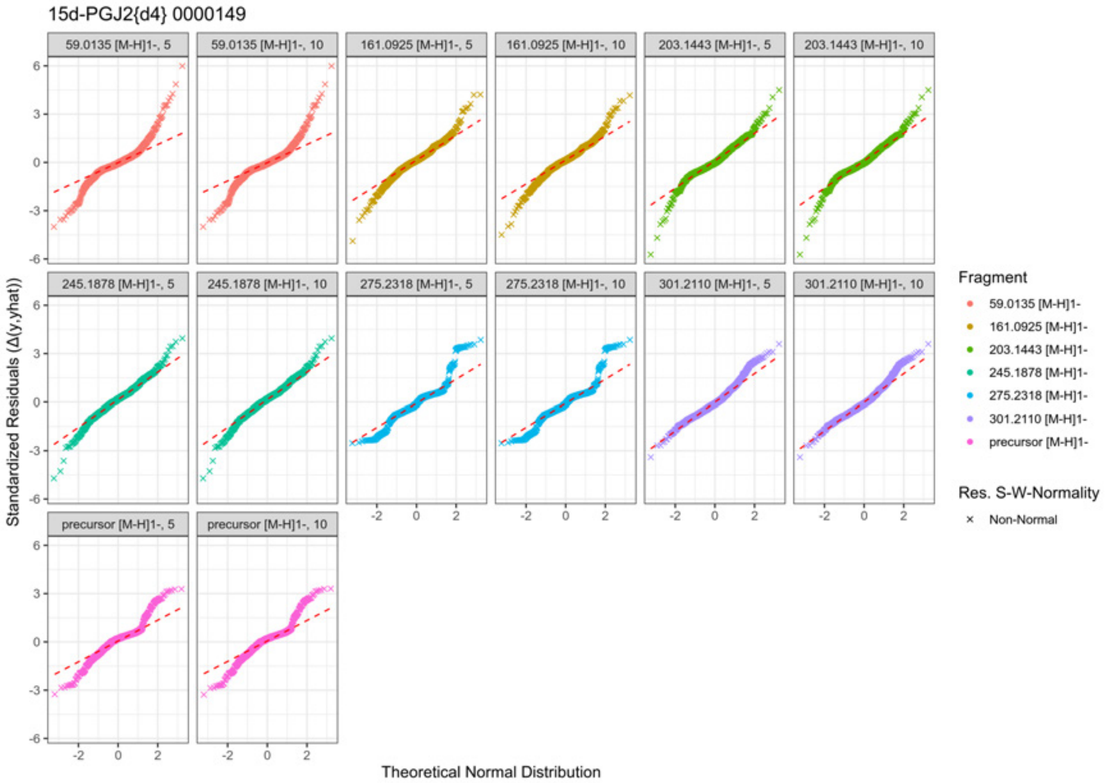

Figure 88. Quantile-quantile plot of residuals

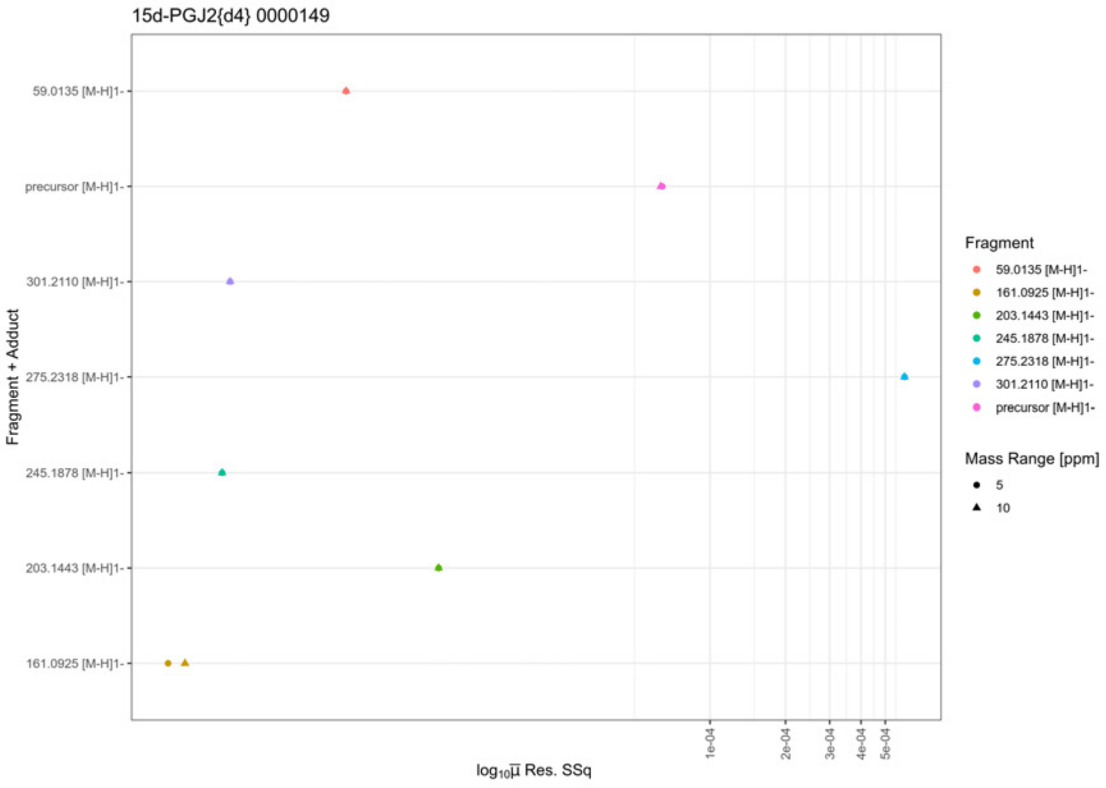

Figure 89. Normalized sum-of-squares of the residuals

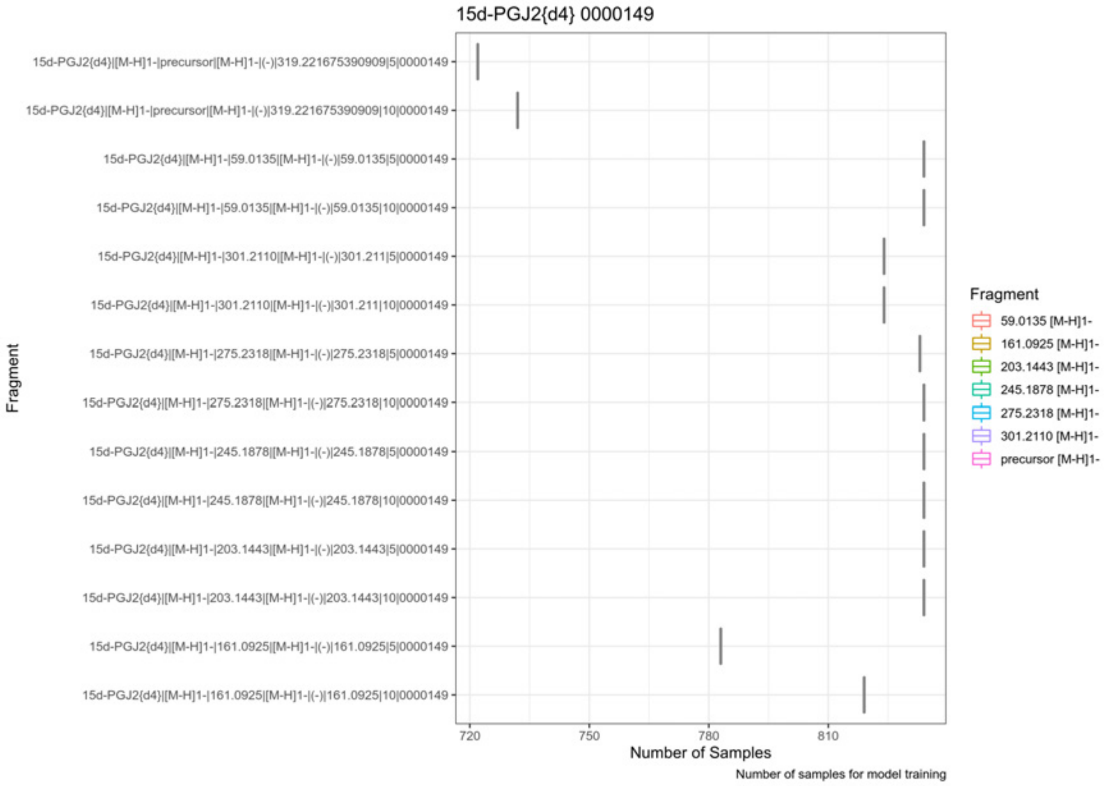

Figure 90. Number of samples used for training per combination Id

1.19. 16-HDoHE [M-H]1- 0001271

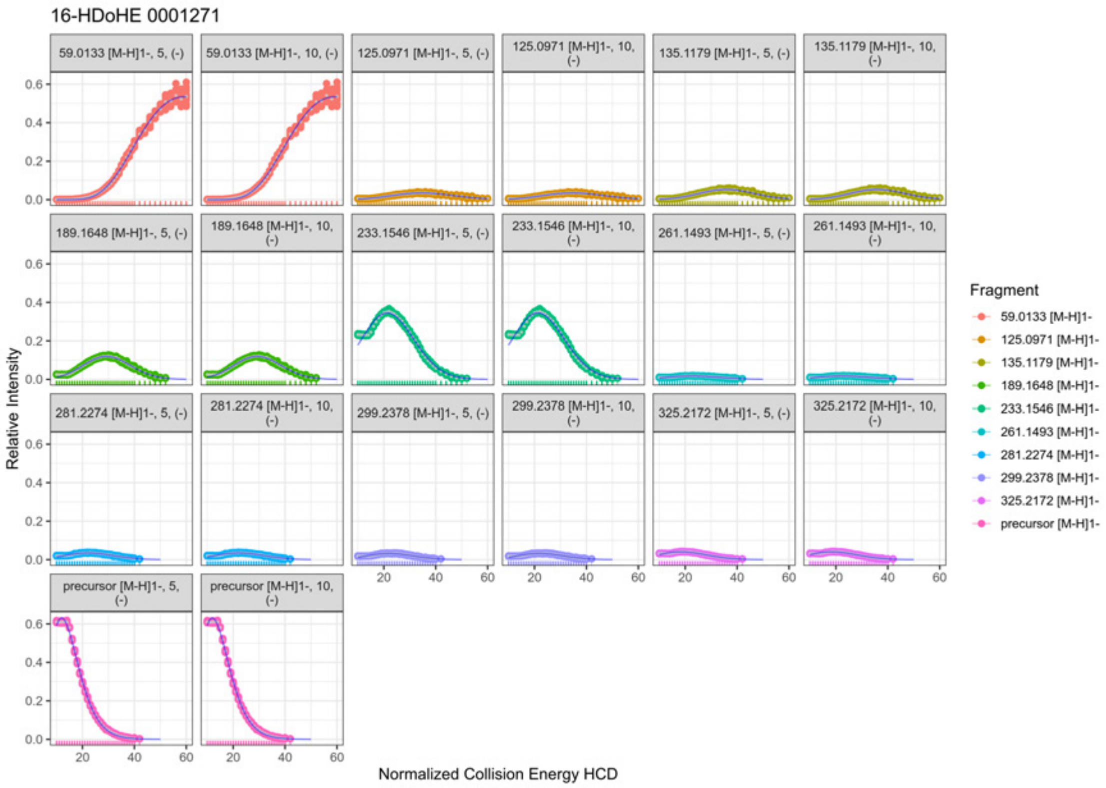

Figure 91. Nonlinear fit

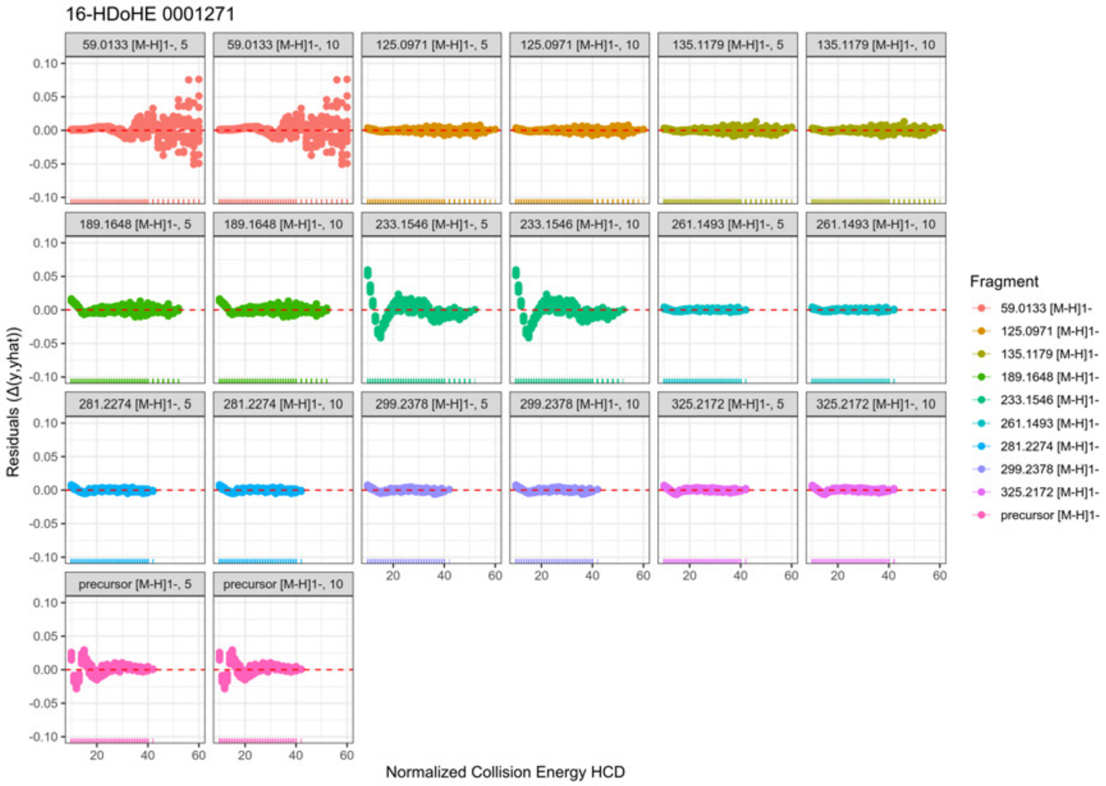

Figure 92. Residuals of nonlinear fit

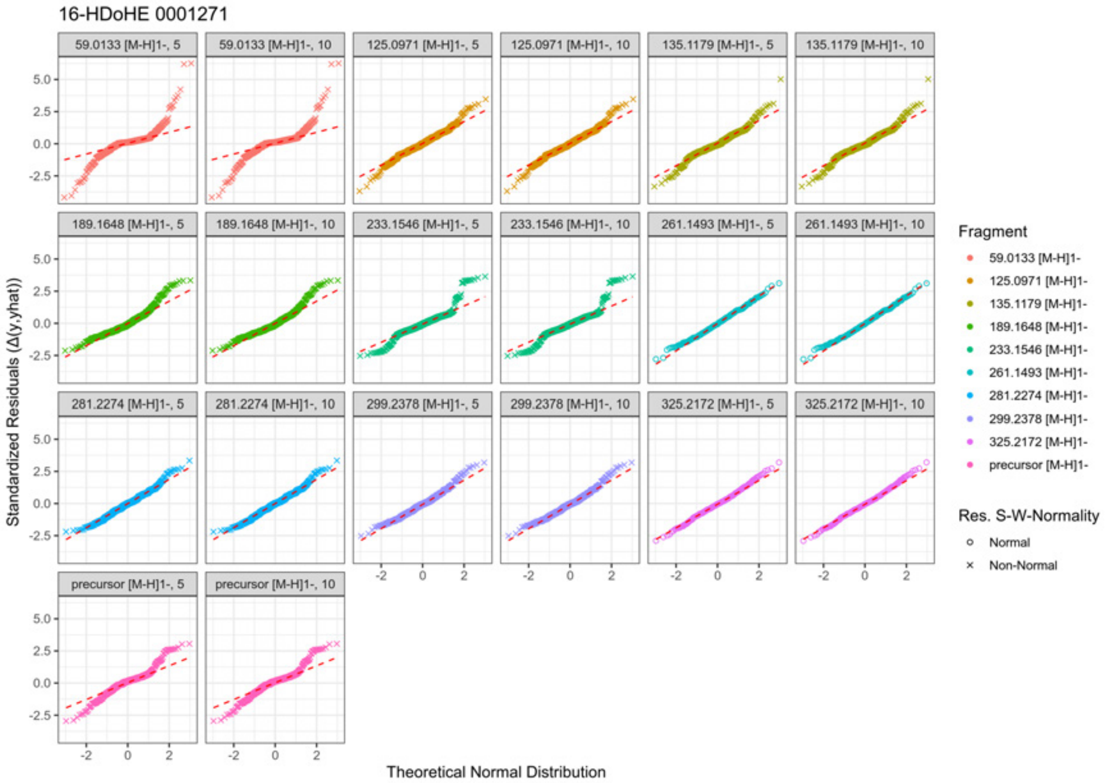

Figure 93. Quantile-quantile plot of residuals

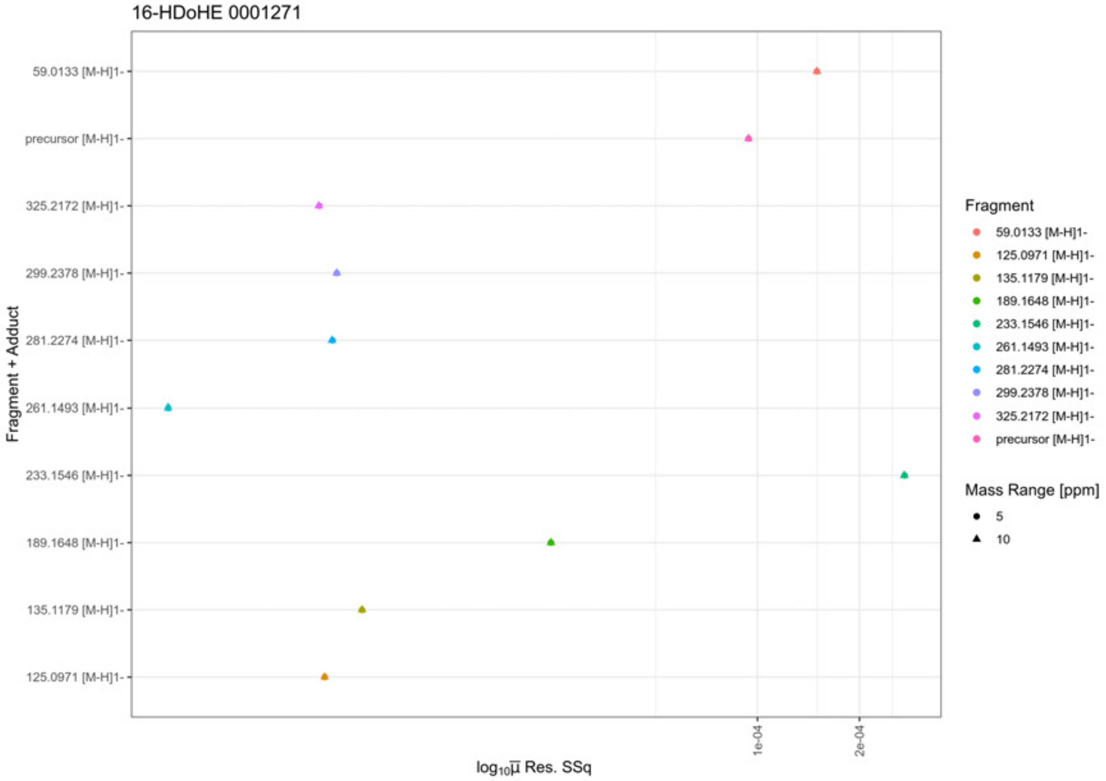

Figure 94. Normalized sum-of-squares of the residuals

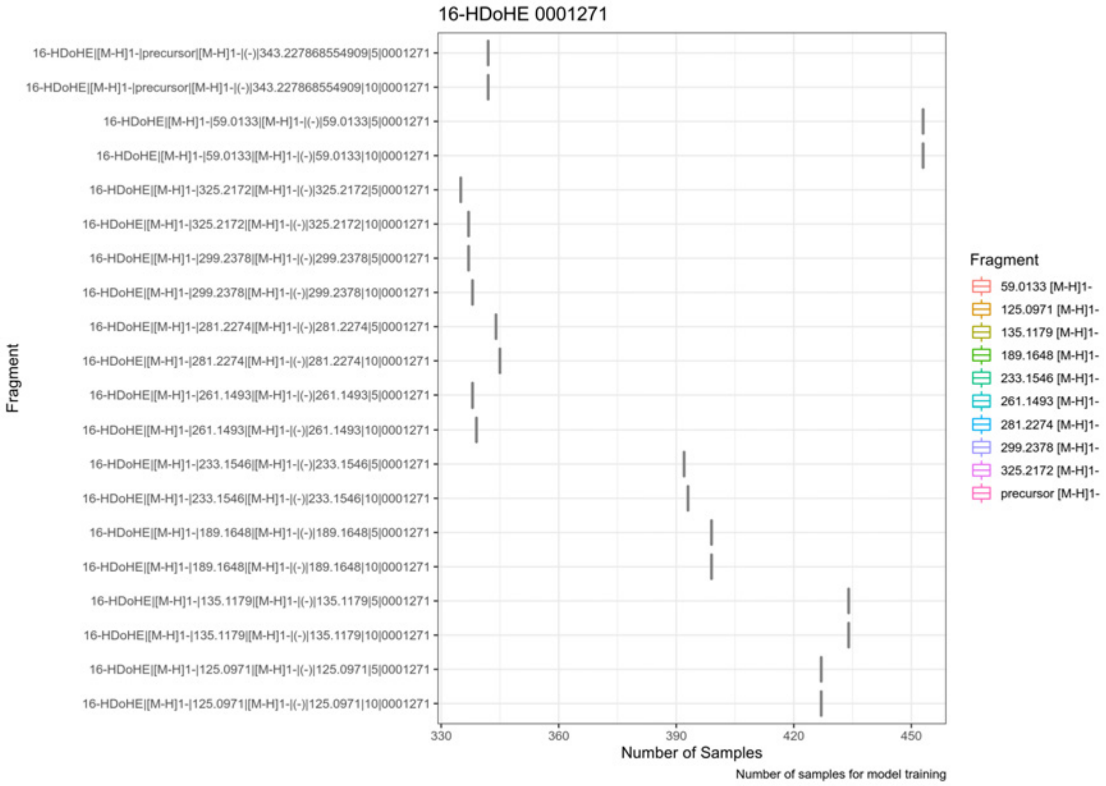

Figure 95. Number of samples used for training per combination Id

1.20. 18-HEPE [M-H]1- 0000131

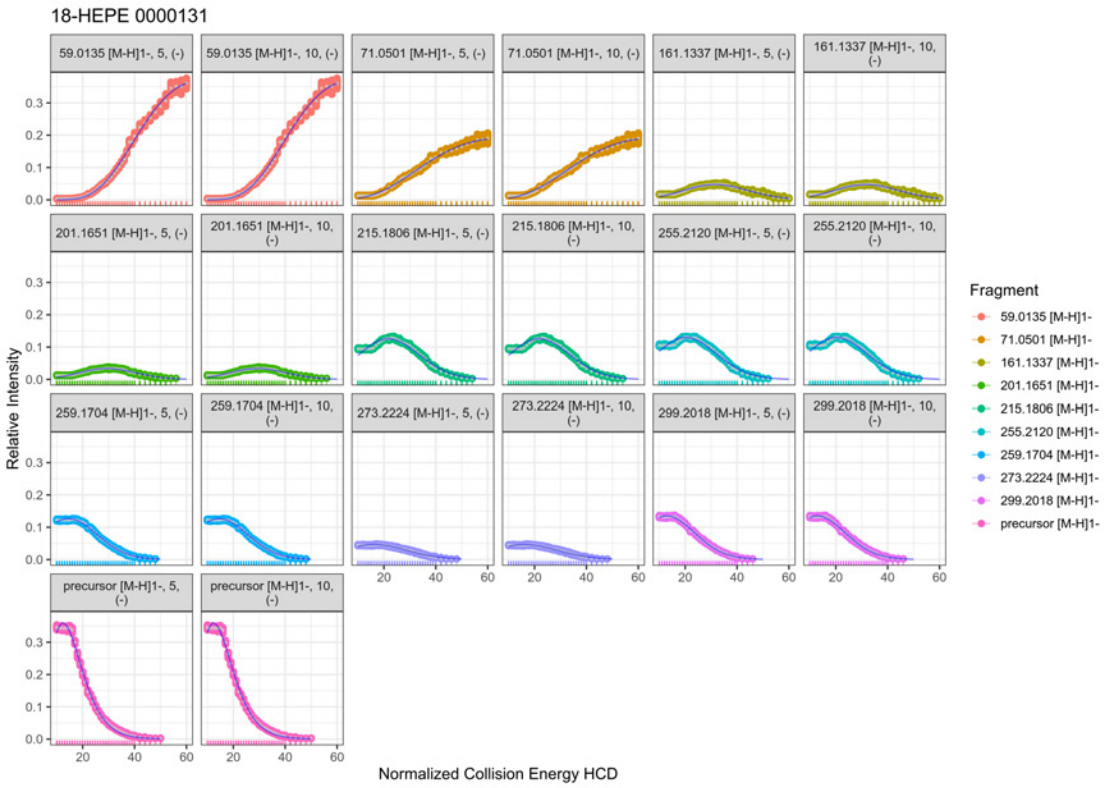

Figure 96. Nonlinear fit

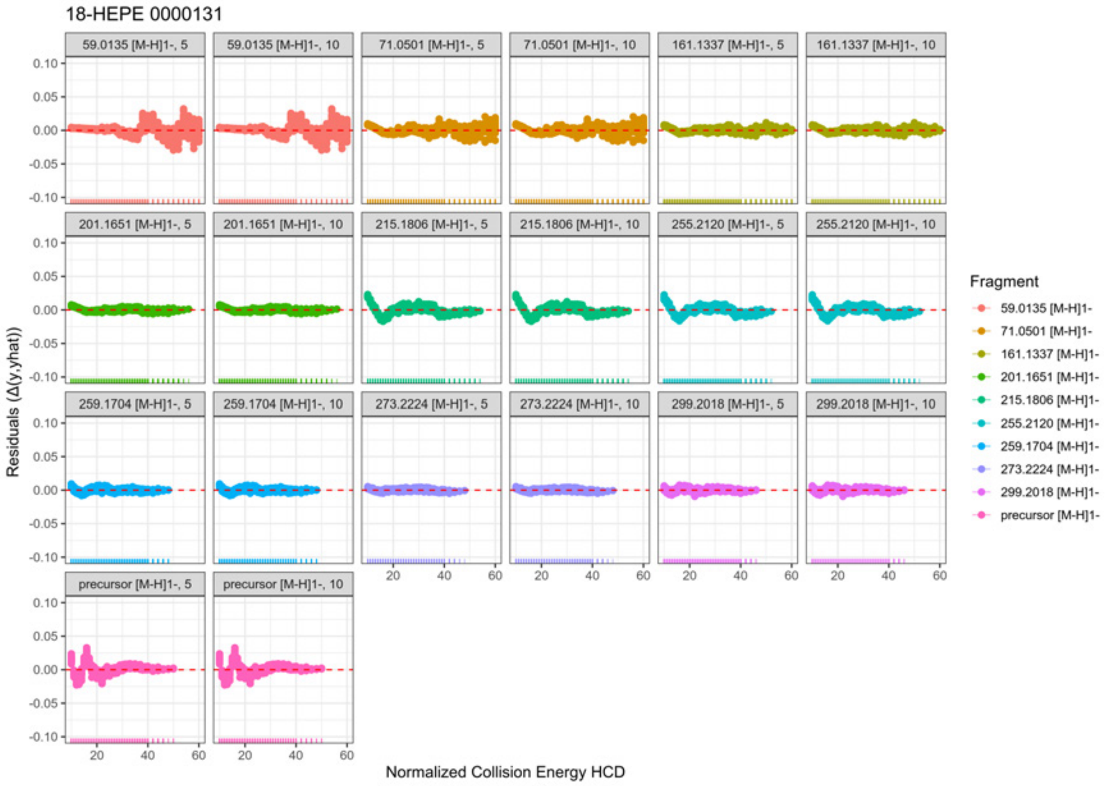

Figure 97. Residuals of nonlinear fit

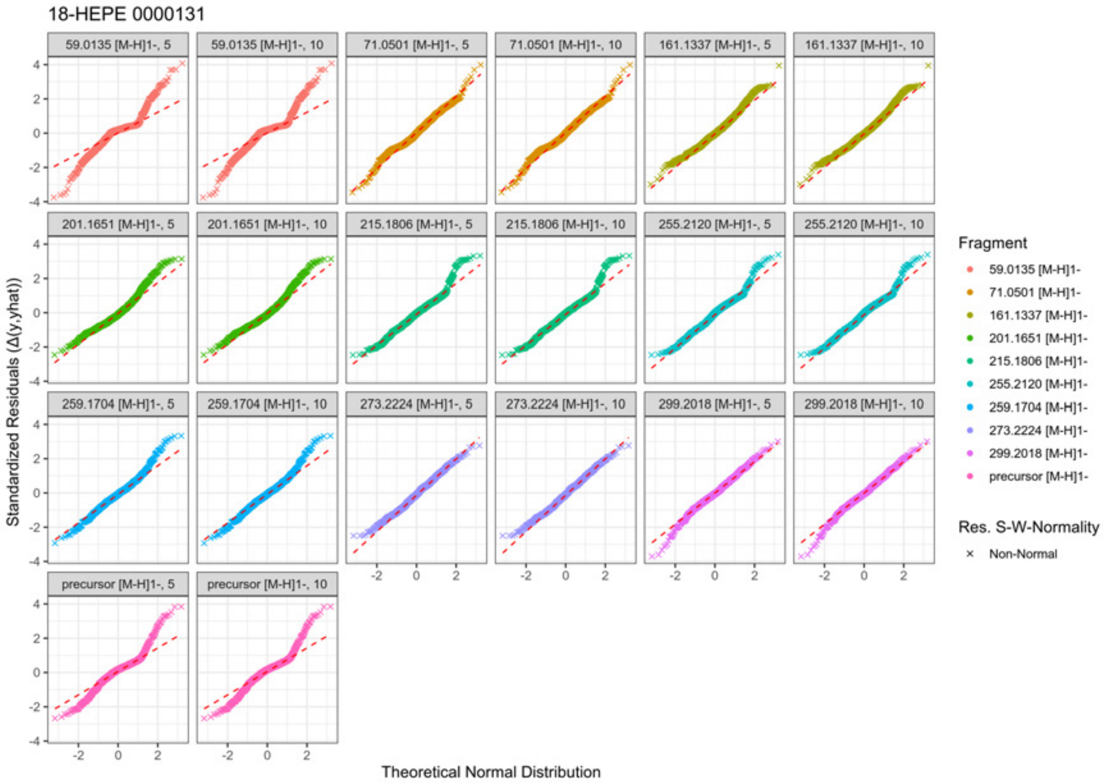

Figure 98. Quantile-quantile plot of residuals

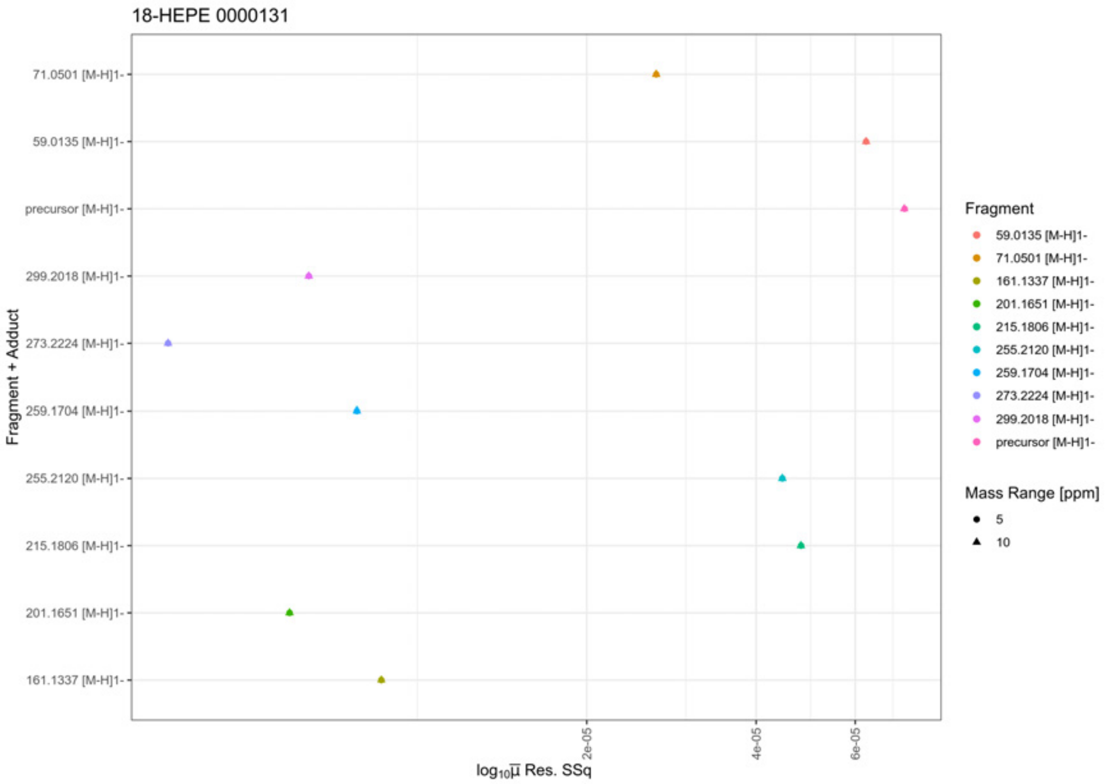

Figure 99. Normalized sum-of-squares of the residuals

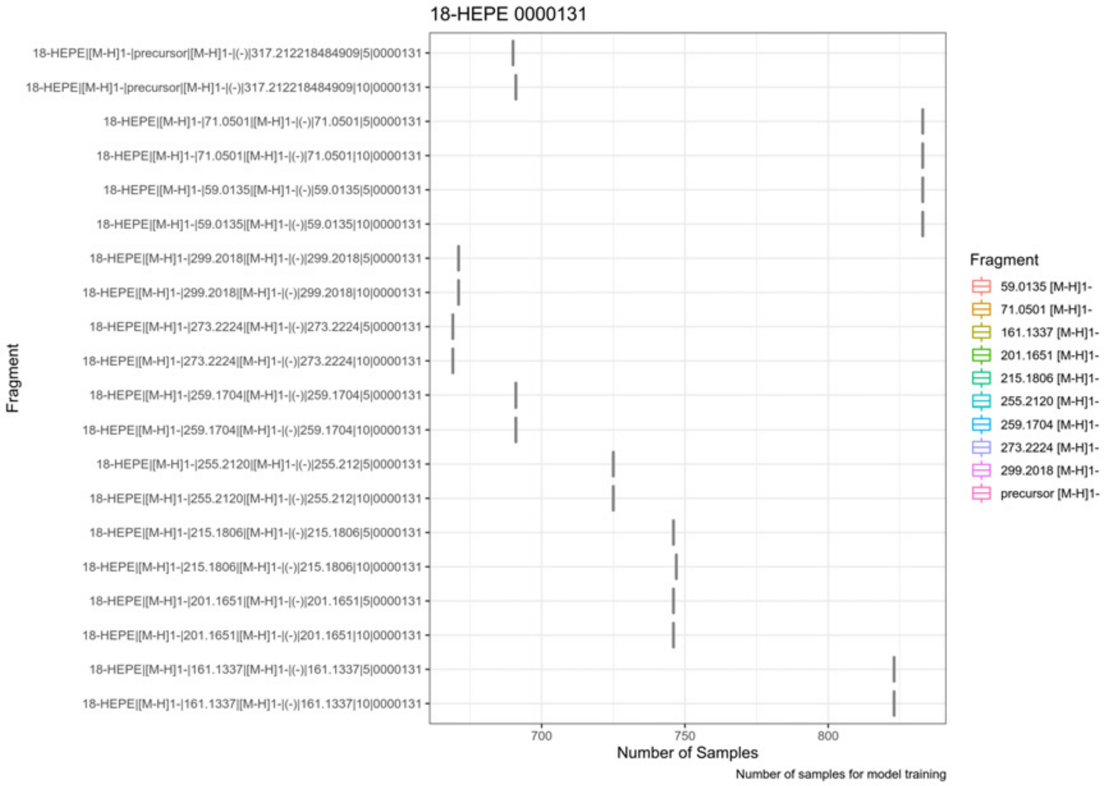

Figure 100. Number of samples used for training per combination Id

1.21. 5(6)-EET{d11} [M-H]1- 0000161

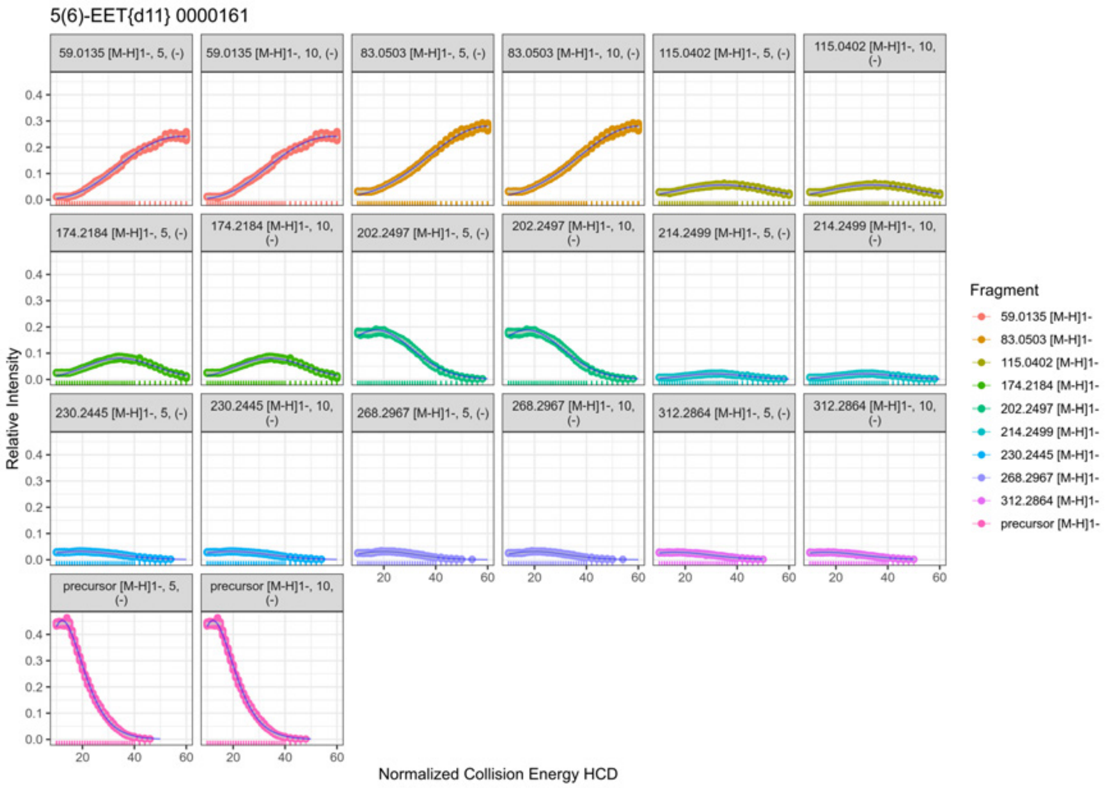

Figure 101. Nonlinear fit

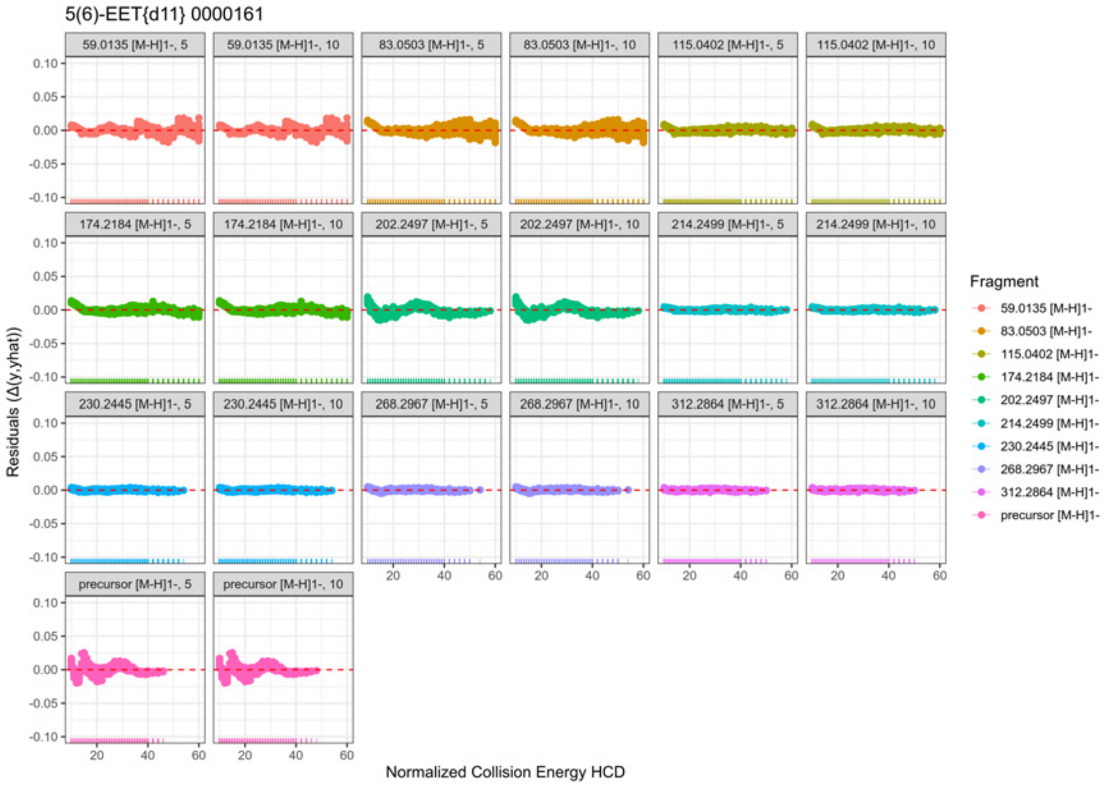

Figure 102. Residuals of nonlinear fit

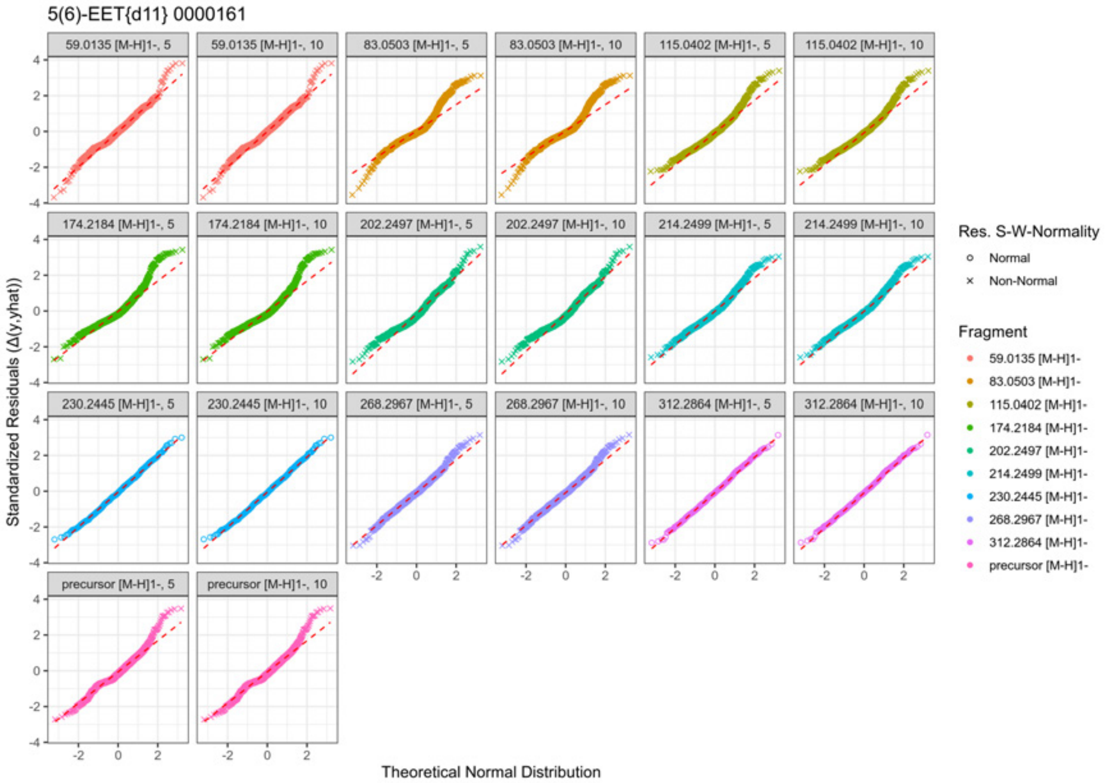

Figure 103. Quantile-quantile plot of residuals

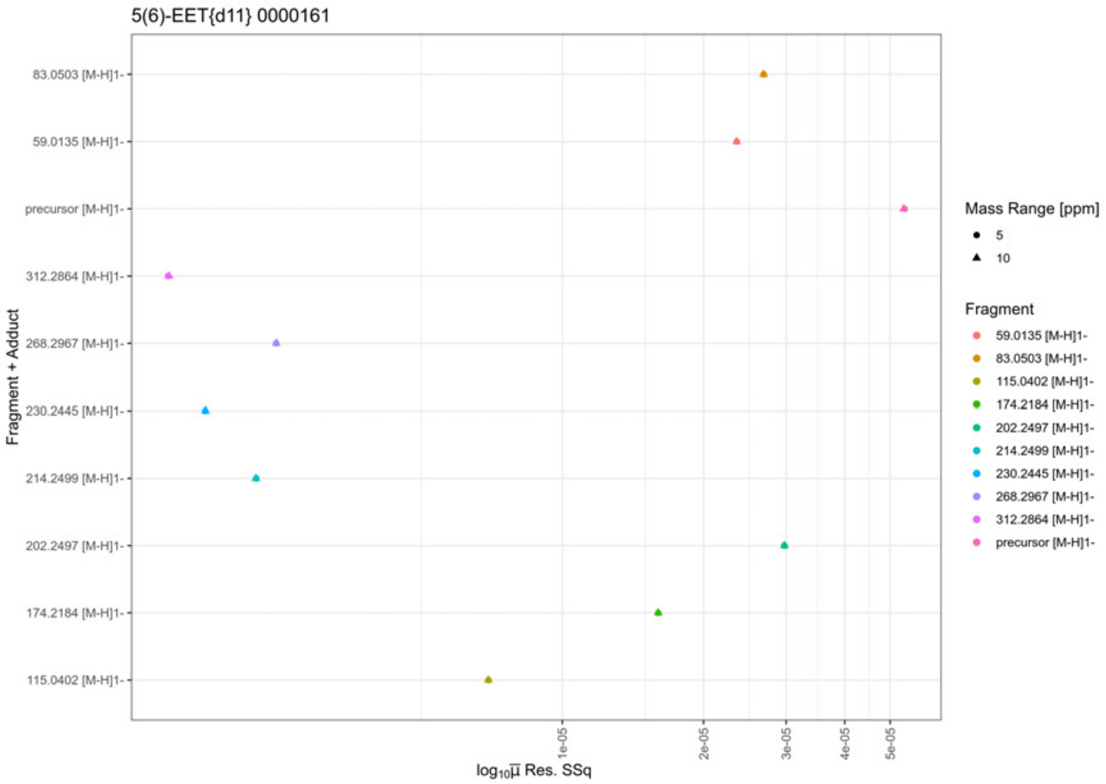

Figure 104. Normalized sum-of-squares of the residuals

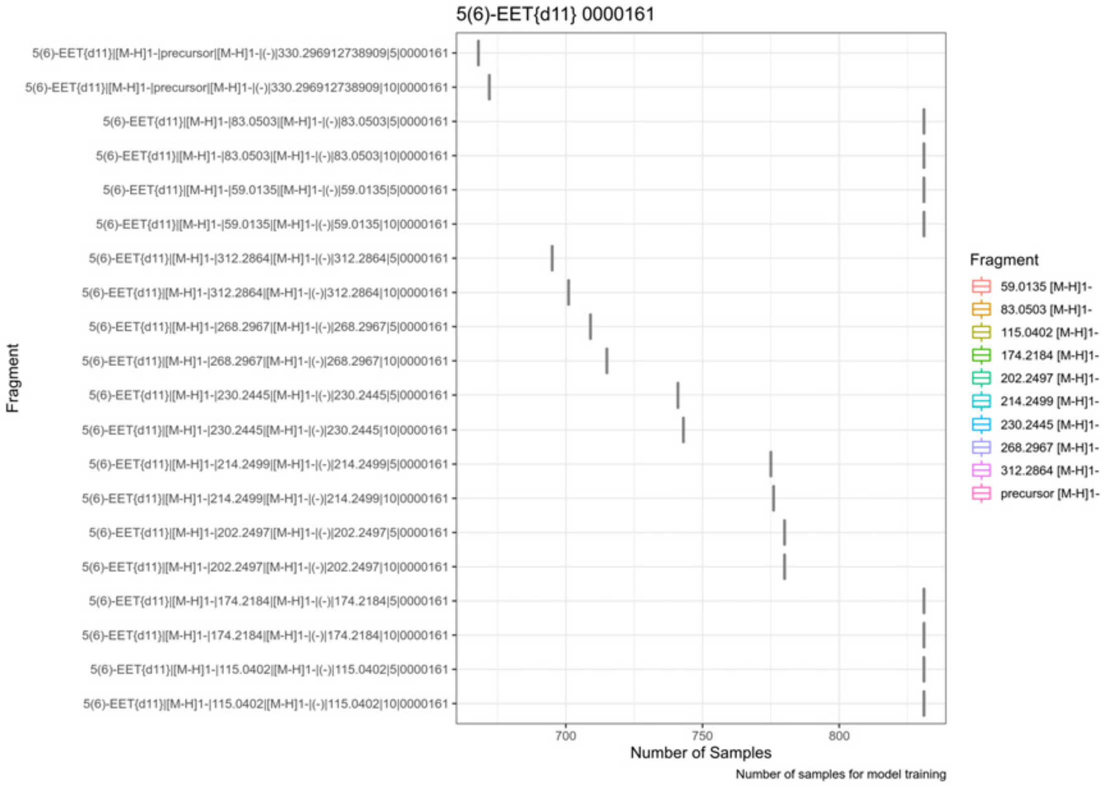

Figure 105. Number of samples used for training per combination Id

1.22. 5,12-DiHETE [M-H]1- 0001299

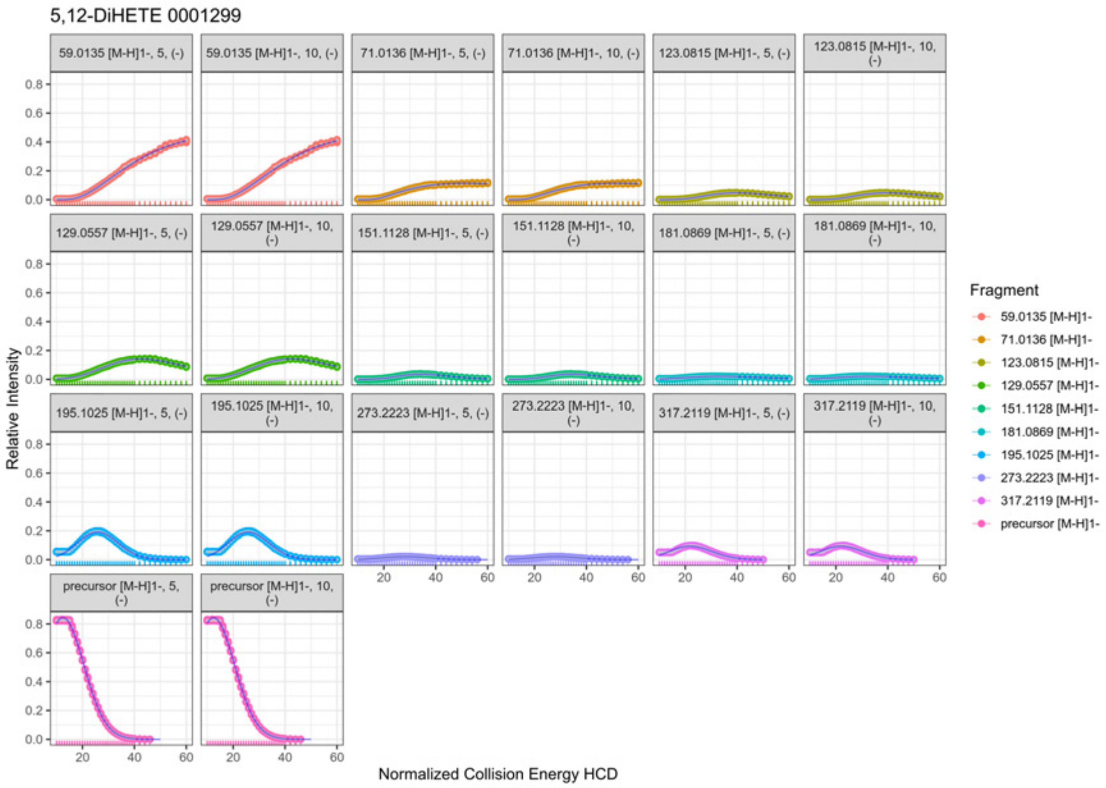

Figure 106. Nonlinear fit

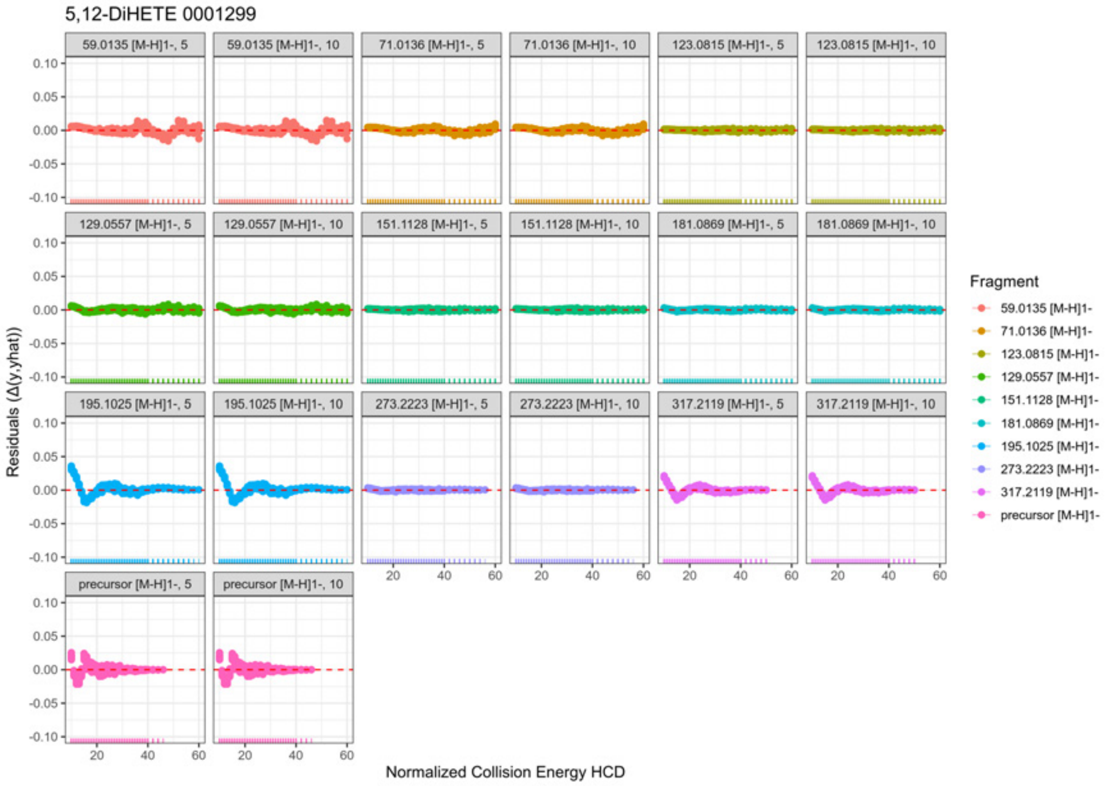

Figure 107. Residuals of nonlinear fit

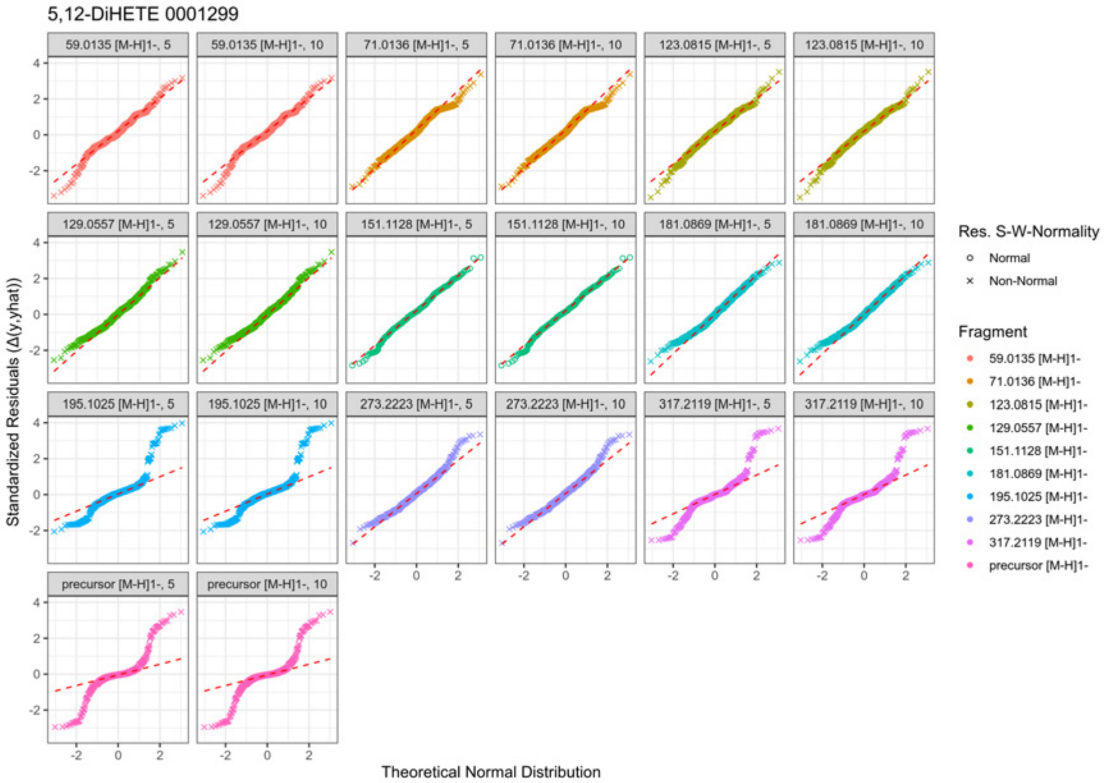

Figure 108. Quantile-quantile plot of residuals

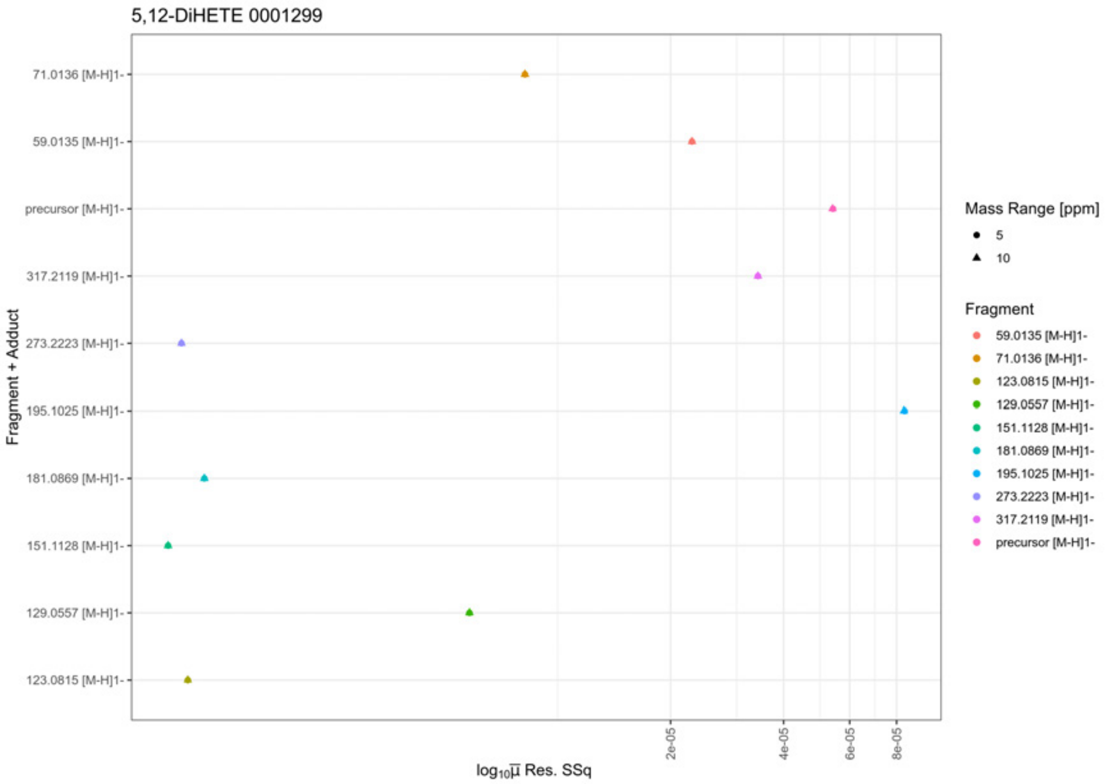

Figure 109. Normalized sum-of-squares of the residuals

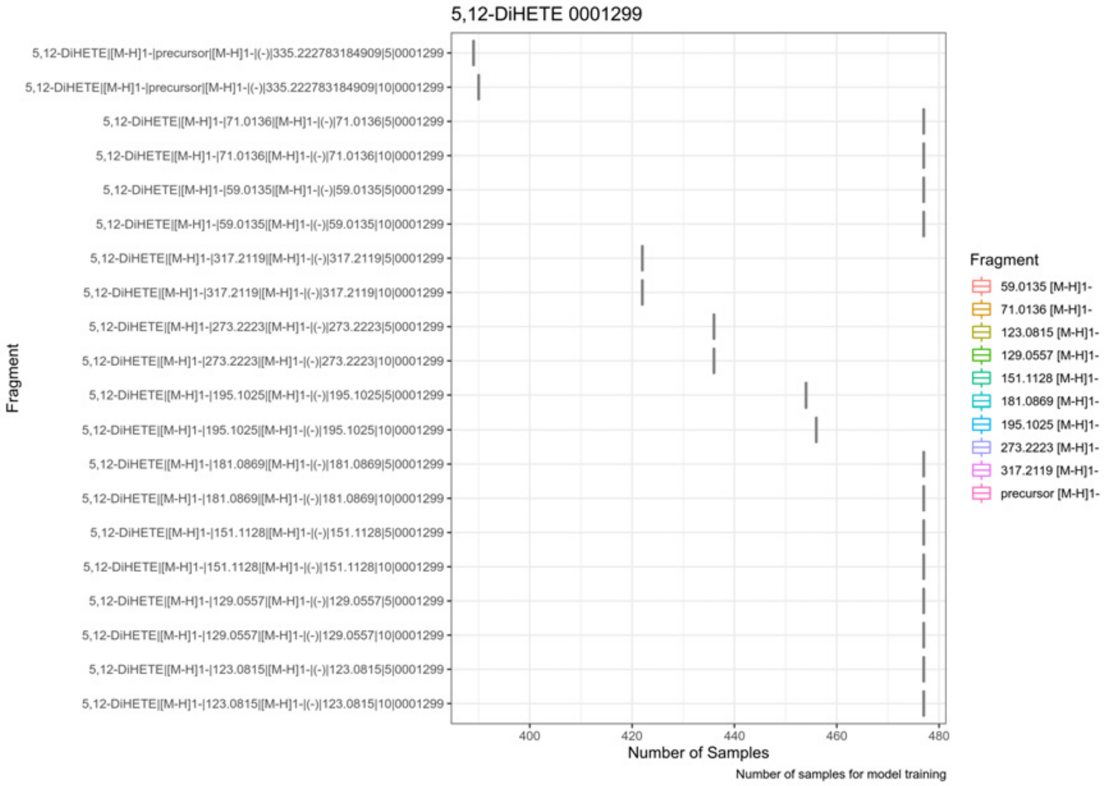

Figure 110. Number of samples used for training per combination Id

1.23. 5,6-DiHETE [M-H]1- 0001301

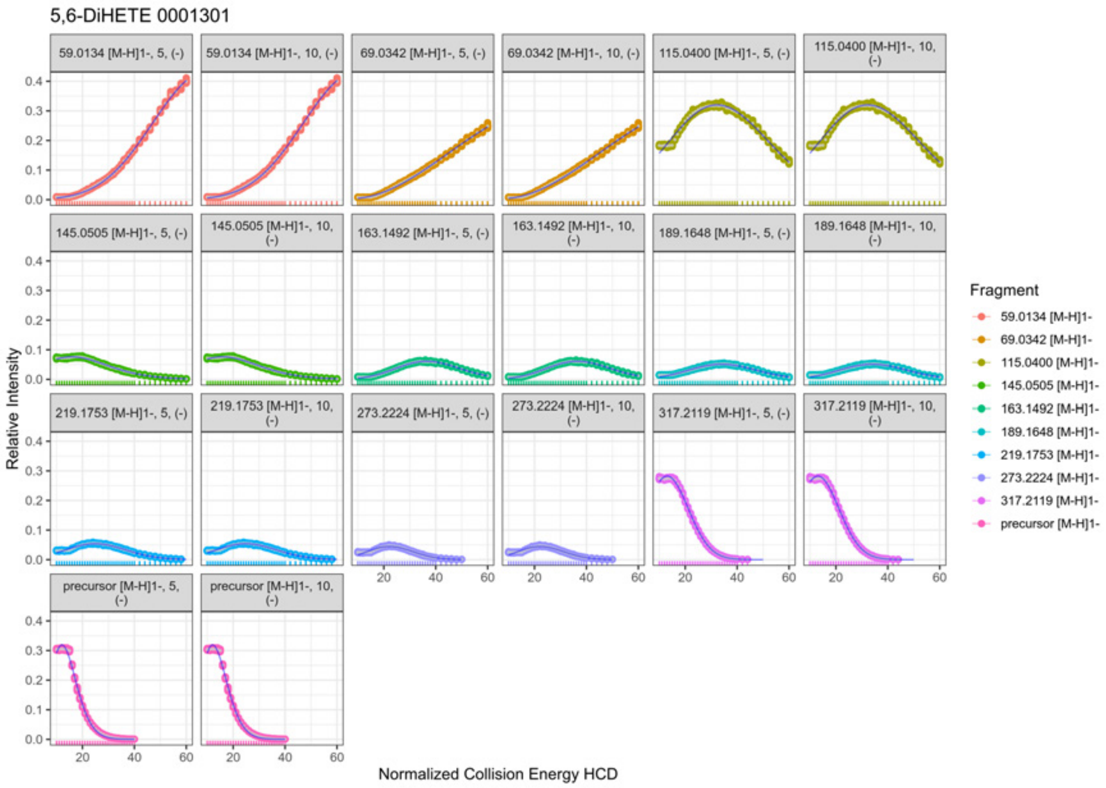

Figure 111. Nonlinear fit

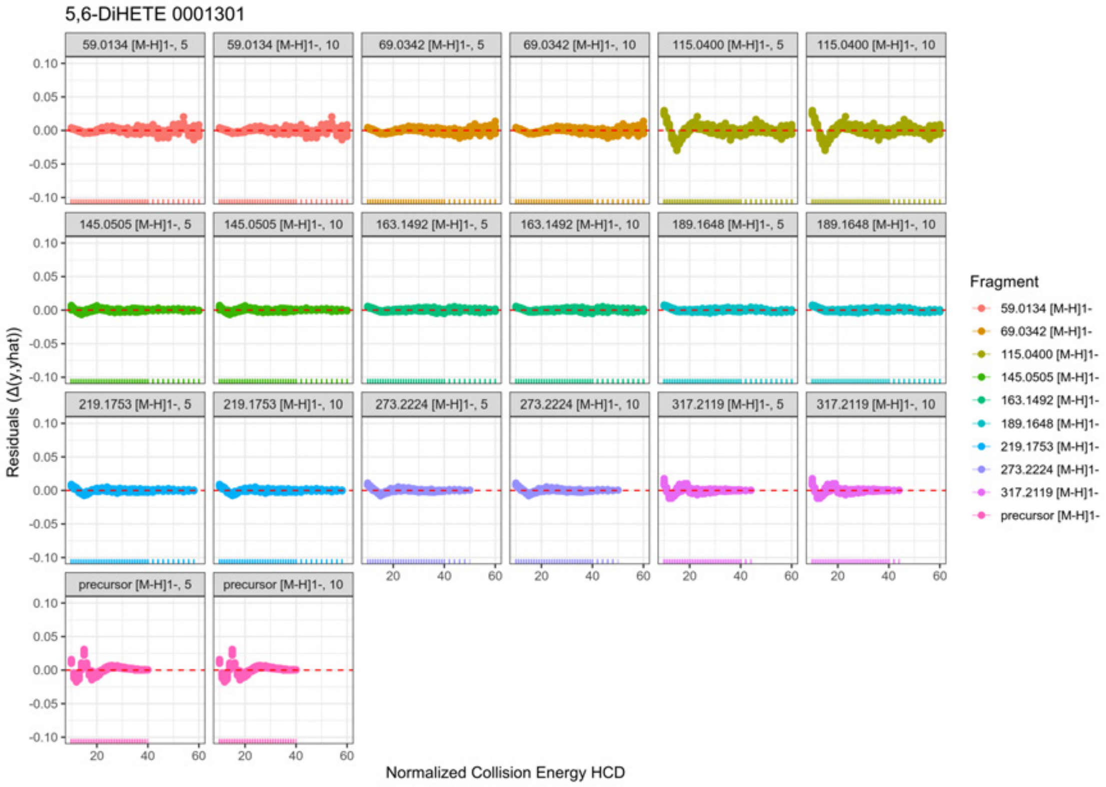

Figure 112. Residuals of nonlinear fit

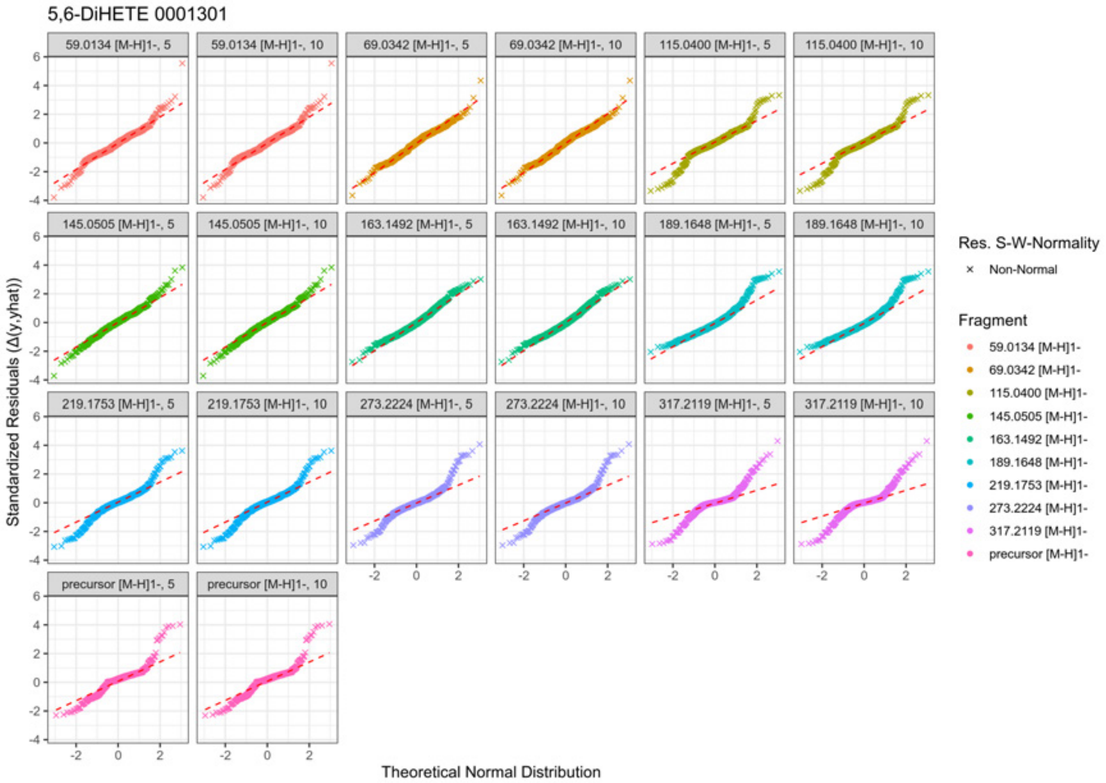

Figure 113. Quantile-quantile plot of residuals

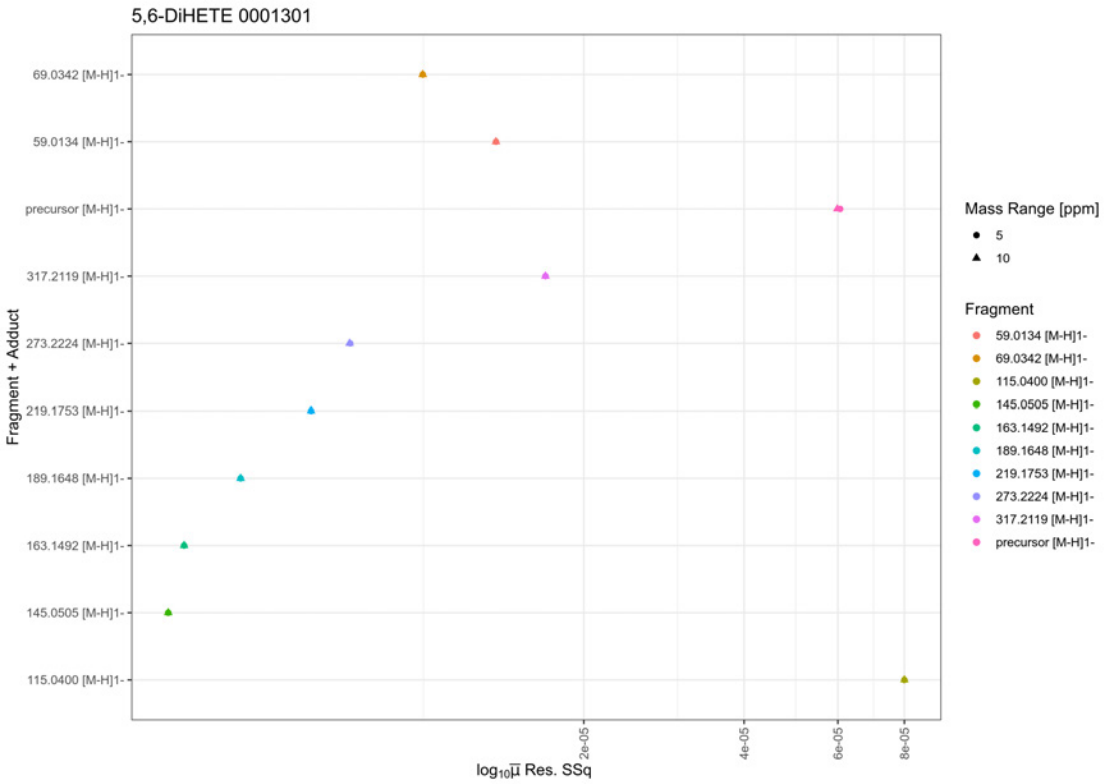

Figure 114. Normalized sum-of-squares of the residuals

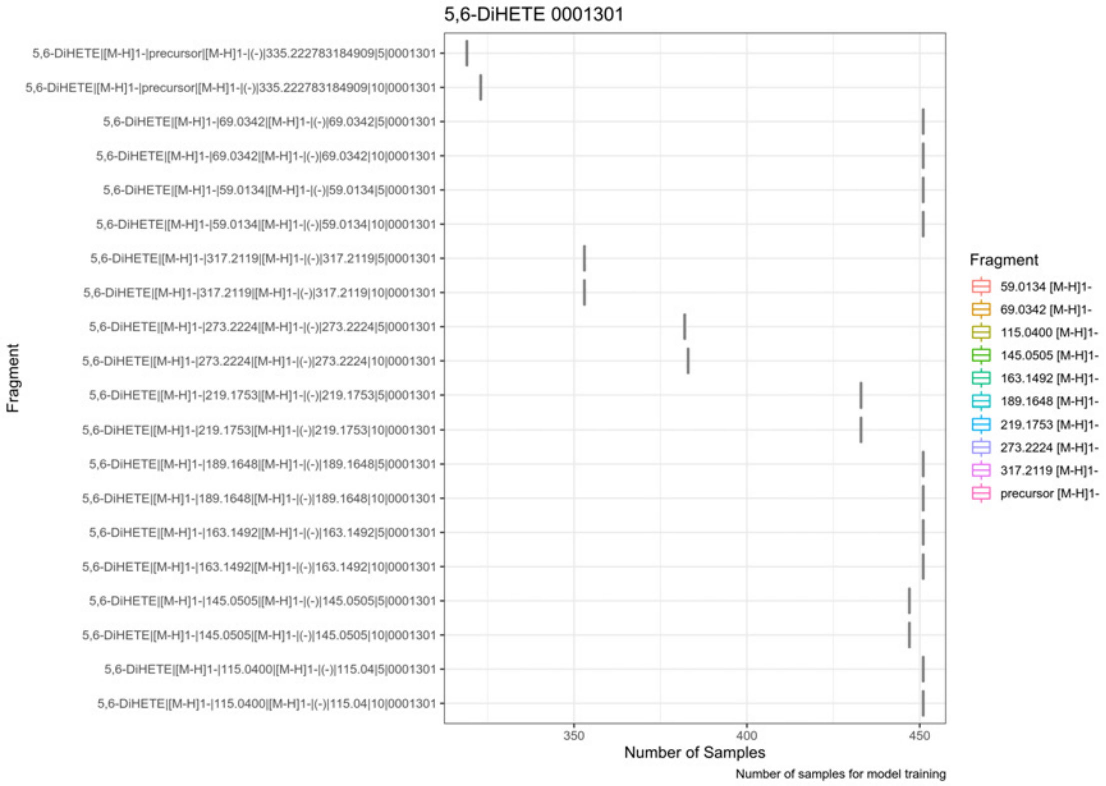

Figure 115. Number of samples used for training per combination Id

1.24. 5-HEPE [M-H]1- 0000133

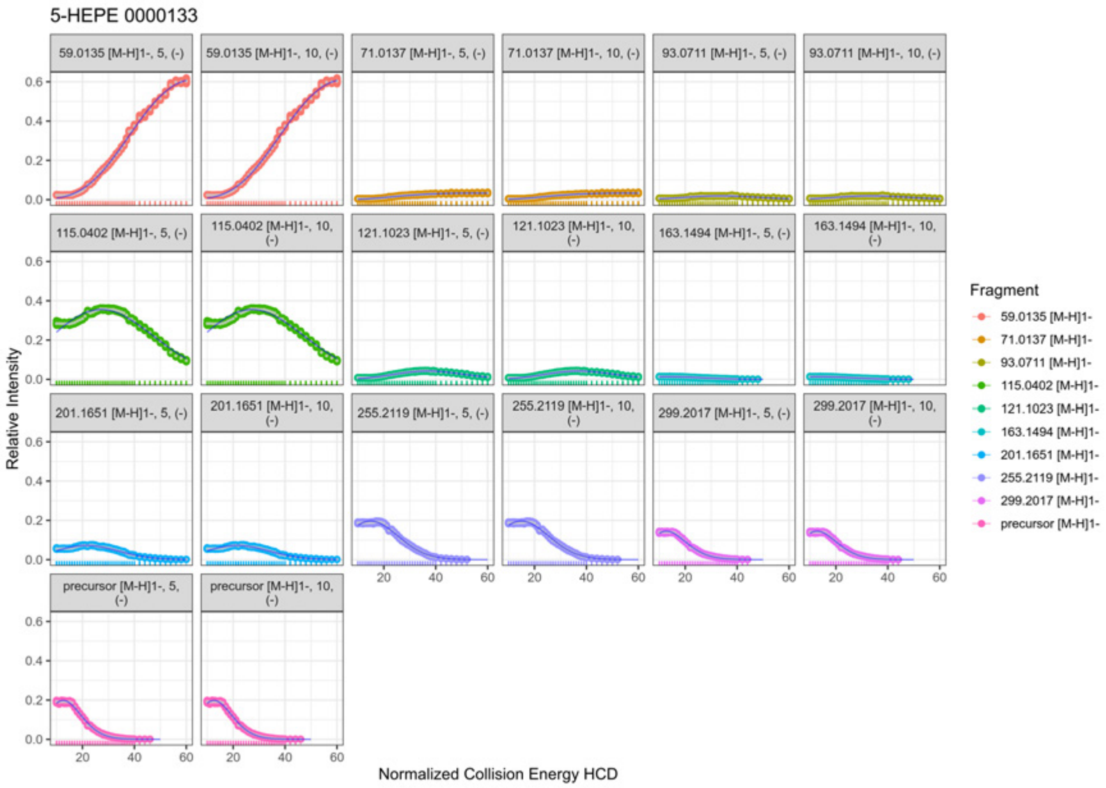

Figure 116. Nonlinear fit

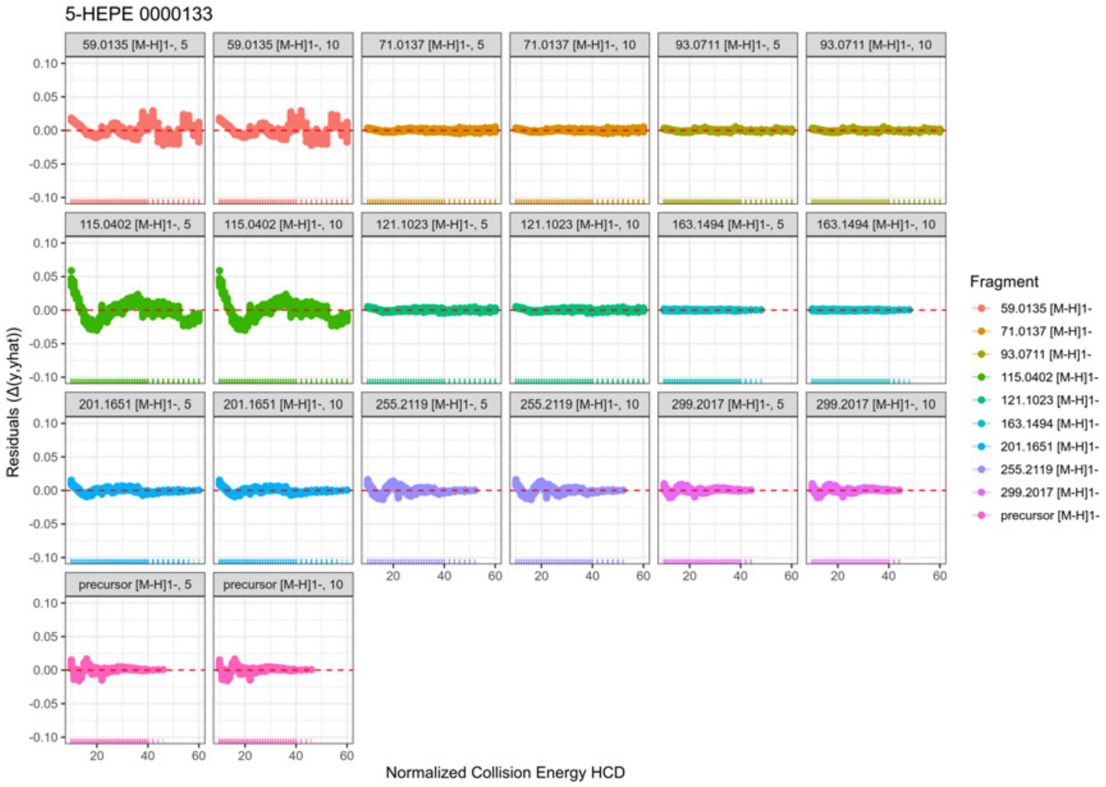

Figure 117. Residuals of nonlinear fit

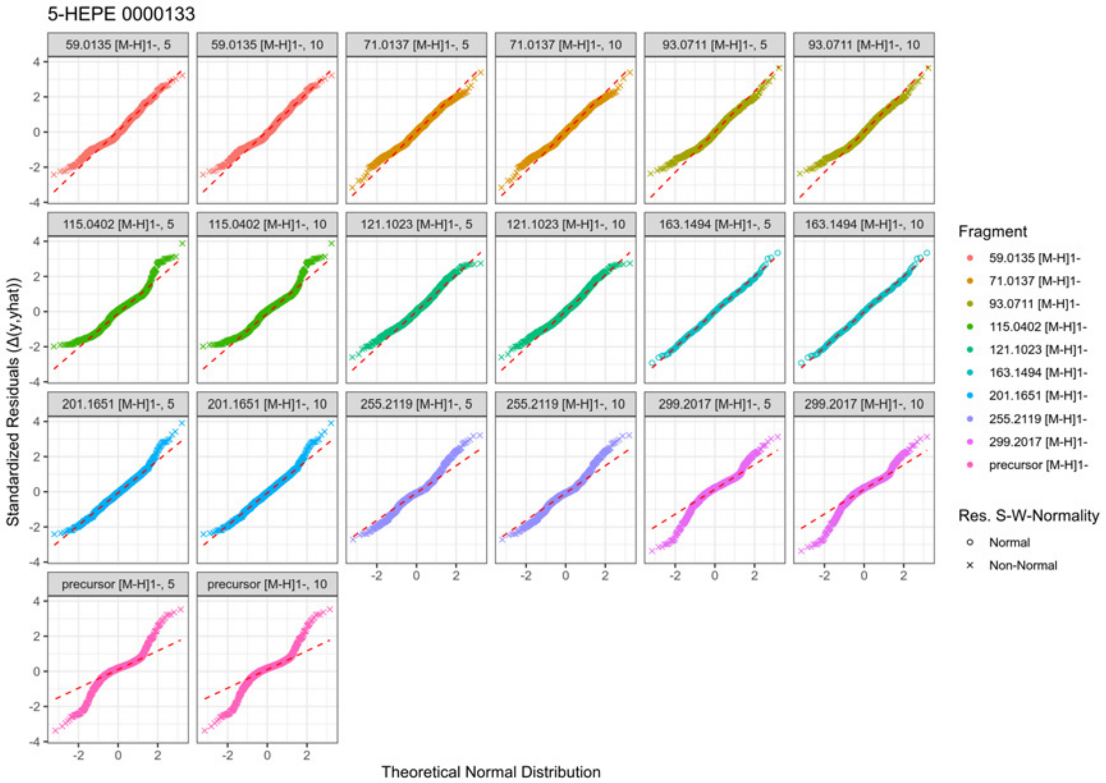

Figure 118. Quantile-quantile plot of residuals

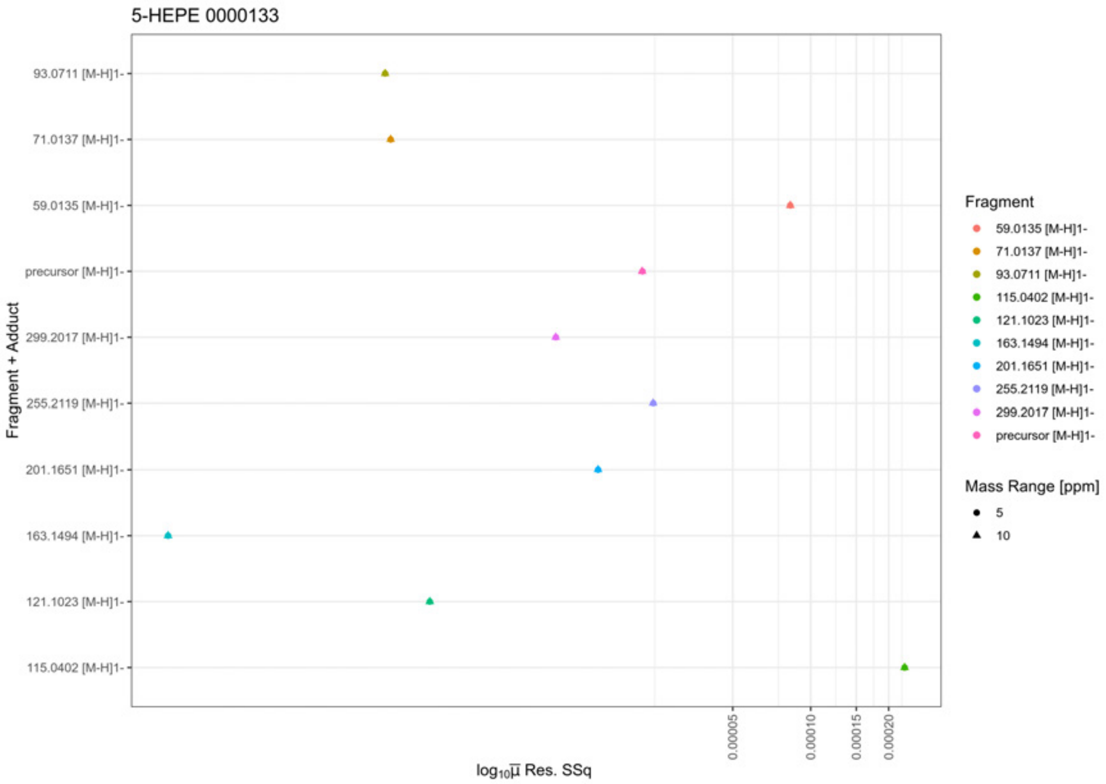

Figure 119. Normalized sum-of-squares of the residuals

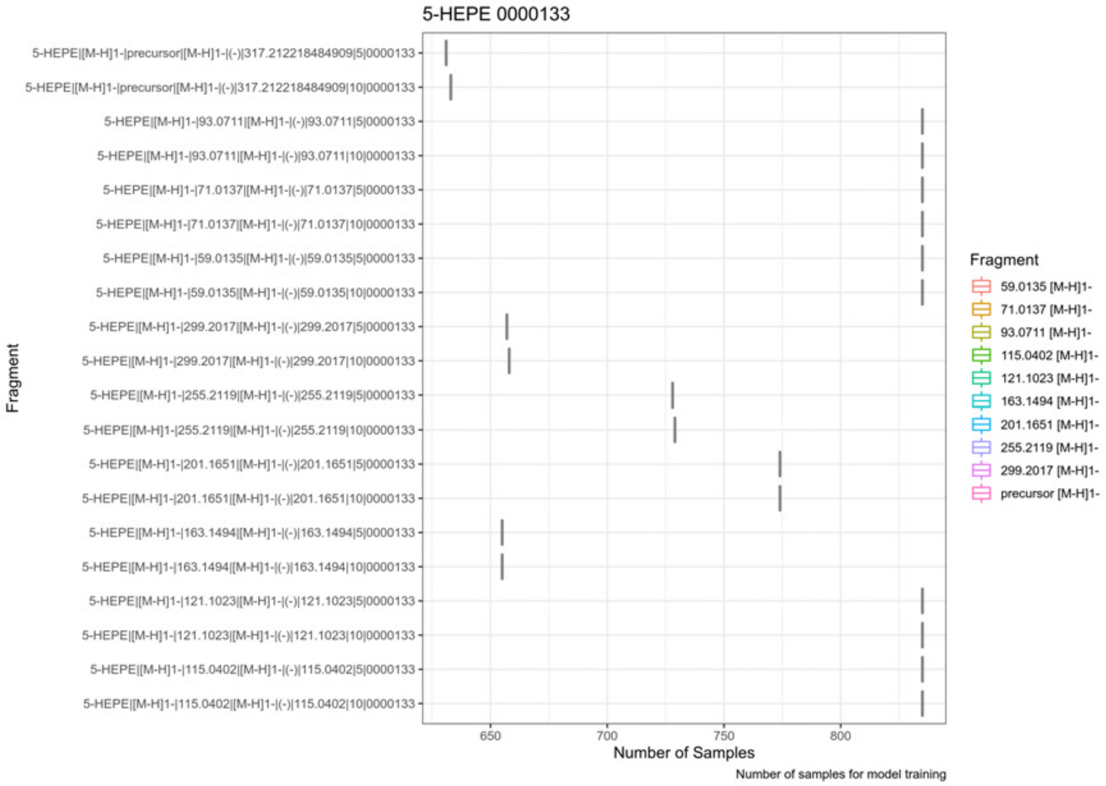

Figure 120. Number of samples used for training per combination Id

1.25. 5-HETE{d8} [M-H]1- 0000145

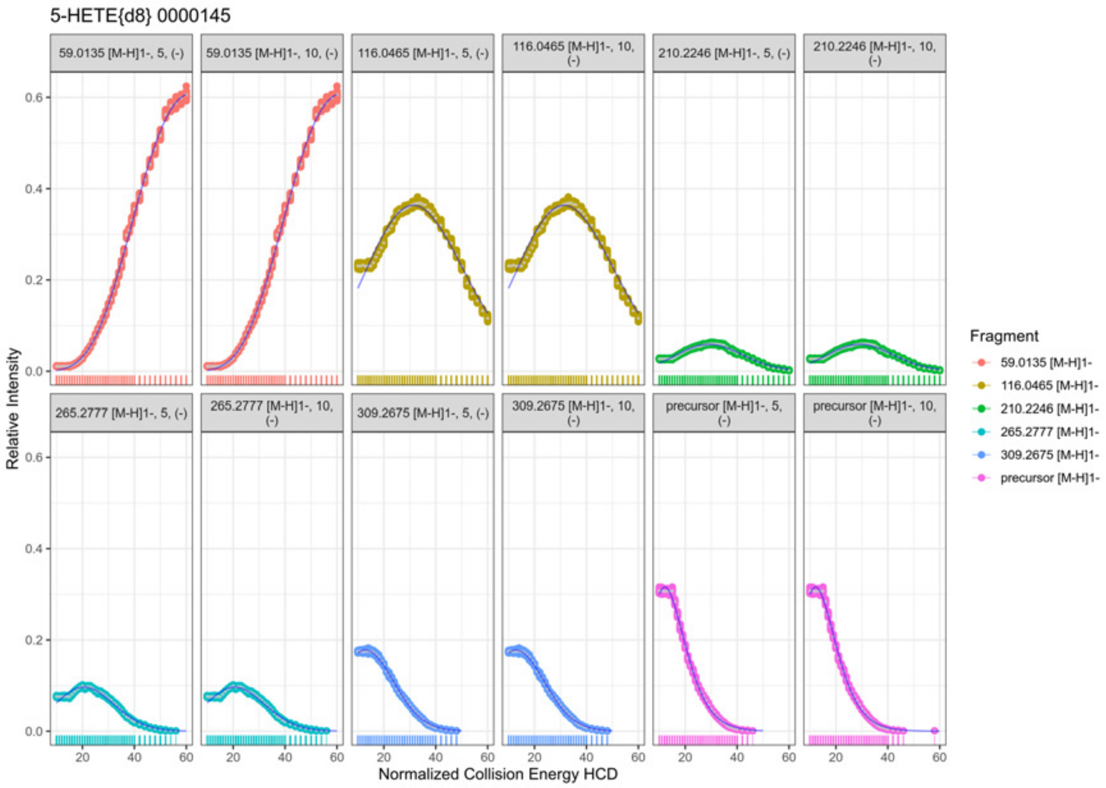

Figure 121. Nonlinear fit

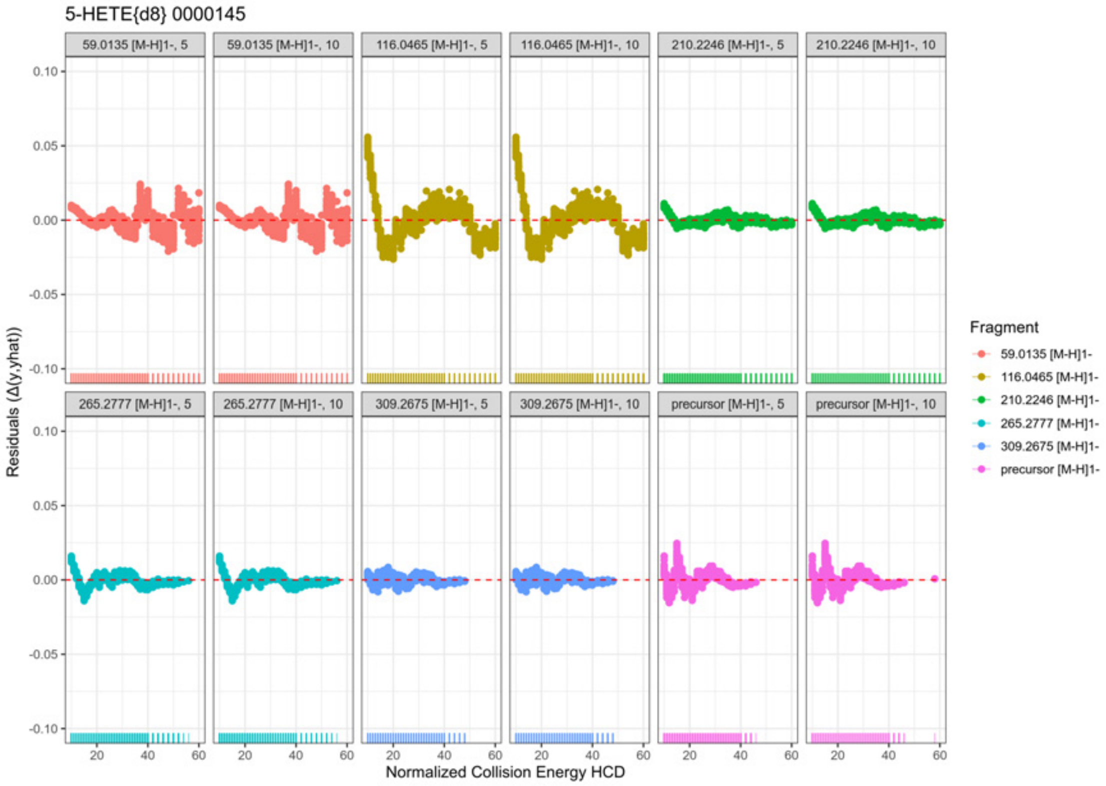

Figure 122. Residuals of nonlinear fit

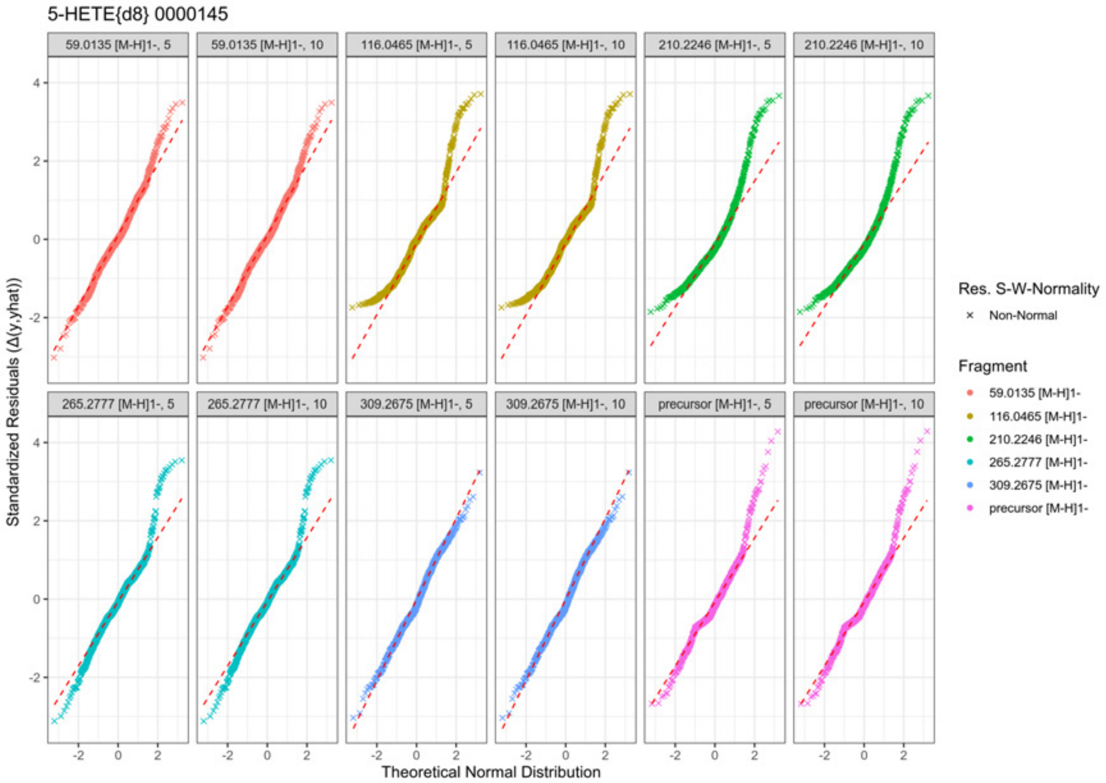

Figure 123. Quantile-quantile plot of residuals

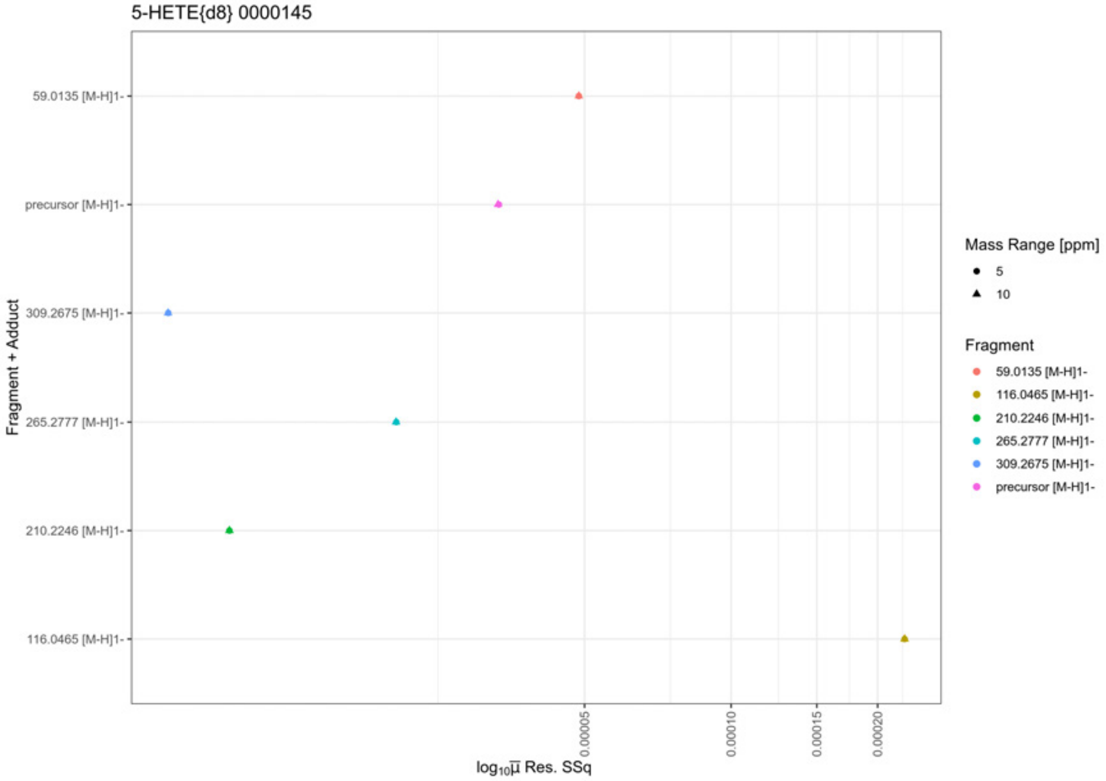

Figure 124. Normalized sum-of-squares of the residuals

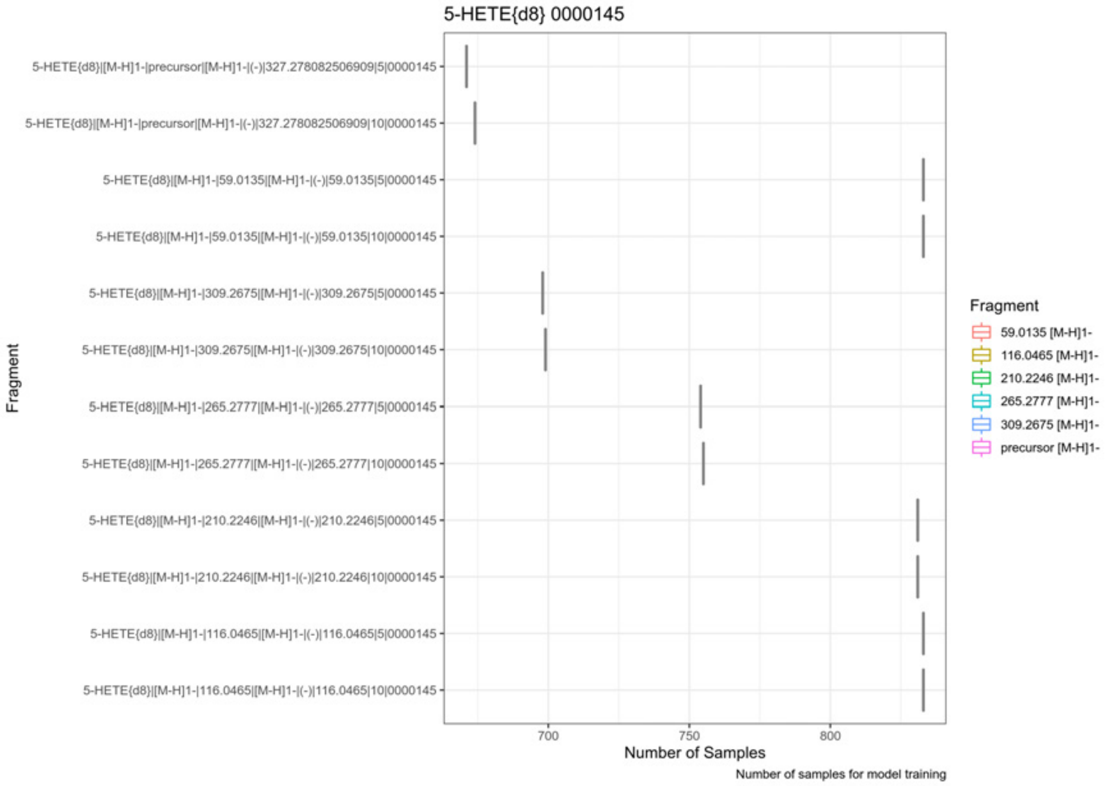

Figure 125. Number of samples used for training per combination Id

1.26. 5-HpETE [M-H]1- 0001297

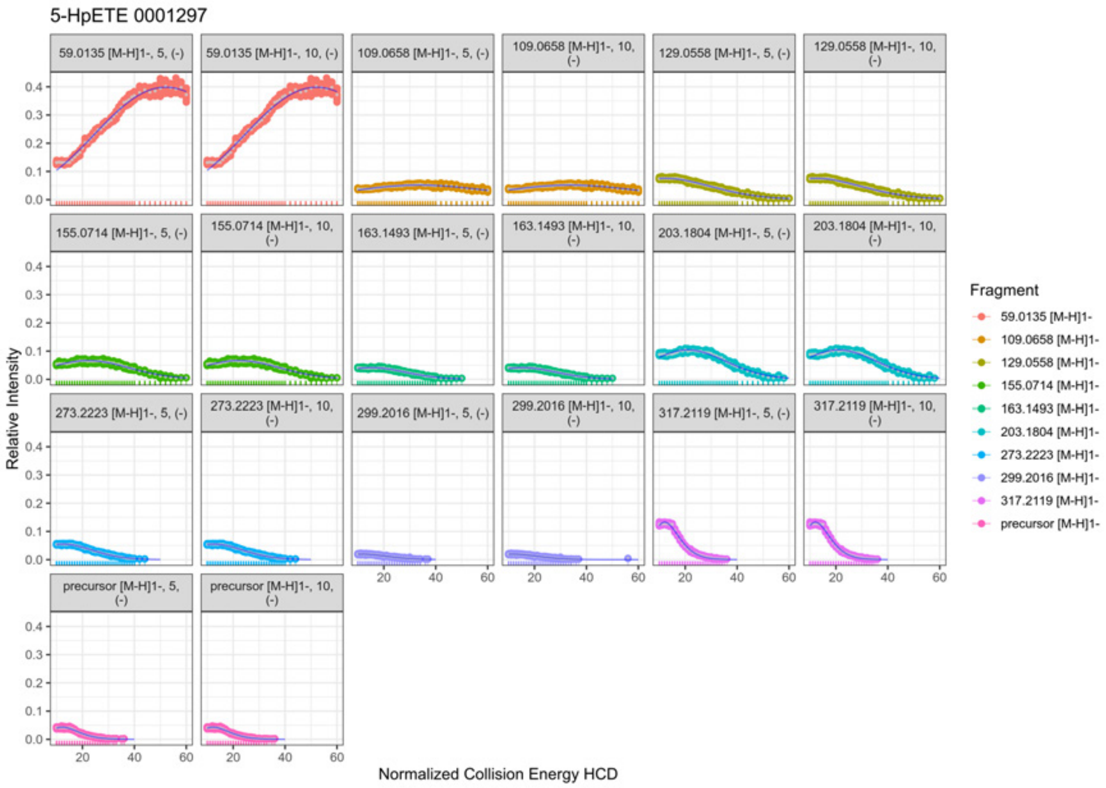

Figure 126. Nonlinear fit

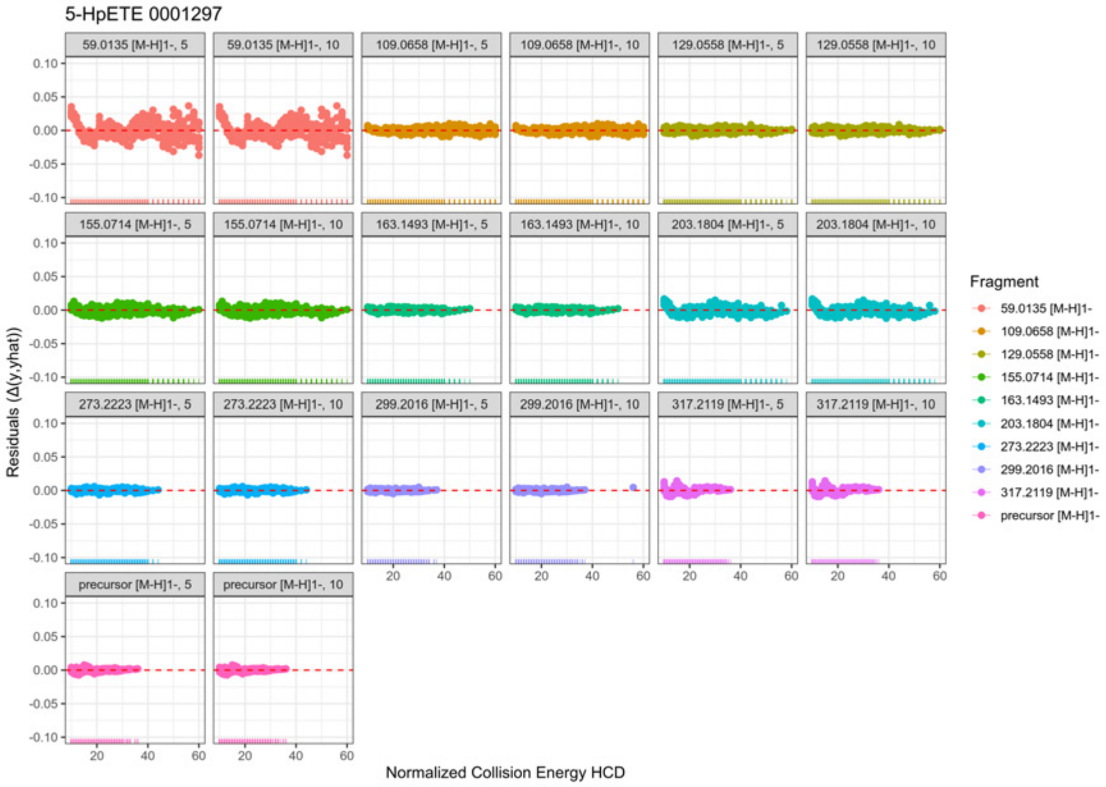

Figure 127. Residuals of nonlinear fit

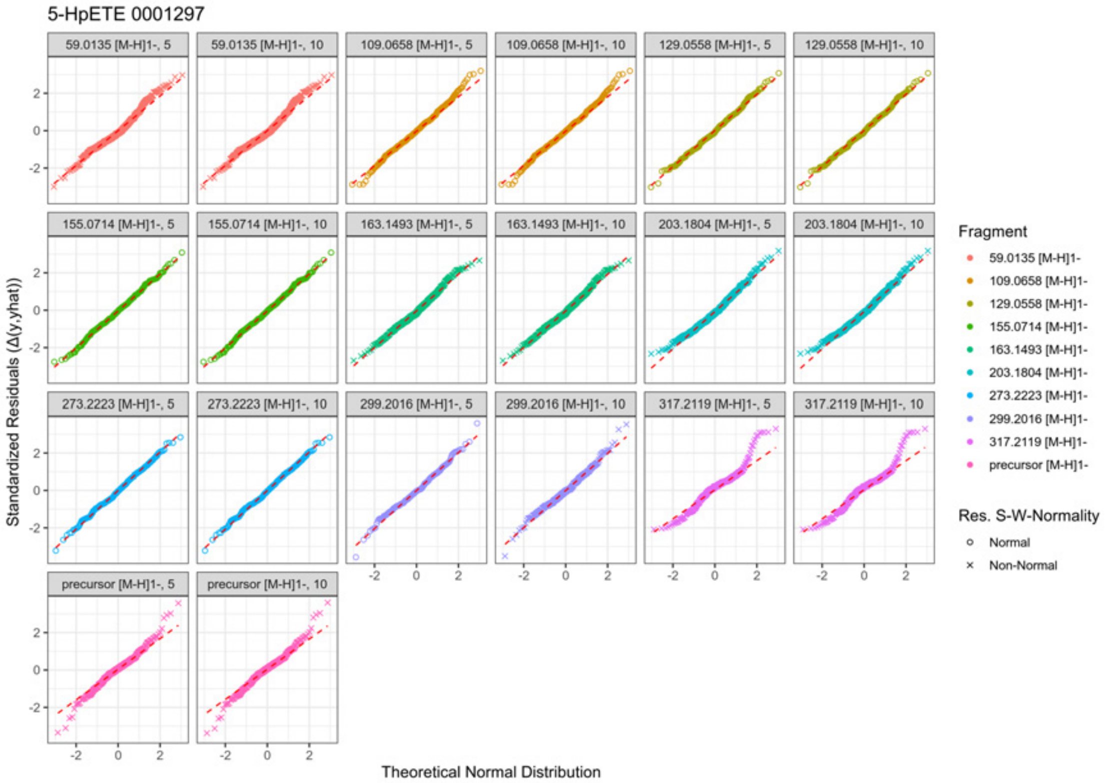

Figure 128. Quantile-quantile plot of residuals

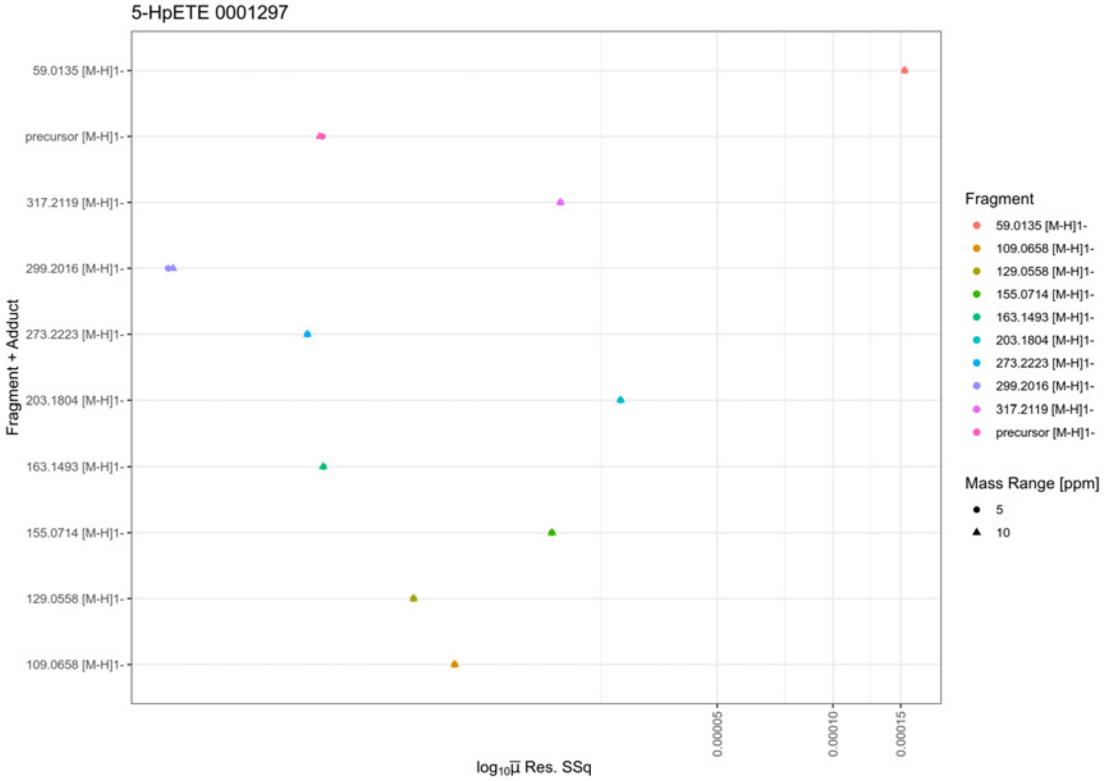

Figure 129. Normalized sum-of-squares of the residuals

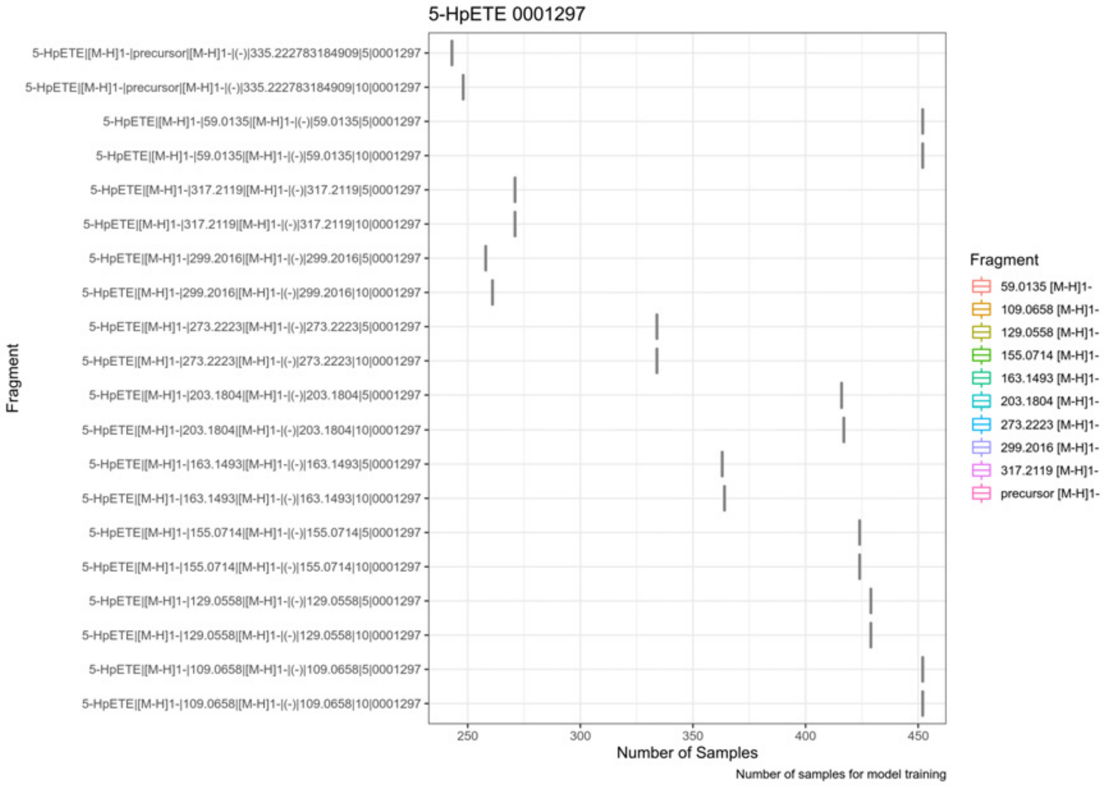

Figure 130. Number of samples used for training per combination Id

1.27. 5-OxoETE{d7} [M-H]1- 0000147

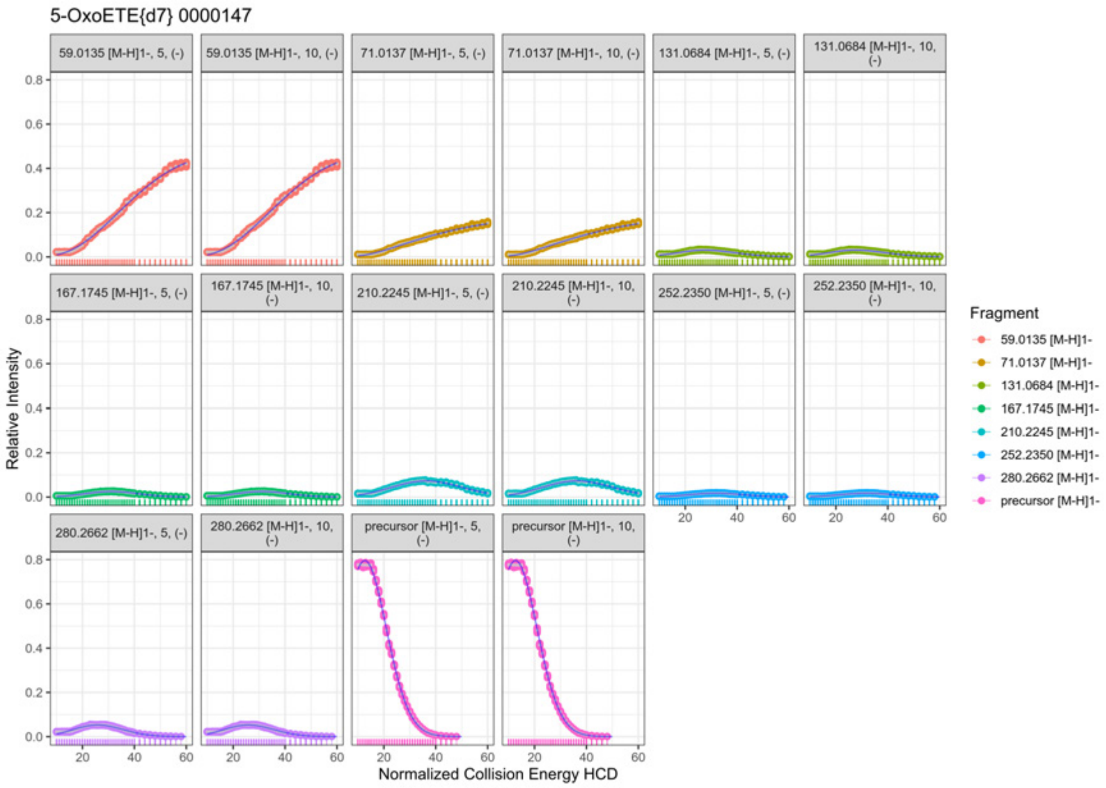

Figure 131. Nonlinear fit

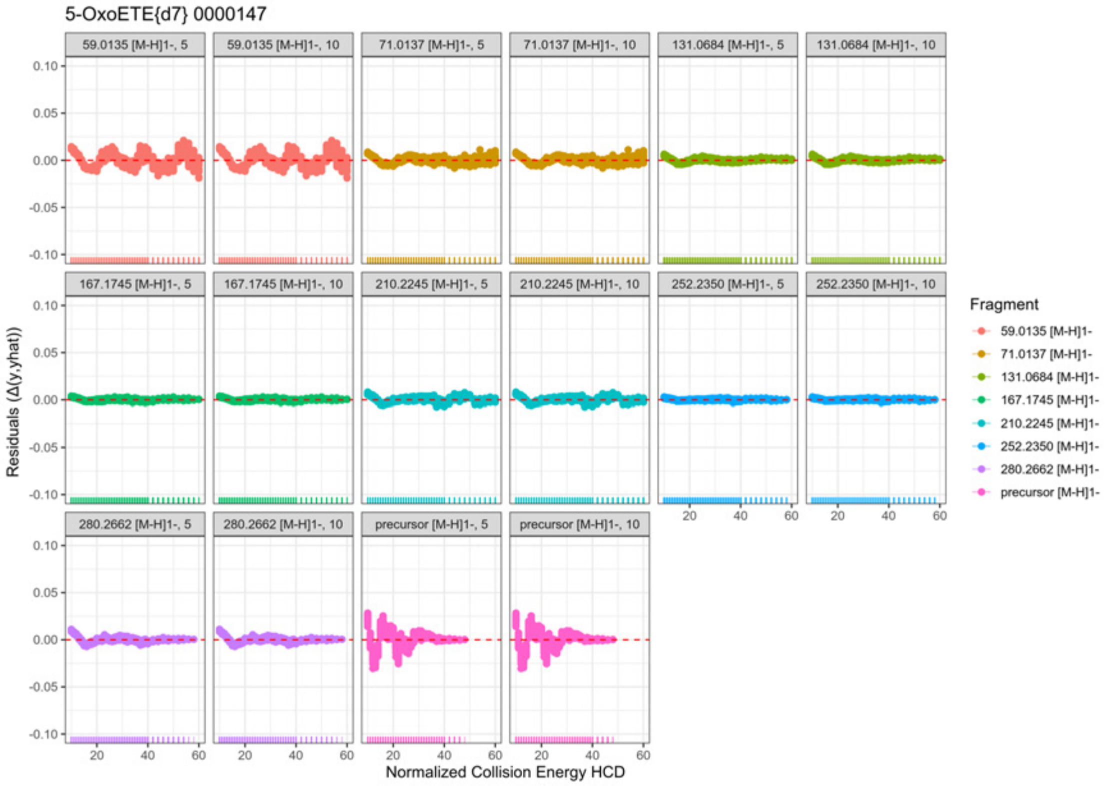

Figure 132. Residuals of nonlinear fit

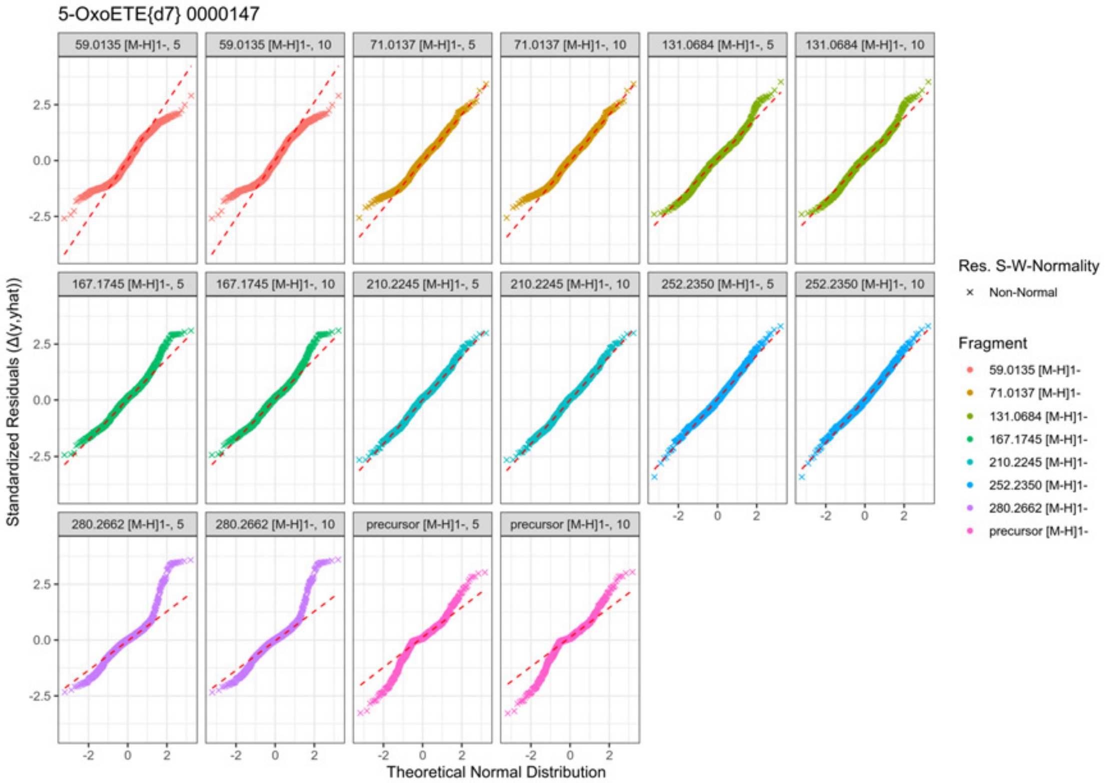

Figure 133. Quantile-quantile plot of residuals

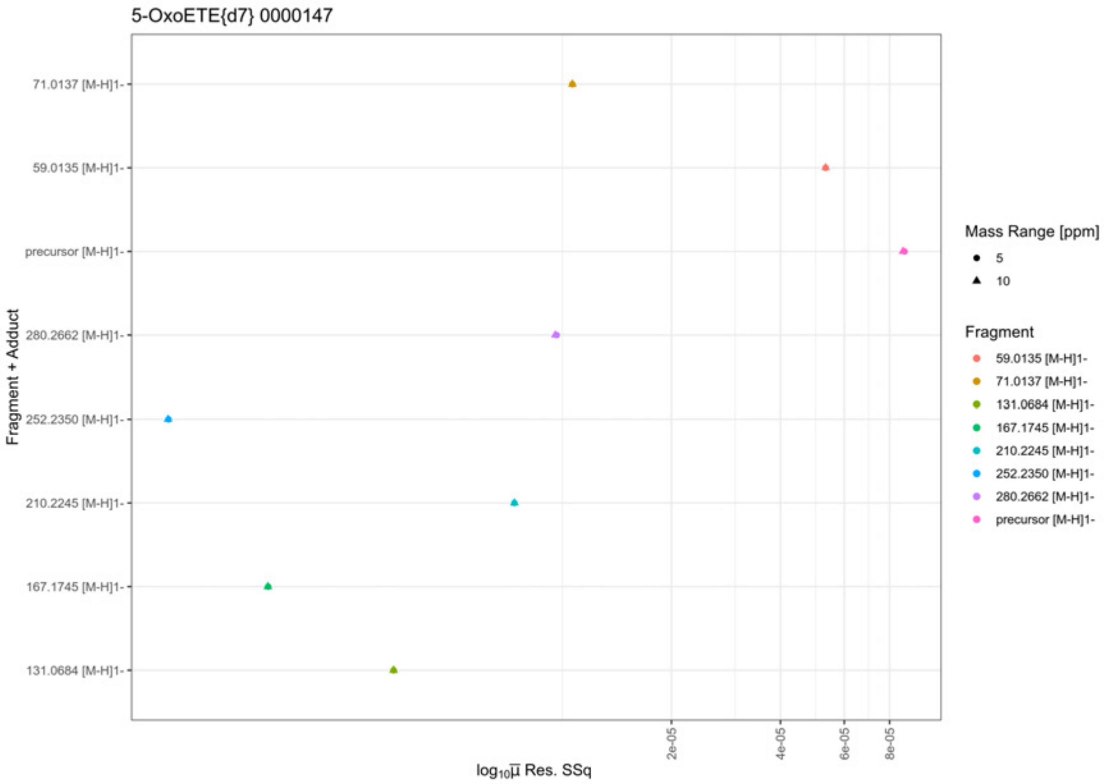

Figure 134. Normalized sum-of-squares of the residuals

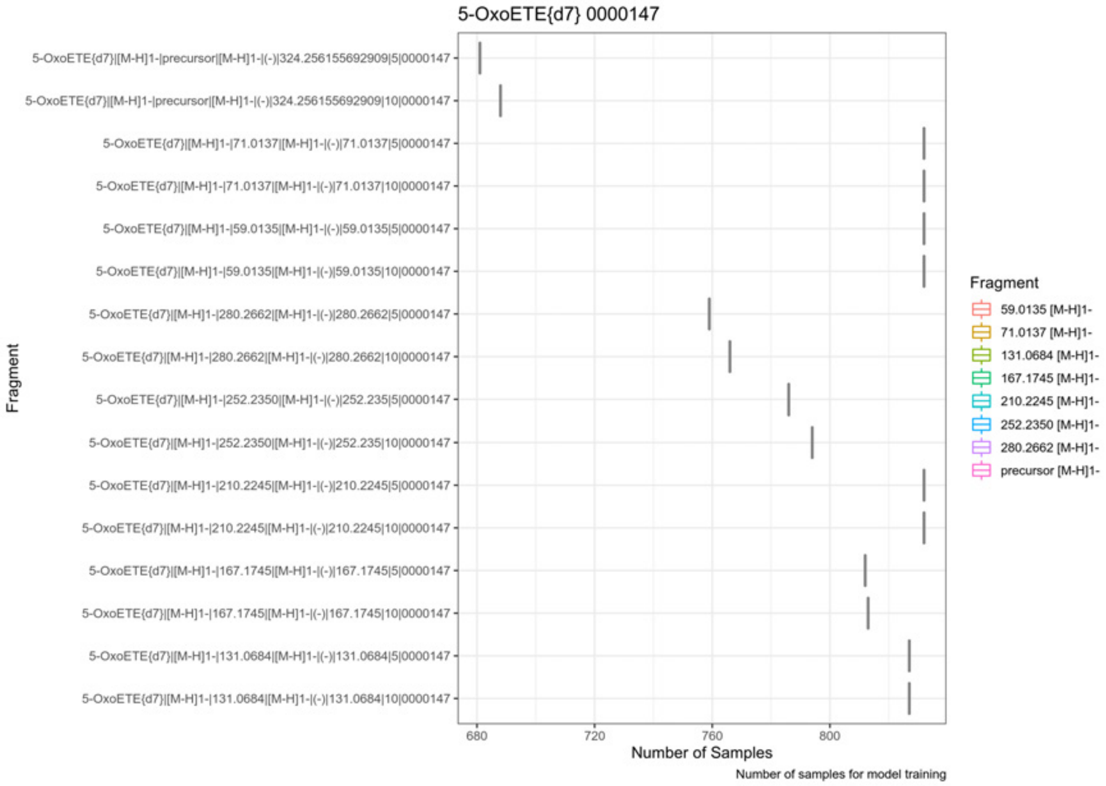

Figure 135. Number of samples used for training per combination Id

1.28. 8(9)-EET{d11} [M-H]1- 0001309

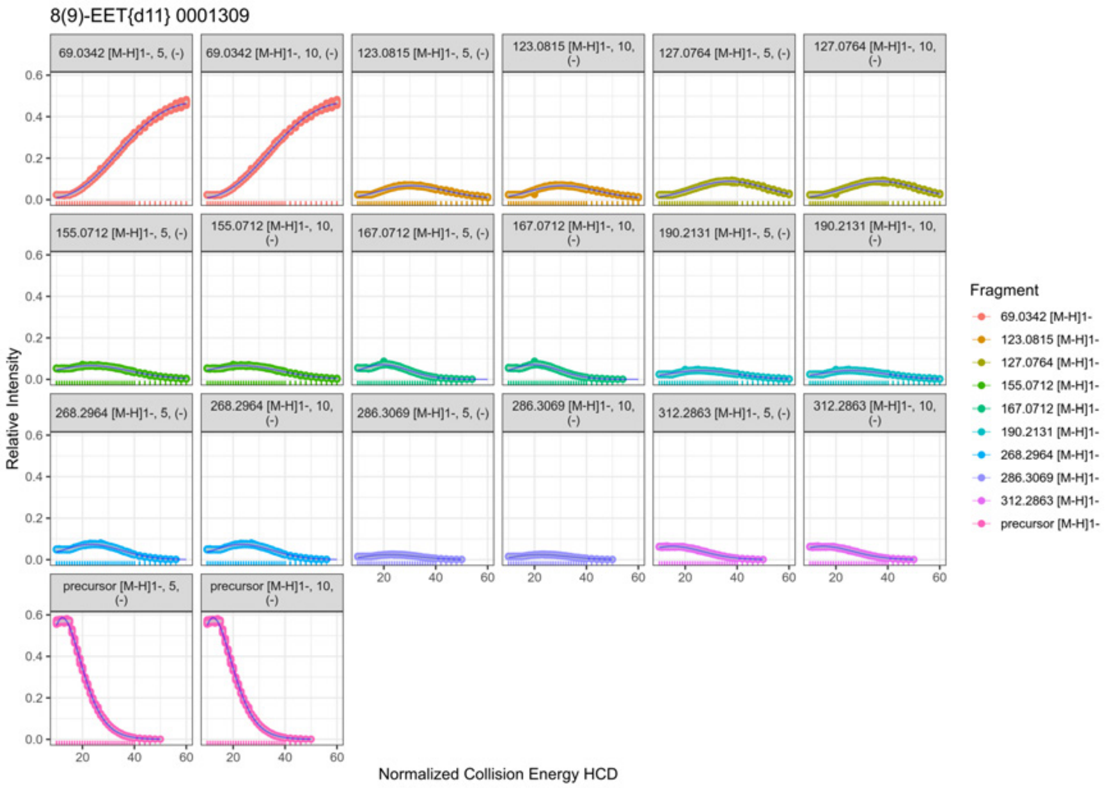

Figure 136. Nonlinear fit

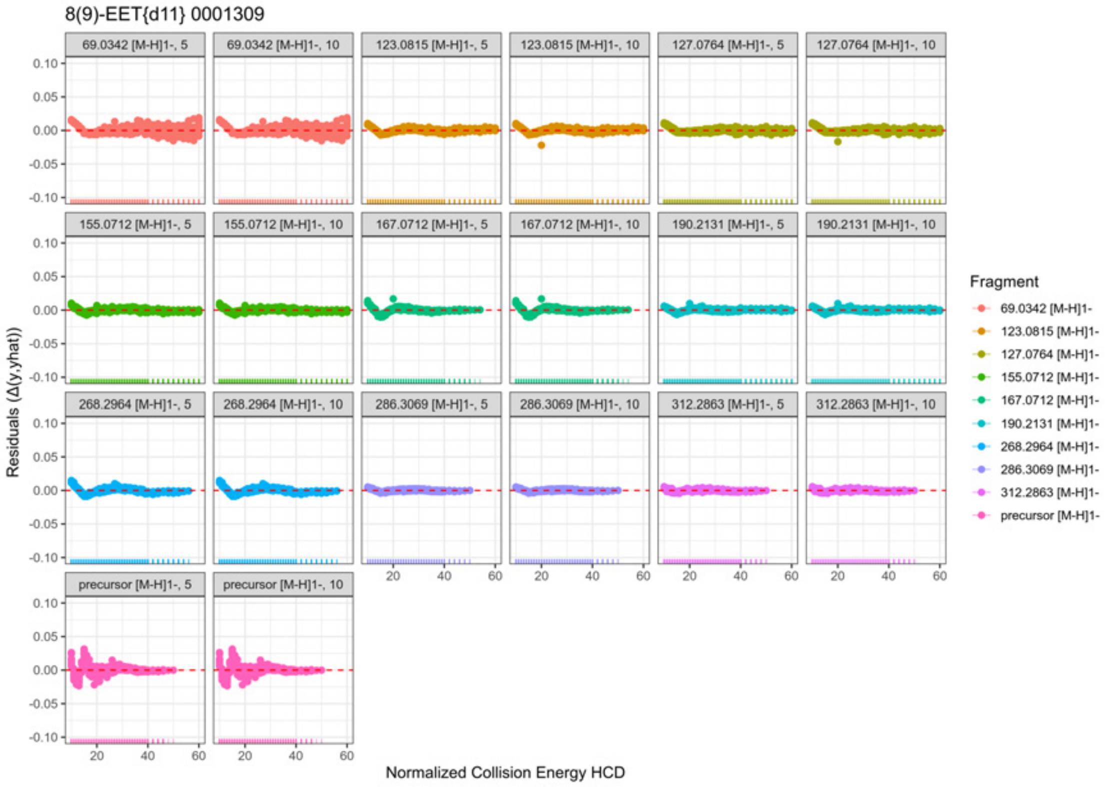

Figure 137. Residuals of nonlinear fit

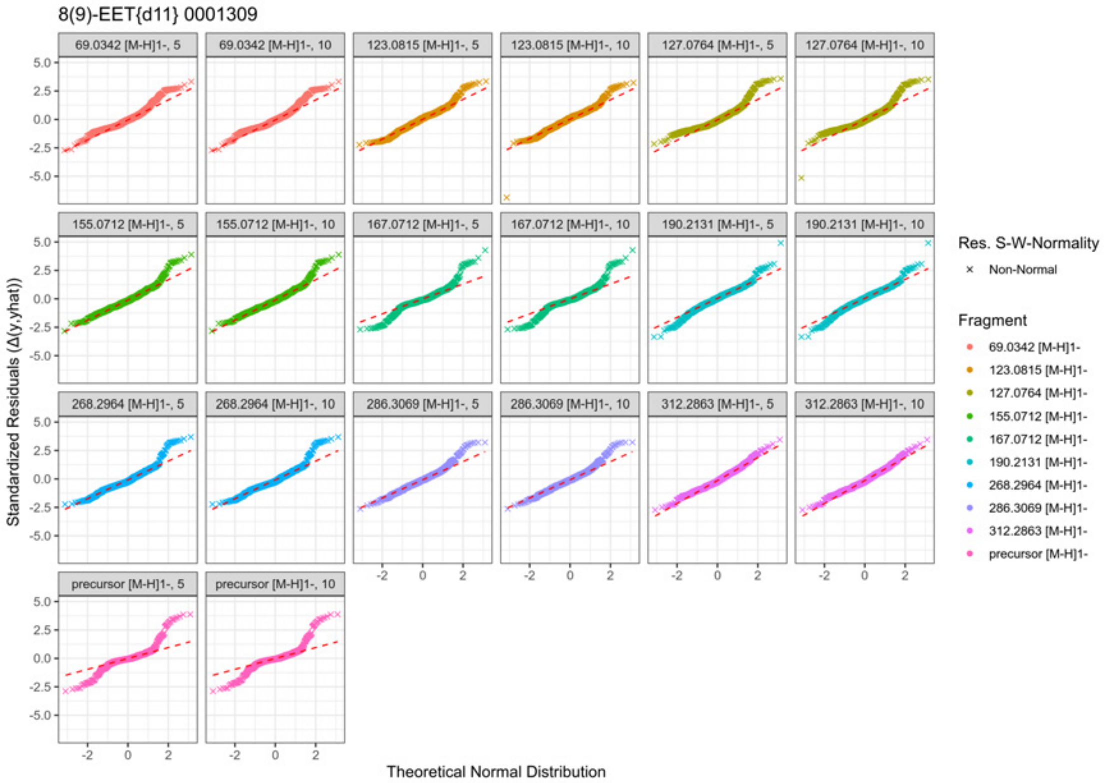

Figure 138. Quantile-quantile plot of residuals

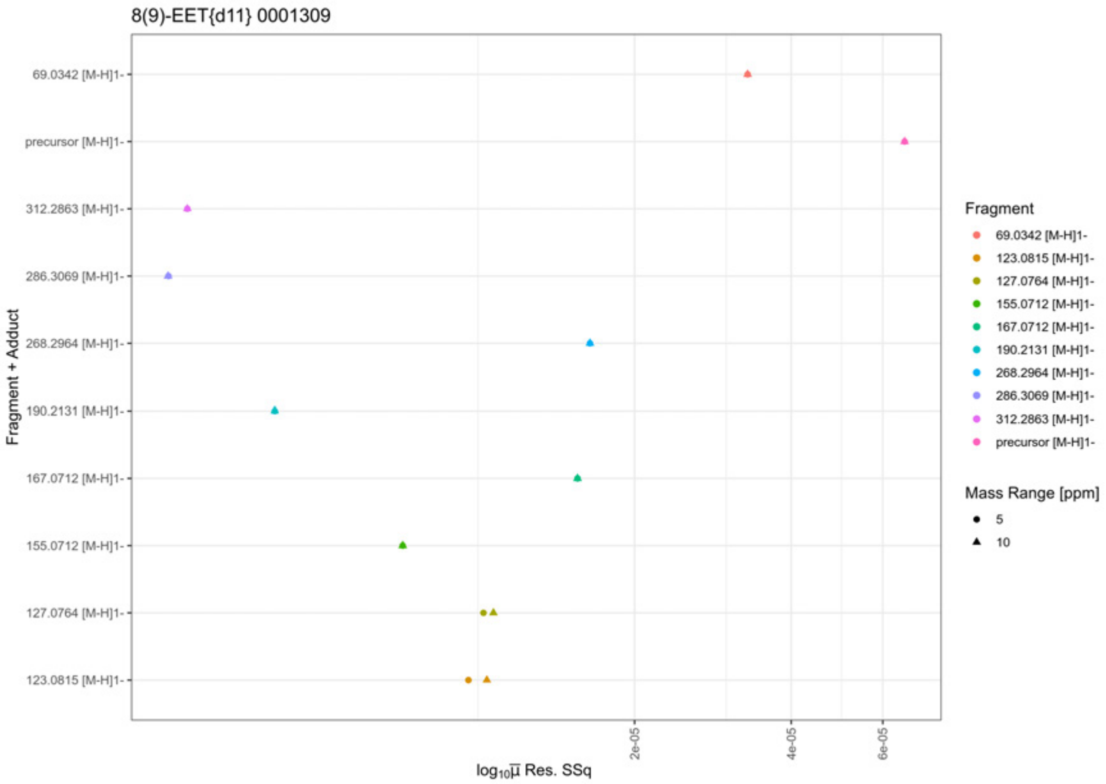

Figure 139. Normalized sum-of-squares of the residuals

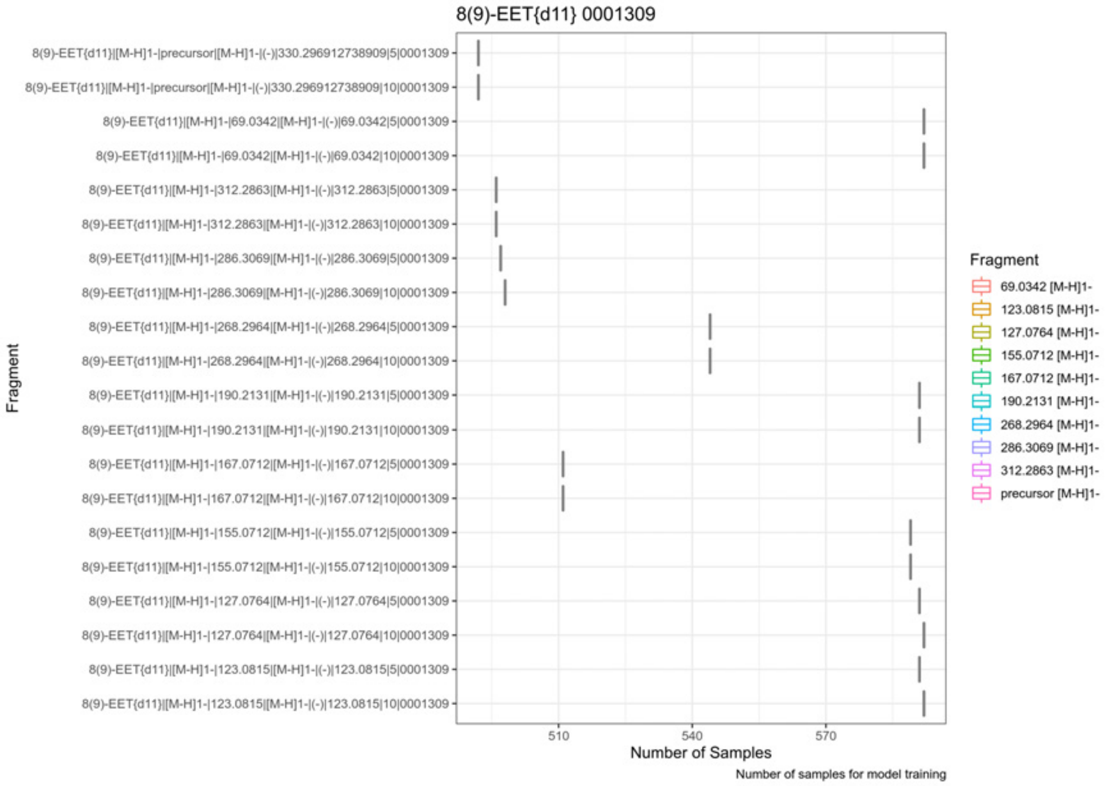

Figure 140. Number of samples used for training per combination Id

1.29. 8,9-DHET{d11} [M-H]1- 0000159

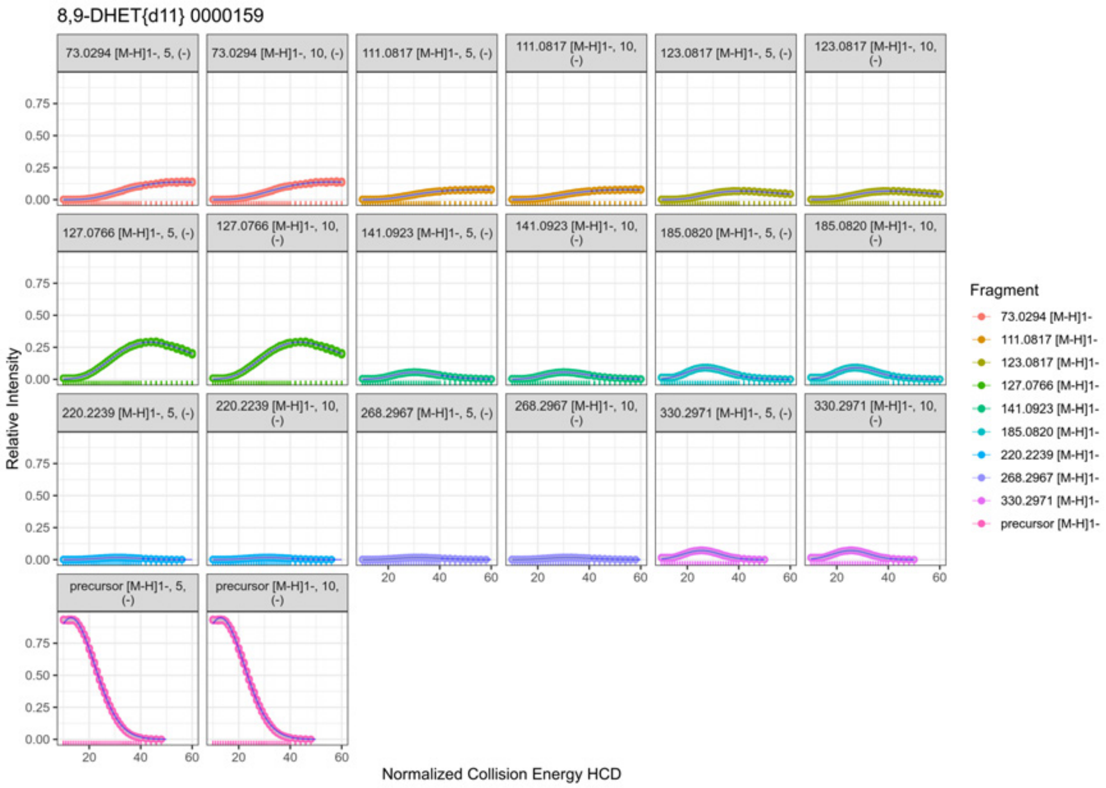

Figure 141. Nonlinear fit

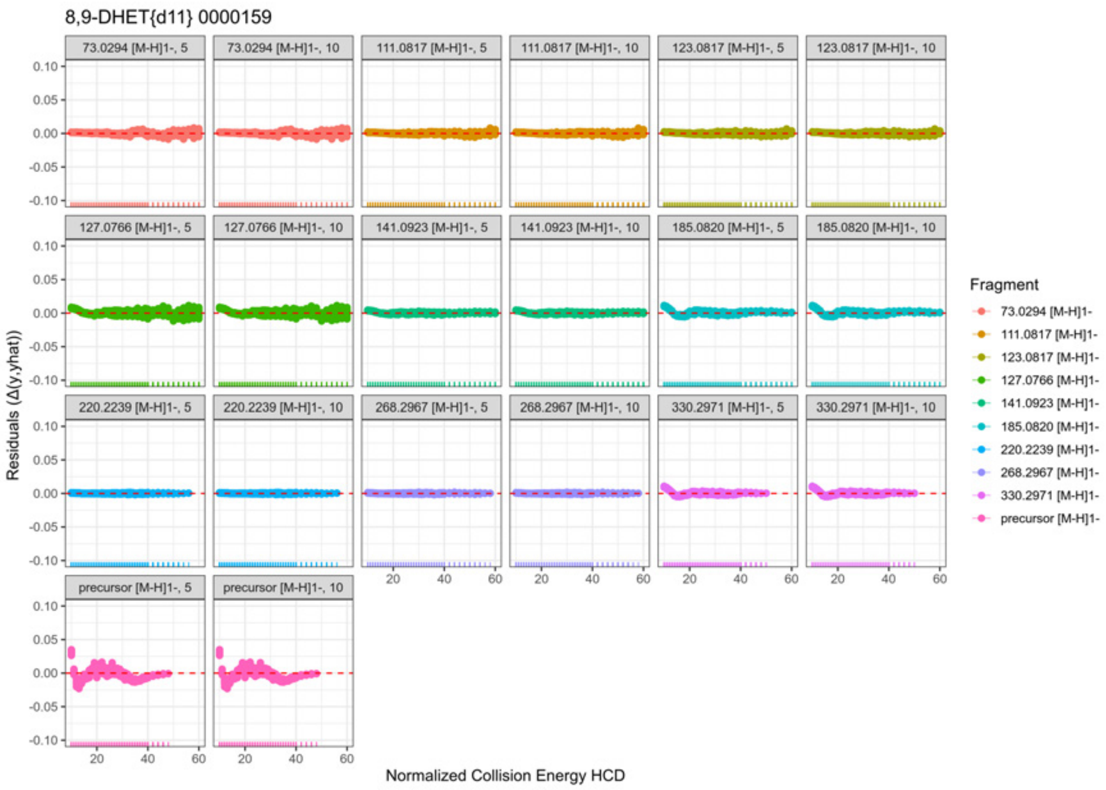

Figure 142. Residuals of nonlinear fit

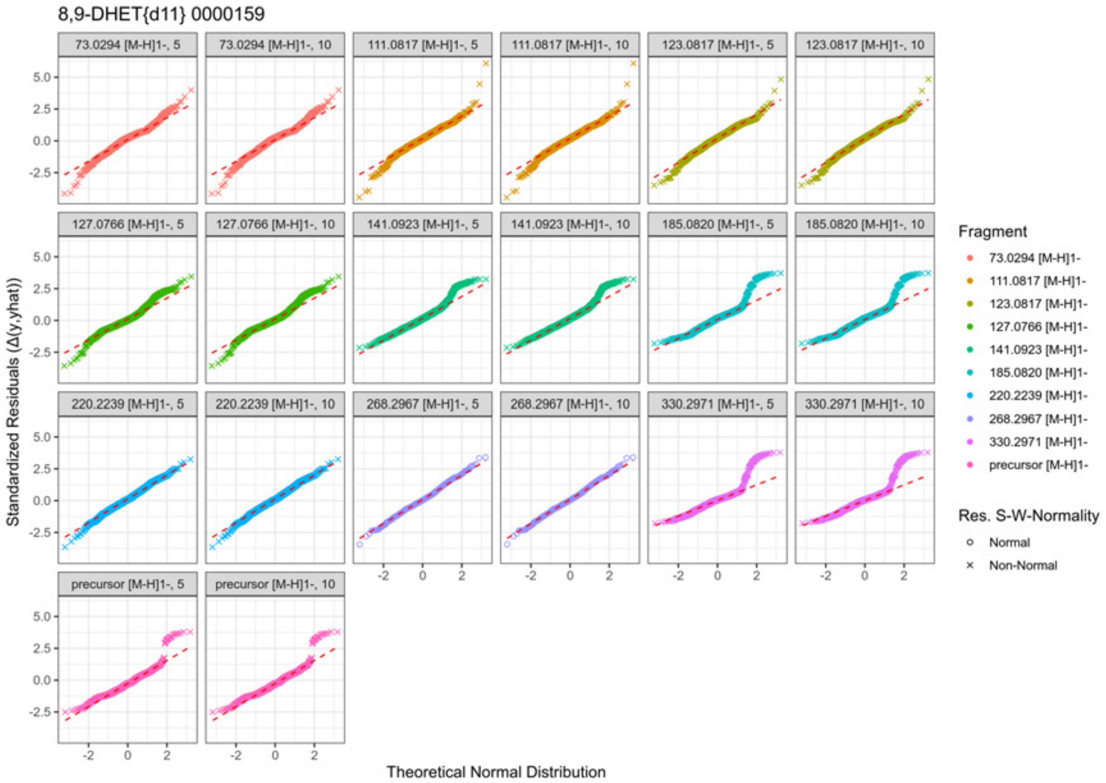

Figure 143. Quantile-quantile plot of residuals

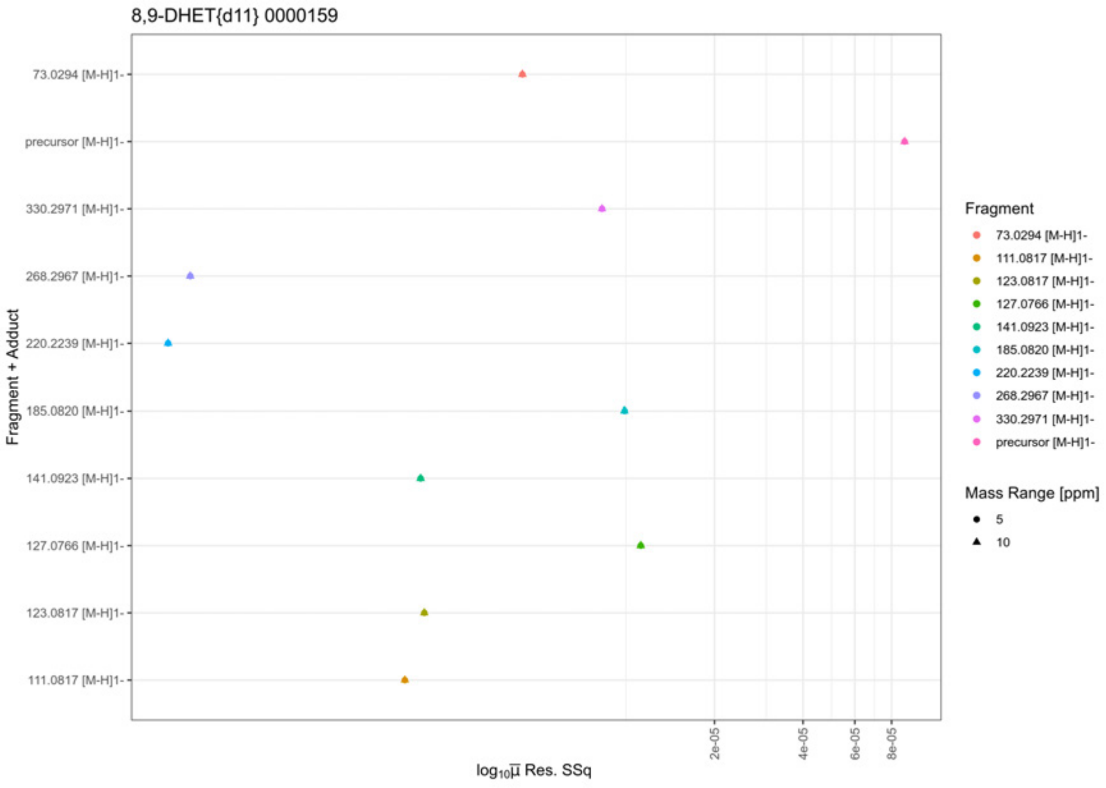

Figure 144. Normalized sum-of-squares of the residuals

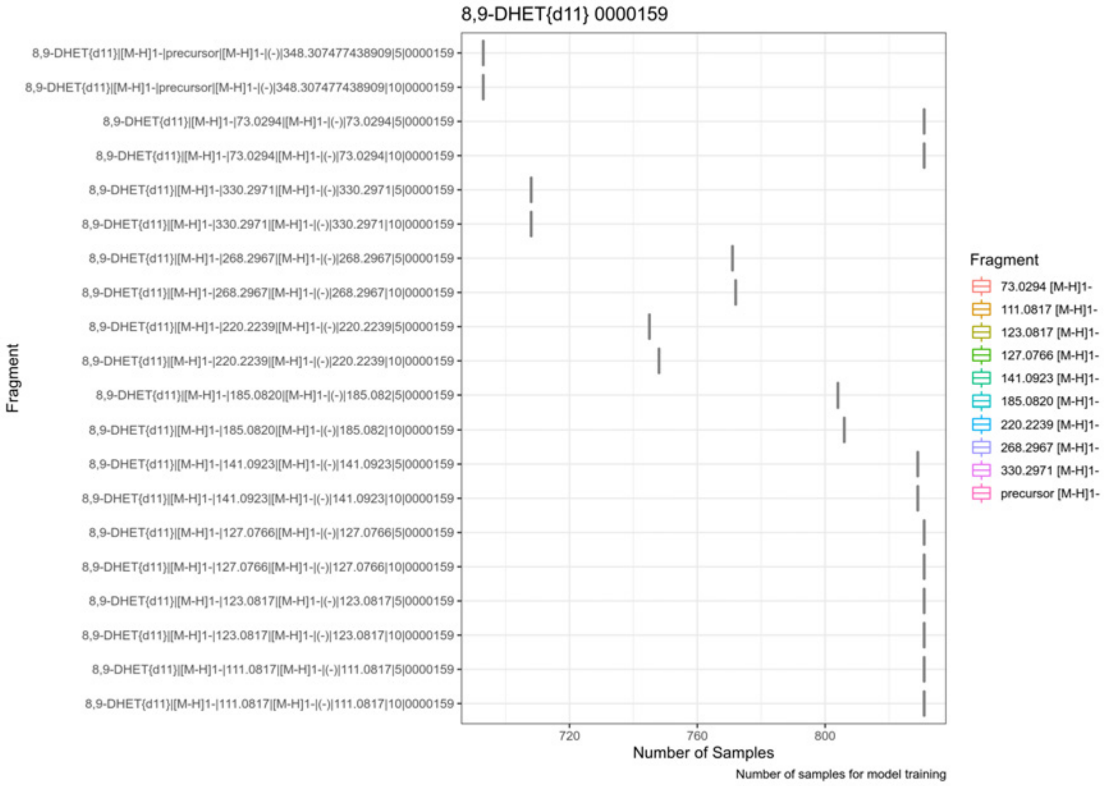

Figure 145. Number of samples used for training per combination Id

1.30. 8-HDoHE [M-H]1- 0001273

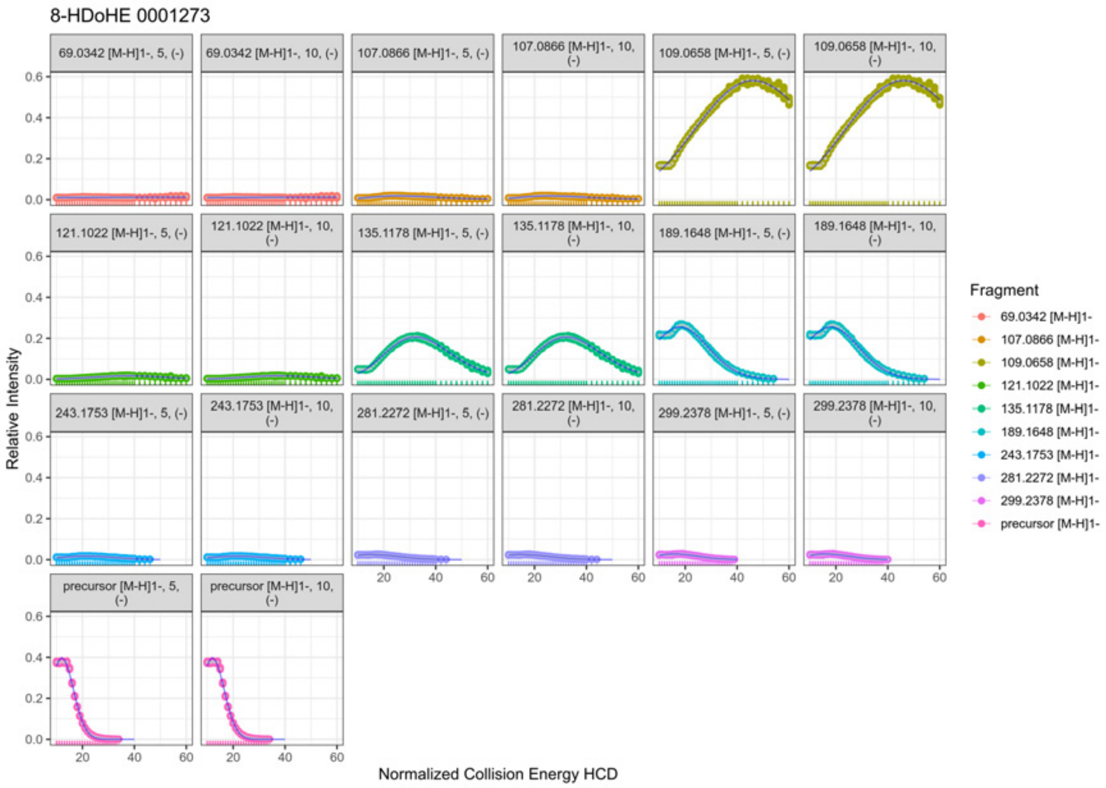

Figure 146. Nonlinear fit

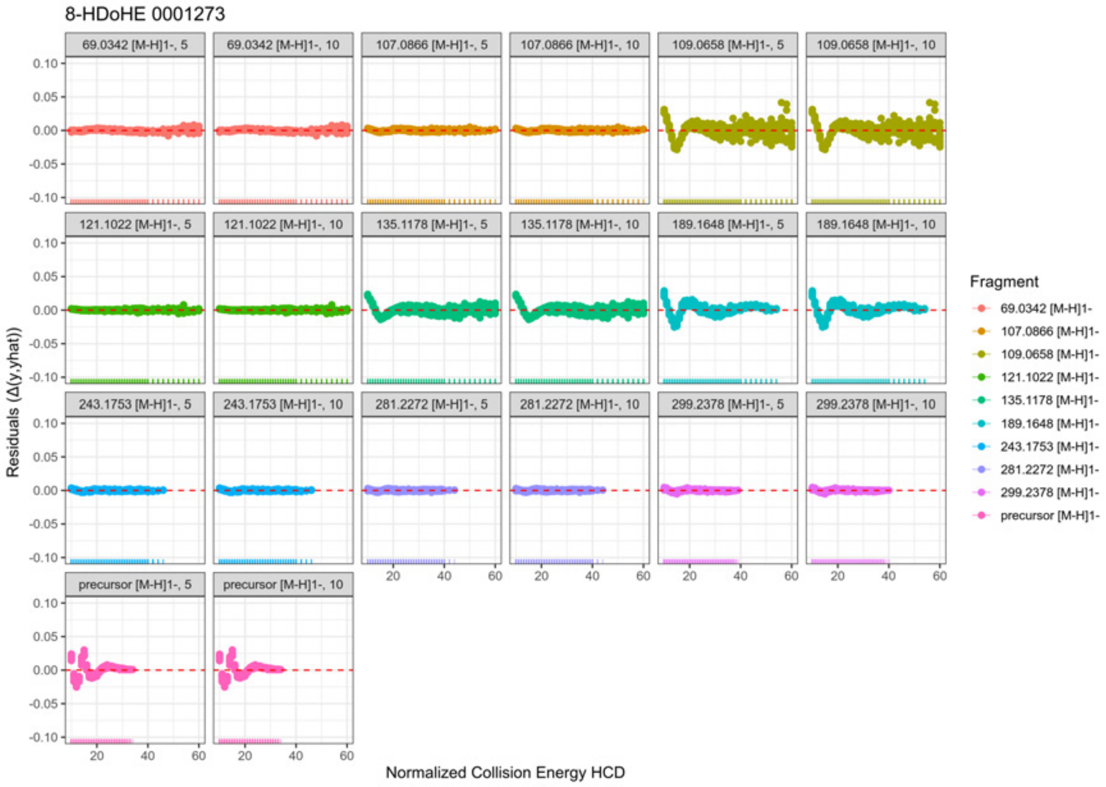

Figure 147. Residuals of nonlinear fit

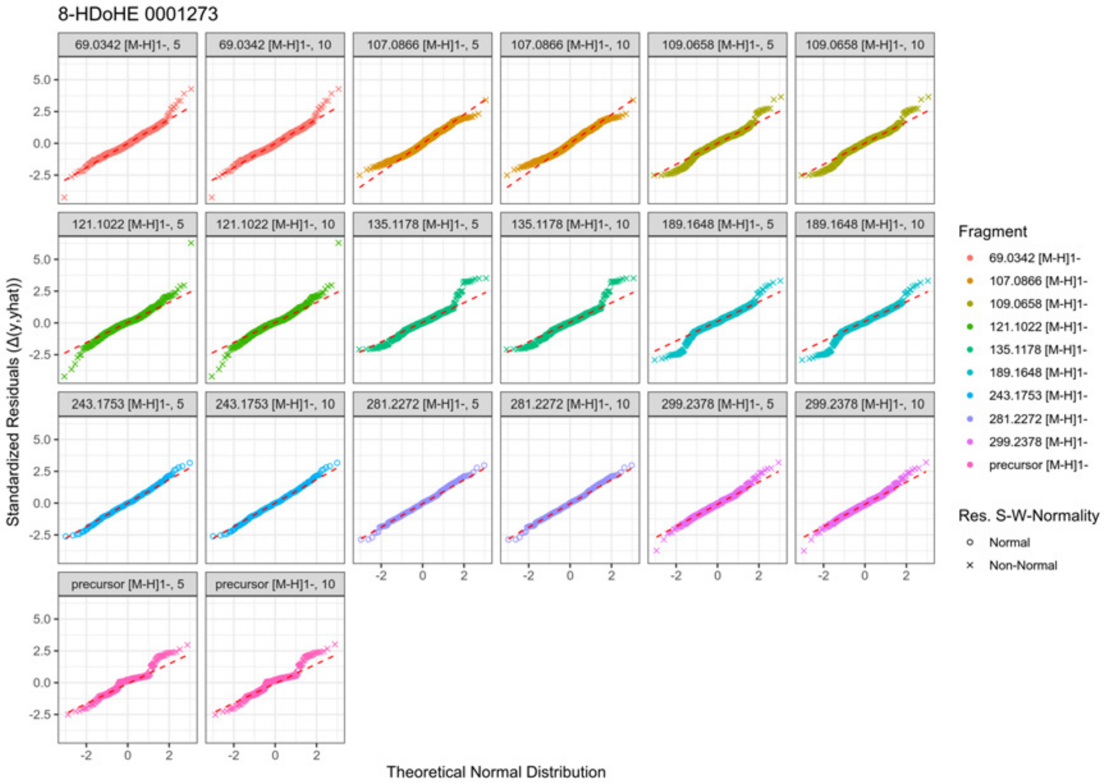

Figure 148. Quantile-quantile plot of residuals

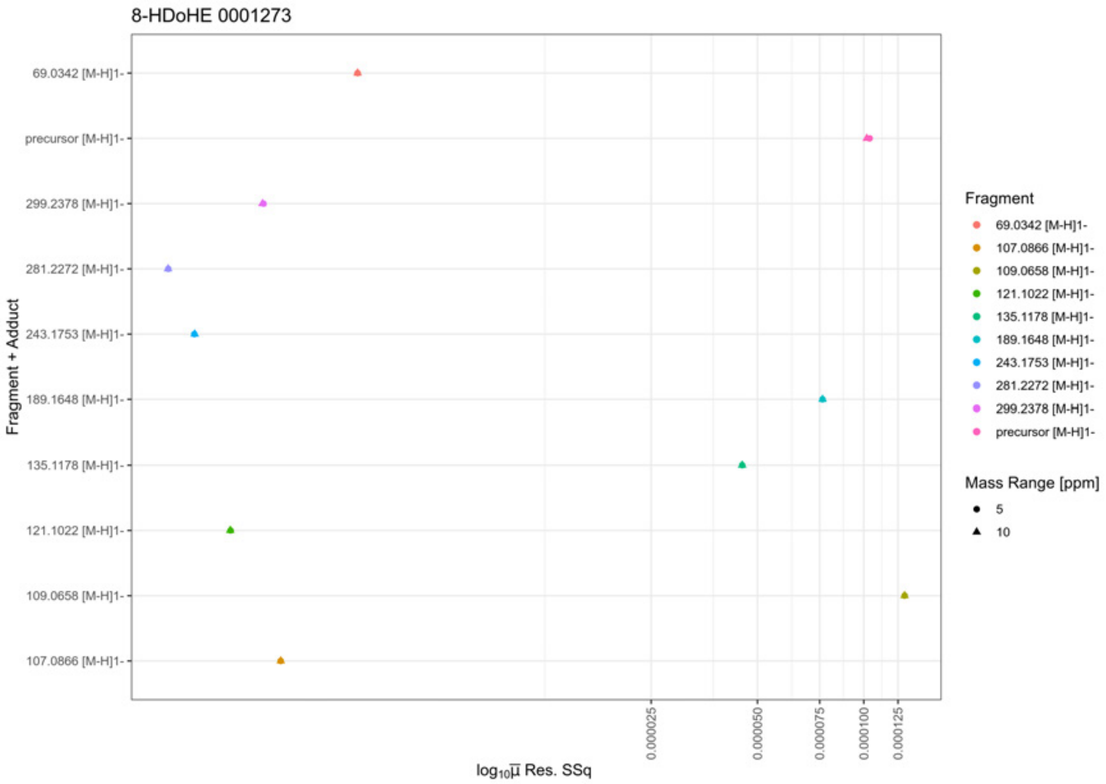

Figure 149. Normalized sum-of-squares of the residuals

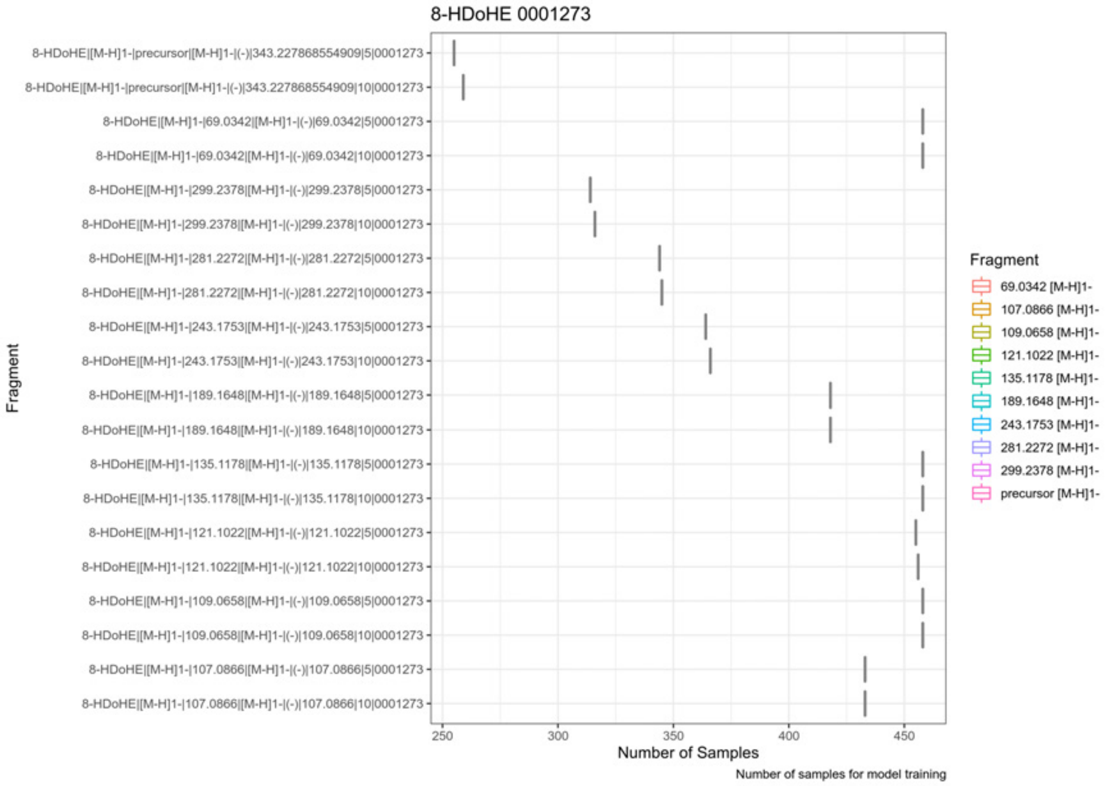

Figure 150. Number of samples used for training per combination Id

1.31. 8-HETE [M-H]1- 0001287

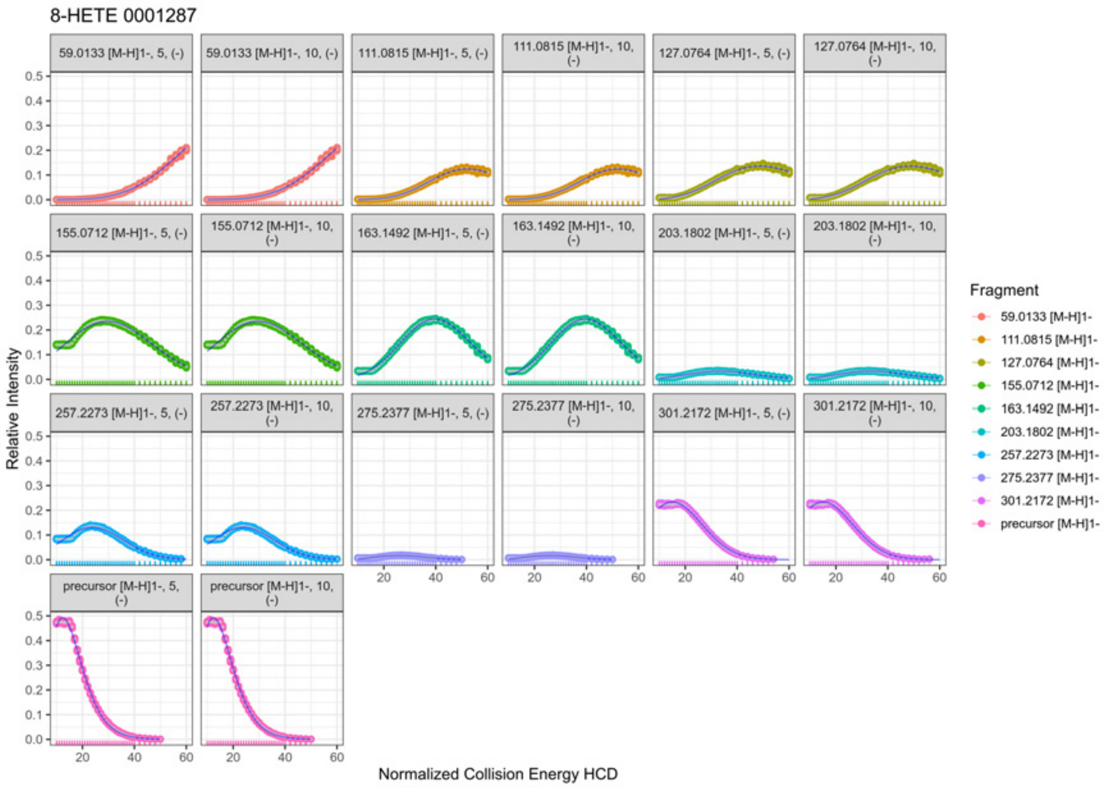

Figure 151. Nonlinear fit

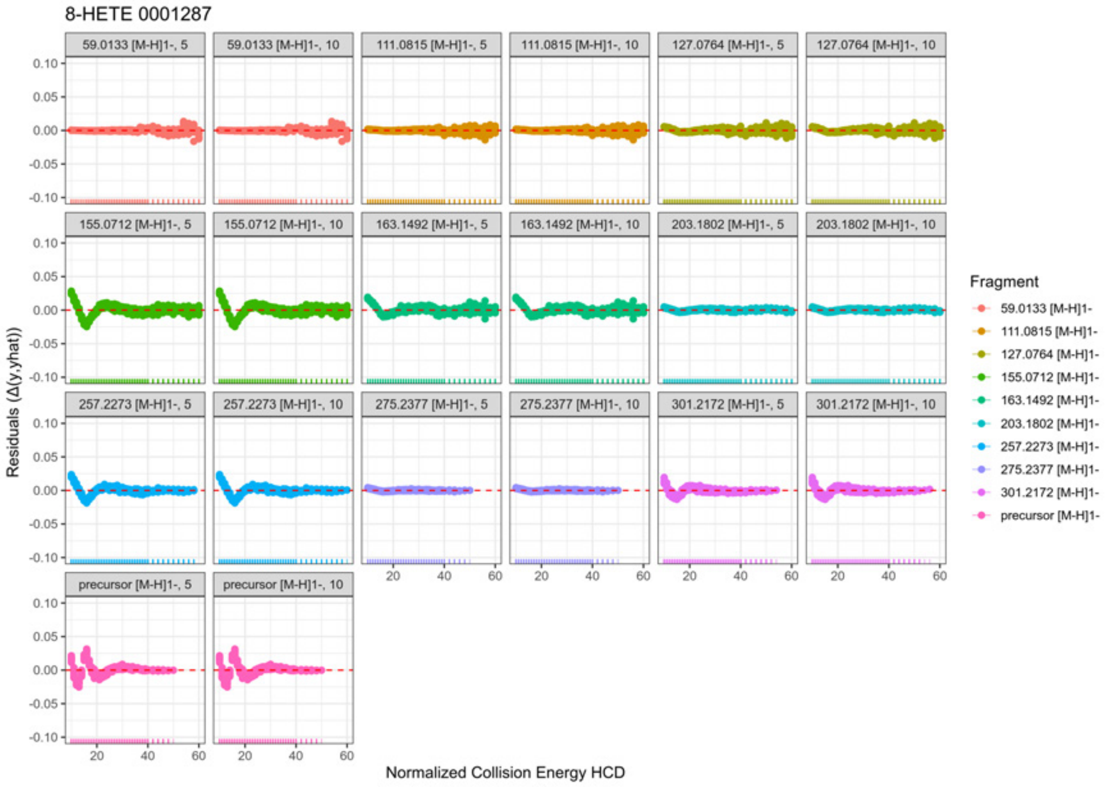

Figure 152. Residuals of nonlinear fit

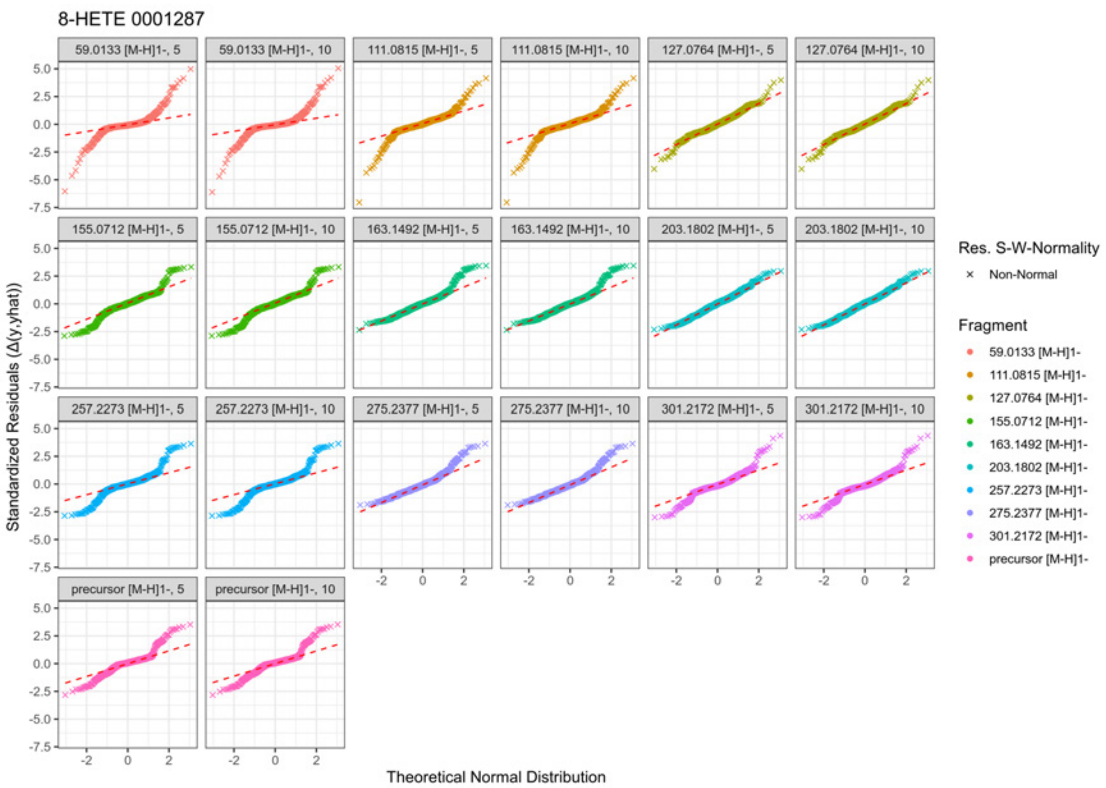

Figure 153. Quantile-quantile plot of residuals

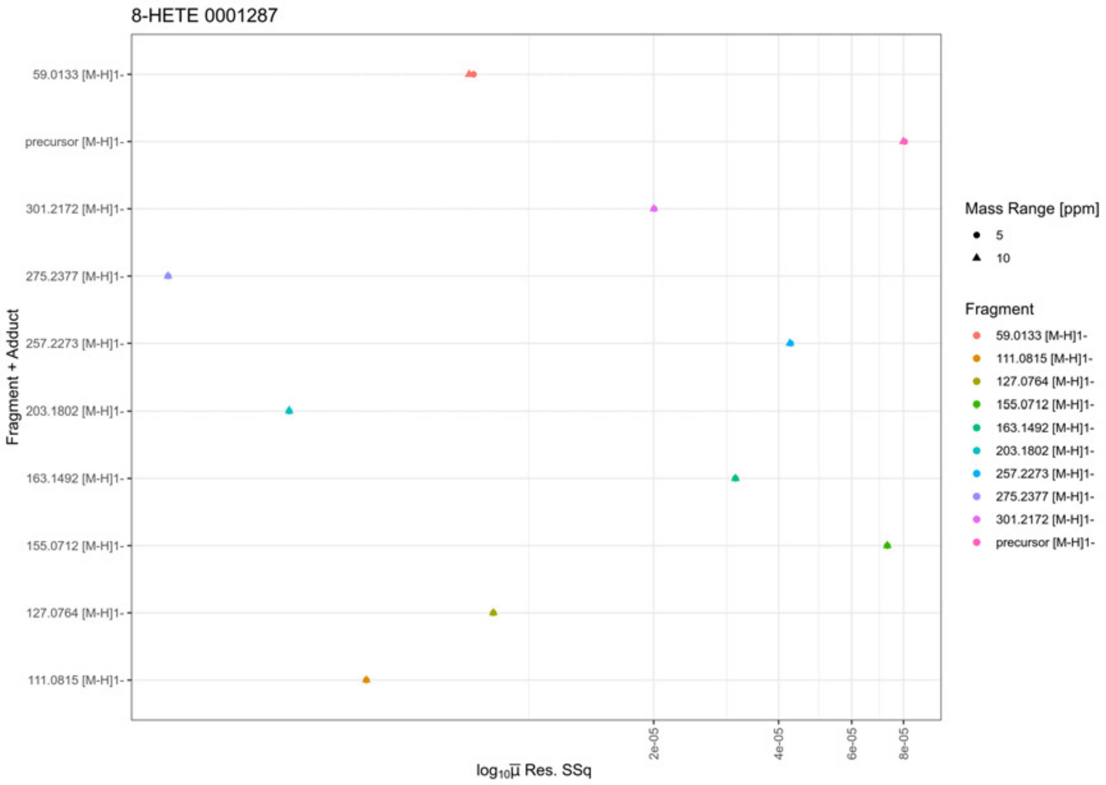

Figure 154. Normalized sum-of-squares of the residuals

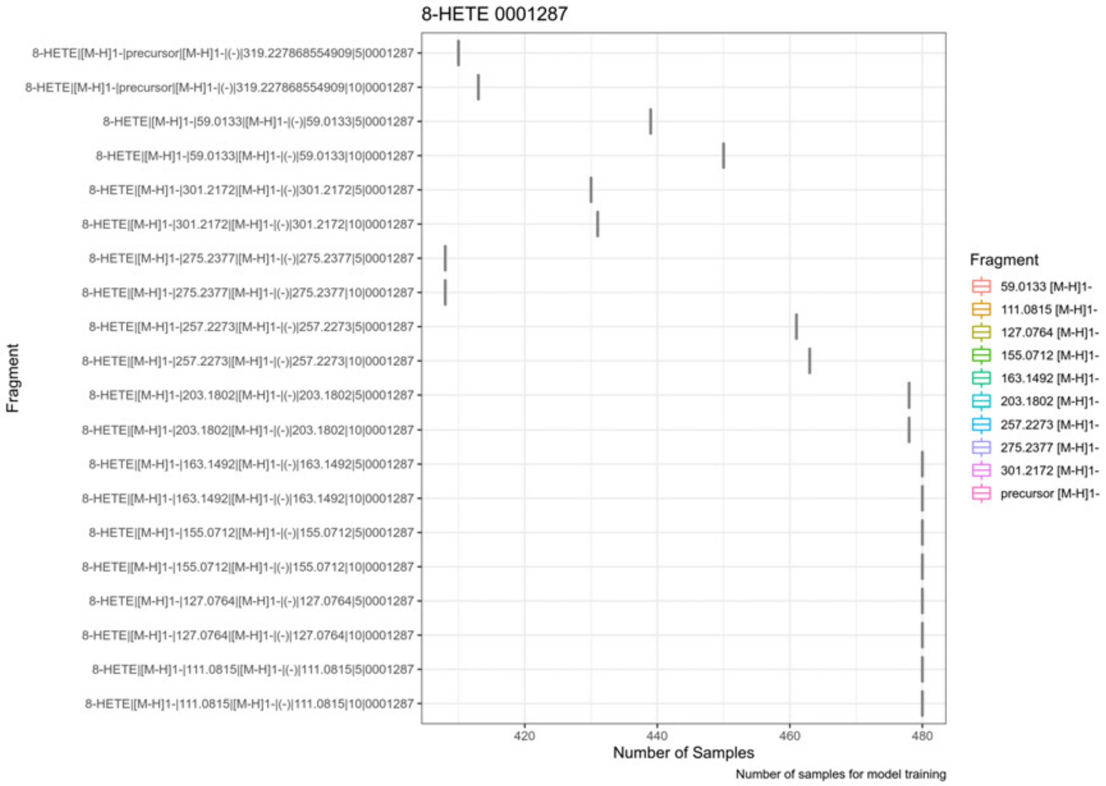

Figure 155. Number of samples used for training per combination Id

1.32. 9(10)-EpOME{d4} [M-H]1- 0001355

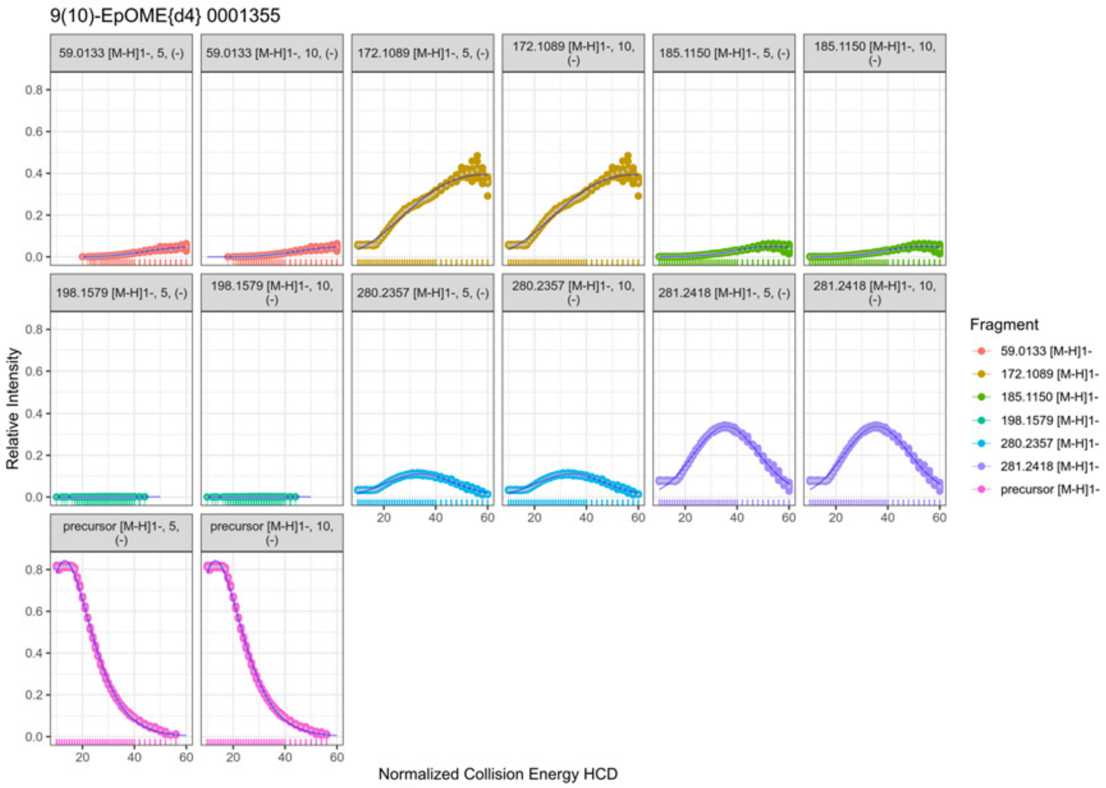

Figure 156. Nonlinear fit

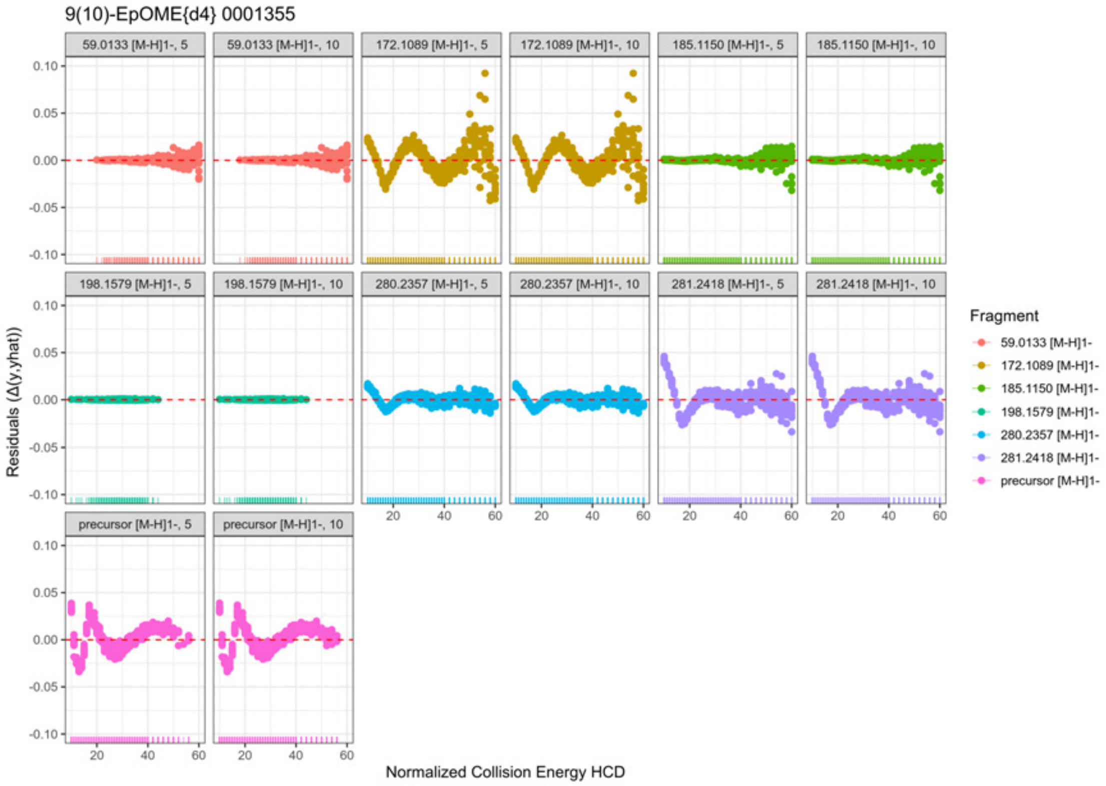

Figure 157. Residuals of nonlinear fit

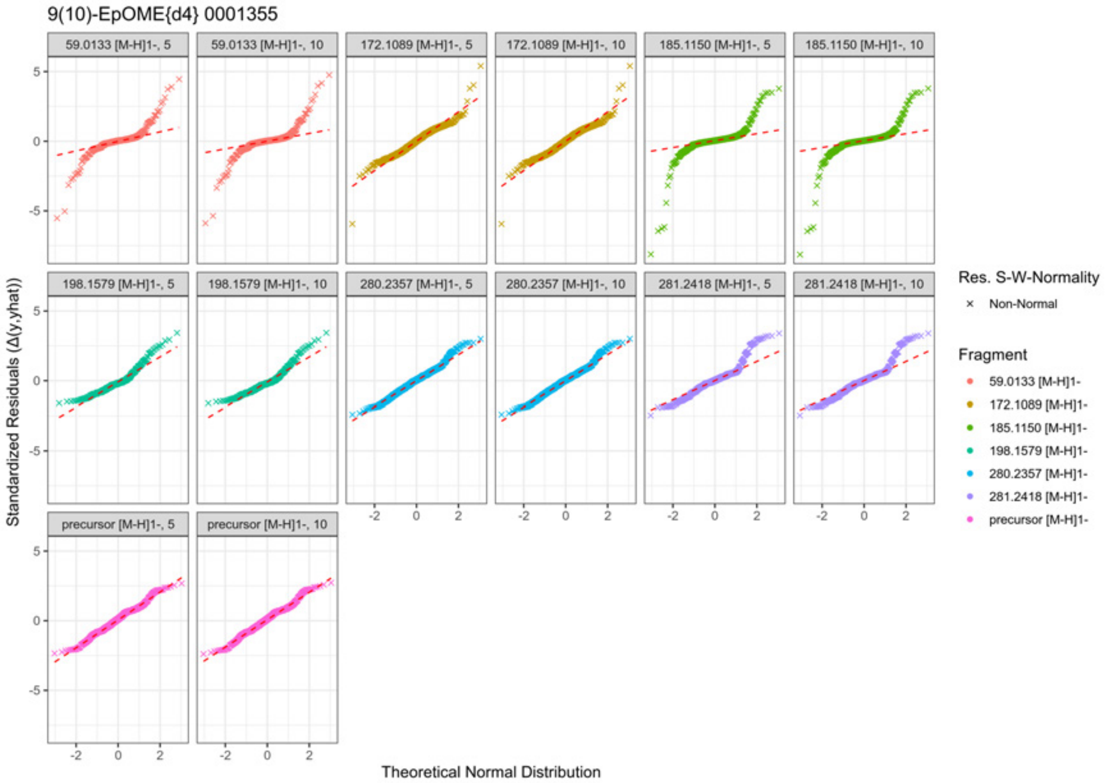

Figure 158. Quantile-quantile plot of residuals

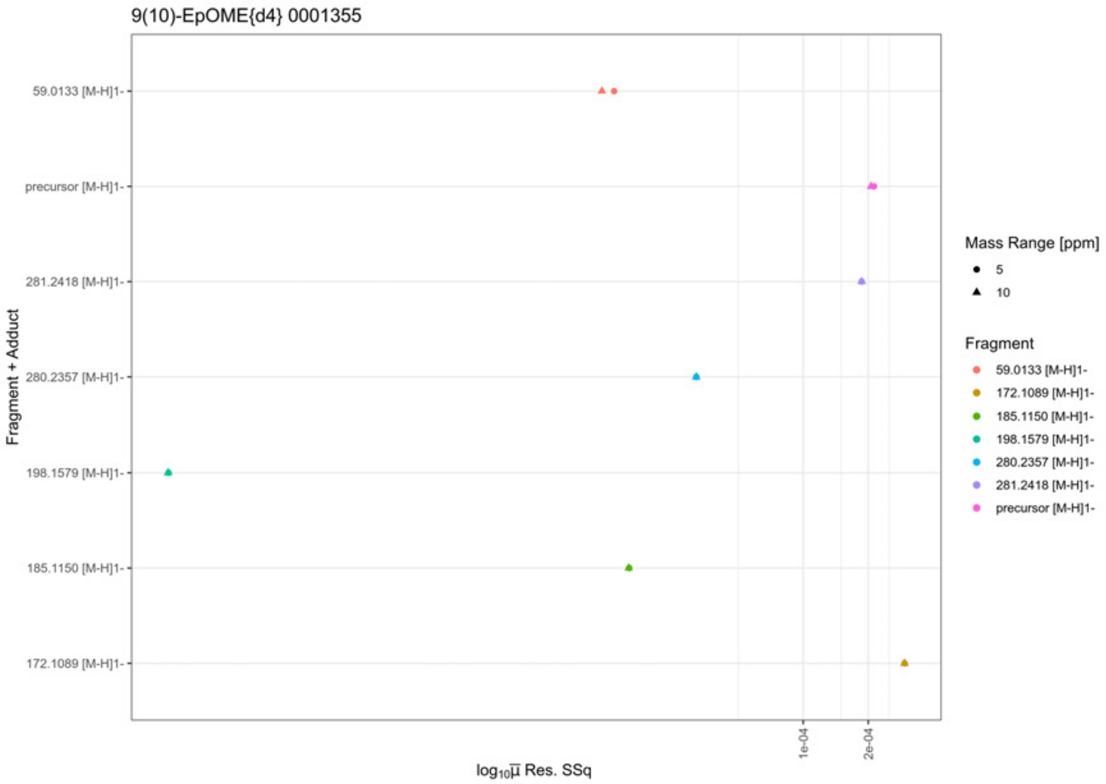

Figure 159. Normalized sum-of-squares of the residuals

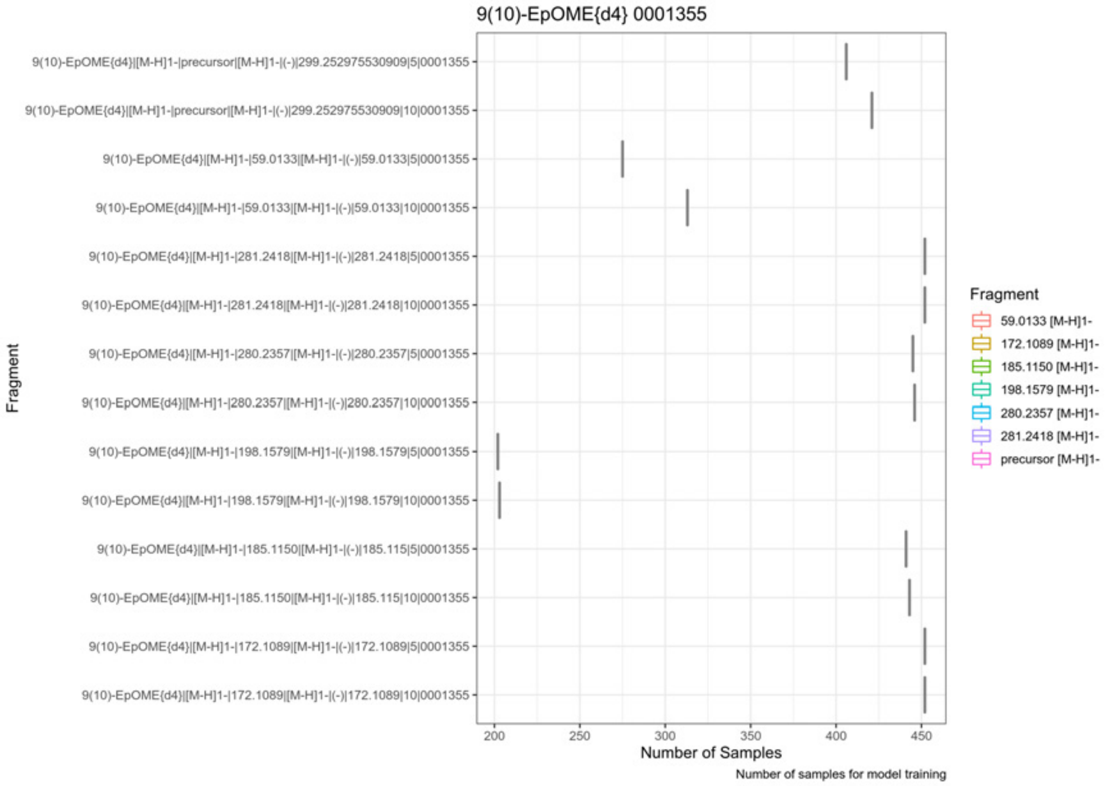

Figure 160. Number of samples used for training per combination Id

1.33. 9-HEPE [M-H]1- 0001279

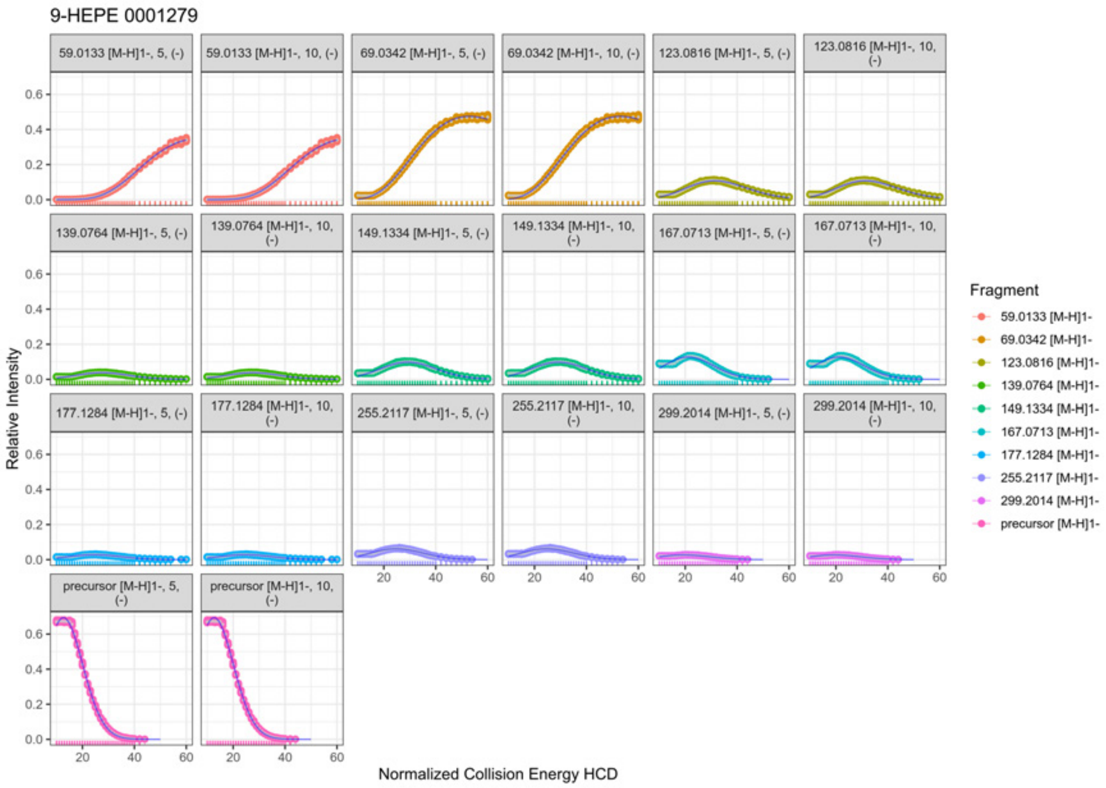

Figure 161. Nonlinear fit

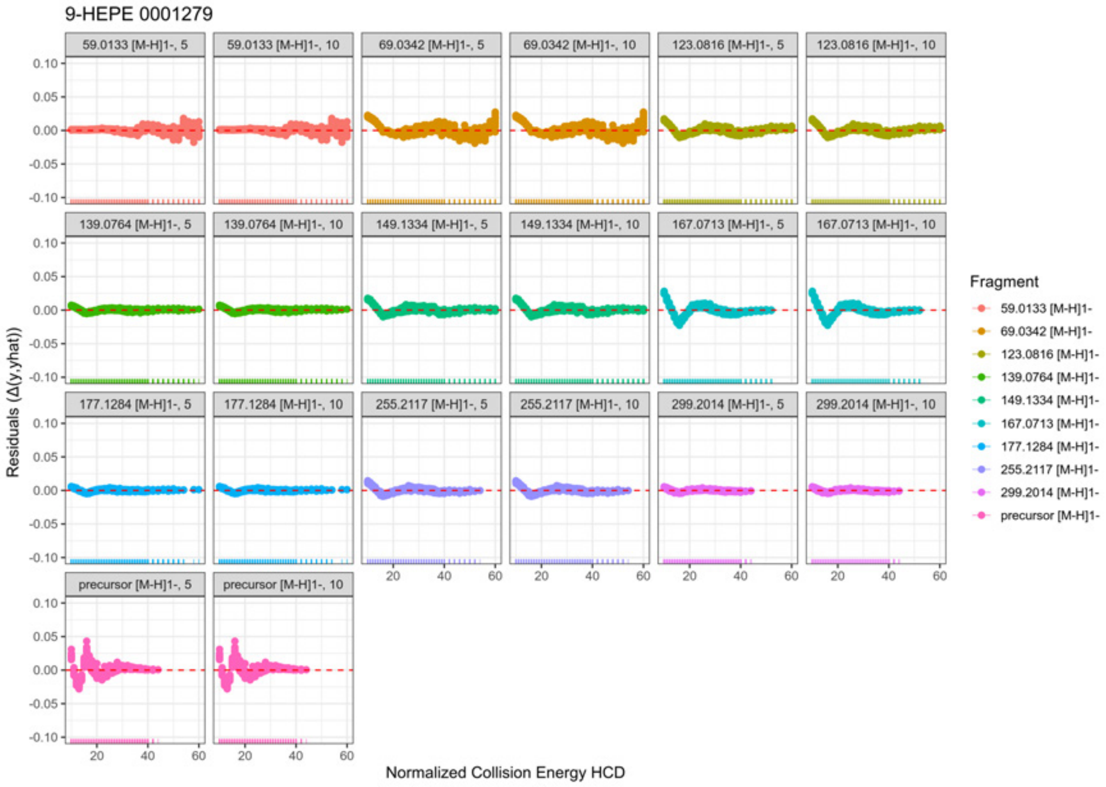

Figure 162. Residuals of nonlinear fit

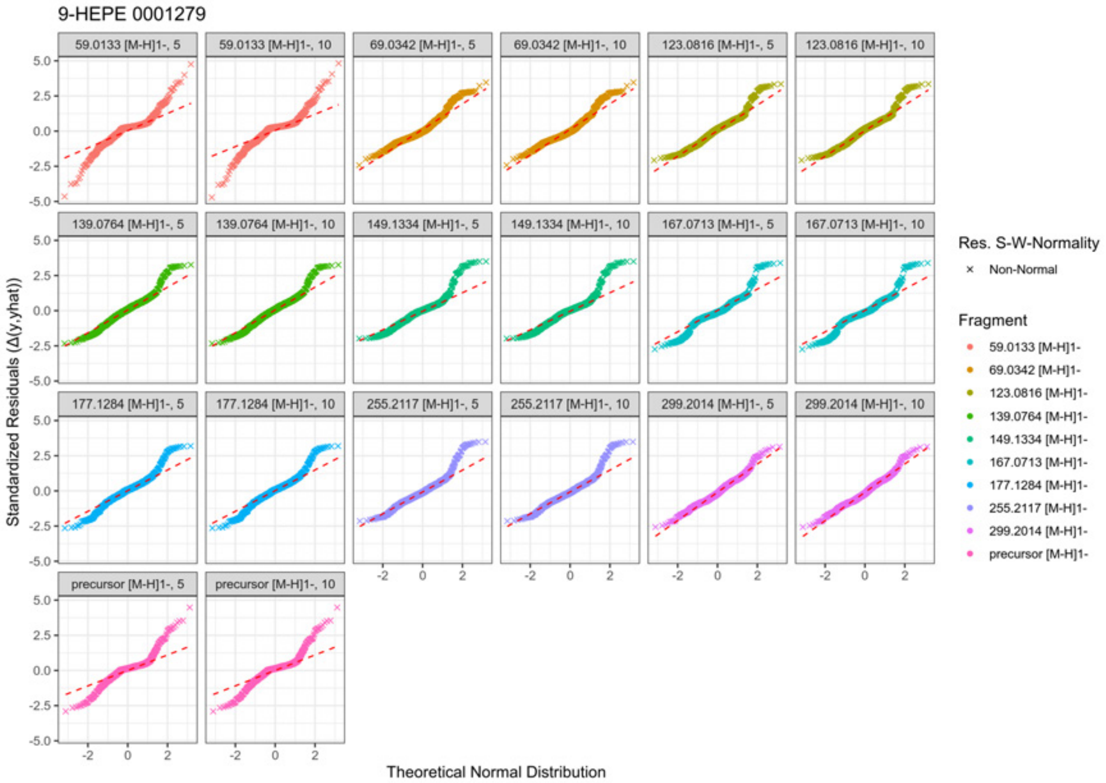

Figure 163. Quantile-quantile plot of residuals

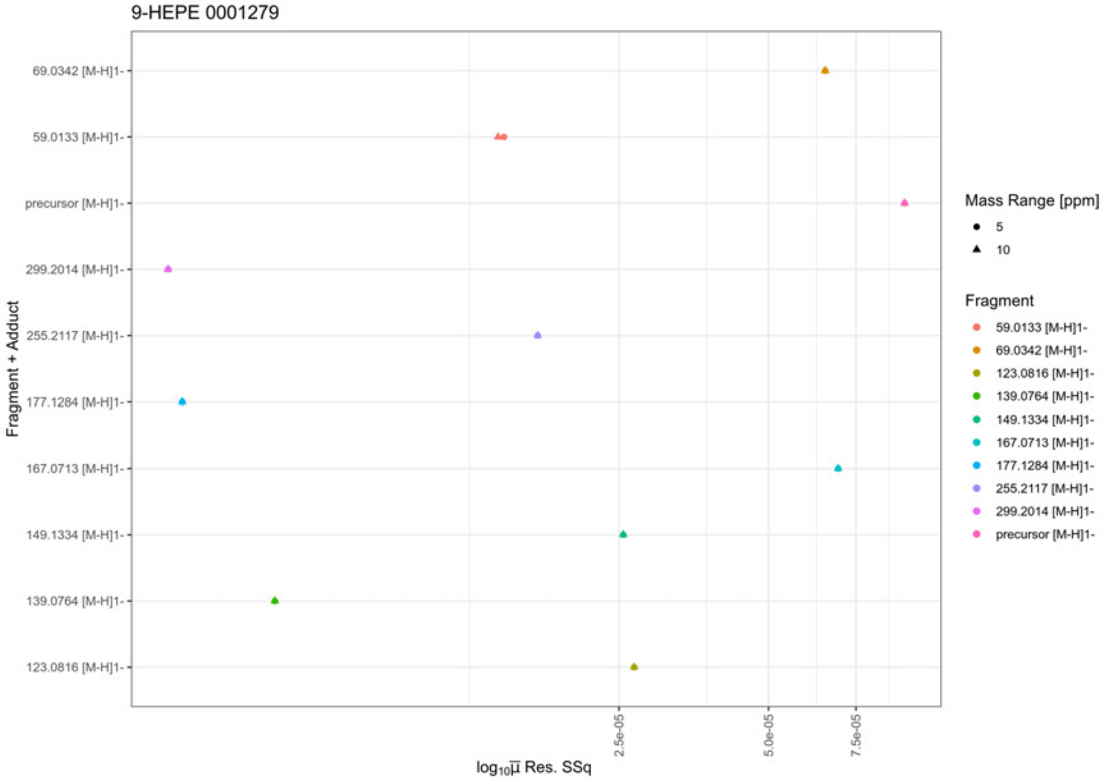

Figure 164. Normalized sum-of-squares of the residuals

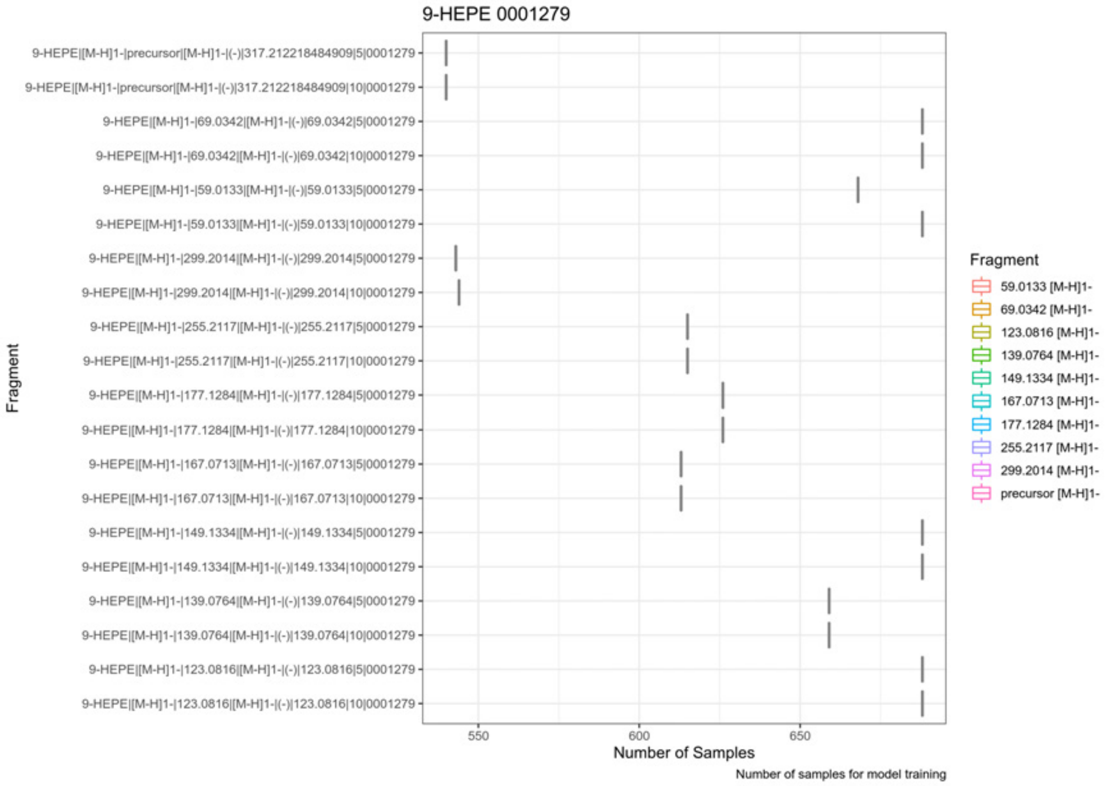

Figure 165. Number of samples used for training per combination Id

1.34. 9-HETE [M-H]1- 0001291

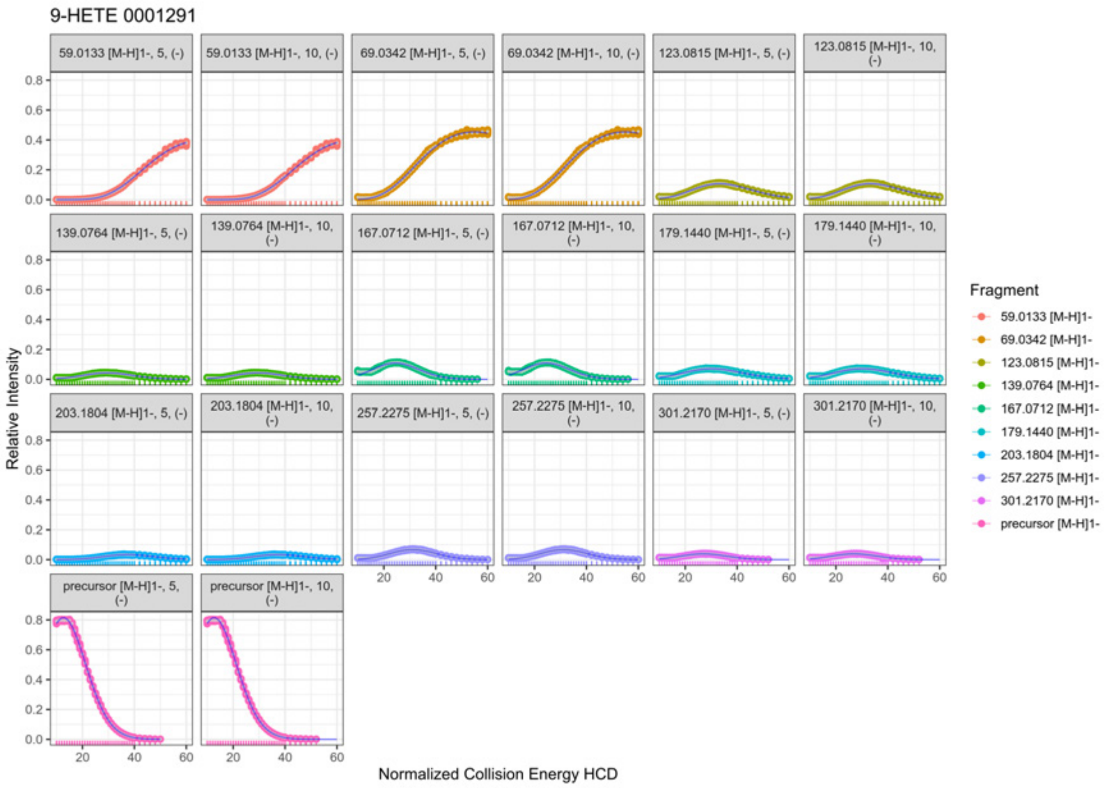

Figure 166. Nonlinear fit

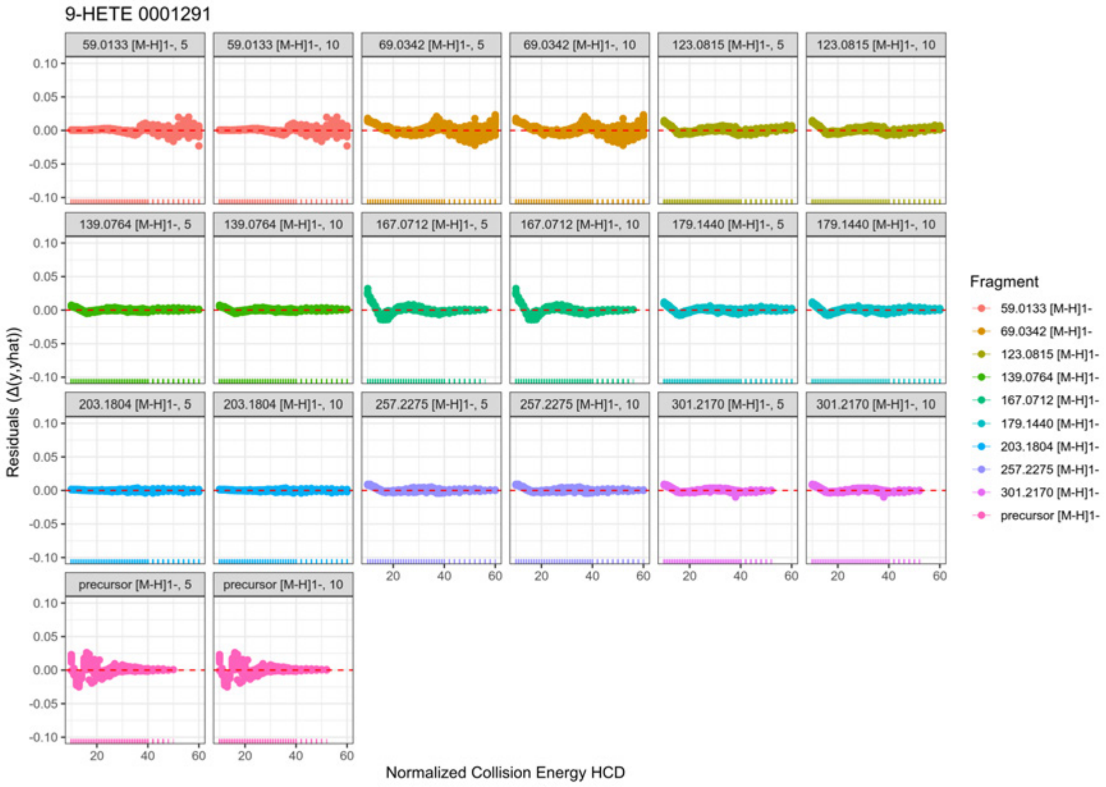

Figure 167. Residuals of nonlinear fit

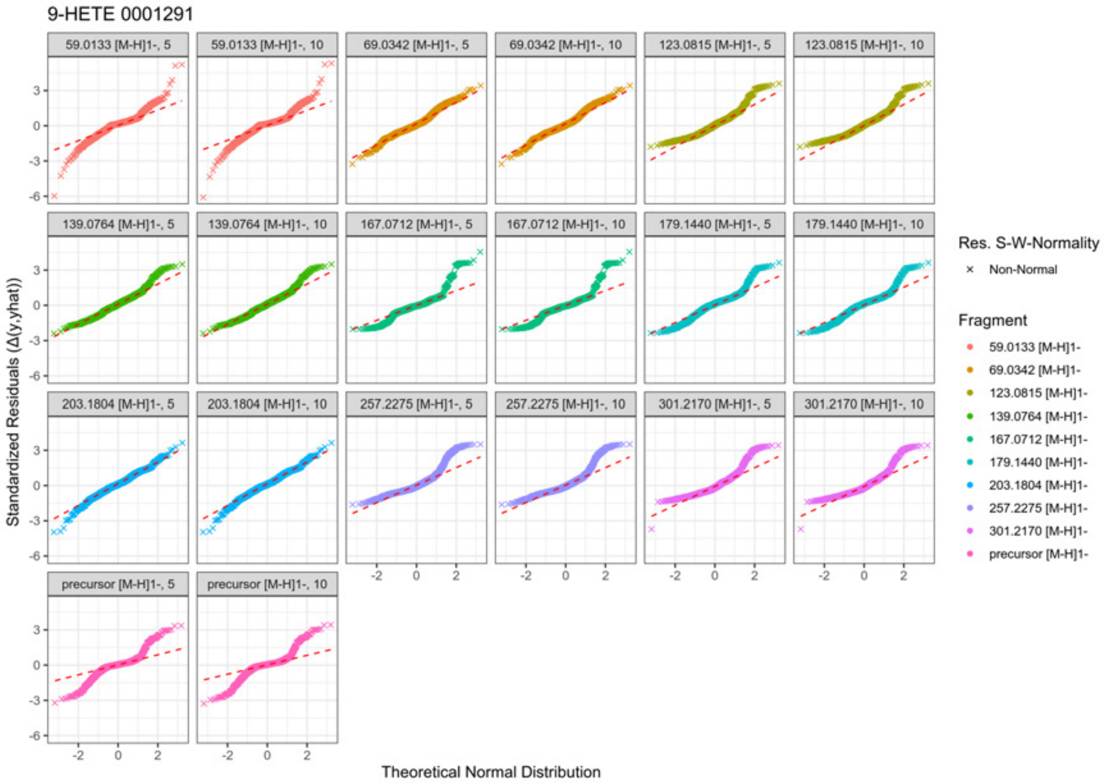

Figure 168. Quantile-quantile plot of residuals

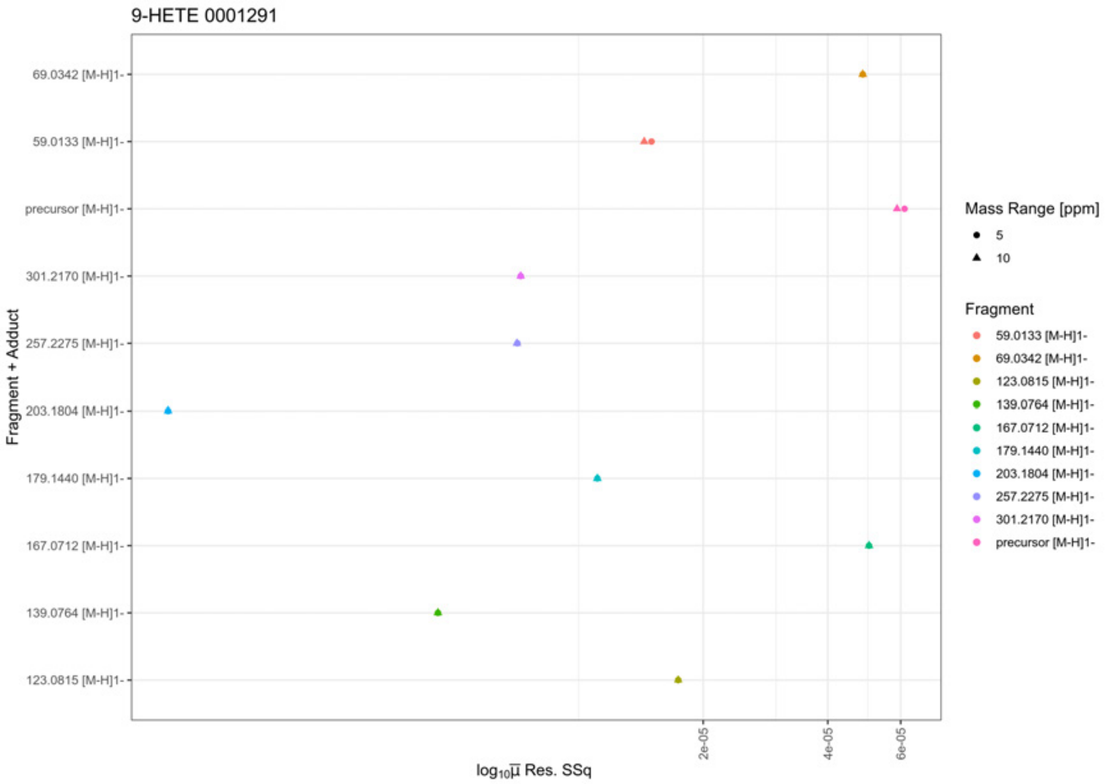

Figure 169. Normalized sum-of-squares of the residuals

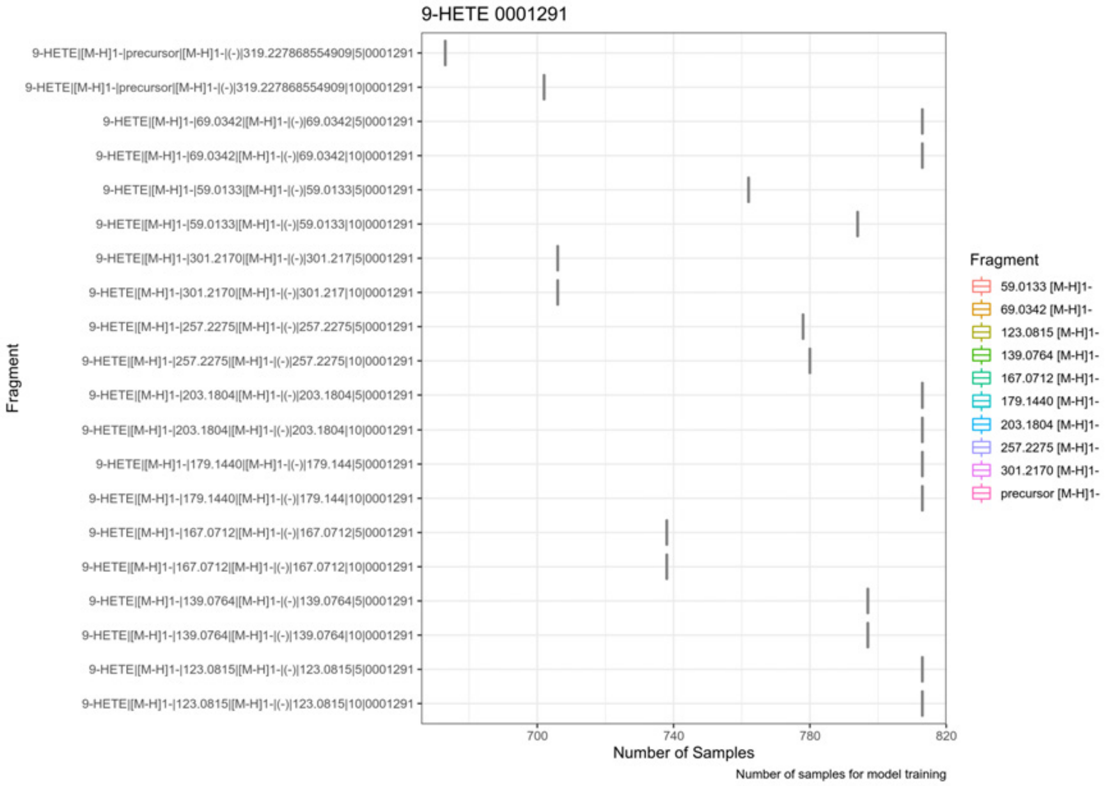

Figure 170. Number of samples used for training per combination Id

1.35. 9-HODE [M-H]1- 0001339

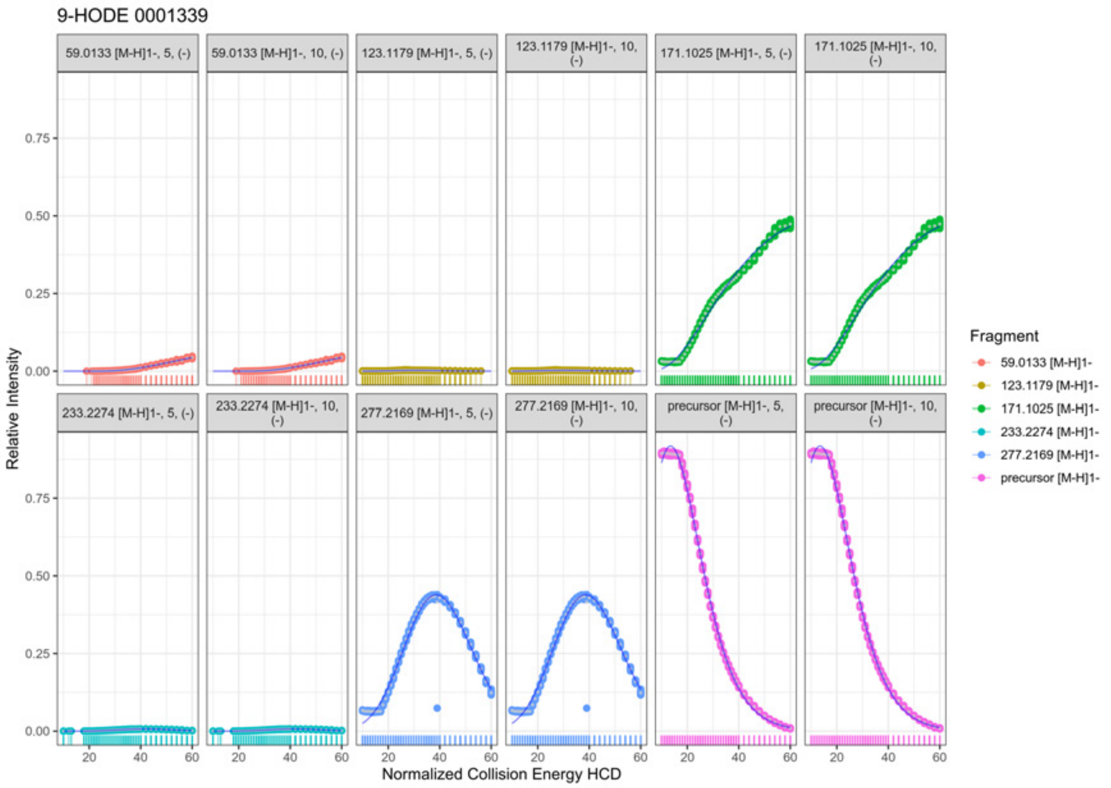

Figure 171. Nonlinear fit

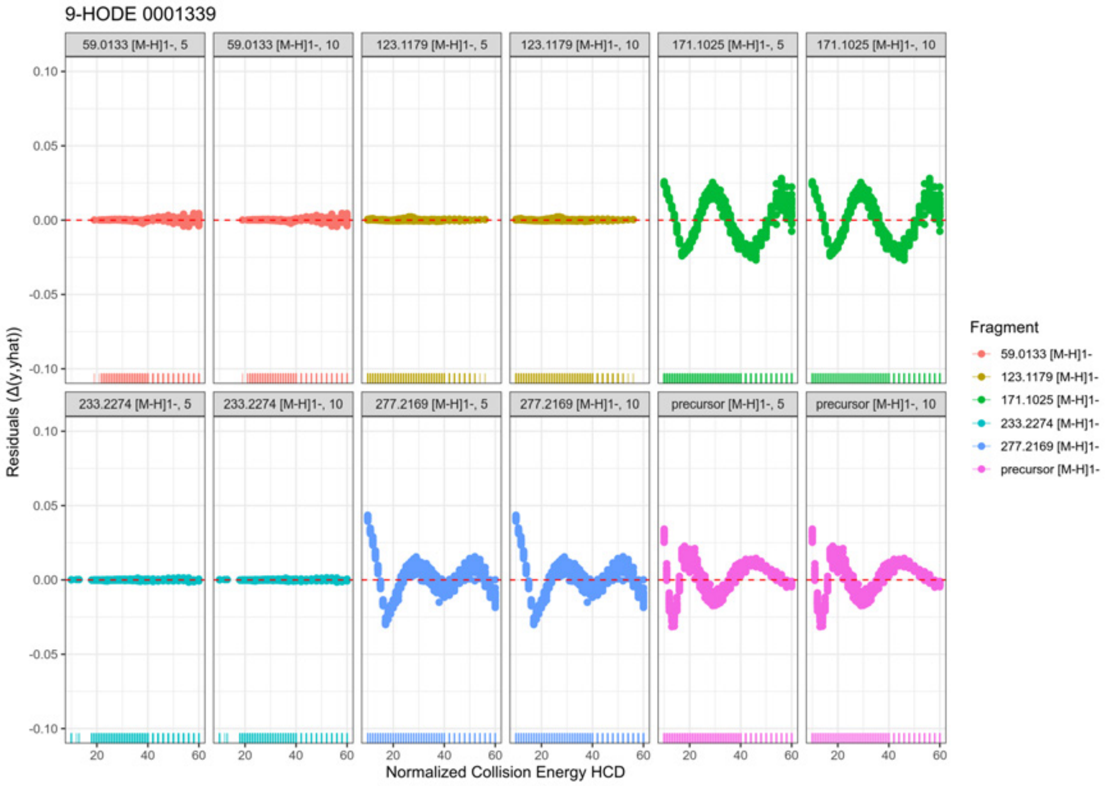

Figure 172. Residuals of nonlinear fit

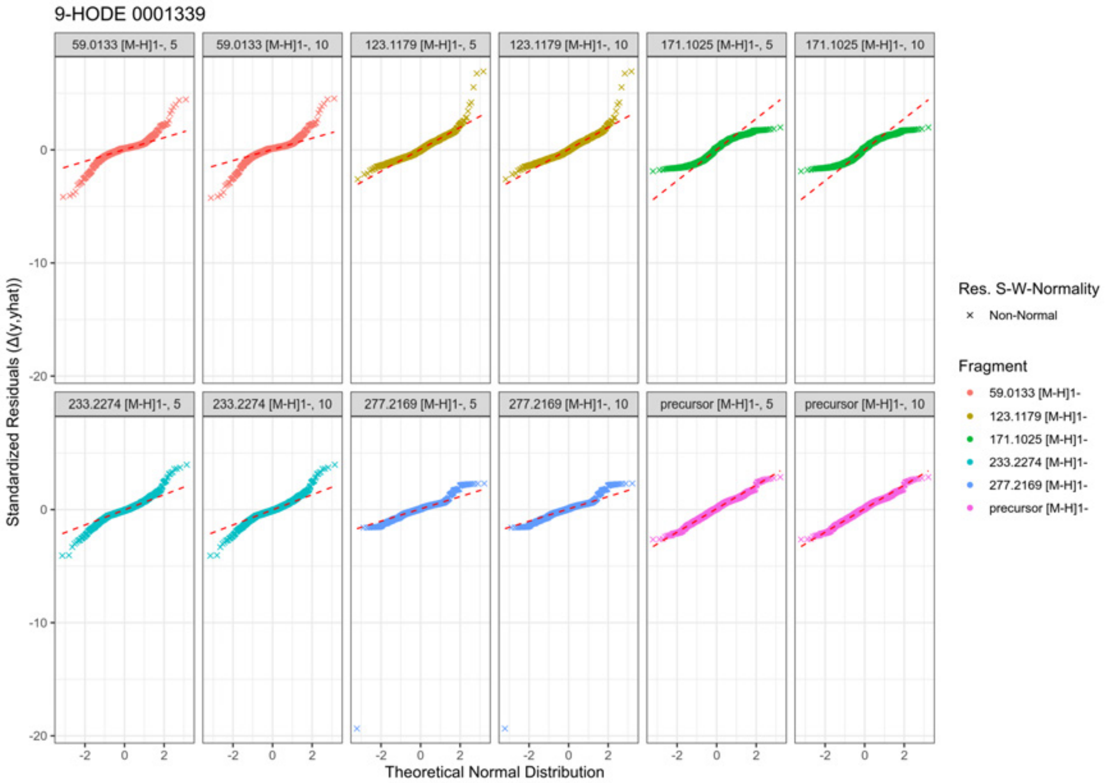

Figure 173. Quantile-quantile plot of residuals

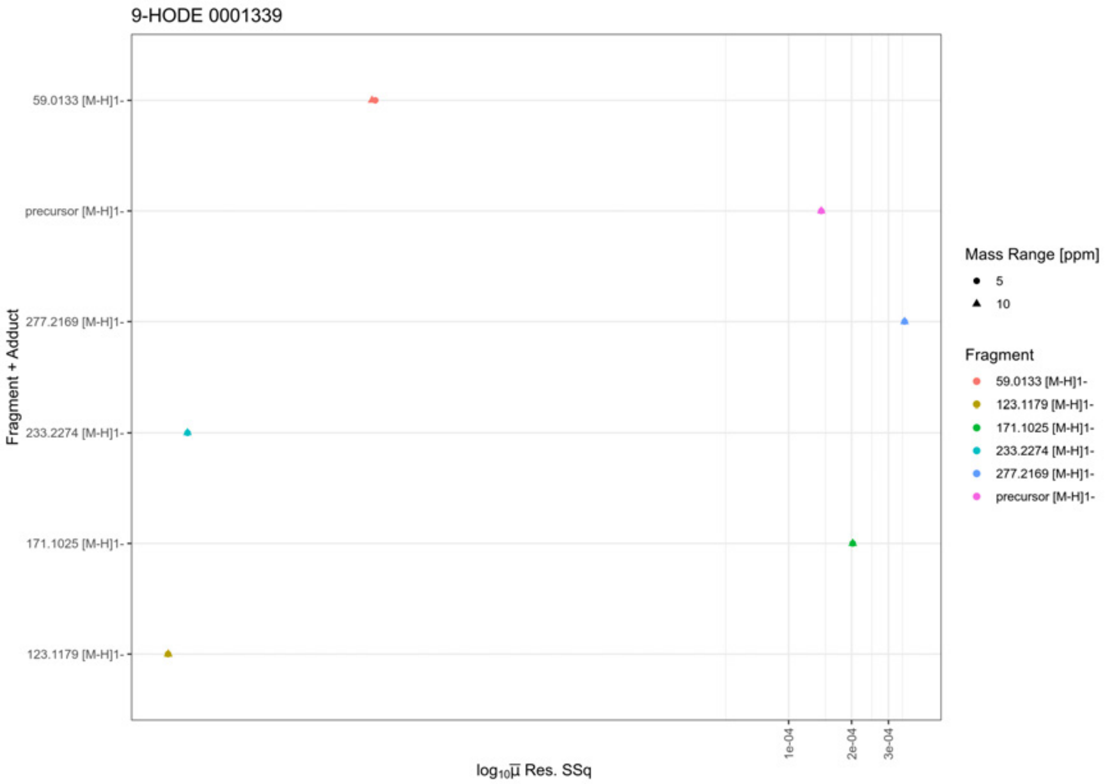

Figure 174. Normalized sum-of-squares of the residuals

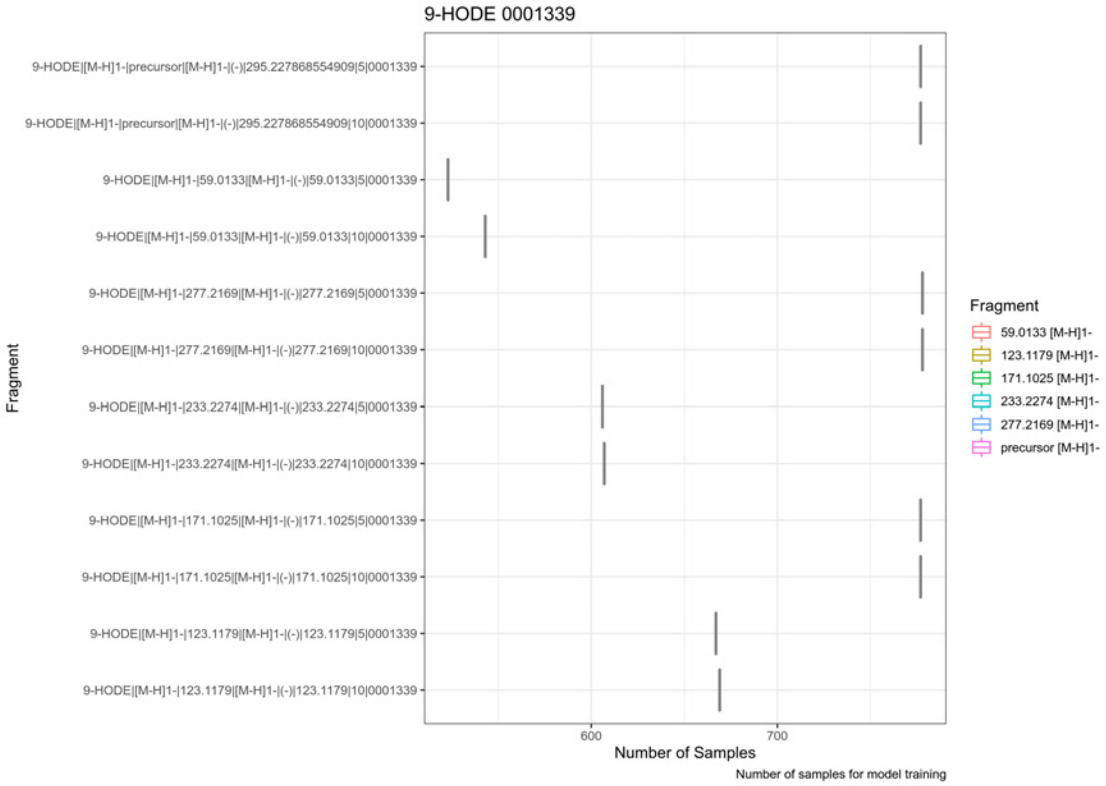

Figure 175. Number of samples used for training per combination Id

1.36. 9-HOTrE [M-H]1- 0000137

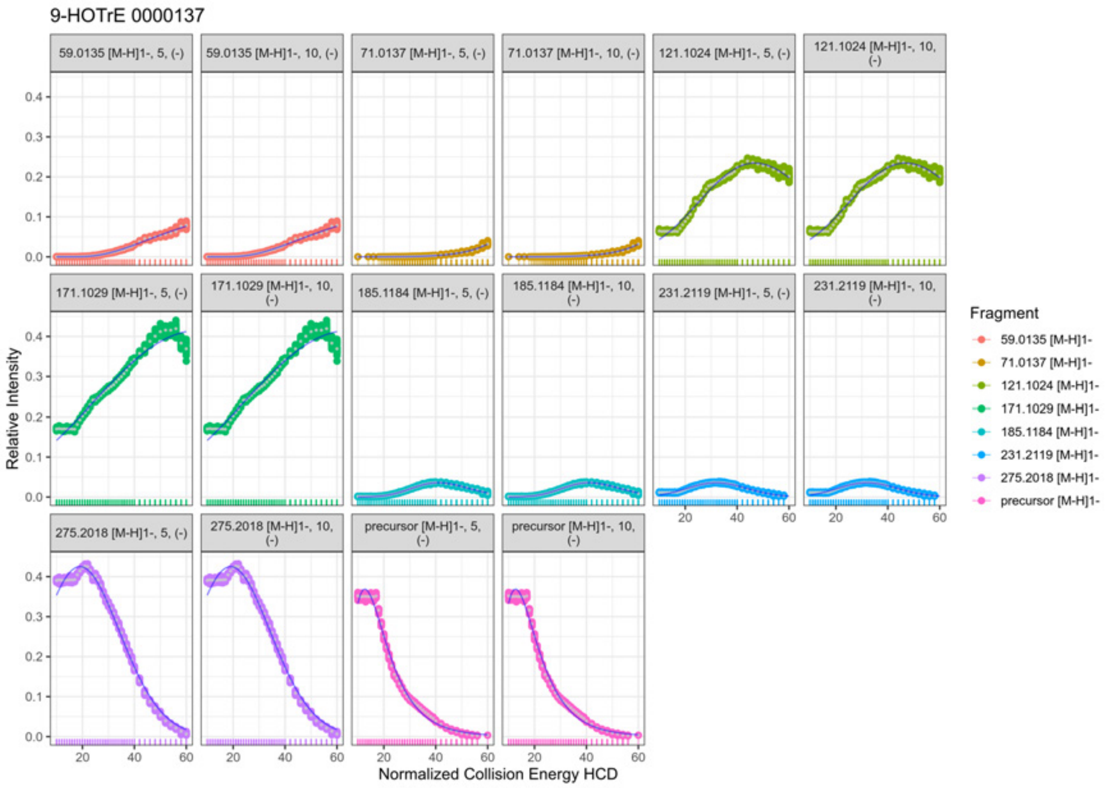

Figure 176. Nonlinear fit

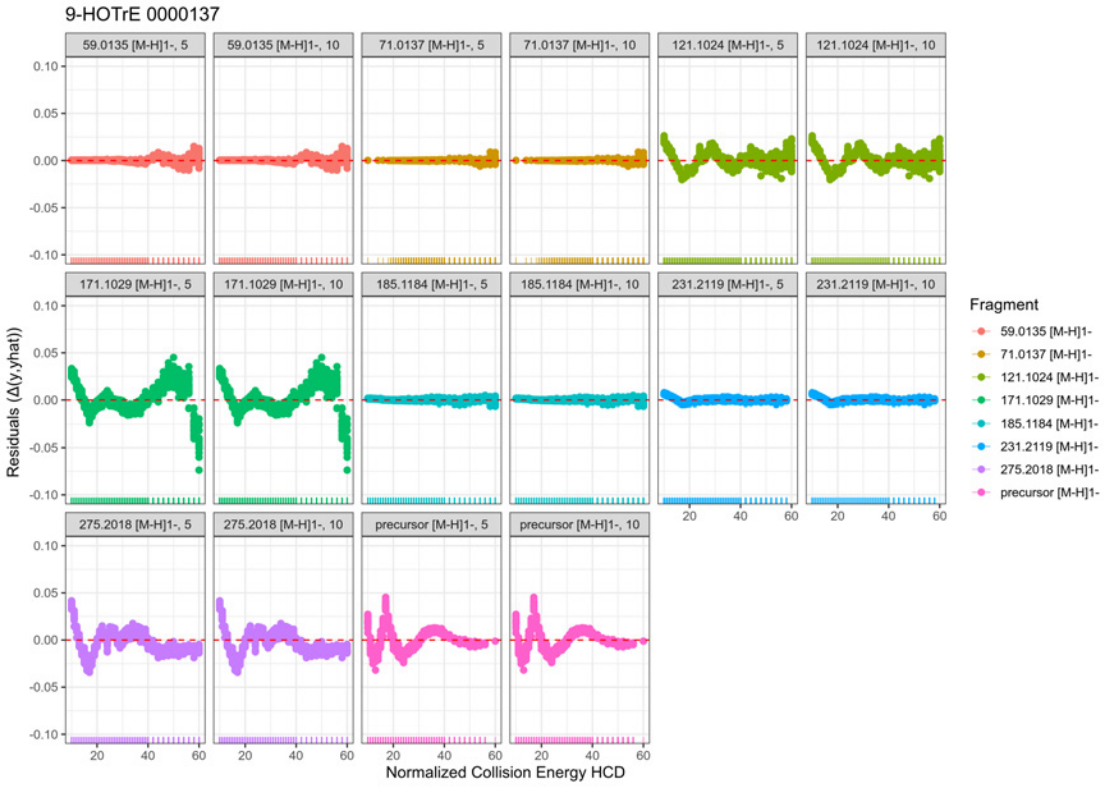

Figure 177. Residuals of nonlinear fit

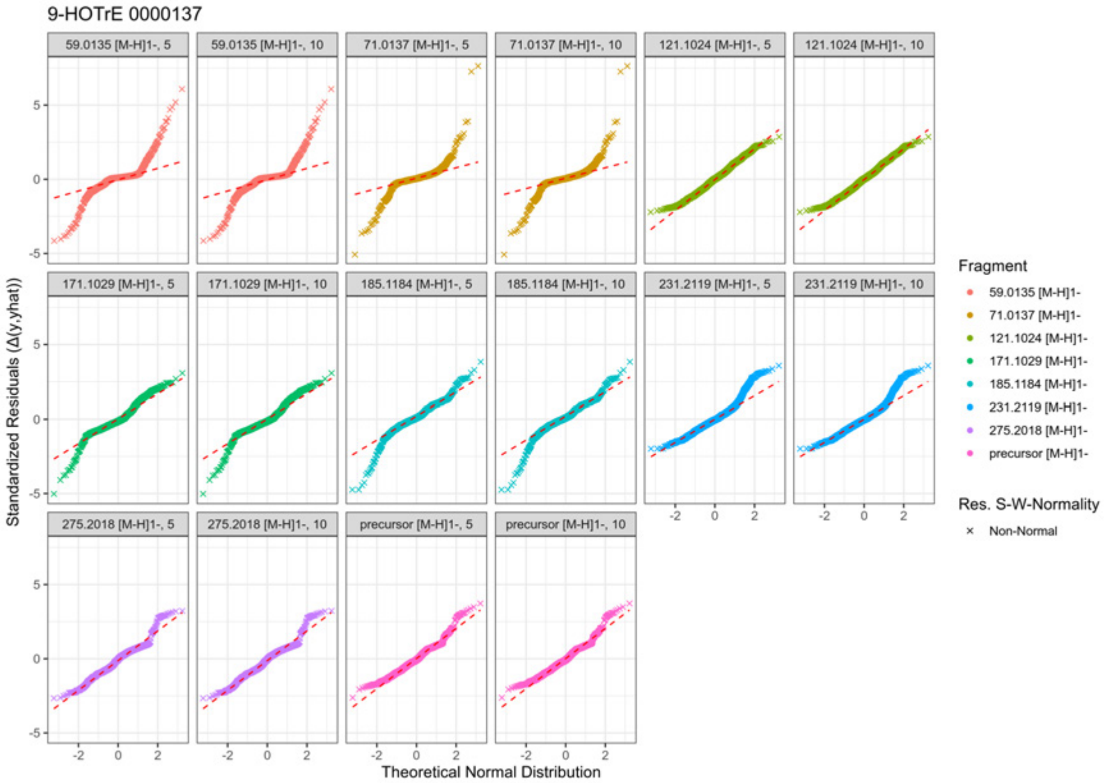

Figure 178. Quantile-quantile plot of residuals

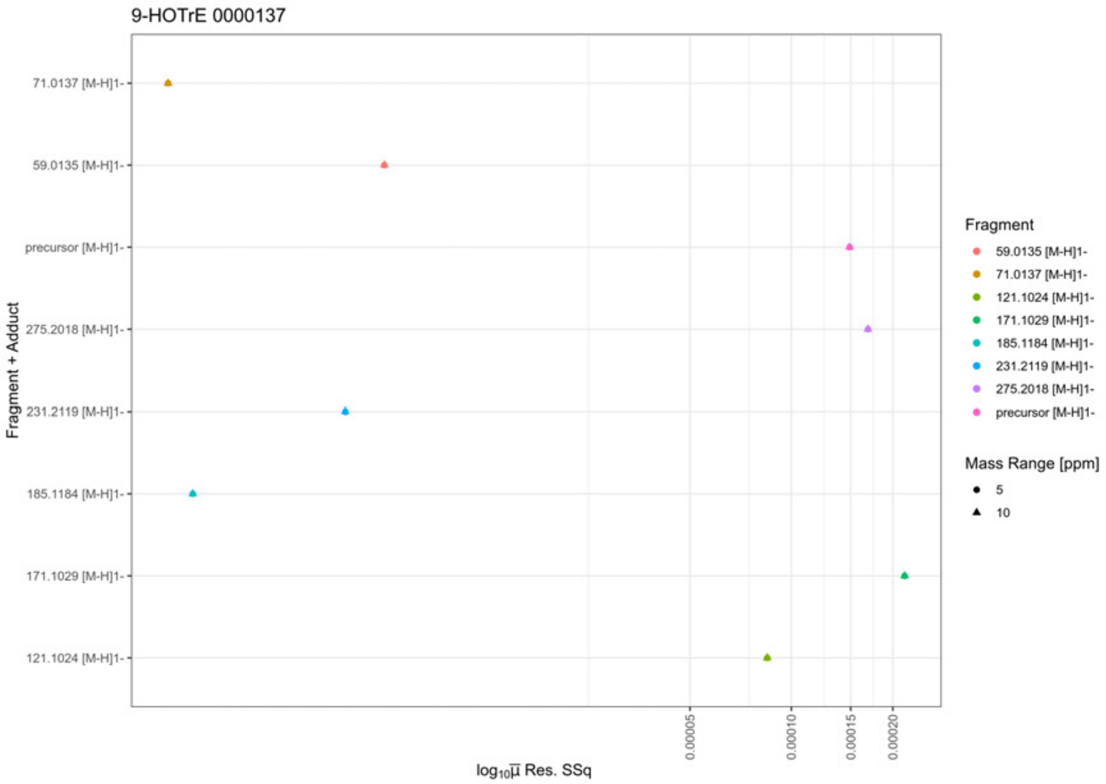

Figure 179. Normalized sum-of-squares of the residuals

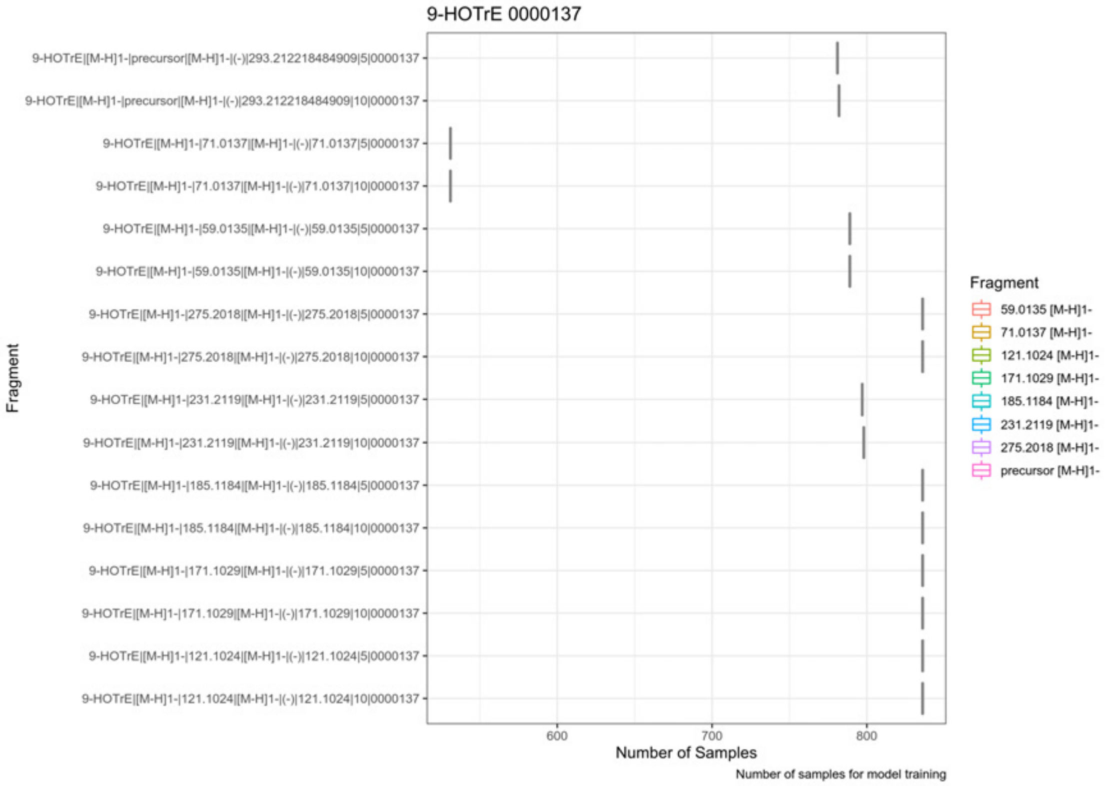

Figure 180. Number of samples used for training per combination Id

1.37. AA{d8} [M-H]1- 0001337

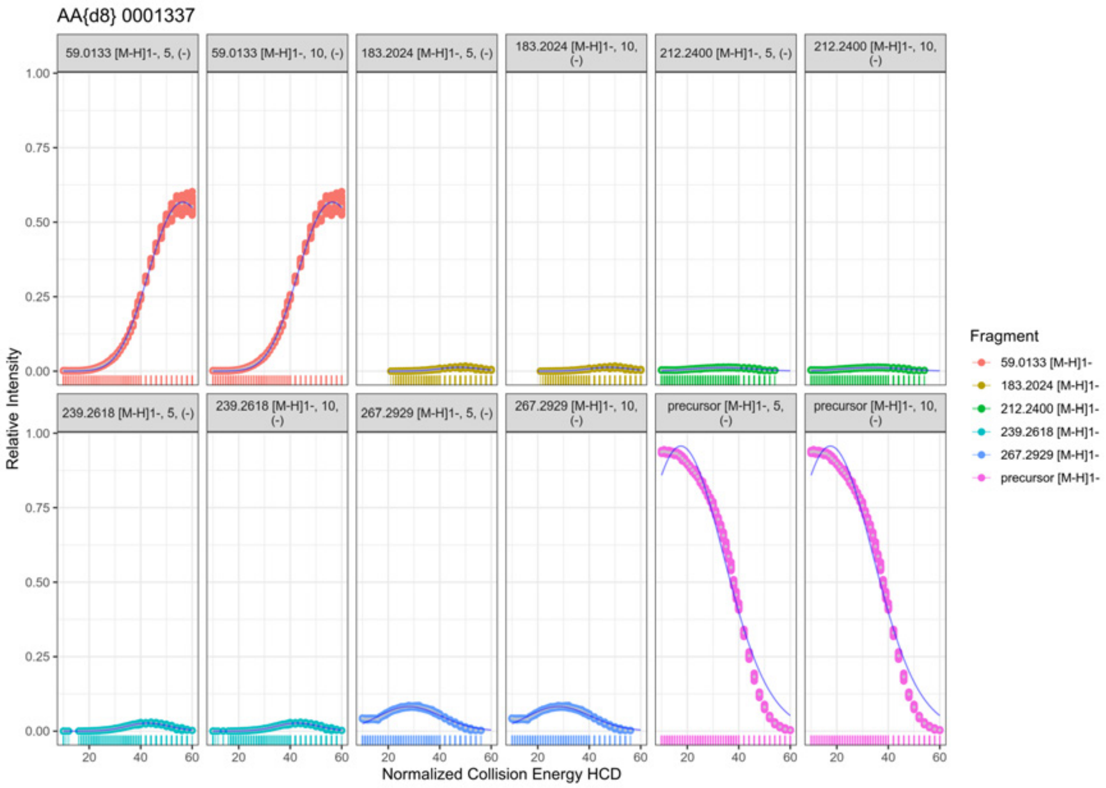

Figure 181. Nonlinear fit

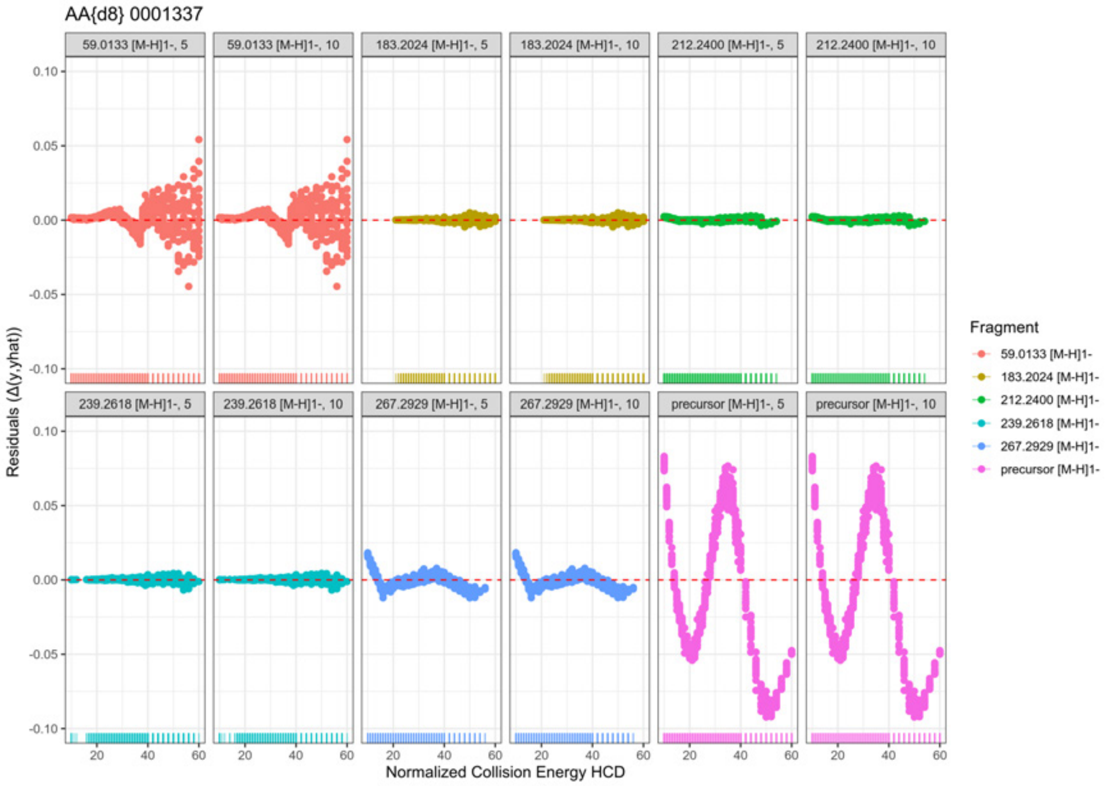

Figure 182. Residuals of nonlinear fit

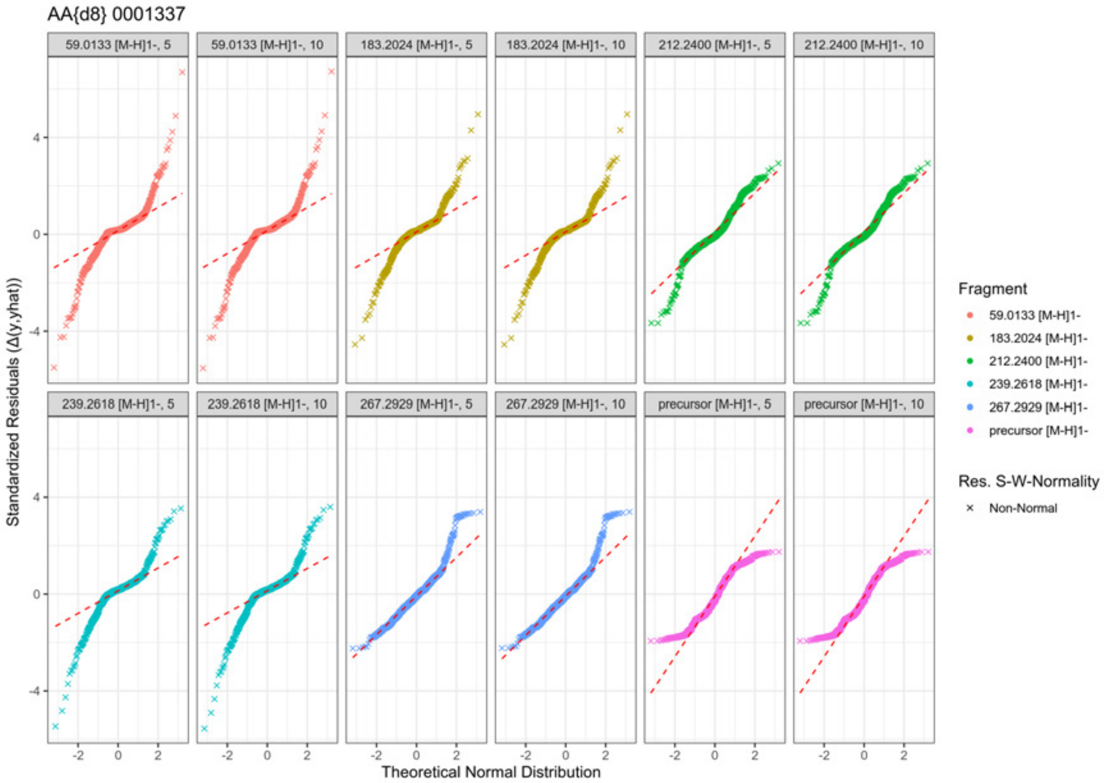

Figure 183. Quantile-quantile plot of residuals

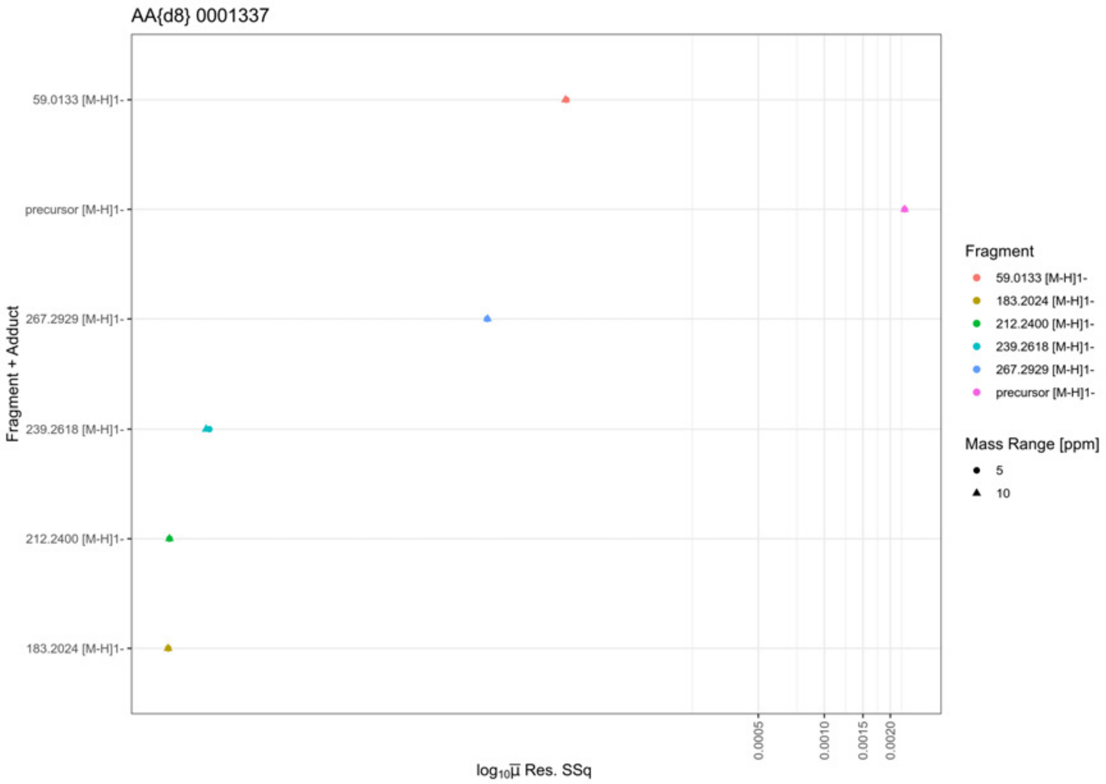

Figure 184. Normalized sum-of-squares of the residuals

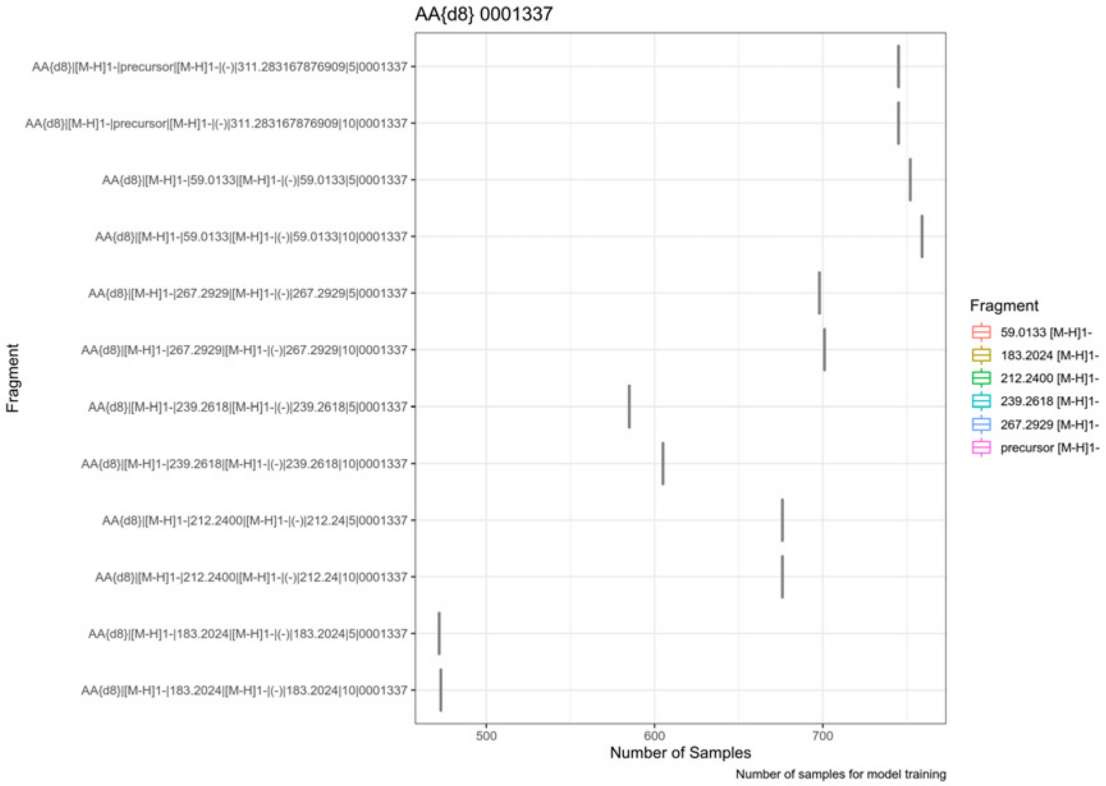

Figure 185. Number of samples used for training per combination Id

1.38. DHA{d5} [M-H]1- 0001353

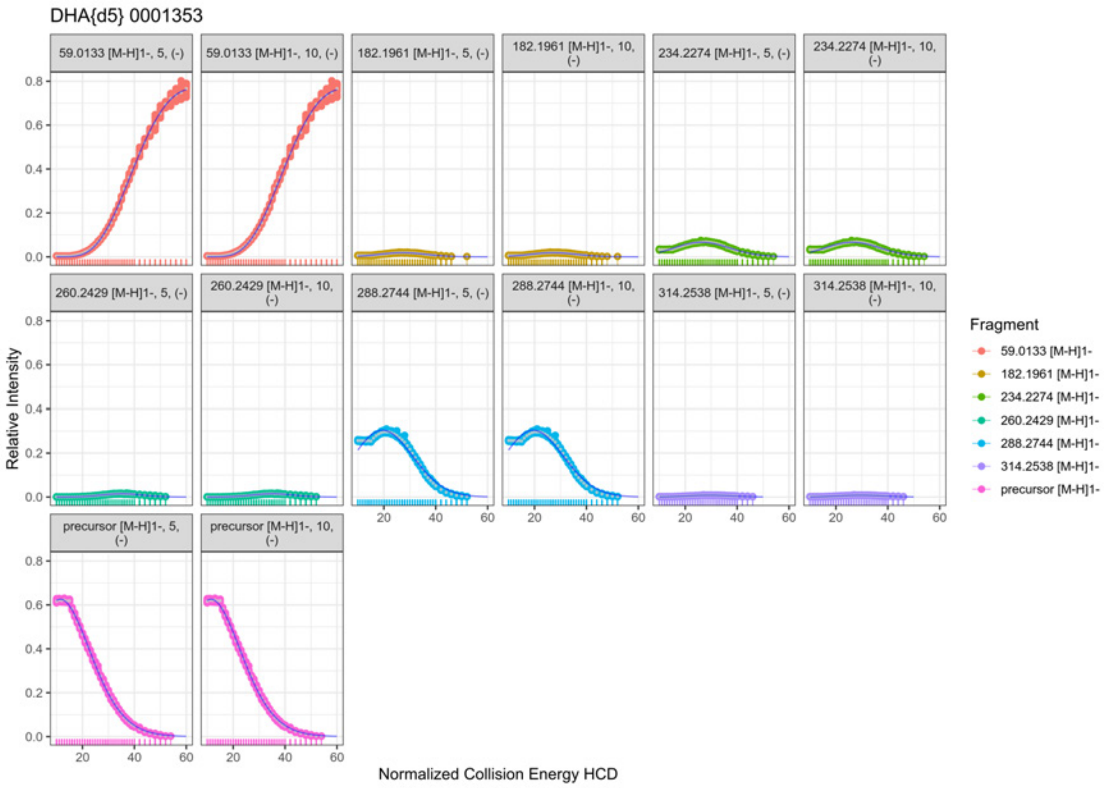

Figure 186. Nonlinear fit

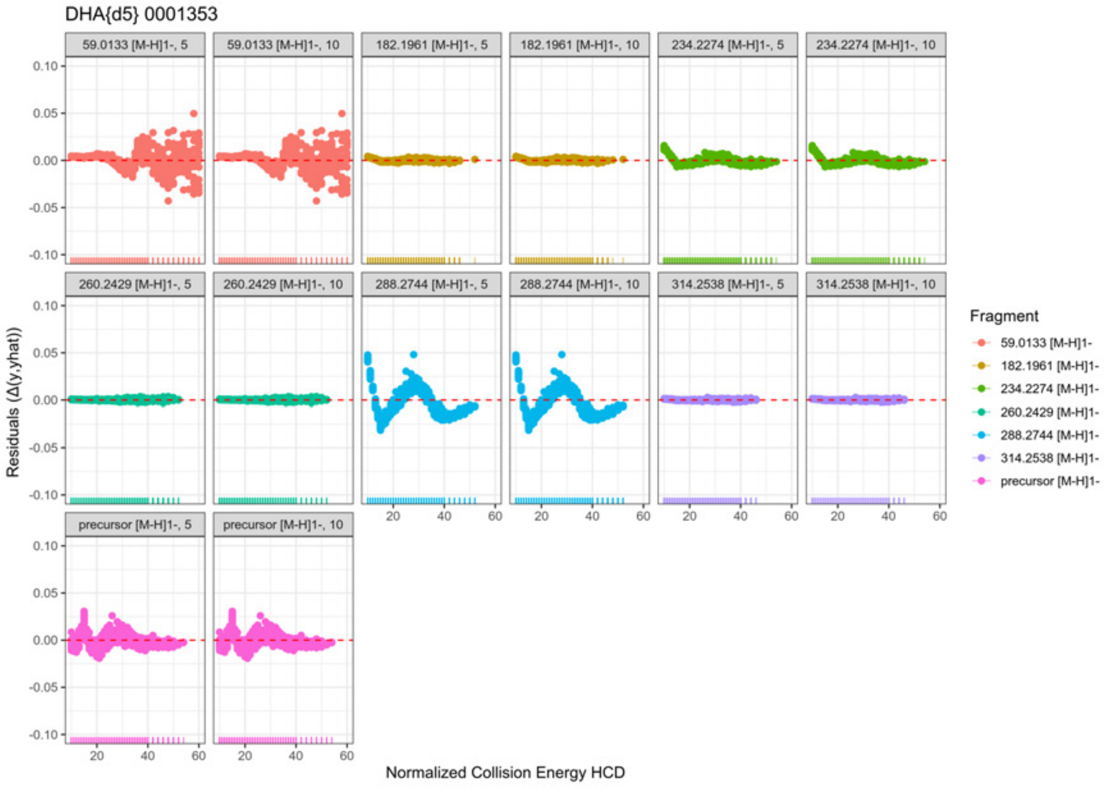

Figure 187. Residuals of nonlinear fit

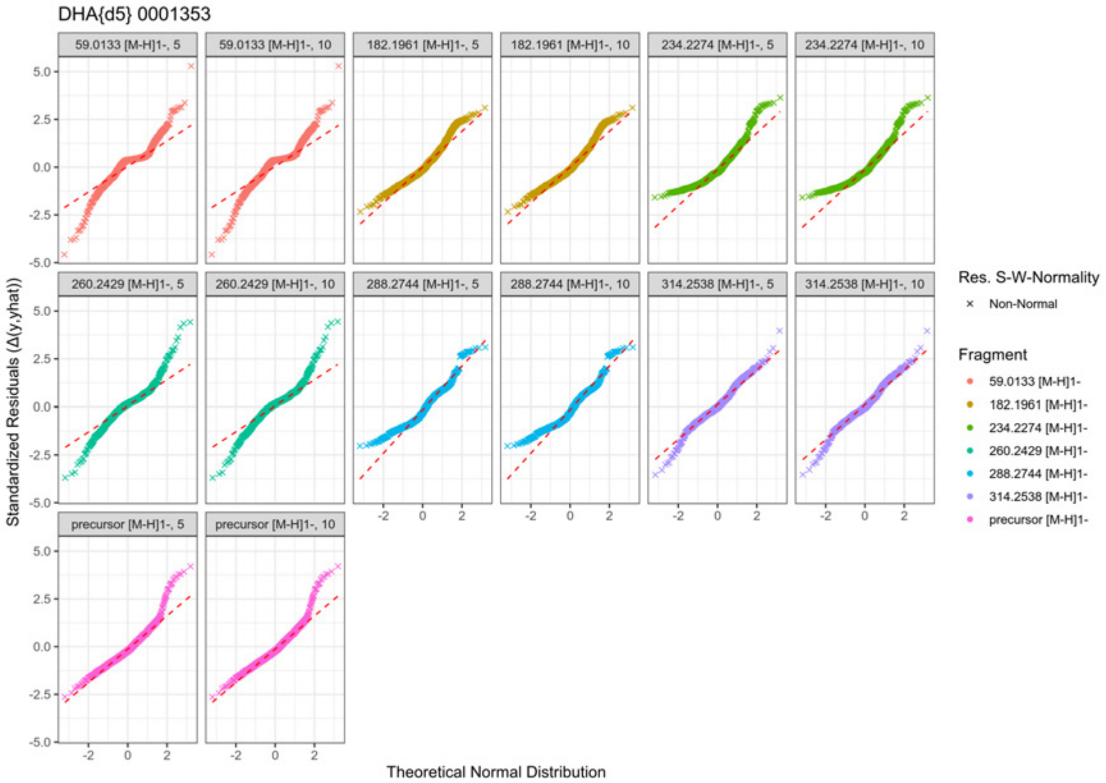

Figure 188. Quantile-quantile plot of residuals

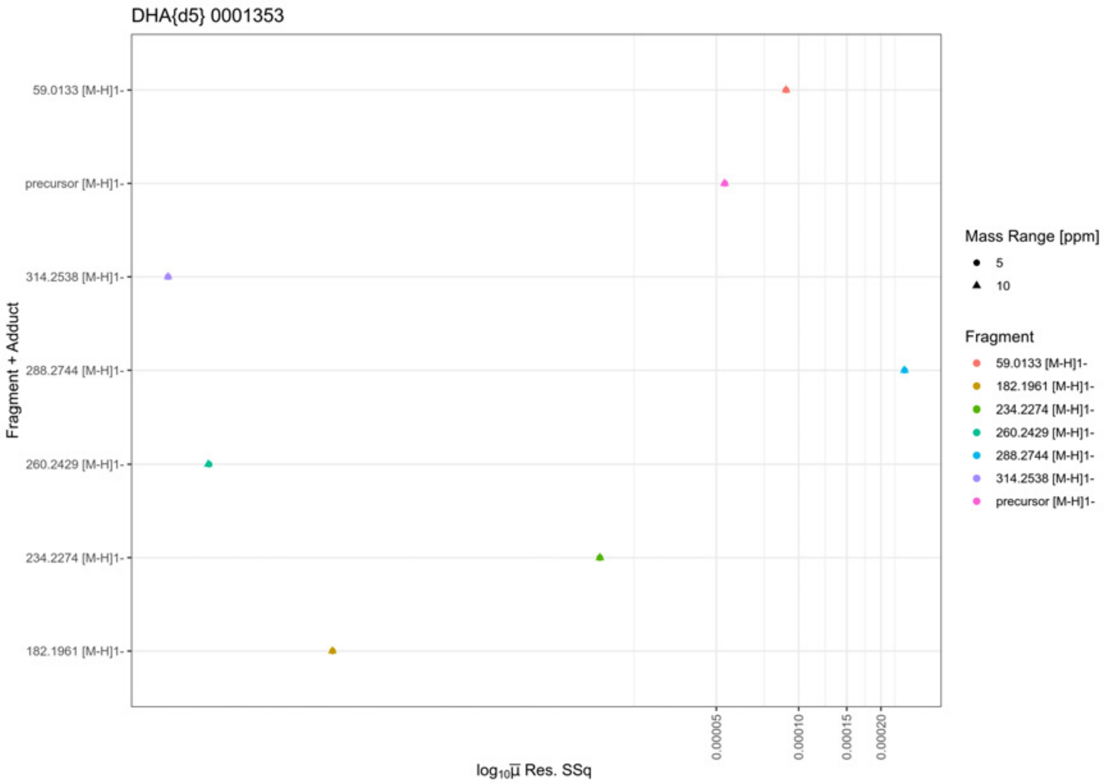

Figure 189. Normalized sum-of-squares of the residuals

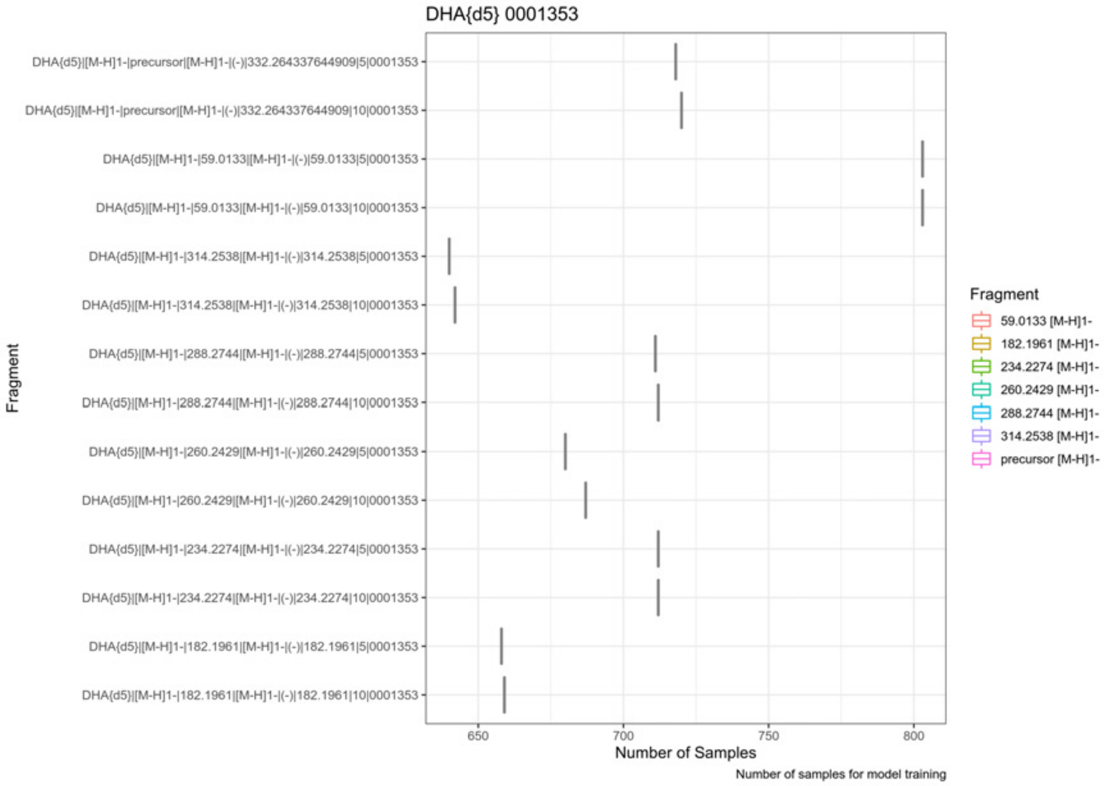

Figure 190. Number of samples used for training per combination Id

1.39. EPA{d5} [M-H]1- 0001335

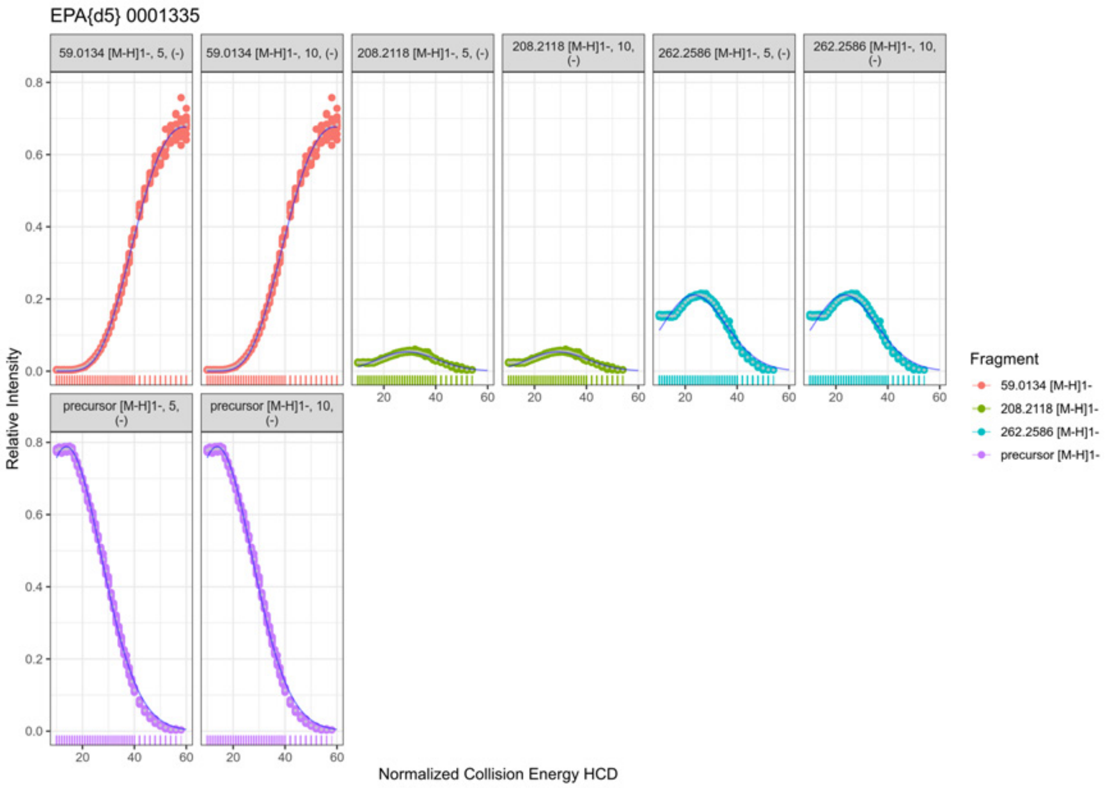

Figure 191. Nonlinear fit

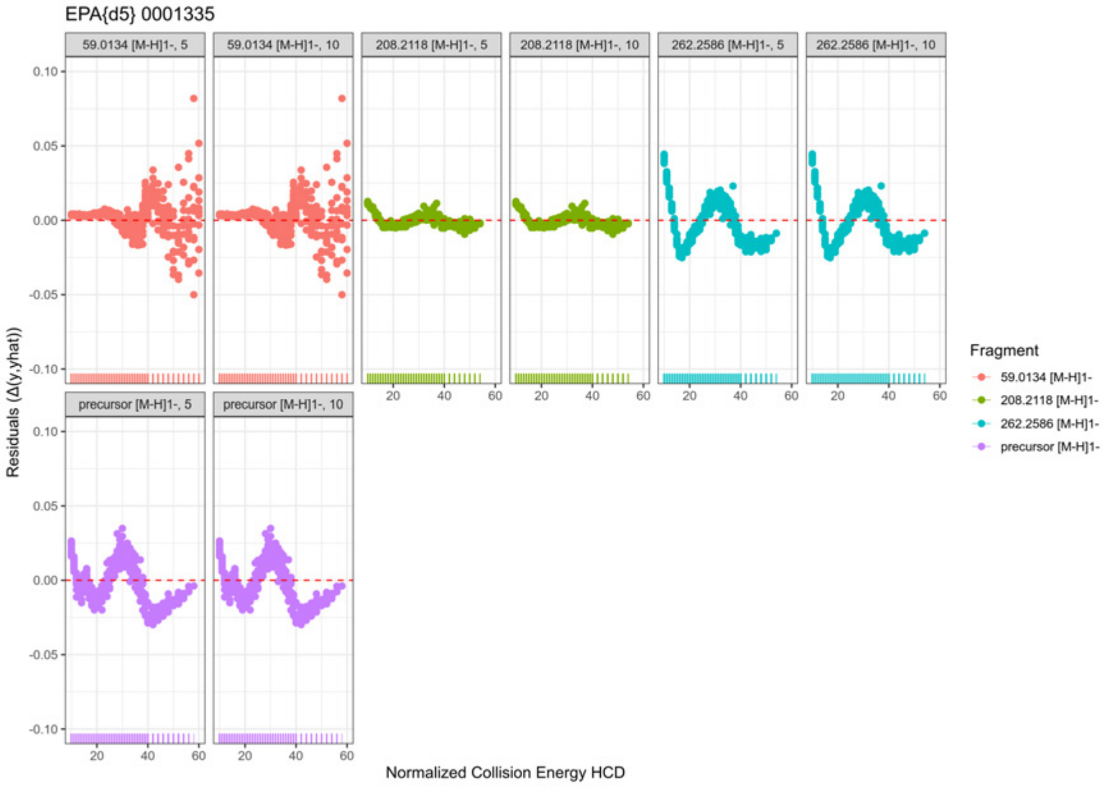

Figure 192. Residuals of nonlinear fit

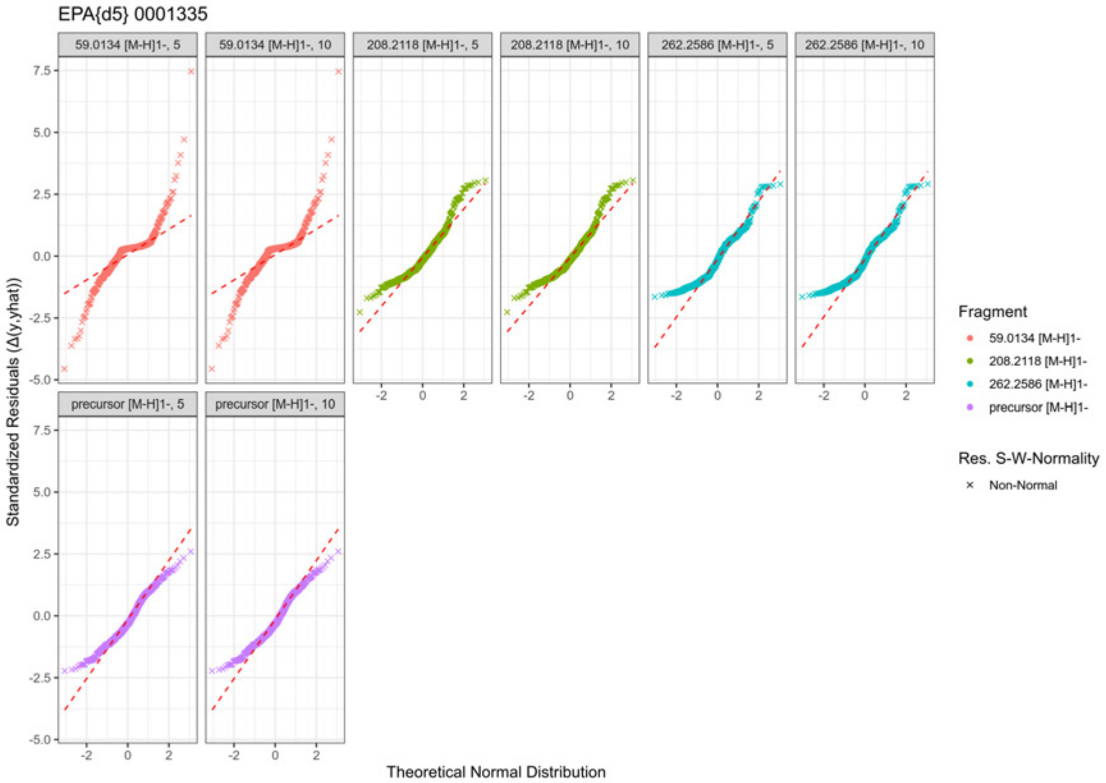

Figure 193. Quantile-quantile plot of residuals

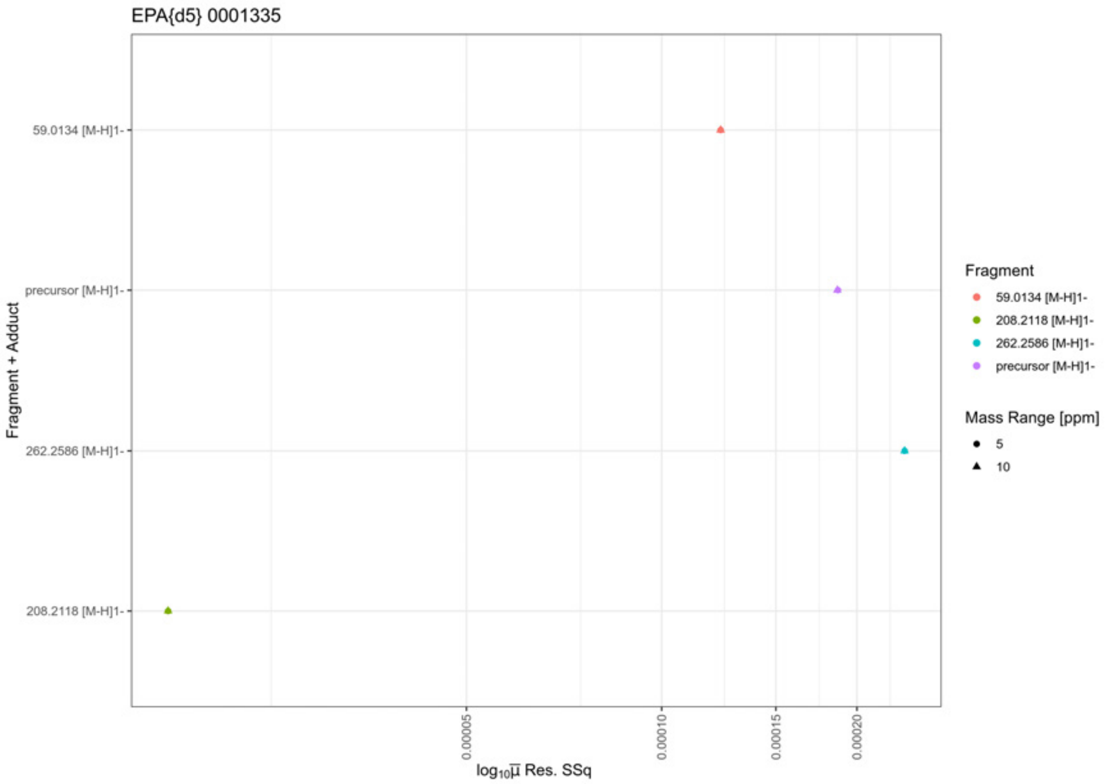

Figure 194. Normalized sum-of-squares of the residuals

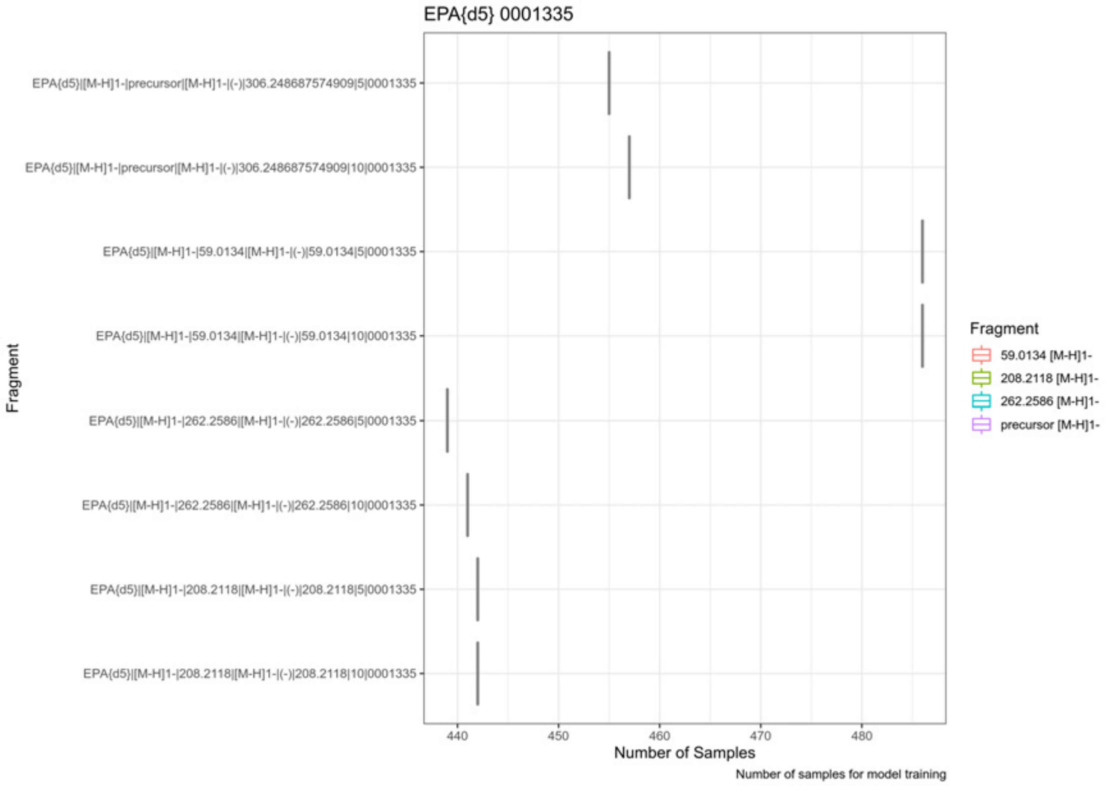

Figure 195. Number of samples used for training per combination Id

1.40. LTB4{d4} [M-H]1- 0001331

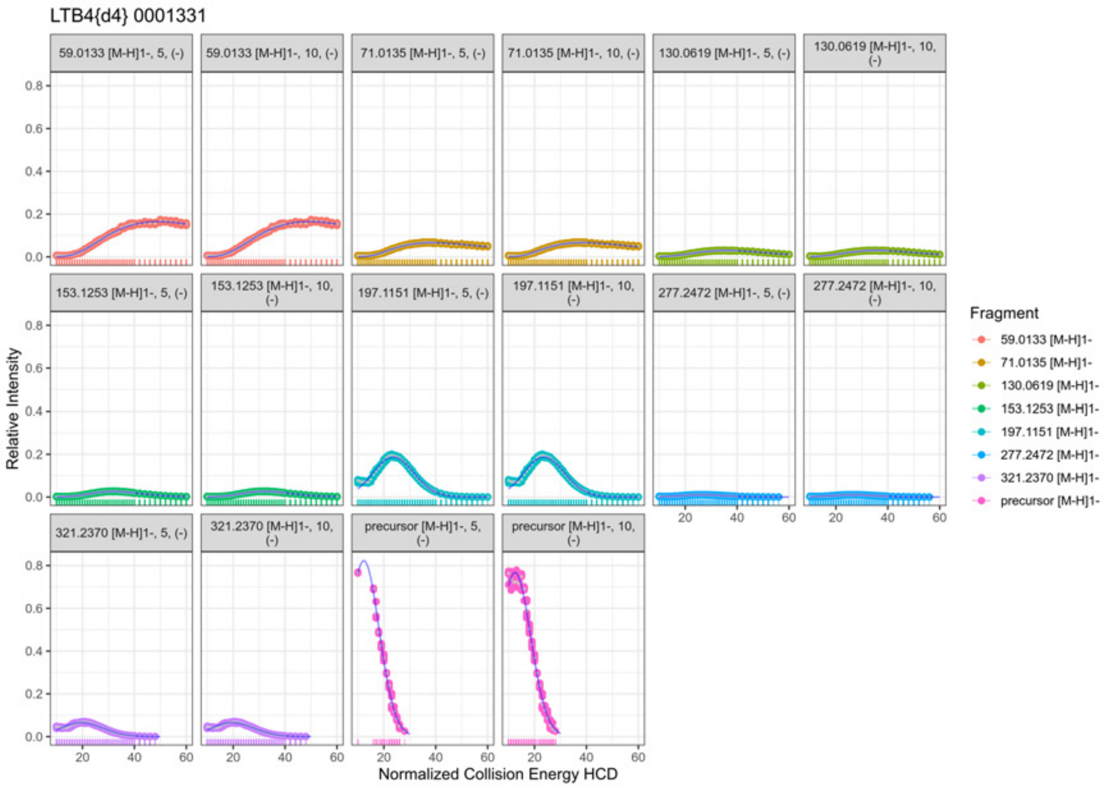

Figure 196. Nonlinear fit

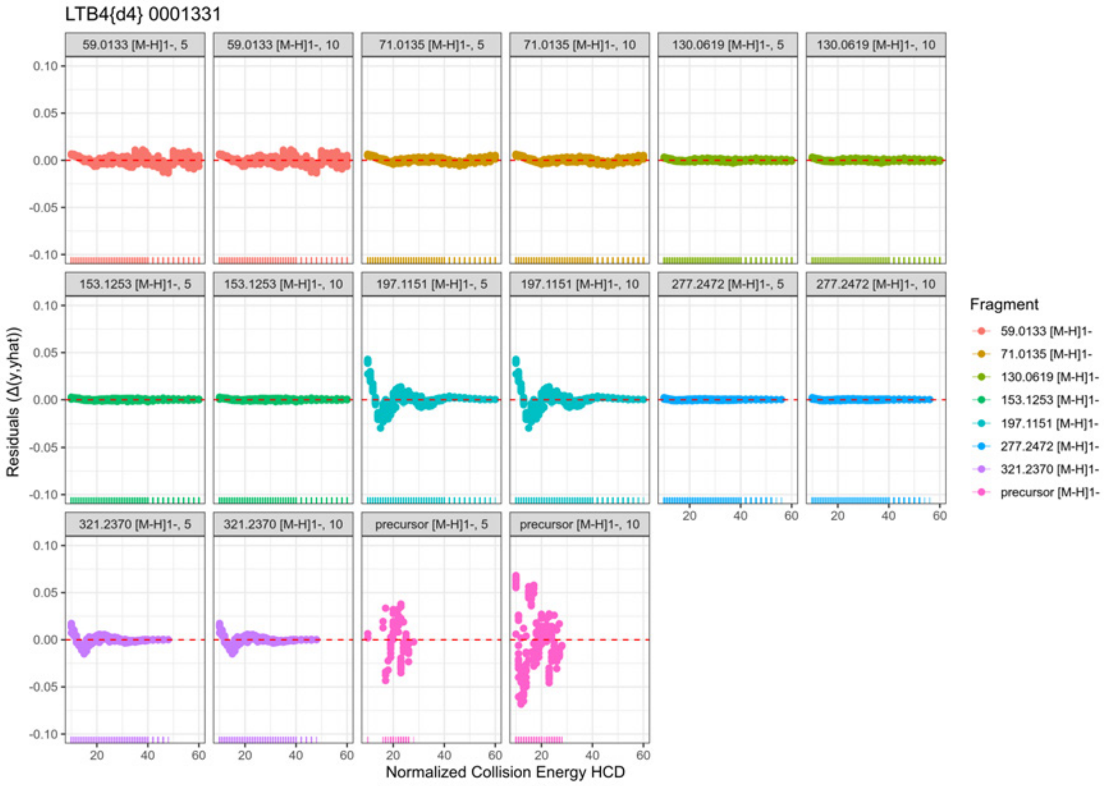

Figure 197. Residuals of nonlinear fit

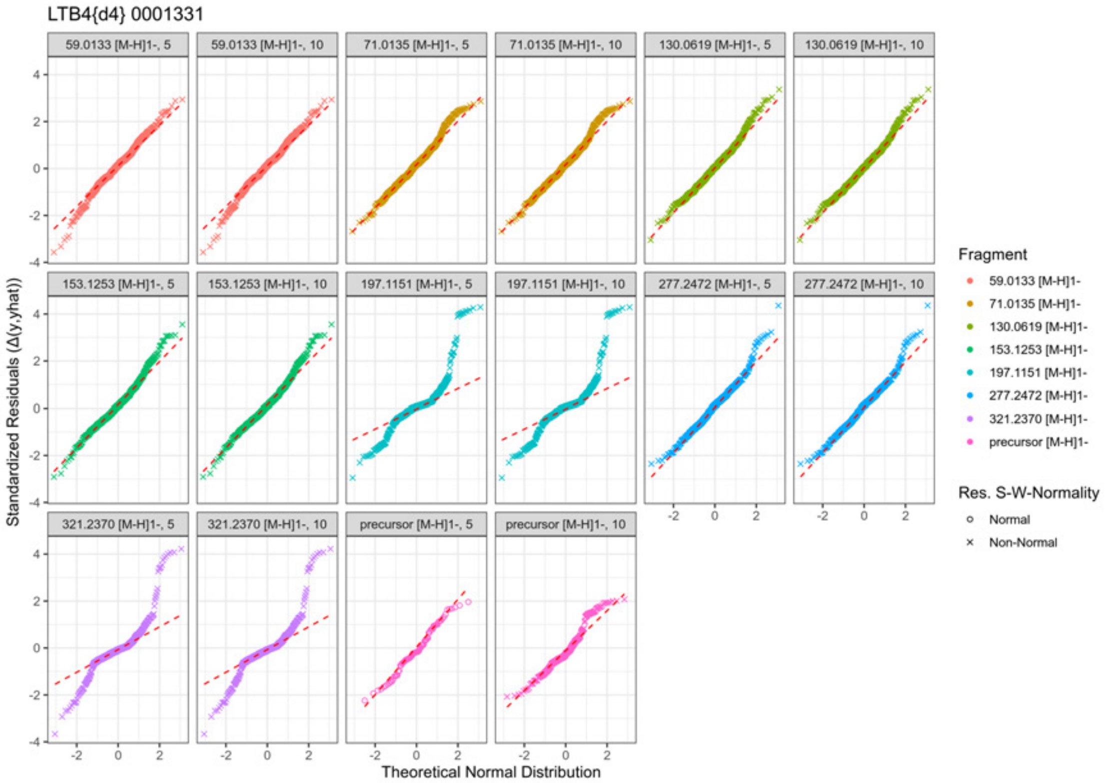

Figure 198. Quantile-quantile plot of residuals

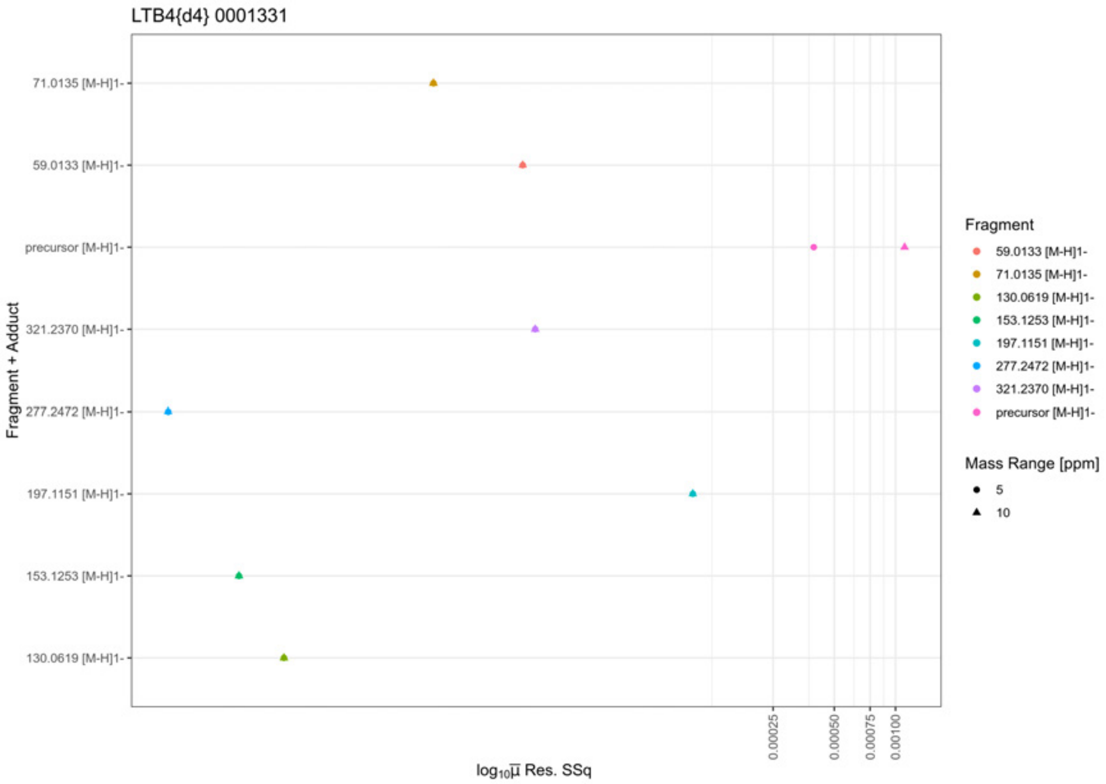

Figure 199. Normalized sum-of-squares of the residuals

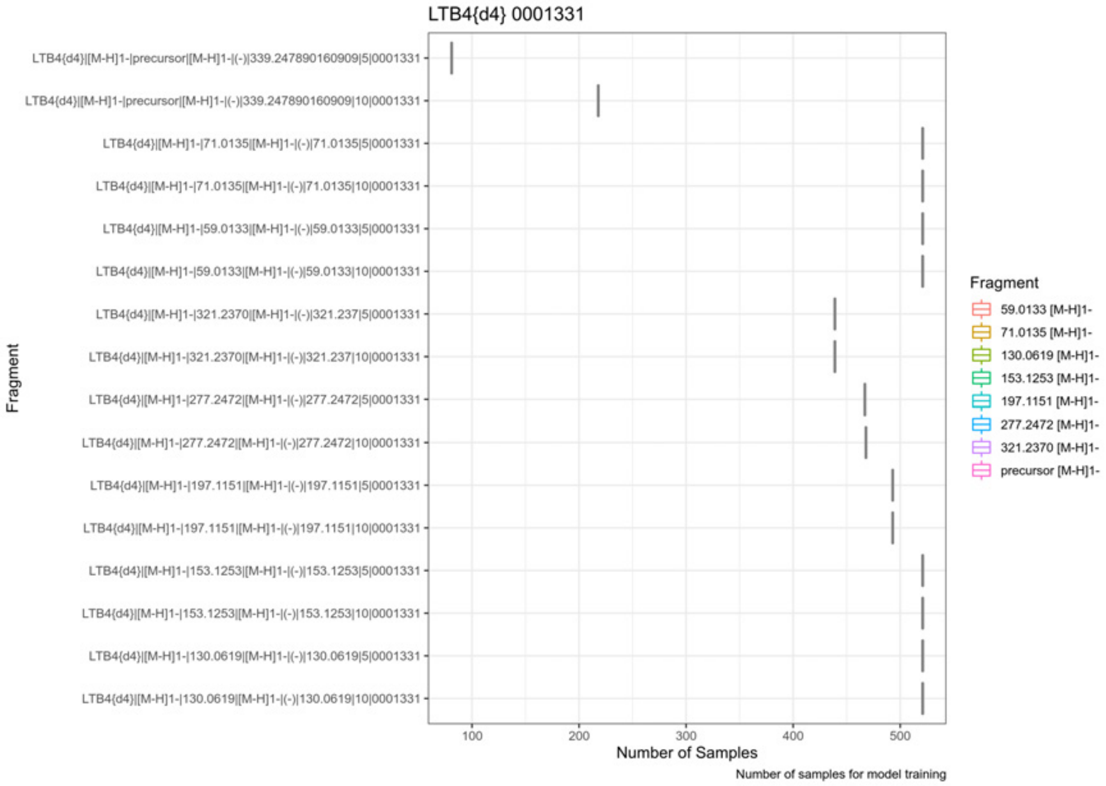

Figure 200. Number of samples used for training per combination Id

1.41. LTC4{d5} [M-H]1- 0000163

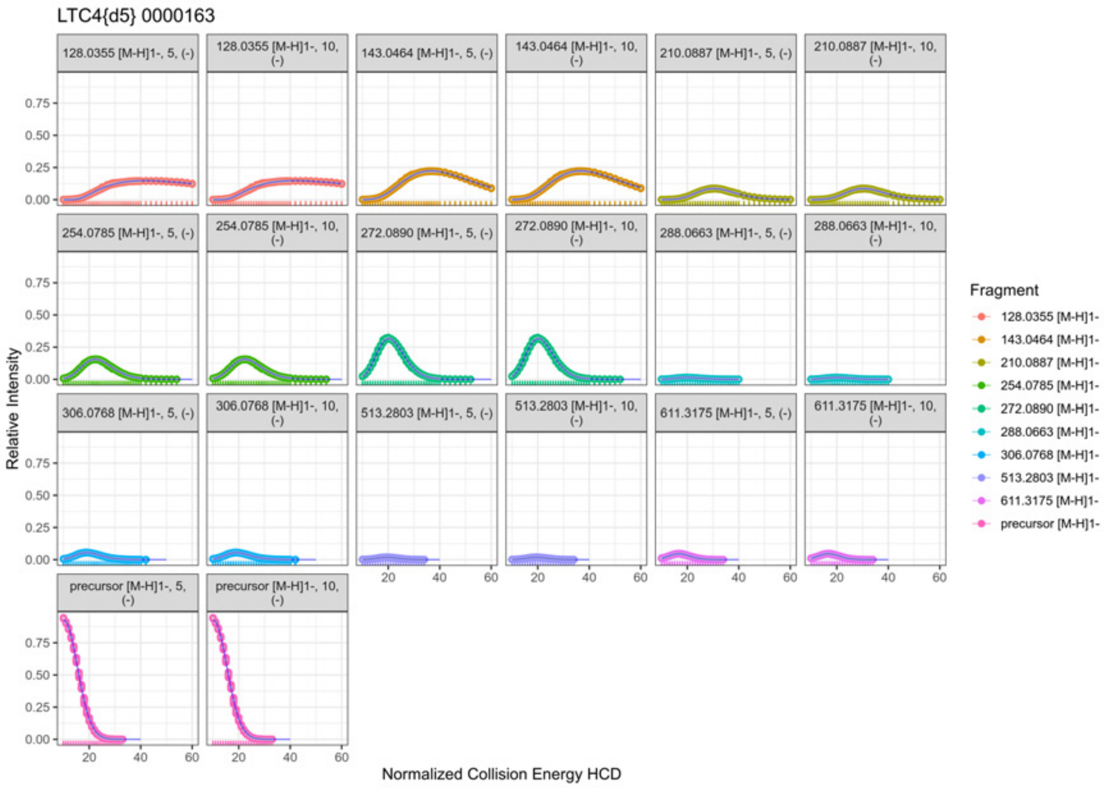

Figure 201. Nonlinear fit

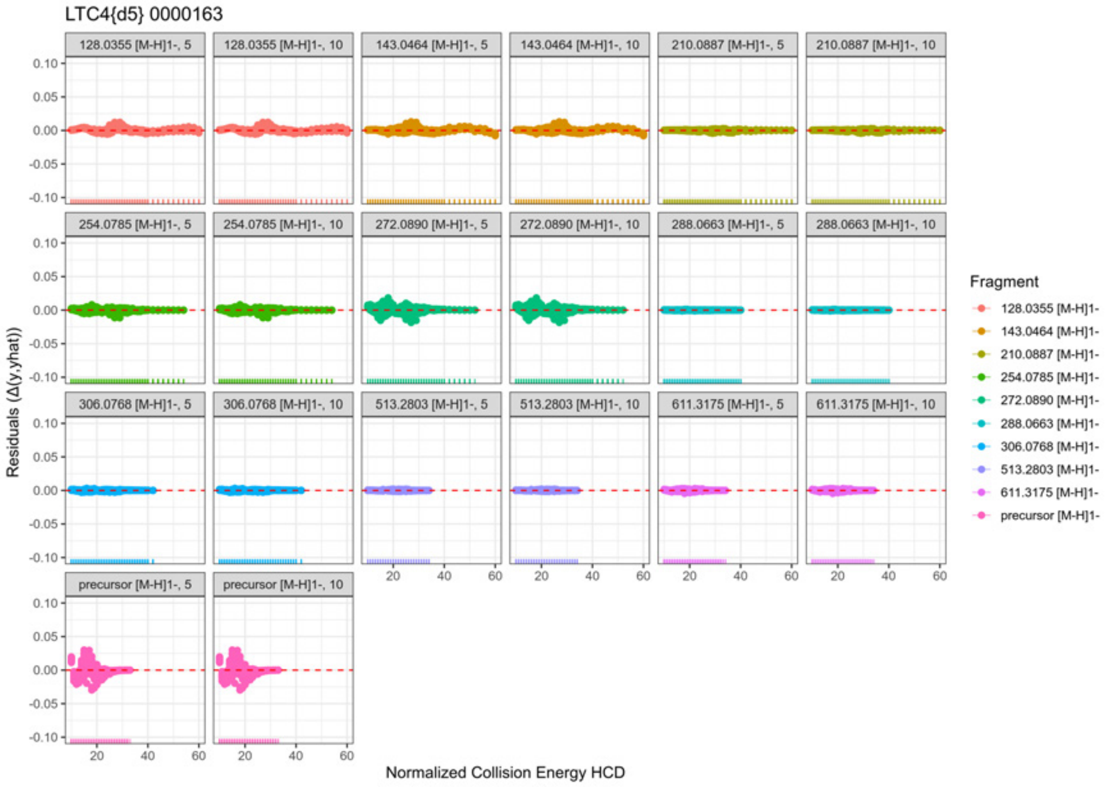

Figure 202. Residuals of nonlinear fit

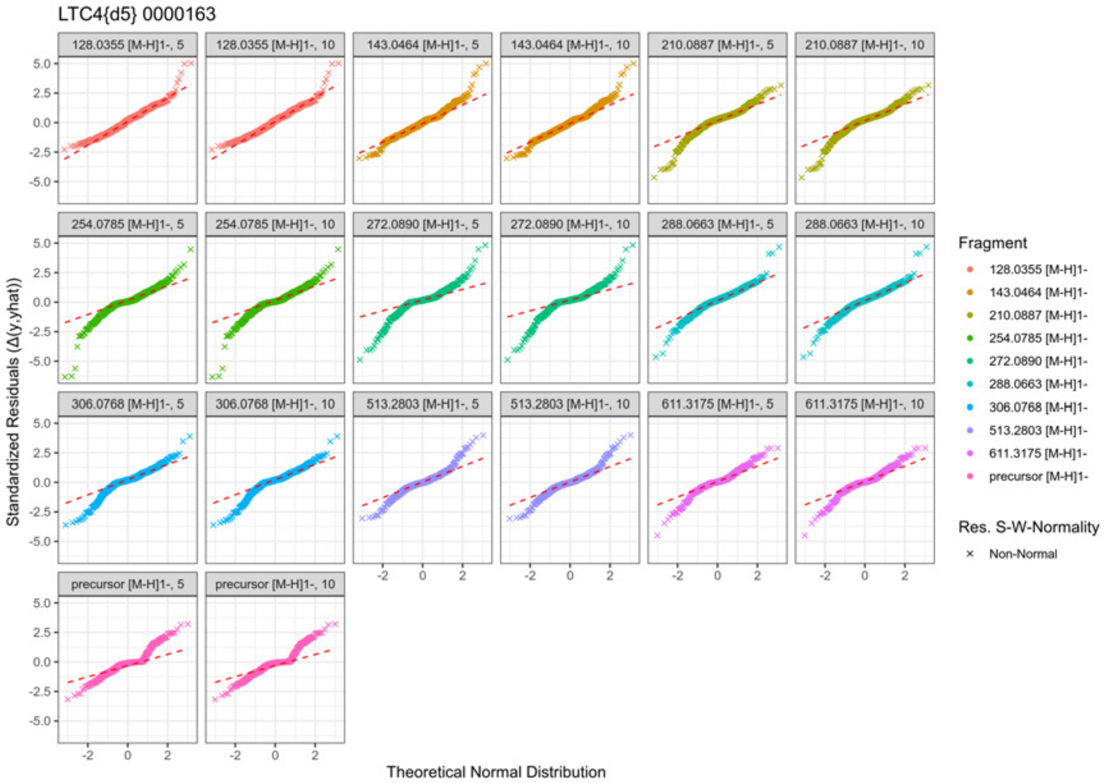

Figure 203. Quantile-quantile plot of residuals

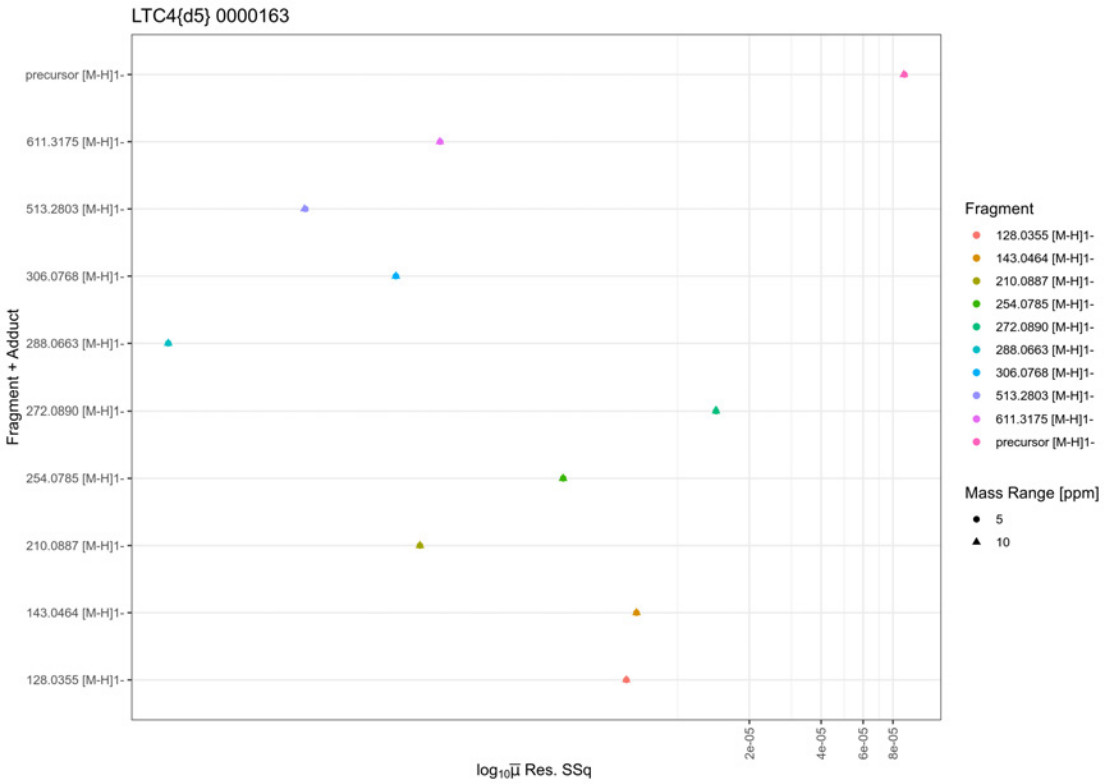

Figure 204. Normalized sum-of-squares of the residuals

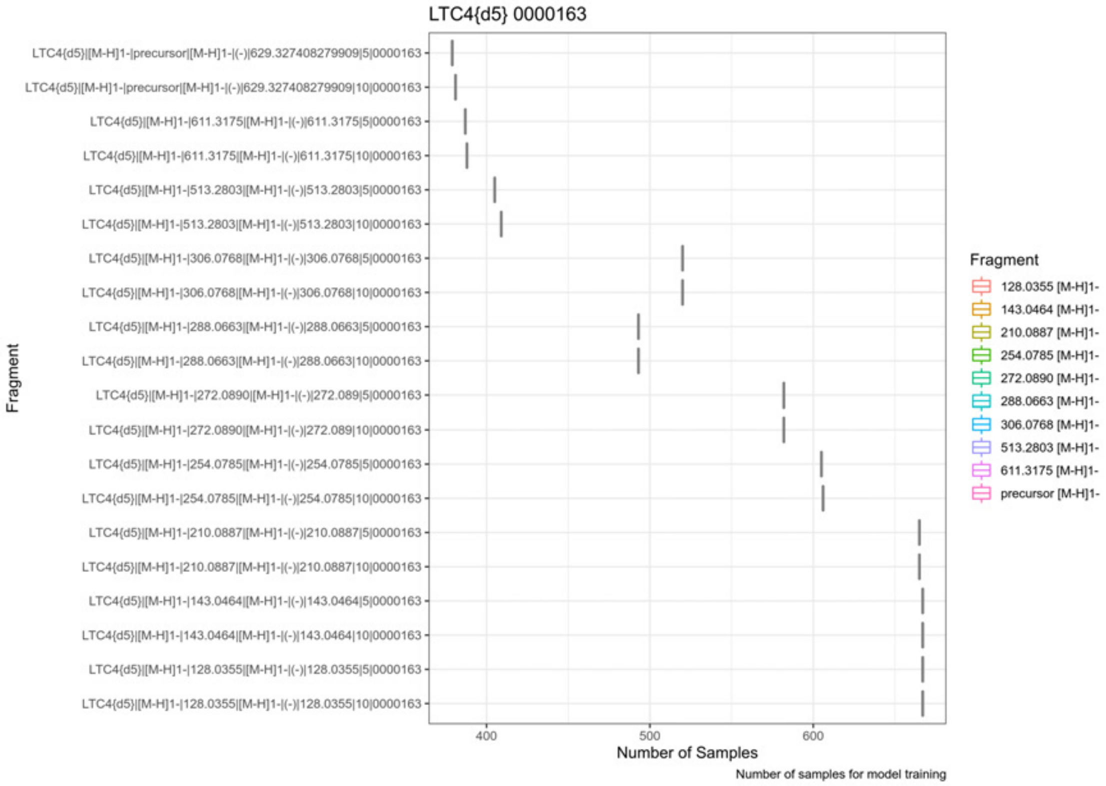

Figure 205. Number of samples used for training per combination Id

1.42. LTD4{d5} [M-H]1- 0000153

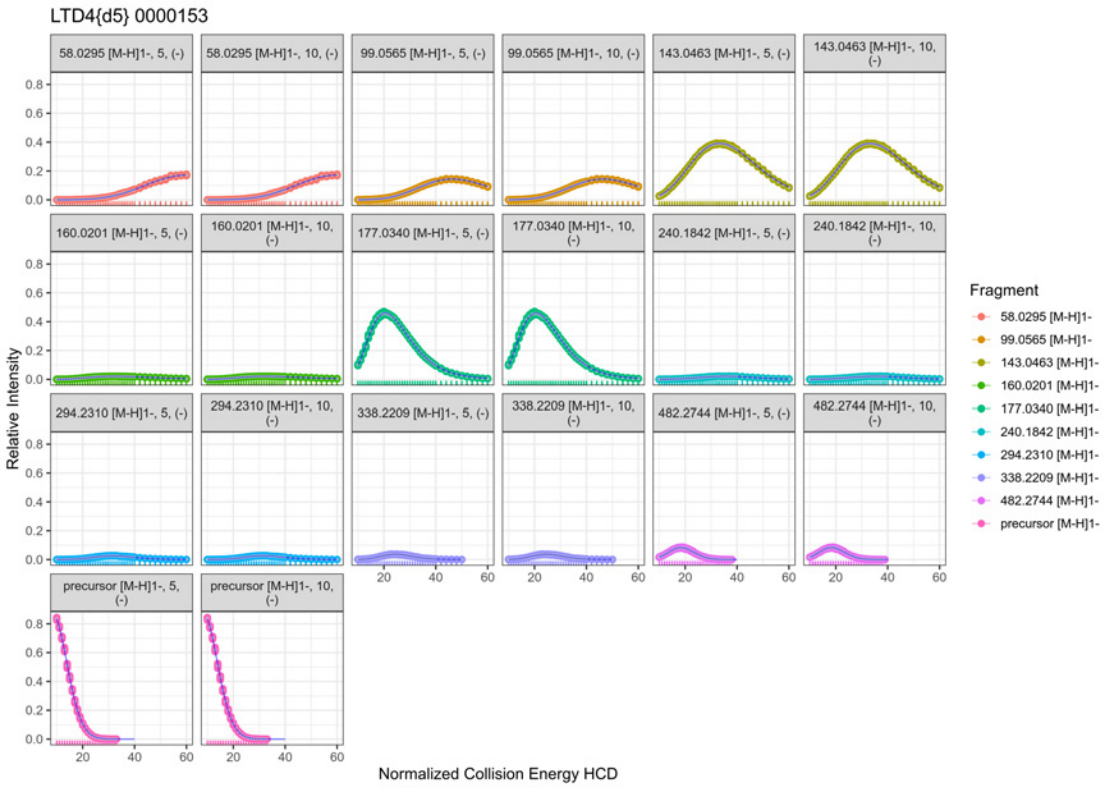

Figure 206. Nonlinear fit

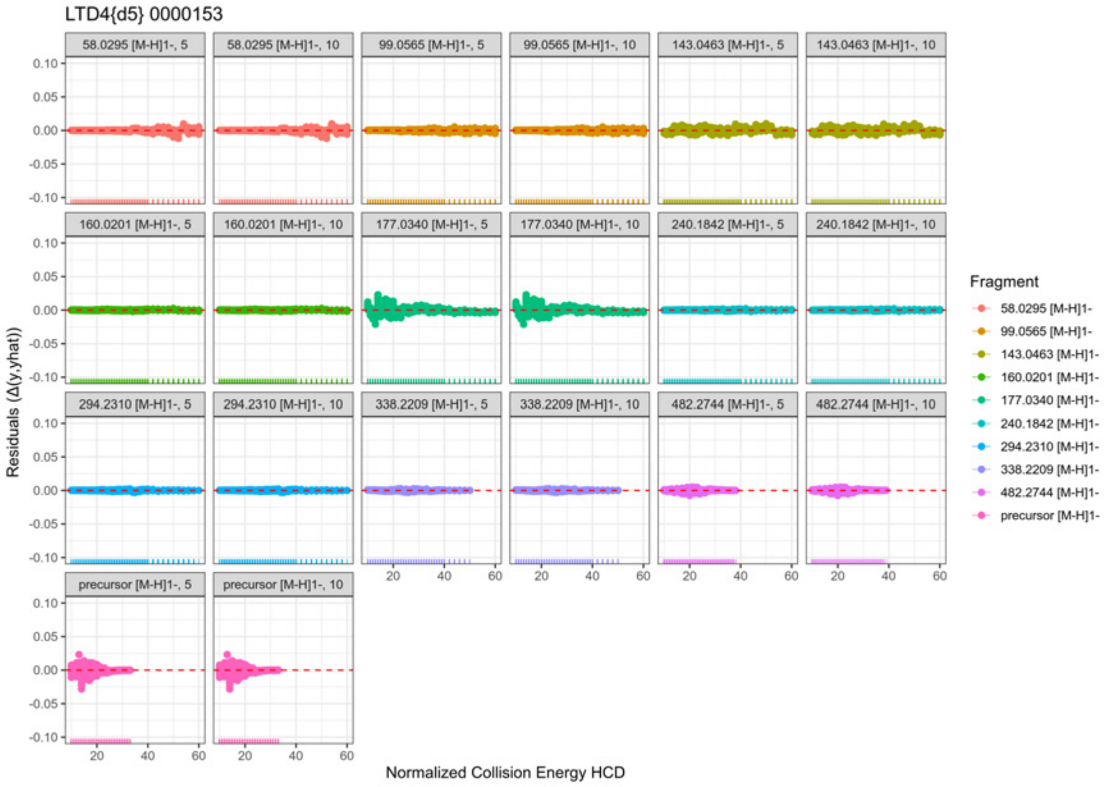

Figure 207. Residuals of nonlinear fit

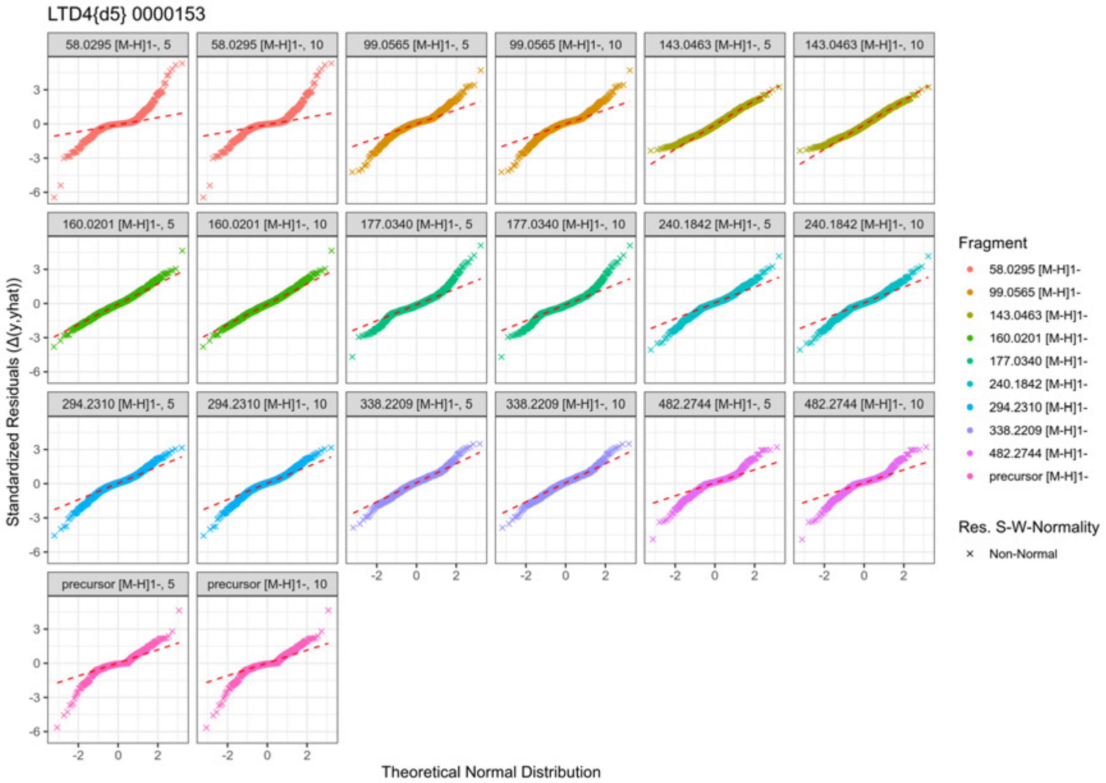

Figure 208. Quantile-quantile plot of residuals

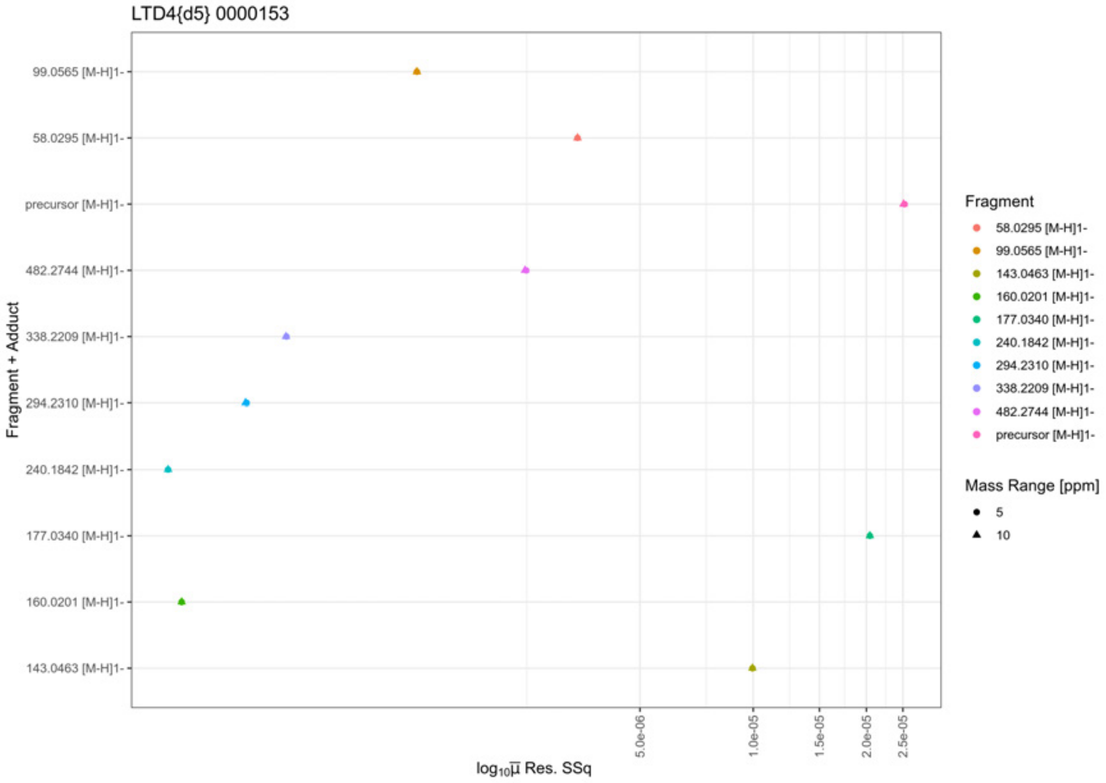

Figure 209. Normalized sum-of-squares of the residuals

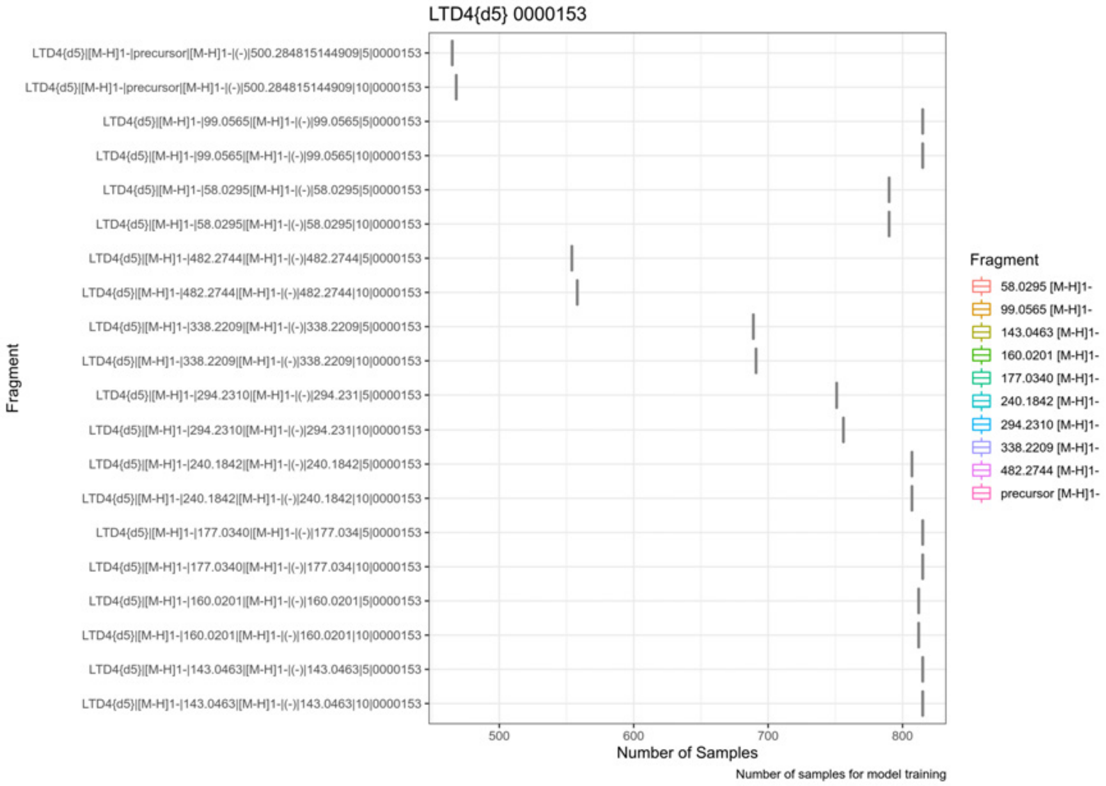

Figure 210. Number of samples used for training per combination Id

1.43. Maresin 1 [M-H]1- 0001319

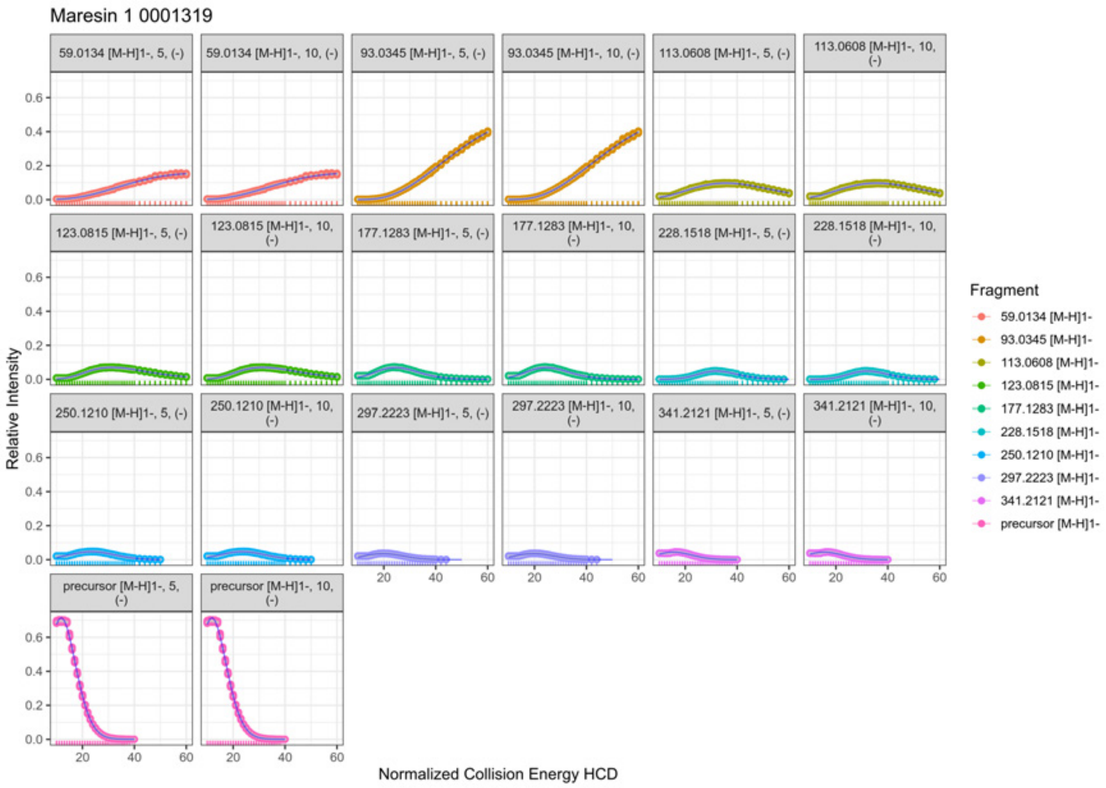

Figure 211. Nonlinear fit

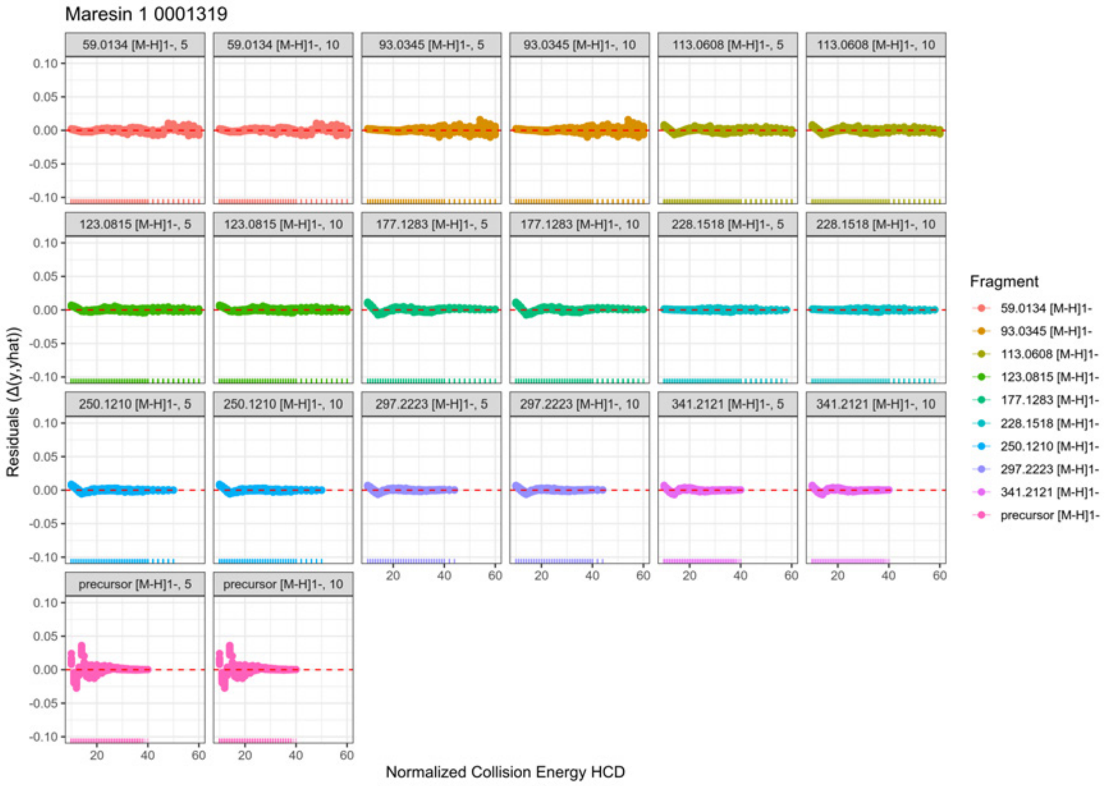

Figure 212. Residuals of nonlinear fit

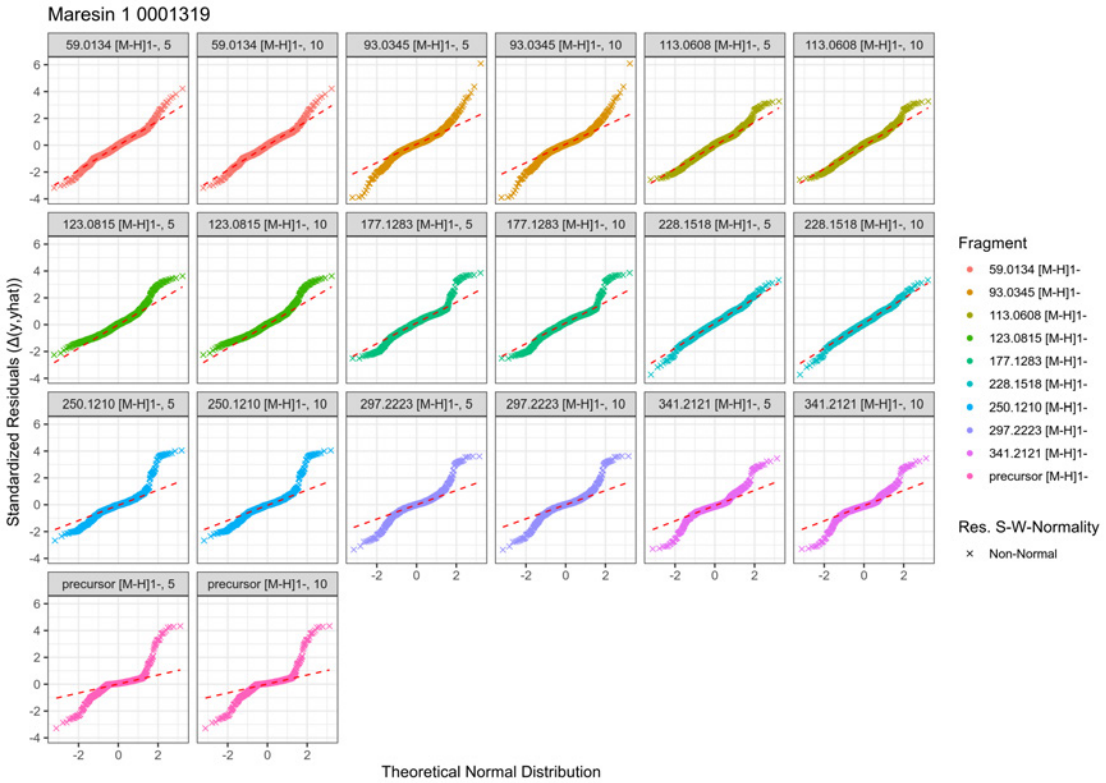

Figure 213. Quantile-quantile plot of residuals

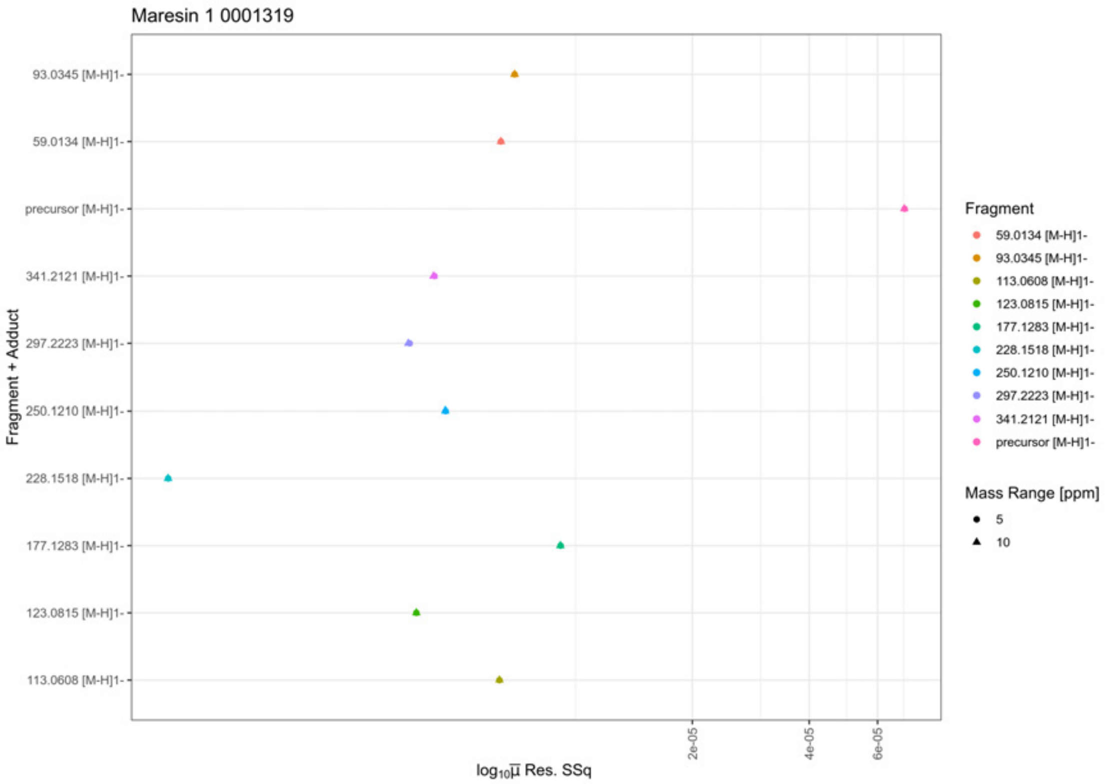

Figure 214. Normalized sum-of-squares of the residuals

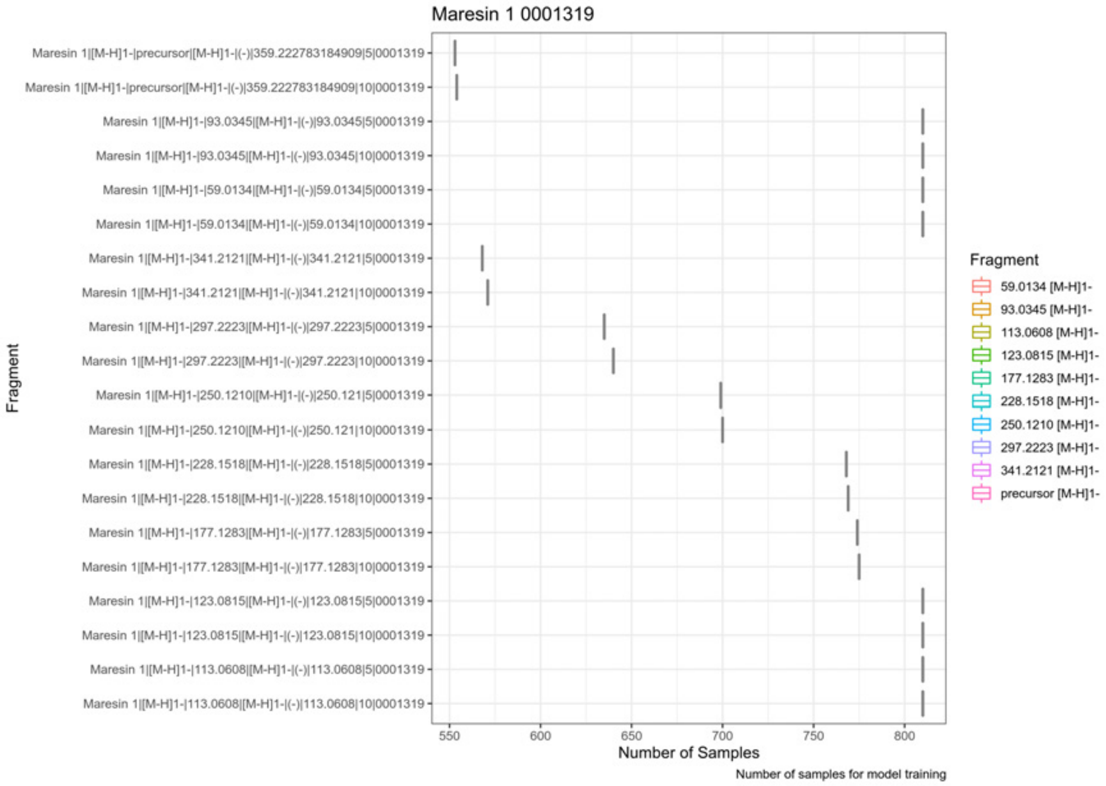

Figure 215. Number of samples used for training per combination Id

1.44. PGB2{d4} [M-H]1- 0000151

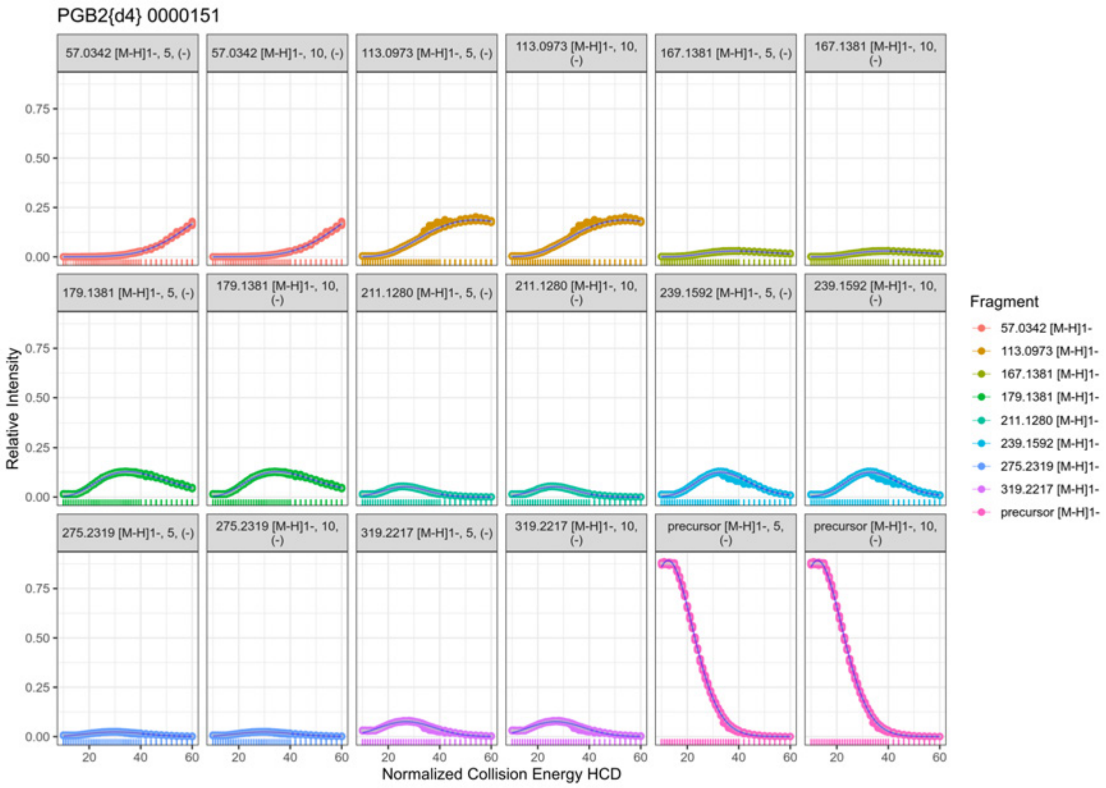

Figure 216. Nonlinear fit

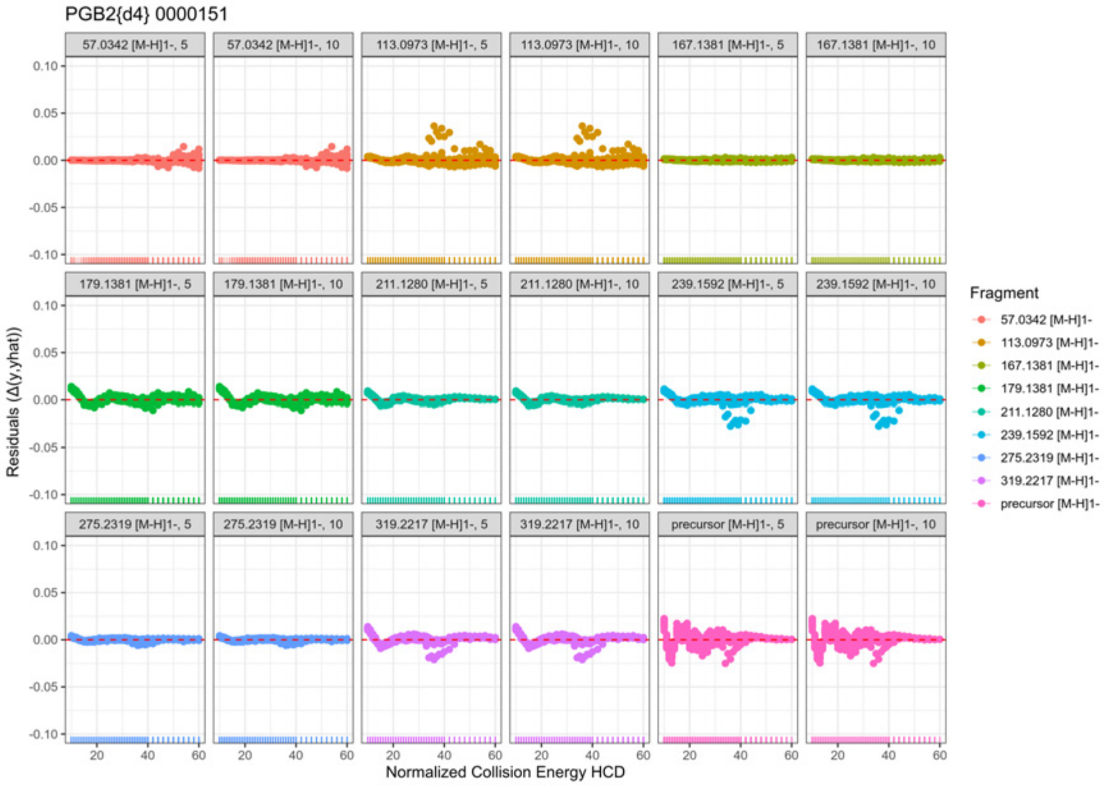

Figure 217. Residuals of nonlinear fit

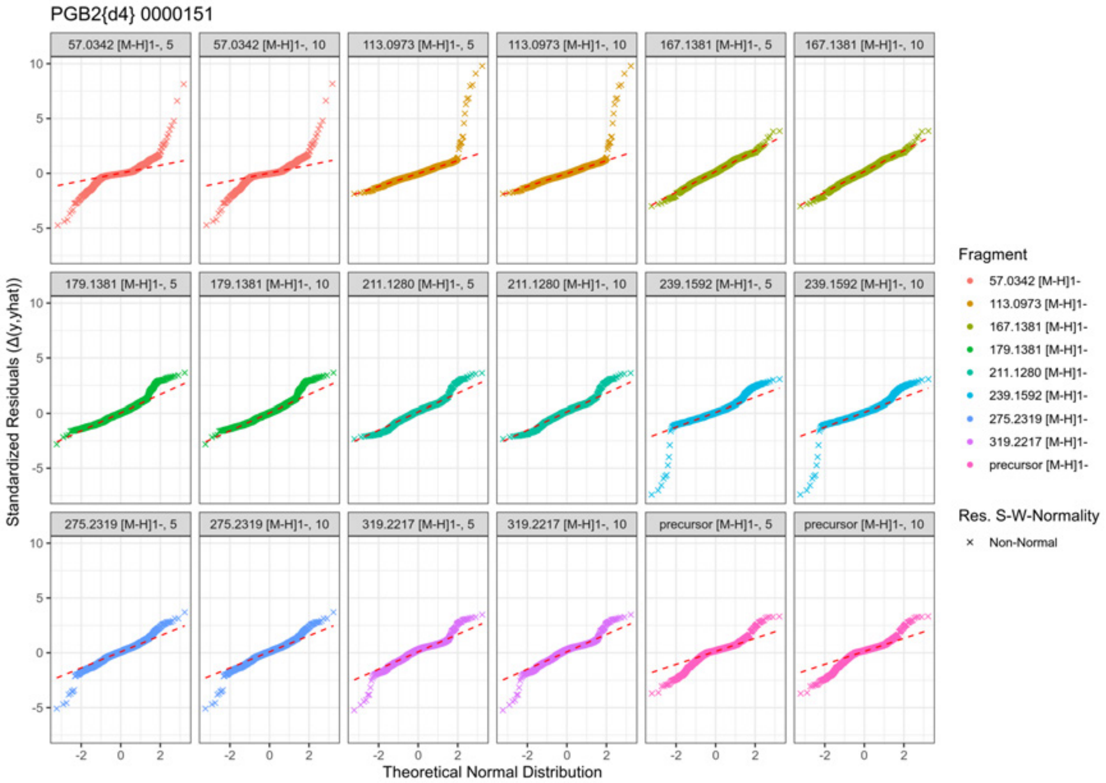

Figure 218. Quantile-quantile plot of residuals

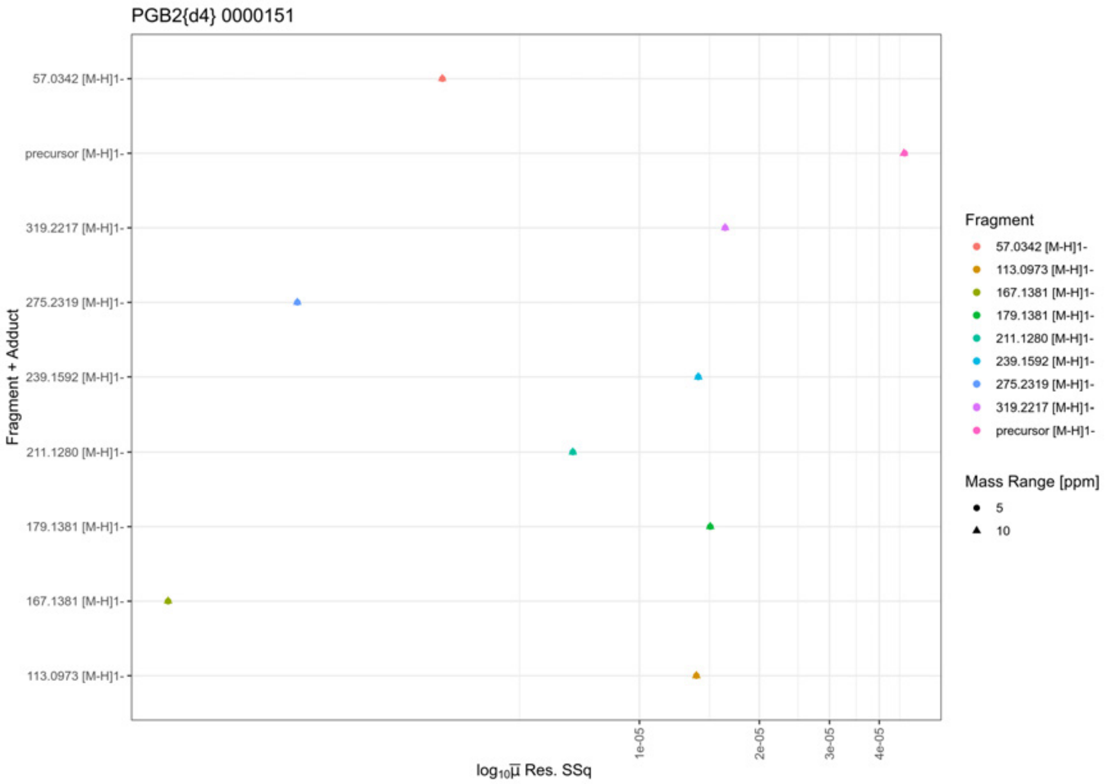

Figure 219. Normalized sum-of-squares of the residuals

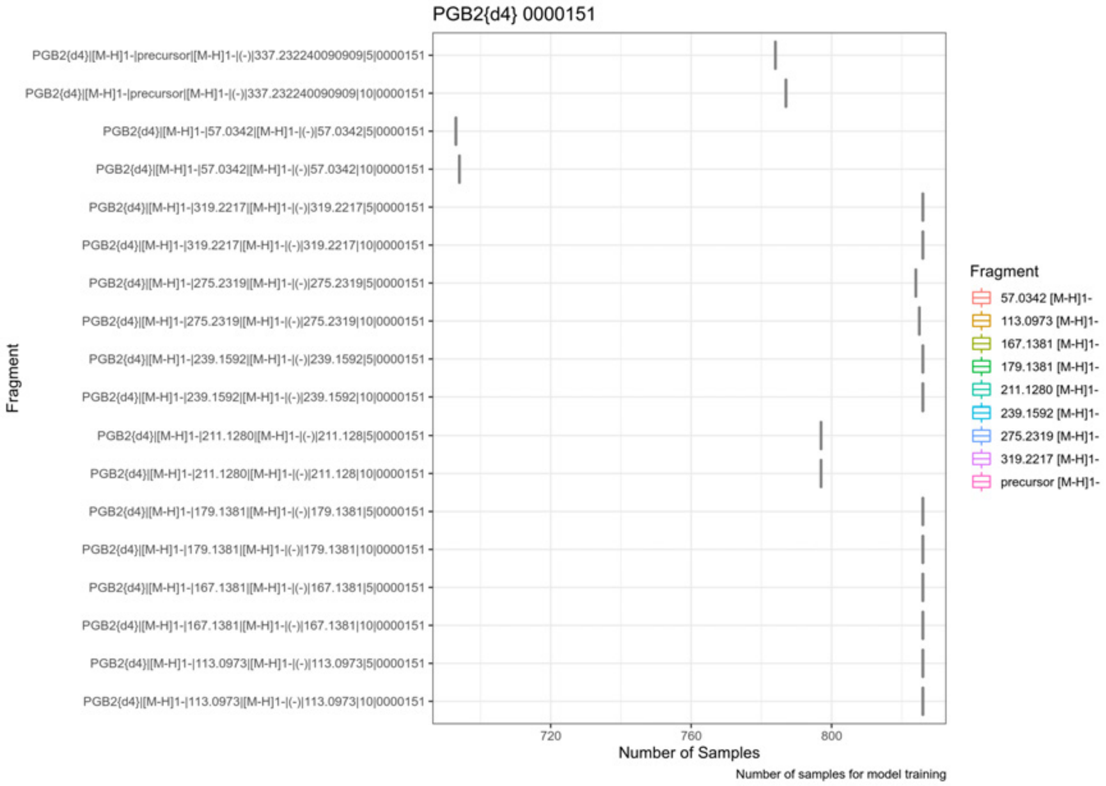

Figure 220. Number of samples used for training per combination Id

1.45. PGD2{d4} [M-H]1- 0001311

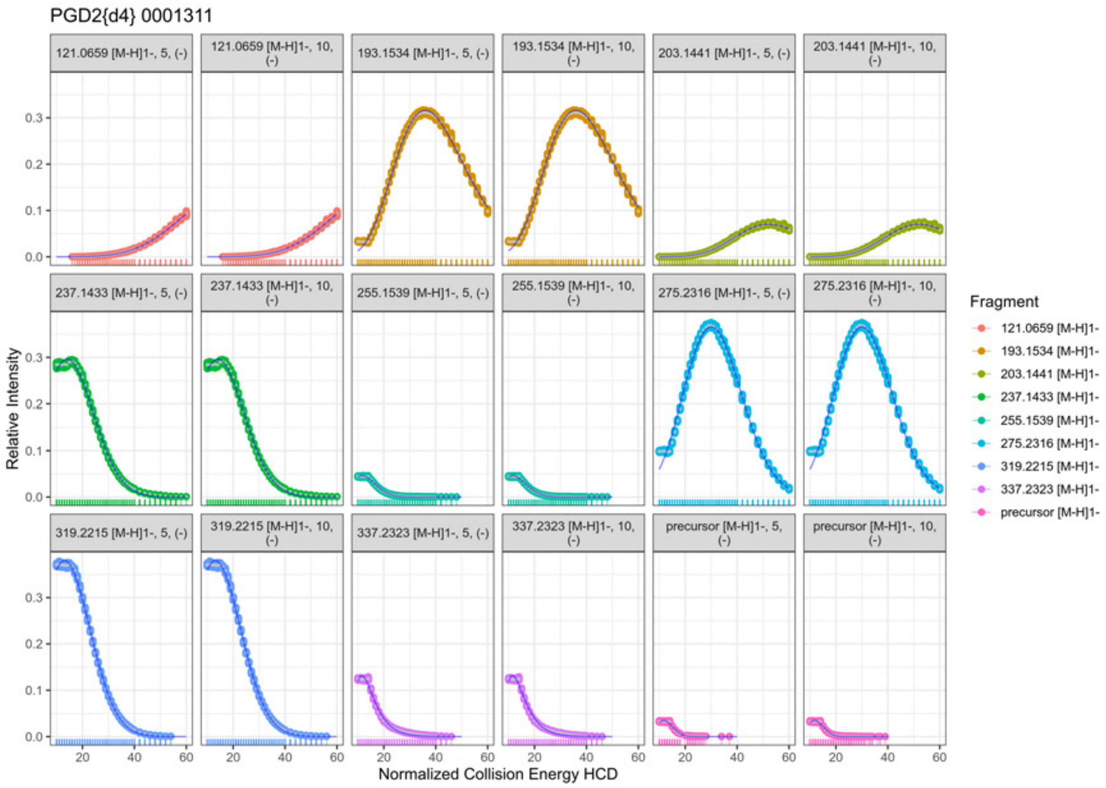

Figure 221. Nonlinear fit

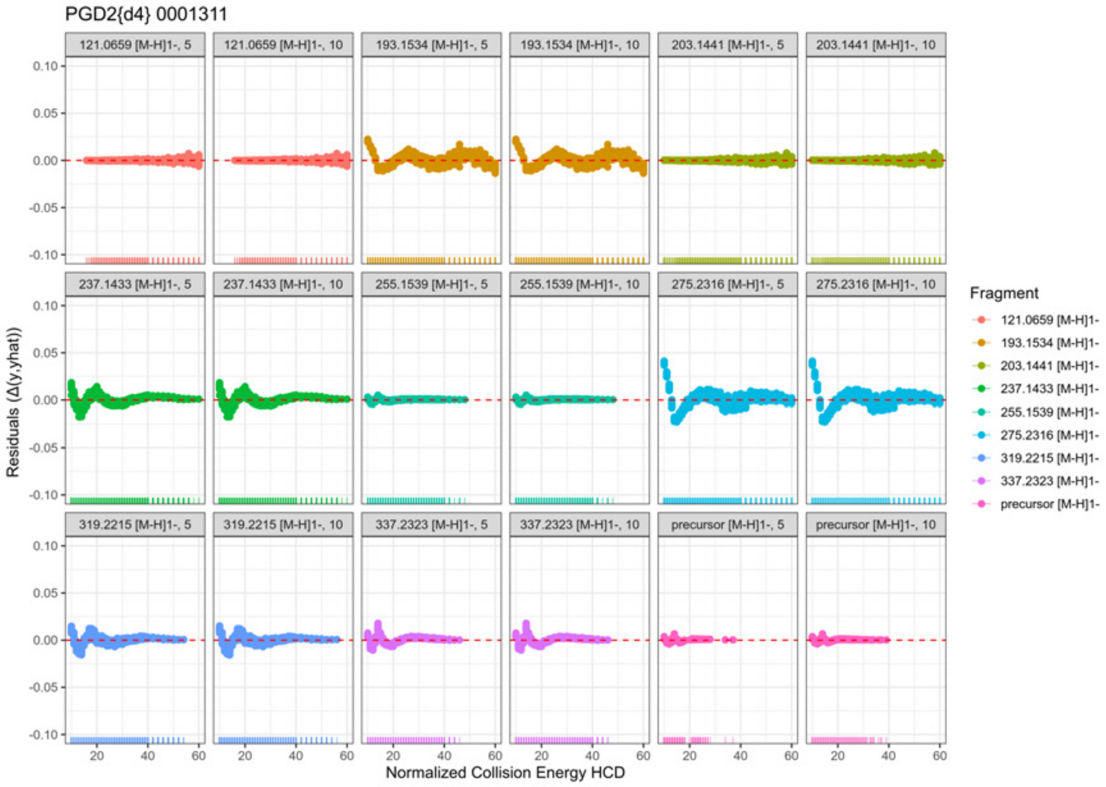

Figure 222. Residuals of nonlinear fit

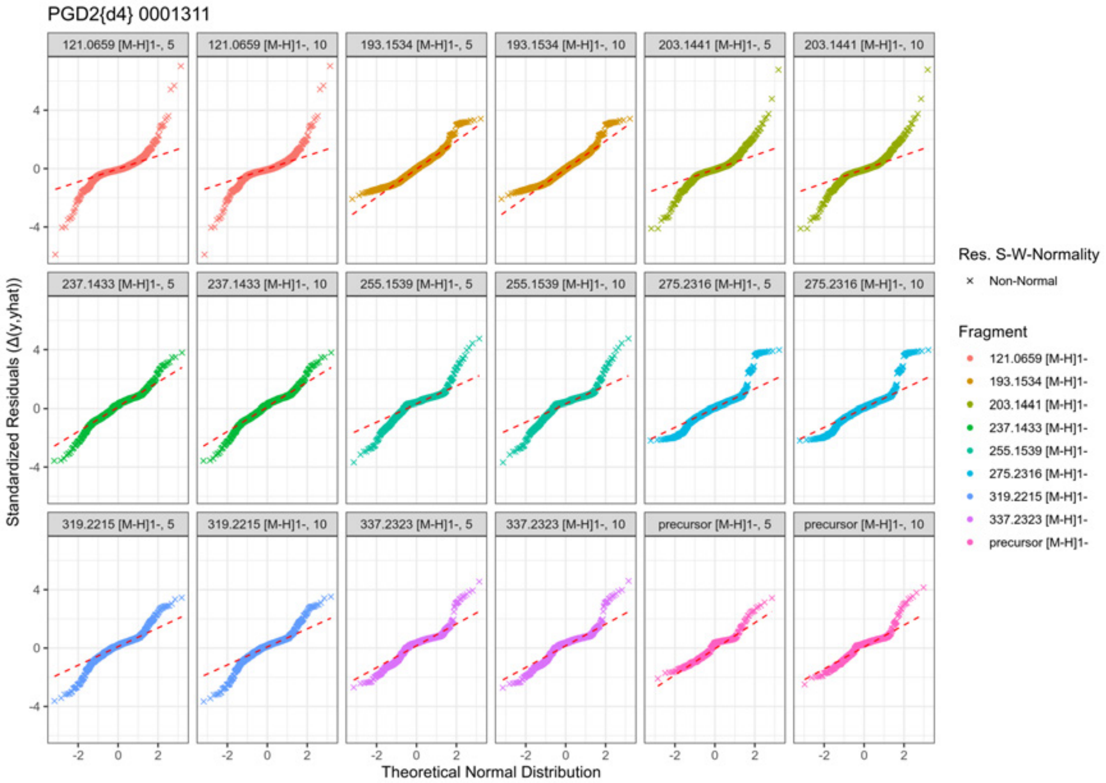

Figure 223. Quantile-quantile plot of residuals

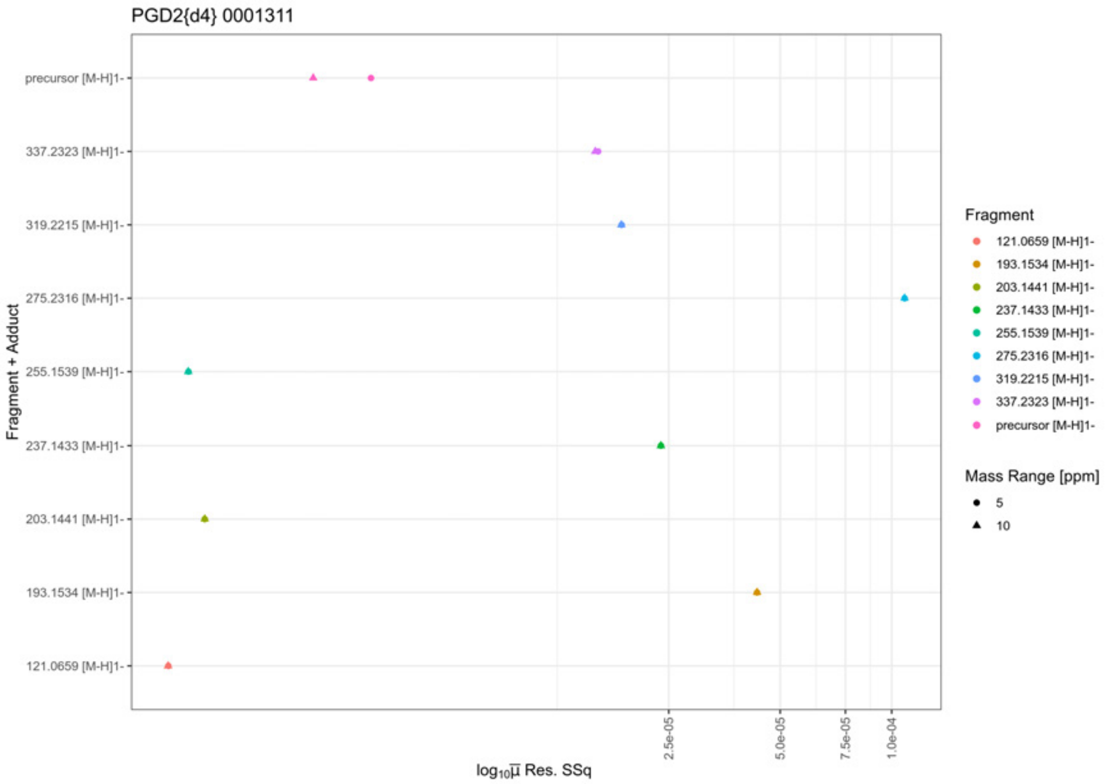

Figure 224. Normalized sum-of-squares of the residuals

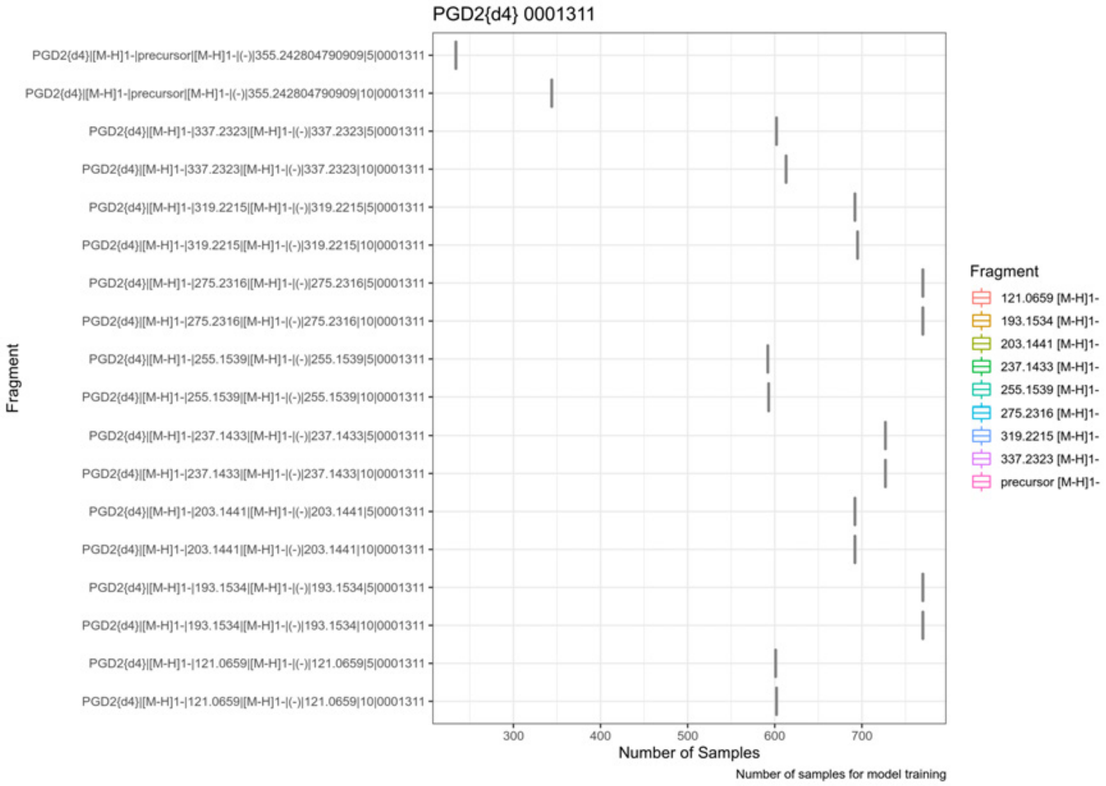

Figure 225. Number of samples used for training per combination Id

1.46. PGE2{d4} [M-H]1- 0001313

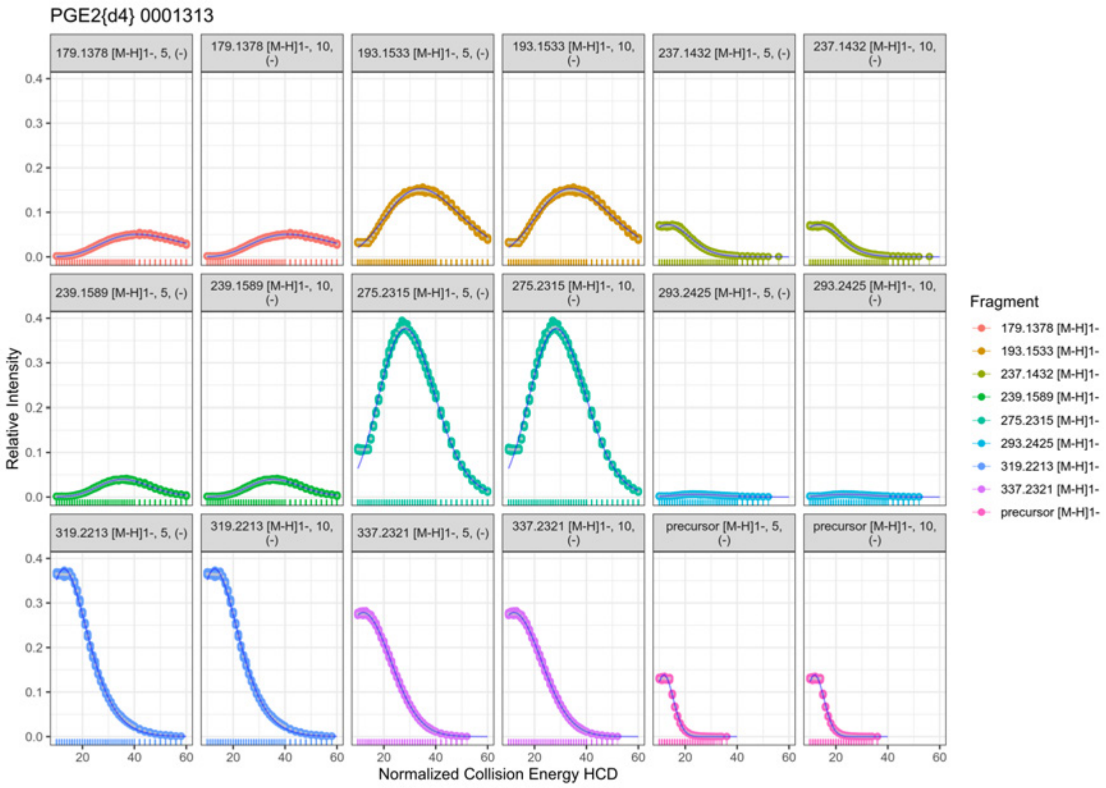

Figure 226. Nonlinear fit

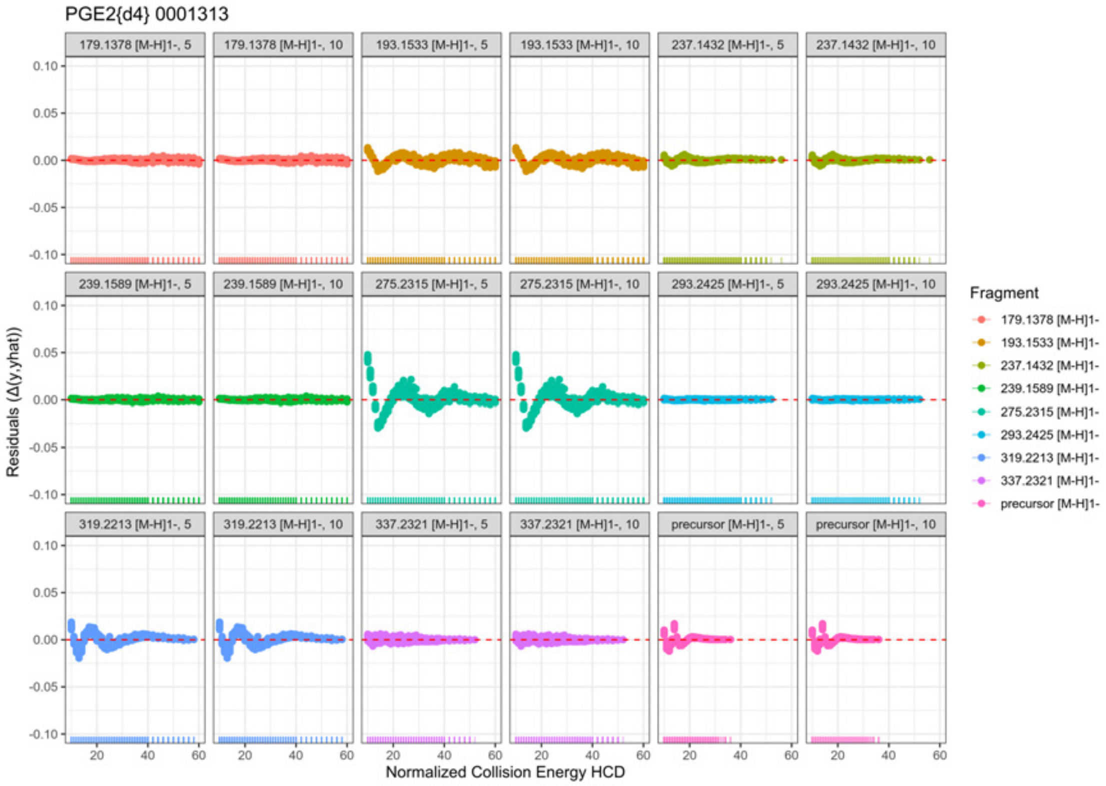

Figure 227. Residuals of nonlinear fit

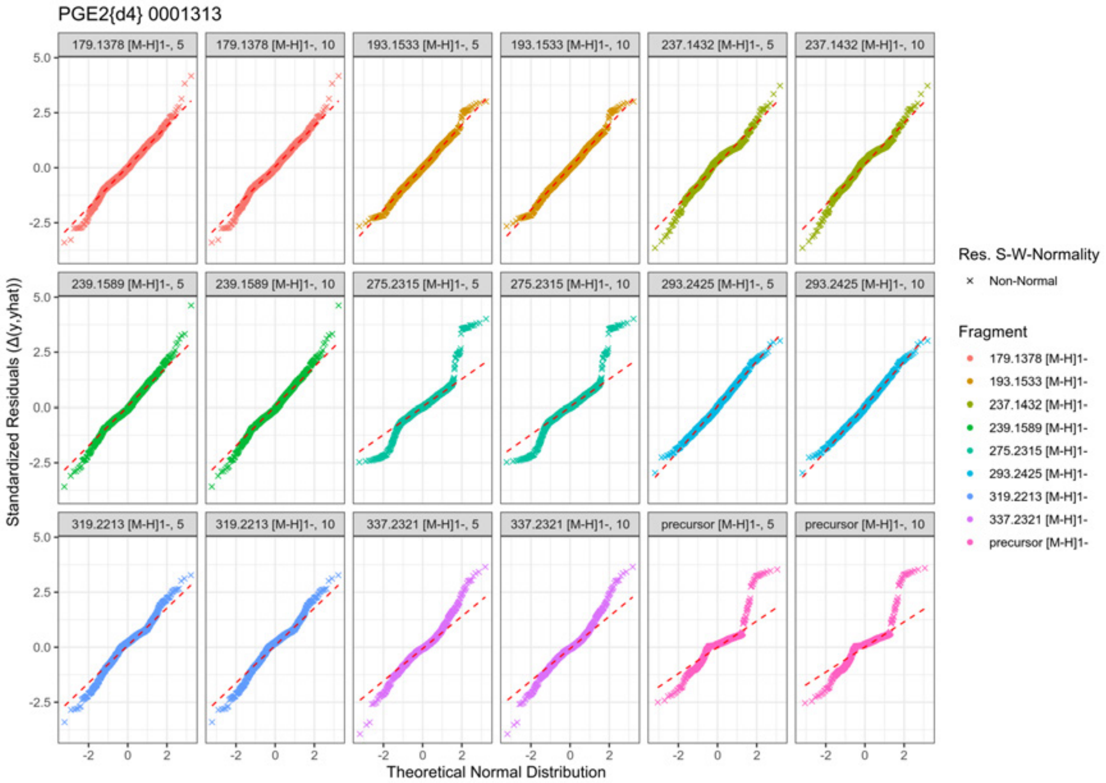

Figure 228. Quantile-quantile plot of residuals

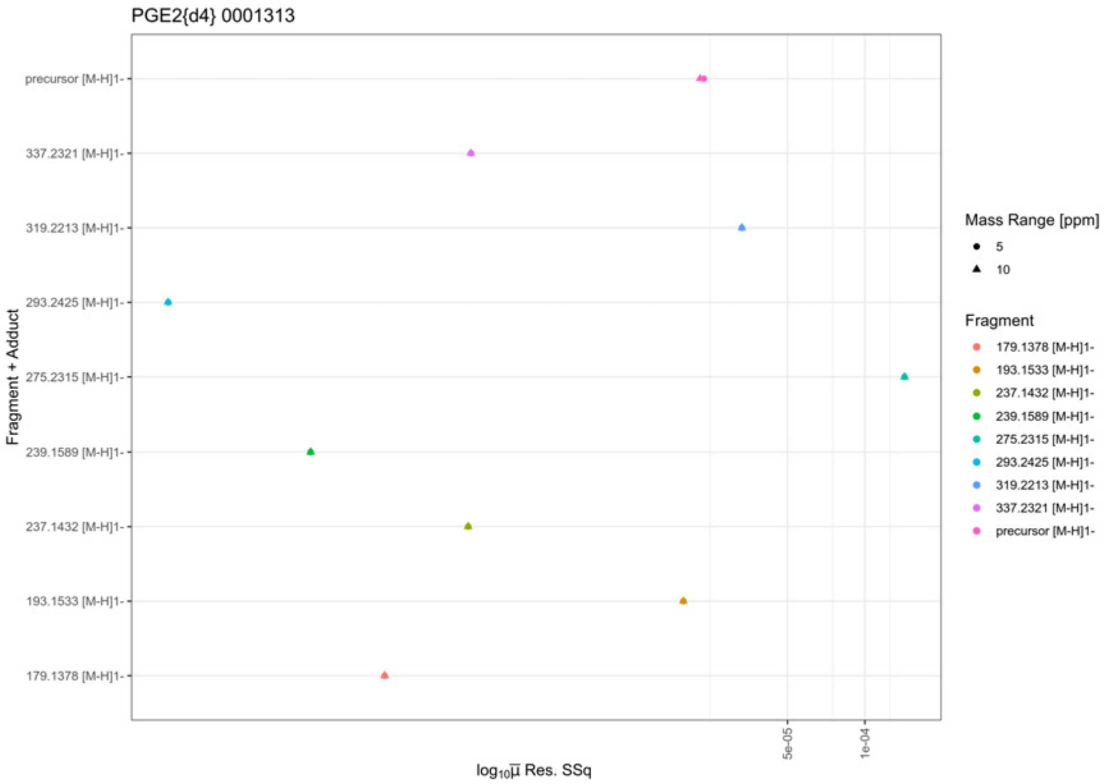

Figure 229. Normalized sum-of-squares of the residuals

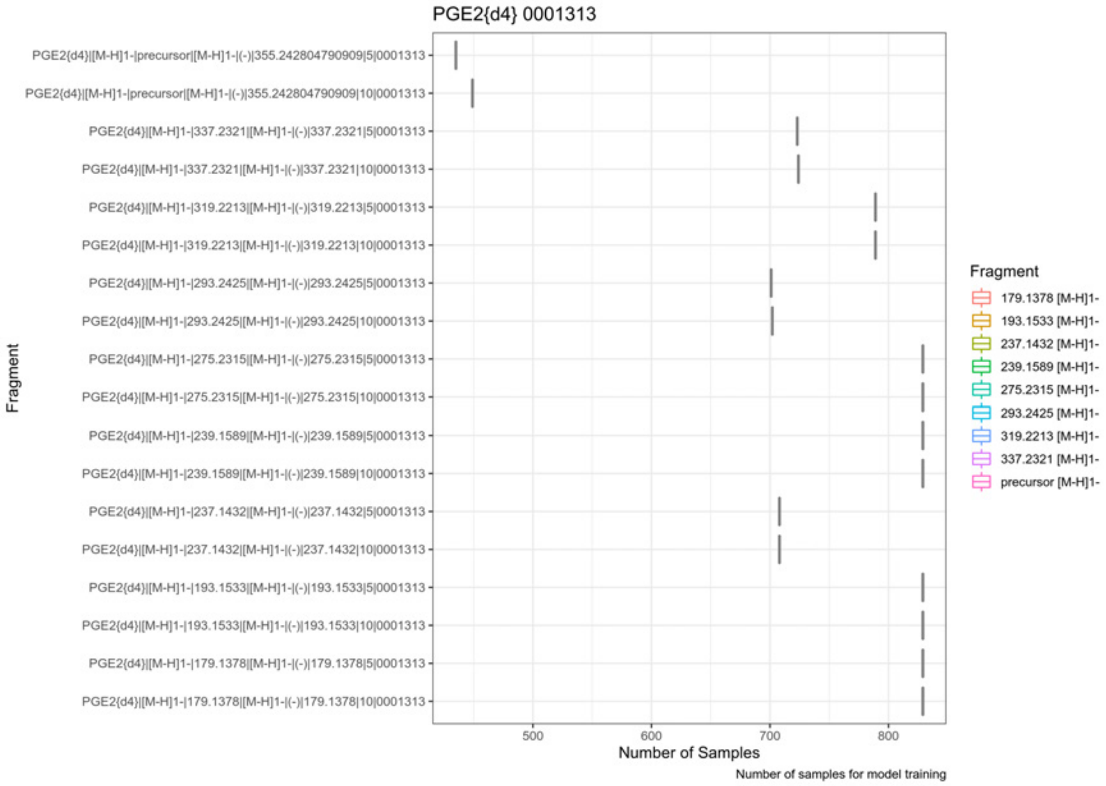

Figure 230. Number of samples used for training per combination Id

1.47. PGE2{d9} [M-H]1- 0001321

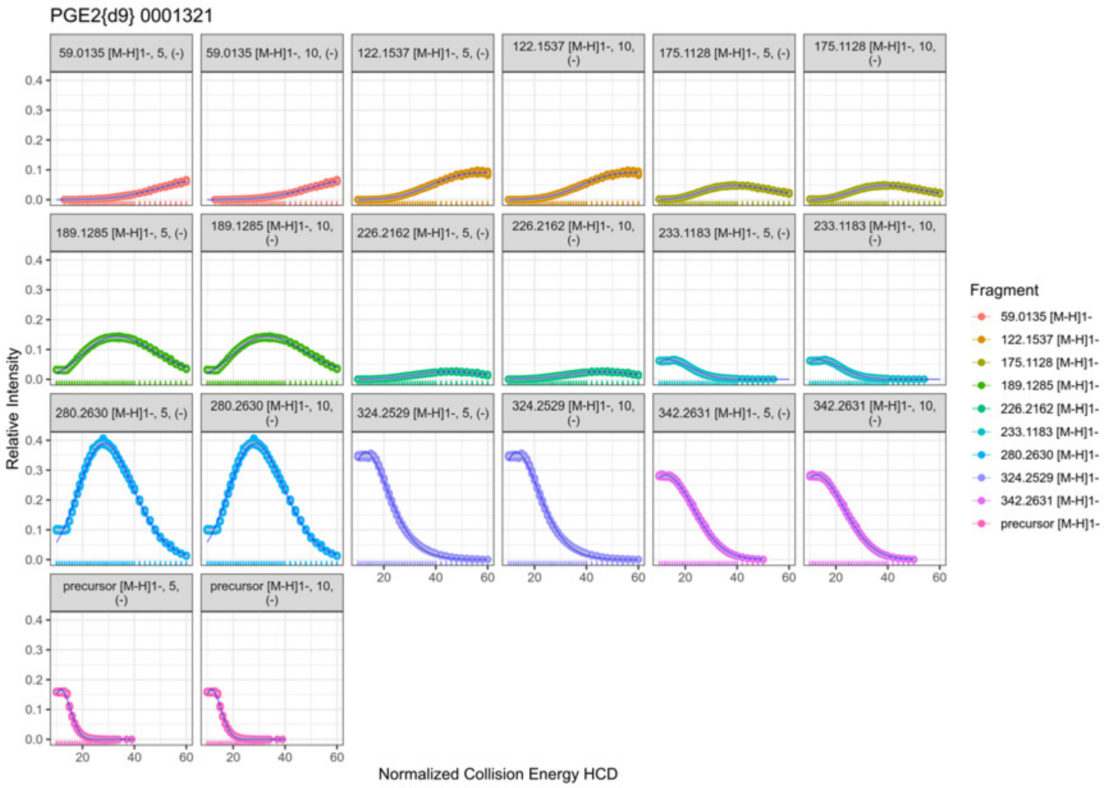

Figure 231. Nonlinear fit

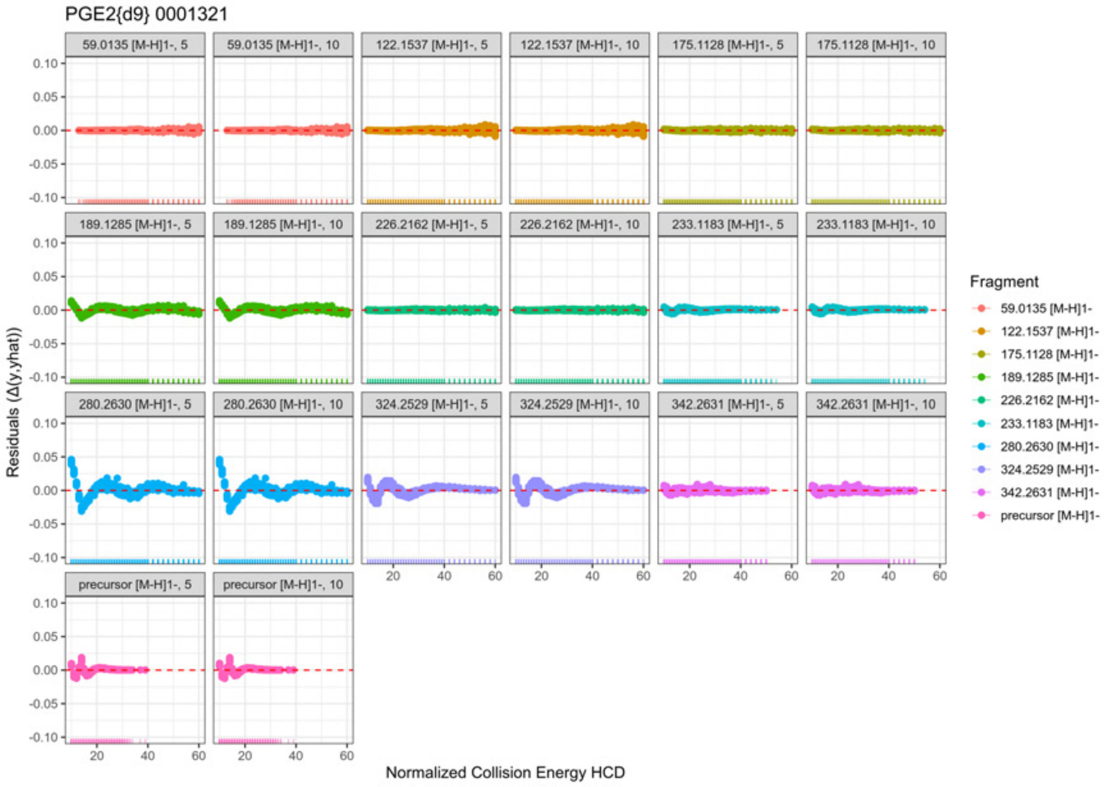

Figure 232. Residuals of nonlinear fit

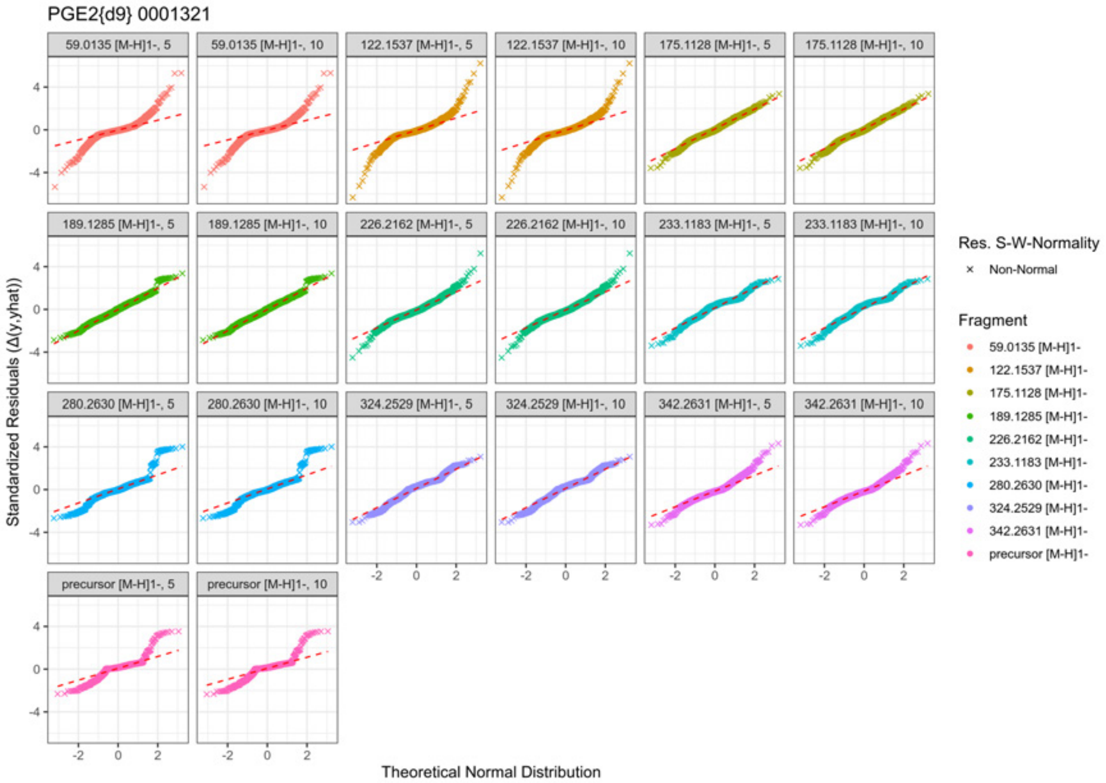

Figure 233. Quantile-quantile plot of residuals

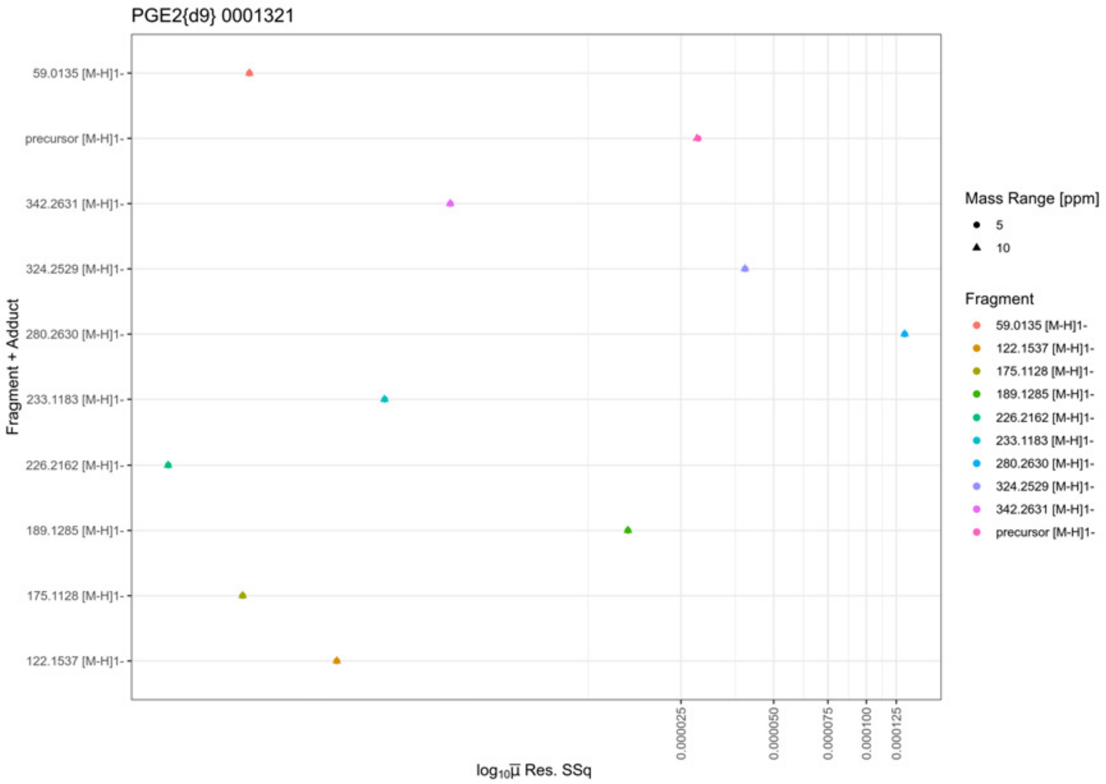

Figure 234. Normalized sum-of-squares of the residuals

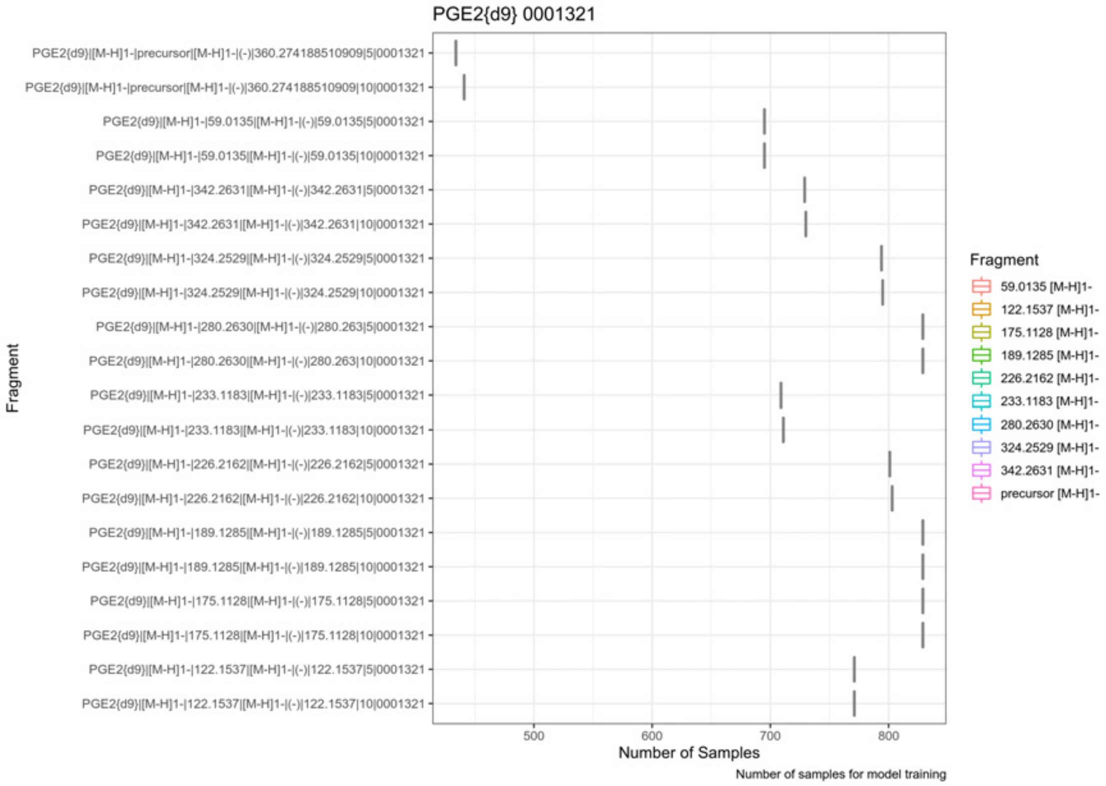

Figure 235. Number of samples used for training per combination Id

1.48. PGF2alpha{d4} [M-H]1- 0000125

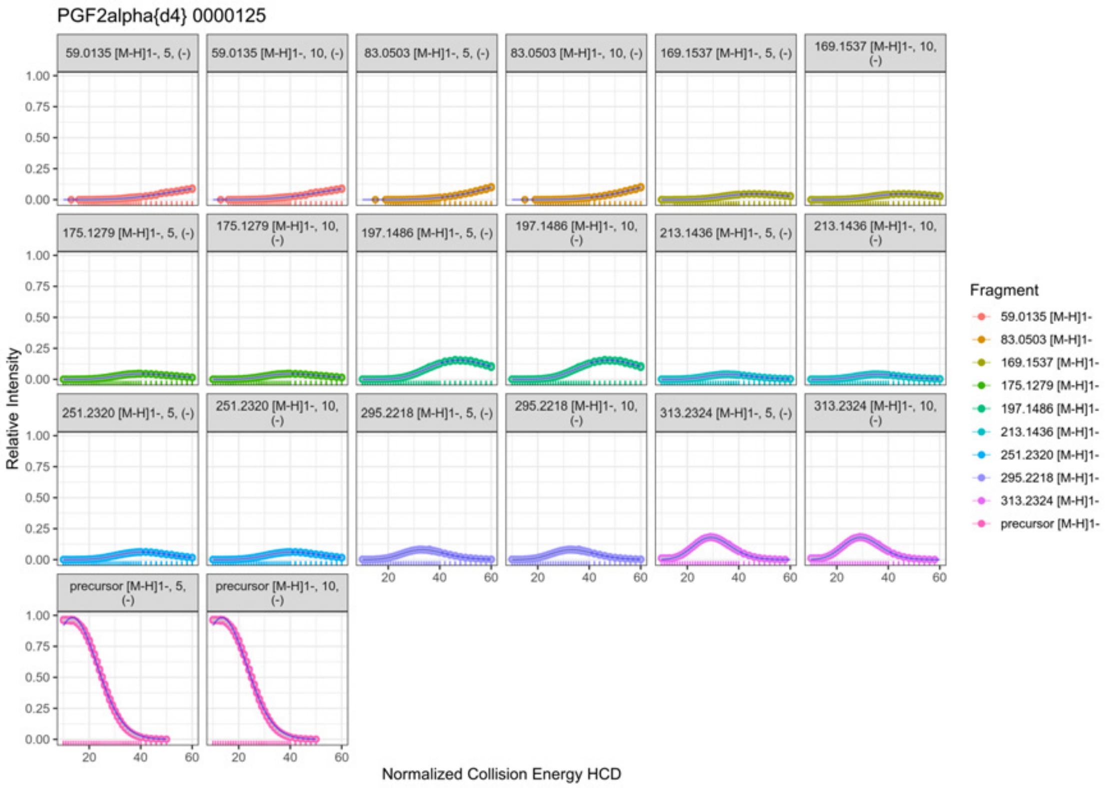

Figure 236. Nonlinear fit

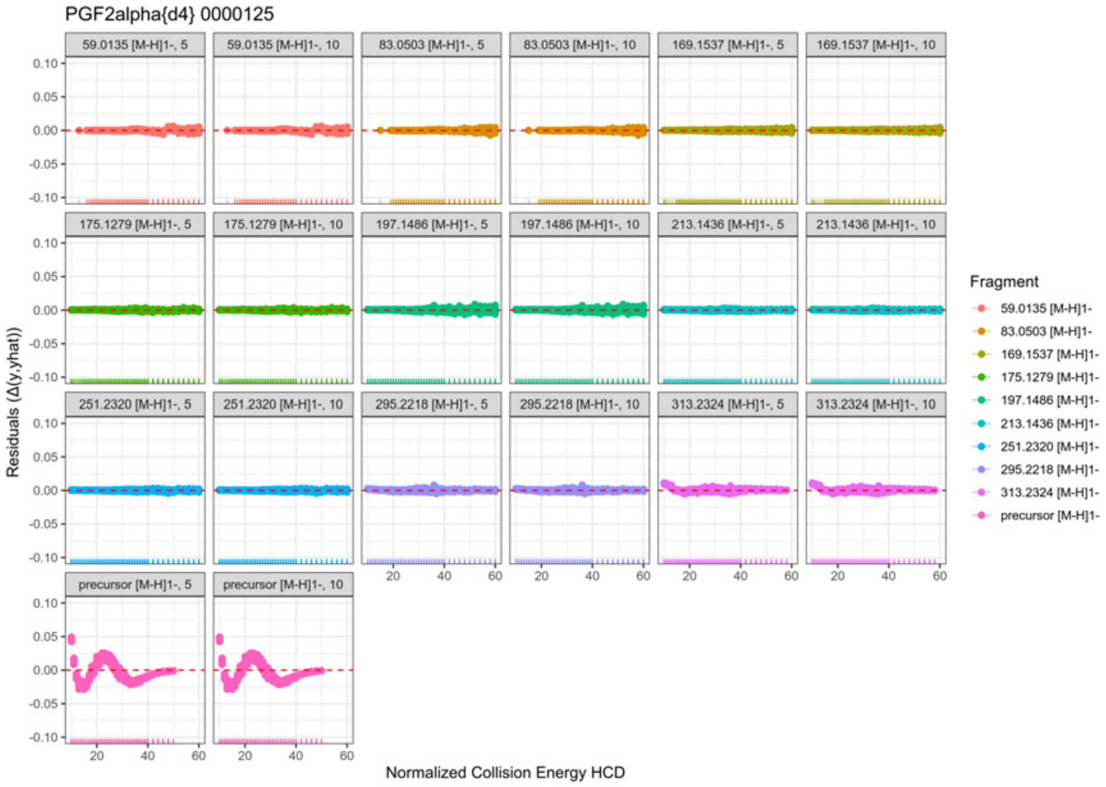

Figure 237. Residuals of nonlinear fit

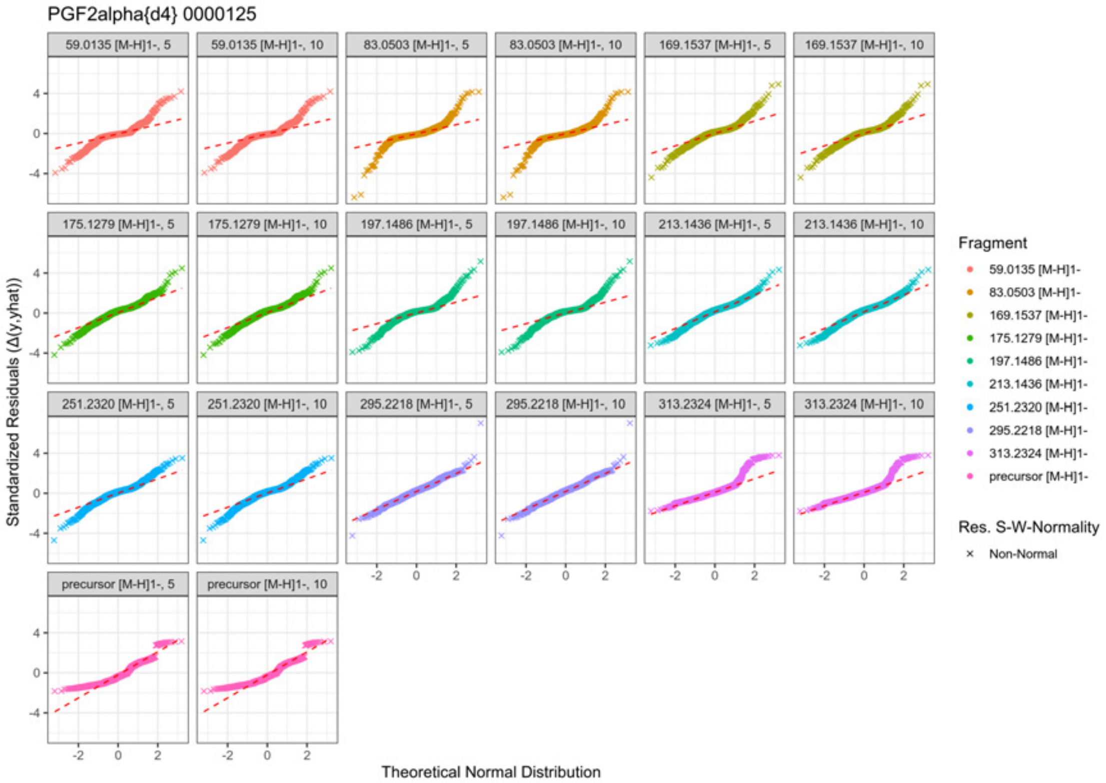

Figure 238. Quantile-quantile plot of residuals

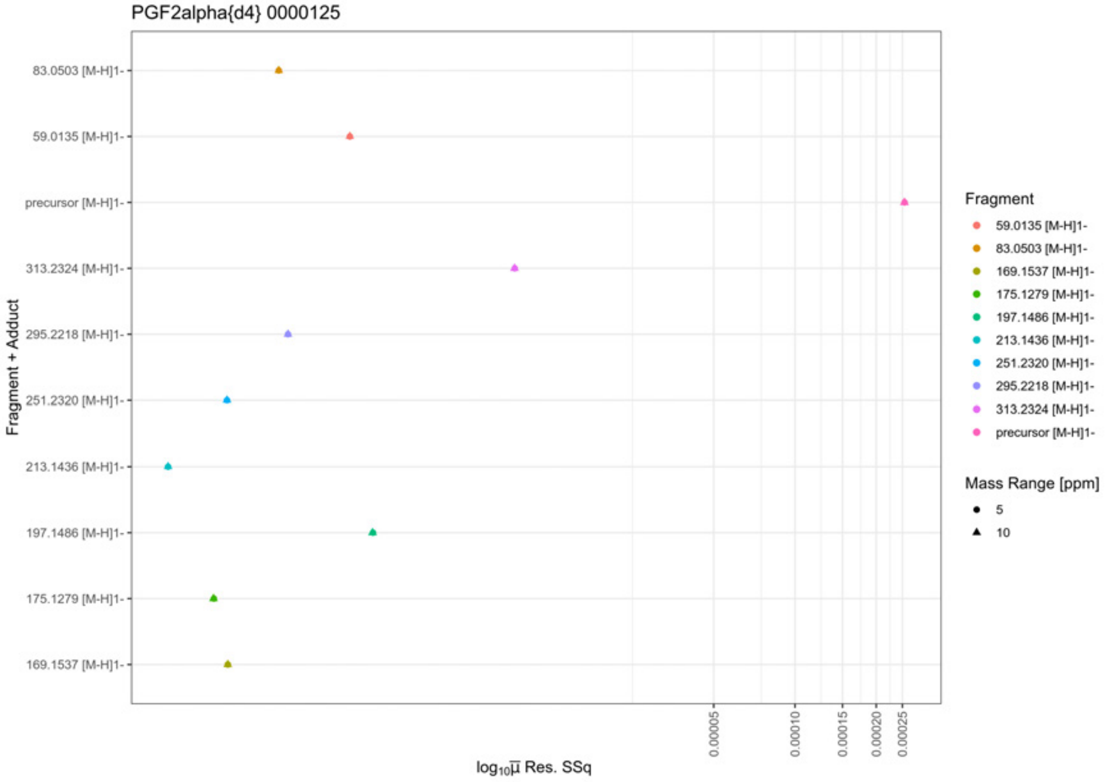

Figure 239. Normalized sum-of-squares of the residuals

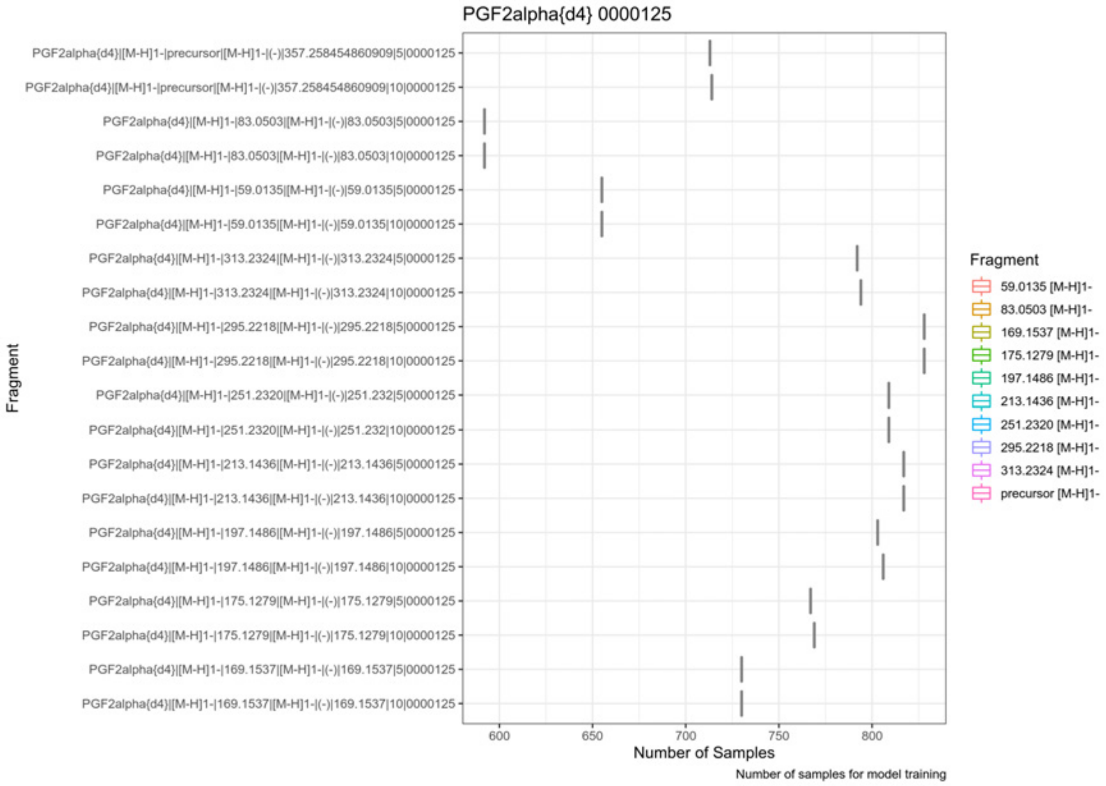

Figure 240. Number of samples used for training per combination Id

1.49. PGI2 [M-H]1- 0000127

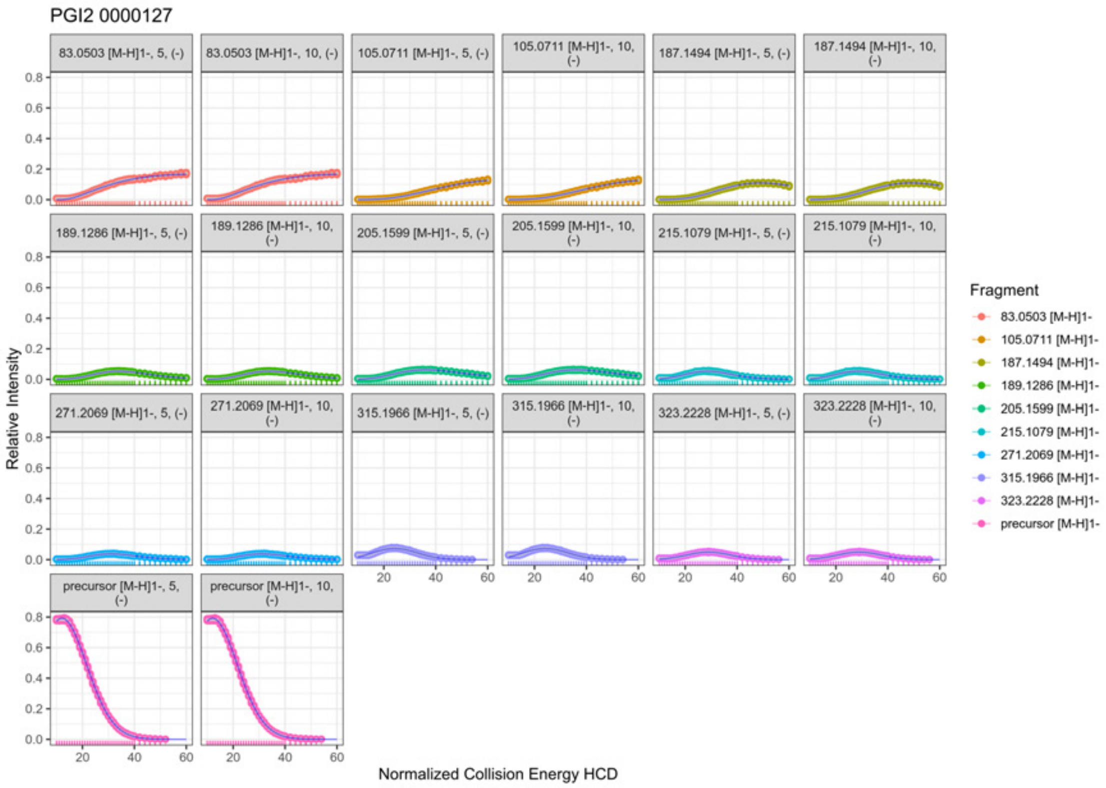

Figure 241. Nonlinear fit

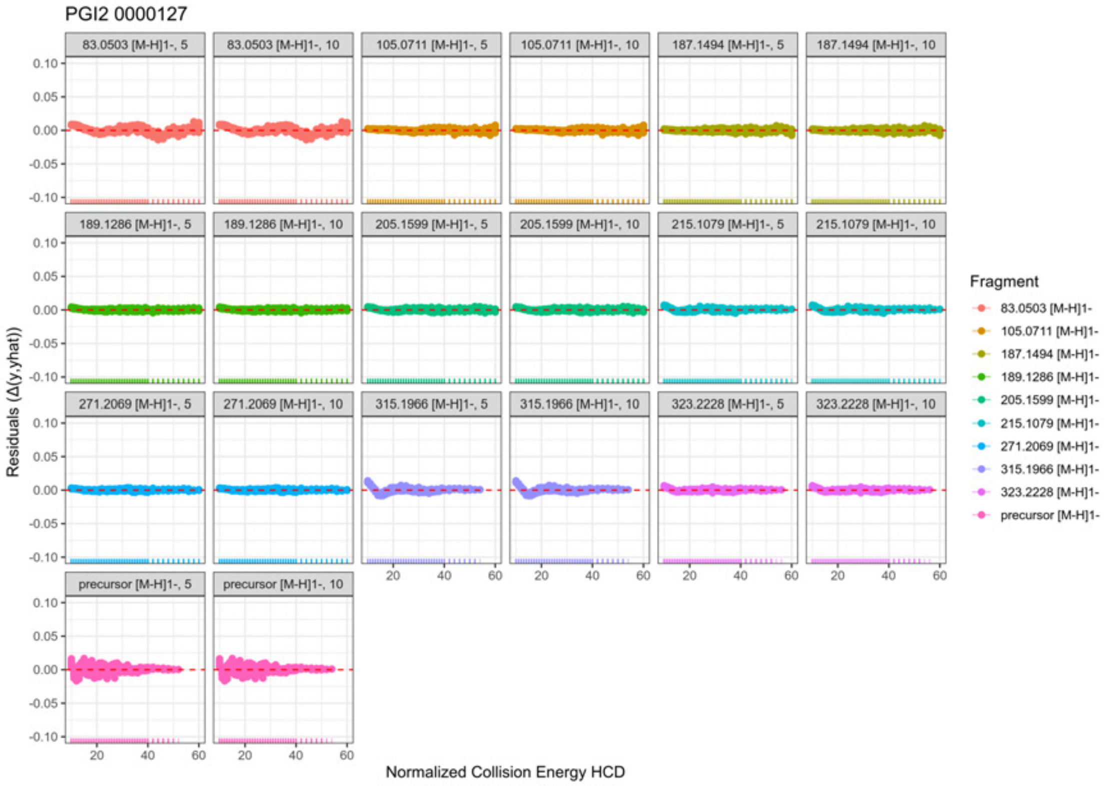

Figure 242. Residuals of nonlinear fit

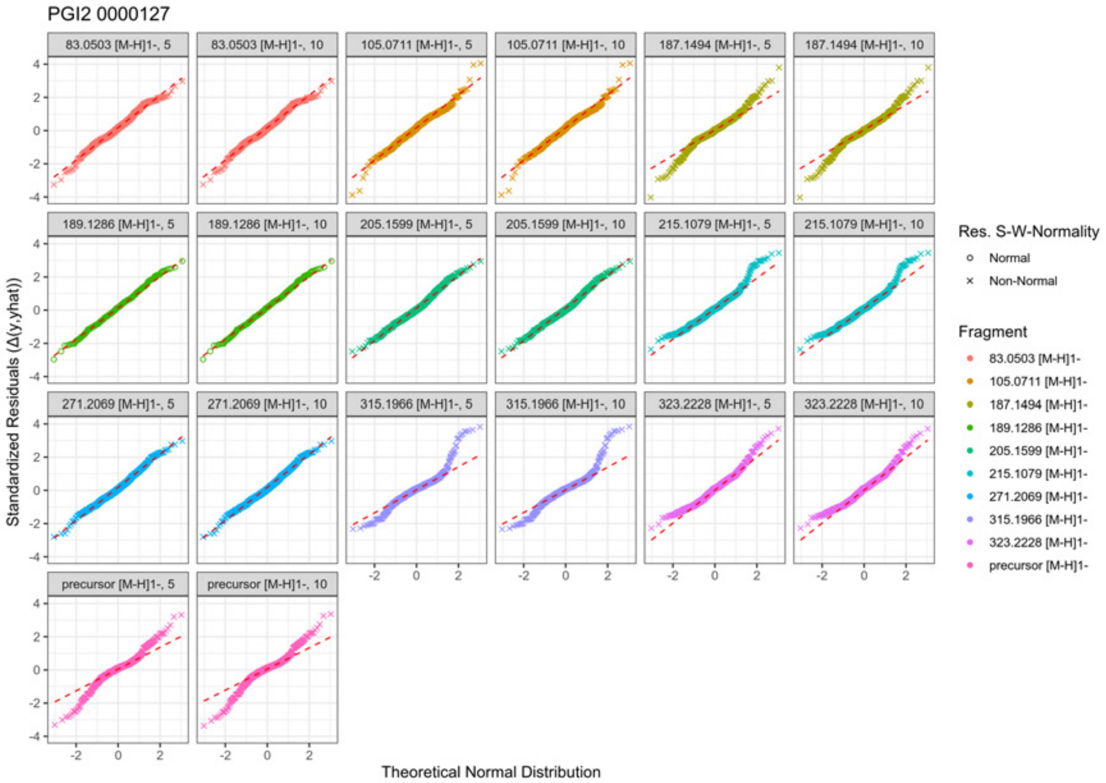

Figure 243. Quantile-quantile plot of residuals

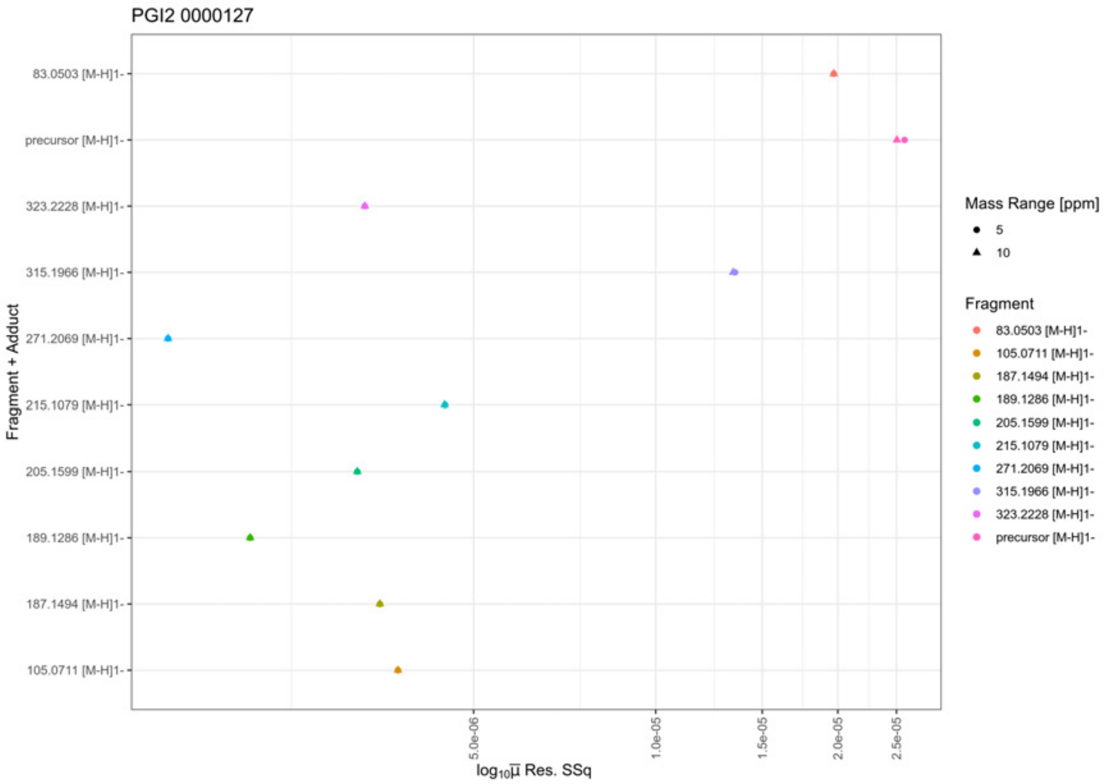

Figure 244. Normalized sum-of-squares of the residuals

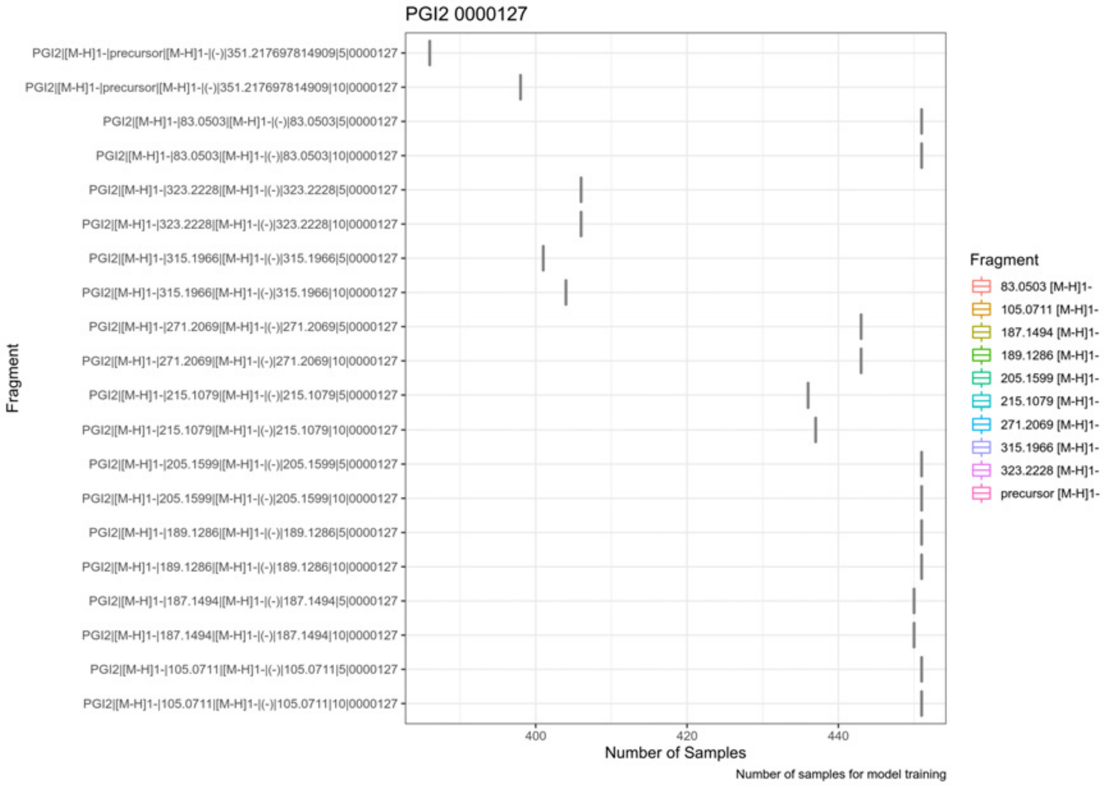

Figure 245. Number of samples used for training per combination Id

1.50. Resolvin D1{d5} [M-H]1- 0001333

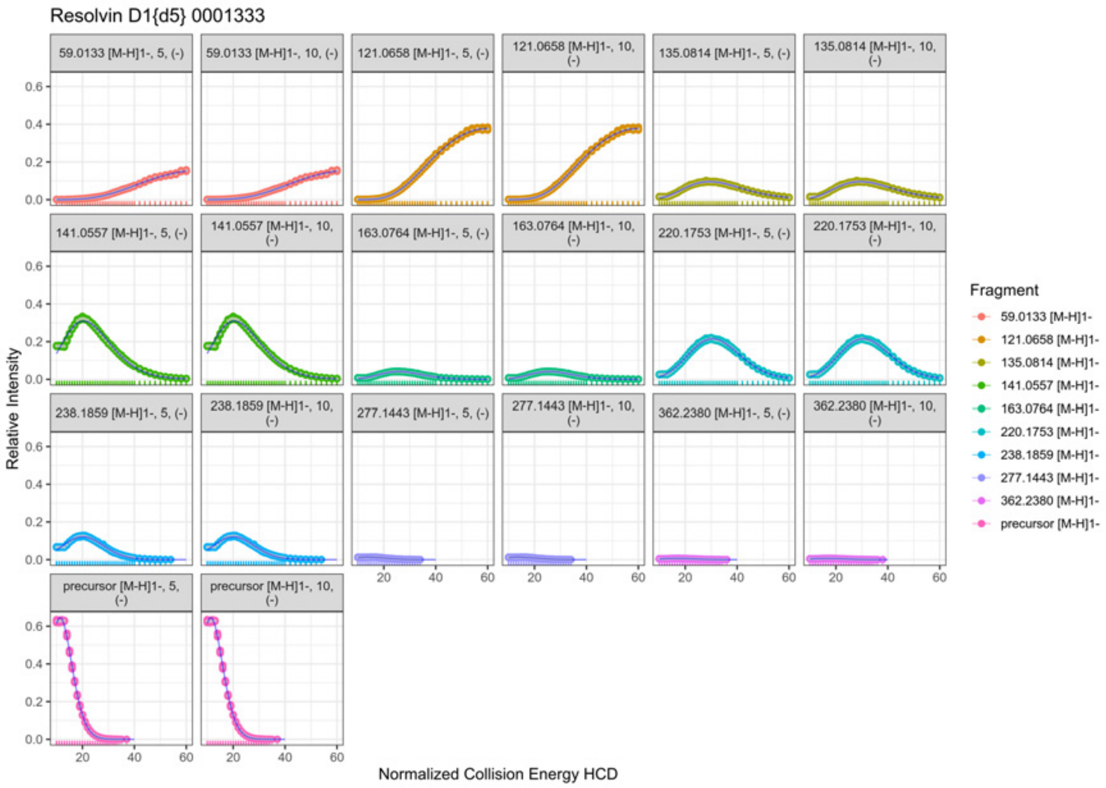

Figure 246. Nonlinear fit

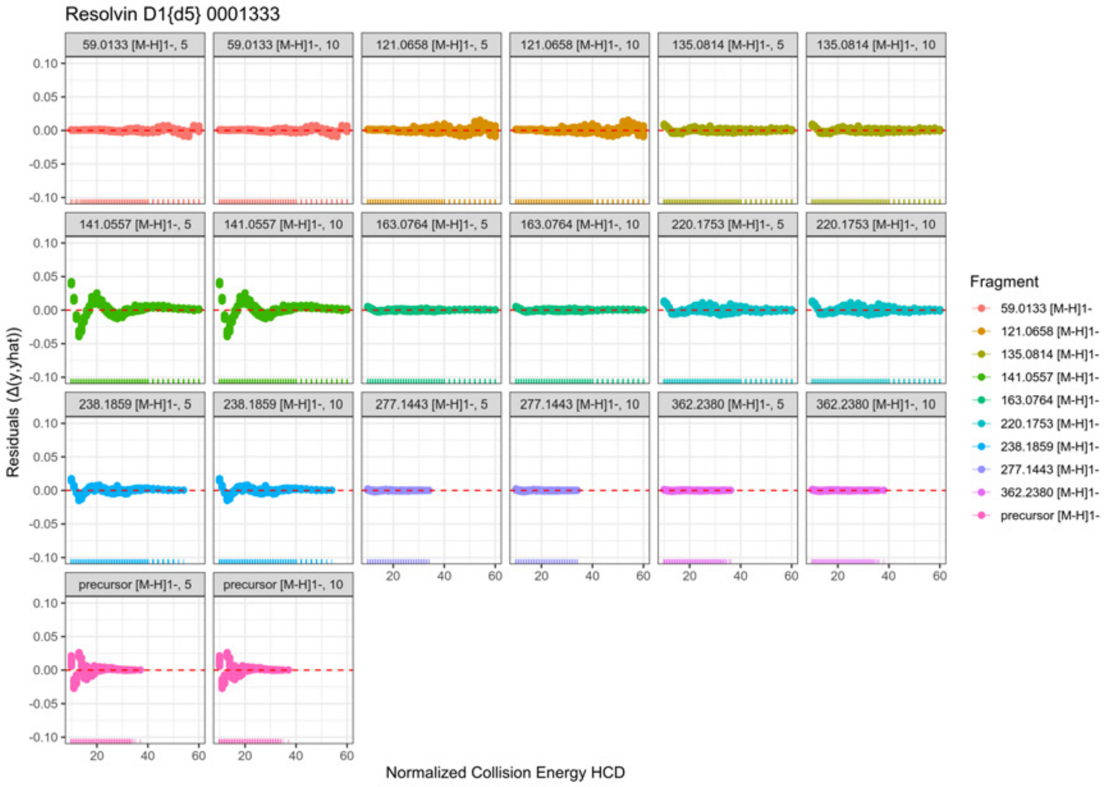

Figure 247. Residuals of nonlinear fit

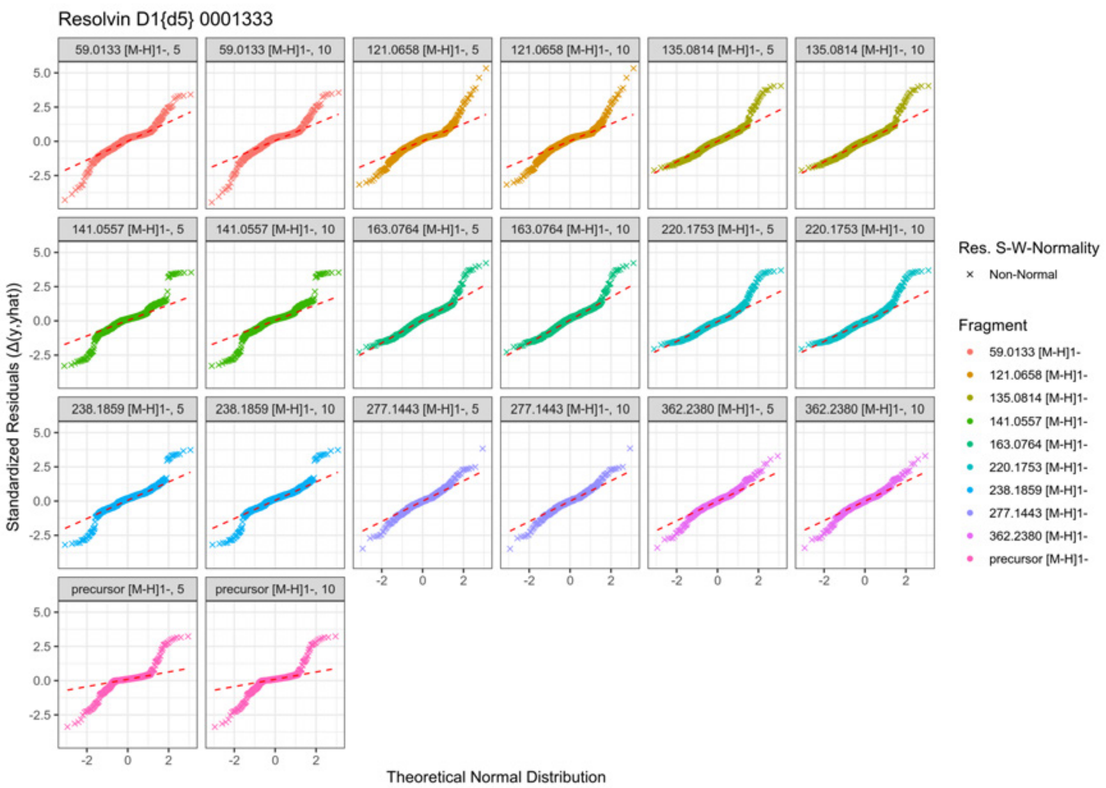

Figure 248. Quantile-quantile plot of residuals

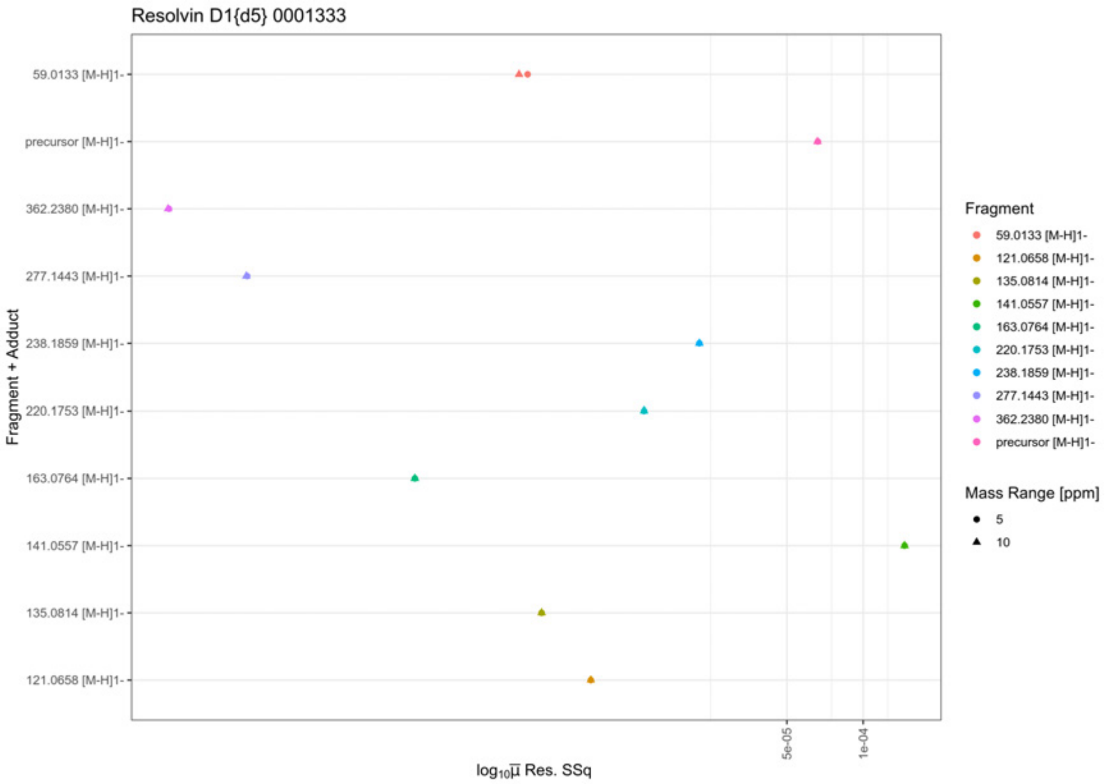

Figure 249. Normalized sum-of-squares of the residuals

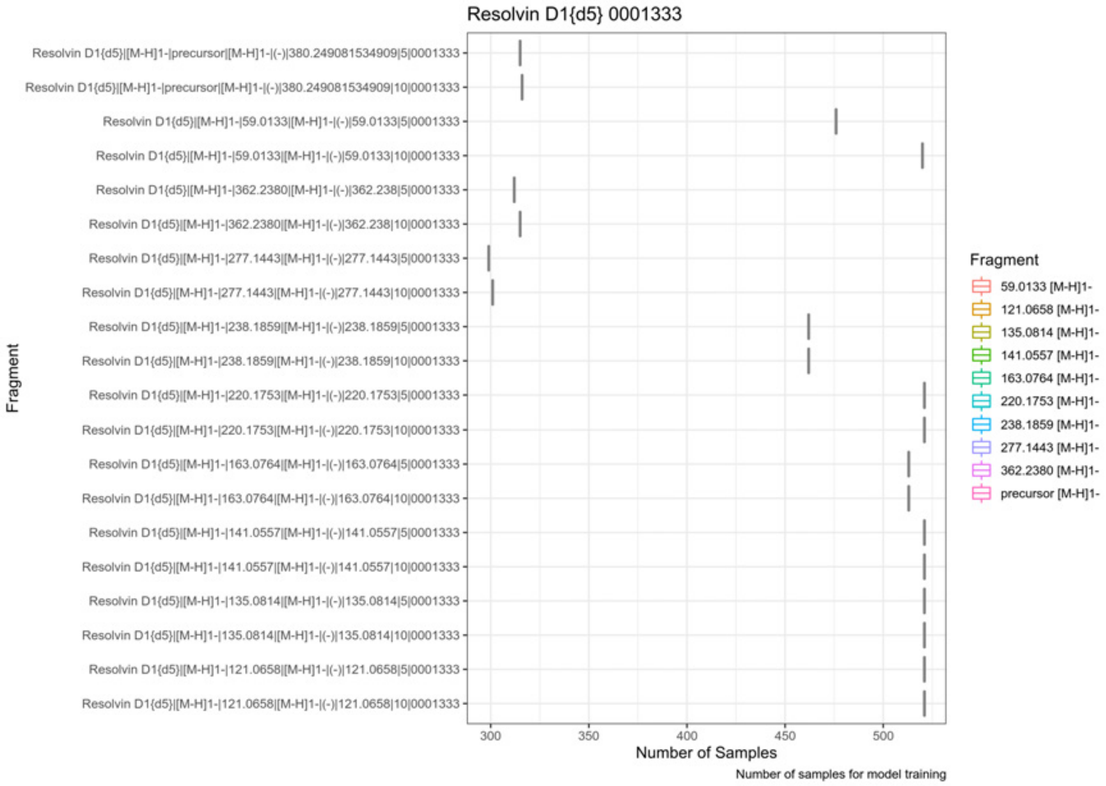

Figure 250. Number of samples used for training per combination Id

1.51. Resolvin D2{d5} [M-H]1- 0000129

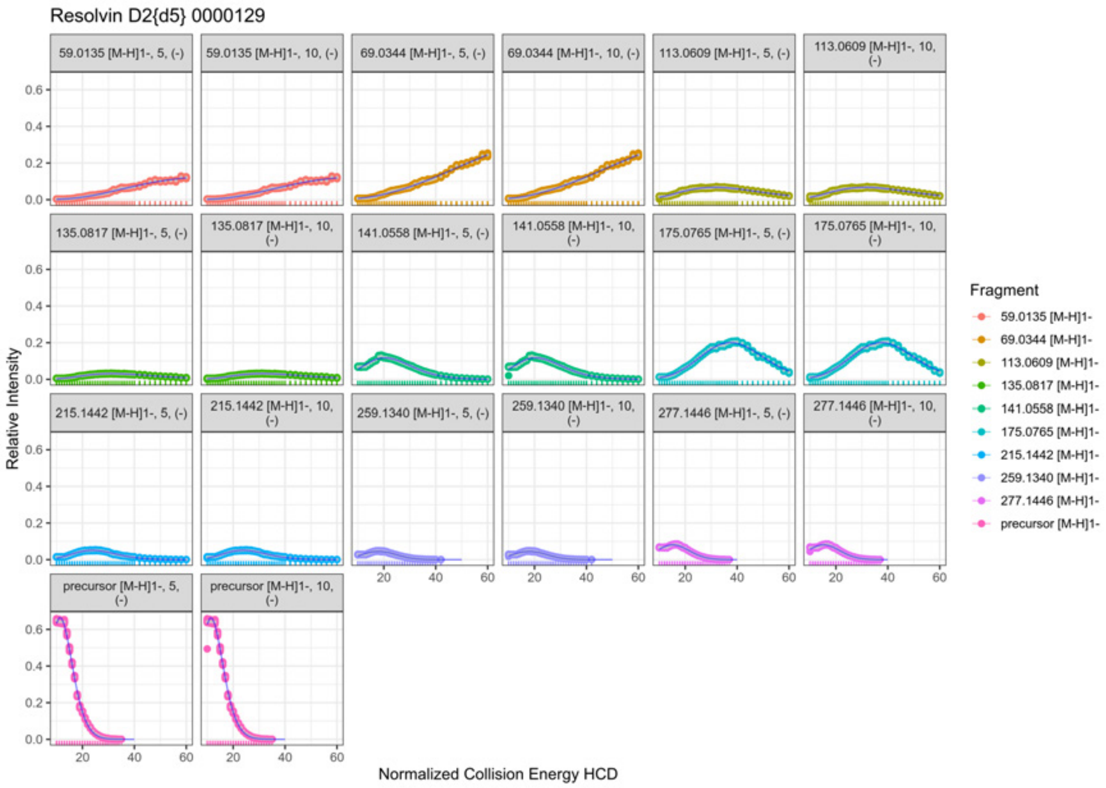

Figure 251. Nonlinear fit

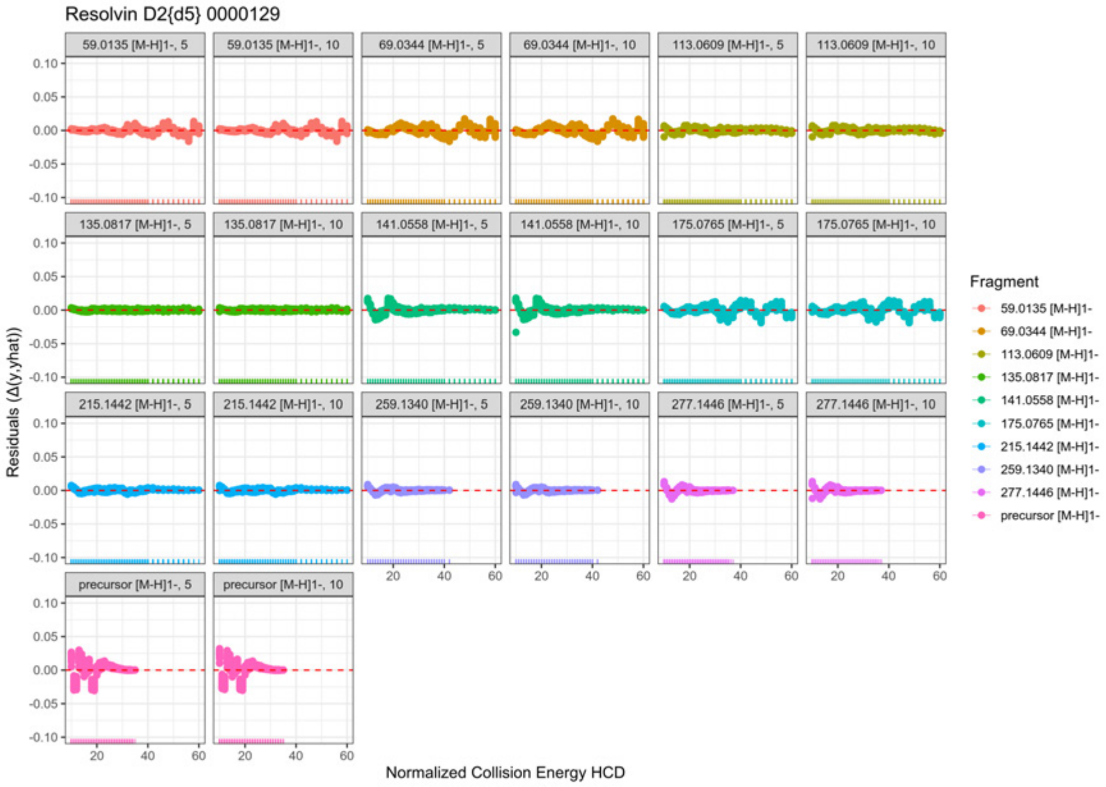

Figure 252. Residuals of nonlinear fit

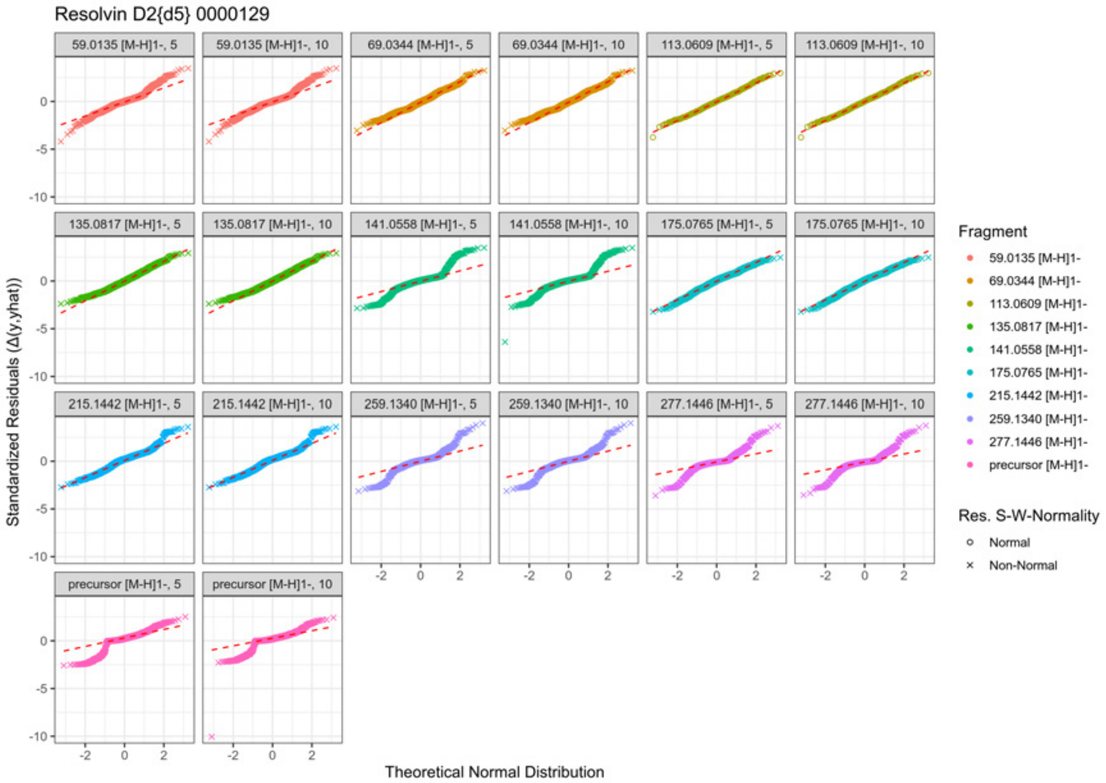

Figure 253. Quantile-quantile plot of residuals

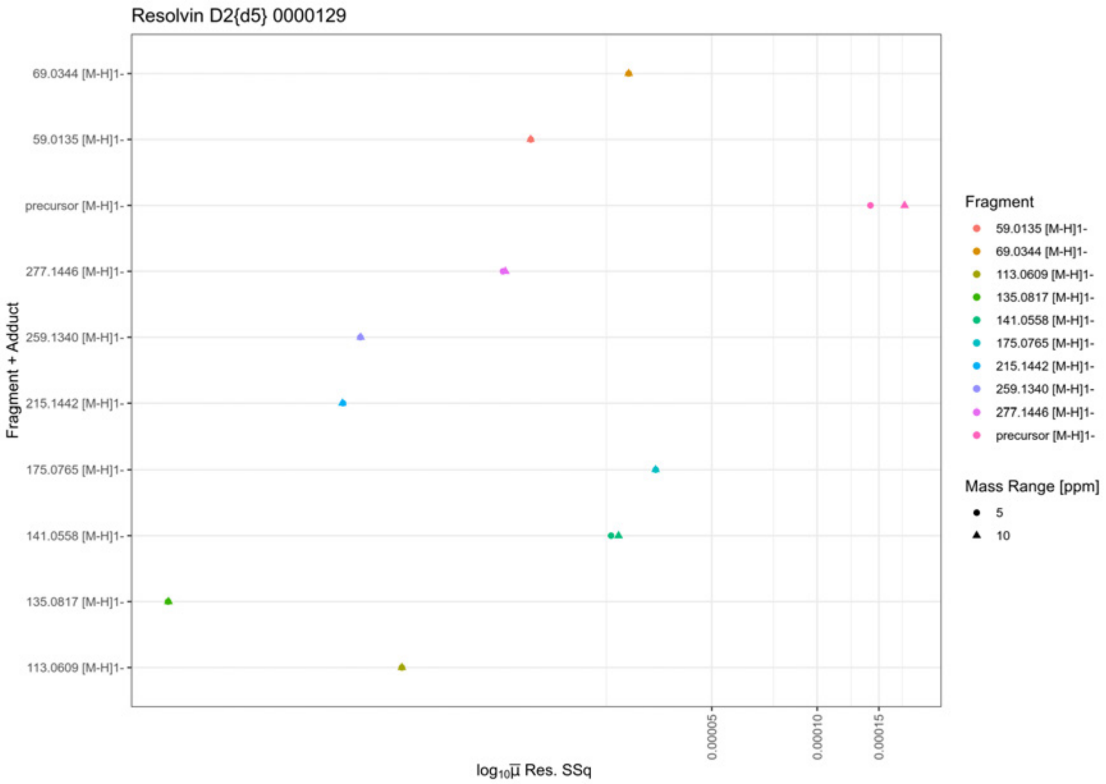

Figure 254. Normalized sum-of-squares of the residuals

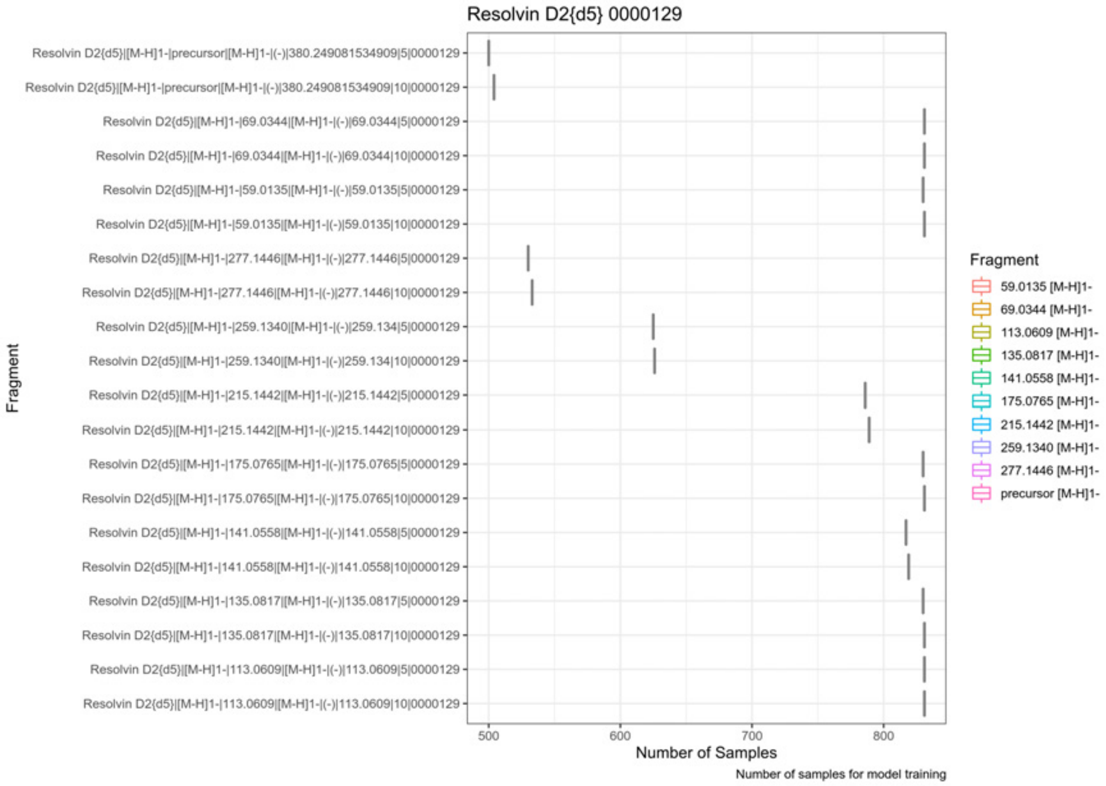

Figure 255. Number of samples used for training per combination Id

1.52. Resolvin D3 [M-H]1- 0000141

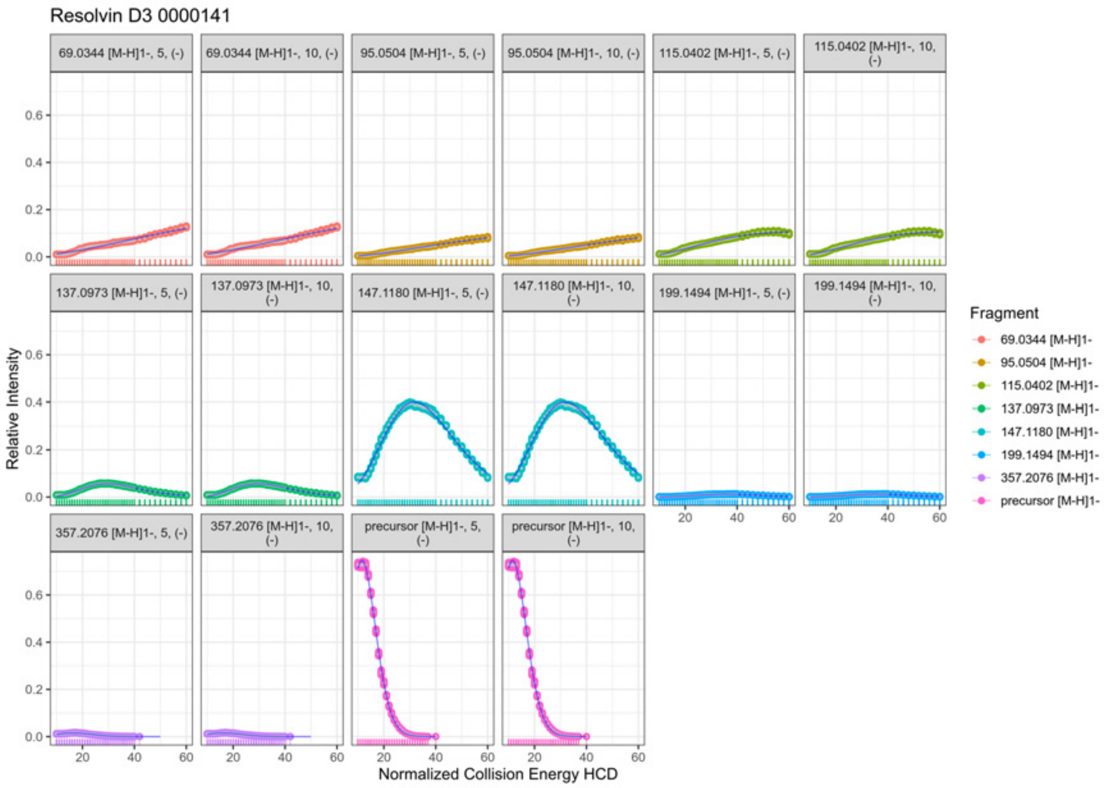

Figure 256. Nonlinear fit

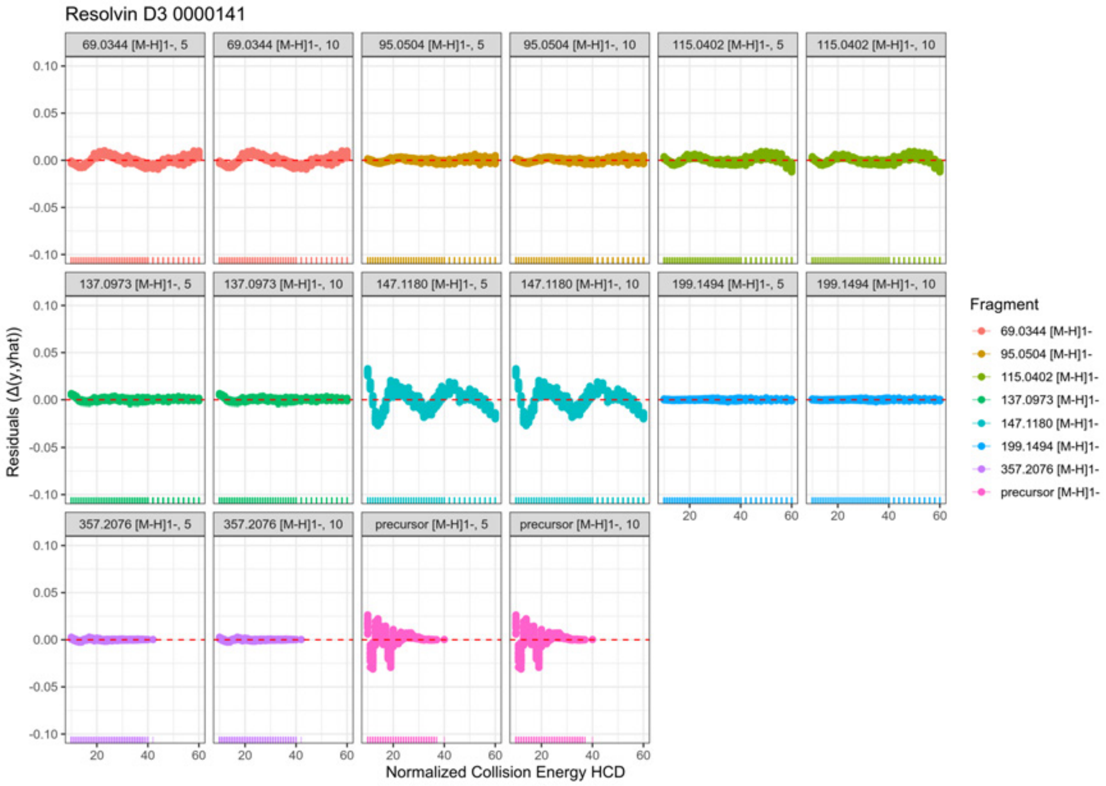

Figure 257. Residuals of nonlinear fit

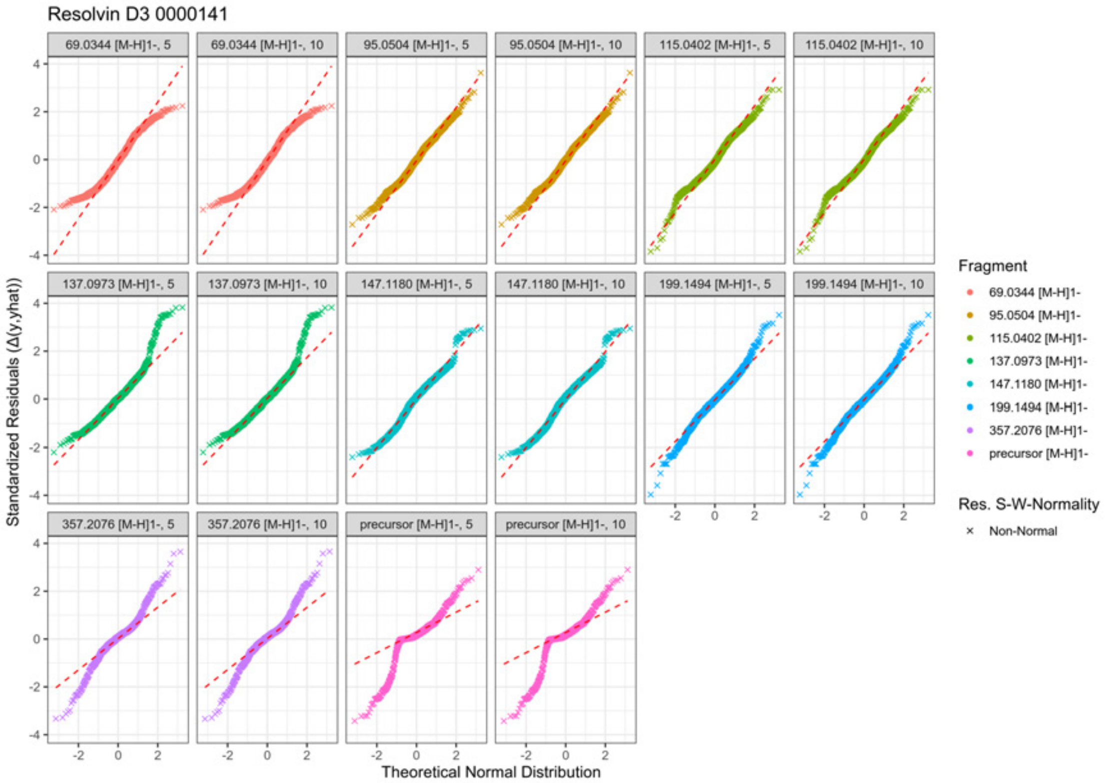

Figure 258. Quantile-quantile plot of residuals

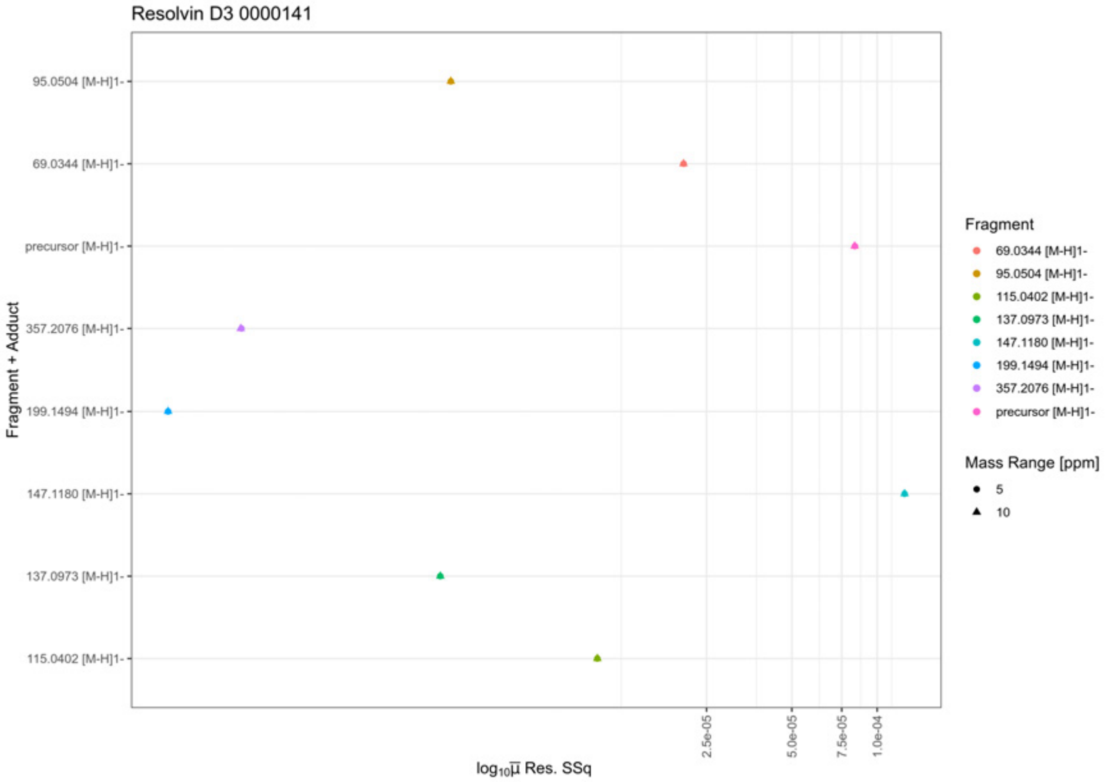

Figure 259. Normalized sum-of-squares of the residuals

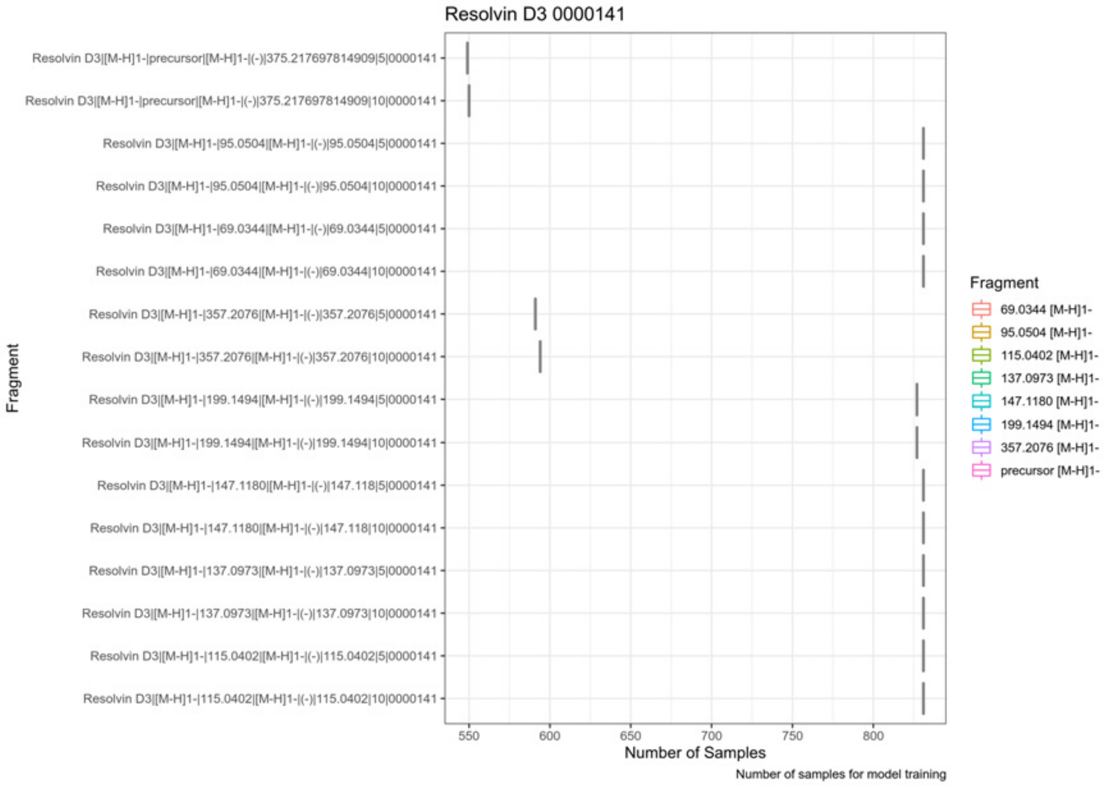

Figure 260. Number of samples used for training per combination Id

1.53. Resolvin D5 [M-H]1- 0000143

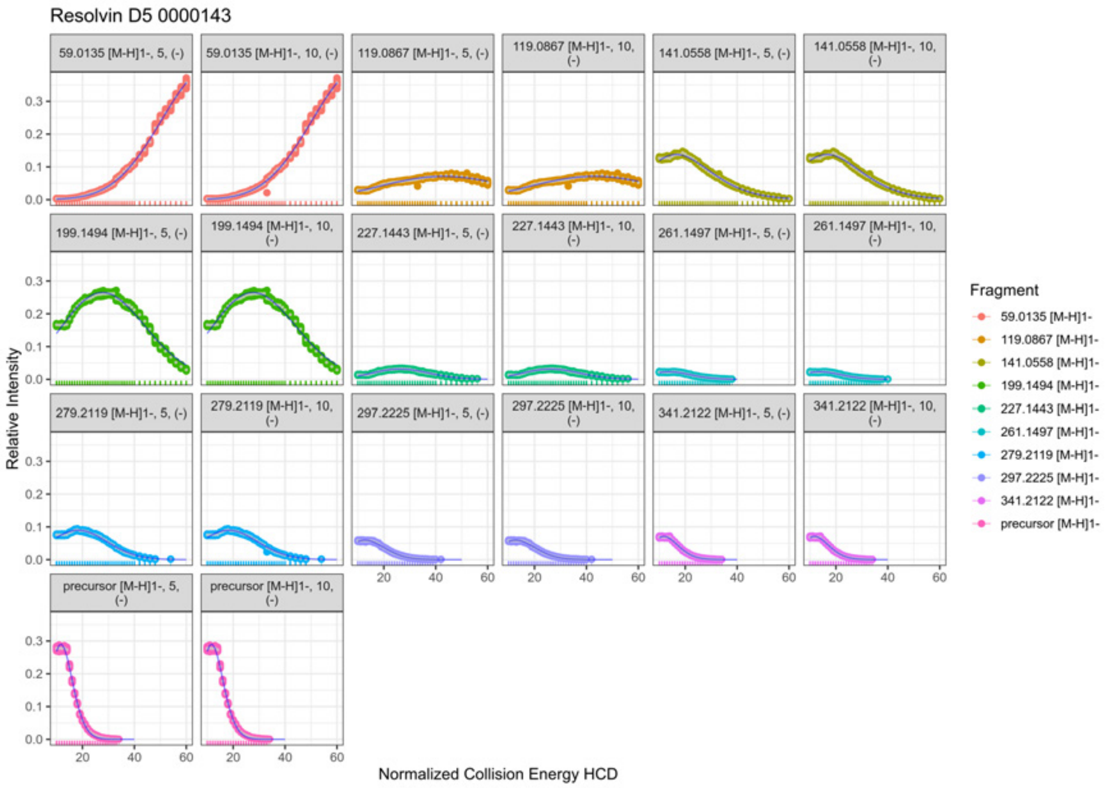

Figure 261. Nonlinear fit

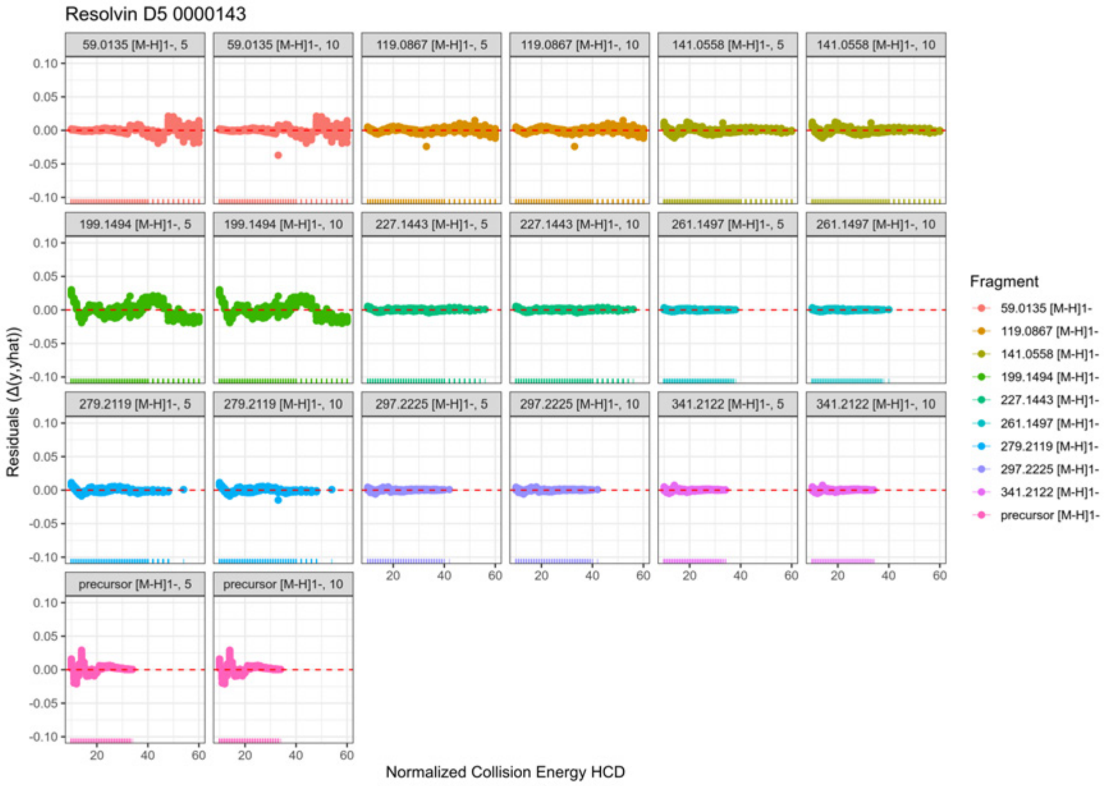

Figure 262. Residuals of nonlinear fit

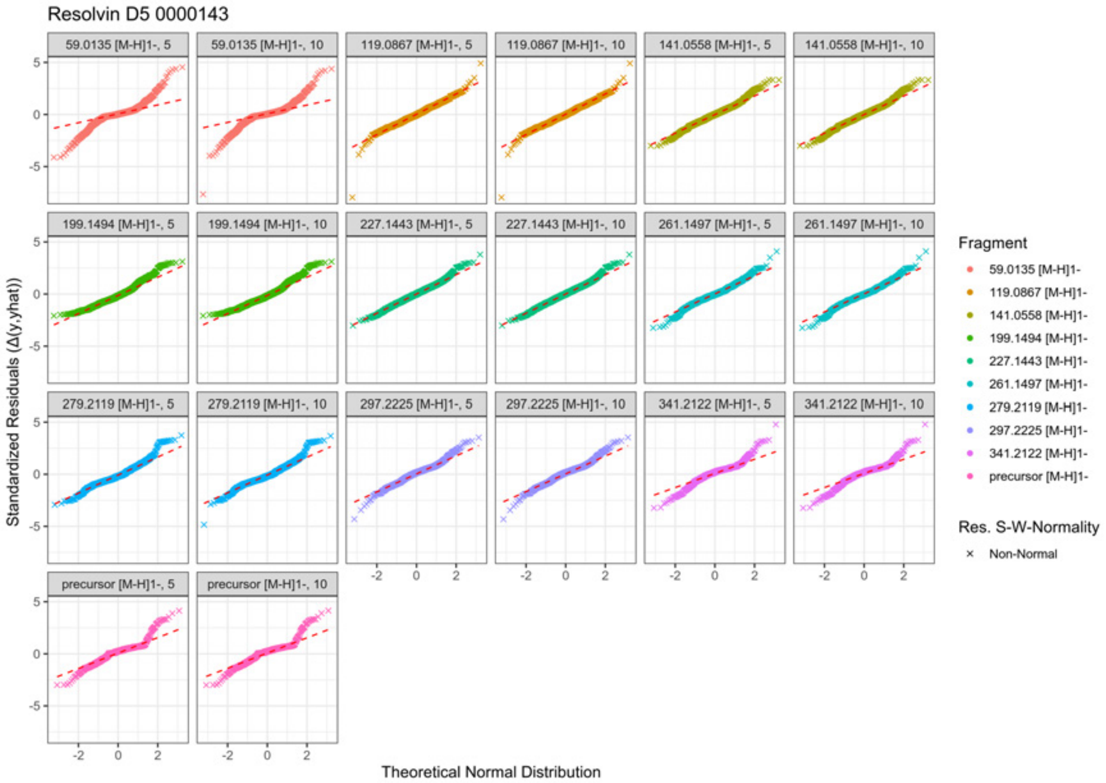

Figure 263. Quantile-quantile plot of residuals

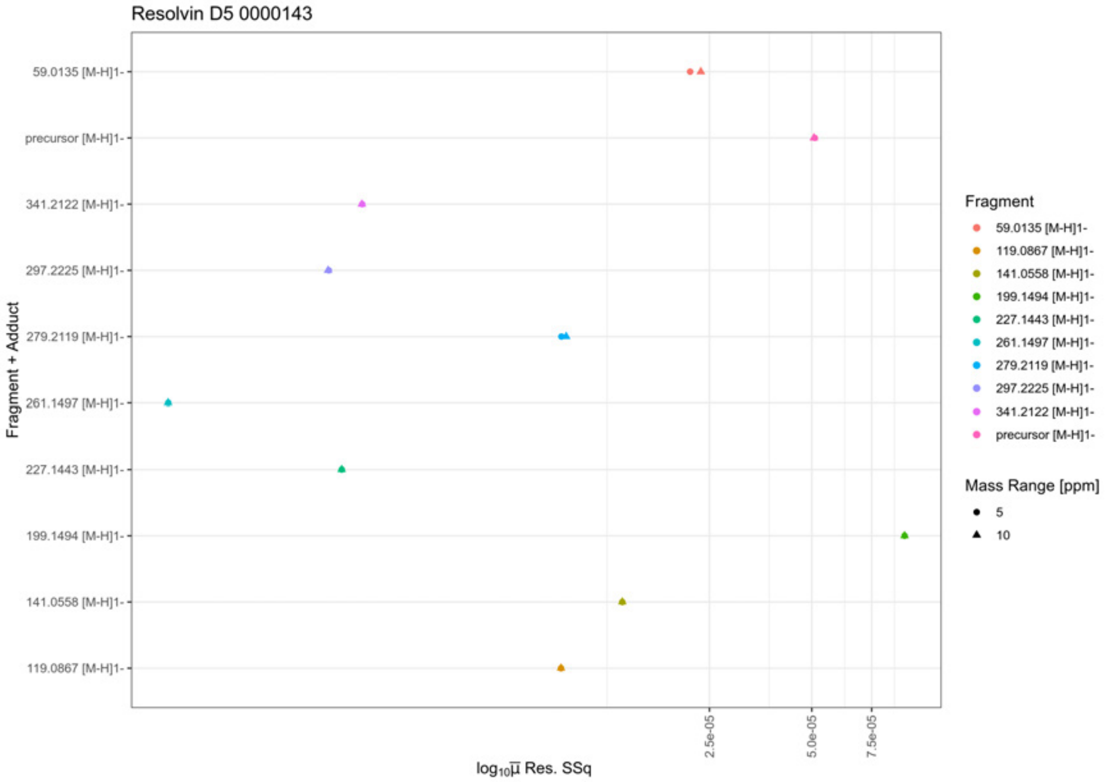

Figure 264. Normalized sum-of-squares of the residuals

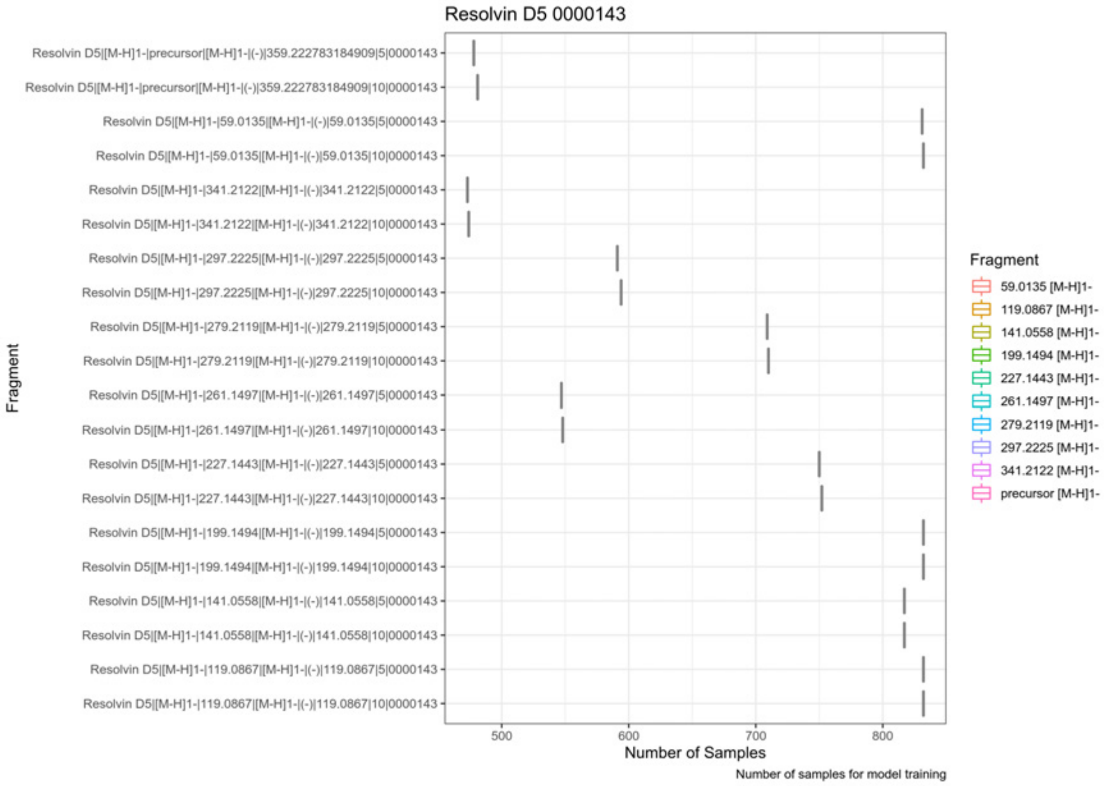

Figure 265. Number of samples used for training per combination Id

1.54. TXB1 [M-H]1- 0001325

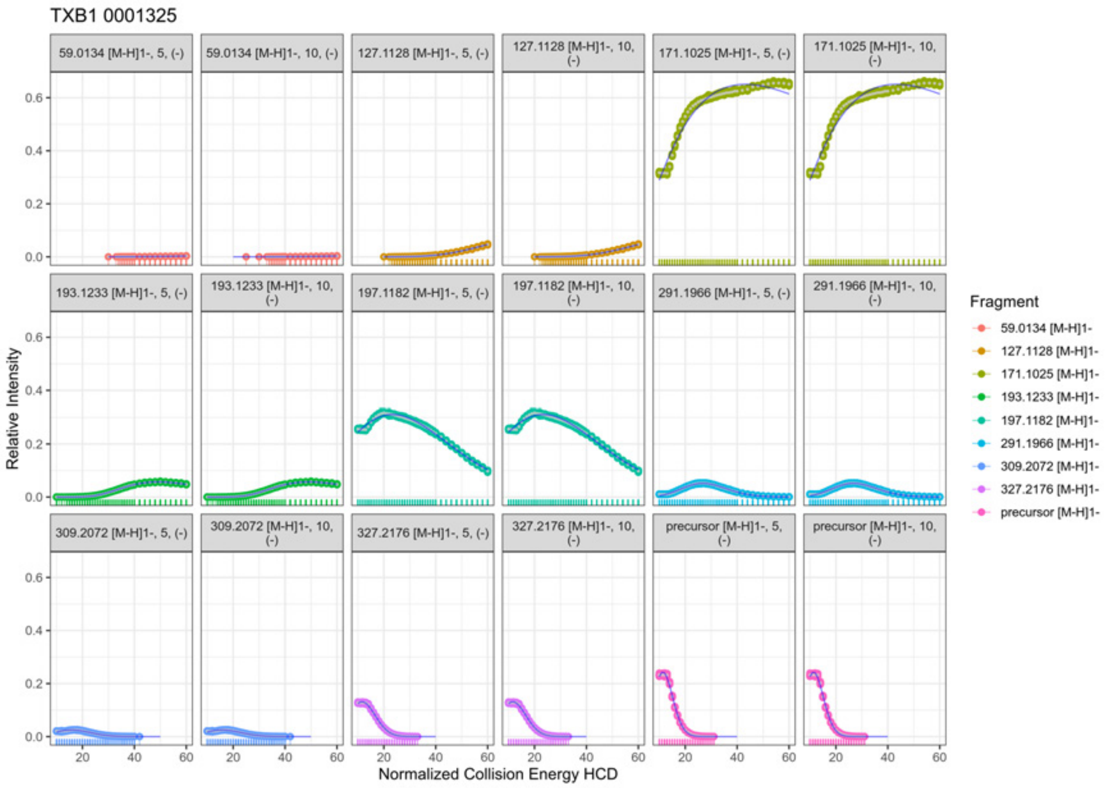

Figure 266. Nonlinear fit

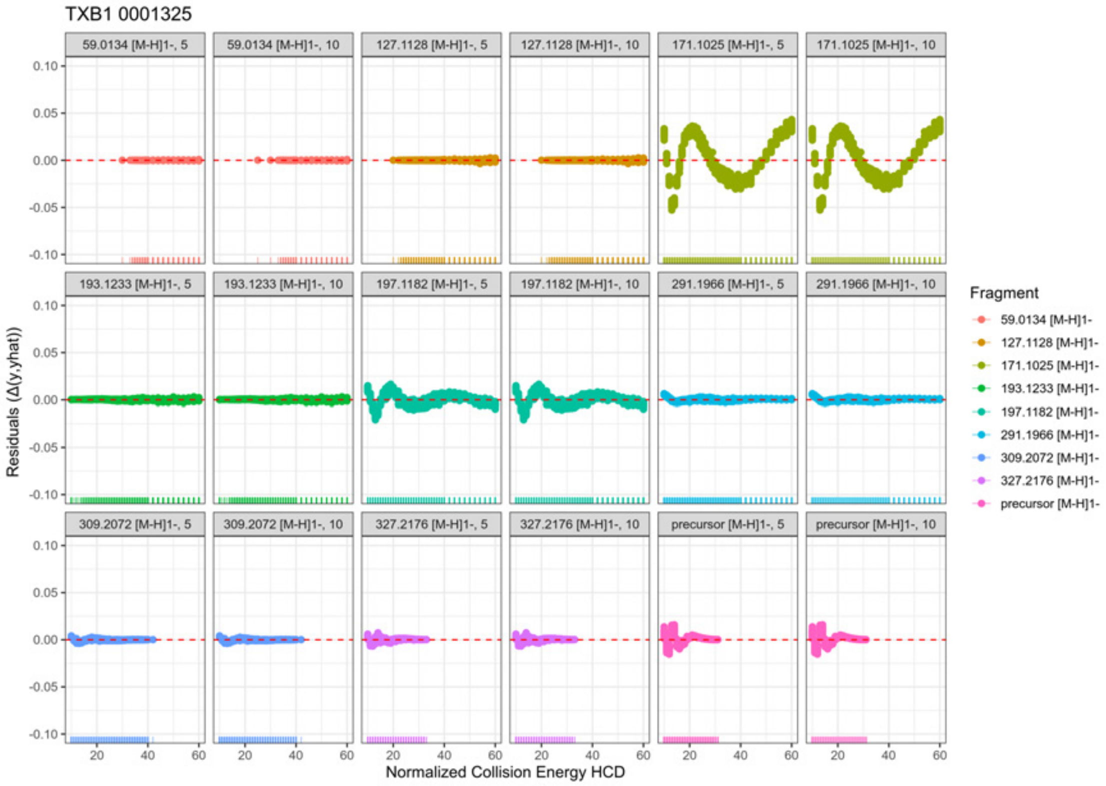

Figure 267. Residuals of nonlinear fit

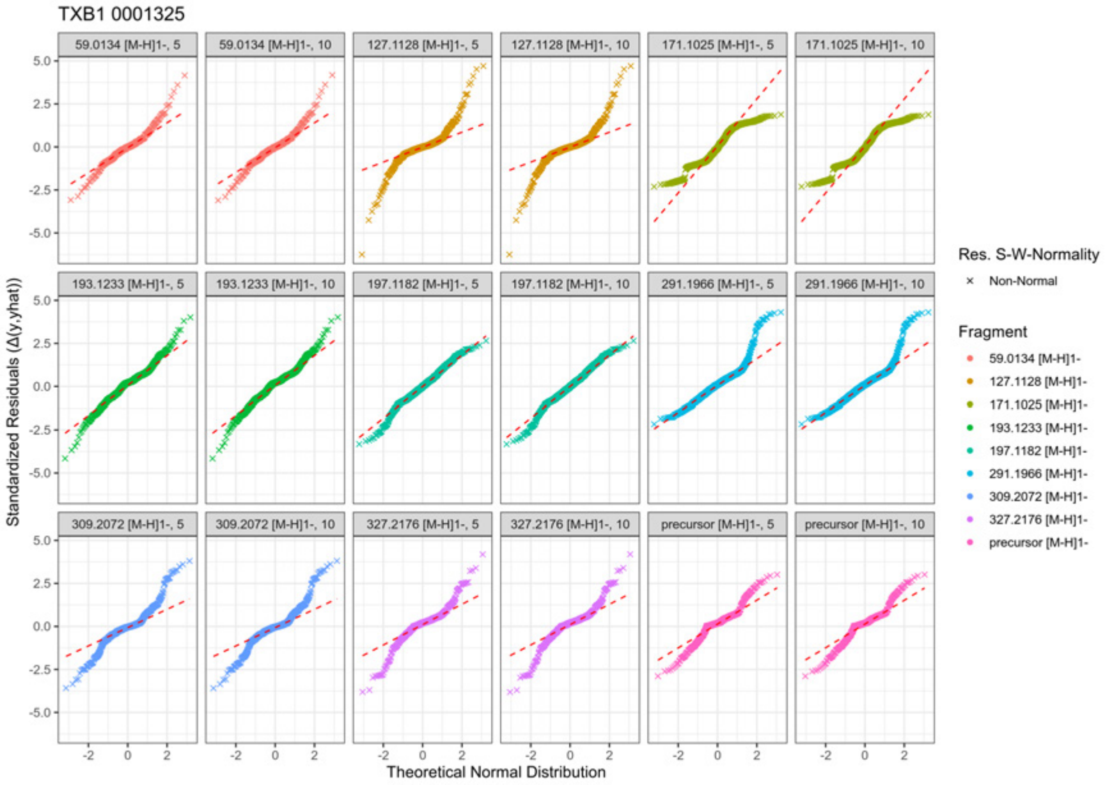

Figure 268. Quantile-quantile plot of residuals

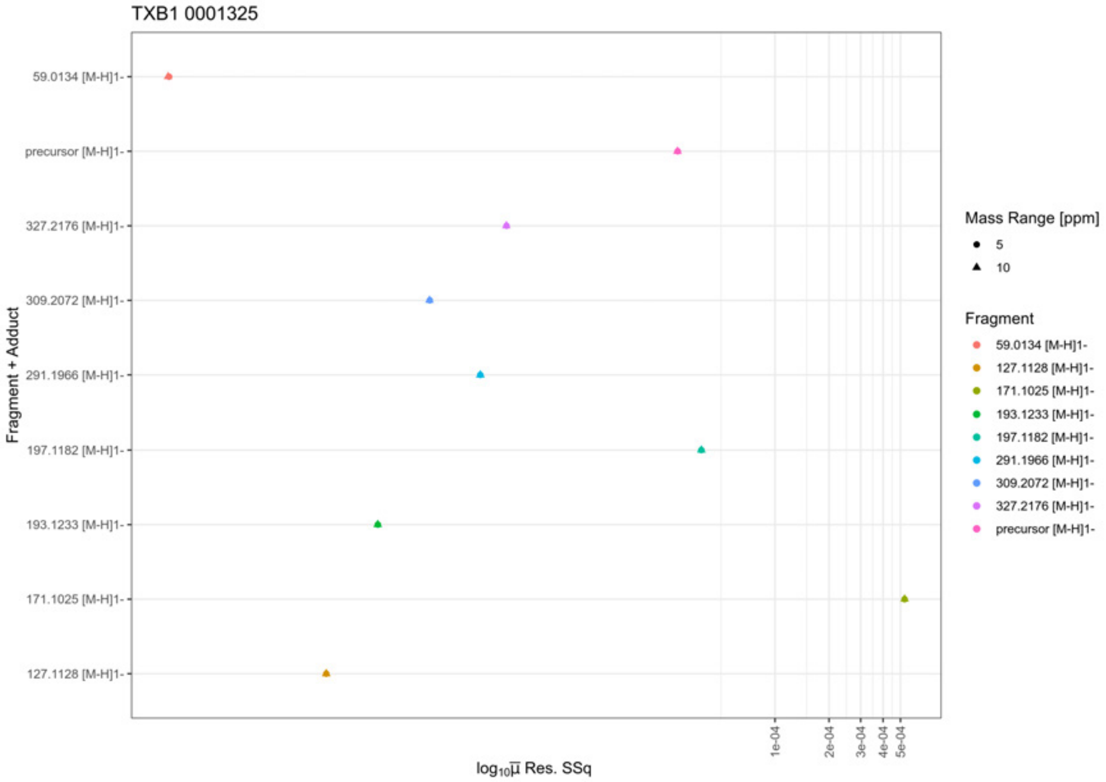

Figure 269. Normalized sum-of-squares of the residuals

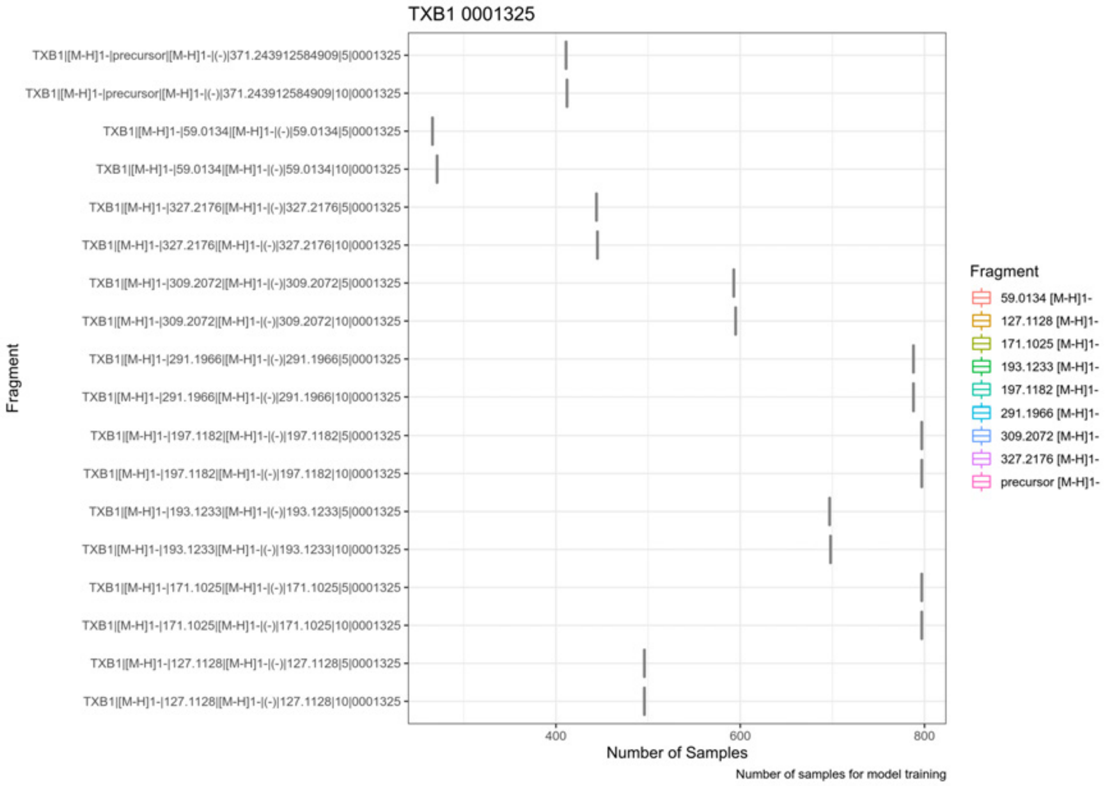

Figure 270. Number of samples used for training per combination Id

1.55. TXB2{d4} [M-H]1- 0001327

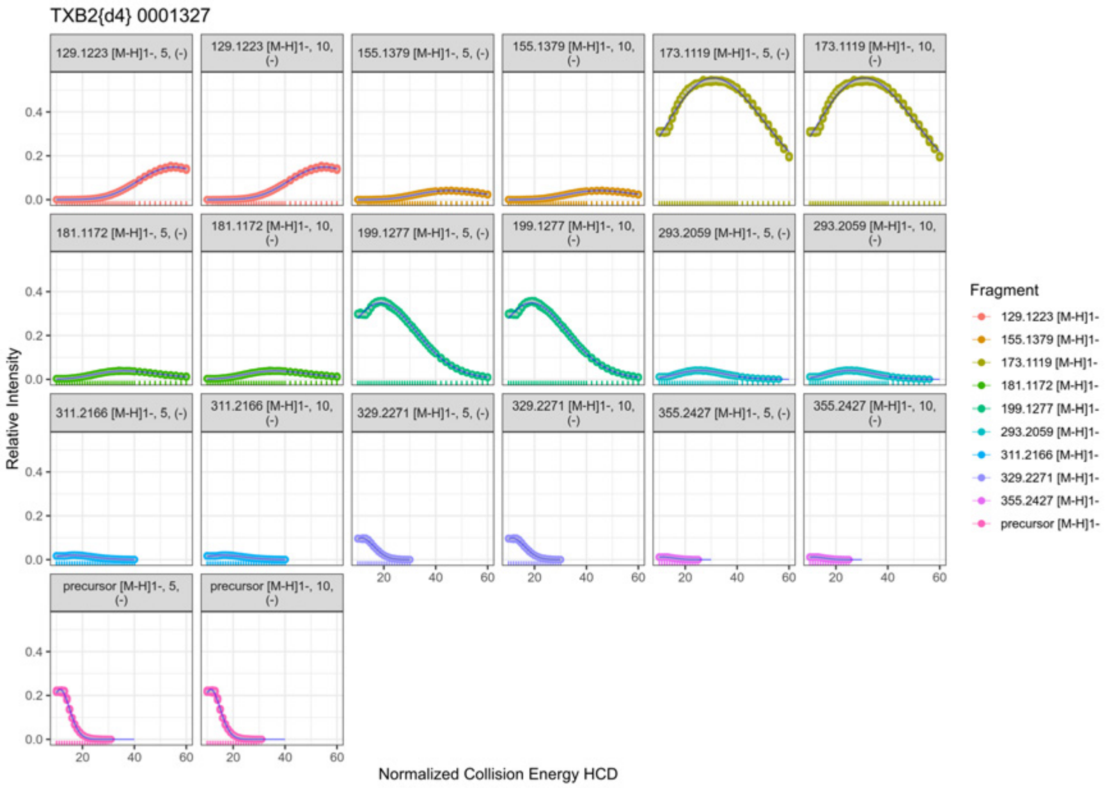

Figure 271. Nonlinear fit

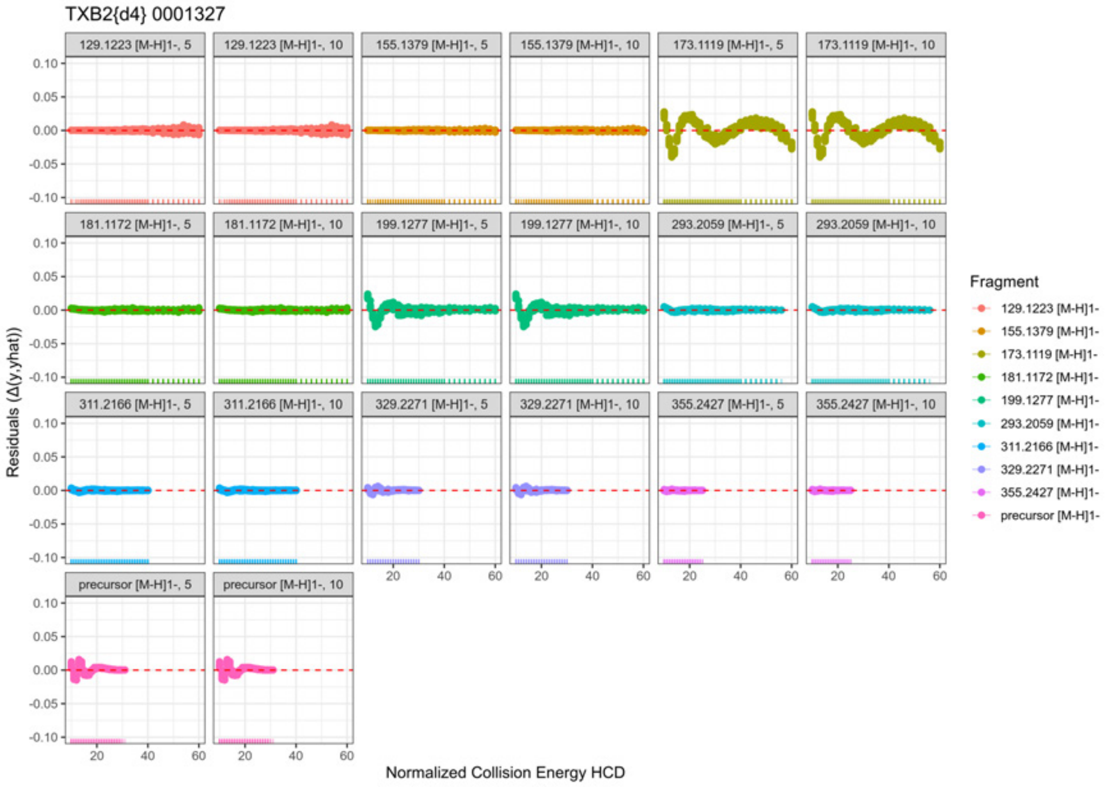

Figure 272. Residuals of nonlinear fit

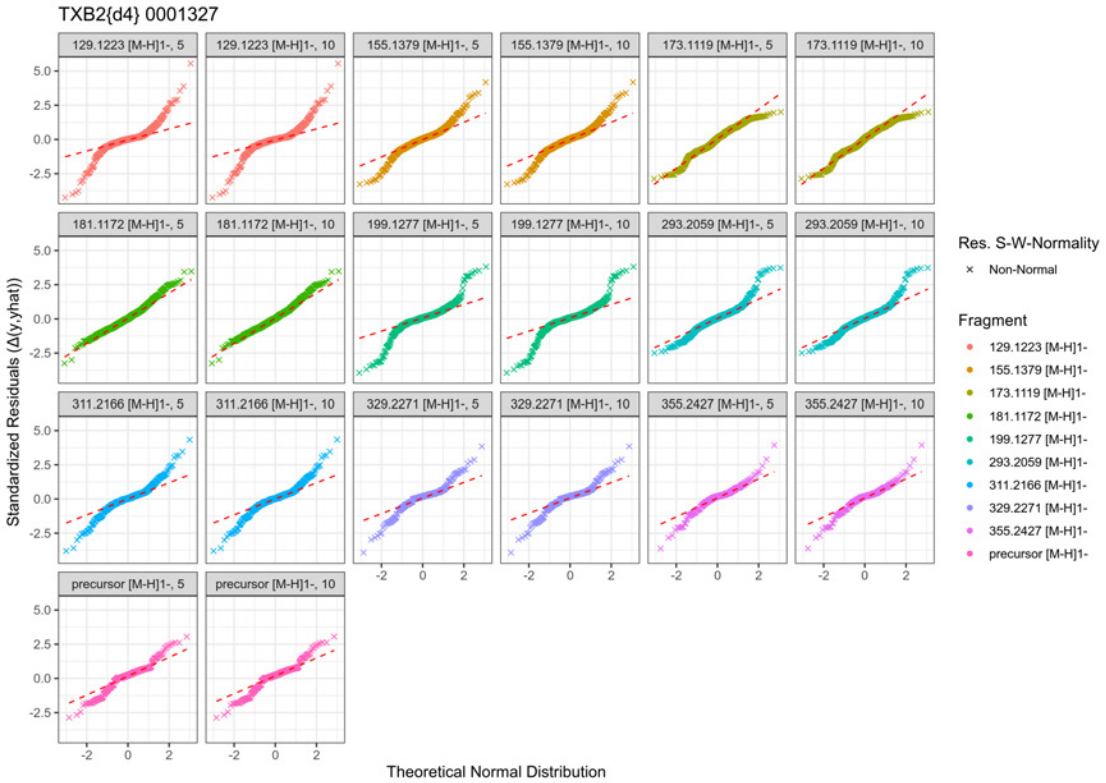

Figure 273. Quantile-quantile plot of residuals

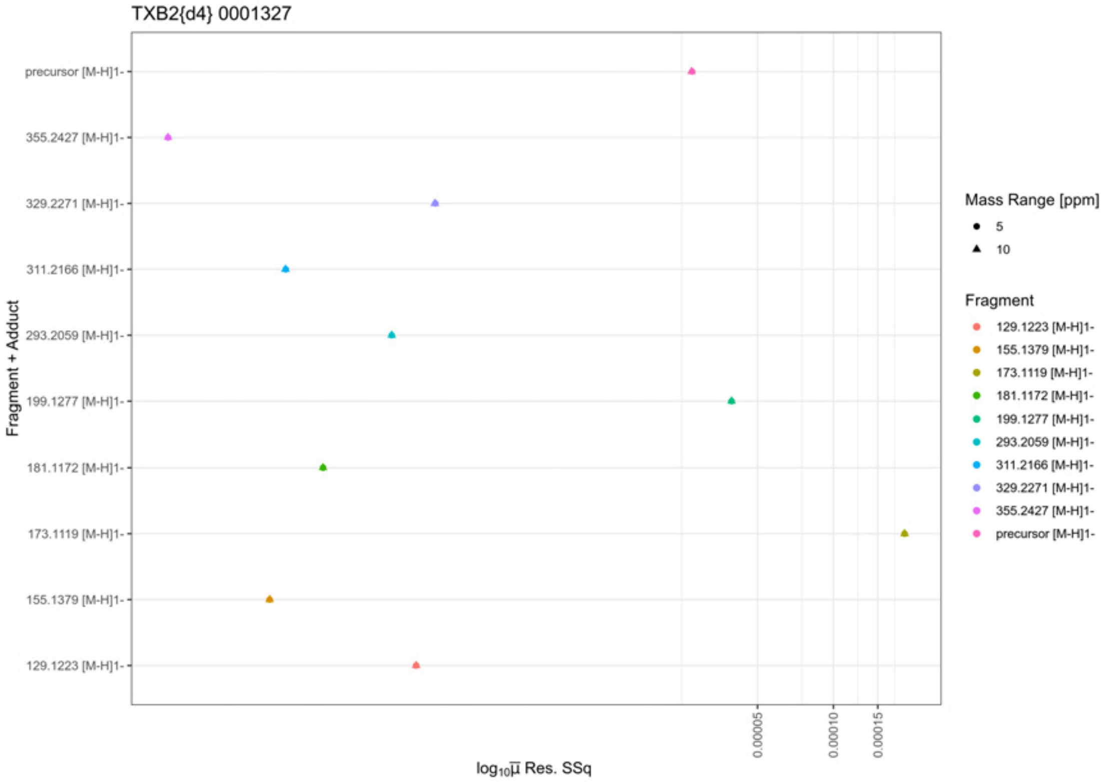

Figure 274. Normalized sum-of-squares of the residuals

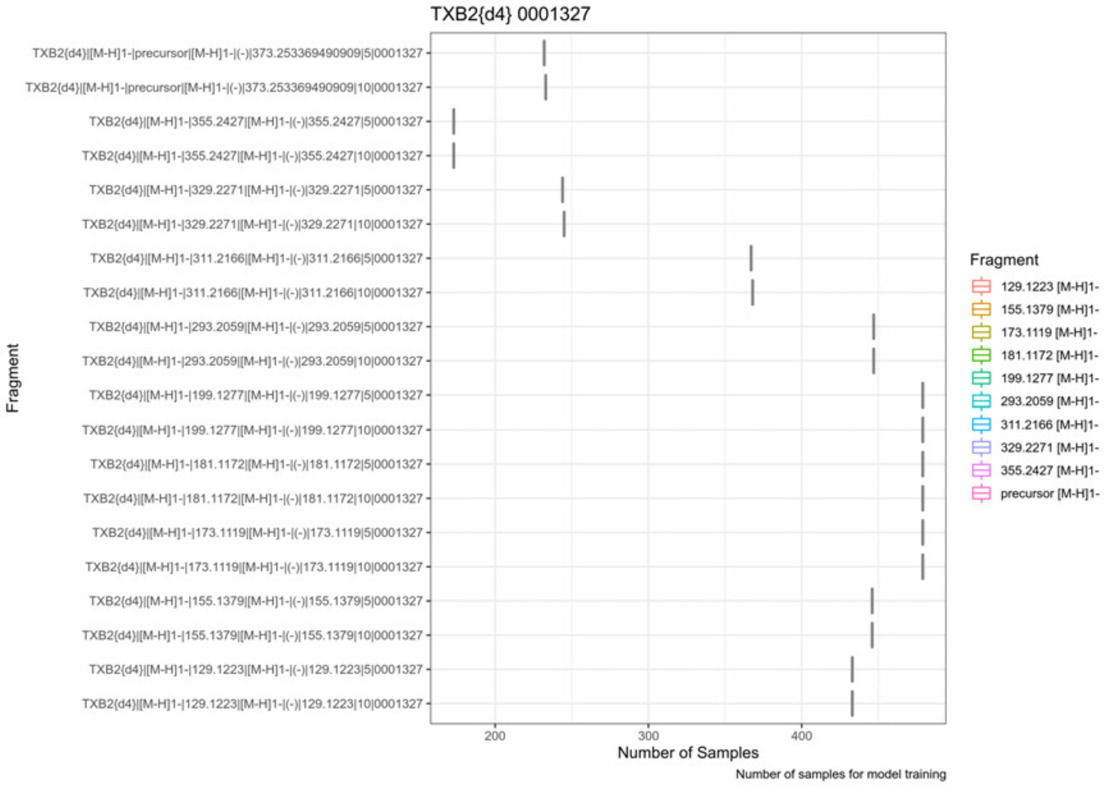

Figure 275. Number of samples used for training per combination Id

1.56. TXB3 [M-H]1- 0001323

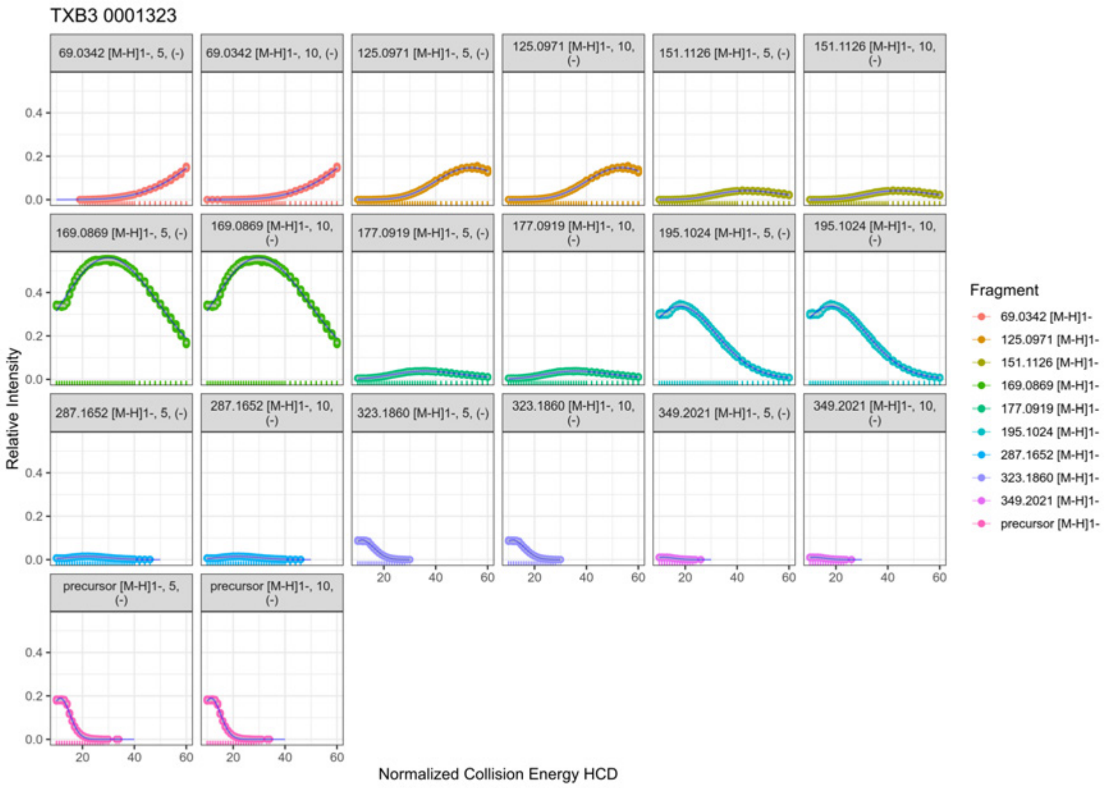

Figure 276. Nonlinear fit

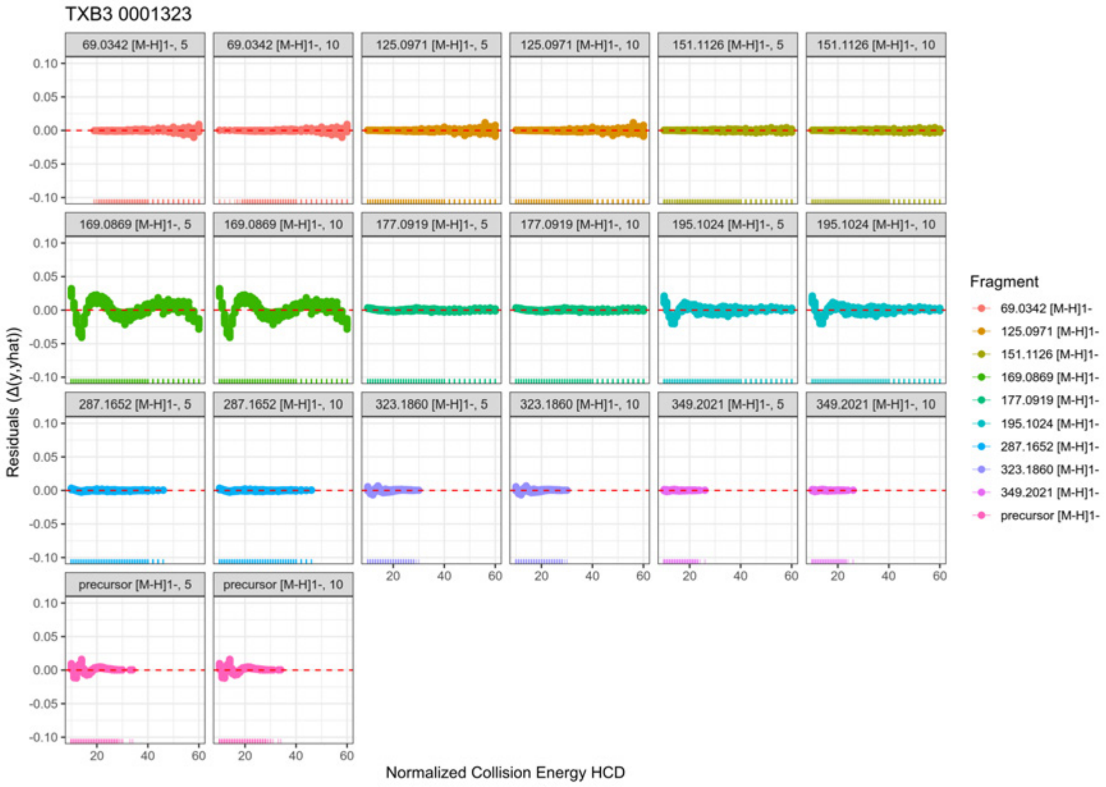

Figure 277. Residuals of nonlinear fit

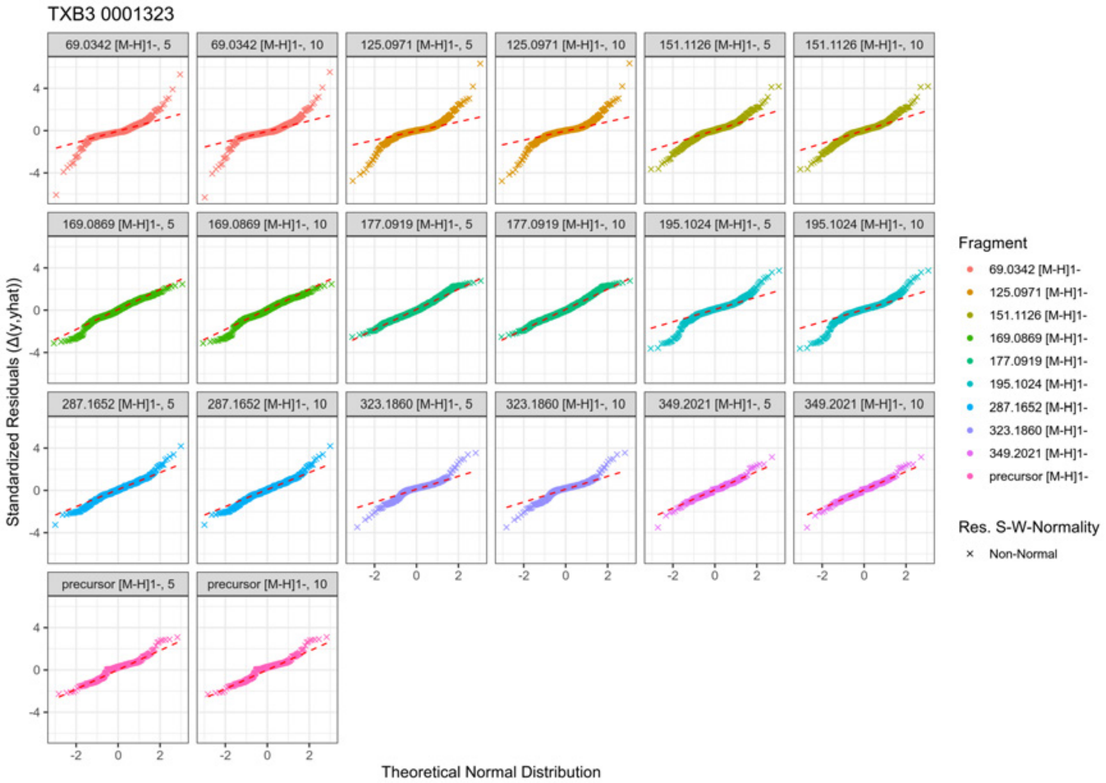

Figure 278. Quantile-quantile plot of residuals

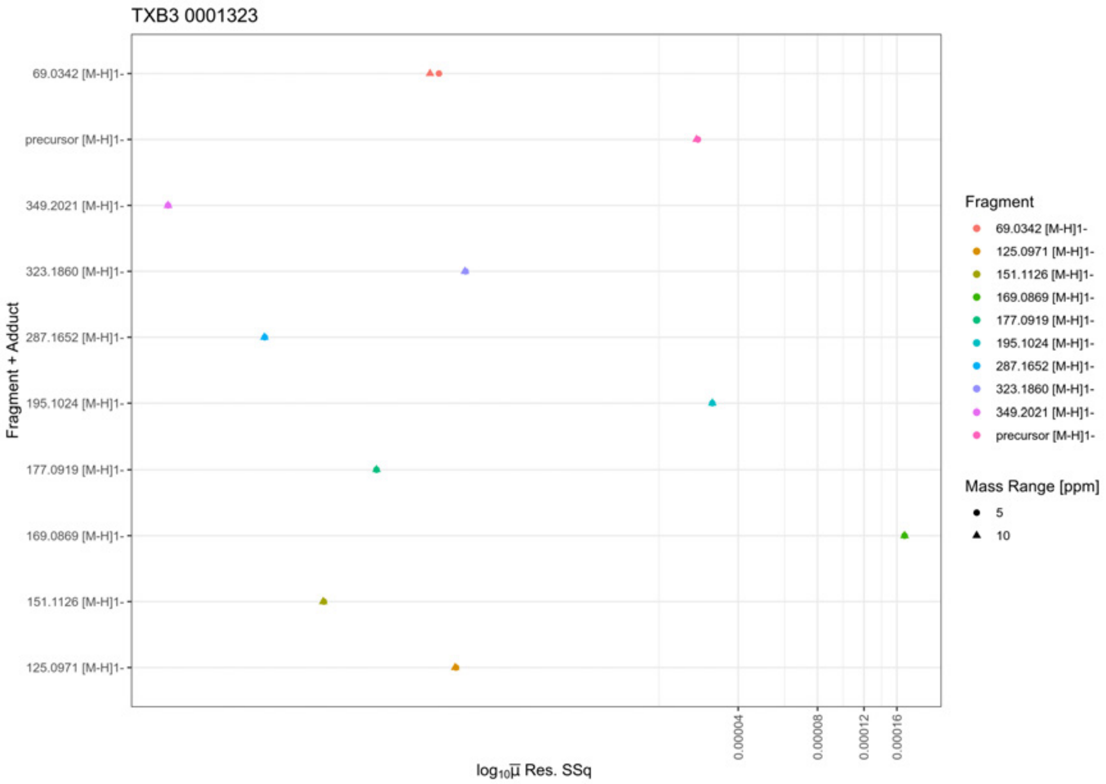

Figure 279. Normalized sum-of-squares of the residuals

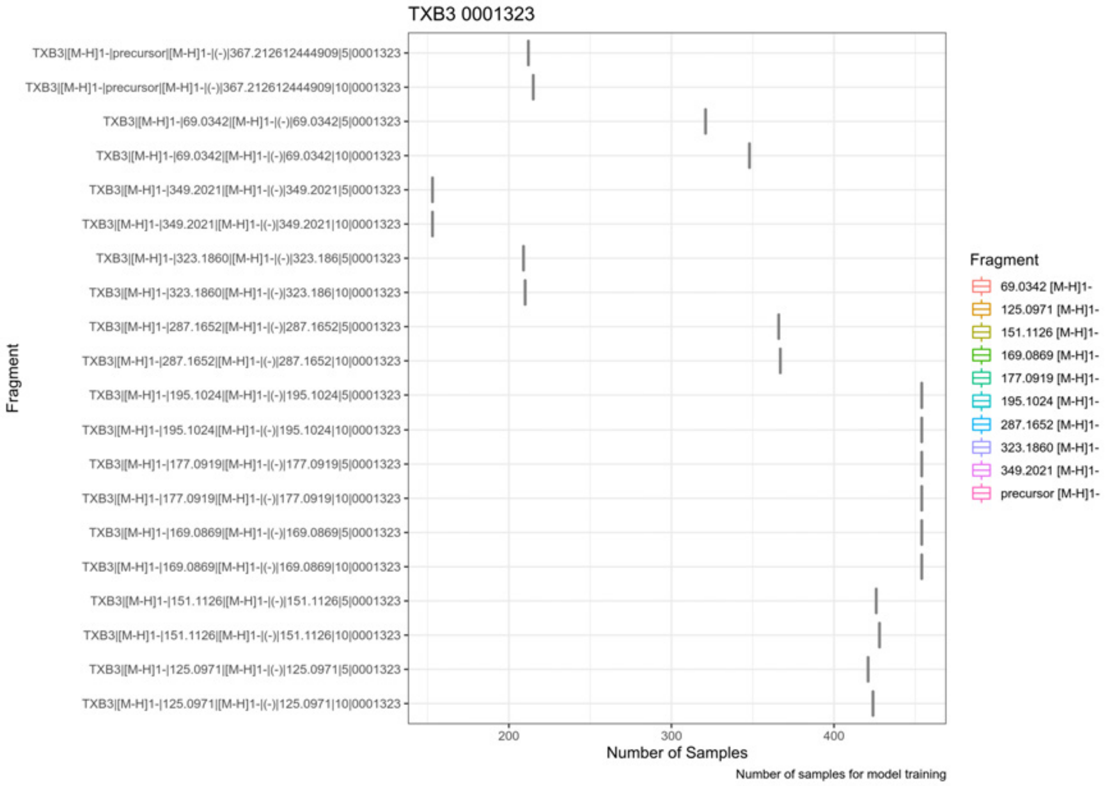

Figure 280. Number of samples used for training per combination Id

1.57. alpha-LA{d14} [M-H]1- 0000135

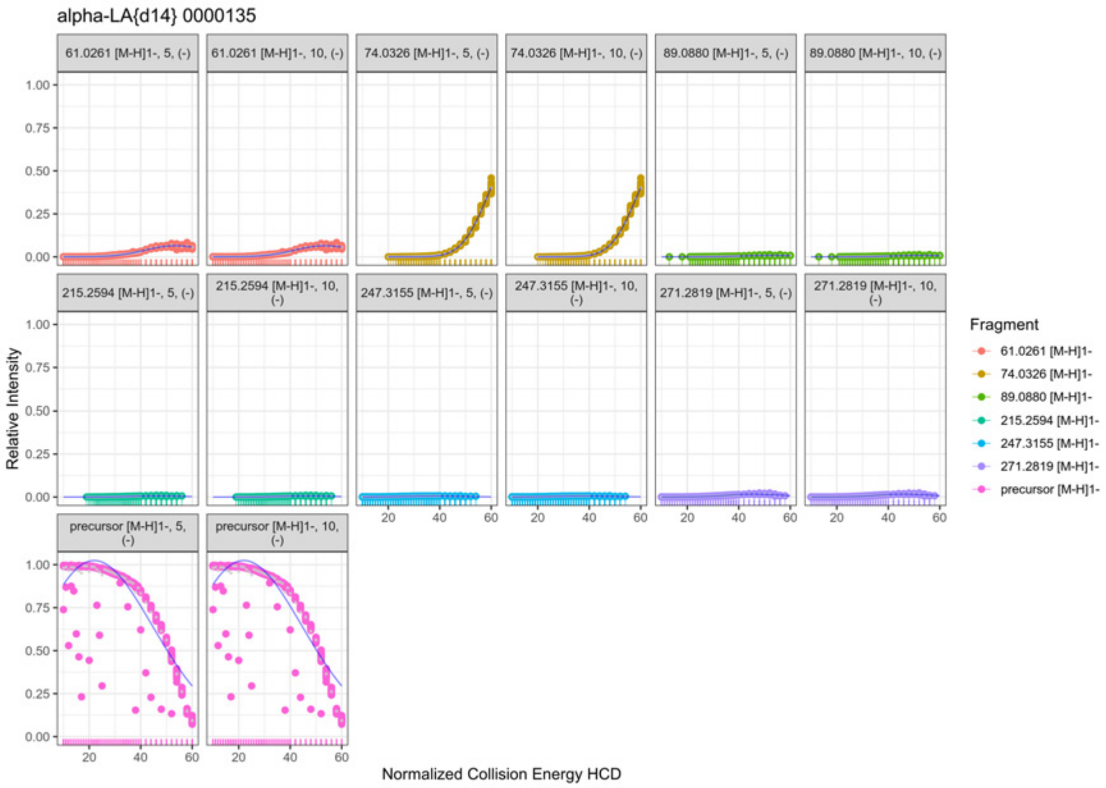

Figure 281. Nonlinear fit

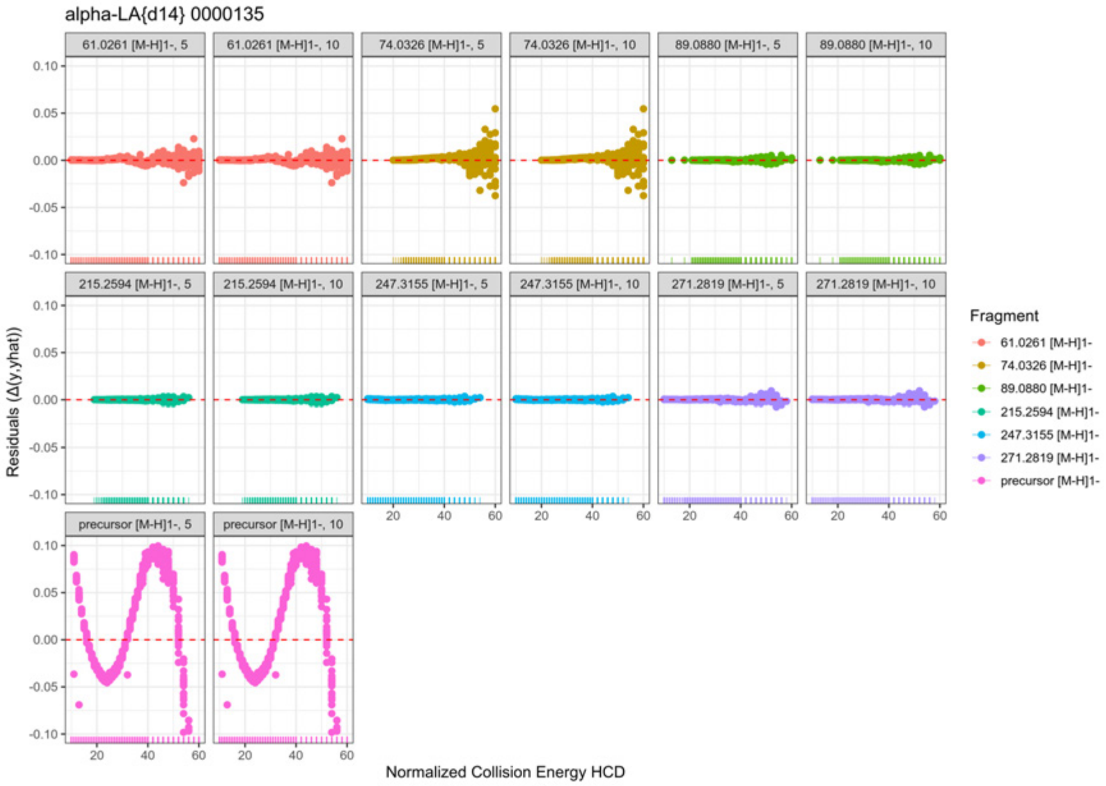

Figure 282. Residuals of nonlinear fit

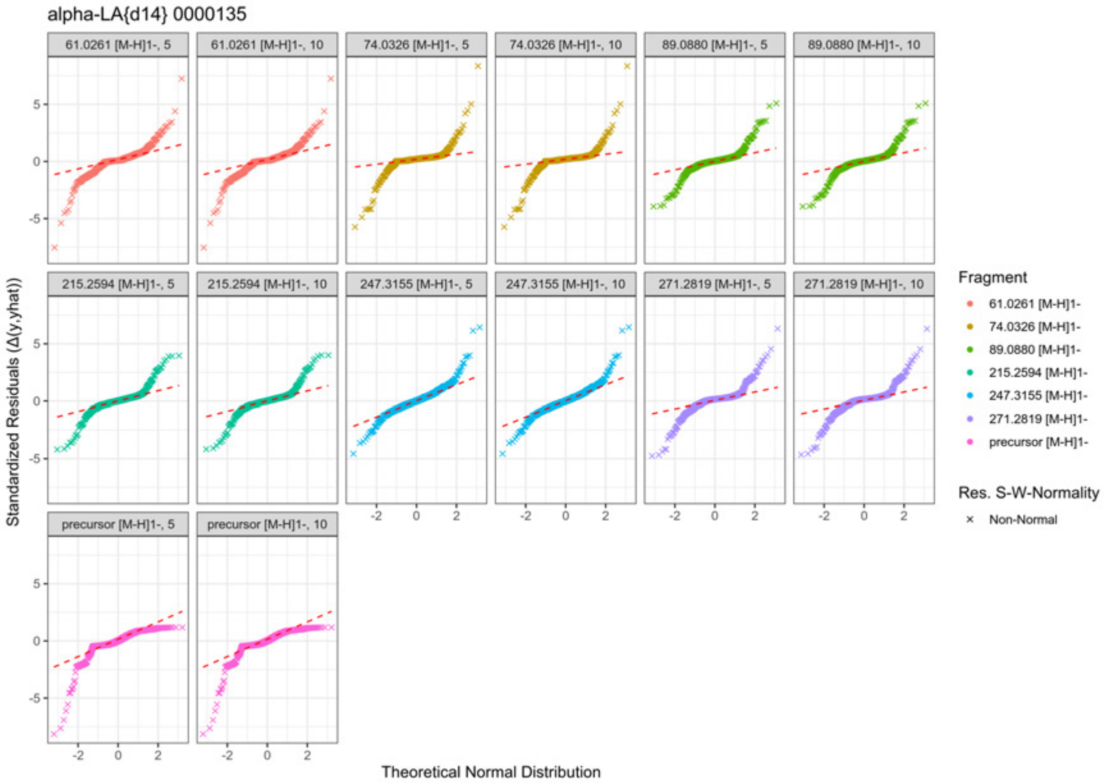

Figure 283. Quantile-quantile plot of residuals

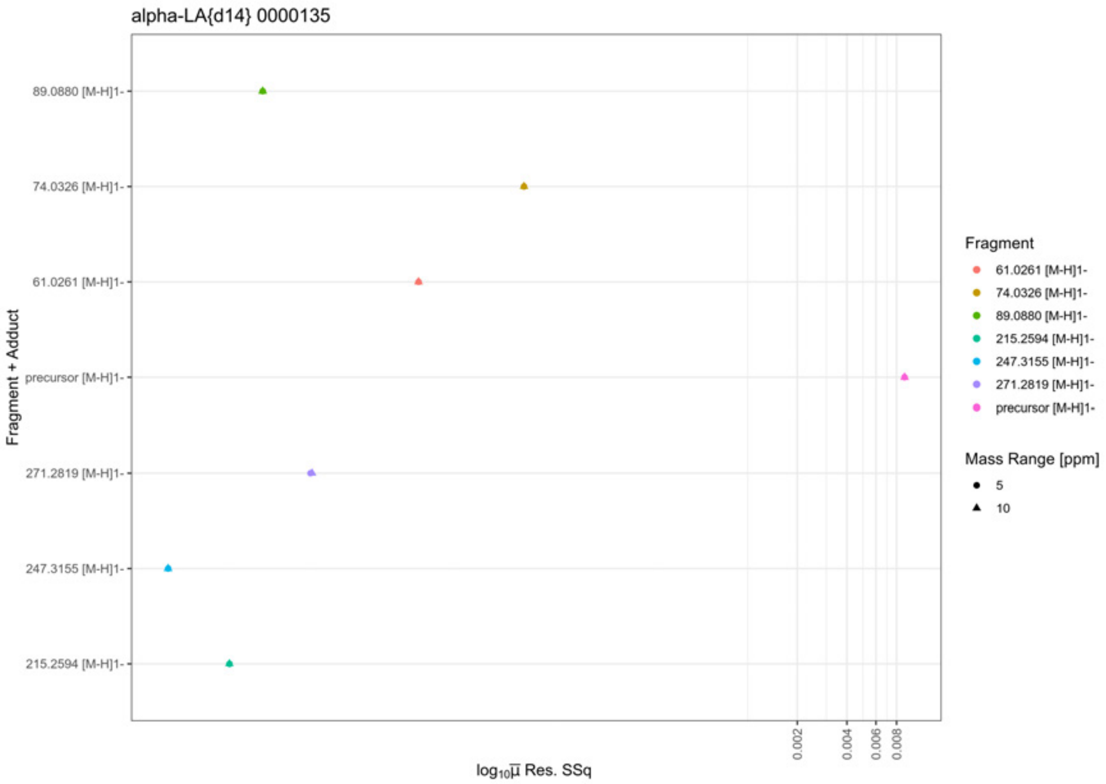

Figure 284. Normalized sum-of-squares of the residuals

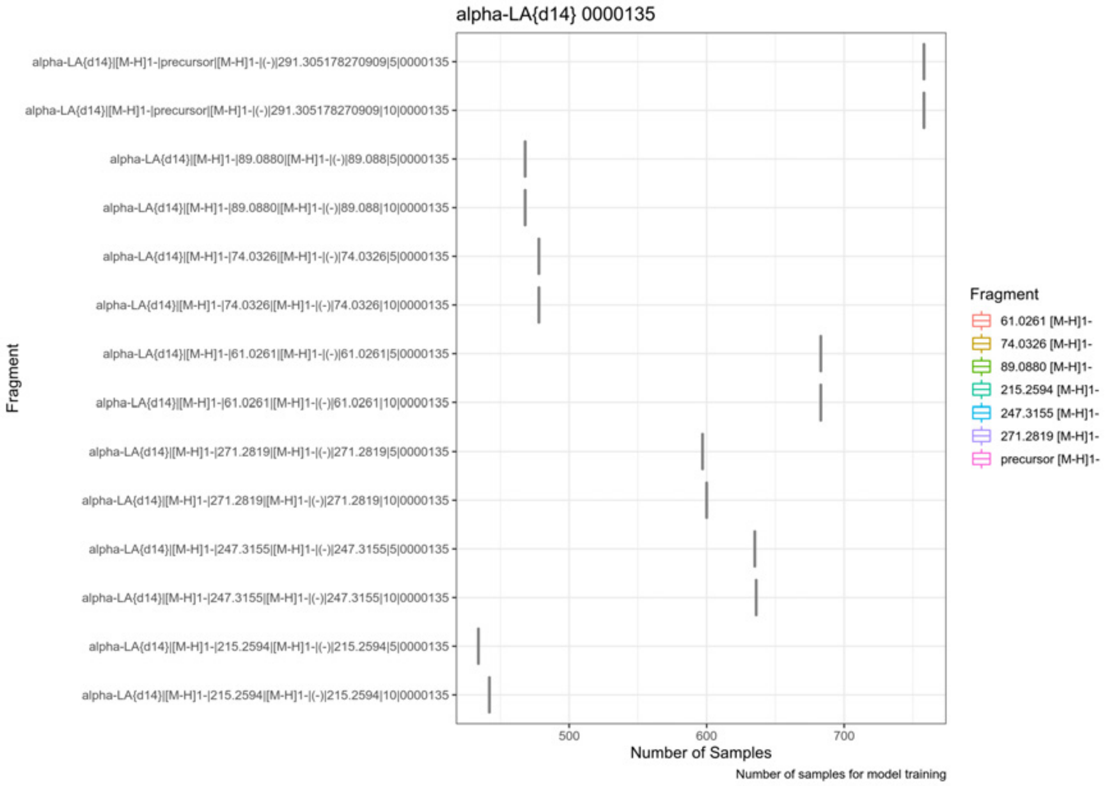

Figure 285. Number of samples used for training per combination Id

1.58. tetranor-12-HETE [M-H]1- 0001351

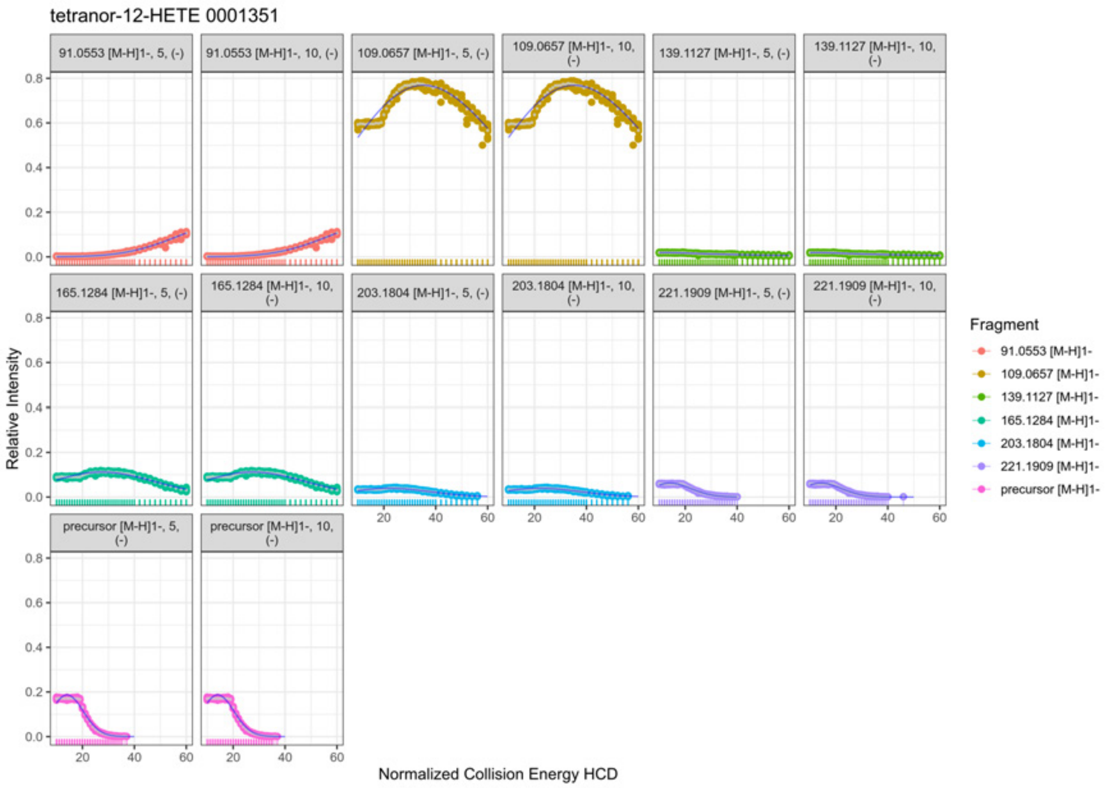

Figure 286. Nonlinear fit

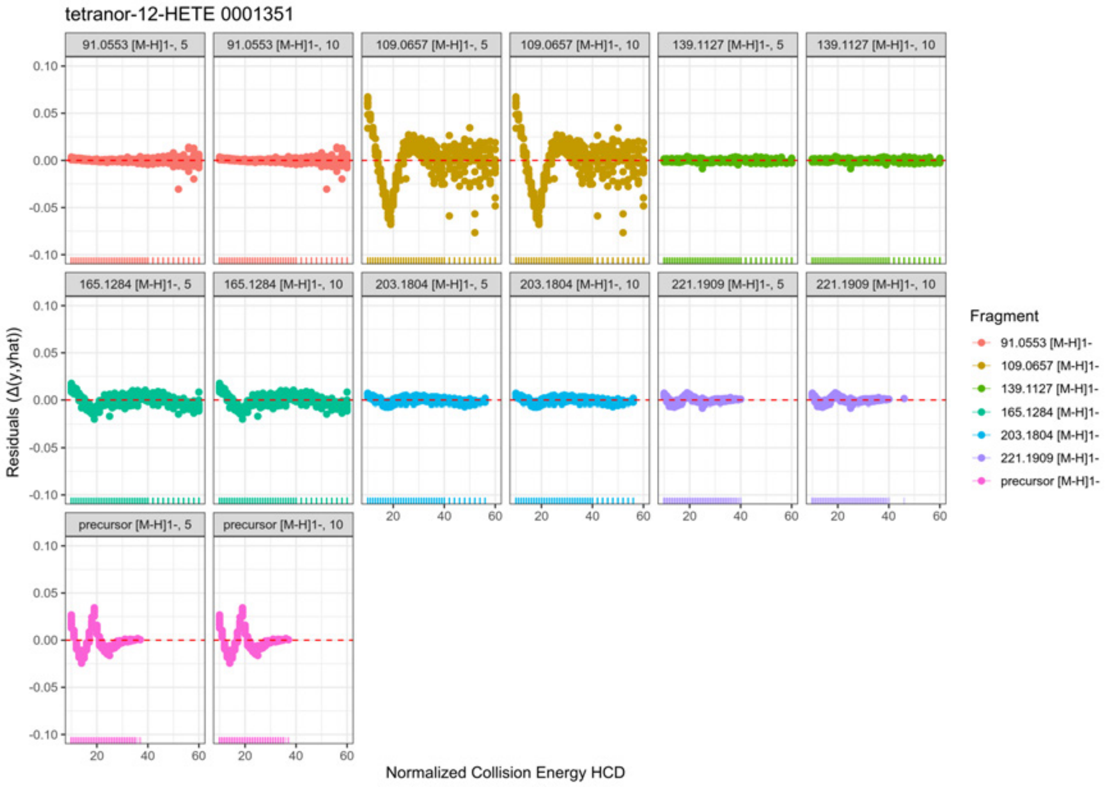

Figure 287. Residuals of nonlinear fit

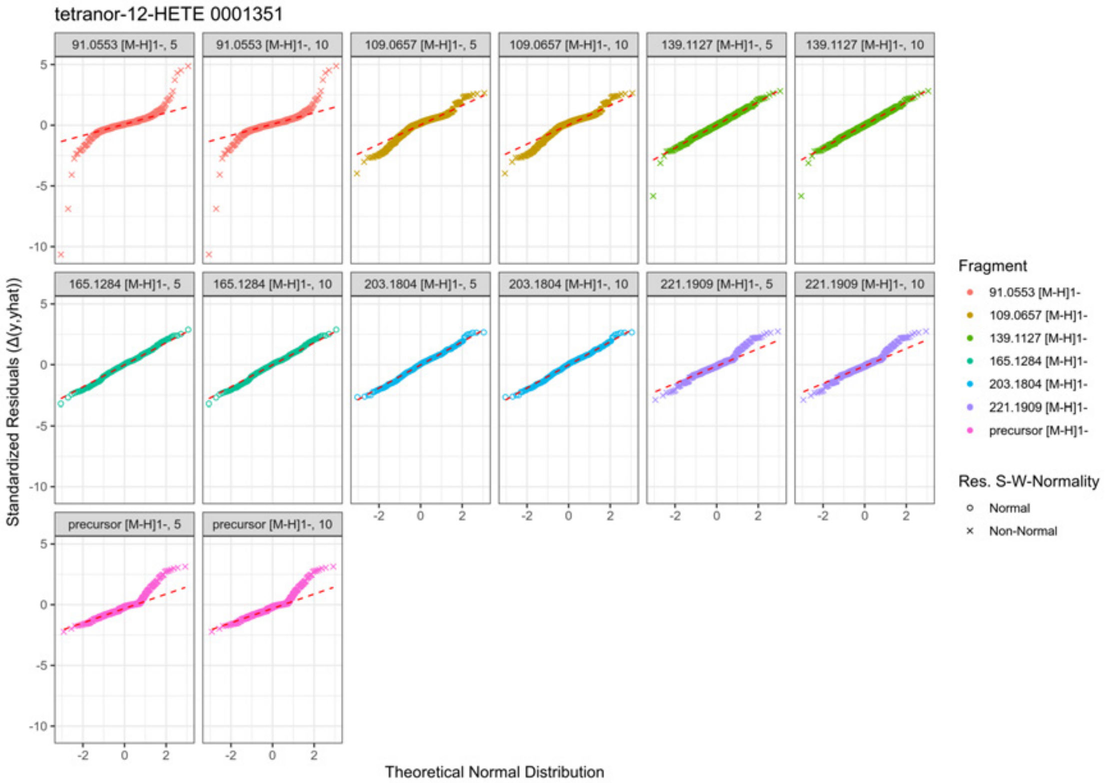

Figure 288. Quantile-quantile plot of residuals

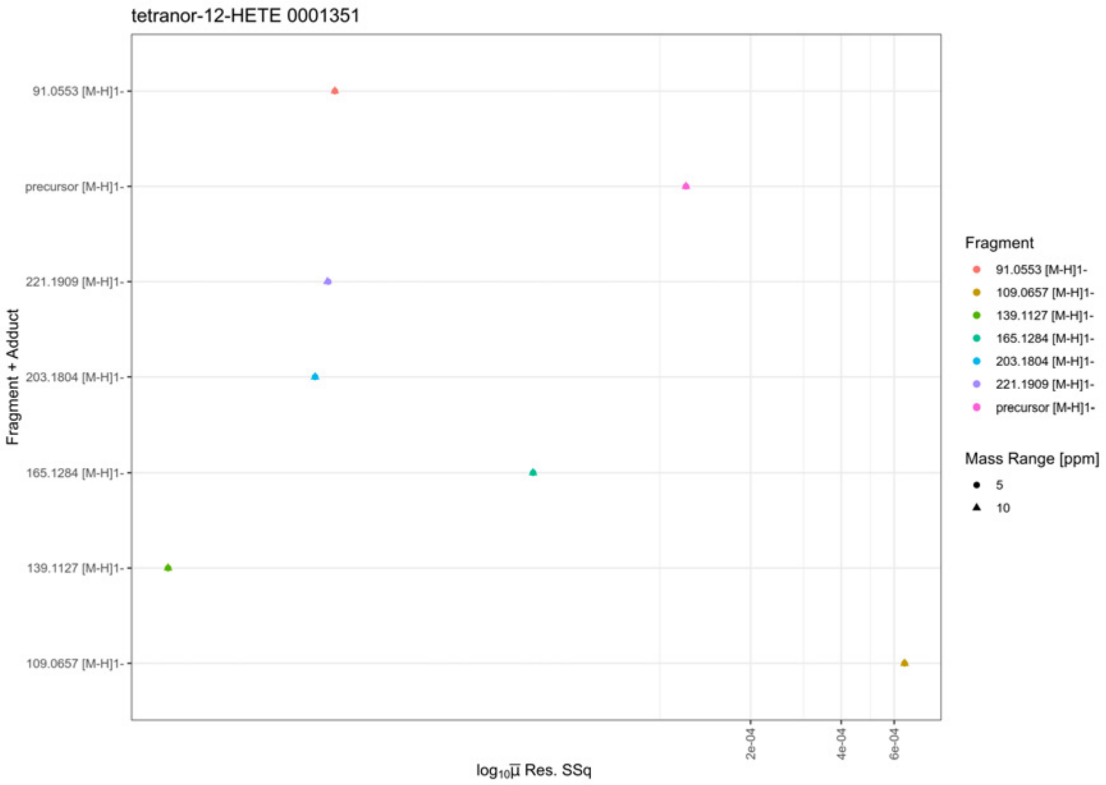

Figure 289. Normalized sum-of-squares of the residuals

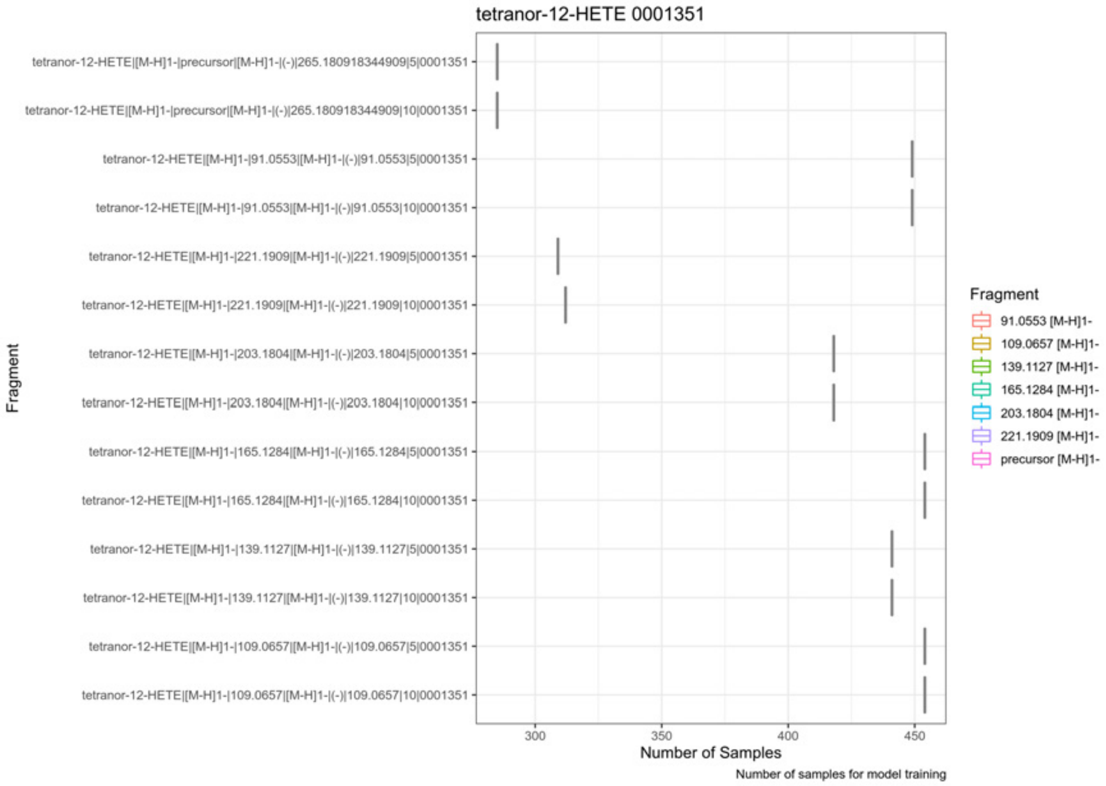

Figure 290. Number of samples used for training per combination Id
