# Supplementary figures and images for: LipidCreator workbench to probe the lipidomic landscape
Source: Nat Commun. 2020 Apr 28;11:2057. doi: 10.1038/s41467-020-15960-z (PMC7188904; doi:10.1038/s41467-020-15960-z)

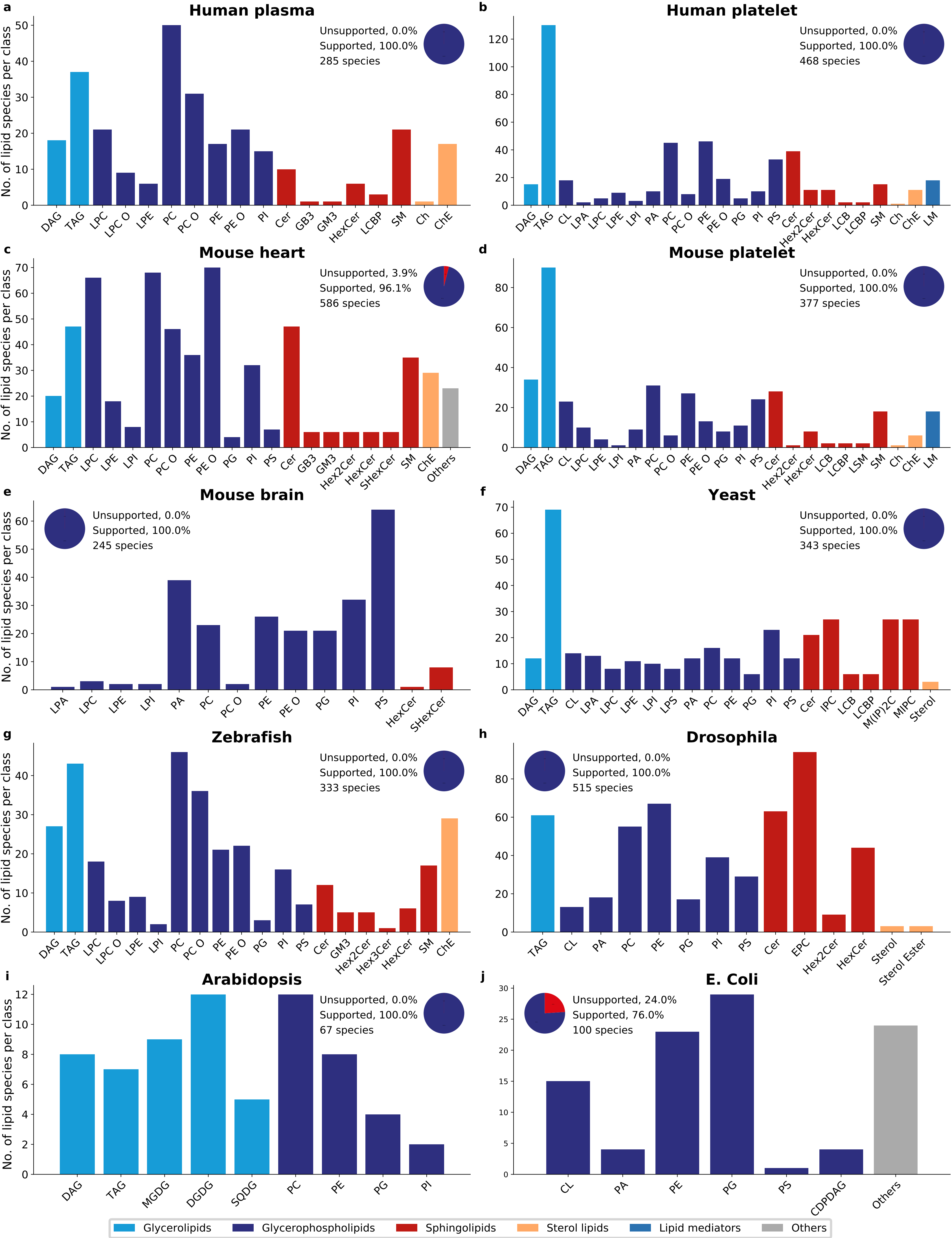

Supplement: Supplementary file 10 — Supplementary Data 7 [file 41467_2020_15960_MOESM10_ESM.zip › Scripts/Figure 4 - Lipid Distribution/lipid_distribution.pdf]

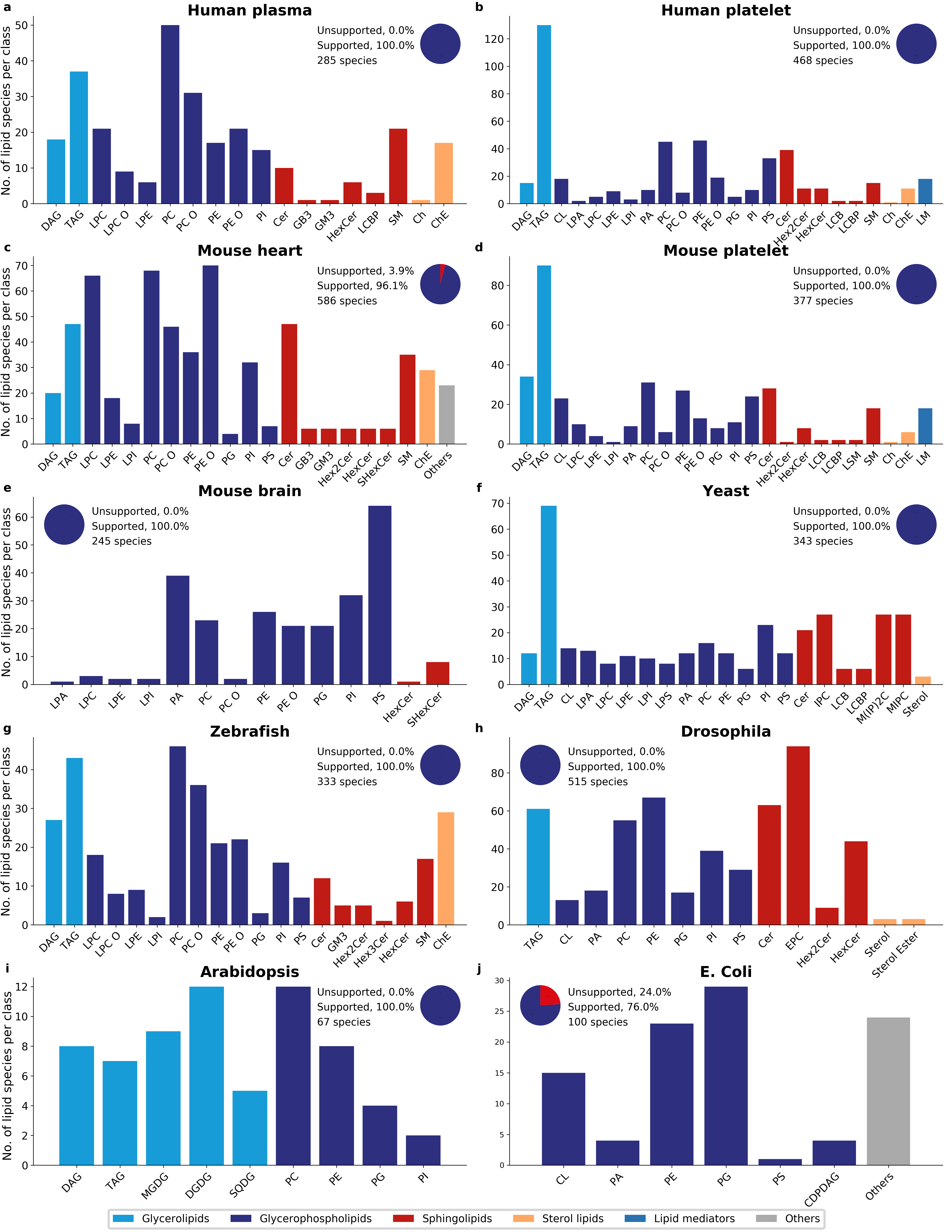

Supplement: Supplementary file 10 — Supplementary Data 7 [file 41467_2020_15960_MOESM10_ESM.zip › Scripts/Figure 4 - Lipid Distribution/lipid_distribution.png]

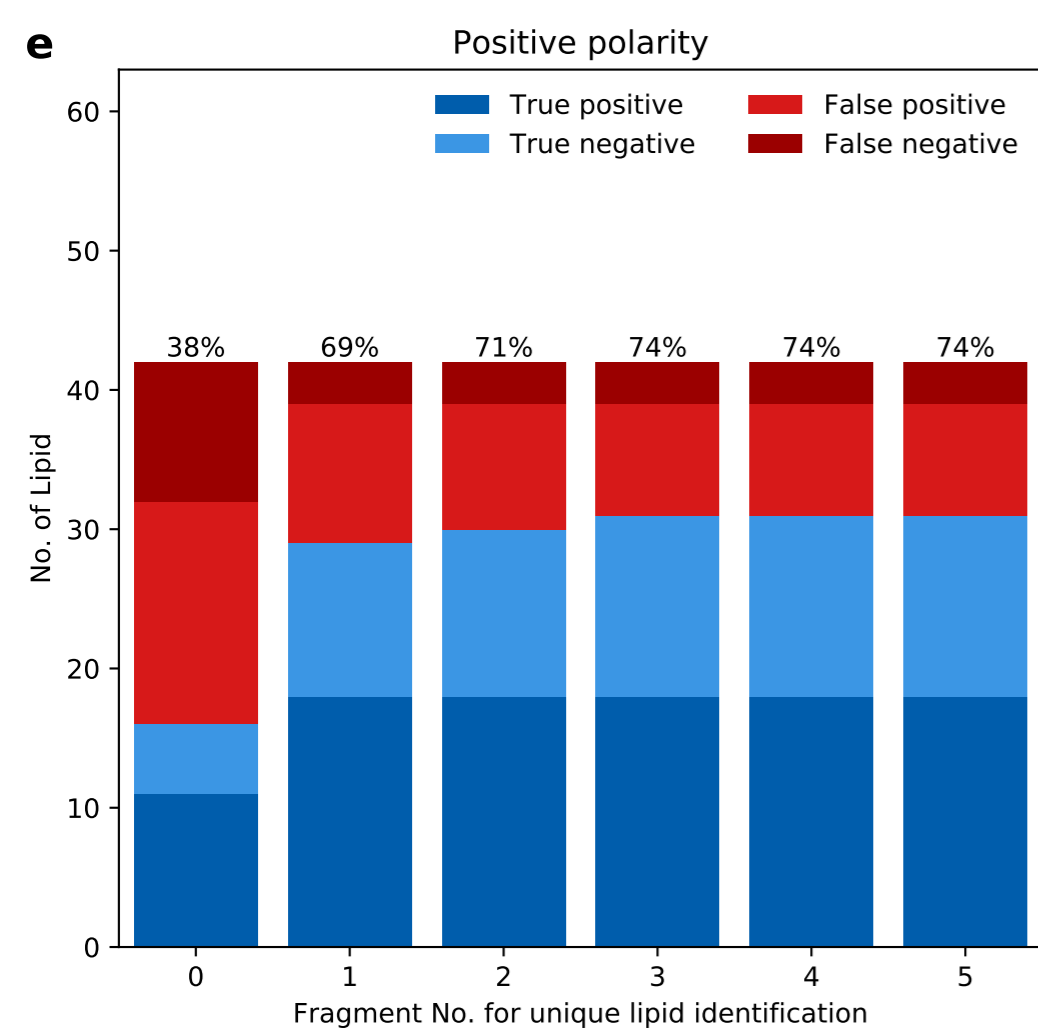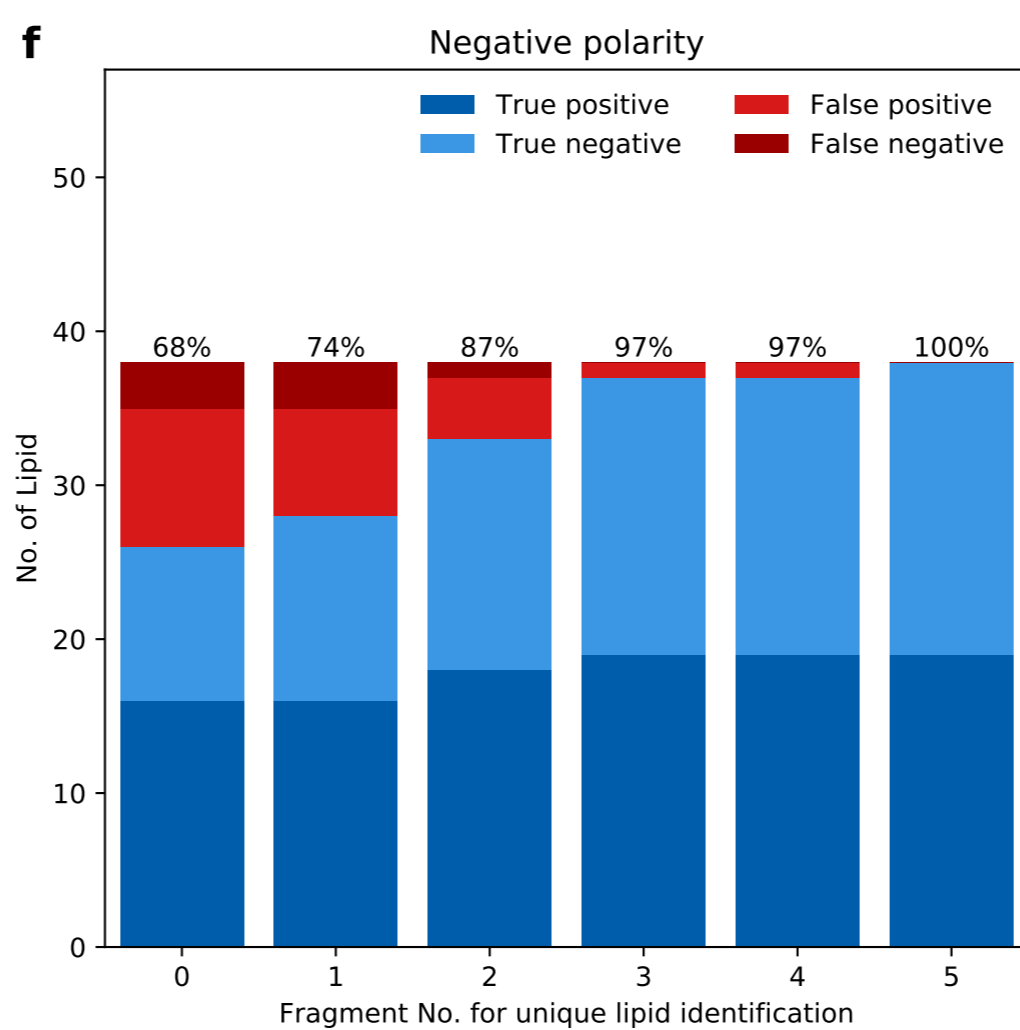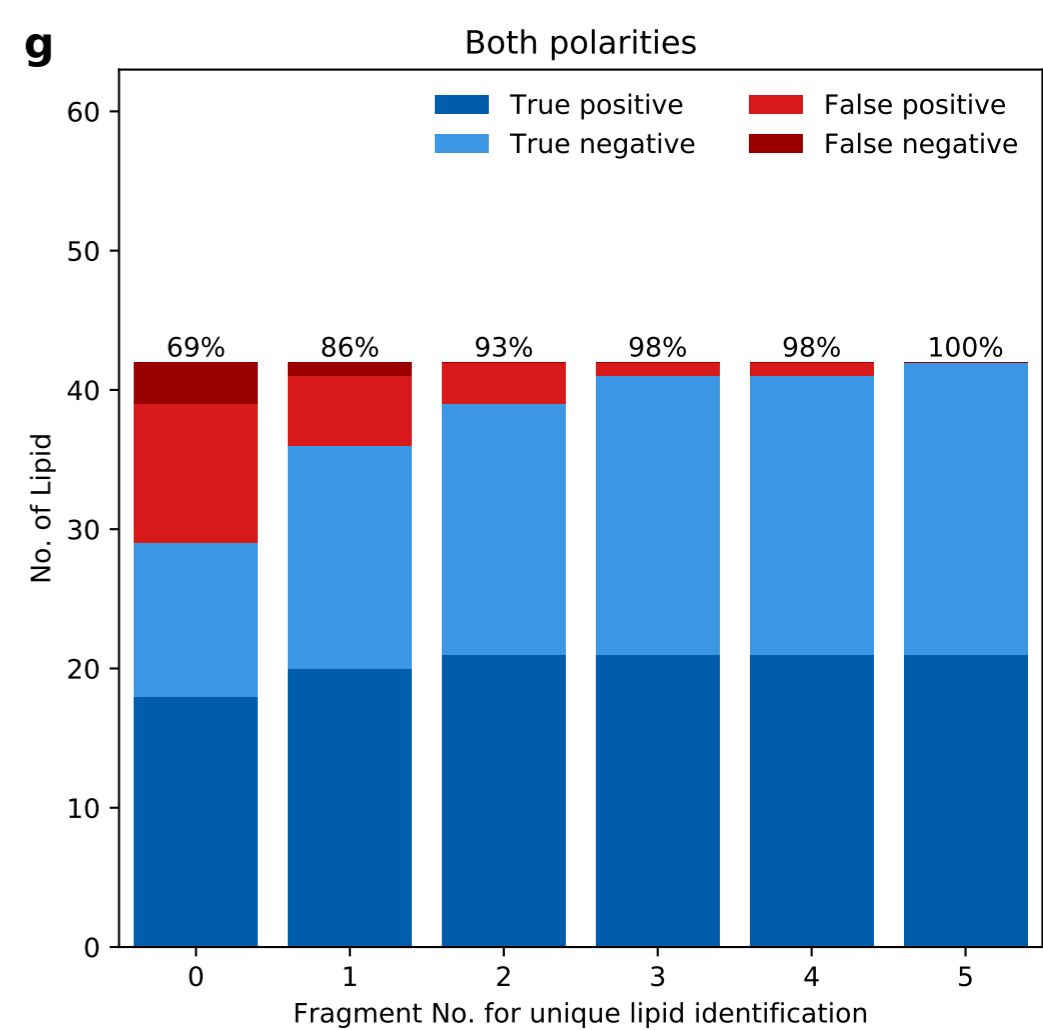

Supplement: Supplementary file 10 — Supplementary Data 7 [file 41467_2020_15960_MOESM10_ESM.zip › Scripts/Figure 5 - Probability and False Match/False-match.pdf]

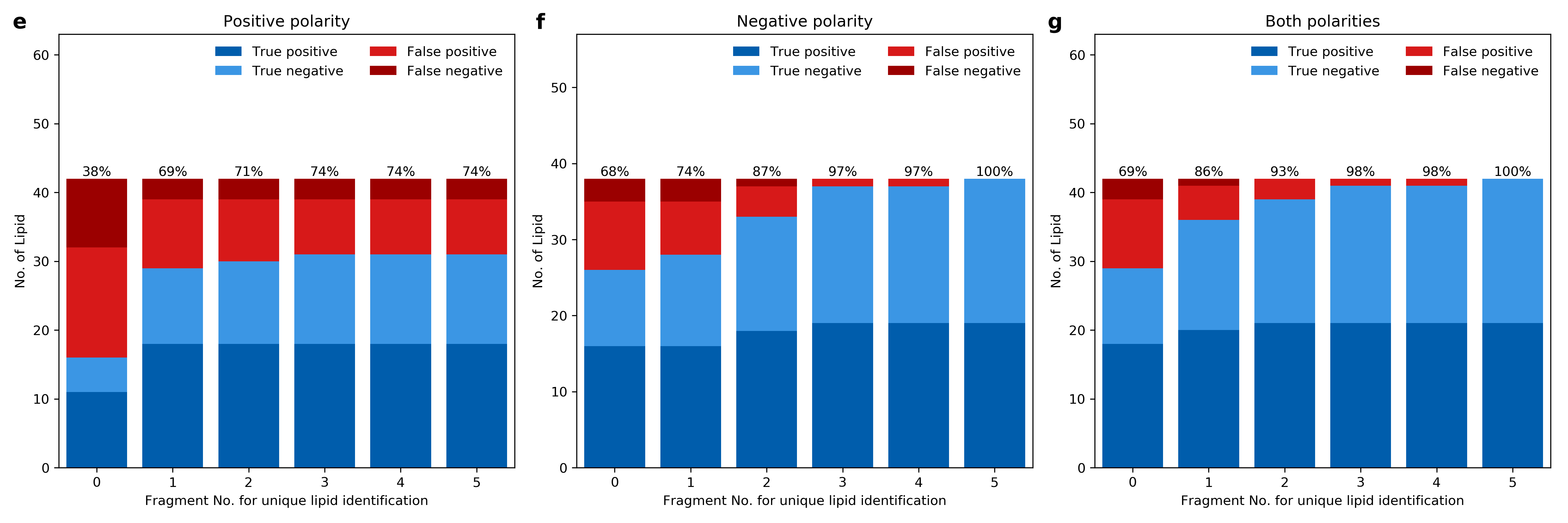

Supplement: Supplementary file 10 — Supplementary Data 7 [file 41467_2020_15960_MOESM10_ESM.zip › Scripts/Figure 5 - Probability and False Match/False-match.png]

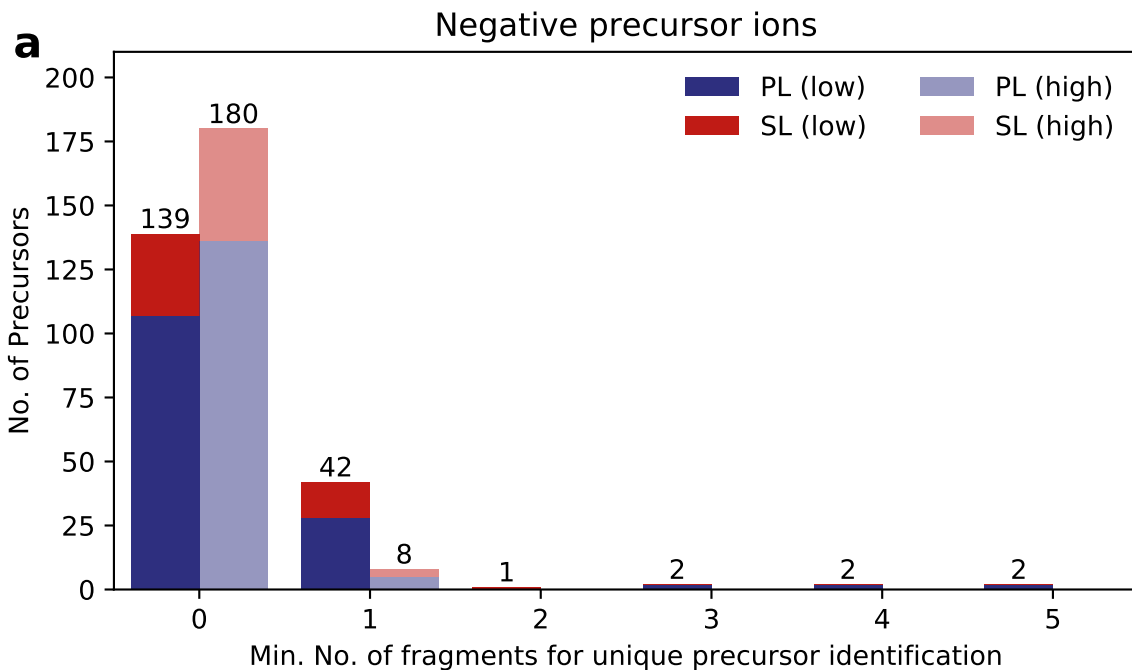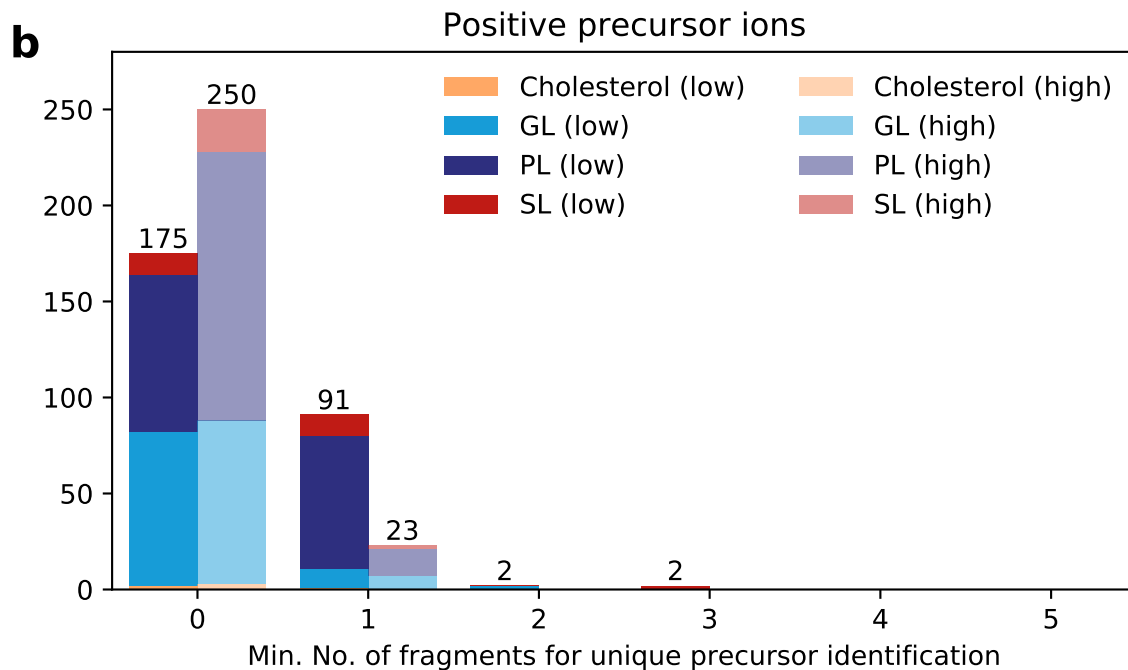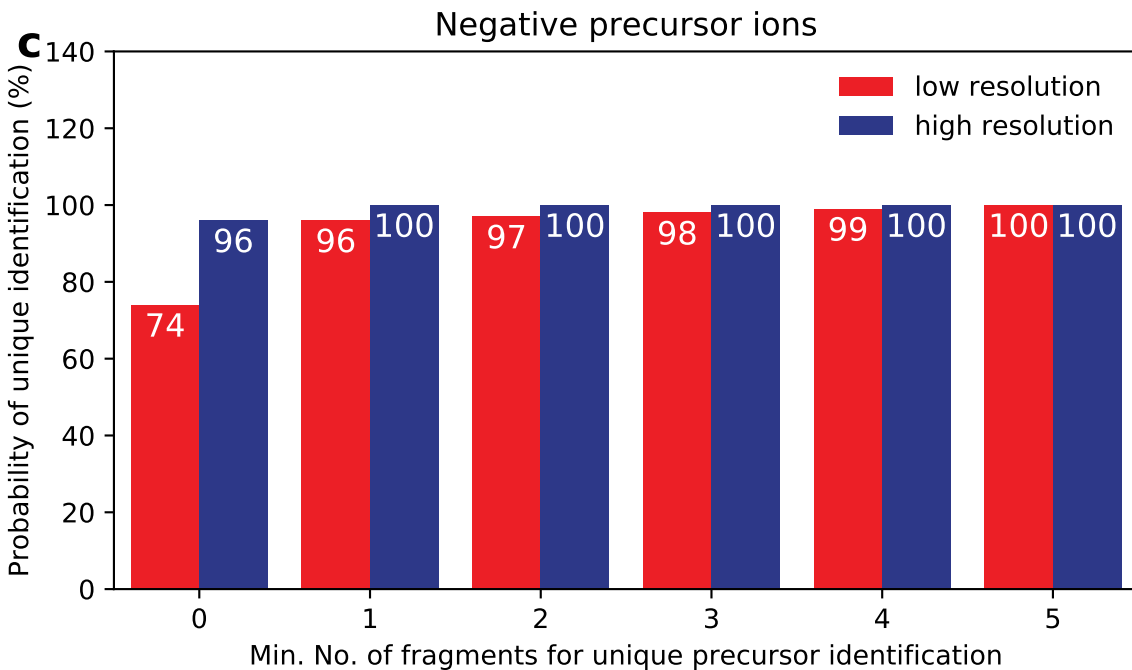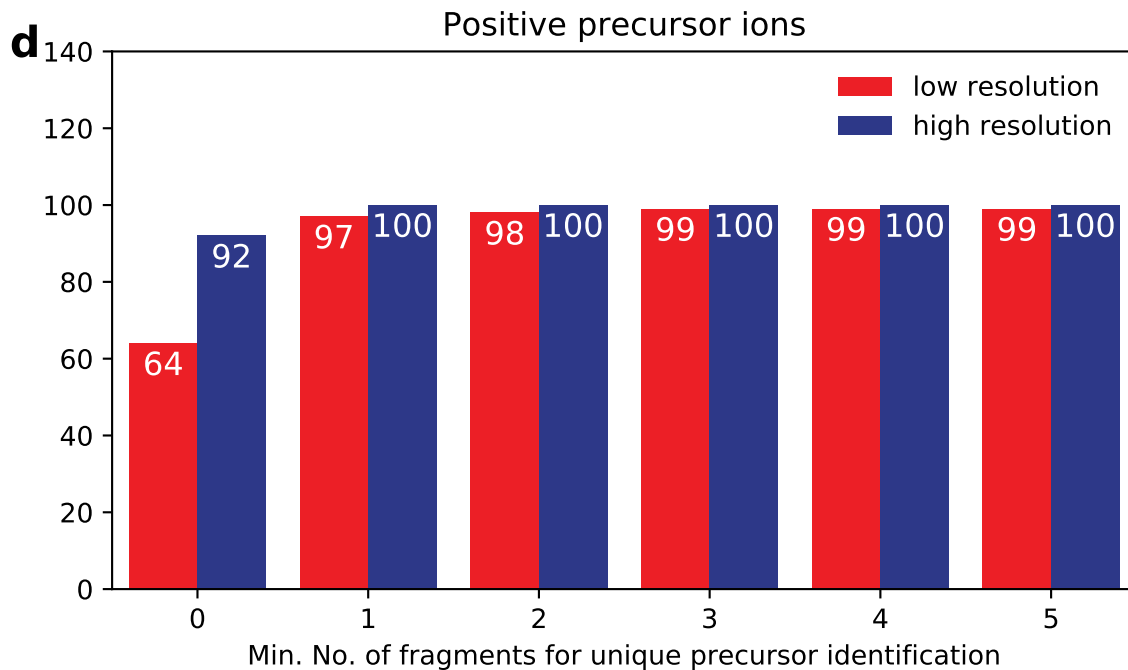

Supplement: Supplementary file 10 — Supplementary Data 7 [file 41467_2020_15960_MOESM10_ESM.zip › Scripts/Figure 5 - Probability and False Match/Probability-plot.pdf]

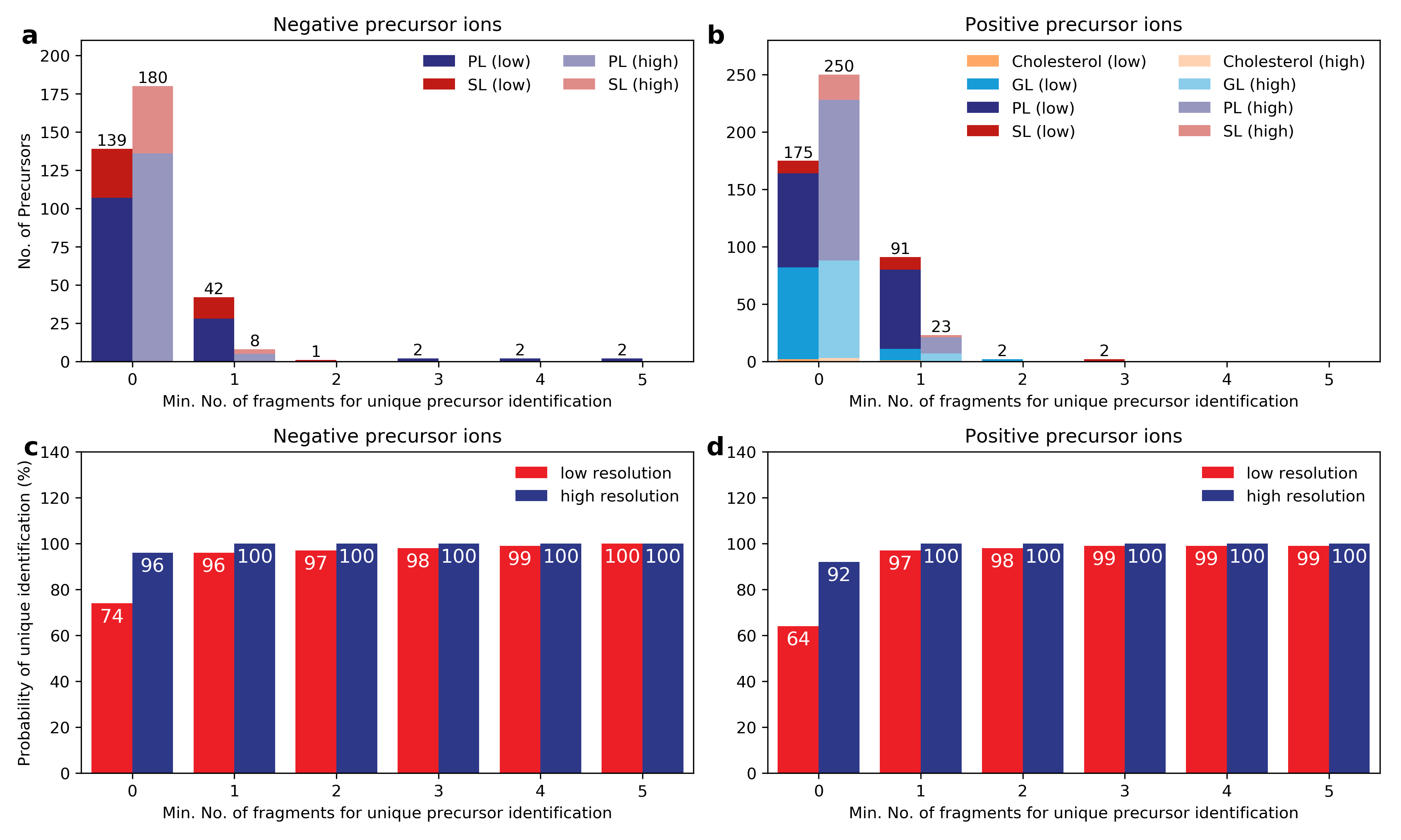

Supplement: Supplementary file 10 — Supplementary Data 7 [file 41467_2020_15960_MOESM10_ESM.zip › Scripts/Figure 5 - Probability and False Match/Probability-plot.png]

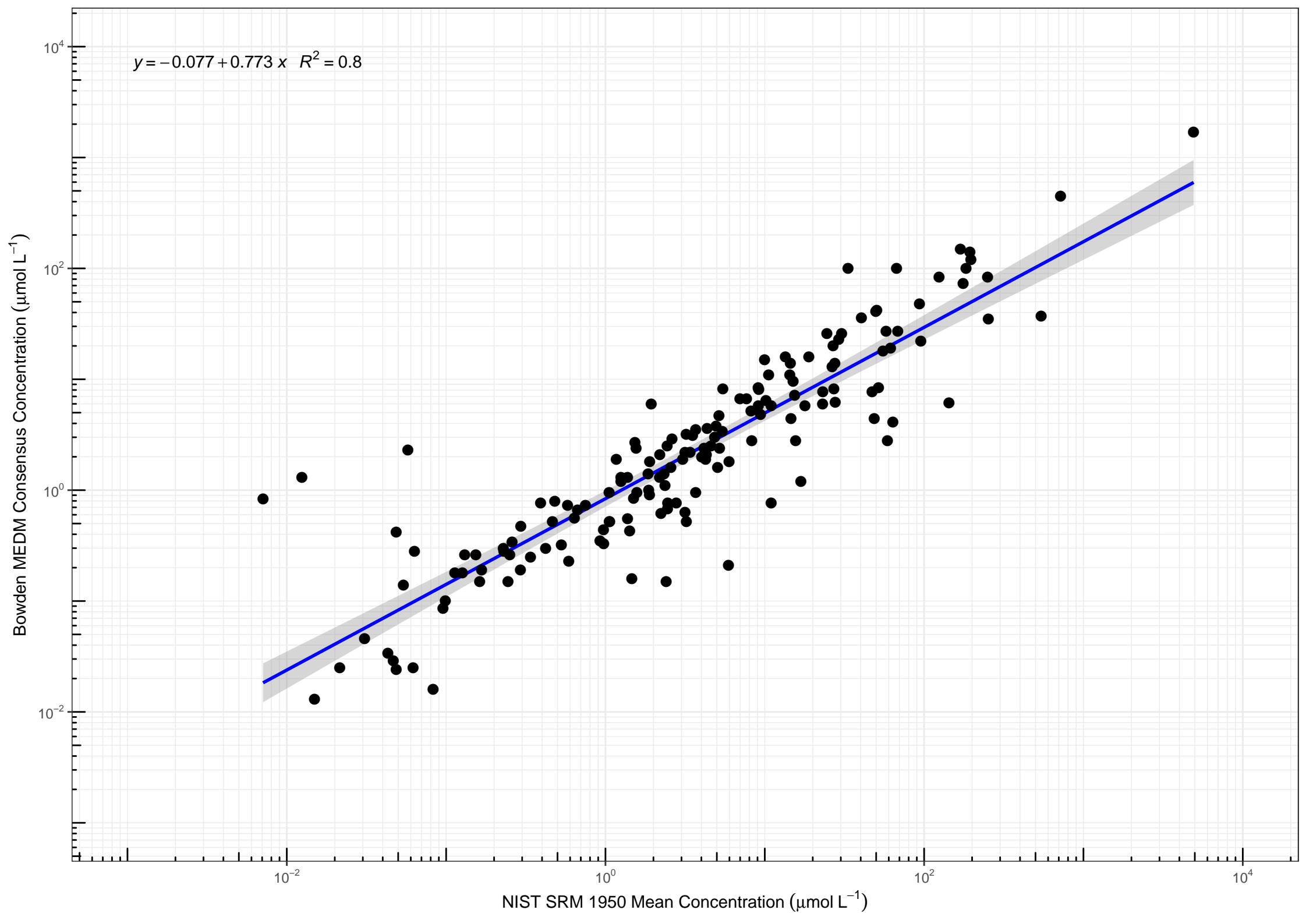

Supplement: Supplementary file 10 — Supplementary Data 7 [file 41467_2020_15960_MOESM10_ESM.zip › Scripts/Figure 6 - Quantitative comparisons/nistVsMedmPlotLinearCorrelation.pdf]

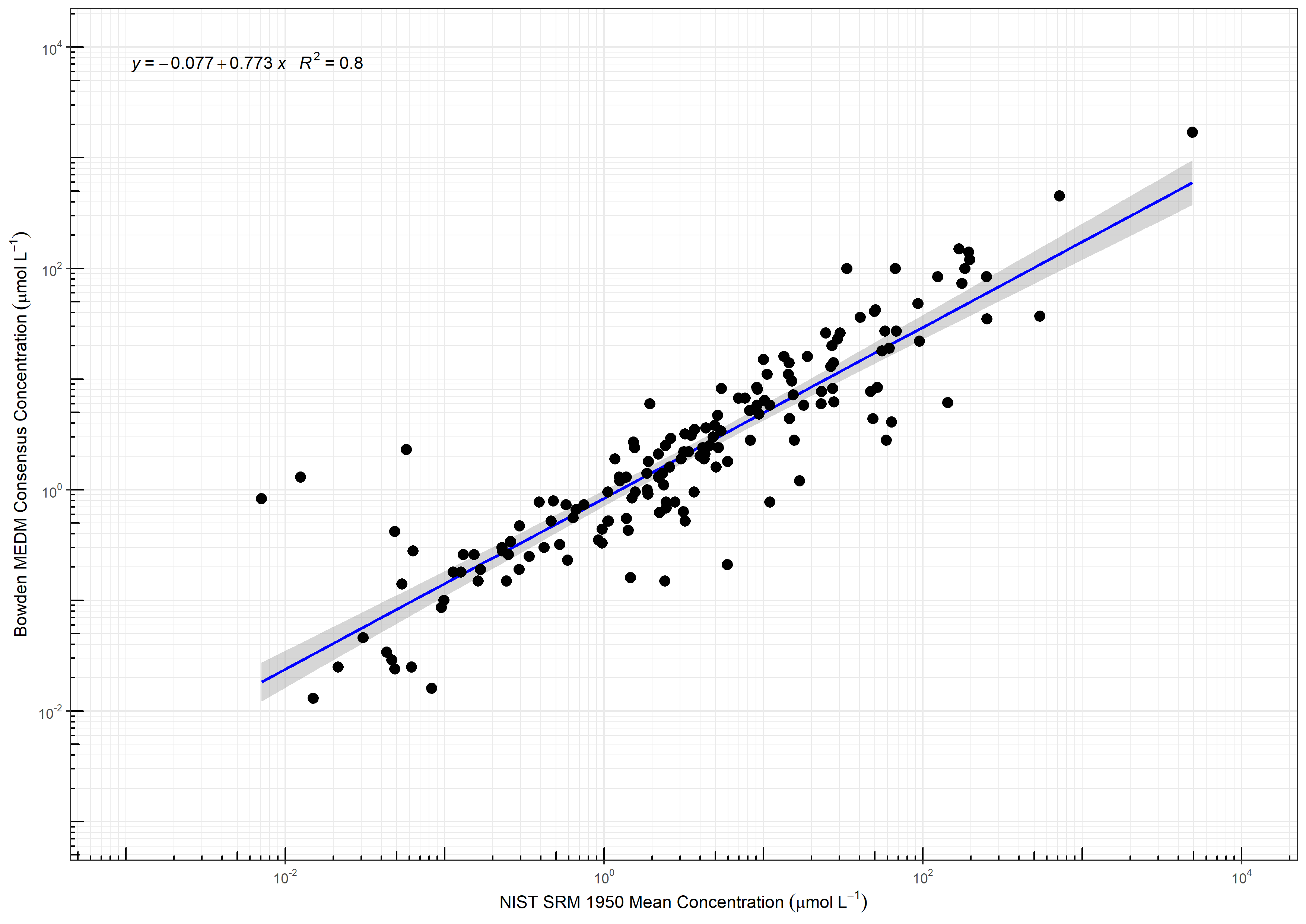

Supplement: Supplementary file 10 — Supplementary Data 7 [file 41467_2020_15960_MOESM10_ESM.zip › Scripts/Figure 6 - Quantitative comparisons/nistVsMedmPlotLinearCorrelation.png]

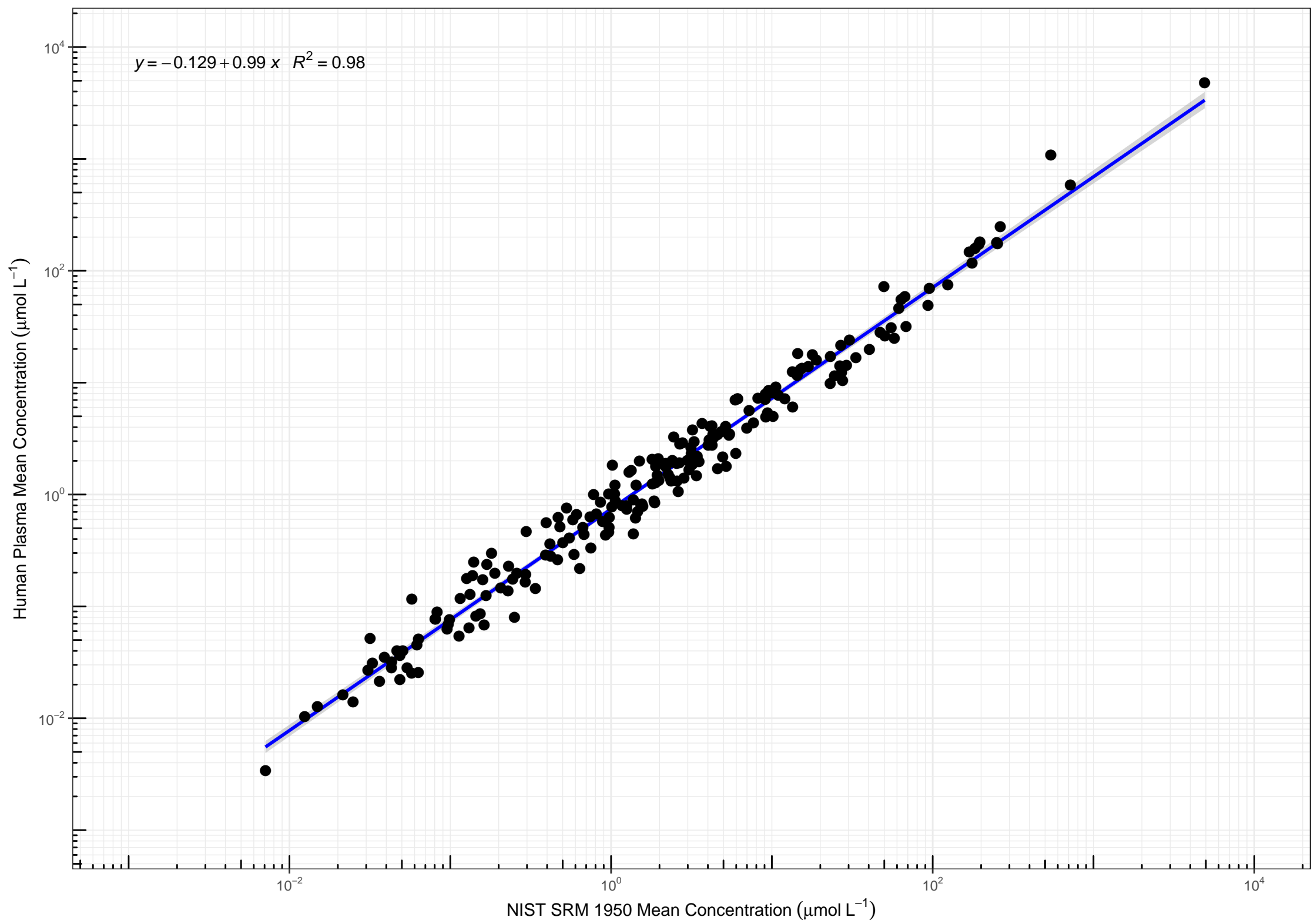

Supplement: Supplementary file 10 — Supplementary Data 7 [file 41467_2020_15960_MOESM10_ESM.zip › Scripts/Figure 6 - Quantitative comparisons/nistVsSubjectsPlotLinearCorrelation.pdf]

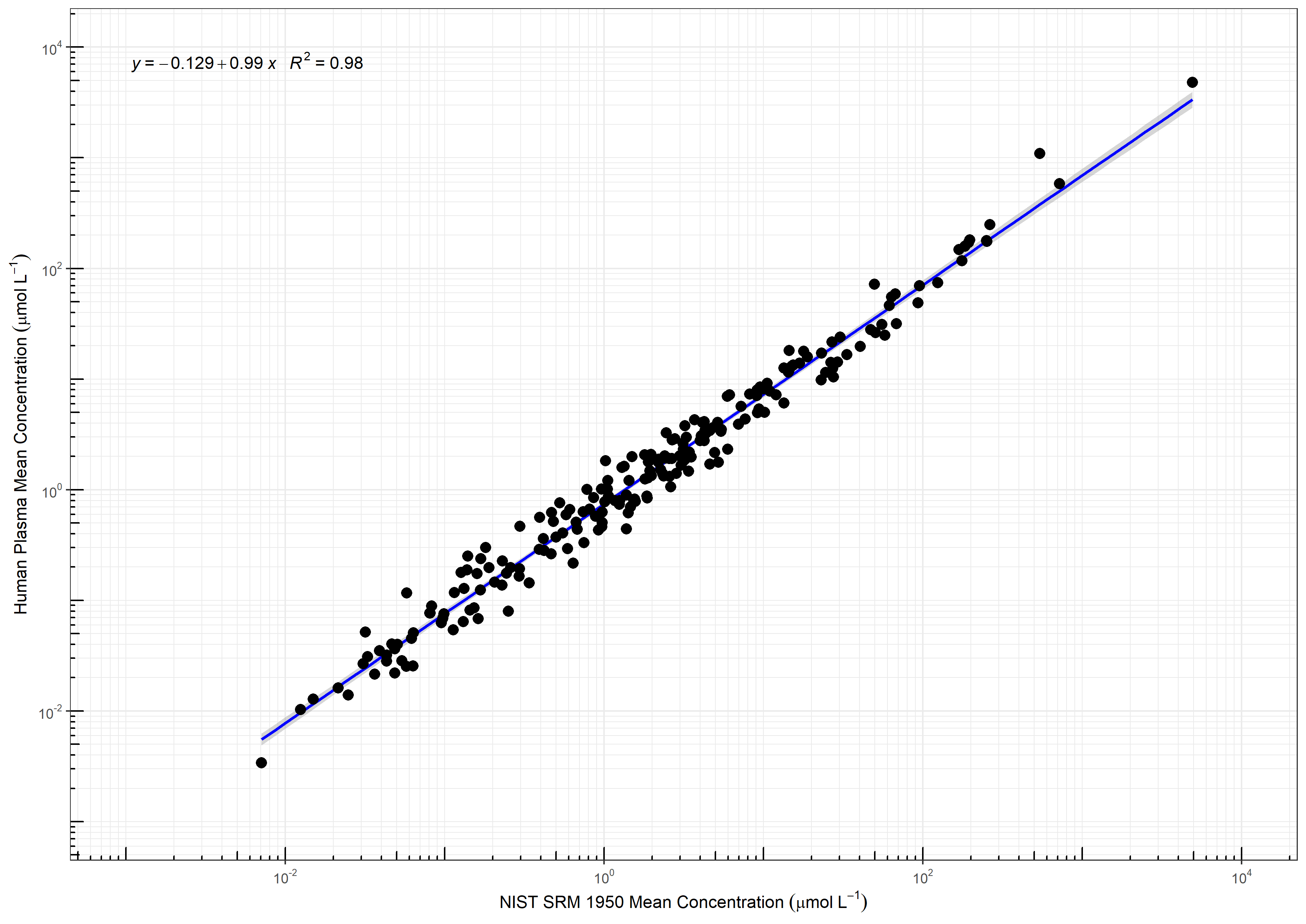

Supplement: Supplementary file 10 — Supplementary Data 7 [file 41467_2020_15960_MOESM10_ESM.zip › Scripts/Figure 6 - Quantitative comparisons/nistVsSubjectsPlotLinearCorrelation.png]

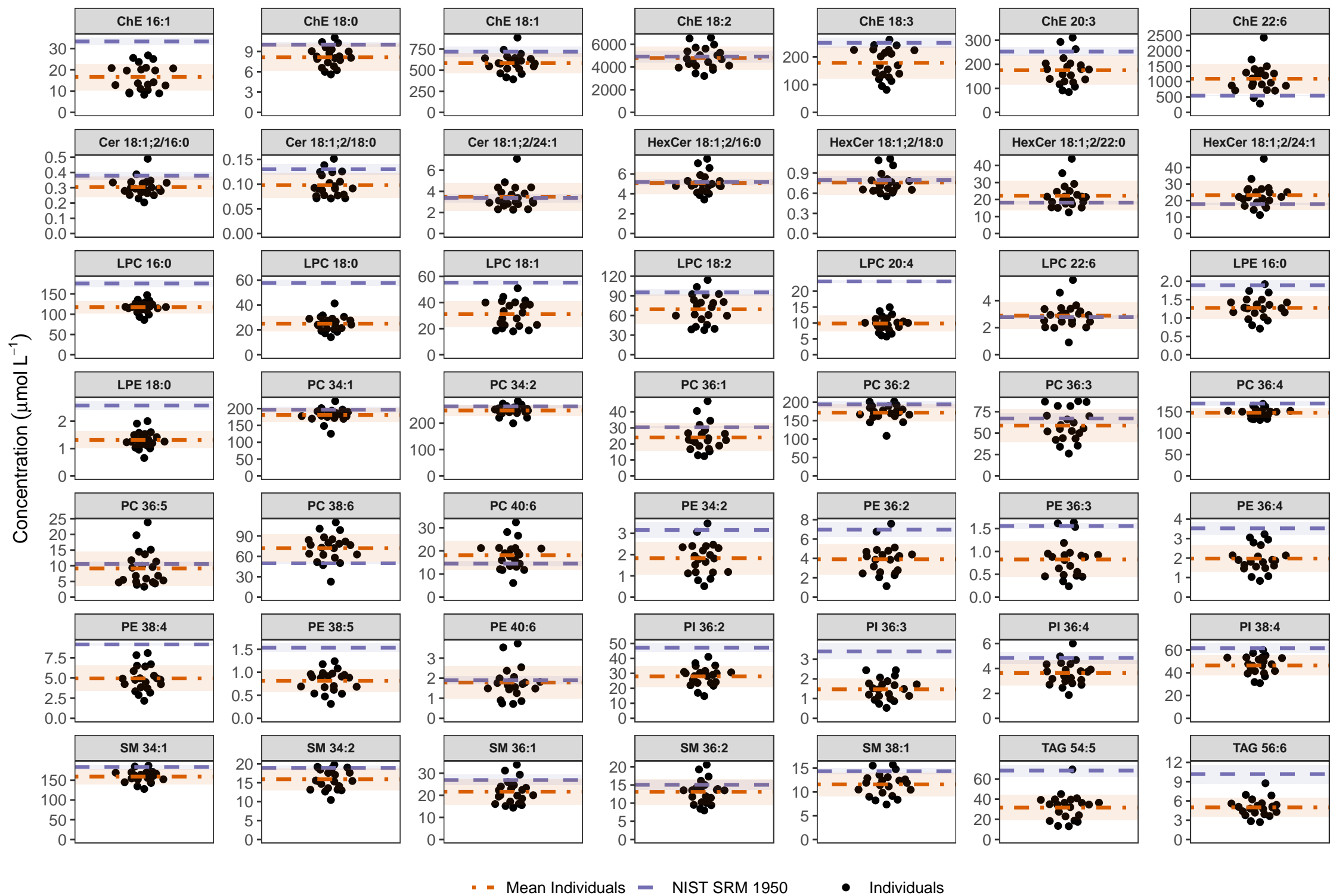

Supplement: Supplementary file 10 — Supplementary Data 7 [file 41467_2020_15960_MOESM10_ESM.zip › Scripts/Figure 6 - Quantitative comparisons/plasmaComparisonPlot.pdf]

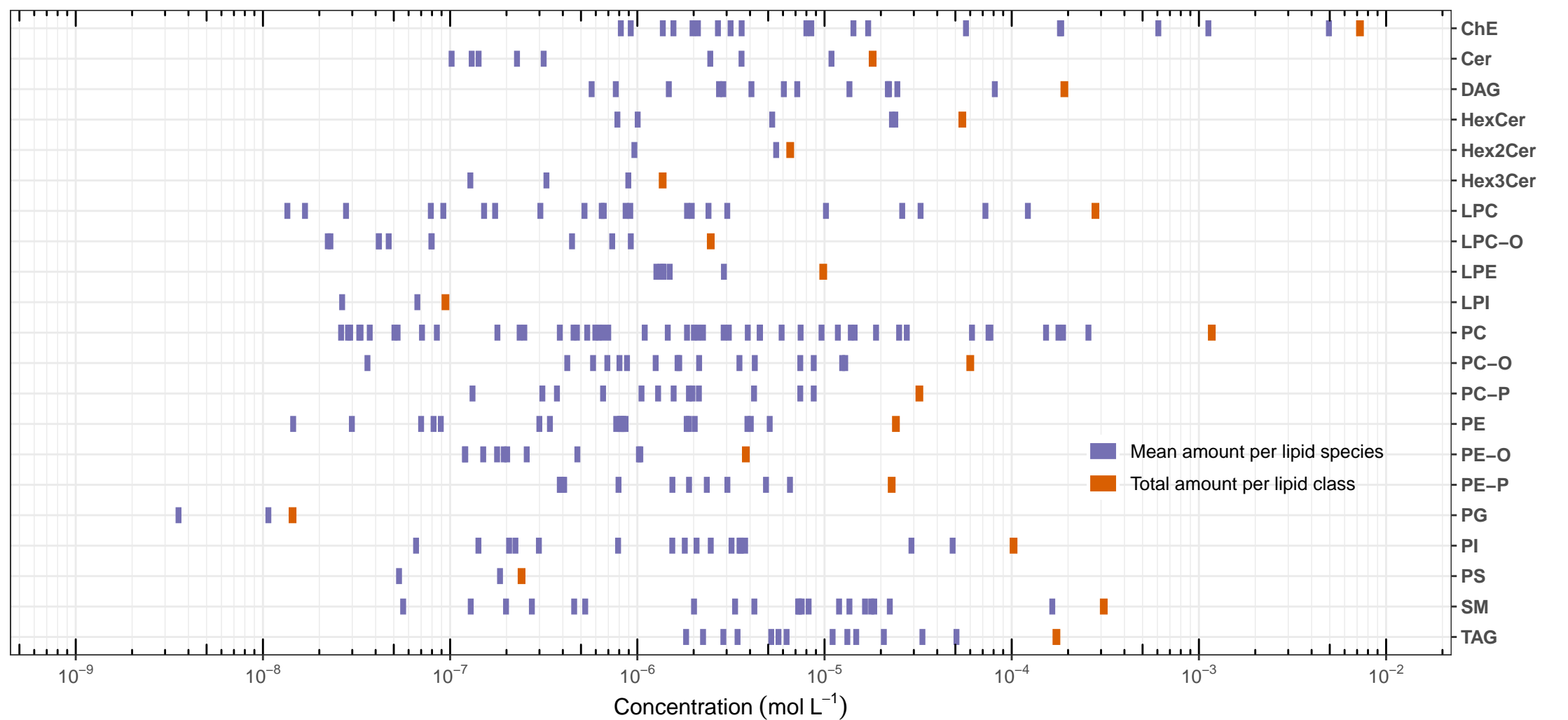

Supplement: Supplementary file 10 — Supplementary Data 7 [file 41467_2020_15960_MOESM10_ESM.zip › Scripts/Figure 6 - Quantitative comparisons/plasmaConcentrationRangePlot.pdf]

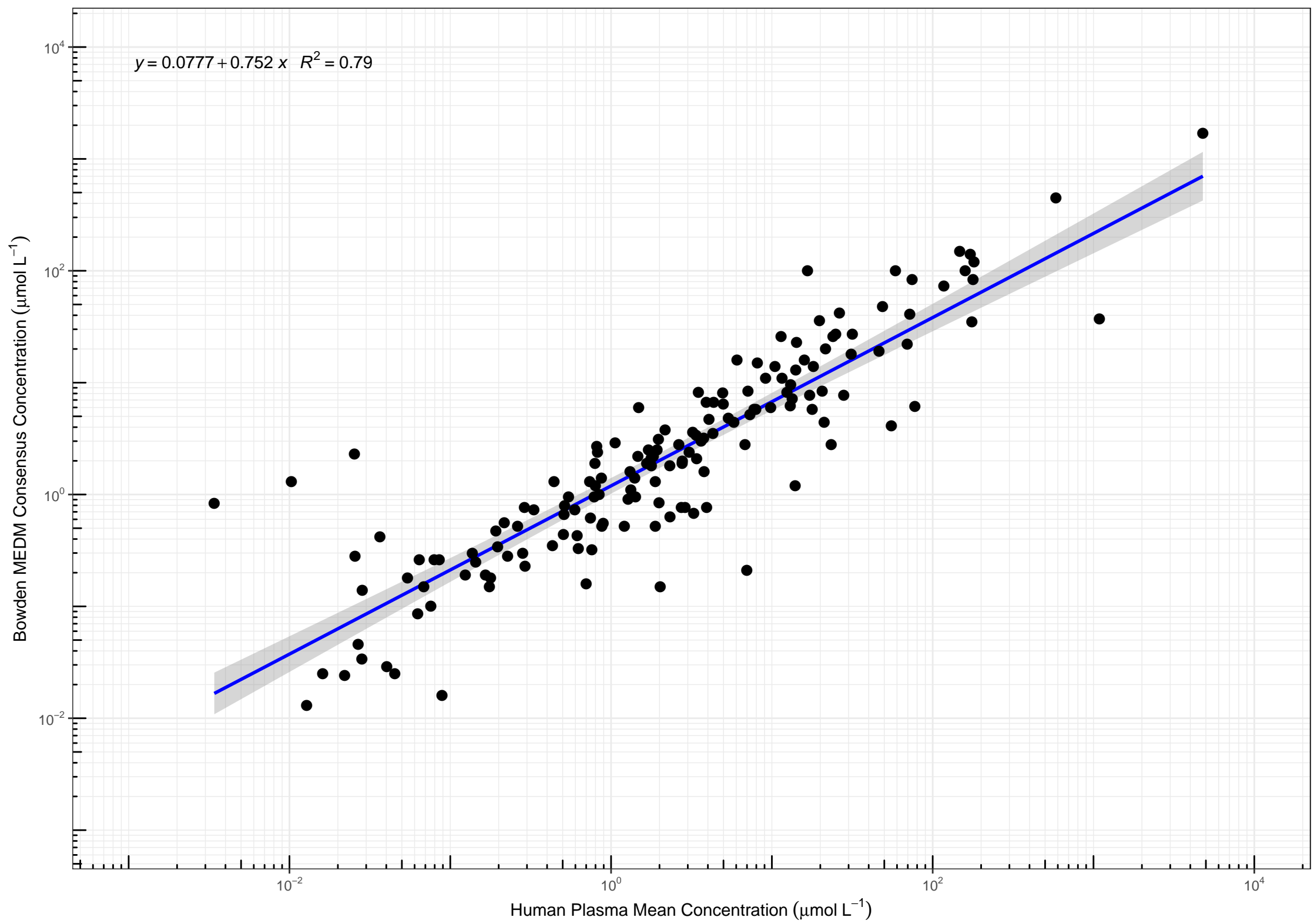

Supplement: Supplementary file 10 — Supplementary Data 7 [file 41467_2020_15960_MOESM10_ESM.zip › Scripts/Figure 6 - Quantitative comparisons/plasmaVsMedmPlotLinearCorrelation.pdf]

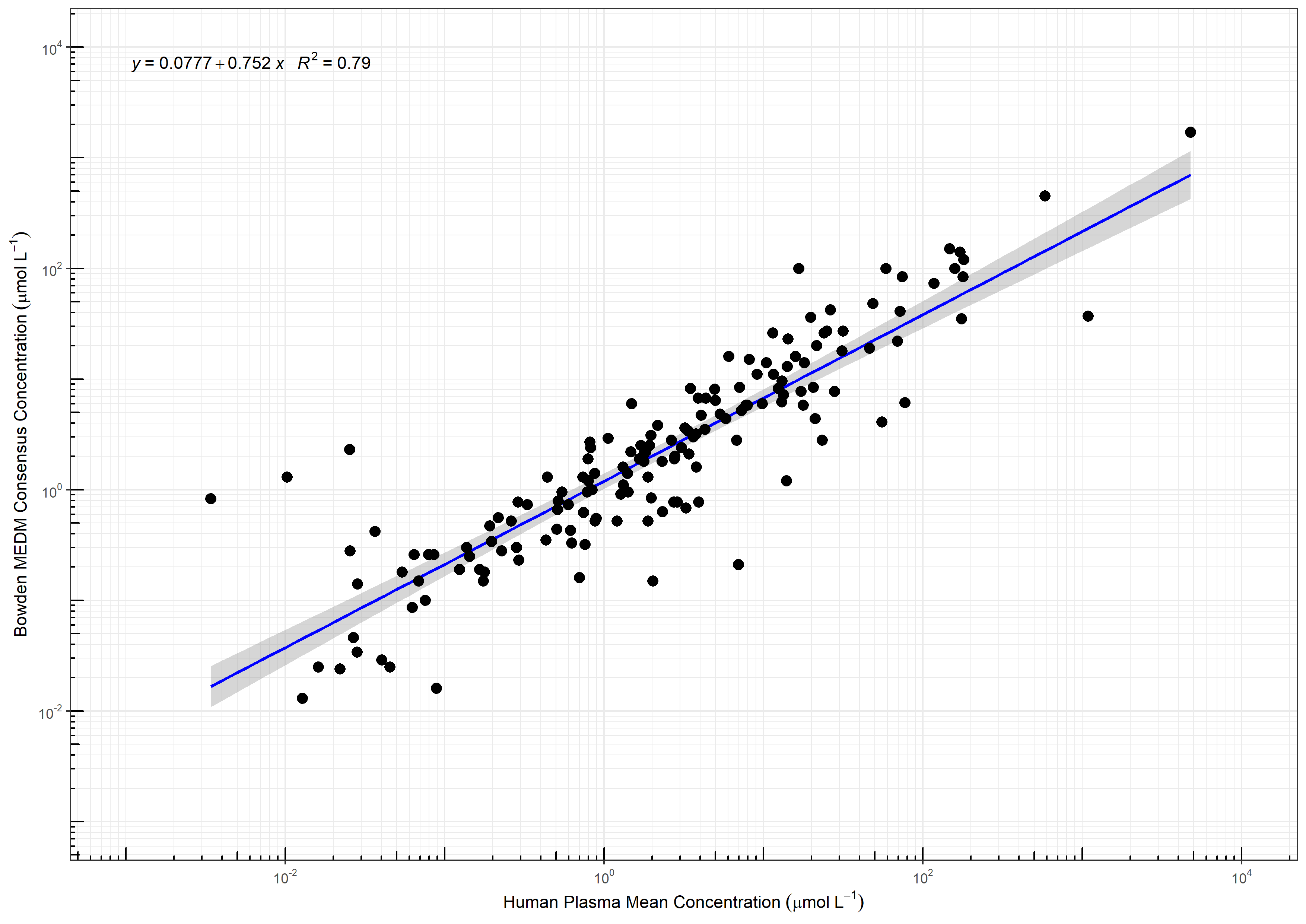

Supplement: Supplementary file 10 — Supplementary Data 7 [file 41467_2020_15960_MOESM10_ESM.zip › Scripts/Figure 6 - Quantitative comparisons/plasmaVsMedmPlotLinearCorrelation.png]

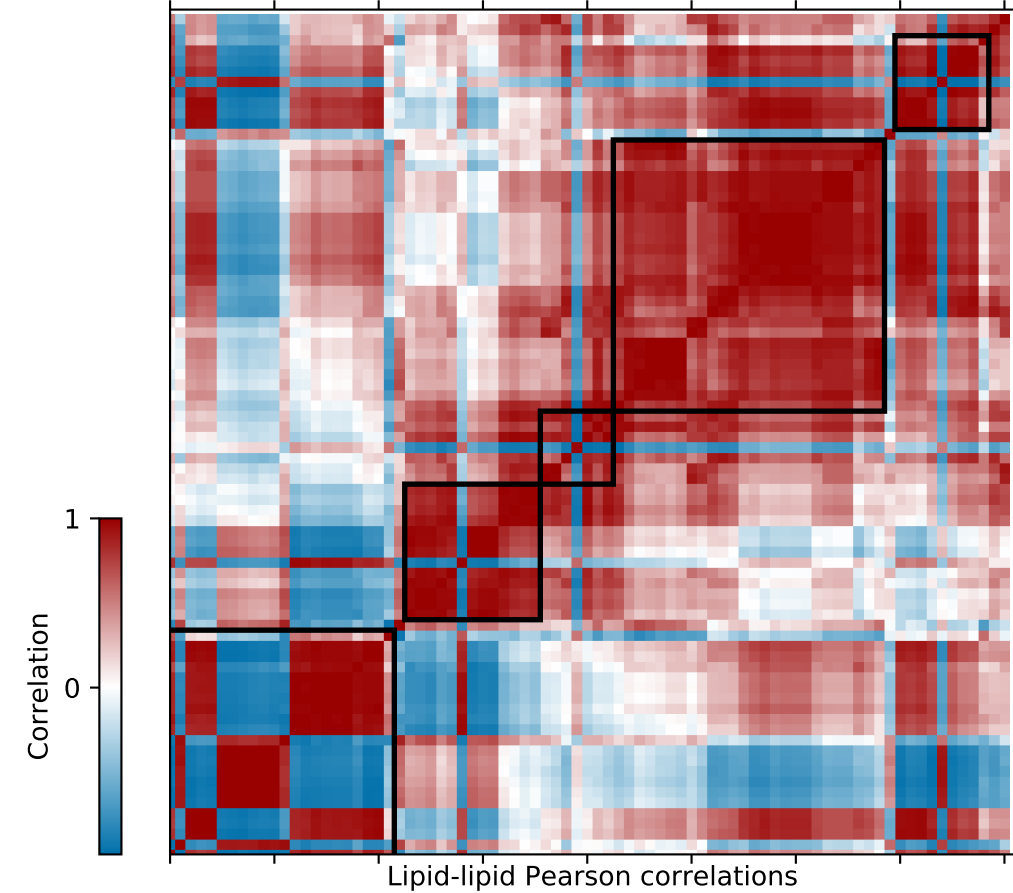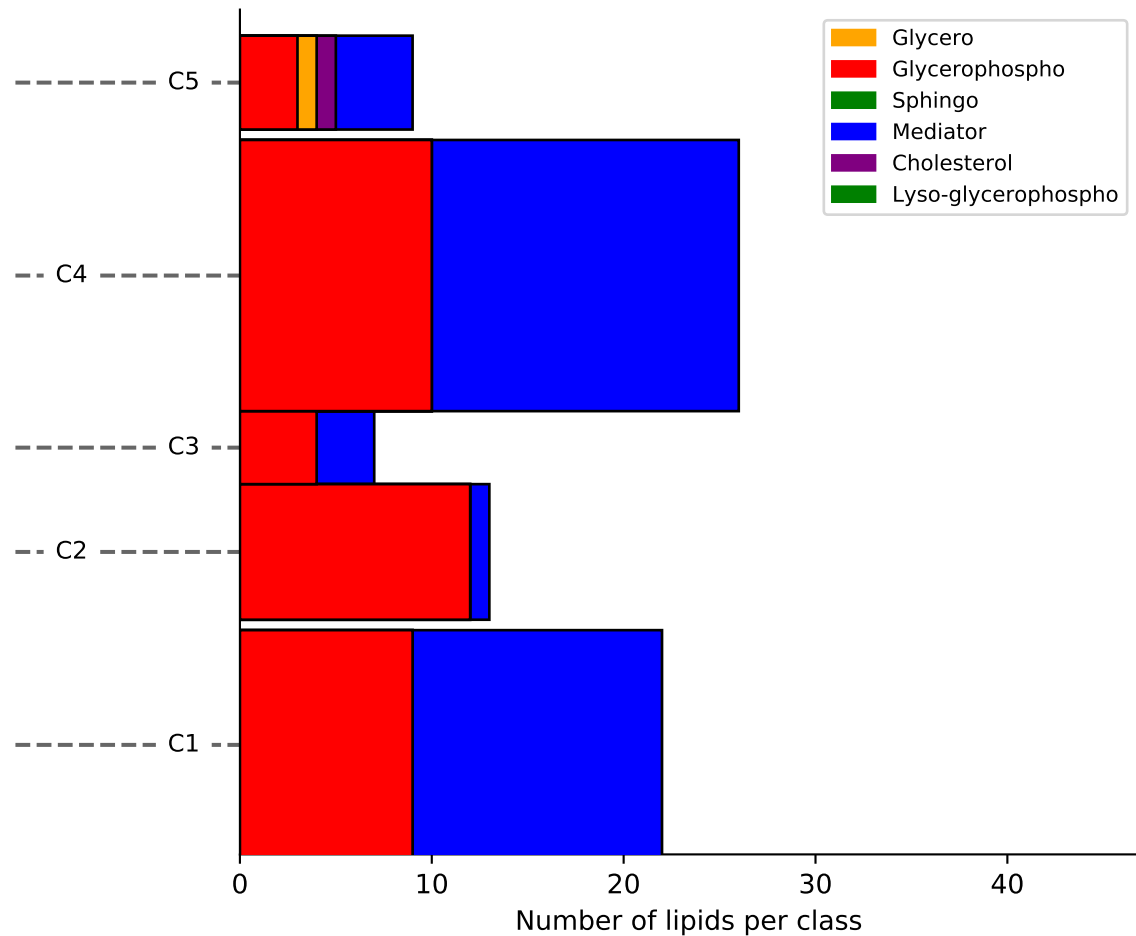

Supplement: Supplementary file 10 — Supplementary Data 7 [file 41467_2020_15960_MOESM10_ESM.zip › Scripts/Figure 7 - Lipid Network/Figures and Output/correlation_and_lipid_group.pdf]

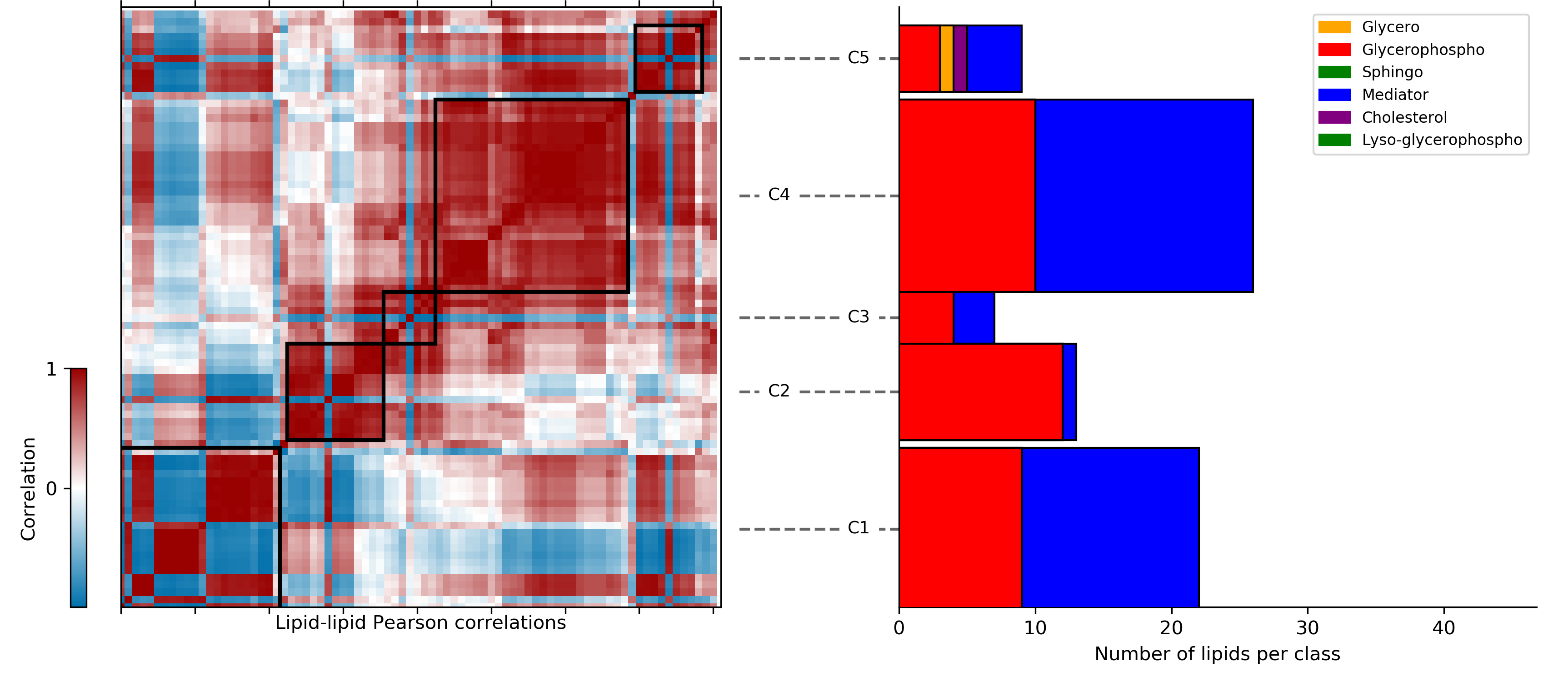

Supplement: Supplementary file 10 — Supplementary Data 7 [file 41467_2020_15960_MOESM10_ESM.zip › Scripts/Figure 7 - Lipid Network/Figures and Output/correlation_and_lipid_group.png]

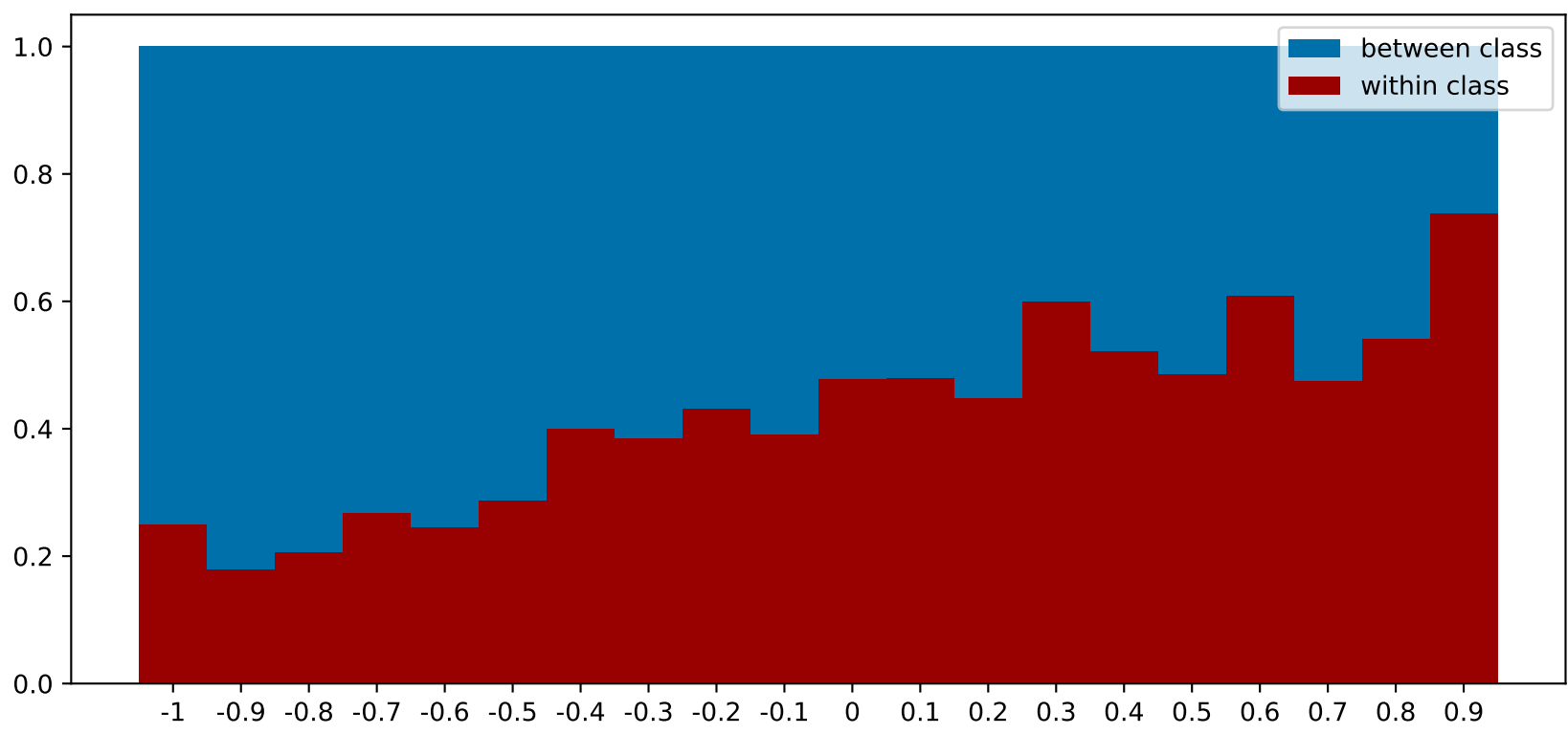

Supplement: Supplementary file 10 — Supplementary Data 7 [file 41467_2020_15960_MOESM10_ESM.zip › Scripts/Figure 7 - Lipid Network/Figures and Output/lipid_lipid_fraction.pdf]

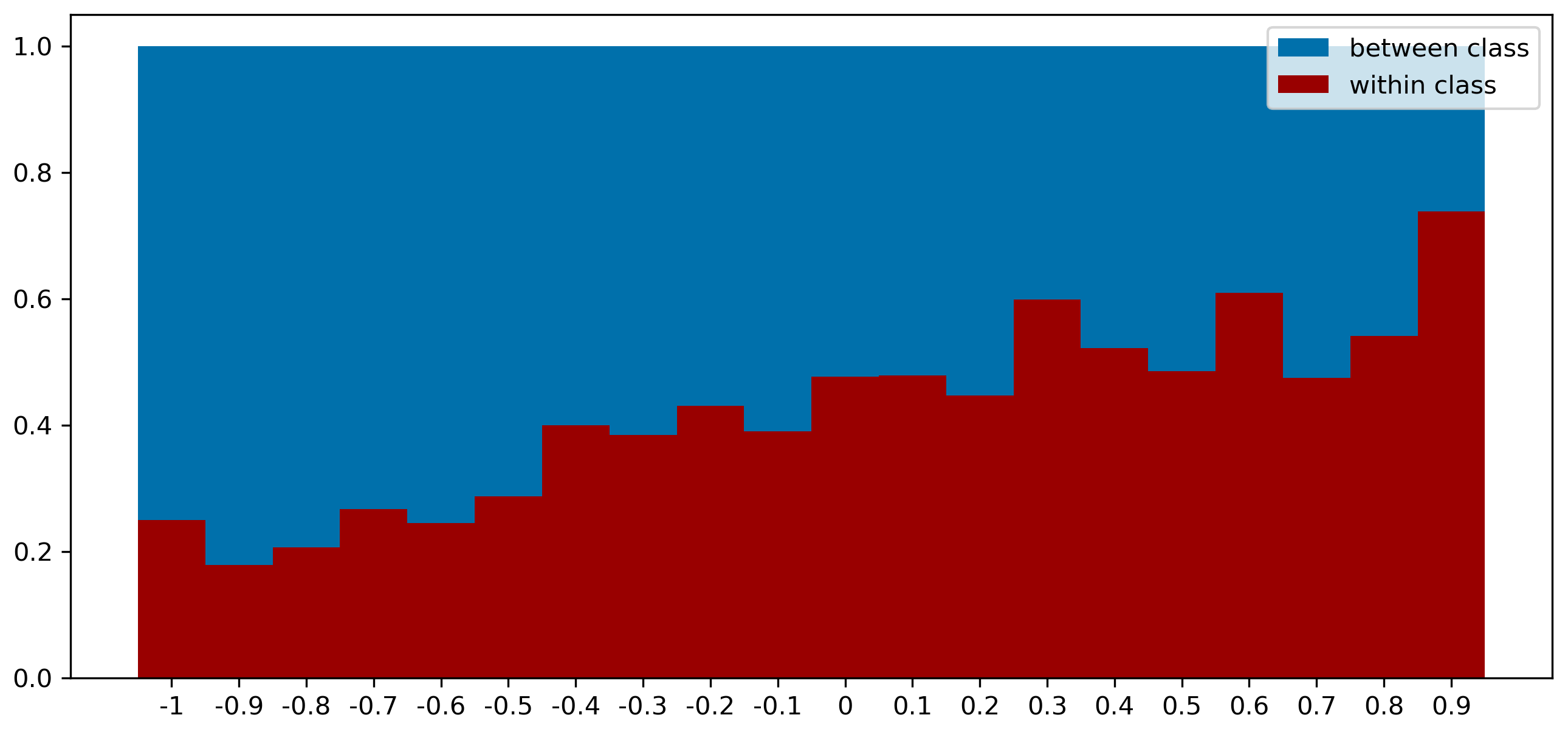

Supplement: Supplementary file 10 — Supplementary Data 7 [file 41467_2020_15960_MOESM10_ESM.zip › Scripts/Figure 7 - Lipid Network/Figures and Output/lipid_lipid_fraction.png]

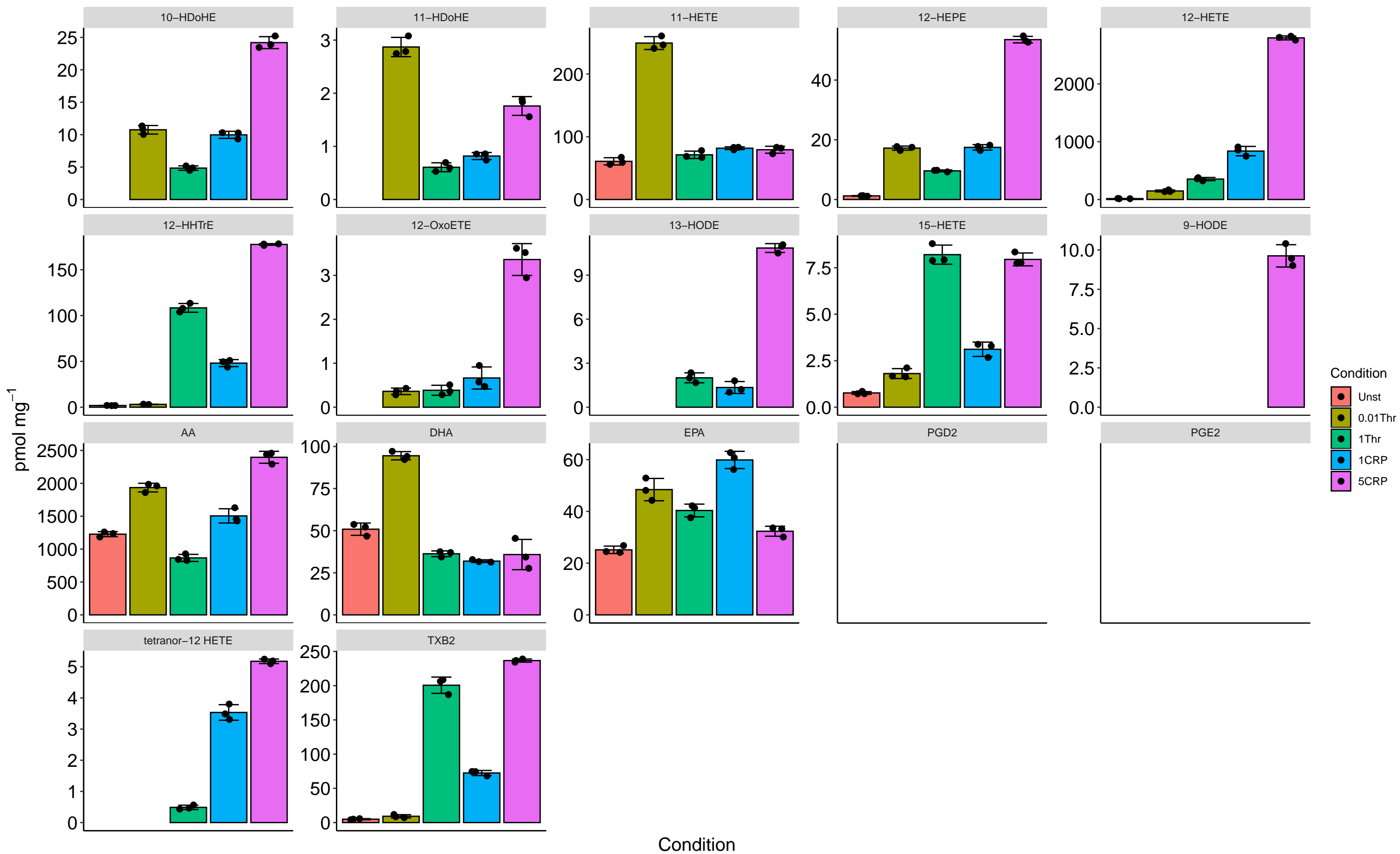

Supplement: Supplementary file 10 — Supplementary Data 7 [file 41467_2020_15960_MOESM10_ESM.zip › Scripts/Figure 7f - Lipid Mediators/figure7-f-pellet.pdf]

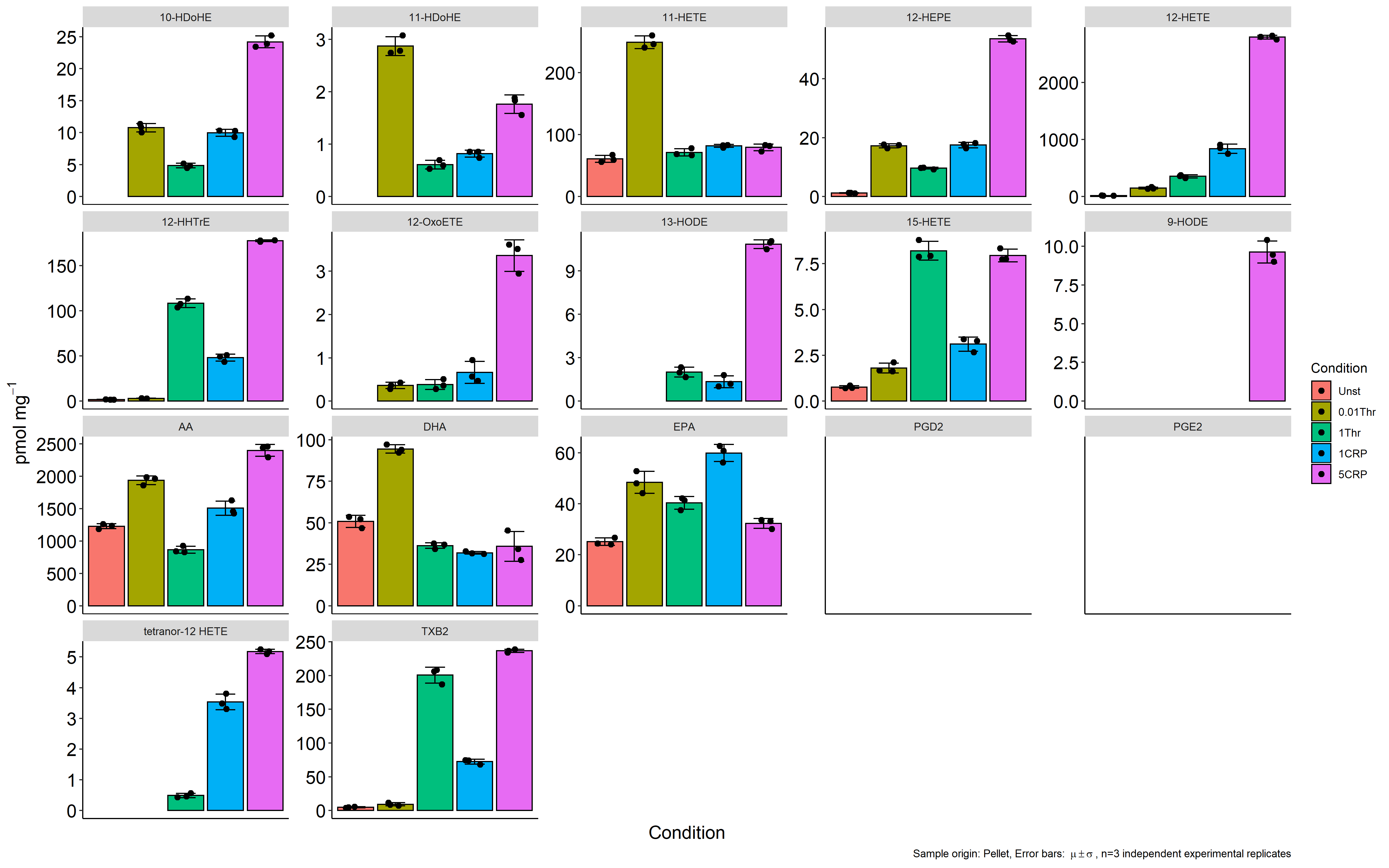

Supplement: Supplementary file 10 — Supplementary Data 7 [file 41467_2020_15960_MOESM10_ESM.zip › Scripts/Figure 7f - Lipid Mediators/figure7-f-pellet.png]

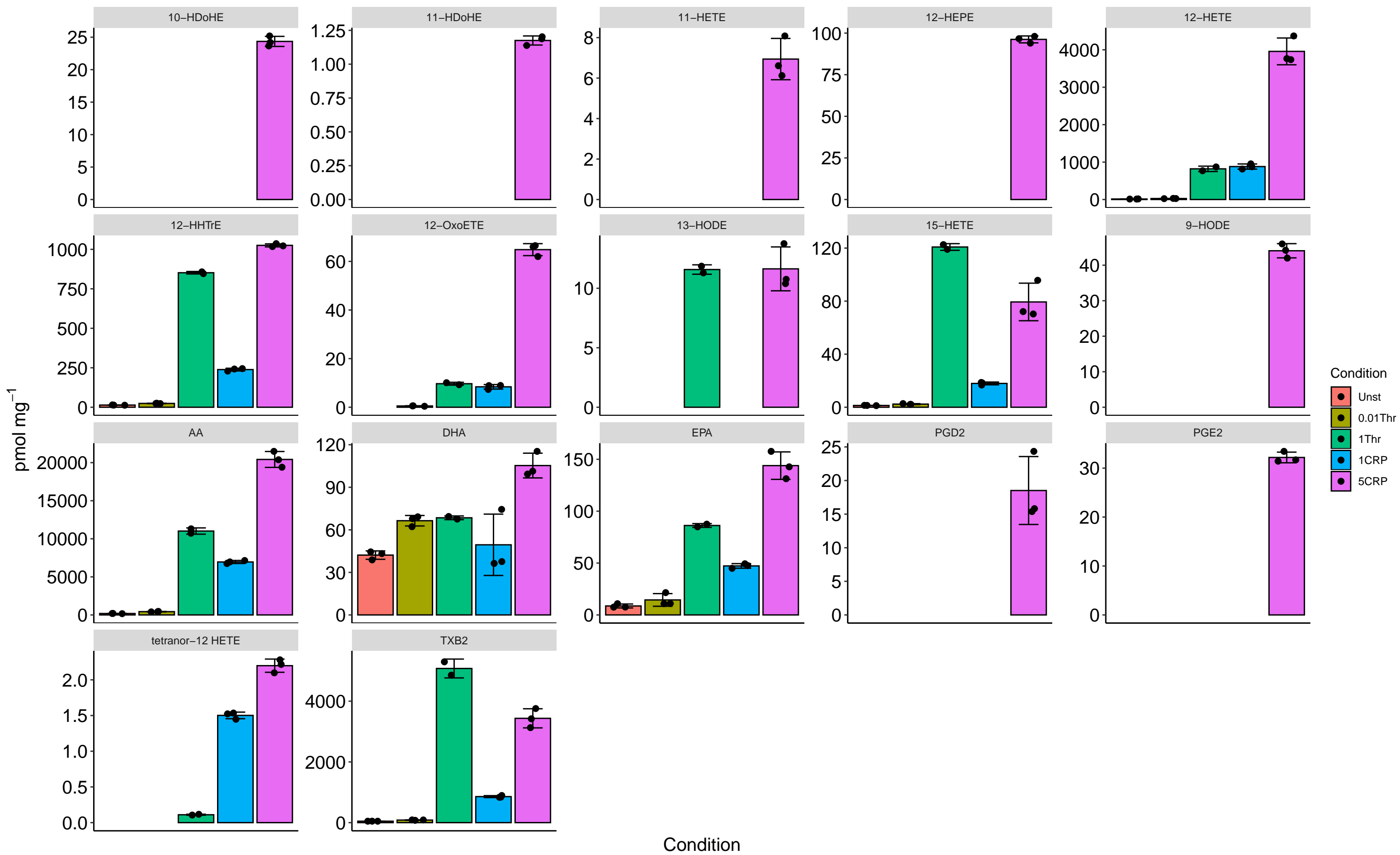

Supplement: Supplementary file 10 — Supplementary Data 7 [file 41467_2020_15960_MOESM10_ESM.zip › Scripts/Figure 7f - Lipid Mediators/figure7-f-supernatant.pdf]

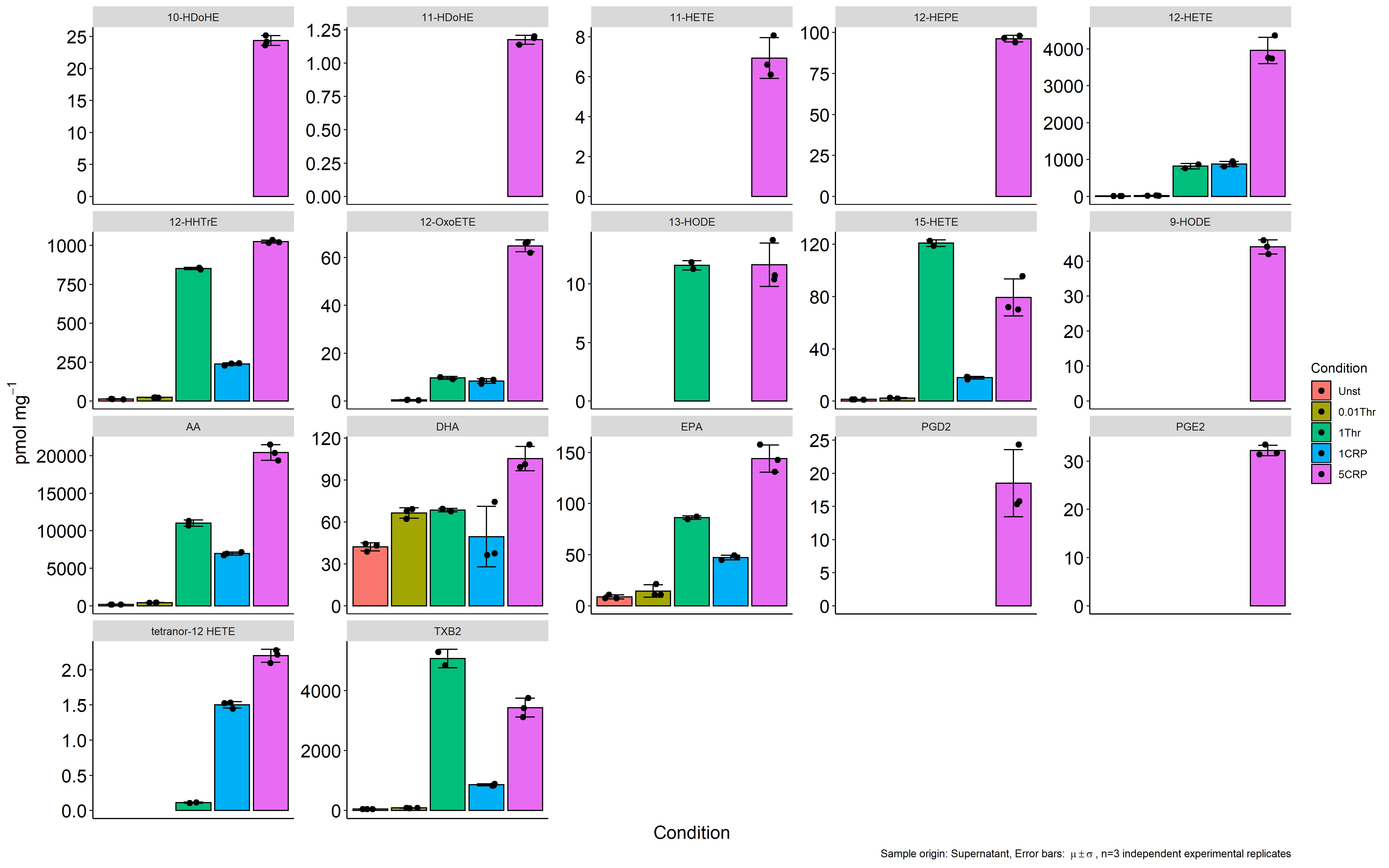

Supplement: Supplementary file 10 — Supplementary Data 7 [file 41467_2020_15960_MOESM10_ESM.zip › Scripts/Figure 7f - Lipid Mediators/figure7-f-supernatant.png]

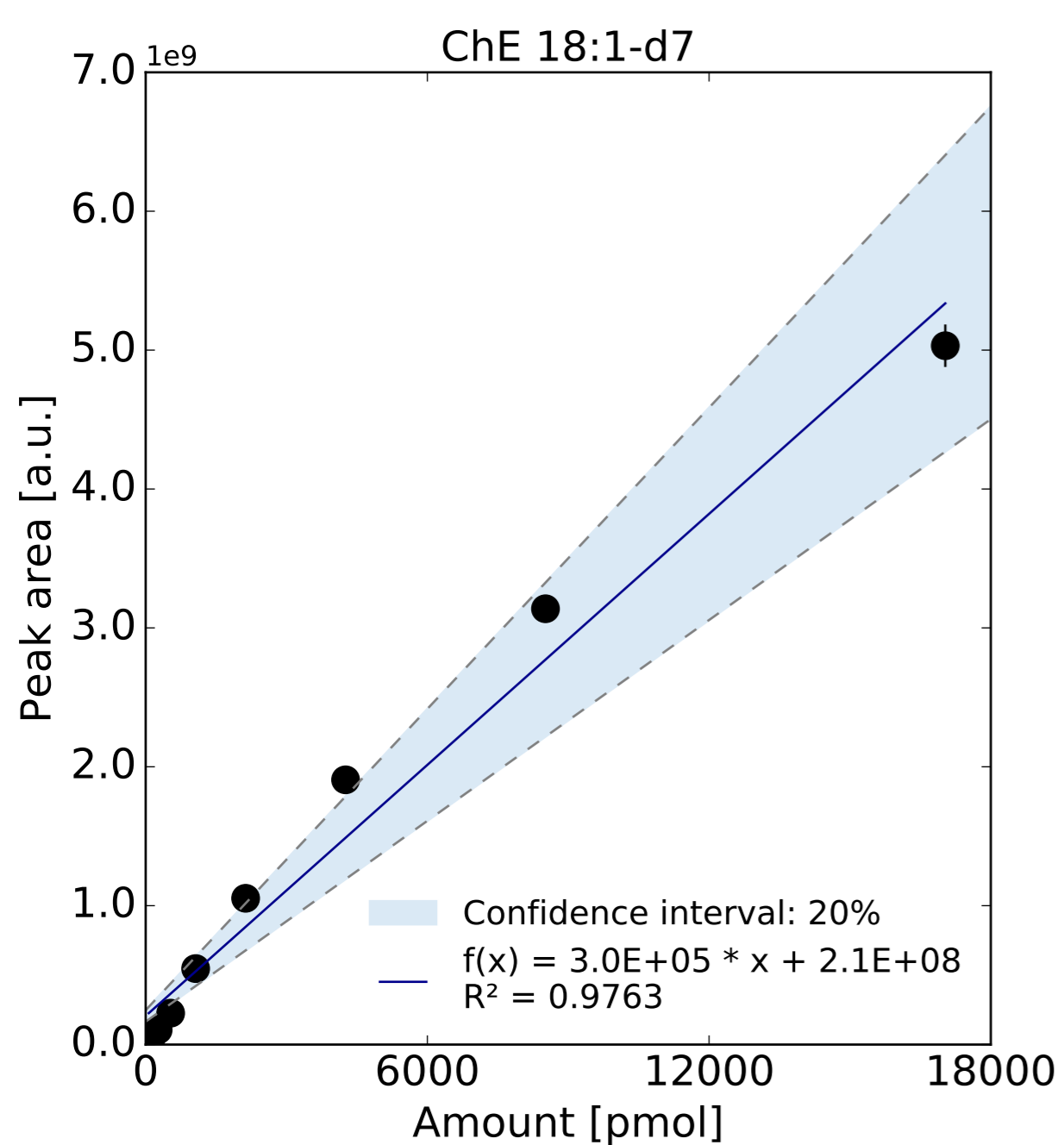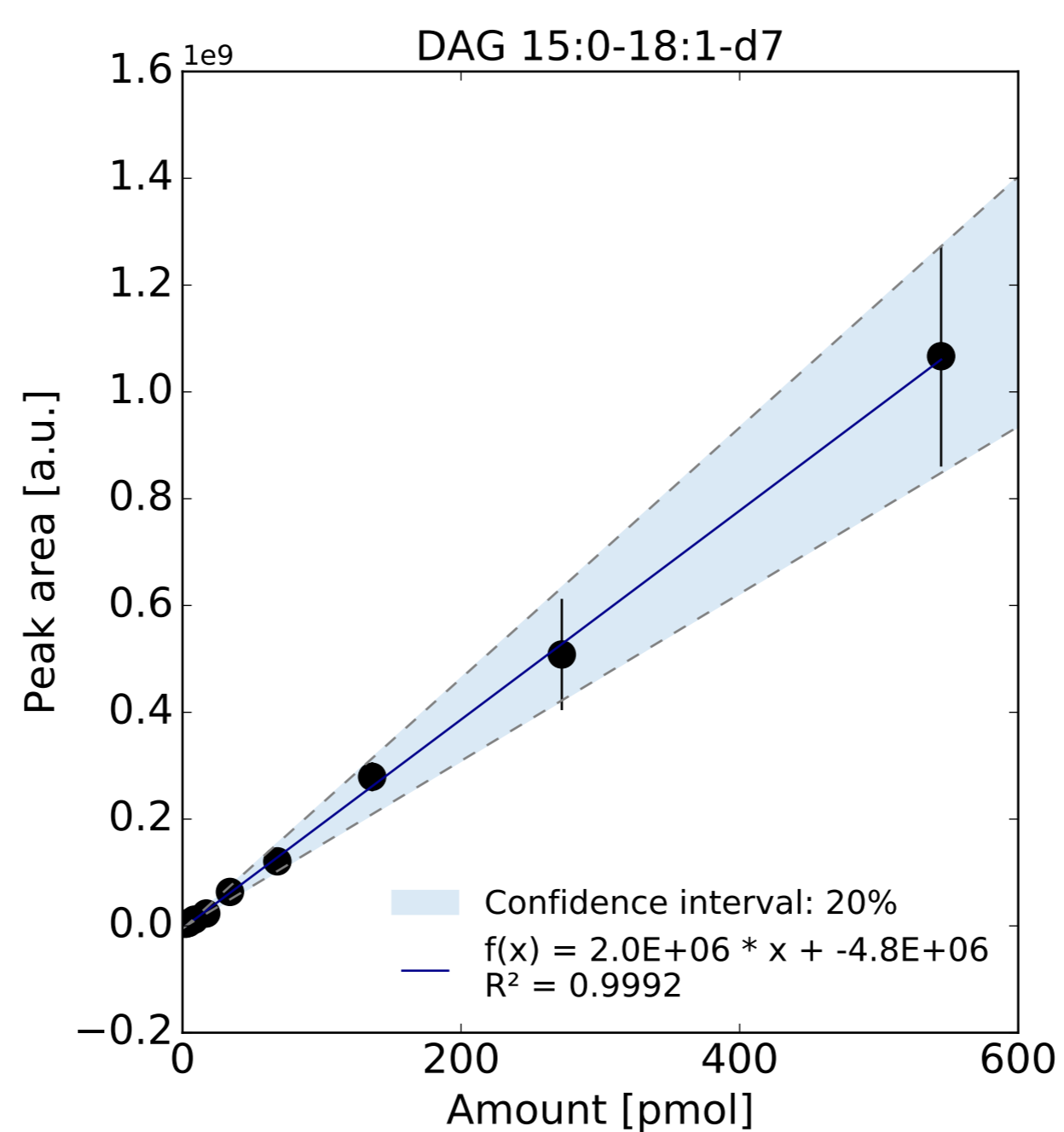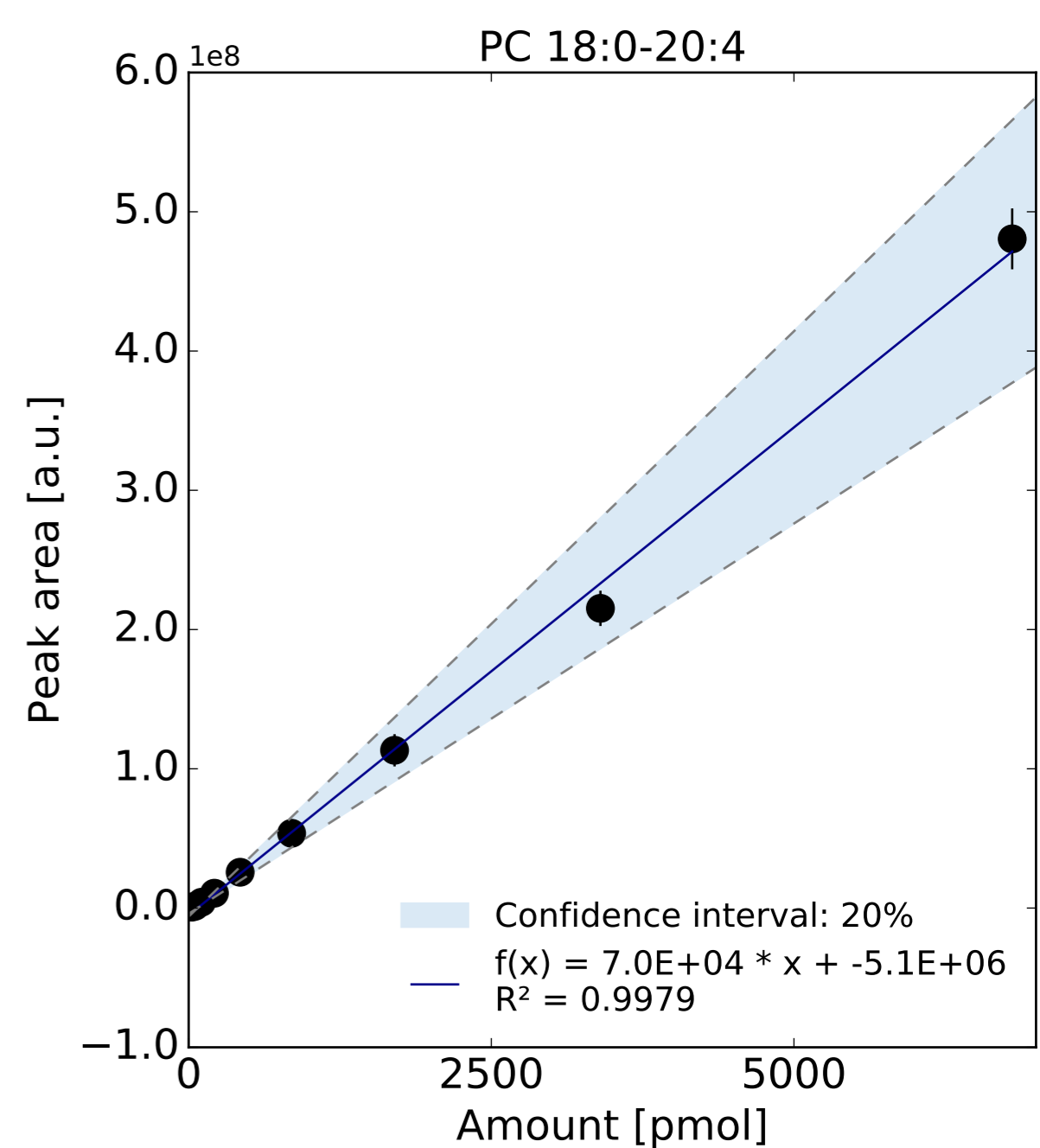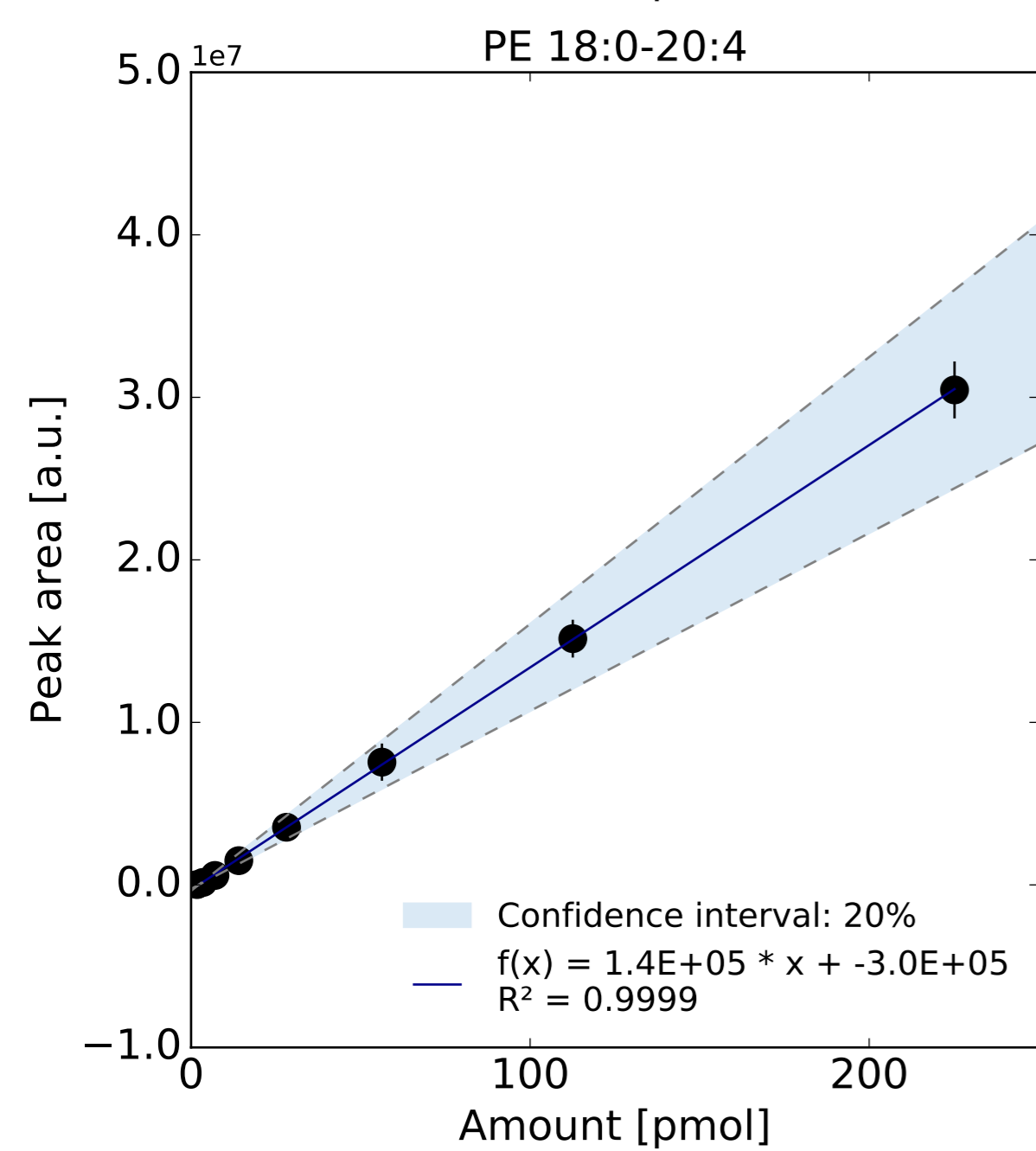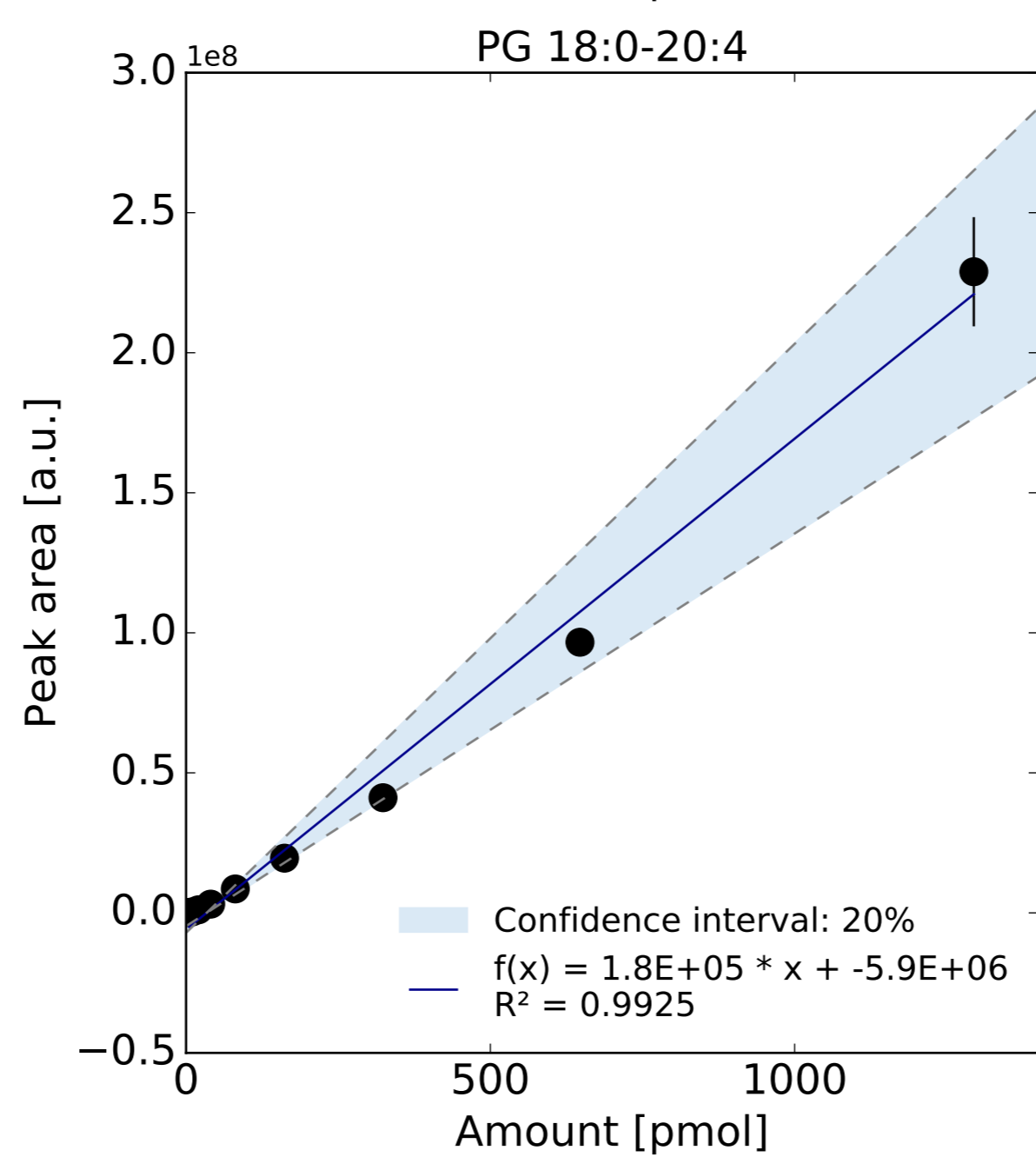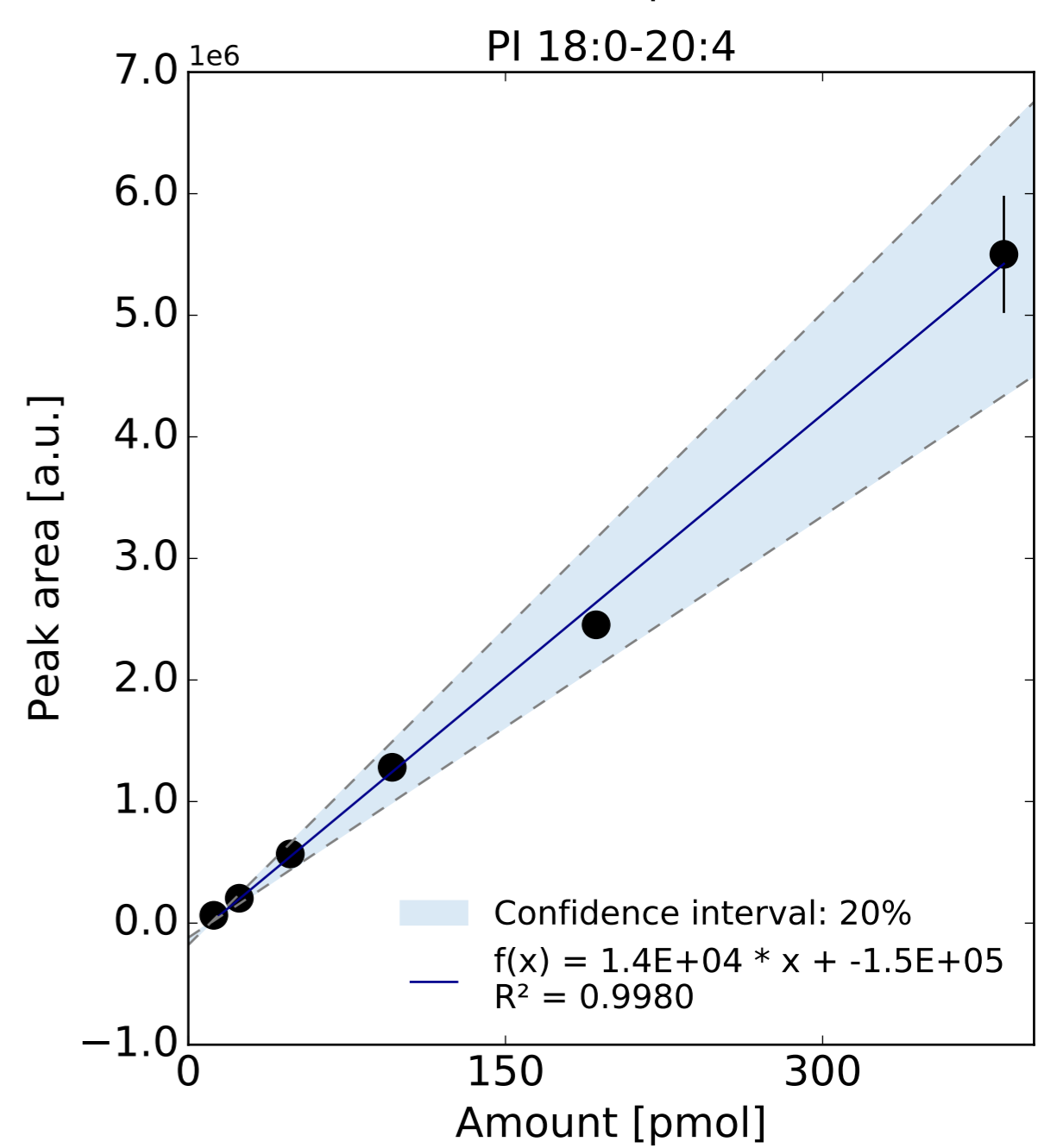

Supplement: Supplementary file 10 — Supplementary Data 7 [file 41467_2020_15960_MOESM10_ESM.zip › Scripts/Supplementary Figure 2 - Calibration curves/Calibration-curves.pdf]
