# Supplementary material for: LipidCreator workbench to probe the lipidomic landscape
Source: Nat Commun. 2020 Apr 28;11:2057. doi: 10.1038/s41467-020-15960-z (PMC7188904; doi:10.1038/s41467-020-15960-z)
Supplement: Supplementary file 12 — Reporting Summary [file 41467_2020_15960_MOESM12_ESM.pdf]

natureresearch

Corresponding author(s): Robert Ahrends

Last updated by author(s): 25/3/20

Reporting Summary

Nature Research wishes to improve the reproducibility of the work that we publish. This form provides structure for consistency and transparency in reporting. For further information on Nature Research policies, see Authors & Referees and the Editorial Policy Checklist .

Statistics

For all statistical analyses, confirm that the following items are present in the figure legend, table legend, main text, or Methods section.

n/a	Confirmed	
[ ]	[x]	The exact sample size (*n*) for each experimental group/condition, given as a discrete number and unit of measurement
[ ]	[x]	A statement on whether measurements were taken from distinct samples or whether the same sample was measured repeatedly
[ ]	[x]	The statistical test(s) used AND whether they are one- or two-sided<br>*Only common tests should be described solely by name; describe more complex techniques in the Methods section.*
[x]	[ ]	A description of all covariates tested
[ ]	[x]	A description of any assumptions or corrections, such as tests of normality and adjustment for multiple comparisons
[x]	[ ]	A full description of the statistical parameters including central tendency (e.g. means) or other basic estimates (e.g. regression coefficient) AND variation (e.g. standard deviation) or associated estimates of uncertainty (e.g. confidence intervals)
[x]	[ ]	For null hypothesis testing, the test statistic (e.g. *F*, *t*, *r*) with confidence intervals, effect sizes, degrees of freedom and *P* value noted<br>*Give P values as exact values whenever suitable.*
[x]	[ ]	For Bayesian analysis, information on the choice of priors and Markov chain Monte Carlo settings
[x]	[ ]	For hierarchical and complex designs, identification of the appropriate level for tests and full reporting of outcomes
[x]	[ ]	Estimates of effect sizes (e.g. Cohen's *d*, Pearson's *r*), indicating how they were calculated

Our web collection on statistics for biologists contains articles on many of the points above.

Software and code

Policy information about availability of computer code

Data collection	Lipid standard samples for the CE-dependent relative intensity prediction model training were acquired using Chipsoft 8.3.1 (Advion Biosciences) for the nanoflow ion source TriVersa NanoMate, Agilent MassHunter 8.0 for the QTOF platform and with Thermo Xcalibur 2.8-280502/2.8.1.2806 for the QExactive HF platform. Vendor specific MS measurement files were converted with msConvert / ProteoWizard 3.0.11537 to mzML with vendor peak-picking and MS1+MS2 scan inclusion. The R packages minpack.lm 1.2-1 and nls.multstart 1.0.0 were used to establish the collision energy model.
Data analysis	LipidCreator 1.1.0 was used for creating transition lists for yeast, plasma and human platelets for the application.<br><br>Skyline 4.2 and 19.01 were used to search and quantify the targeted lipids for the application.<br><br>In-house developed R script 'flipR' was utilized in version 1.0.6 on R 3.6 to compute parameters for the statistical models describing collision energy for lipid species. Previously converted mzML files and transition lists created with LipidCreator were used to create input tables for 'flipR' for each lipid measurement, transition and collision energy, based on custom JAVA code using MSDK 0.0.12.

For manuscripts utilizing custom algorithms or software that are central to the research but not yet described in published literature, software must be made available to editors/reviewers. We strongly encourage code deposition in a community repository (e.g. GitHub). See the Nature Research guidelines for submitting code & software for further information.

Data

Policy information about availability of data

All manuscripts must include a data availability statement. This statement should provide the following information, where applicable:

- Accession codes, unique identifiers, or web links for publicly available datasets
- A list of figures that have associated raw data
- A description of any restrictions on data availability

All raw files and processed data tables are available from public repositories. Skyline projects for the human platelet activation measurements are available from the Panorama repository. Raw MS data, mzML converted data, transition lists, picked and integrated peak areas from exported from Skyline and the final, quantified lipid result tables are available from MetaboLights. More detailed description of the dataset generation is provided in the Supplementary Information.
Panorama:
Human platelet data: [https://panoramaweb.org/lipidcreator.url].

MetaboLights:
Yeast data: MTBLS1376 [https://www.ebi.ac.uk/metabolights/MTBLS1376]
Targeted analysis of human plasma samples: MTBLS1375 [https://www.ebi.ac.uk/metabolights/MTBLS1375]
Human platelet data: Targeted LC-MS/MS analysis of phospholipids, glycerolipids and sphingolipids: MTBLS1369 [https://www.ebi.ac.uk/metabolights/MTBLS1369]
Targeted analysis of mediators: MTBLS1381 [https://www.ebi.ac.uk/metabolights/MTBLS1381]
DIA validation: MTBLS1382 [https://www.ebi.ac.uk/metabolights/MTBLS1382]
Training Data for CE optimization model training of lipid mediators:
QExactive HF Platform: MTBLS1333 [https://www.ebi.ac.uk/metabolights/MTBLS1333]
QTOF Platform: MTBLS1334 [https://www.ebi.ac.uk/metabolights/MTBLS1334]

MassBank.eu:
Averaged CE spectra of lipid mediator standards measured on the Thermo QExactive HF and Agilent QTOF platforms are available from MassBank:
[https://massbank.eu/MassBank/Result.jsp?type=rcdidx&idxtype=site&srchkey=ISAS_Dortmund].
Scripts for figures:
The scripts underlying Figures 4, 5, 6, 7 and Supplementary Figure 2 are provided as Supplementary-Data-7.zip
Code availability:
The source code of LipidCreator is available at [https://github.com/lifs-tools/lipidcreator]. The source code of flipR, the training harness and the code required to recreate Supplementary Data 5 and 6 are available from [https://github.com/lifs-tools/flipr].
Software availability:
A binary compiled version of LipidCreator is available as Supplementary Software 1.zip.
Releases of LipidCreator are available at [https://lifs.isas.de/lipidcreator] and at Zenodo: [https://doi.org/10.5281/zenodo.3529484].
Source Data:
The source data underlying Figure 4, 5, 6, 7 and Supplementary Figure 2 are provided as a Source Data file.

Field-specific reporting

Please select the one below that is the best fit for your research. If you are not sure, read the appropriate sections before making your selection.

[x] Life sciences [ ] Behavioural & social sciences [ ] Ecological, evolutionary & environmental sciences

For a reference copy of the document with all sections, see nature.com/documents/nr-reporting-summary-flat.pdf

Life sciences study design

All studies must disclose on these points even when the disclosure is negative.

Sample size	We used 5 platelet samples derived from healthy humans (pellet and supernatant) and 21 plasma samples derived from healthy humans. Sample sizes were chosen to be able to determine the specificity of the lipidomics assays concerning identification and quantification. No specific sample size calculation was applied. Experiments were performed independently for at least 5 times (platelets) and 21 times (plasma).
Data exclusions	No data was excluded from the analysis.
Replication	The platelet and plasma samples were only collected and processed once. For the lipidomics analysis, the full set of samples (platelet lipid extracts) was measured three times. The full set of samples (plasma lipid extracts) was measured twice, whereby each extract in each analysis was analyzed in duplicate, resulting in a total of four measurements per extract of plasma sample. All attempts at replications were successful.
Randomization	Groups and samples were allocated in random order to eleminate time dependent effects on sample preparation (platelet). Samples were stratified/randomized for gender and age during sample preparation and lipidomics analysis (plasma).
Blinding	Sample preparation, measurement and data analysis were blinded. No individual patient data was shared.

Reporting for specific materials, systems and methods

We require information from authors about some types of materials, experimental systems and methods used in many studies. Here, indicate whether each material, system or method listed is relevant to your study. If you are not sure if a list item applies to your research, read the appropriate section before selecting a response.

Materials & experimental systems

n/a	Involved in the study
☒	☐ Antibodies
☒	☐ Eukaryotic cell lines
☒	☐ Palaeontology
☒	☐ Animals and other organisms
☐	☒ Human research participants
☒	☐ Clinical data

Methods

n/a	Involved in the study
☒	☐ ChIP-seq
☒	☐ Flow cytometry
☒	☐ MRI-based neuroimaging

Human research participants

Policy information about studies involving human research participants

Population characteristics	Platelets: 5 healthy volunteers (males, 20 - 30 years old) from Germany.<br>Plasma: 21 healthy individuals (12 males, 9 females; 22 - 44 years old) from Singapore (Chinese, Malay and Caucasian).
Recruitment	Volunteers were recruited among the employees of the clinic. This study focuses on methodological aspects, and even if there is an unintended selection bias, we do not expect this to impact the findings and conclusions of this publication.
Ethics oversight	All volunteers gave informed consent for blood samples. The platelet study was approved by the institutional ethics committee (270/2011BO1) at University Hospital Tübingen (Germany) and complies with the declaration of Helsinki and good clinical practice guidelines.<br>The collection and use of human plasma samples has been approved by the Institutional Review Board of the National University of Singapore (NUS-IRB N-17-082E and NUS-IRB B-15-094, Singapore).

Note that full information on the approval of the study protocol must also be provided in the manuscript.
