# Supplementary material for: LipidCreator workbench to probe the lipidomic landscape
Source: Nat Commun. 2020 Apr 28;11:2057. doi: 10.1038/s41467-020-15960-z (PMC7188904; doi:10.1038/s41467-020-15960-z)
Supplement: Supplementary file 11 — Supplementary Software [file 41467_2020_15960_MOESM11_ESM.zip › LipidCreator.zip!/data/goslin/html/Goslin.html]

### SPACE

' '

### COLON

':'

### SEMICOLON

';'

### DASH

'-'

### UNDERSCORE

'\_'

### SLASH

'/'

### BACKSLASH

'\\'

### COMMA

','

### ROB

'('

### RCB

')'

### lipid

lipid\_purelipid\_pureadduct\_info

### lipid\_pure

glplslcholesterolmediatorc

### adduct\_info

'[M'adduct']'chargecharge\_signadduct\_separator'[M'adduct']'chargecharge\_sign

### adduct

'+H''+2H''+NH4''-H''-2H''+HCOO''+CH3COO'adduct4adduct4adduct4

### adduct4

adduct2adduct2adduct2

### adduct2

charactercharactercharacter

### character

'a''b''c''d''e''f''g''h''i''j''k''l''m''n''o''p''q''r''s''t''u''v''w''x''y''z''A''B''C''D''E''F''G''H''I''J''K''L''M''N''O''P''Q''R''S''T''U''V''W''X''Y''Z''0''1''2''3''4''5''6''7''8''9''+''-'

### charge

'1''2''3''4'

### charge\_sign

'-''+'

### gl

mgldglsgltgl

### mgl

hg\_mglheadgroup\_separatorfa

### dgl

hg\_dglheadgroup\_separatorfaunsorted\_fa\_separatorfahg\_dglheadgroup\_separatorfasorted\_fa\_separatorfa

### sgl

hg\_sglheadgroup\_separatorfaunsorted\_fa\_separatorfahg\_sglheadgroup\_separatorfasorted\_fa\_separatorfa

### tgl

hg\_tglheadgroup\_separatorfaunsorted\_fa\_separatorfaunsorted\_fa\_separatorfahg\_tglheadgroup\_separatorfasorted\_fa\_separatorfasorted\_fa\_separatorfa

### hg\_mgl

'MAG'

### hg\_dgl

'DAG'

### hg\_sgl

'MGDG''DGDG''SQDG'

### hg\_tgl

'TAG'

### pl

lpldplpl\_oclmlcl

### pl\_o

lpl\_odpl\_o

### lpl

hg\_lplcheadgroup\_separatorfa

### lpl\_o

hg\_lpl\_ocheadgroup\_separatorfa

### dpl

hg\_plcheadgroup\_separatorfaunsorted\_fa\_separatorfahg\_plcheadgroup\_separatorfasorted\_fa\_separatorfa

### dpl\_o

hg\_pl\_ocheadgroup\_separatorfaunsorted\_fa\_separatorfahg\_pl\_ocheadgroup\_separatorfasorted\_fa\_separatorfa

### cl

hg\_clcheadgroup\_separatorfaunsorted\_fa\_separatorfaunsorted\_fa\_separatorfaunsorted\_fa\_separatorfahg\_clcheadgroup\_separatorfasorted\_fa\_separatorfasorted\_fa\_separatorfasorted\_fa\_separatorfa

### mlcl

hg\_mlclcheadgroup\_separatorfaunsorted\_fa\_separatorfaunsorted\_fa\_separatorfahg\_mlclcheadgroup\_separatorfasorted\_fa\_separatorfasorted\_fa\_separatorfa

### hg\_clc

hg\_clhg\_clheavy

### hg\_cl

'CL'

### hg\_mlclc

hg\_mlclhg\_mlclheavy

### hg\_mlcl

'MLCL'

### hg\_plc

hg\_plhg\_plheavy

### hg\_pl

'BMP''CDPDAG''DMPE''MMPE''PA''PC''PE''PEt''PG''PI''PIP''PIP2''PIP3''PS'

### hg\_lplc

hg\_lplhg\_lplheavy

### hg\_lpl

'LPA''LPC''LPE''LPG''LPI''LPS'

### hg\_lpl\_oc

hg\_lpl\_ohg\_lpl\_oheavy

### hg\_lpl\_o

'LPC O''LPE O'

### hg\_pl\_oc

hg\_pl\_ohg\_pl\_oheavy

### hg\_pl\_o

'PC O''PE O'

### sl

lsldsl

### lsl

hg\_lslcheadgroup\_separatorlcb

### dsl

hg\_dslcheadgroup\_separatorlcbsorted\_fa\_separatorfa

### hg\_lslc

hg\_lslhg\_lslheavy

### hg\_lsl

'lcb''lcbP''LHexCer''LSM'

### hg\_dslc

hg\_dslhg\_dslheavy

### hg\_dsl

'Cer''CerP''EPC''GB3''GB4''GD3''GM3''GM4''Hex2Cer''HexCer''IPC''M(IP)2C''MIPC''SHexCer''SM'

### cholesterol

chcche

### chc

chchheavy

### ch

'Ch'

### che

hg\_checheadgroup\_separatorfa

### hg\_chec

hg\_chehg\_cheheavy

### hg\_che

'ChE'

### mediatorc

mediatormediatorheavy

### mediator

'10-HDoHE''11-HDoHE''11-HETE''11,12-DHET''11(12)-EET''12-HEPE''12-HETE''12-HHTrE''12-OxoETE''12(13)-EpOME''13-HODE''13-HOTrE''14,15-DHET''14(15)-EET''14(15)-EpETE''15-HEPE''15-HETE''15d-PGJ2''16-HDoHE''16-HETE''18-HEPE''5-HEPE''5-HETE''5-HpETE''5-OxoETE''5,12-DiHETE''5,6-DiHETE''5,6,15-LXA4''5(6)-EET''8-HDoHE''8-HETE''8,9-DHET''8(9)-EET''9-HEPE''9-HETE''9-HODE''9-HOTrE''9(10)-EpOME''AA''alpha-LA''DHA''EPA''Linoleic acid''LTB4''LTC4''LTD4''Maresin 1''Palmitic acid''PGB2''PGD2''PGE2''PGF2alpha''PGI2''Resolvin D1''Resolvin D2''Resolvin D3''Resolvin D5''tetranor-12-HETE''TXB1''TXB2''TXB3'

### fa

fa\_purefa\_pureether

### fa\_pure

carboncarbon\_db\_separatordbcarboncarbon\_db\_separatordbdb\_hydroxyl\_separatorhydroxylcarboncarbon\_db\_separatordbheavycarboncarbon\_db\_separatordbdb\_hydroxyl\_separatorhydroxylheavy

### ether

'a''p'

### lcb

carboncarbon\_db\_separatordbdb\_hydroxyl\_separatorhydroxylold\_hydroxylcarboncarbon\_db\_separatordb

### carbon

number

### db

db\_countdb\_countround\_open\_bracketdb\_positionround\_close\_bracket

### db\_count

number

### db\_position

numbercistransnumbercistransdb\_positiondb\_position\_separatordb\_position

### cistrans

'E''Z'

### hydroxyl

number

### old\_hydroxyl

'd''t'

### number

'0''1''2''3''4''5''6''7''8''9'numbernumber

### heavy

'(+'isotopes')'

### isotopes

isotopesisotopes'['number']'elementnumber

### element

'C''H''O''N''P''S'

### sorted\_fa\_separator

SLASHBACKSLASH

### adduct\_separator

SPACE

### unsorted\_fa\_separator

DASHUNDERSCORE

### headgroup\_separator

SPACE

### carbon\_db\_separator

COLON

### db\_hydroxyl\_separator

SEMICOLON

### db\_position\_separator

COMMA

### round\_open\_bracket

ROB

### round\_close\_bracket

RCB
