# Supplementary material for: LipidCreator workbench to probe the lipidomic landscape
Source: Nat Commun. 2020 Apr 28;11:2057. doi: 10.1038/s41467-020-15960-z (PMC7188904; doi:10.1038/s41467-020-15960-z)
Supplement: Supplementary file 11 — Supplementary Software [file 41467_2020_15960_MOESM11_ESM.zip › LipidCreator.zip!/data/docs/LipidCreator_Manual_v18.pdf]

LipidCreator (v1.1.0.x)

Manual (v18), Mar. 27th, 2020

Contributors: Bing Peng, Dominik Kopzcyinski, Nils Hoffmann

T1. An Overview of the LipidCreator Workflow 2
T2. Using the Wizard 3
T3. Generating lipid molecule transitions 7
T4. Import/export lipid list/setting/project 18
T5. Lipid name translator 19
T6. Selection of MS2 fragments 20
T7. Adding user-defined fragments 23
T8. Managing heavy isotopes 27
T9. Filters for the transition list 30
T10. Collision energy optimization function 31
T11. Reviewing the lipid transition list 33
T12. Storing a transition list 37
T13. Storing a spectral library 37
T14. Integration with Skyline 37
T15. Statistics for LipidCreator launching 39
T16. Command line usage 40
T17. Support for additional platforms for CE Optimization 43

This manual provides general information on the usage of LipidCreator. It explains all functionalities covered by LipidCreator's wizard (See Using the Wizard), the four interactive tutorials and provides additional information for more advanced use-cases. To start a tutorial, the user has to click on one of the four tutorial buttons in the "Home" tab as illustrated in Figure T1. Home tab of LipidCreator. The following tutorials are currently provided: i) setting up a PRM or ii) SRM analysis, iii) creating heavy isotope labeled lipids and iv) adding optimized collision energies for mediator analysis. These tutorials guide the user through the user interface of LipidCreator and explain step by step all important information relevant for the understanding of the four workflows.

If you spot any issues or require help with LipidCreator, please contact us at: https://lifs.isas.de/support.html

T1. An Overview of the LipidCreator Workflow

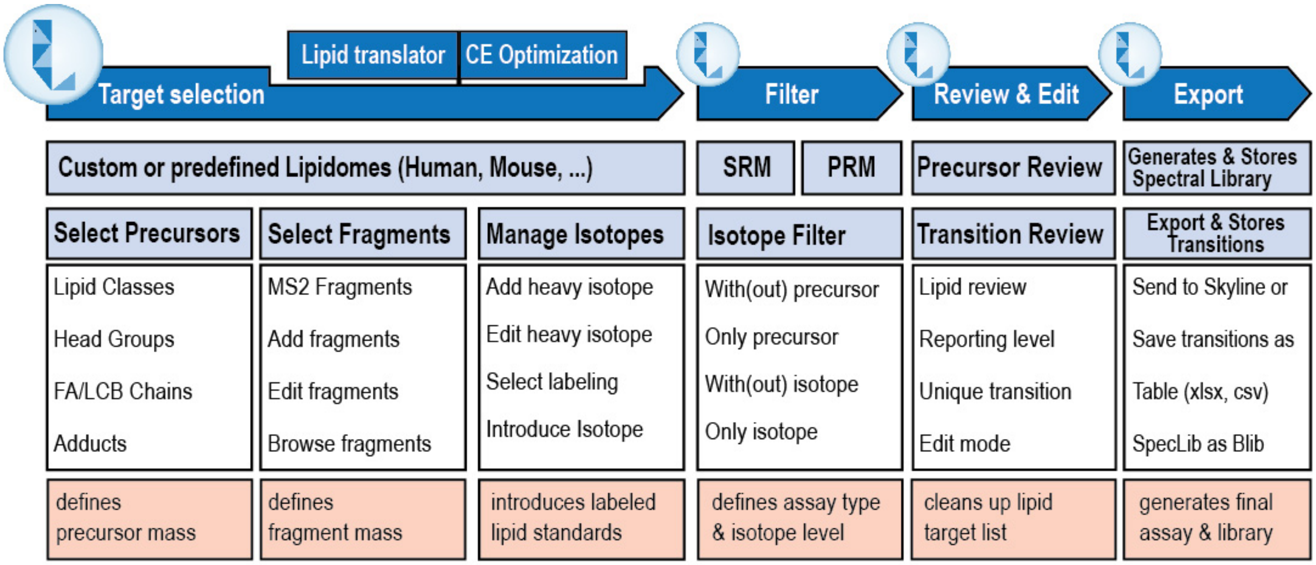

Figure T1. LipidCreator Workflow.
The LipidCreator workflow consists of four main steps to generate SRM or PRM transition lists that can either be transferred to Skyline or exported for use in other software for targeted mass spectrometry method development, refinement and analysis.
These steps are:

1. Target selection – define, which lipid classes and species, which fragments and which labels, if any, should be included in the transition lists.
2. Filter – select, whether precursor and / or fragments should be included in the transition list and whether unlabeled, labeled or both combinations should be included in the transition list, where applicable.
3. Review & Edit – select, edit, or refine the lipid transitions, define the reporting level, e.g. lipid species or subspecies level, check and warn about potentially interfering transitions (with the same m/z).
4. Export – transfer the reviewed transitions directly to Skyline, or store them in tabular format. If CE Optimization has been enabled, optionally transfer an *in-silico* spectral library directly to Skyline, or store it in Bibliospec (BLIB) format.

T2. Using the Wizard

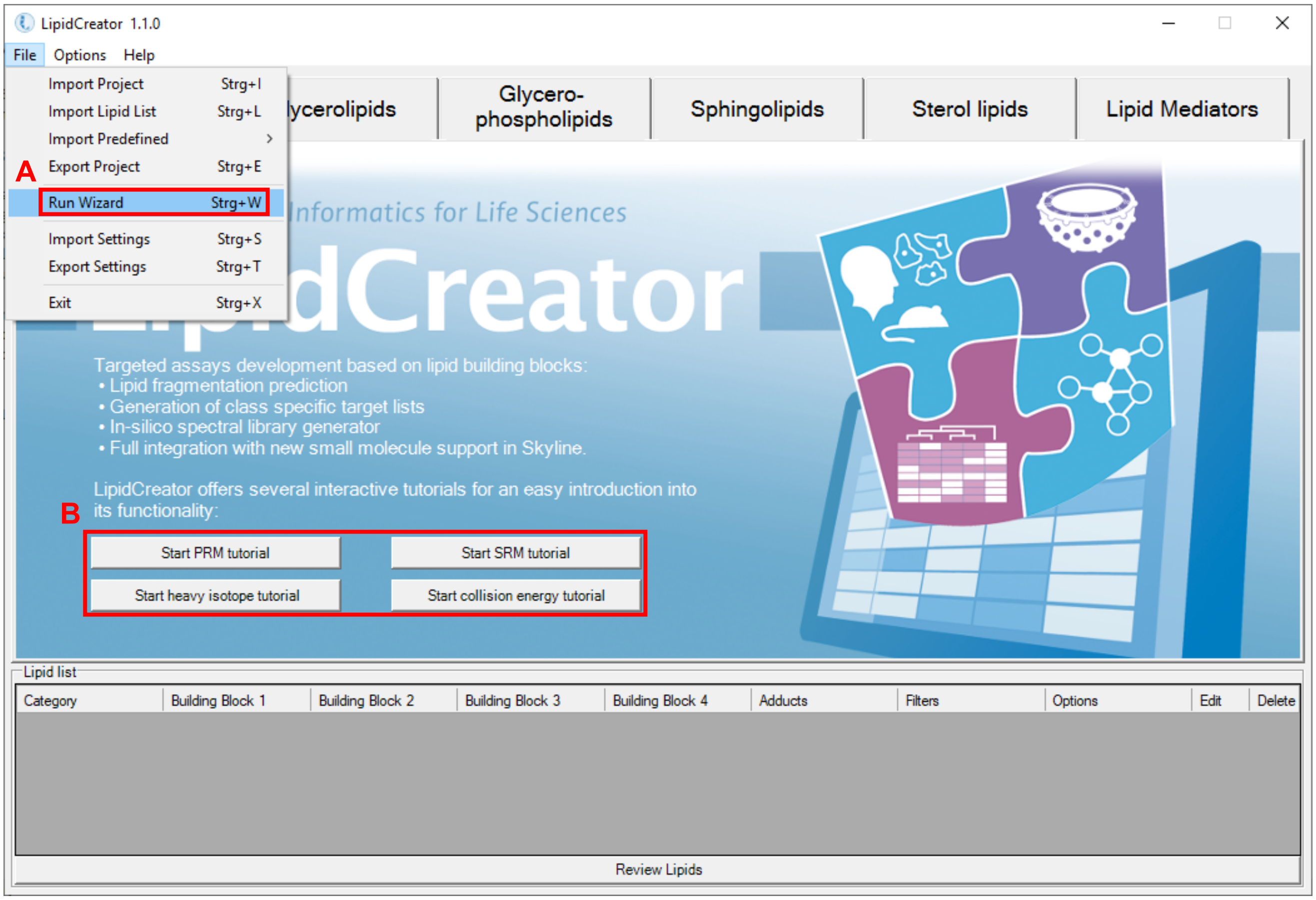

Figure W1. Home tab of LipidCreator.

A: The LipidCreator Wizard can be started via the **File → Run Wizard** menu entry. Please note that the wizard panels are context-dependent. Thus, not all panels will be available for every lipid species. Also, the wizard does not provide direct filters for isotope labeling. However, after the transitions have been added to the 'Lipid list', they are editable individually. See Filters for the transition list for more details.

B: Interactive tutorials that guide the user step-by-step through LipidCreator.

To learn how to exploit LipidCreator's full potential, especially for users with a more advanced background in Lipidomics, Generating lipid molecule transitions provides a step-by-step description of the LipidCreator user interface and all options.

The next pages describe the individual wizard panels in more detail.

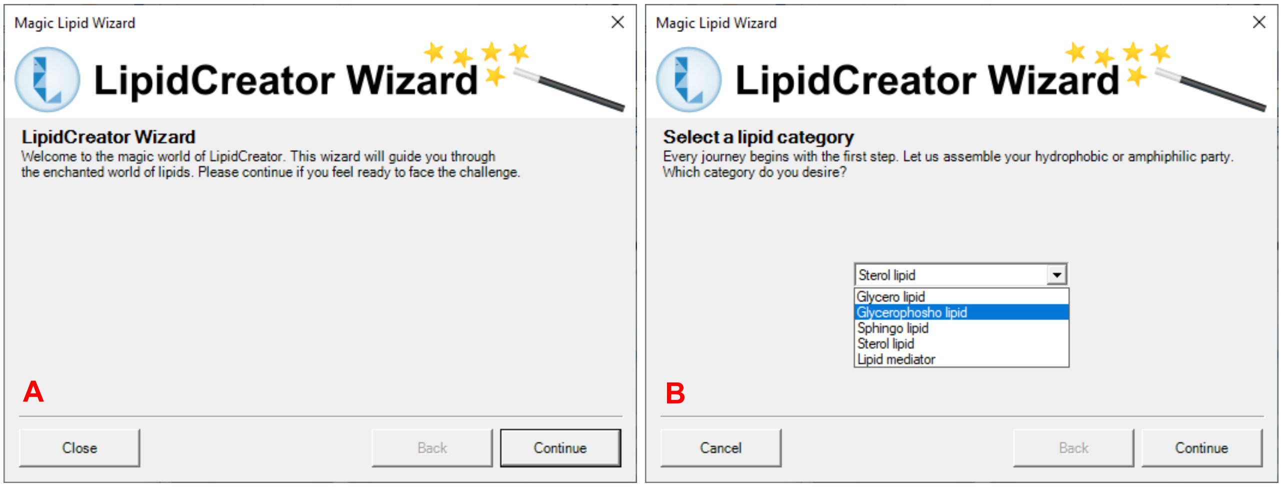

Figure W2. LipidCreator Wizard welcome screen and Lipid Category selection panel.

A: By clicking on ‘Continue’, the Wizard will transition to the ‘Lipid category’ selection panel. The user can decide to close the Wizard. In that case, no wizard data is saved! Click ‘Continue’ to go to the next panel.

B: After selection of an initial lipid category, the user can proceed to the next step ‘’. Please note that the Wizard is intentionally simple. If you want to have full flexibility, please use the regular user interface (See Generating lipid molecule transitions). Click ‘Continue’ to proceed.

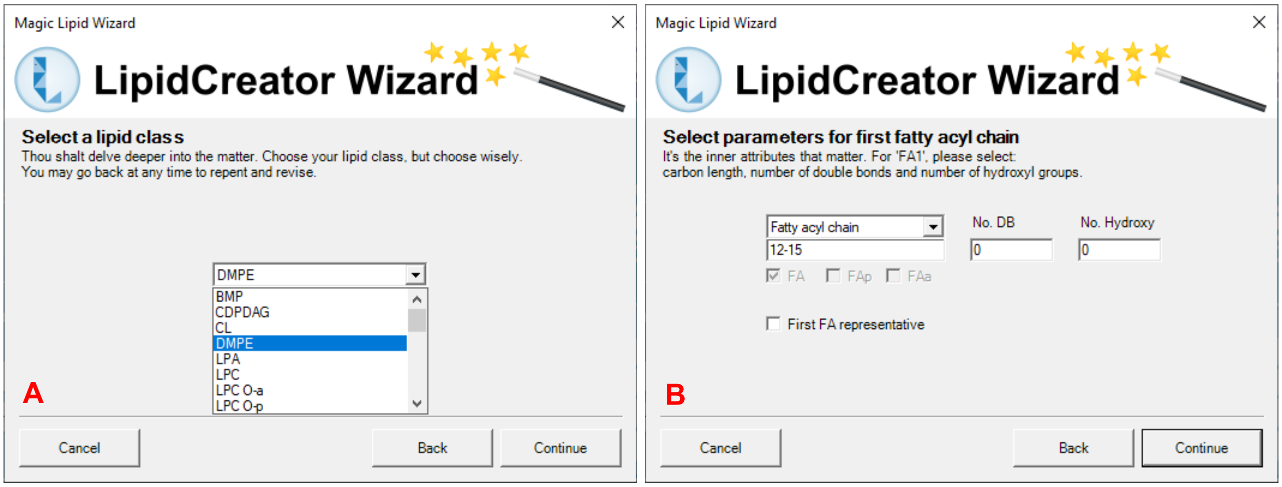

Figure W3. LipidCreator Wizard lipid class selection and first fatty acyl chain definition panels.

A: The lipid class selection shows the available lipid classes for the previously selected lipid category. Choose one and click ‘Continue’ to proceed to the FA chain definition panels.

B: The FA definition panel (depending on the lipid class, there may be multiple FAs to define) allows to set the number of carbon atoms (also as ranges), the number of double bonds and hydroxylations. Also, the bond can be set to be of type plasmenyl (FAp) or plasmanyl (FAa), depending on the lipid species. Ticking ‘First FA representative’ will copy settings for the first FA to the other FAs. Click ‘Continue’ for the next panel.

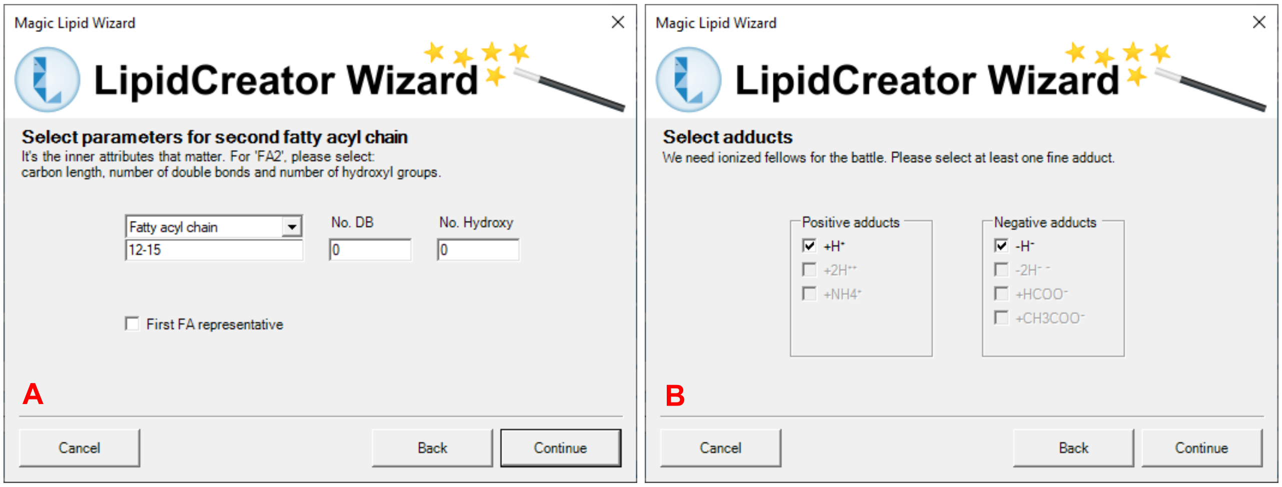

Figure W4. Second fatty acyl chain definition and adduct selection panels.

A: Some lipids may have more than one FA to define, or a long-chain-base (LCB). Thus, more than one FA panel may be part of your Wizard sequence. The definitions are basically the same, but may differ slightly based on the lipid species context. Click ‘Continue’ to go to the adduct selection panel.

B: The adduct selection panel shows potential adducts for the specific lipid species, in positive or negative mode. Any or all of the chemically feasible adducts can be selected. At least one adduct must be selected. Click on ‘Continue’ to proceed to precursor and transition filtering.

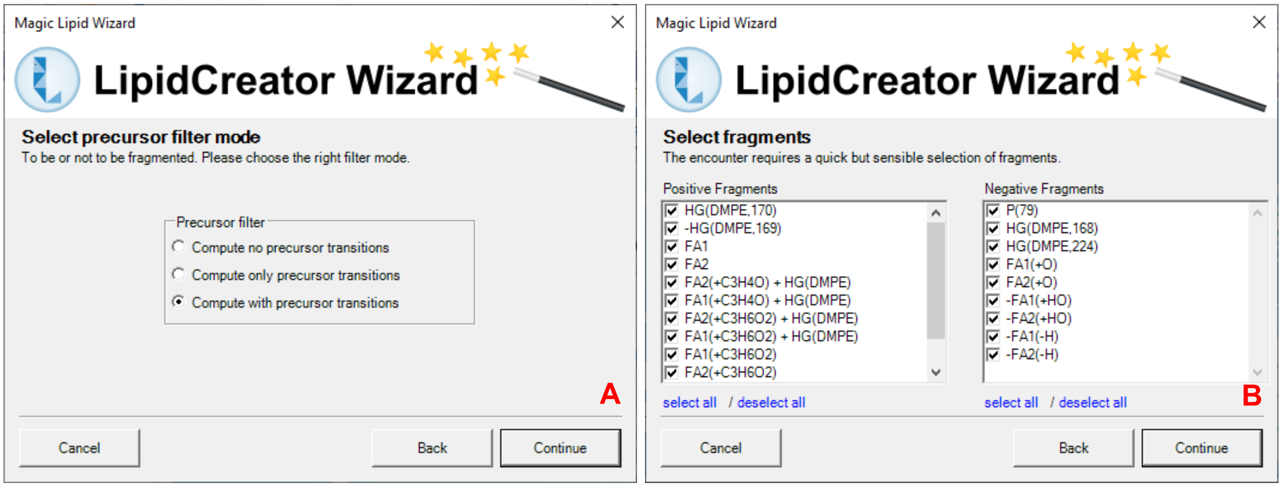

Figure W5. Precursor filter selection and MS2 fragment selection panels.

A: This panel allows to select either only MS2 fragment transitions, only the precursor transitions, or both. Click ‘Continue’ to select the MS2 fragments to include.

B: Here, the MS2 fragments (positive and negative mode, where applicable) defined in the LipidCreator knowledgebase for the currently configured lipid species are shown. Select all, or deselect undesirable ones and click ‘Continue’ to proceed to the final steps.

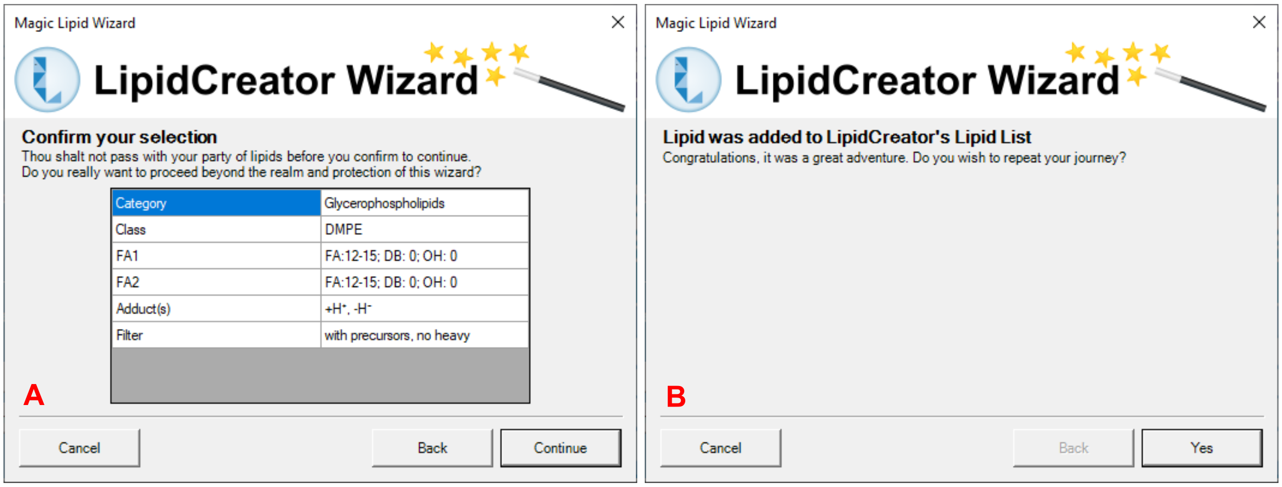

Figure W6. Selection confirmation and continuation panels.

A: After clicking on ‘Continue’, the currently configured lipid transitions are transferred to LipidCreator’s ‘Lipid list’ (See Generating lipid molecule transitions). The Wizard can be canceled at any time. In that case, no wizard data is saved!

B: After successful addition of the transitions to the ‘Lipid list’, the user can decide to either close the Wizard, or to continue from the beginning. The Wizard will then start over from the beginning.

T3. Generating lipid molecule transitions

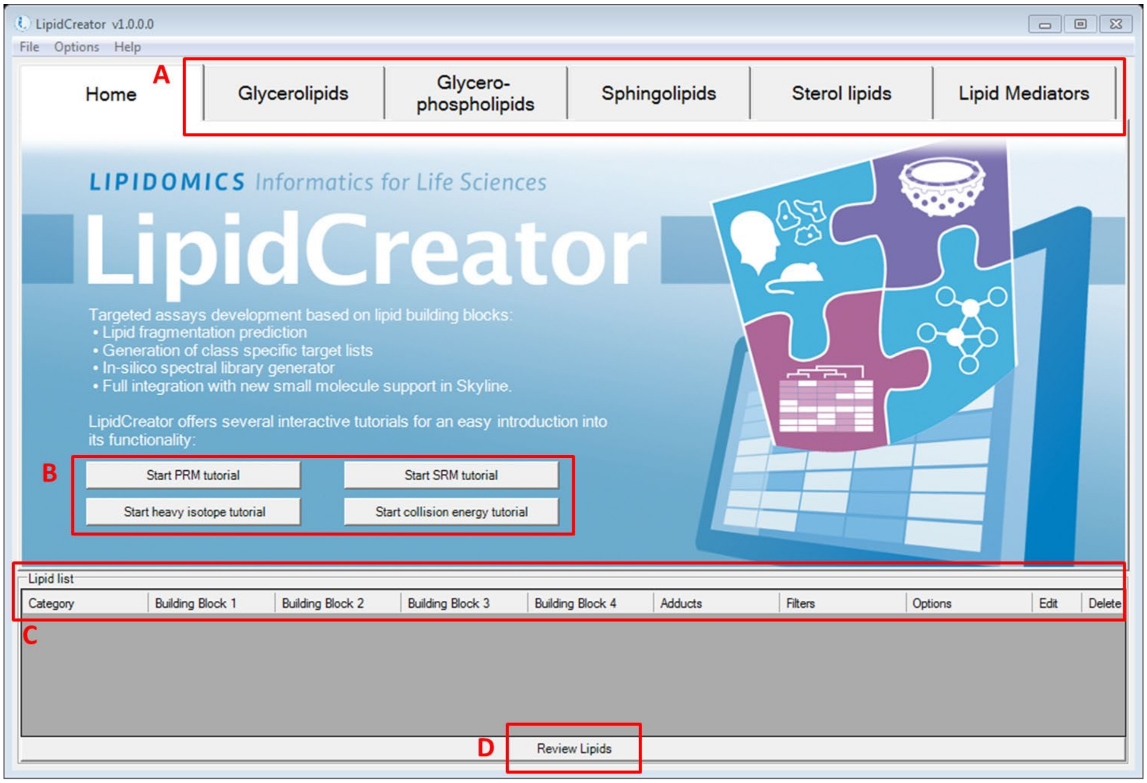

Figure T1. Home tab of LipidCreator.

The main user interface after starting LipidCreator shows the home tab, four buttons to start workflow-specific, interactive tutorials, and the ‘Lipid list’ that collects user-defined lipid specifications for the later transition list generation.

A: Tab pages for five lipid categories. Each tab provides an individual interface for a specific lipid category.

B: Interactive tutorials that guide the user step-by-step through LipidCreator.

C: ‘Lipid list’ serves as a “shopping basket” to collect lipid assemblies.

D: Launching the computation of a transition list according to the ‘Lipid list’ selection.

The next pages give an overview of the individual tabs, their functionalities and the ‘Lipid list’ that collects the used-defined lipid specifications from the different tabs.

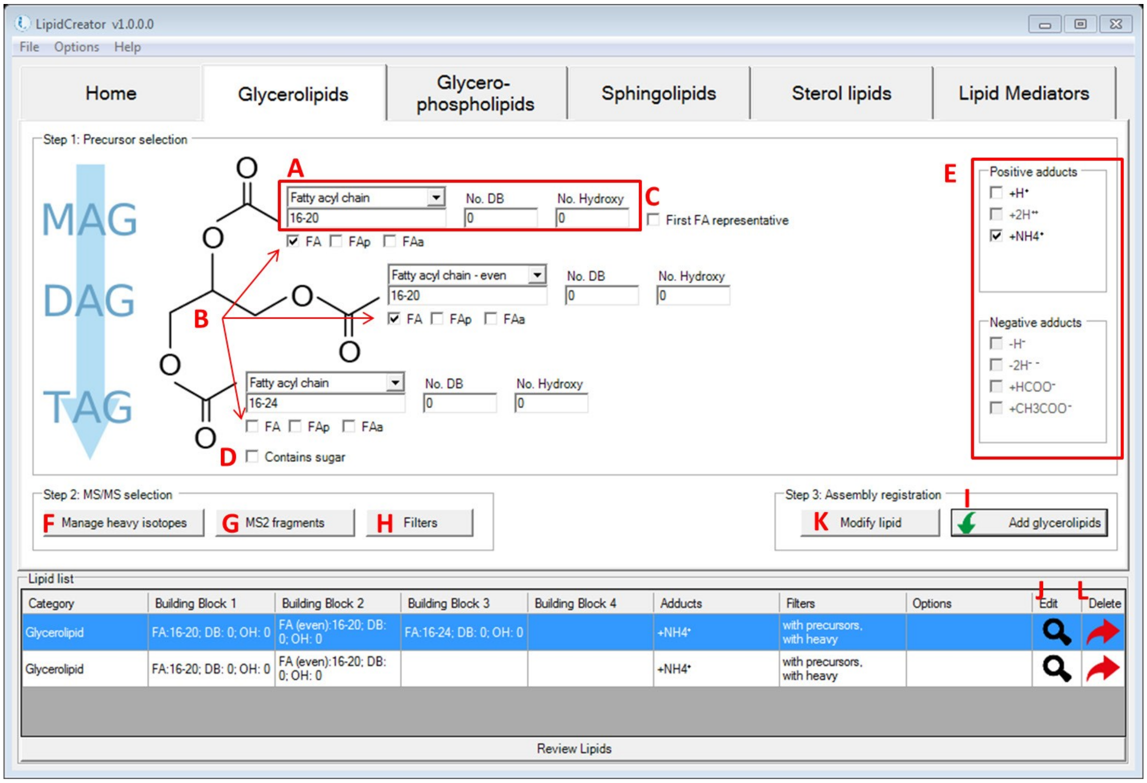

Figure T2. Interface for the definition of transitions for glycerolipids.

A: These fields let the user define one fatty acyl chain. From the drop-down list, the calculation for fatty acyl (FA) length can be chosen for even, odd or all potentially possible FA lengths. Input of either FA length, number of double bonds (DB), or number of hydroxy groups on this FA may be a range of numbers or individual values, e.g. '8, 9, 16-20, 23'. In LipidCreator, the allowed range for one FA length is 2-30, No. DB is 0-6, Hydroxy No. is 0-10 (except for sphingolipids). For sphingolipids, Hydroxy No. for long chain base (LCB) is 2 and 3, for FA, it is 0-3.

B: The check boxes for (de)selecting different types of FAs. For glycerolipids, the number of checked FAs defines the lipid class. TAG has three FAs; DAG has two FAs, whereas MAG has only one FA. FA here refers to an ester-linked fatty acid. FAp and FAa are ether-linked fatty acids. FAp has an ether bond to an alkenyl group, and FAa has an ether bond to an alkyl group, respectively. The minimum number of DBs for FAp is 1.

C: The "First FA representative" checkbox is to quickly apply FA information from (A) to all other FAs in current interface.

D: This checkbox is to replace one FA with a glucose head group (Figure T3).

E: All supported adducts in LipidCreator. For positive mode, [M+H]1+, [M+2H]2+ and [M+NH4]1+ are valid. For negative mode, [M-H]1-, [M-2H]2-, [M+HCOO]1- and [M+CH3COO]1- are valid. In LipidCreator, recommendation on adduct selection can be reviewed, when hovering the mouse cursor over different head groups (HG).

F: To manage heavy isotopes for glycerolipids, please go to Managing heavy isotopes for details.

G: To select MS^2 fragments for glycerolipids, please go to Selection of MS2 fragments for details.

H: To apply filters for glycerolipids, please go to Filters for the transition list for details.

I: Adds the complete lipid assembly into the 'Lipid list" basket.

J-K: To modify a lipid assembly, double click on the icon to retrieve information according to Step 1 window. After making changes (including HG selection, FA profile, adducts selection, management of heavy isotopes, MS^2 fragments selection and filters selection), click on 'Modify lipid' from Step 2 window to update the assembly.

L: To delete a lipid assembly from the 'Lipid list', double click on the Icon.

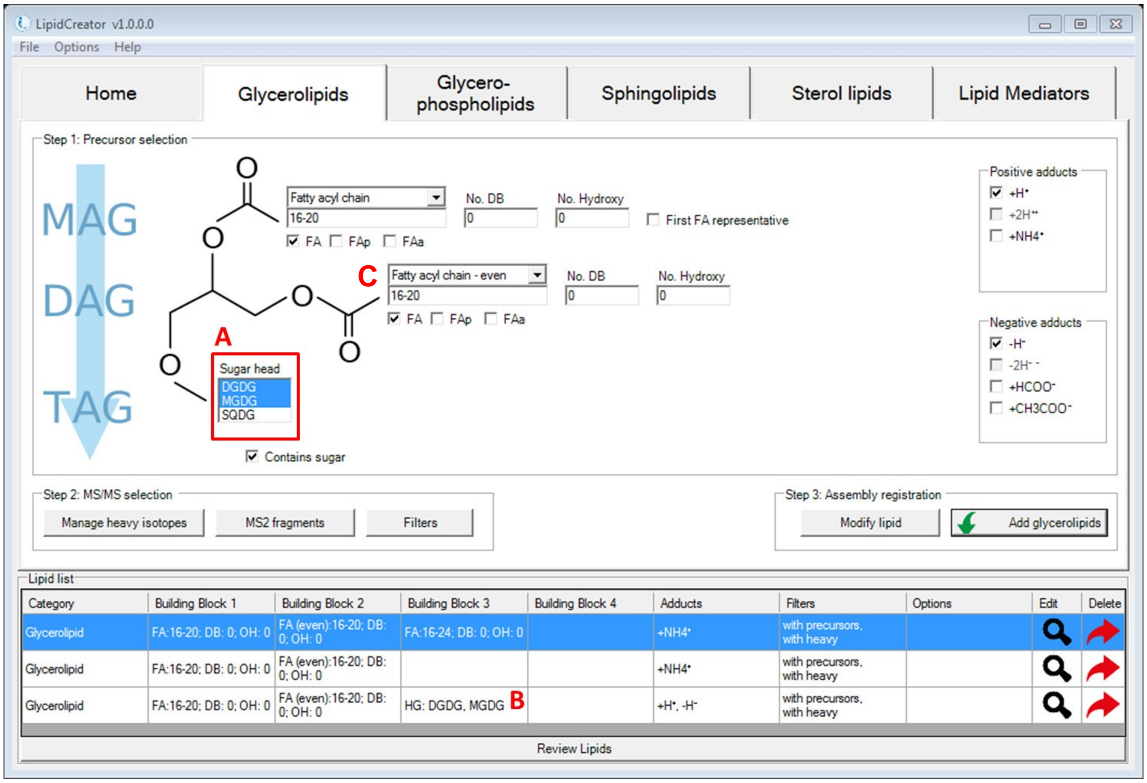

Figure T3. Interface for the definition of transitions for glycerolipids with glucose head group.

A: Multiple selection is possible when choosing head groups in LipidCreator.

B: The selected head groups are displayed in 'Lipid list'.

C: Ester- or ether-linked fatty acyls (fatty acid, plasmenyl or plasmanyl) can be defined and parameterized.

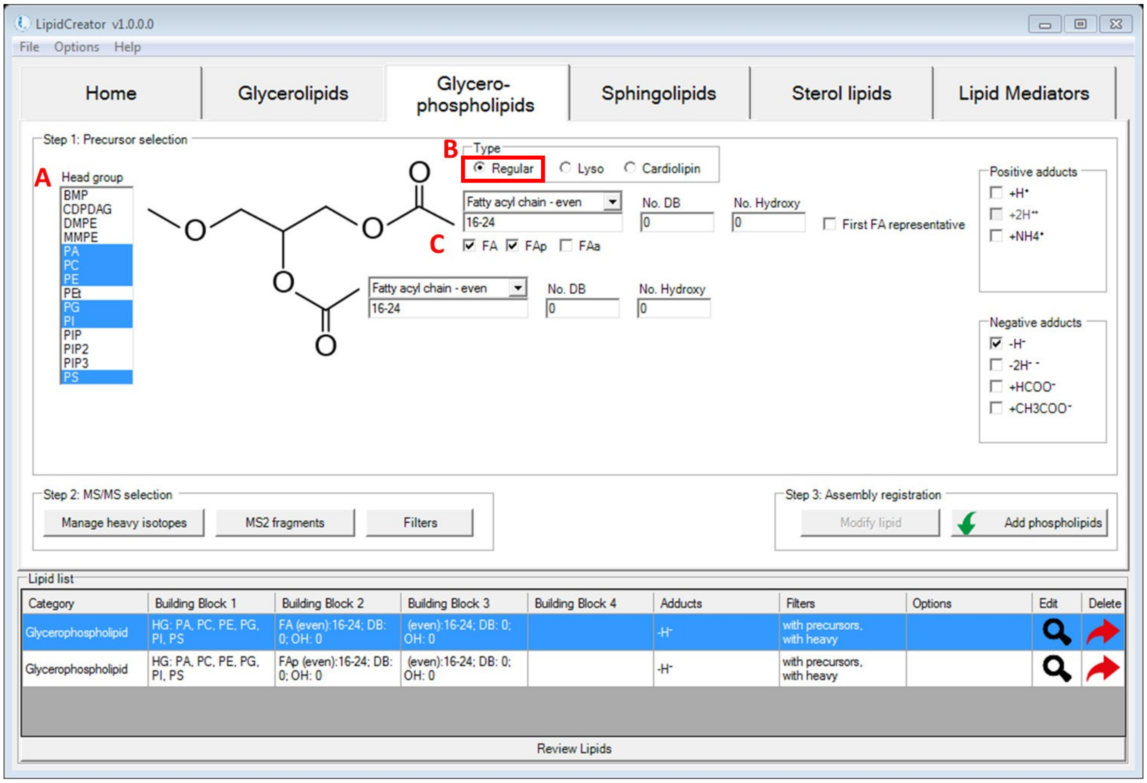

Figure T4. Interface for the definition of transitions for glycerophospholipids.

A: Multiple selection of head groups is possible when choosing them from the head group menu.

B: Radio buttons switch between different types of glycerophospholipids.

C: Ester- or ether-linked fatty acyls (fatty acid, plasmenyl or plasmanyl) can be defined and parameterized.

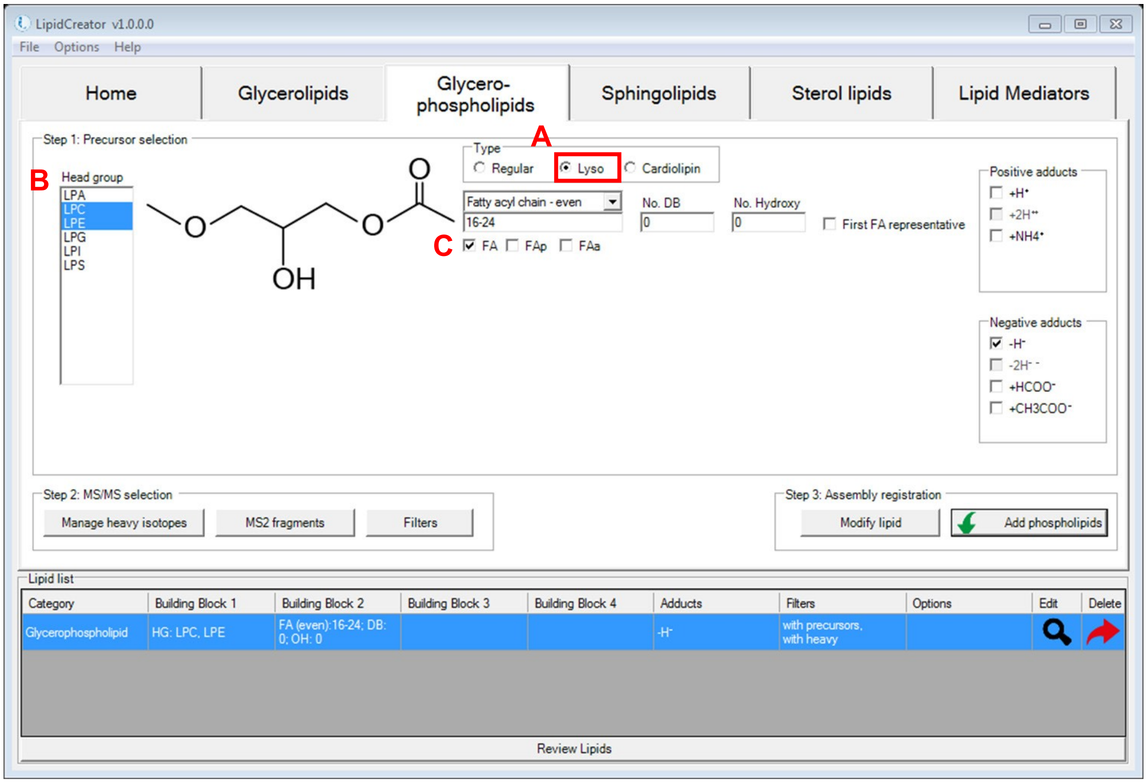

Figure T5. Interface for the definition of transitions for lyso-glycerophospholipids.

A: Selecting the ‘Lyso’ radio button switches the Glycerophospholipids tab into ‘lyso’ mode.

B: Here, only ‘Lyso’ headgroups for PA, PC, PE, PG, PI and PS are displayed.

C: Ester- or ether-linked fatty acyls (fatty acid, plasmenyl or plasmanyl) can be defined and parameterized.

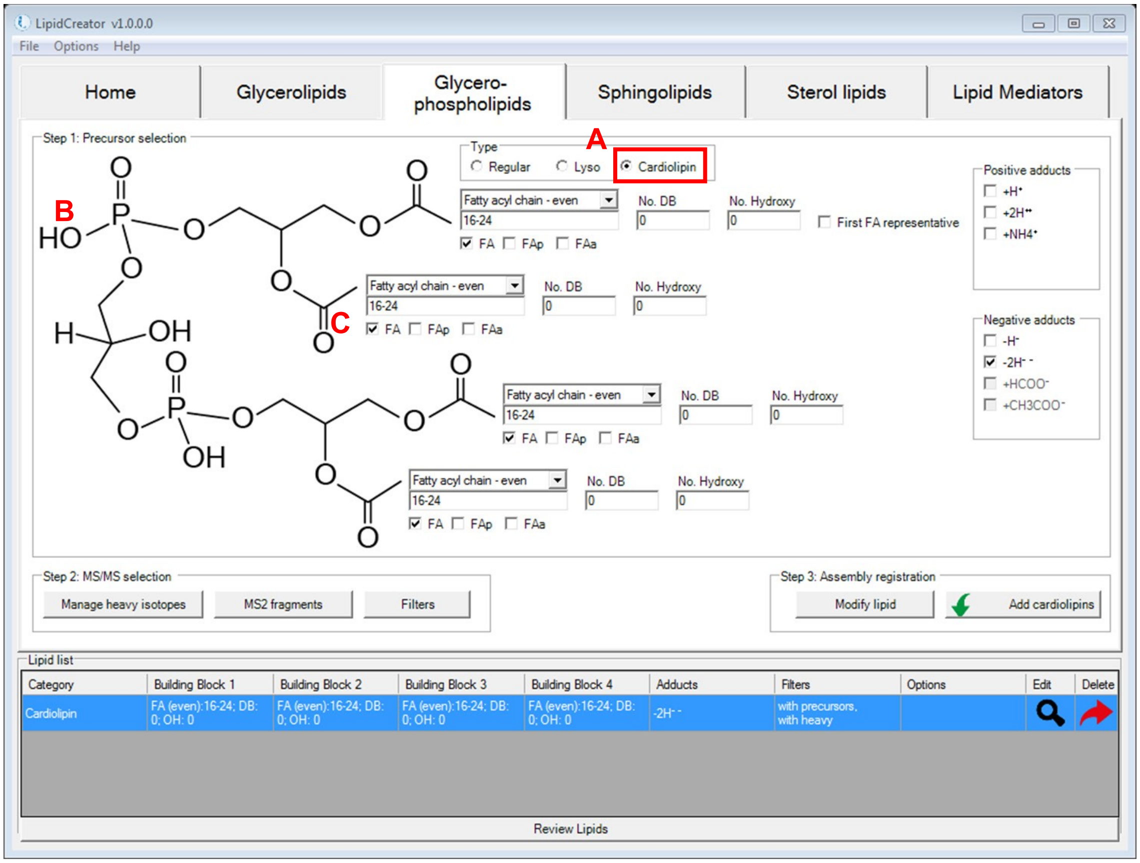

Figure T6. Interface for the definition of transitions for cardiolipins.

A: Selecting the ‘Cardiolipin’ radio button switches the Glycerophospholipids tab into ‘Cardiolipin’ mode.

B: Here, only the four fatty acyl chains can be defined. The headgroup is fixed and non-modfiable.

C: Ester- or ether-linked fatty acyls (fatty acid, plasmenyl or plasmanyl) can be defined and parameterized.

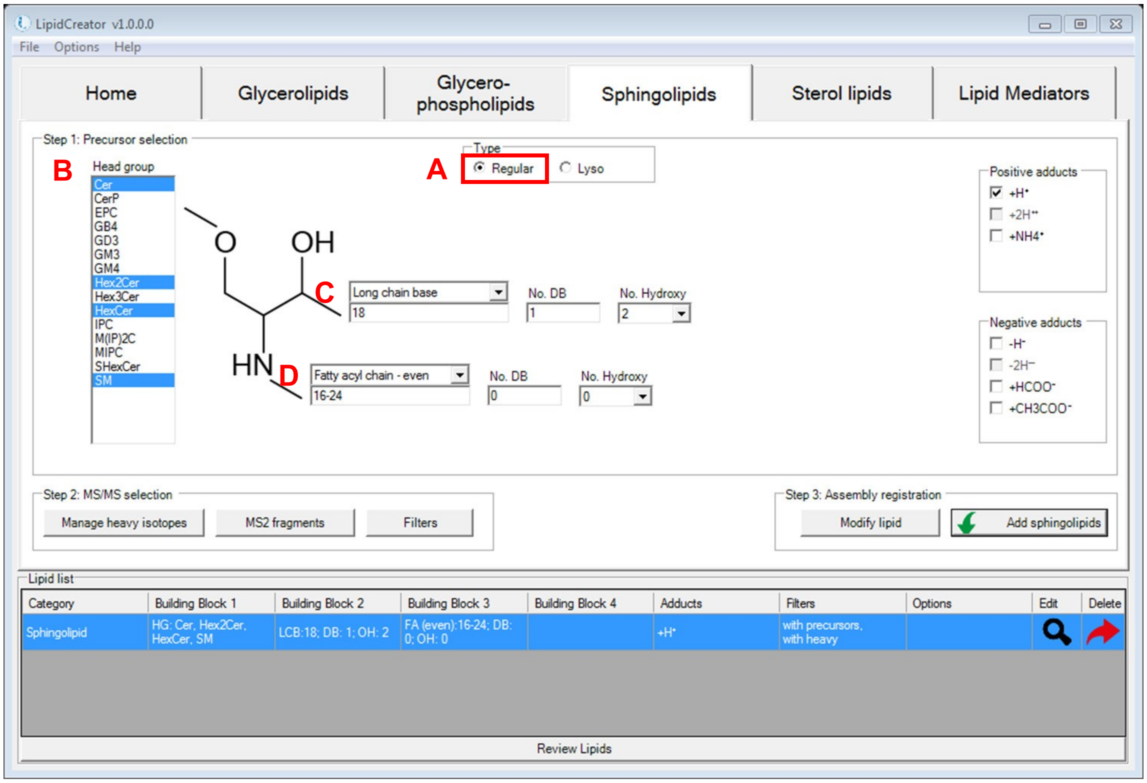

Figure T7. Interface for the generation of transitions for sphingolipids.

A: Radio buttons switch between different backbones of sphingolipids. In this case, the ‘Regular’ type is selected.

B: Head group selection for sphingolipids in LipidCreator.

C: Long-chain base definition for sphingolipids.

D: The fatty acyl chain can be defined and parameterized.

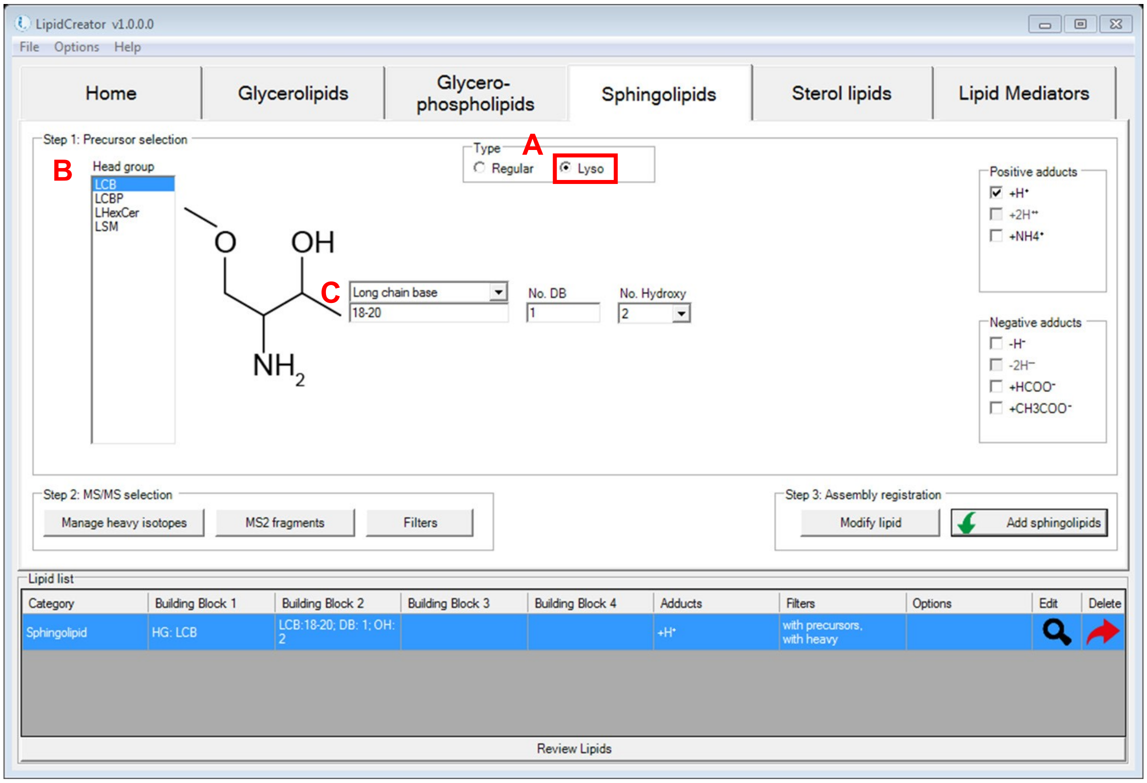

Figure T8. Interface for the definition of transitions for lyso-sphingolipids.

A: Radio buttons switch between different backbones of sphingolipids. In this case, the ‘Lyso’ type is selected.

B: Head group selection for Lyso-sphingolipids in LipidCreator.

C: Long-chain base definition for sphingolipids.

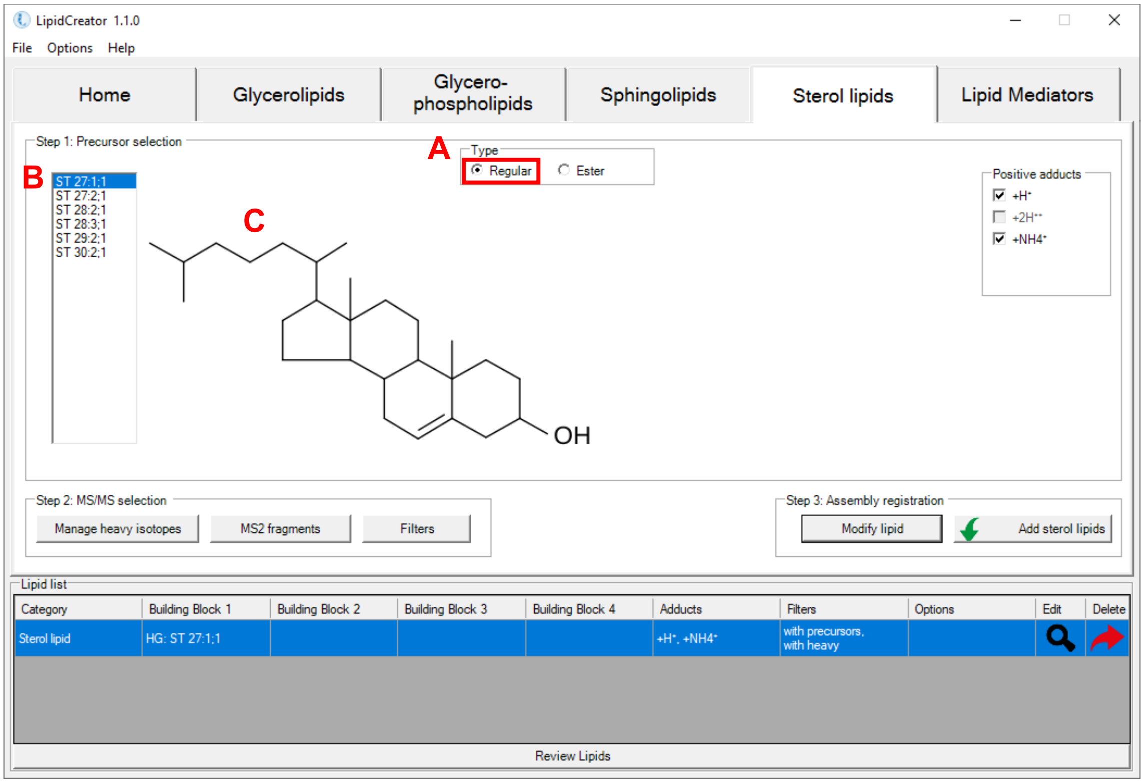

Figure T9. Interface for the definition of transitions for sterols.

A: Radio buttons switch between regular sterols and sterol esters. In this case, the ‘Regular’ type is selected.

B: Head group selection for sterol lipids in LipidCreator.

C: Cholesterol backbone. In this special case, no further parameterization is supported, apart from the adducts selection. Please see the next page for sterol esters.

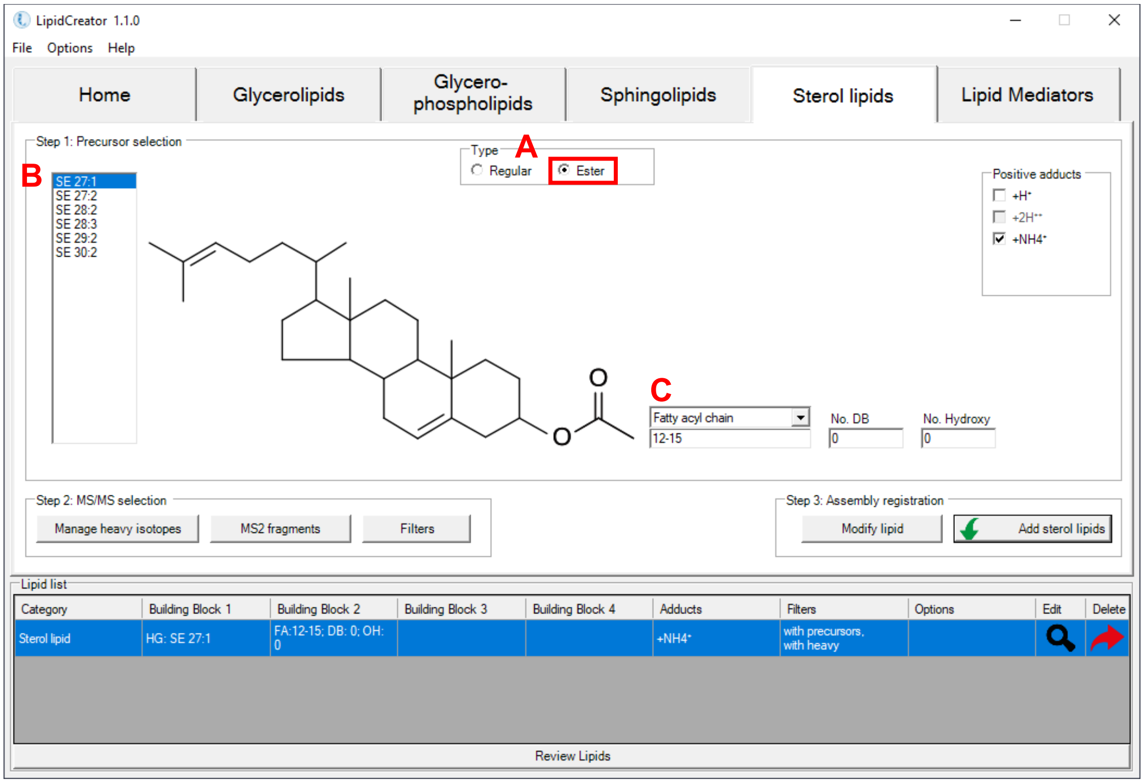

Figure T10. Interface for the definition of transitions for sterol esters.

A: Selecting the ‘Ester’ radio button switches the ‘Sterol lipids’ tab to sterol ester mode.

B: Head group selection for sterol ester lipids.

C: The fatty acyl chain can be defined and parameterized.

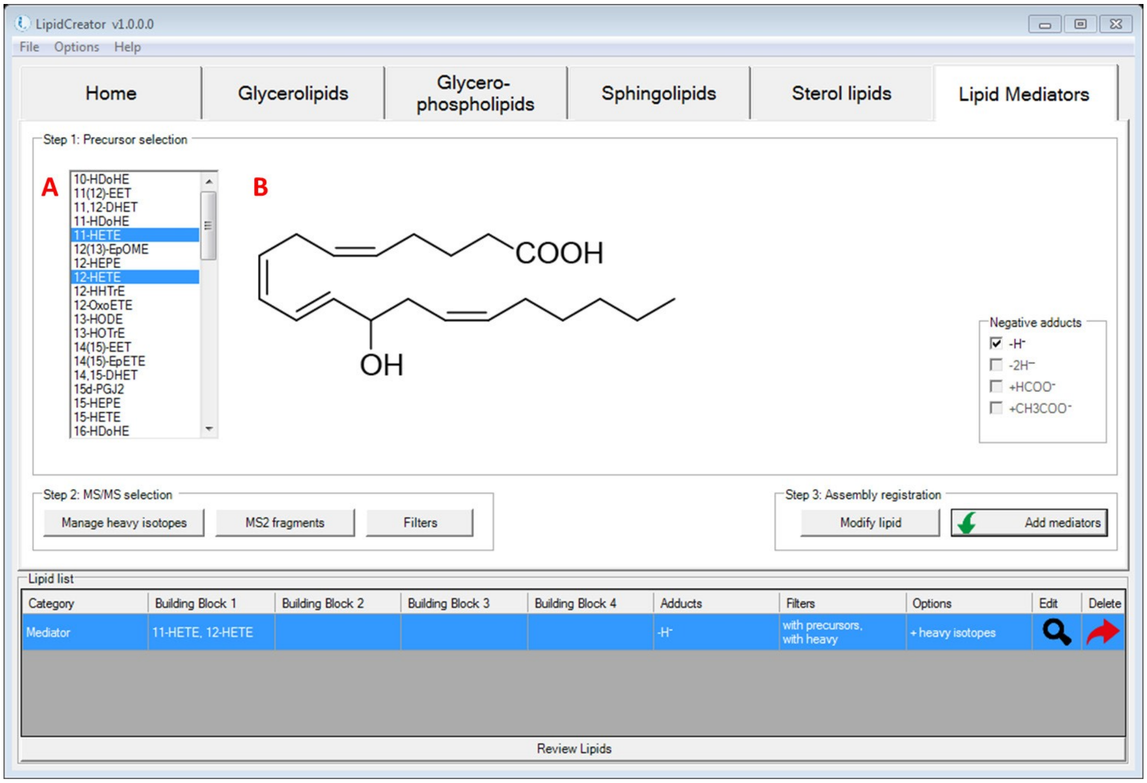

Figure T11. Interface for the definition of transitions for lipid mediators.

A: Head group selection for lipid mediators in LipidCreator.

B: This area displays the chemical structure of each lipid mediator when hovering the mouse cursor over different mediator names.

T4. Import/export lipid list/setting/project

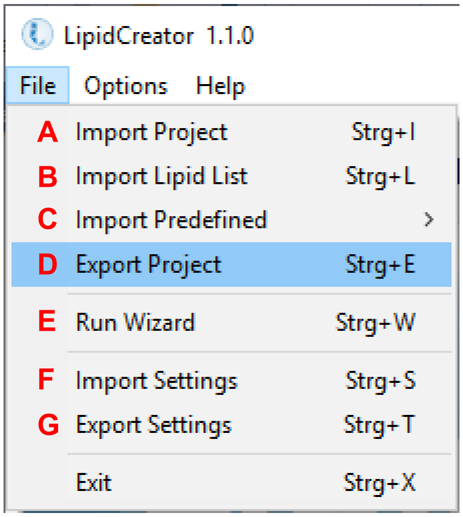

Figure T12. File Menu of LipidCreator.

A: Import project into LipidCreator. A project includes the lipid list, user defined MS^2 fragments, user-defined heavy labelled isotopes, and a selection of optimal collision energies.

B: Import lipid list from *.csv file. The lipid list should follow the nomenclature described in Table S2. Otherwise, please use the lipid name translator (Lipid name translator) for import. In the *.csv file, lipid names (including adduct name) should be given one per line.

C: Import predefined lipid lists from previous work.

PNAS yeast, DOI: 10.1073/pnas.0811700106
Mouse brain, DOI: 10.1007/s13361-014-1013-x and 10.1038/s41592-018-0010-6
Mouse heart, DOI: 10.1016/j.celrep.2018.08.017
Mouse platelet, doi: 10.1182/blood-2017-12-822890
Human platelet, doi: 10.1182/blood-2017-12-822890

D: Export current project in *.lcXML format from LipidCreator for storage.

It is possible to add user-defined project into 'Predefined' when use LipidCreator standalone. Copy *.lcXML file into the folder (or create new folder) at ../LipidCreator/data/predefined.

E: Start the LipidCreator Wizard. This will guide you through the steps required to set up a lipidomics transition list (Using the Wizard).

F: Import settings in LipidCreator. Settings include user defined MS^2 fragments, user defined heavy labelled isotopes, and a selection of optimal collision energies.

G: Export the current settings in *.lcXML format.

T5. Lipid name translator

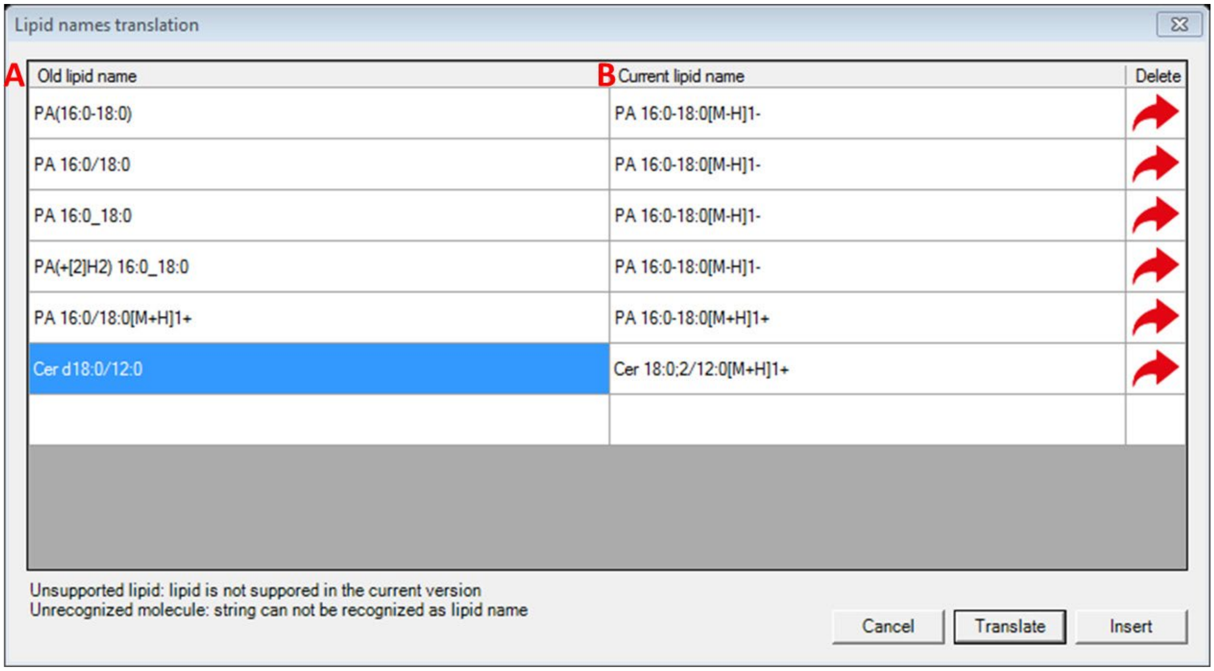

Figure T13. Interface for the lipid name translator (Options→Lipid name translator).

A: List of old lipid names. Not all isotope formats can be recognized directly from the name. The isotope labels need to be defined additionally in LipidCreator.

B: List of translated lipid name according to the nomenclature in LipidCreator. When the old lipid name has no adduct defined, a default adduct will be appended to the translated name automatically. After translation, the list will be imported into LipidCreator by clicking on 'Insert'.

T6. Selection of MS2 fragments

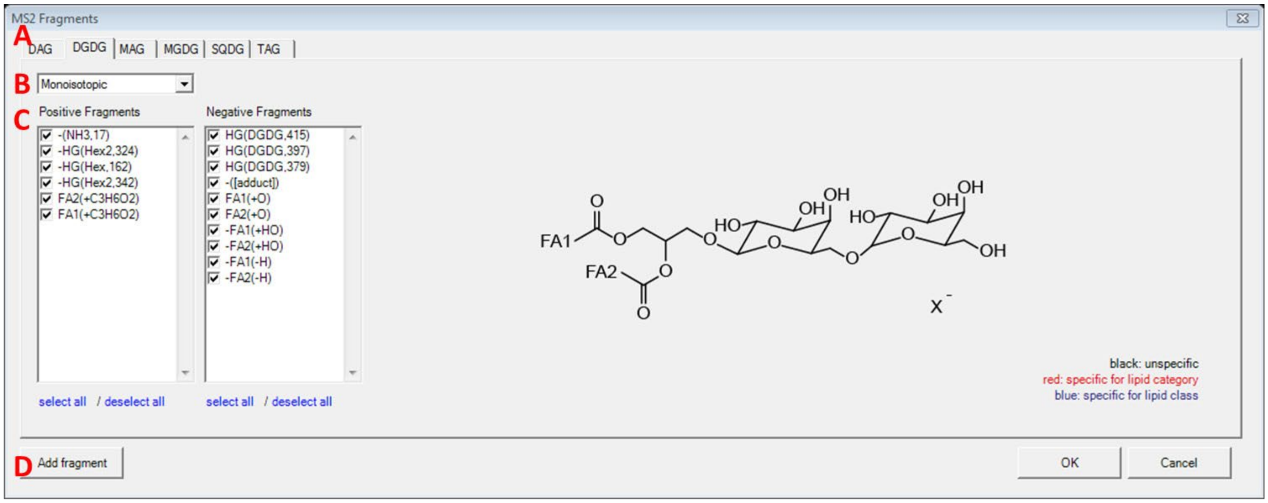

Figure T14. Interface for MS^2 fragments for glycerolipids.

A: Tabs for selecting lipid classes.

B: Drop-down list for selecting either monoisotopic or isotopic species. Isotopic coded species will only show up after being defined (Managing heavy isotopes).

C: List of MS^2 fragment types of each lipid class for positive and negative mode. Each predefined fragment type offers a chemical structure preview on the right side.

D: Add user-defined fragments, please see Adding user-defined fragments for details.

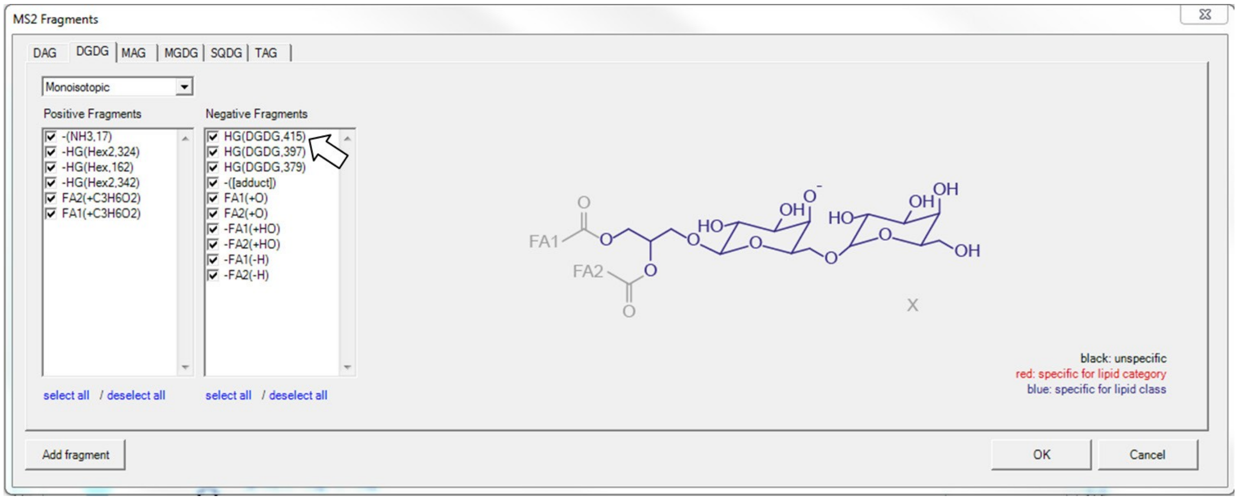

Figure T15. Preview of MS^2 fragments for glycerolipids when hovering over fragment names.

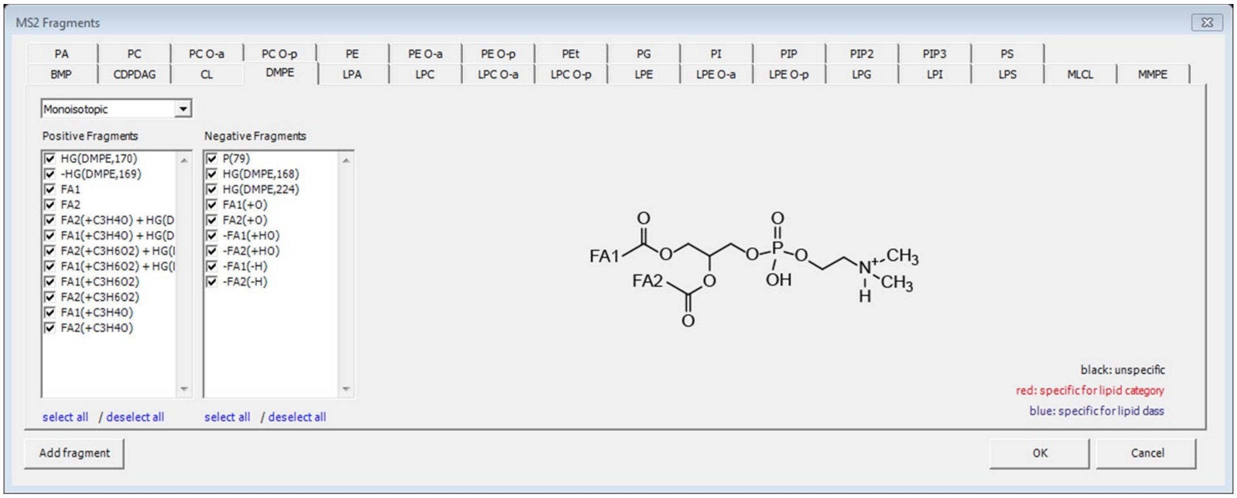

Figure T16. Interface for MS^2 fragments for glycerophospholipids.

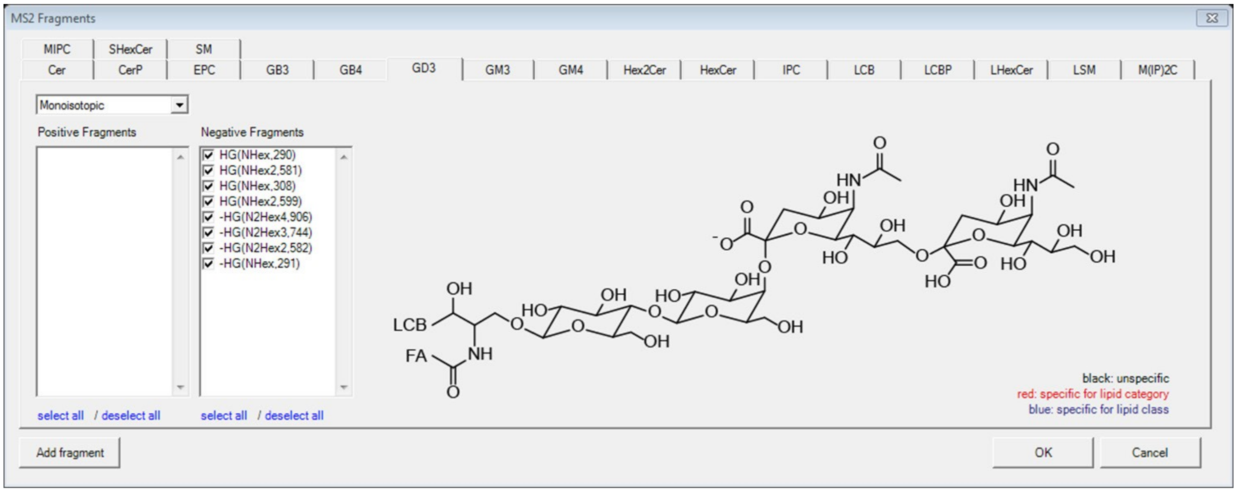

Figure T17. Interface for MS^2 fragments for sphingolipids.

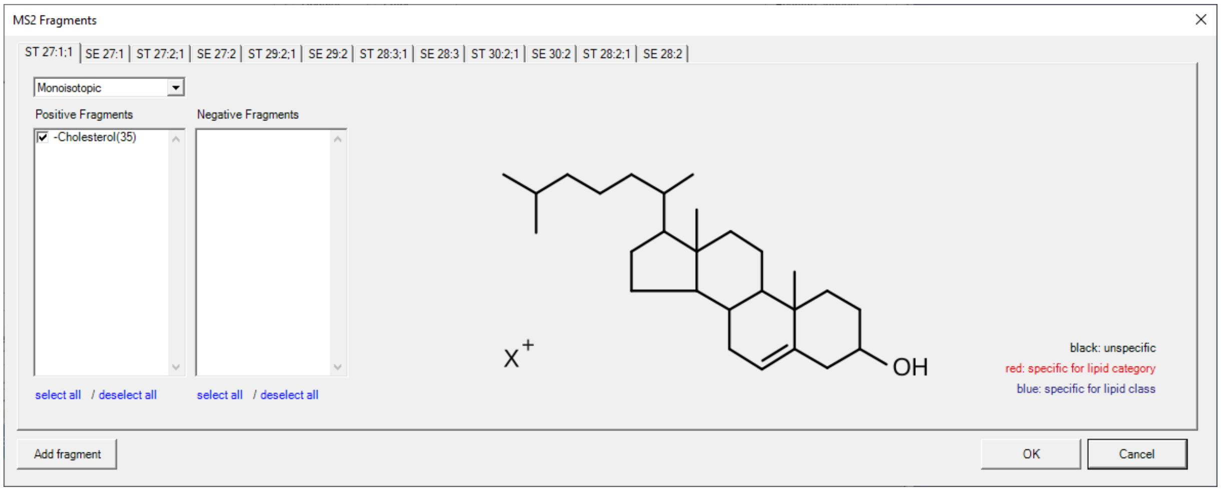

Figure T18. Interface for MS^2 fragments for sterols and cholesterol esters.

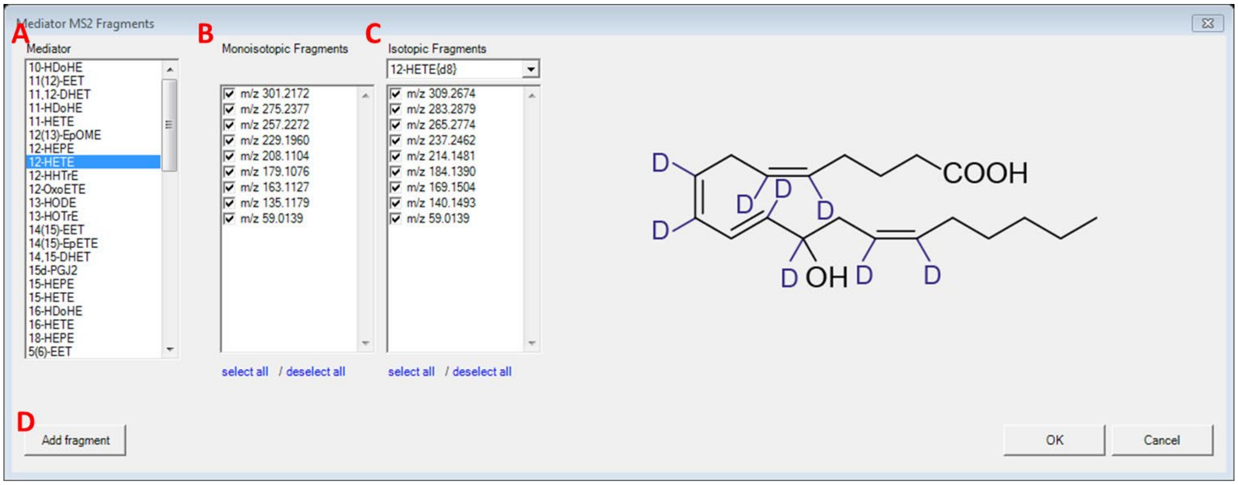

Figure T19. Interface for MS^2 fragment masses for lipid mediators.

A: List of individual lipid mediators.

B: List of MS^2 fragment masses for selected lipid mediator.

C: List of MS^2 fragment masses for isotope labelled versions of the selected lipid mediator.

D: Add user-defined fragments, please see Adding user-defined fragments for details.

T7. Adding user-defined fragments

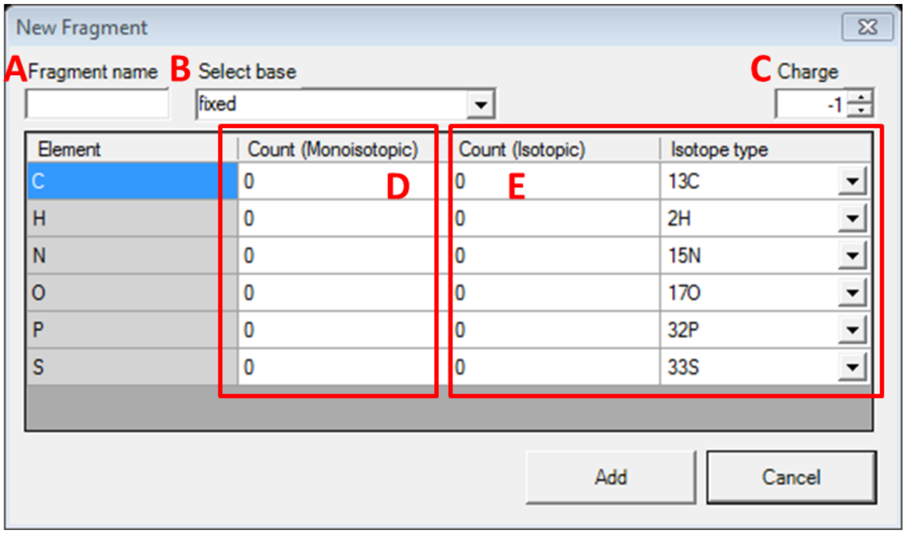

Figure T20. Interface for adding new fragments.

A: User-defined fragment name.

B: Select base of the user-defined fragment type. Depending on the chosen lipid class, either the base can be fixed or it can contain building blocks, e.g. HG, HG + FA1, FA1 + FA2, etc.

C: Fragment charge. A positive value indicates that this fragment originated from positive ionization mode, while a negative value indicates that this fragment originated from negative ionization mode.

D: A constant set of elements can be defined which will be added to the fragment. When 'fixed' base is selected, element numbers can only be positive; otherwise negative counts are also allowed.

E: A field to input constant set of isotopic elements and drop-down list to select the type of isotopic elements.

The next page shows how to add new types of fragments for PG.

Here is an example of how to add new types of fragments for PG.

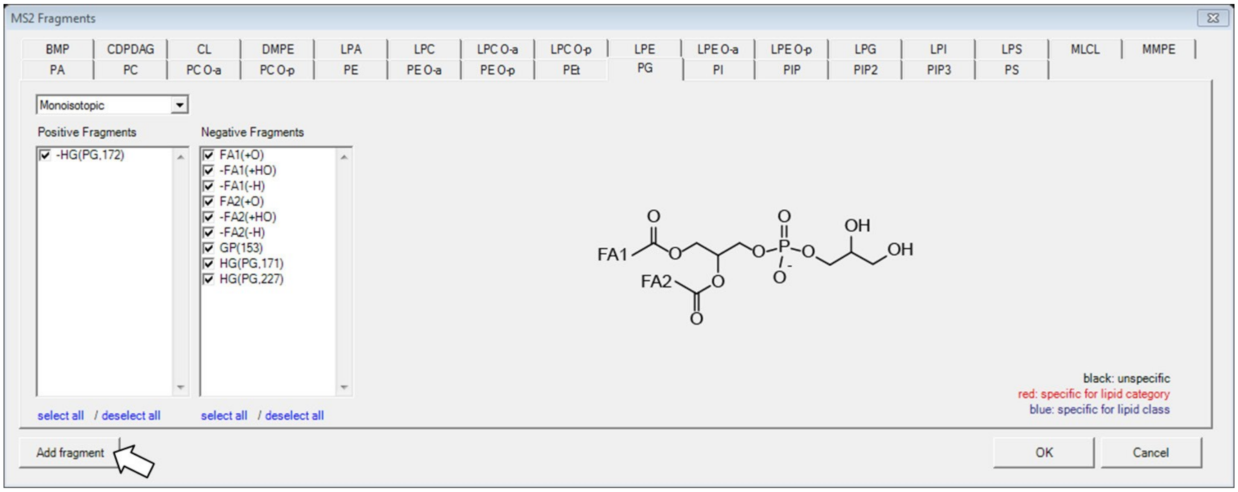

Figure T21. Click on ‘Add fragment’ from the PG tab of glycerophospholipids.

This will preselect the ‘PG’ class as the target class for the new fragment.

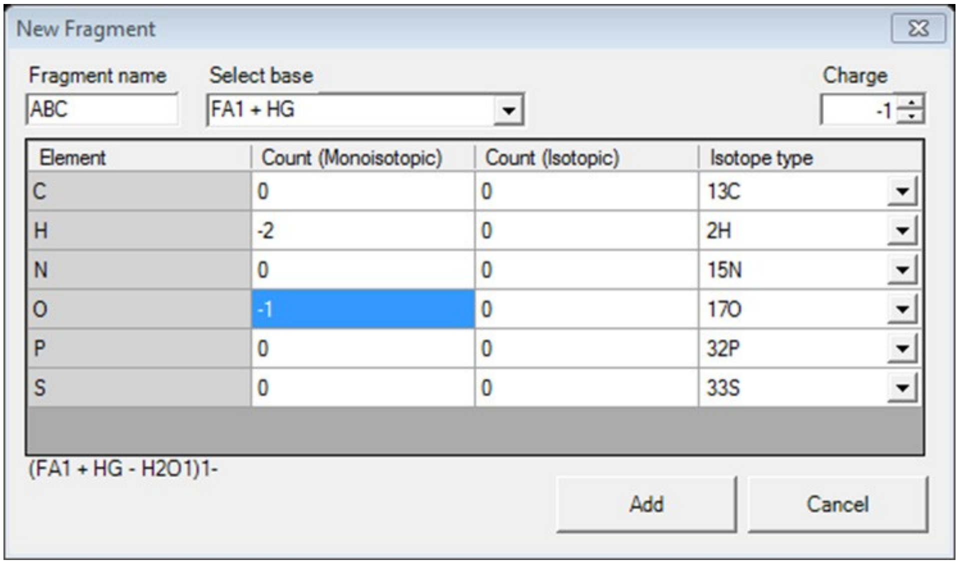

Figure T22. Generate a fragment ‘ABC’ for PG class.

In order to add a new fragment, enter its (unique) name, select the proper base and charge. Then, define the elemental composition of the fragment by entering the respective atom counts, either monoisotopic, or isotopic if you want to define a heavy labeled fragment. Click ‘Add’ to add the fragment to LipidCreator’s knowledge base. You can now include this new fragment within the ‘MS2 Fragments’ dialog.

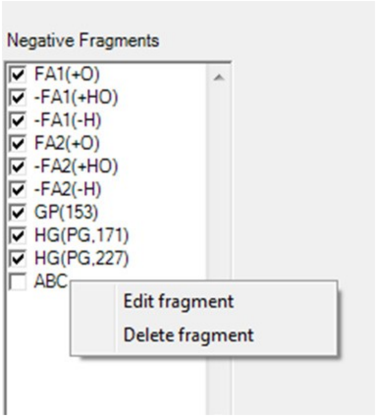

Figure T23. The 'ABC' fragment has been added to the fragment list.

User-defined fragments can be edited or deleted by right clicking on the fragment name.

Example: adding a new mediator fragment

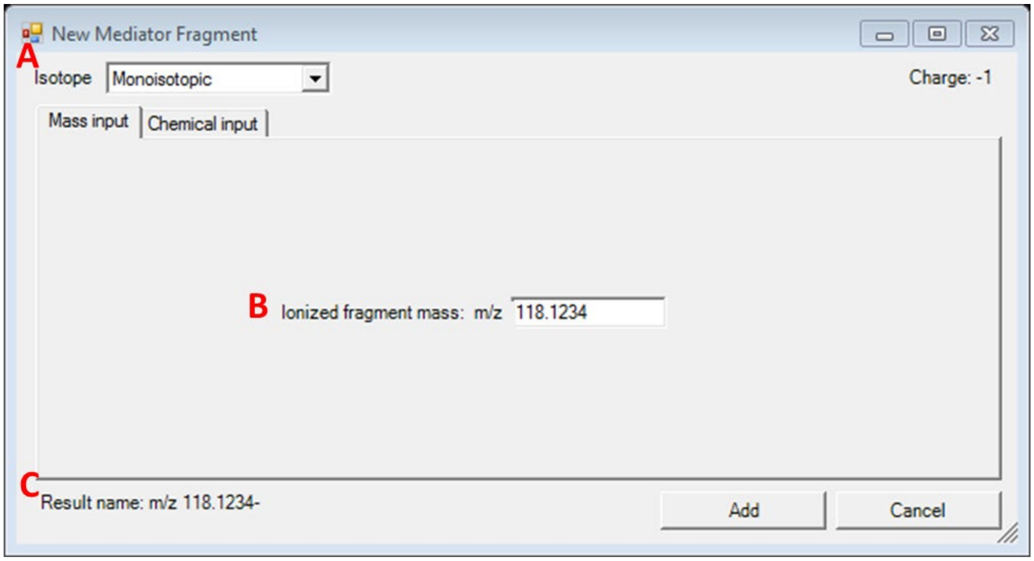

Figure T24. Adding a monoisotopic mediator fragment with direct mass input for Mediator.

A: In the 'Isotope' drop-down list, either monoisotopic or other isotopes can be selected to add a fragment, depending on the mediator selection in <u>Figure T19. Interface for MS2 fragment masses for lipid mediators.</u>

B: In the 'Mass Input' tab, please directly type in the ionized fragment mass. The fragment charge is set to -1 by default.

C: The preview for display name of the fragment.

The mass will appear in the transition list with exactly this value and will be identical in the current list. Identified replicates will be denied when adding.

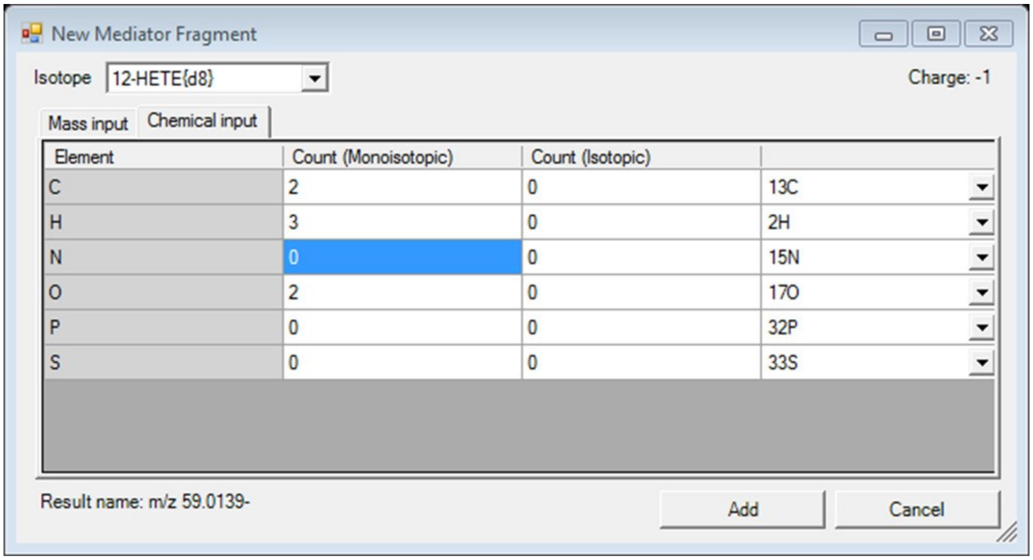

Figure T25. Adding a fragment using the chemical formula input for Mediator.

To add a fragment for a monoisotopic species, use only the ‘Count (Monoisotopic)’ column to enter the counts of elements. For heavy isotope species, both ‘Count (Monoisotopic)’ and ‘Count (Isotopic)’ are valid. The result name will be displayed as the (m/z) mass.

T8. Managing heavy isotopes

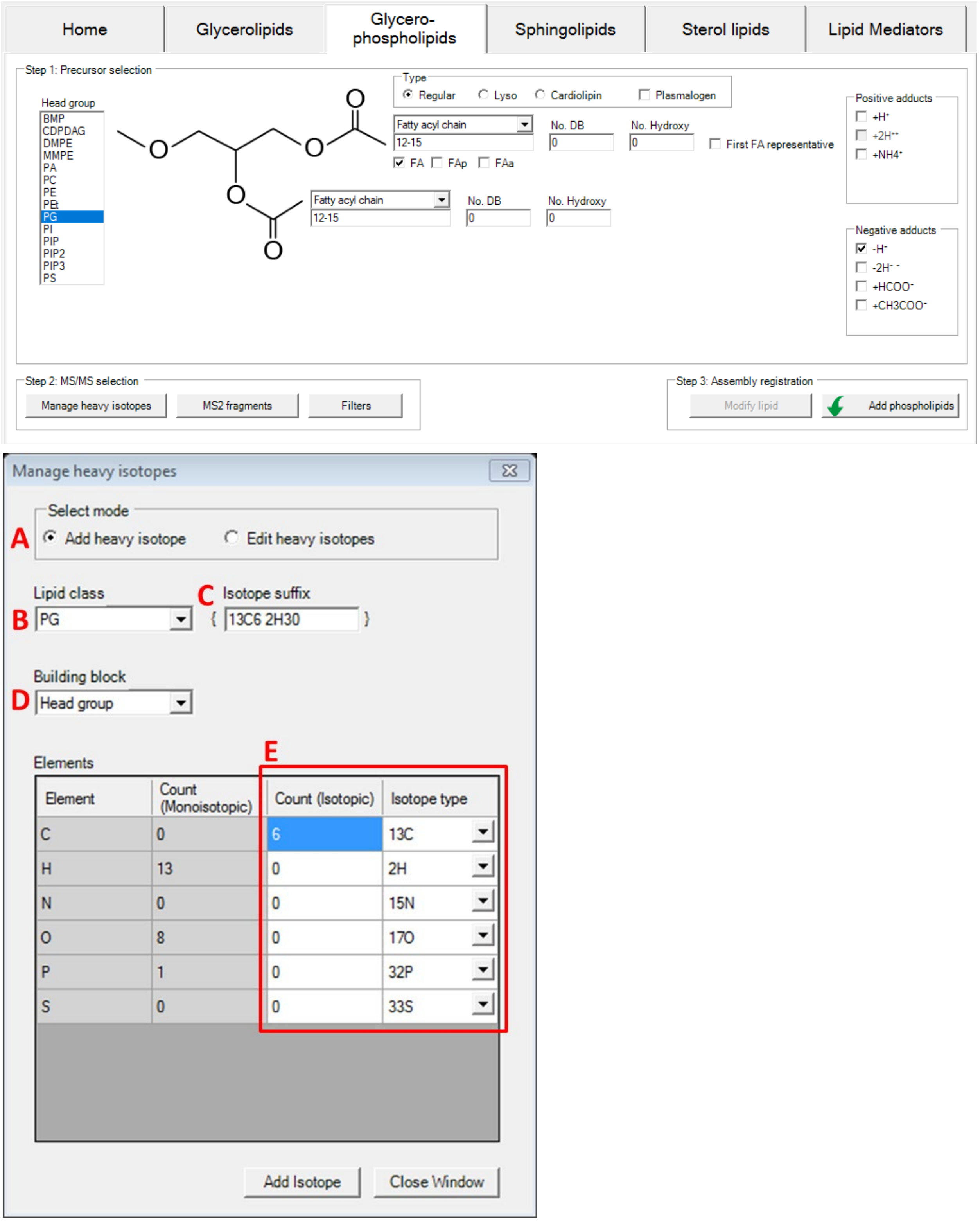

Figure T26. Interface for managing heavy isotopes for glycerophospholipids.

This interface is automatically adapted to currently selected lipid category tab. It is accessible from the ‘Manage heavy isotopes’ button in the ‘Step 2: MS/MS selection’ pane of each of the lipid category tabs within the LipidCreator user interface.

A: Radio buttons for either adding new isotopes or editing existing user-defined isotopes.

B: Drop-down list for selecting the lipid class (depending on the lipid category).

C: User-defined name as suffix for the lipid class.

D: Drop-down list for selecting building blocks.

E: A field to input constant set of isotopic elements. The count for monoisotopic elements will be automatically changed according to the typed count of isotopic elements.

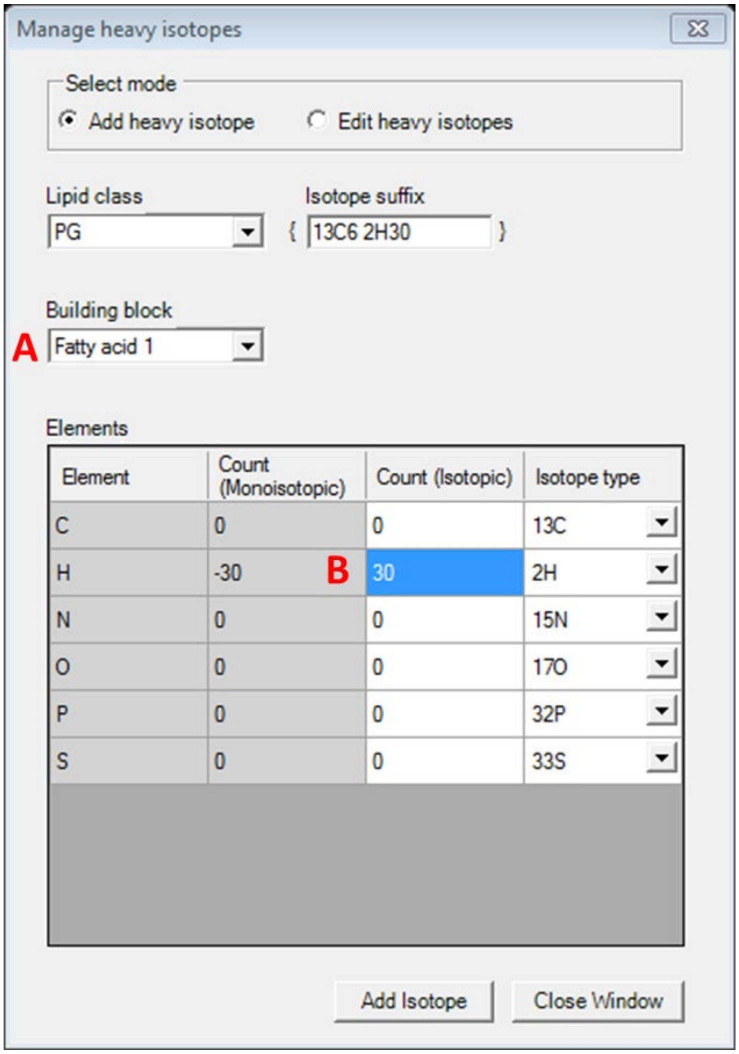

Figure T27. Interface for managing heavy isotopes for glycerophospholipids.

A: Select ‘Fatty acyl 1’ to add isotope elements.

B: The heavy-labelled element numbers act as an upper limit for the element, since the fatty acyl building block has a variable number of elements depending e.g. on the carbon chain length.

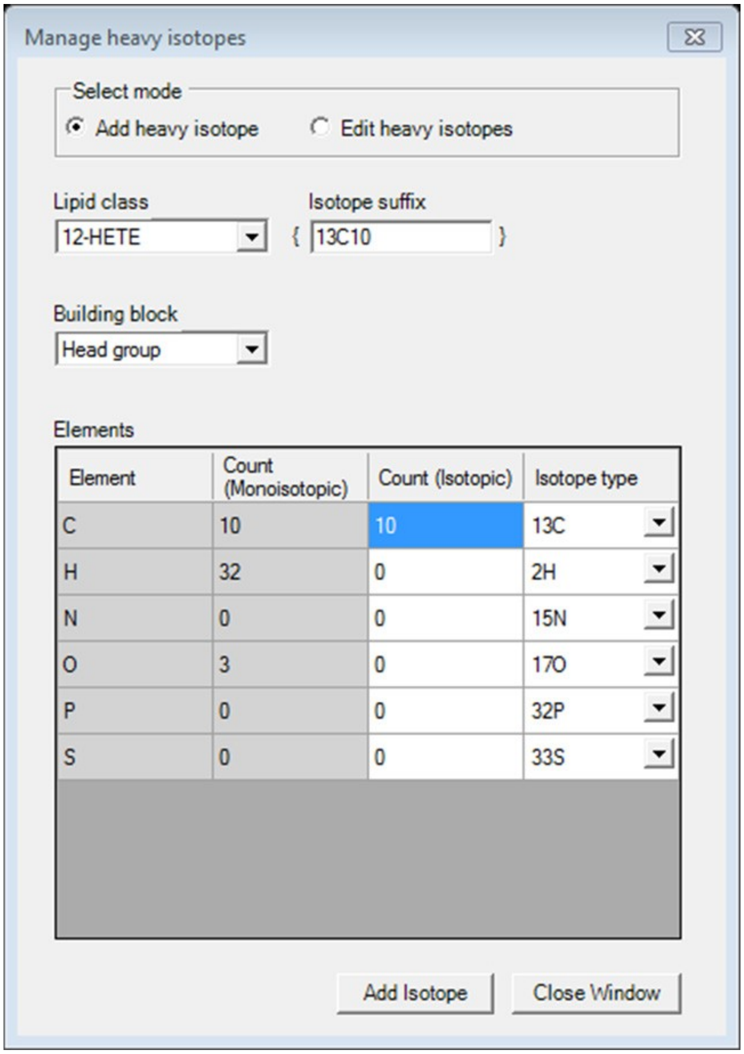

Figure T28. Interface for managing heavy isotopes for mediators.

The chemical formula (numbers of elements in 'Count (Monoisotopic)' column) for all mediators are provided when adding heavy isotopes. The only selectable building block is the 'Head group' in this case, which entails the complete mediator molecule.

T9. Filters for the transition list

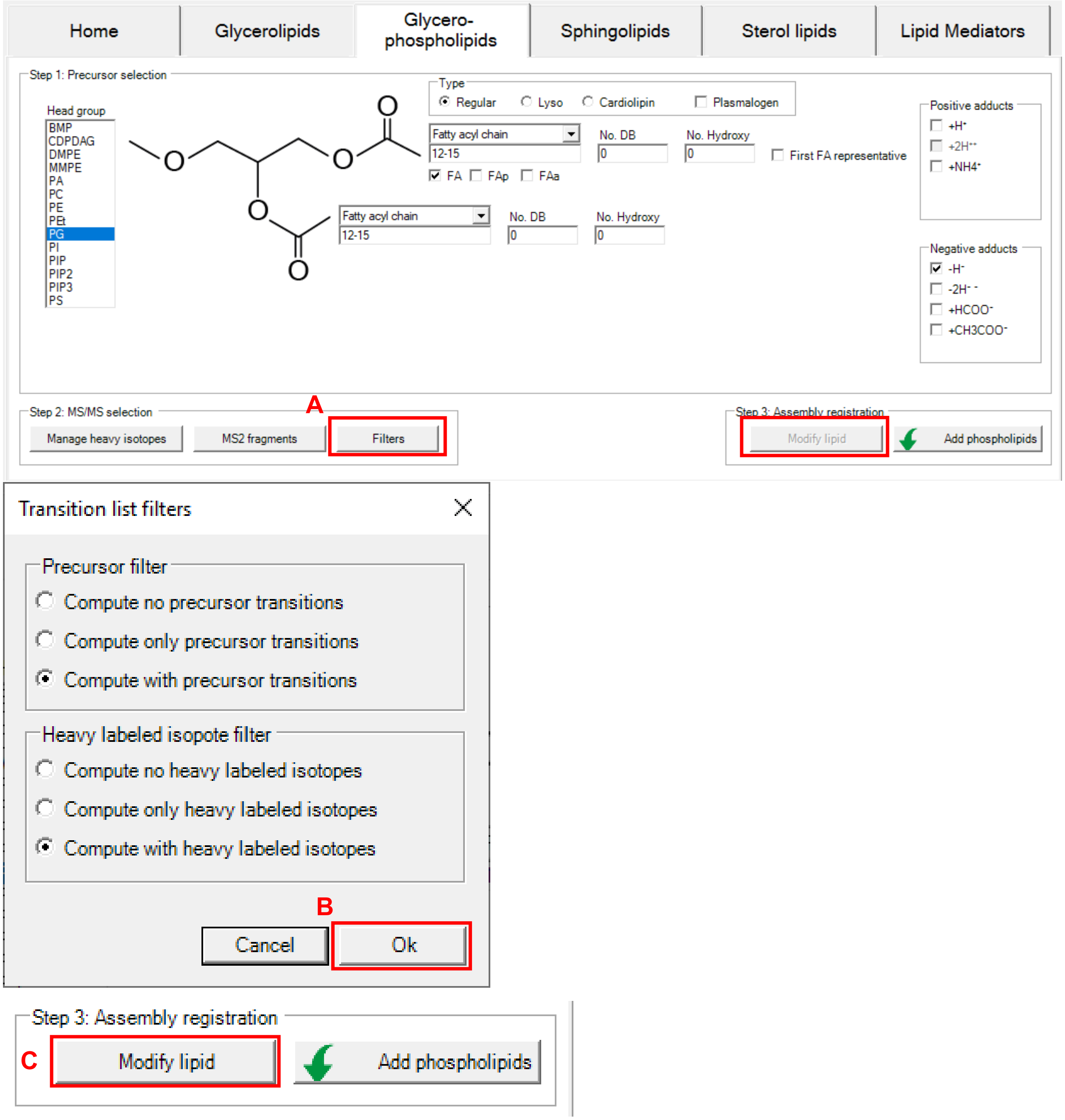

Figure T29. Filters are applied before the transition lists are calculated.

After changing the transition list filter settings (A), e.g. to exclude precursor transitions, click ‘Ok’ (B), then click on ‘Modify lipid’ (C) to update the ‘Lipid list’. Note: the button (C) is only enabled when you have at least one lipid of the current tab / lipid category in your ‘Lipid list’.

T10. Collision energy optimization function

Collision energy function is valid for lipid mediators. To activate this function, first click on Options→ 'Collision Energy Computation'→ 'Thermo Scientific Q Exactive HF' or 'Agilent 6545 Q-TOF LC/MS'.

Afterwards, open the dialog from Options→ 'Collision Energy optimization'.

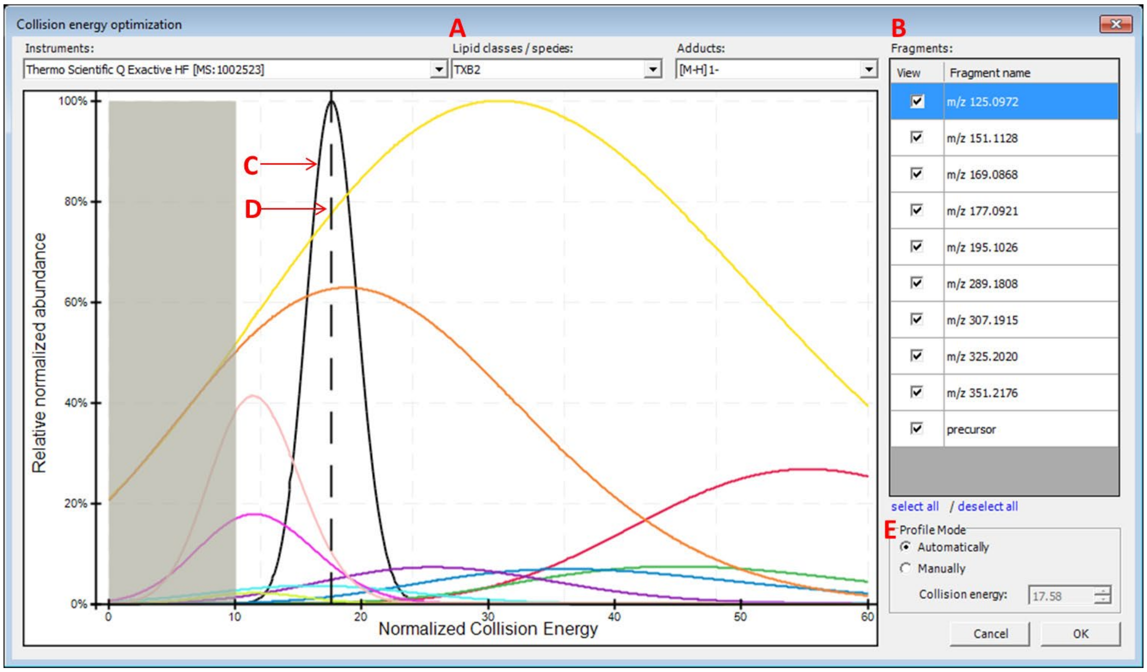

Figure T30. Collision energy optimization interface.

A: Choose a lipid mediator species from the drop-down list. Isotopes appear as separate entries to their monoisotopic counterparts.

B: Fragment list for the chosen lipid species. Optimal collision energy depends on fragment selection.

C: The black curve is the automatically calculated product distribution over all selected fragment distributions from the list. Its mode indicates the optimal collision energy over all selected fragments.

D: The dashed line indicates the chosen collision energy. This line is moveable in manual profile mode.

E: Automatic or manual profile mode can be selected. When the manual profile mode is activated, either move the dashed line with the mouse or type in a collision energy value of your choice.

Activating the computation of collision energy is independent from added lipid species in the 'Lipid list'. The collision energy can be defined before or after lipid assembly, these two information will combine after click on 'Review Lipids'.

By applying collision energy computation, the CE value can be used either to generate an MS method from the transition list or to provide corresponding relative intensities for fragments in the generated *in-silico* spectral library.

T11. Reviewing the lipid transition list

After clicking on ‘Review Lipids’, the calculated lipid precursor names (Figure T31. Interface for reviewing the name of calculated lipid precursors.) will be listed for further selection before calculation of the final transitions (Figure T32. Interface for reviewing calculated lipid transitions. & Figure T33. Interface for reviewing calculated lipid transitions with collision energy.).

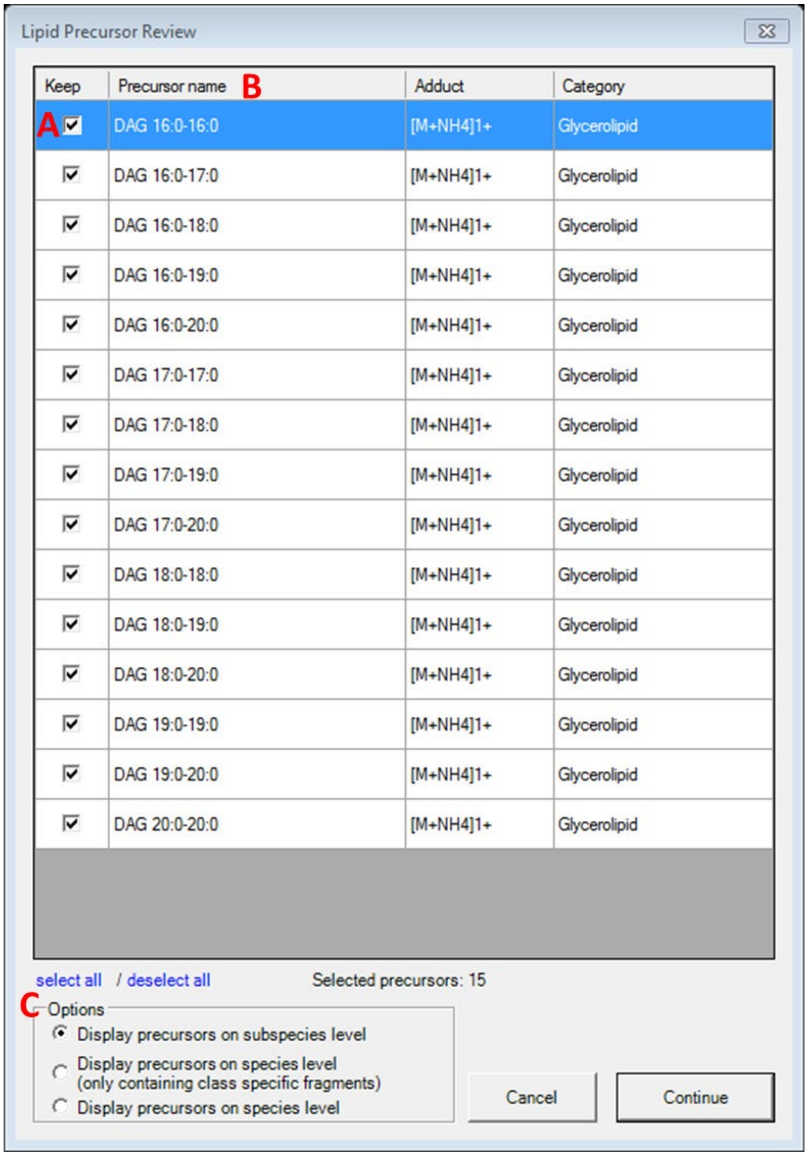

Figure T31. Interface for reviewing the name of calculated lipid precursors.

A: The checkbox allows you to select/deselect the generated lipids for further calculation of transitions.

B: Click on the name of each column to activate sort ascending/descending functions.

C: Display options between subspecies and species level of lipid name. e.g. DAG 16:0-16:0 in subspecies level, DAG 32:0 in species level.

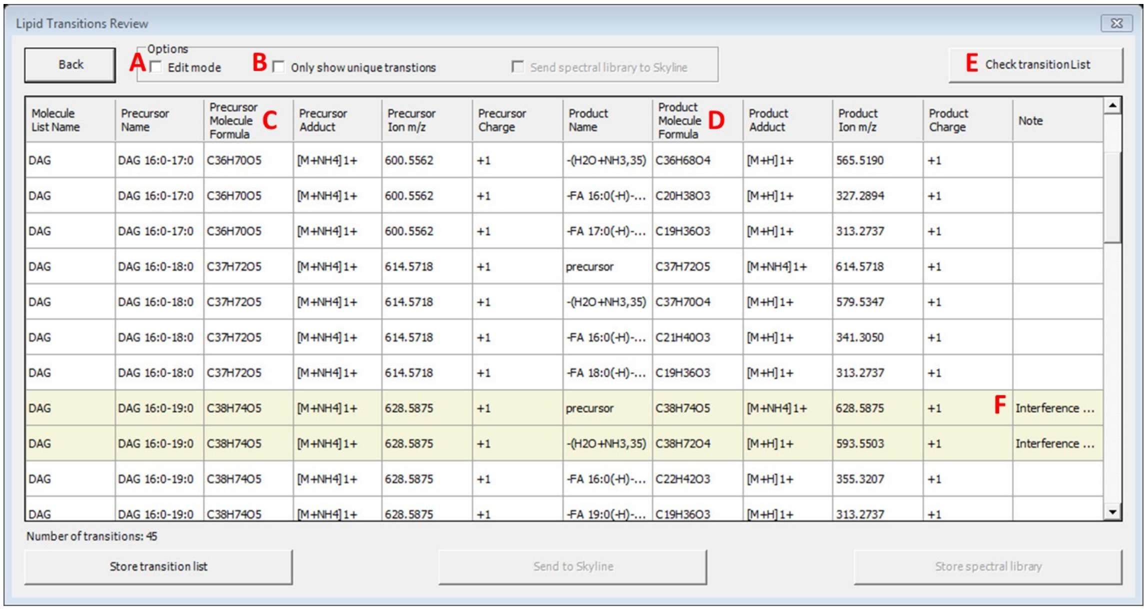

Figure T32. Interface for reviewing calculated lipid transitions.

A: The checkbox allows you to activate user's edit mode for manually editing, adding and deleting items within the transition list.

B: The checkbox allows you to discard the non-unique transitions from the review panel and further actions.

C & D: The chemical formulas in the transition list represent neutral lipids/fragments. The masses represent ionized precursors/fragments after adding the corresponding adducts.

E: This button is to check whether the current transition list is compatible with Skyline.

F: In the review list, the non-unique transitions (only the precursor and product masses are considered) will be highlighted and noted when being repeated. The note will be imported to Skyline when the transition list is being sent.

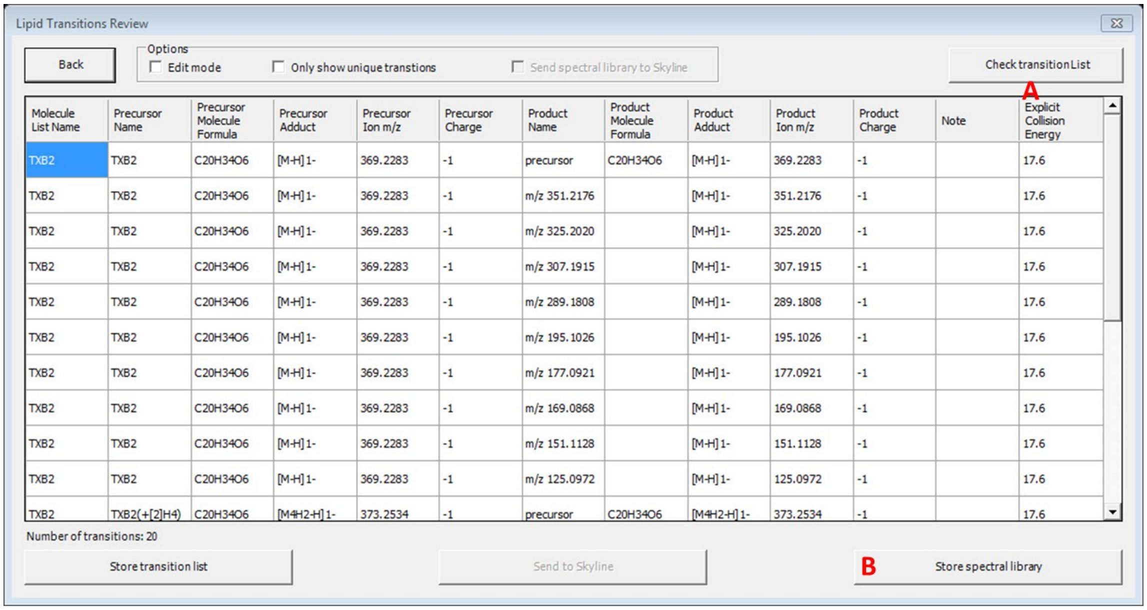

Molecule List Name	Precursor Name	Precursor Molecule Formula	Precursor Adduct	Precursor Ion m/z	Precursor Charge	Product Name	Product Molecule Formula	Product Adduct	Product Ion m/z	Product Charge	Note	Explicit Collision Energy
TXB2	TXB2	C20H34O6	[M-H]1-	369.2283	-1	precursor	C20H34O6	[M-H]1-	369.2283	-1		17.6
TXB2	TXB2	C20H34O6	[M-H]1-	369.2283	-1	m/z 351.2176		[M-H]1-	351.2176	-1		17.6
TXB2	TXB2	C20H34O6	[M-H]1-	369.2283	-1	m/z 325.2020		[M-H]1-	325.2020	-1		17.6
TXB2	TXB2	C20H34O6	[M-H]1-	369.2283	-1	m/z 307.1915		[M-H]1-	307.1915	-1		17.6
TXB2	TXB2	C20H34O6	[M-H]1-	369.2283	-1	m/z 289.1808		[M-H]1-	289.1808	-1		17.6
TXB2	TXB2	C20H34O6	[M-H]1-	369.2283	-1	m/z 195.1026		[M-H]1-	195.1026	-1		17.6
TXB2	TXB2	C20H34O6	[M-H]1-	369.2283	-1	m/z 177.0921		[M-H]1-	177.0921	-1		17.6
TXB2	TXB2	C20H34O6	[M-H]1-	369.2283	-1	m/z 169.0868		[M-H]1-	169.0868	-1		17.6
TXB2	TXB2	C20H34O6	[M-H]1-	369.2283	-1	m/z 151.1128		[M-H]1-	151.1128	-1		17.6
TXB2	TXB2	C20H34O6	[M-H]1-	369.2283	-1	m/z 125.0972		[M-H]1-	125.0972	-1		17.6
TXB2	TXB2(+[2]H4)	C20H34O6	[M4H2-H]1-	373.2534	-1	precursor	C20H34O6	[M4H2-H]1-	373.2534	-1		17.6

Figure T33. Interface for reviewing calculated lipid transitions with collision energy.

A: The ‘Explicit Collision Energy’ column will appear after collision energy computation has been activated.

B: The ‘Store spectral library’ button is valid after collision energy computation has been activated.

T12. Storing a transition list

Following the steps described in Reviewing the lipid transition list, the reviewed transition list can be exported as a
*.csv or *.xls file by clicking on 'Store transition list'. The list can be stored in either one full list or it can be split into two lists separated by polarity mode.

T13. Storing a spectral library

The creation of a spectral library is possible after collision energy computation is activated according to Collision energy optimization function. Spectral libraries are written in Skyline *.blib format, which is a SQLIte database file.

T14. Integration with Skyline

1. Installation of Skyline

Please install Skyline through https://skyline.ms/project/home/software/Skyline/begin.view

2. Install LipidCreator in Skyline

Please install LipidCreator from Skyline Tools→Tool store.

Or

Please add the downloaded .zip file to Skyline through Tools→ External Tools→ Add→ From file…→ Choose LipidCreator.zip→ Wait until LipidCreator shows in the 'Menu contents' (this step may take some seconds)→ OK.

3. Create transition list for Skyline

Start LipidCreator from Tools and use it as described in Section T1 and following. After the created transition list has been reviewed, click on 'Send to Skyline'. The lipid list will appear in the 'Targets' window in Skyline. Then either export the project (see Import/export lipid list/setting/project) for further editing or close LipidCreator directly.

4. Create *In-silico* spectral library

After activating the collision energy computation (see Collision energy optimization function), the created transition list and spectral library can be sent to Skyline at once (Figure T34. Interface for reviewing calculated lipid transition with collision energy on Skyline platform.).

Or

Add *.blib file manually through Settings→Peptide Settings→Library→Edit list→Add

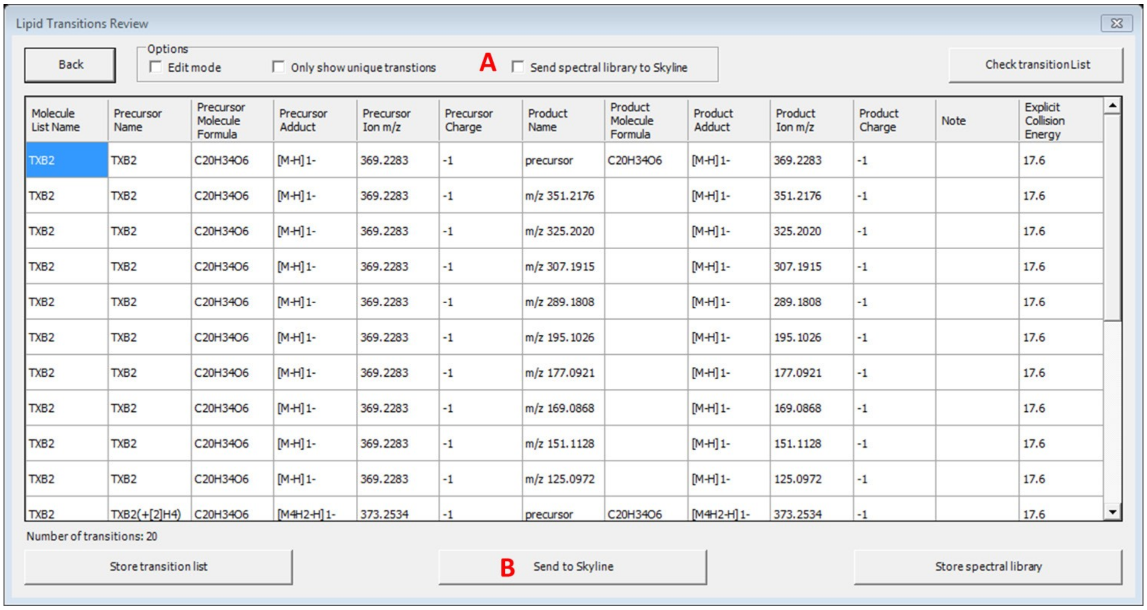

Molecule List Name	Precursor Name	Precursor Molecule Formula	Precursor Adduct	Precursor Ion m/z	Precursor Charge	Product Name	Product Molecule Formula	Product Adduct	Product Ion m/z	Product Charge	Note	Explicit Collision Energy
TXB2	TXB2	C20H34O6	[M-H]1-	369.2283	-1	precursor	C20H34O6	[M-H]1-	369.2283	-1		17.6
TXB2	TXB2	C20H34O6	[M-H]1-	369.2283	-1	m/z 351.2176		[M-H]1-	351.2176	-1		17.6
TXB2	TXB2	C20H34O6	[M-H]1-	369.2283	-1	m/z 325.2020		[M-H]1-	325.2020	-1		17.6
TXB2	TXB2	C20H34O6	[M-H]1-	369.2283	-1	m/z 307.1915		[M-H]1-	307.1915	-1		17.6
TXB2	TXB2	C20H34O6	[M-H]1-	369.2283	-1	m/z 289.1808		[M-H]1-	289.1808	-1		17.6
TXB2	TXB2	C20H34O6	[M-H]1-	369.2283	-1	m/z 195.1026		[M-H]1-	195.1026	-1		17.6
TXB2	TXB2	C20H34O6	[M-H]1-	369.2283	-1	m/z 177.0921		[M-H]1-	177.0921	-1		17.6
TXB2	TXB2	C20H34O6	[M-H]1-	369.2283	-1	m/z 169.0868		[M-H]1-	169.0868	-1		17.6
TXB2	TXB2	C20H34O6	[M-H]1-	369.2283	-1	m/z 151.1128		[M-H]1-	151.1128	-1		17.6
TXB2	TXB2	C20H34O6	[M-H]1-	369.2283	-1	m/z 125.0972		[M-H]1-	125.0972	-1		17.6
TXB2	TXB2(+[2]H4)	C20H34O6	[M4H2-H]1-	373.2534	-1	precursor	C20H34O6	[M4H2-H]1-	373.2534	-1		17.6

Figure T34. Interface for reviewing calculated lipid transition with collision energy on Skyline platform.

A. This check box allows you to send spectral library to Skyline without additionally save *.blib file at local drive.

B. By clicking on this button, transitions (and spectral library) will be sent to Skyline.

5. Export MS method from Skyline

The final MS method can be generated from Skyline by selecting favored MS vendor/type.

6. Viewing LipidCreator log messages in Skyline

LipidCreator displays warnings and errors directly to the user as dialogs. However, detailed error messages are also logged to the Tools→Immediate Window in Skyline and into a log file "lipidcreator.log" below the Tools\LipidCreator\data directory of the Skyline installation.

7. Review PRM/DIA results with spectral matching

To review transitions including precursor, please set Transition setting→Filter→Ion types: f,p.

Select *in-silico* spectral library through Settings→Peptide Settings→Library. Then click on Library Match from View menu. After import data, extracted MS2 spectral through click on chromatographic peak.

T15. Statistics for LipidCreator launching

When initially launching LipidCreator, a dialog will appear with the following message:

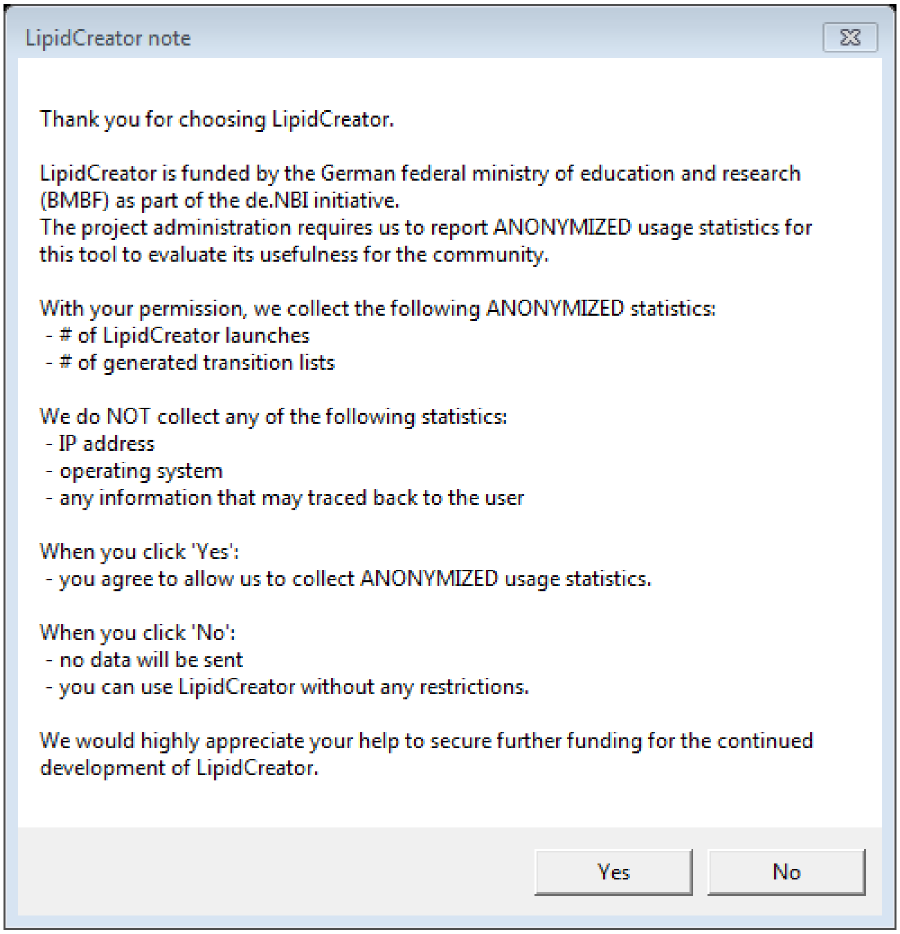

Figure T35. Note when launching LipidCreator for the first time.

If the user changes his/her mind after clicked on ‘Yes’ or ‘No’, this function can be turned off/on at any time by (de)selecting Options→ ‘Send anonymous statistics’.

T16. Command line usage

LipidCreator has a comprehensive command line interface allowing the user to run several tasks without a graphical user interface. Therefore, it is suitable for an integration into automated pipeline frameworks capable of adding customized processing nodes[1,2] or running command line executions[3]. Launching LipidCreator from the command line with the argument “help” prints the following output:

```
> .\LipidCreator.exe  help
usage: LipidCreator.exe (option)

options are:
  dev:                  launching LipidCreator as  developer
  transitionlist:       creating transition list from lipid list
  translate:            translating a list with old lipid names into
                        current nomenclature
  library:              creating a spectral library in *.blib format
                        from a lipid list
  random:               generating a random lipid name (not
                        necessarily reasonable in terms of chemistry)
  agentmode:            secret agent mode
```

The options ‘transitionlist’, ‘translate’ and ‘library’ are the most common modes.

---

[1] Berthold, Michael R., et al. "KNIME-the Konstanz information miner: version 2.0 and beyond." AcM SIGKDD explorations Newsletter 11.1 (2009): 26-31.

[2] Goecks, Jeremy, Anton Nekrutenko, and James Taylor. "Galaxy: a comprehensive approach for supporting accessible, reproducible, and transparent computational research in the life sciences." Genome biology 11.8 (2010): R86.

[3] Köster, Johannes, and Sven Rahmann. "Snakemake—a scalable bioinformatics workflow engine." Bioinformatics 28.19 (2012): 2520-2522.

Creating a transition list

A transition list contains all necessary information about the precursor name / mass / charge / adduct in combination with its fragment name / mass / charge / adduct / (collision energy). To create a transition list, a lipid list is necessary. An 'example-lipid-list.csv' can be found within the LipidCreator root directory in the folder 'data\examples'. The 'transitionlist' mode has the following options:

```
> .\LipidCreator.exe transitionlist help
Creating a transition list from a lipid list

usage: LipidCreator.exe transitionlist input_csv output_csv [opts
[opts ...]]
  opts are:
    -p 0:               Compute no precursor transitions
    -p 1:               Compute only precursor transitions
    -p 2:               Compute with precursor transitions
    -h 0:               Compute no heavy labeled isotopes
    -h 1:               Compute only heavy labeled isotopes
    -h 2:               Compute with heavy labeled isotopes
    -s:                 Split in positive and negative list
    -x:                 Developer or Xpert mode
    -l:                 Create LipidCreator project file instead
                        of transition list
    -d:                 Delete replicate transitions (equal
                        precursor and fragment mass)
    -c instrument mode: Compute with optimal collision energy (not
                        available for all lipid classes)
```

Optionally, several options (e.g. precursor or heavy labeled fragments) can be set or collision energy optimization can be enabled. A command for creating a transition list without replicates and heavy labeled isotope fragments (if by default included in LipidCreator) looks like this:

```
> .\LipidCreator.exe transitionlist data\examples\example-lipid-
list.csv output-transition-list.csv -h 0 -d
```

The resulting transition list file can be further imported in follow-up analysis tools such as Skyline.

Translating lipid names

Several different structural nomenclatures for lipid names exist. Especially, many legacy names exist for the same lipid species. This results in problems when parsing lipid lists in tools which only have one nomenclature implemented. To solve this issue, a lipid name translation engine was developed to recognize old and deprecated lipid names (e.g. as still used by LIPID MAPS)

to translate them into lipid species names according to the current nomenclature[4]. To translate lipid lists into the current nomenclature, the user has to type in:

```
> .\LipidCreator.exe translate data\examples\old-lipid-name-list.csv new-nomenclature-lipid-list.csv
```

The old lipid names will be preserved in the new list. The recognition system is far from being complete, so we encourage all users who encounter issues with the translator to send us[5] their lipid lists. Such feedback allows us to extend the translation engine further.

Creating a spectral library

It is possible to create an *in-silico* spectral library in *.blib format with the command line interface. To estimate the relative fragment abundances, true MS^2 measurements were used to train parameterized statistical models. Although we measured different lipid species with different adducts on different platforms, we do not guarantee correctness of the estimated spectra or generalizability to arbitrary platforms. To create a spectral library for lipids, a lipid list (*.csv) or LipidCreator project file (*.lcXML) as well as the PSI-MS controlled vocabulary term[6] of the measurement platform is required. For example, the user has to type:

```
> .\LipidCreator.exe library data/examples/example-lipid-list.csv
output-library.blib MS:1002523
```

Here, ‘MS:1002523’ is the CV term for the instrument ‘Thermo Scientific Q Exactive HF’. The created spectral library can be now easily imported in follow-up analysis tools such as Skyline[7].

[4] Pauling, Josch K., et al. "Proposal for a common nomenclature for fragment ions in mass spectra of lipids." PloS one 12.11 (2017): e0188394.

[5] https://lifs.isas.de/

[6] https://www.ebi.ac.uk/ols/ontologies/ms

[7] MacLean, Brendan, et al. "Skyline: an open source document editor for creating and analyzing targeted proteomics experiments." Bioinformatics 26.7 (2010): 966-968.

T17. Support for additional platforms for CE Optimization

Due to slight differences in the way that vendor data is transformed, and specifically, how activation/collision dissociation details are reported in *.mzML, an adaptation of the existing codebase may be necessary.

Please request access to the necessary tools and support at https://lifs.isas.de/support.html if you want to add data for a specific new platform or if you wish to use your own reference data for an existing platform. The following sections describe the tables that are required for a) LipidCreator to read/use a custom model, and b) the tables used by flipR as input and those produced by it for import in LipidCreator. The required projects are available at https://github.com/lifs-tools/flipr.

T15.1 MS Instrument Table

The comma-separated MS instrument table for LipidCreator (data\ms-instruments.csv) contains PSI-MS controlled vocabulary terms for instrument model to identify individual MS platforms on each row. The following columns are required in this file:

- **CV_term**: the PSI-MS CV term id identifying the instrument.
- **model**: the PSI-MS CV term's description, e.g. the name of the instrument (term MS:1002523, 'Q Exactive HF').
- **min_CE**: the minimum collision energy covered by the instrument. Collision energies calculated by LipidCreator can not be lower than this threshold. The model calculated by flipR may exceed the range set by min_CE and max_CE.
- **max_CE**: the maximum collision energy covered by the instrument. Collision energies calculated by LipidCreator can not be higher than this threshold. The model calculated by flipR may exceed the range set by min_CE and max_CE.
- **x axis label**: the label for the collision energy axis, e.g. 'Collision Energy [eV]' or 'Relative Collision Energy'.
- **modes**: the modes to enable for the instrument platform, e.g. 'PRM' for PRM-only mode.

Example ('…' represent skipped lines in the file):

```
CV_term,model,min_CE,max_CE,x                    axis                       label,modes
...
MS:1002523,Thermo Scientific Q Exactive HF,10,60,Normalized Collision Energy,PRM
```

To feed data into the fragment intensity prediction (FIP) pipeline, MS/MS data needs to be available in *.mzML format. We recommend msConvert for the conversion from native vendor format into *.mzML.

msConvert may not always report the most precise term for the instrument, but rather a more generic term that identifies the instrument family.

A list of the available instruments is available via the OntologyLookupService[8].

T15.2 Transition Table

The transition table used for the CE model calculation can be generated using LipidCreator, similarly to how a transition list is created for Skyline. However, LipidCreator uses specific internal IDs for heavy labeled species, which requires the "developer" mode to create modified transition lists that allow a reimport of the parameter files after the parameter estimation step.

You can start LipidCreator from the command line in developer mode as follows:

```
LipidCreator.exe dev
```

This will ensure that precursor names for heavy-labeled instances are written with a specific placeholder, e.g. "{d8}" for 8 Deuterium atoms replacing 8 hydrogens. This allows the correct mapping of the corresponding model parameters in LipidCreator. Adduct names for heavy labeled instances are also exported unaltered in developer mode, not with the Skyline specific nomenclature, e.g. [M8H2-H]1-, to ensure correct mapping to the originating monoisotopic precursor. Precursor and product masses are correctly calculated for heavy labeled instances. The following columns are required in this file (following the format for Skyline transition lists[9]):

- **MoleculeGroup**
- **PrecursorName**
- **PrecursorFormula**
- **PrecursorAdduct**
- **PrecursorMz**
- **PrecursorCharge**
- **ProductName**

[8] https://www.ebi.ac.uk/ols/ontologies/ms/terms?iri=http%3A%2F%2Fpurl.obolibrary.org%2Fobo%2FMS_1000031

[9] https://skyline.ms/_webdav/home/software/Skyline/%40files/tutorials/SmallMolecule-3_6.pdf

- **ProductFormula**
- **ProductAdduct**
- **ProductMz**
- **ProductCharge**
- **Note**

Example ('…' represent skipped lines in the file):

```
MoleculeGroup PrecursorName PrecursorFormula     PrecursorAdduct      PrecursorMz
      PrecursorCharge      ProductName   ProductFormula      ProductAdduct ProductMz
      ProductCharge Note
…
PIP2   PIP2 17:0-20:4      C46H83O19P3   [M-H]1-      1031.46686541991    -1
      precursor    C46H83O19P3   [M-H]1-      1031.46686541991    -1
```

Please follow the transition list tutorial for LipidCreator to create a transition list for your target molecules (see Generating lipid molecule transitions). When you reach the step "LipidsReview", select "Store transition list", choose "tsv files (*.tsv)" as your output file format and select "No" when asked whether to split the output by polarity.

T15.3 Mapping Table (Transitions to measurements)

This tab-separated table defines the mapping from transitions to actual measurements and acts as the glue between LipidCreator transitions lists and the transition extraction and model training with flipR.

- **Instrument:** the PSI-MS CV term for the instrument, e.g. 'MS:1002523'.
- **MoleculeGroup:** the molecule group / class of the molecule, e.g. 'PIP2'.
- **PrecursorName:** the name of the precursor molecule, e.g. 'PIP2 17:0-20:4'.
- **PrecursorAdduct:** the precursor adduct, e.g. '[M-H]1-'.
- **PPMS:** a '|' (bar) separated list of ppms to use for transition / m/z matching, e.g. '5|10'.
- **File:** the source file for this instance, containing MS2 scans.
- **Group:** a group identifier to distinguish multiple measurements of the same molecule.

Example ('…' represent skipped lines in the file):

```
Instrument     MoleculeGroup PrecursorName  PrecursorAdduct        PPMS    File    Group
…
MS:1002523     PIP2    PIP2 17:0-20:4 [M-H]1- 5|10    measurements/QExHF03 NM 0001427.mzML 0001427
```

T15.4 Feature Table (Transitions applied to mzMLs)

The feature table (*-fip.tsv) is created by the transition extraction step and serves as the main data input for flipR. It holds one m/z feature per row, with the following columns, specifying its provenance, parameters and information that is used by downstream steps to maintain a mapping between input transition list and output parameter file for LipidCreator. The following columns are reported in the output file (some correspond to *.mzML elements / attributes, some come from LipidCreator / Skyline):

- **instrument**
- **localDateTimeCreated**
- **origin**
- **scanNumber**
- **polarity**
- **basePeakMz**
- **basePeakIntensity**
- **totalIonCurrent**
- **id**
- **scanDefinition**
- **msLevel**
- **isolationWindowTargetMz[0]**
- **isolationWindowLowerOffset[0]**
- **isolationWindowUpperOffset[0]**
- **precursorActivationType**
- **precursorCollisionEnergy**
- **precursorCollisionEnergyUnit**
- **ionInjectionTime[0]**
- **isolationMzMin[0]**
- **isolationMzMax[0]**
- **precursorCharge[0]**
- **precursorMz[0]**
- **msFunction**

- **retentionTime**
- **spectrumType**
- **rawTic**
- **group**
- **foundMass**
- **foundMassRange[ppm]**
- **foundMassLowerBound**
- **foundMassUpperBound**
- **foundMassError[ppm]**
- **foundIntensity**
- **scanRelativeIntensity**
- **calculatedMass**
- **species**
- **precursorAdduct**
- **fragment**
- **adduct**

Example (some columns omitted for brevity):

```
instrument	localDateTimeCreated	origin	scanNumber	polarity	basePeakMz
	basePeakIntensity	totalIonCurrent	…	isolationWindowTargetMz[0]
	isolationWindowLowerOffset[0]	isolationWindowUpperOffset[0]
	precursorActivationType	precursorCollisionEnergy
	precursorCollisionEnergyUnit	…	rawTic	group	foundMass
	foundMassRange[ppm]	foundMassLowerBound	foundMassUpperBound	foundMassError[ppm]
	foundIntensity	scanRelativeIntensity	calculatedMass	species
	precursorAdduct	fragment	adduct
MS:1002523	2018-11-21T08:02:11.851	QExHF03_NM_0001279.mzML	1	NEGATIVE
	317.2117523	1.8311354e07	2.737138e07	…	317.212188720703	0.25	0.25
	HCD	10.0	electronvolt	…	2.6918232E7	0001279
	299.2013854980469	5	299.19990399299996	299.20289600700005	-
0.04846886780363687	567272.8	0.021073926	299.2014	9-HEPE	[M-H]1-
	299.201	[M-H]1-
```

S.2.15.5 LipidCreator Parameter Table (After model training and selection)

The comma-separated lipid creator parameter file (data\ce-parameters\MS_CVTERMID.csv, e.g. **MS_1002523.csv** for the Thermo Scientific Q Exactive HF) contains collision energy

calculation parameters for each lipid class, as reported and concatenated by flipR. There are as many rows for each fragment, as there are parameters. The following columns are required in this file:

- **instrument:** the PSI-MS CV term id identifying the instrument, e.g. MS:**1002523 for** Thermo Scientific Q Exactive HF.
- **class:** the lipid class, e.g. 10-HDoHE, needs to be double quoted, when the name contains a comma.
- **adduct:** the precursor adduct for this lipid class, e.g. [M-H]1-.
- **fragment:** the fragment identifier. If no common name is available, use e.g. “m/z 121.0658”. The precursor must be reported as “precursor”.
- **ParKey:** the model parameter name, currently one of “model”, “meanlog”, “sdlog”, “scale”, and “shift”.
- **ParValue:** the model parameter values, currently, for “model” only “dlnormPar” is recognized. Other parameters are expected to be reported as double numbers with a “.” as the decimal separator.

Example (‘…’ represent skipped lines in the file):

```
instrument,class,adduct,fragment,ParKey,ParValue
…
MS:1002523,10-HDoHE,[M-H]1-,m/z 121.0658,meanlog,4.10313116901712
MS:1002523,10-HDoHE,[M-H]1-,m/z 121.0658,model,dlnormPar
MS:1002523,10-HDoHE,[M-H]1-,m/z 121.0658,scale,0.140234378276546
MS:1002523,10-HDoHE,[M-H]1-,m/z 121.0658,sdlog,0.512948788298359
MS:1002523,10-HDoHE,[M-H]1-,m/z 121.0658,shift,2.97212321271515
```
